# A Comprehensive Systems Biology Approach to Studying Zika Virus

**DOI:** 10.1371/journal.pone.0161355

**Published:** 2016-09-01

**Authors:** Meghan May, Ryan F. Relich

**Affiliations:** 1 Department of Biomedical Sciences, University of New England College of Osteopathic Medicine, Biddeford, Maine, United States of America; 2 Seacoast Biomedical Science Institute, York, Maine, United States of America; 3 Department of Pathology and Laboratory Medicine, Indiana University School of Medicine, Indianapolis, Indiana, United States of America; Centers for Disease Control and Prevention, UNITED STATES

## Abstract

Zika virus (ZIKV) is responsible for an ongoing and intensifying epidemic in the Western Hemisphere. We examined the complete predicted proteomes, glycomes, and selectomes of 33 ZIKV strains representing temporally diverse members of the African lineage, the Asian lineage, and the current outbreak in the Americas. Derivation of the complete selectome is an ‘omics’ approach to identify distinct evolutionary pressures acting on different features of an organism. Employment of the M8 model did not show evidence of global diversifying selection acting on the ZIKV polyprotein; however, a mixed effect model of evolution showed strong evidence (*P*<0.05) for episodic diversifying selection acting on specific sites. Single nucleotide polymorphisms (SNPs) were predictably frequent across strains relative to the derived consensus sequence. None of the 9 published detection procedures utilize targets that share 100% identity across the 33 strains examined, indicating that ZIKV escape from molecular detection is predictable. The predicted O-linked glycome showed marked diversity across strains; however, the N-linked glycome was highly stable. All Asian and American strains examined were predicted to include glycosylation of E protein ASN_154_, a modification proposed to mediate neurotropism, whereas the modification was not predicted for African strains. SNP diversity, episodic diversifying selection, and differential glycosylation, particularly of ASN_154_, may have major biological implications for ZIKV disease. Taken together, the systems biology perspective of ZIKV indicates: a.) The recently emergent Asian/American N-glycotype is mediating the new and emerging neuropathogenic potential of ZIKV; and b.) further divergence at specific sites is predictable as endemnicity is established in the Americas.

## Introduction

Zika virus (ZIKV) is a mosquito-borne pathogen that has recently emerged in the Western Hemisphere. It was discovered in 1947 in a sentinel rhesus macaque placed in the Zika Forest of Uganda at a virological research station; however, its role as a human pathogen was not revealed until 1953 [1, 2]. Prior to its detection in the State of Bahia, Brazil in March, 2015, ZIKV remained largely obscure despite causing several outbreaks of acute febrile disease in parts of Africa, Asia, and Oceania. Since its estimated introduction to continental South America in 2013, widespread autochthonous transmission has been reported in over forty countries in Central and South America as well as the Caribbean [3, 4].

ZIKV is a member of the genus *Flavivirus*, which is 1 of 4 genera comprising the family *Flaviviridae*. ZIKV and its phylogenetically closest relative Spondweni virus form the Spondweni virus group; both of which are known to infect humans [5, 6]. ZIKV virions are enveloped by a host cell-derived lipid bilayer membrane. Anchored in the membrane are glycoproteins involved in adsorption to and infection of host cells, and underlying the membrane is the viral nucleocapsid [7]. The ZIKV genome is comprised of a linear, monopartite, single-stranded, positive-sense RNA molecule that is approximately 10.8 kb long and consists of a single large open reading frame encoding 3 structural proteins (the capsid protein [C], the premembrane/membrane protein [prM], and the envelope glycoprotein [E]) and 7 nonstructural proteins (NS1, NS2A, NS2B, NS3, NS4A, NS4B, and NS5). The translated polyprotein is cleaved into individual proteins by both viral and cellular enzymes [8–10]. Sequencing of the ZIKV NS3 genes from a number of isolates have identified three genetically distinct lineages: East African, West African, and Asian lineages. Characterization of strains circulating in the Western Hemisphere indicate that the original virus introduced to South America was an Asian-lineage virus [11–13].

ZIKV is transmitted in urban and suburban human-mosquito-human cycles primarily by *Aedes aegypti*, but *Aedes albopictus* has also been implicated as a vector in human ZIKV outbreaks [14]. A sylvatic cycle similar to that of yellow fever virus, a relative of ZIKV, has been identified in Africa where the virus circulates between nonhuman primates and *Aedes* spp. mosquitoes. A sylvatic cycle has yet to be formally identified in Asia or the Americas; however, a recent preprint report demonstrated ZIKV RNA in sera from wild marmosets and capuchin-monkeys in Brazil [15]. The potential for or documentation of additional routes of ZIKV transmission, including perinatal, blood transfusion-associated, and sexual transmission, have been described in the literature [16–22].

Human infection with ZIKV results in symptomatic infection in approximately 20% of those infected and the virus has an as-of-now poorly defined incubation period, which is believed to be anywhere from a few days to a week or longer following infection [21]. Symptomatic patients most commonly exhibit one or more of the following: arthralgia, myalgia, nonpurulent conjunctivitis, headache, malaise, and rash. Virtually all cases are self-limited and disease duration is generally short [3, 23]; however, birth defects (microcephaly) and neurological conditions (Guillain-Barré syndrome, encephalopathy) associated with ZIKV infection have been reported in French Polynesia and the Americas [24–29]. To date, few Zika fever fatalities have been recorded, but two such occurrences were reported recently: one in a 15-year-old Colombian female with sickle cell disease and concurrent ZIKV infection [30], and another in a 70-year-old Puerto Rican male who developed severe thrombocytopenic purpura as a rare complication of ZIKV infection [31].

The rapid emergence of ZIKV in the Western hemisphere highlighted the lack of biological and clinical understanding about this virus, and created a sudden need for comprehensive approaches to facilitate control and ultimately treatment and prevention strategies. Here we present a systems biology analysis of ZIKV examining diversity in its sequence and glycosylation patterns, and the reflection of this diversity in its selectome, phylogenomics, molecular disease surveillance, and potentially its clinical presentation.

## Results

### Sequence Diversity Analysis

**Fig 1:**
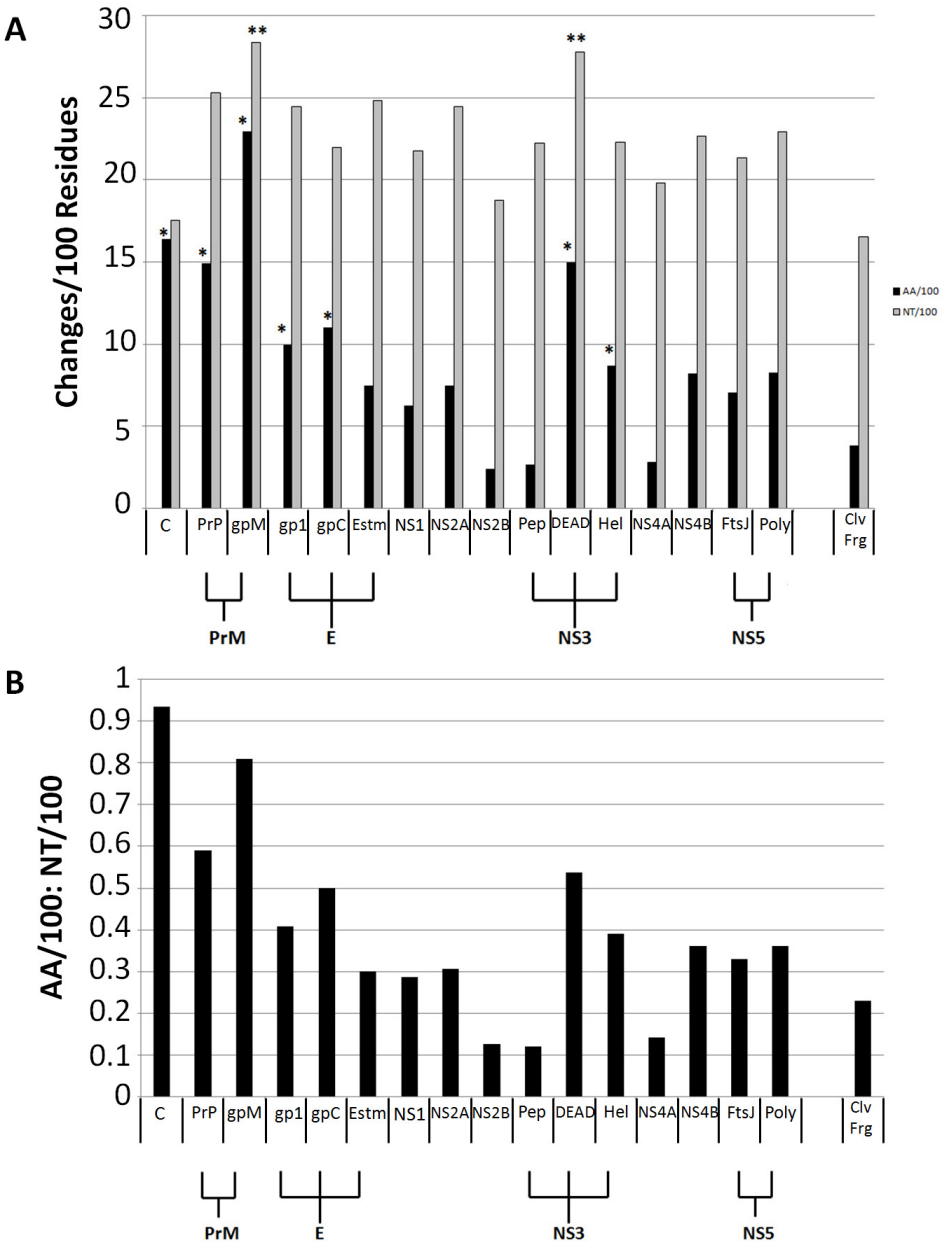
Sequence Diversity Across ZIKV Strains. (A) Average nucleotide diversity (changes per 100 residues relative to consensus; grey bars) varied significantly (** *P*<0.05 across strains). Average amino acid diversity changes per 100 residues relative to consensus; black bars) also varied significantly (* *P*<0.05 across strains). (B) The ratio of amino acid changes per 100 residues to nucleotide changes per 100 residues also varied across strain. Individual domains within certain proteins are represented as indicated. Abbreviations used in this Fig are as follows: C-Capsid; PrP-Propeptide; gpM-Glycoprotein M; gp1-Glycoprotein 1; Glycoprotein C; Estm-E Stem; Pep-NS3 peptidase; DEAD-DEAD NTPase; Hel-Helicase; FtsJ-FtsJ domain; Poly-Polymerase; Clv Frg-Cleavage fragments.

**Fig 2:**
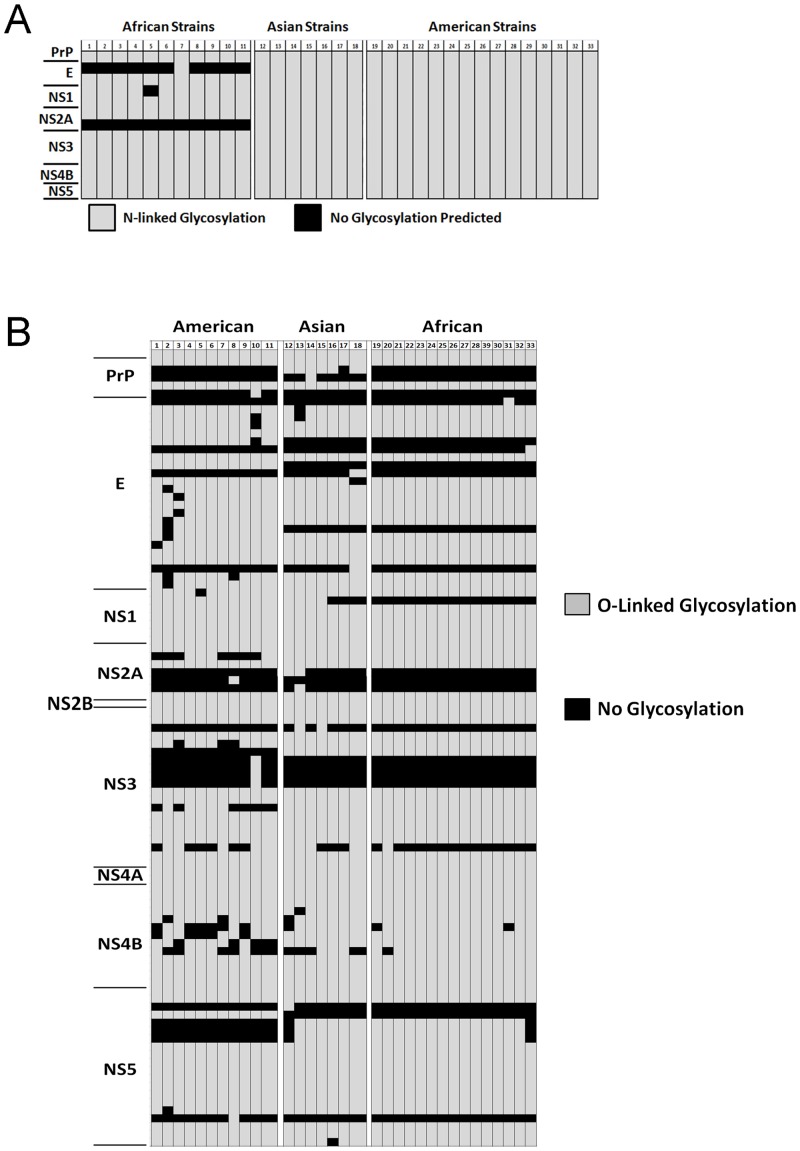
N- and O-Linked Glycotypes of ZIKV Isolates. (A) N-linked glycosylation predictions (grey fill) for each strain (columns) were homologous across strains with the exception of E ASN_154_ and NS2A ASN_148_ (black fill). (B) O-linked glycosylation was highly variable across strains, with glycosylated residues represented by grey fill and non-glycosylated residues represented by black fill. Abbreviations used in this Fig are as follows: C-Capsid; PrP-Propeptide; gpM-Glycoprotein M; gp1-Glycoprotein 1; Glycoprotein C; Estm-E Stem; Pep-NS3 peptidase; DEAD-DEAD NTPase; Hel-Helicase; FtsJ-FtsJ domain; Poly-Polymerase; Clv Frg-Cleavage fragments.

Numerous single nucleotide polymorphisms (SNPs) with respect to the consensus sequence were observed throughout the polyprotein-encoding. The mean number of substitutions per 100 bp across the ZIKV genome was 22.5. The mean number of substitutions per 100 bp within each individual protein-coding sequence ranged from 17.5 for the capsid to 28.3 for the surface glycoprotein gpM (Fig 1A). Rates of amino acid substitution per 100 residues were predictably approximately three-fold lower (mean across sequences = 9.1), but were not uniform across protein sequences. Amino acid substitutions rates ranged from 2.38 for NS2B to 22.97 for gpM (Fig 1A). Ratios of nucleotide to amino acid changes per 100 residues varied considerably between proteins (0.12 for the peptidase NS3 to 0.93 for the capsid) (Fig 1B). Short, in-frame insertions or deletions relative to consensus were detected in strains MR_766, IbH30656, ArB13565, ArD158084, ArD1362, ArD157995, CPC-0740, and ArB15076. N-linked glycosylation sites were completely conserved between Asian and American isolates, while most African isolates exhibited a different N-glycotype. Two African isolates, ArD41519 and ArB15076, had unique N-glycotypes not shared by any other strain examined (Fig 2A). O-linked glycosylation sites were far more diverse across strains, with 19 distinct O-glycotypes predicted (Fig 2B). No intrinsically disordered regions were predicted for the capsid protein, propeptide, glycoprotein M, NS2A, NS4A, and NS4B, and focused disordered regions were predicted for envelope protein E, NS1, NS2B, NS3, and NS5 (S1 Fig). All raw data are available in Supporting Information. Sequences utilized in this study are available in GenBank (Table 1).

**Table 1:** Zika Virus Strain Information.

Strain	ID^a^	Clade	Site of Isolation	Year of Isolation	Source of Isolation	Accession	Reference
MR_766	1	AF	Uganda	1947	Macaque	NC_012532	51
IbH30656	2	AF	Nigeria	1968	Human blood	HQ234500	50
ArD7117	3	AF	Senegal	1968	*Aedes luteocephalus*	KF383116	11
ARB7701	4	AF	CAR	1976	*Aedes africanus*	KF268950	47
ARB15076	5	AF	CAR	1978	*Aedes opok*	KF268949	47
ARB13565	6	AF	CAR	1980	*Aedes africanus*	KF268948	47
ArD41519	7	AF	Senegal	1984	*Aedes africanus*	HQ234501	50
ArD12800	8	AF	Senegal	1997	*Aedes luteocephalus*	KF383117	11
ArD158084	9	AF	Senegal	2001	*Aedes dalzieli*	KF383119	11
ArD157995	10	AF	Senegal	2001	*Aedes dalzieli*	KF383118	32
ArB1362	11	AF	Senegal	2001	*Aedes africanus*	KF383115	11
P6-740	12	AS	Malaysia	1966	*Aedes aegypti*	HQ234499	50
Yap2007	13	AS	Micronesia	2007	Human blood	EU545988	52
Fss13025	14	AS	Cambodia	2010	Human blood	JN860885	50
CPC-0740	15	AS	Philippines	2012	Human blood	KU681082	46
PLCal_zv	16	AS	Canada^b^	2013	Human urine	KF993678	49
H/PF/2013	17	AS/AM	F. Polynesia	2013	Human blood	KJ776791	45
SV0127	18	AS	Thailand	2014	*Aedes albopictus*	KU681081	46
Haiti2014	19	AM	Haiti	2014	Human blood	KU509998	53
SSABr1	20	AM	Brazil	2015	Human blood	KU707826	46
OPY_Martinique	21	AM	Martinique	2015	Human blood	KU647676	46
Natal RGN	22	AM	Brazil	2015	Fetal brain	KU527068	27
103344	23	AM	Guatemala	2015	Human blood	KU501217	32
8375	24	AM	Guatemala	2015	Human blood	KU501216	32
PRVABC59	25	AM	Puerto Rico	2015	Human blood	KU501215	32
BeH815744	26	AM	Brazil	2015	Human blood	KU365780	46
BeH819966	27	AM	Brazil	2015	Human blood	KU365779	46
BeH819015	28	AM	Brazil	2015	Human blood	KU365778	46
BeH818995	29	AM	Brazil	2015	Human blood	KU365777	46
Z1106033	30	AF	Suriname	2015	Human blood	KU312312	13
ZikaSPH2015	31	AM	Brazil	2015	Human blood	KU321639	48
Brazil-Zikv2015	32	AM	Brazil	2015	Amniotic fluid	KU497555	46
GD01	33	AM	China^c^	2016	Human blood	KU740184	46

^a^ID numbers reported were generated for this study and are used in subsequent tables.

^b^This strain was isolated in Canada from a traveler returning from the Philippines, and is therefore considered an Asian strain.

^c^This strain was isolated in China from a traveler returning from South America, and is therefore considered an American strain.

### Diagnostic Reagent Diversity

**Fig 3:**
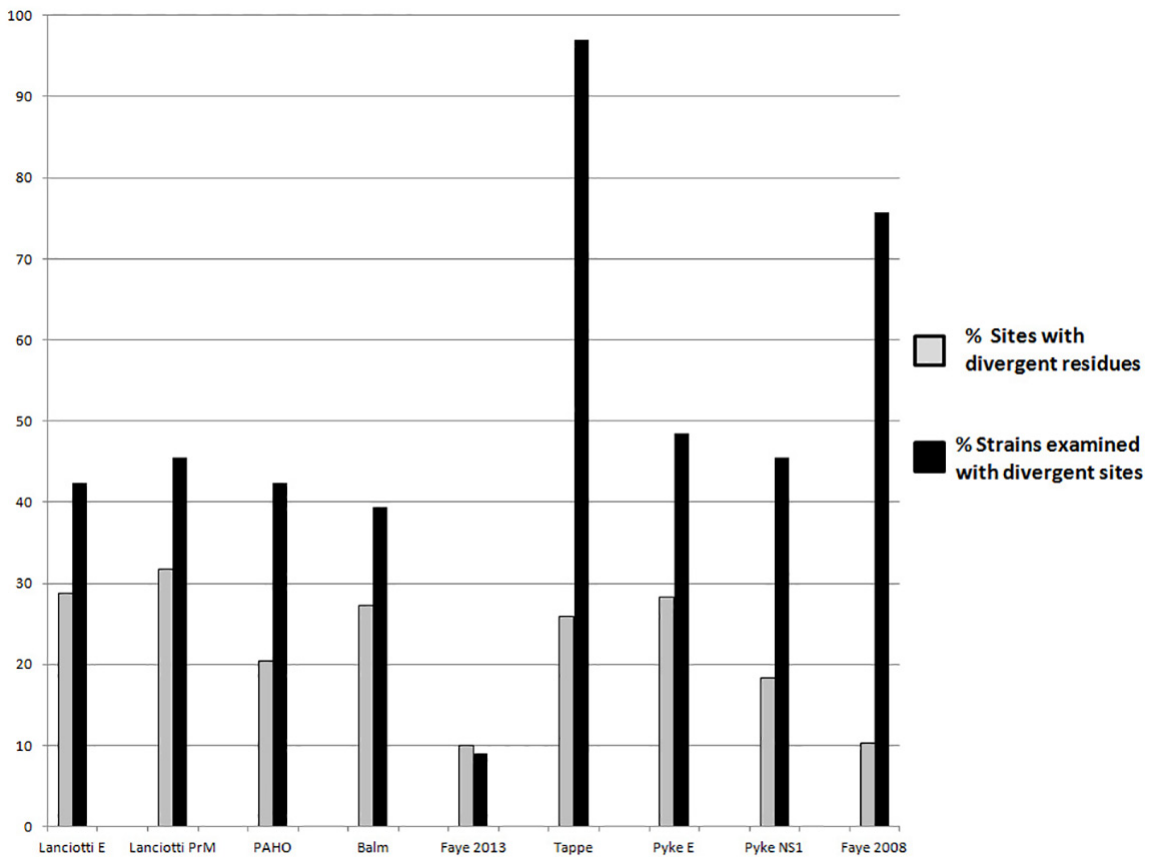
Nucleotide Conservation across Diagnostic Reagents. PCR primers and probes were aligned with 33 ZIKV genomes to explore their fidelity. Diagnostic reagents all had >10% divergence across residues (grey bars). The proportion of strains examined in this study that had at least one mismatch from diagnostic reagents ranged from from <10% to >95% (black bars).

No diagnostic methods examined in this study displayed 100% identity across the 33 strains examined, even accounting for degeneracy (Table 2). The proportion of divergent residues ranged from 10% for ‘Faye2013’ to 31.67% for ‘Lanciotti PrM’. When examining the proportion of examined strains with at least one divergent residue from a methods’ reagents, the percent divergence ranged from 9.09% for ‘Faye2013’ to 96.97% for ‘Tappe’ (Fig 3).

**Table 2:** Diagnostic Test Divergence by Strain^a^.

Strain	Clade^b^	Lanciotti M	Lanciotti E	Pyke E	Pyke NS1	PAHO NS2	Faye NS5	Balm NS5	Faye E	Tappe NS3
MR_766	AF	5	4	9	6	7	✓	✓	✓	✓
P6-740	AS	3	1	2	2	✓	✓	✓	1	5
Yap2007	AS	✓	✓	✓	✓	✓	✓	✓	1	4
IbH30656	AF	6	2	6	✓	7	✓	3	2	4
ArD_41519	AF	6	5	6	5	6	✓	4	✓	2
Fss13025	AS	✓	1	2	4	✓	✓	✓	1	4
CPC-0740	AS	1	1	✓	1	1	✓	1	1	6
GD01	AM	✓	1	1	✓	✓	✓	✓	1	7
SV0127	AS	1	1	✓	✓	✓	✓	3	2	6
Brazil-Zikv2015	AM	✓	1	✓	✓	✓	✓	✓	1	6
SSABr1	AM	✓	1	✓	✓	✓	✓	✓	1	7
OPY_Martinique	AM	✓	1	1	1	✓	✓	✓	✓	7
Natal RGN	AM	✓	1	✓	✓	✓	✓	1	1	7
Haiti2014	AM	✓	1	✓	✓	✓	✓	✓	1	7
103344	AM	✓	1	✓	✓	✓	✓	✓	1	7
8375	AM	✓	1	✓	✓	✓	✓	✓	1	5
PRVABC59	AM	✓	1	✓	✓	✓	✓	✓	1	7
BeH815744	AM	✓	1	✓	✓	✓	✓	✓	✓	7
BeH819966	AM	✓	1	✓	✓	✓	✓	✓	✓	7
BeH819015	AM	✓	1	✓	✓	1	✓	✓	✓	7
BeH818995	AM	✓	1	✓	✓	✓	✓	✓	✓	7
Z1106033	AF	✓	1	✓	✓	✓	✓	✓	✓	6
ZikaSPH2015	AM	✓	1	✓	✓	✓	✓	✓	1	7
ARB7701	AF	4	7	8	5	✓	5	3	1	3
ARB15076	AF	3	5	9	4	✓	✓	3	1	2
ARB13565	AF	2	5	11	6	✓	5	3	1	3
ArD158084	AF	5	4	10	6	✓	✓	1	✓	✓
ArD157995	AF	10	11	12	6	✓	✓	8	✓	1
ArD12800	AF	7	3	10	6	✓	✓	3	✓	3
ArD7117	AF	6	4	7	5	✓	✓	3	✓	2
ArB1362	AF	5	5	9	7	✓	1	9	✓	2
PLCal_zv	AS	1	1	✓	✓	✓	✓	✓	1	6
HPF/2013	AS/AM	✓	1	✓	✓	✓	✓	✓	1	6

^a^The number of divergent sites in each strain for all primers and/or probes of each published diagnostic test are listed (grey shading). Check marks indicate 100% identity within the listed strain in targeted sites.

^b^Clades are abbreviated as follows: Af = African lineage; As = Asian lineage; Am = American lineage

### Phylogenetic Analysis

**Fig 4:**
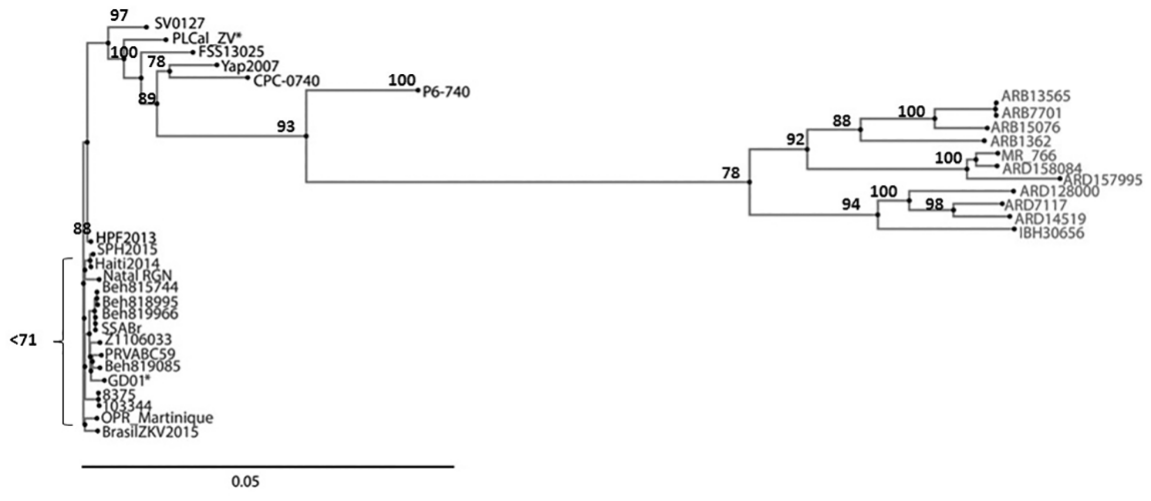
ZIKV Phylogenomics. A Phylogenomic analysis based on the whole polyprotein sequence was generated with neighbor-joining methods. This tree was consistent with previously described isolate relationships based on NS5 and NS3 in resolving African and Asian lineages. The American strains form a distinct group most closely related to Asian strains.

Phylogeny derived from the entire polyprotein sequence is largely consistent with previously described relation based on NS5 or NS3 [5, 11, 32–33]. Examination of the entire encoded proteome reveals that African and Asian strains still largely cluster together, and that the Western hemisphere isolates cluster very tightly into a single clade. A single strain from the Asian clade (H/PF/2013; French Polynesia 2013) groups with the American isolates (Fig 4).

### Evolutionary Selection Analysis

**Fig 5:**
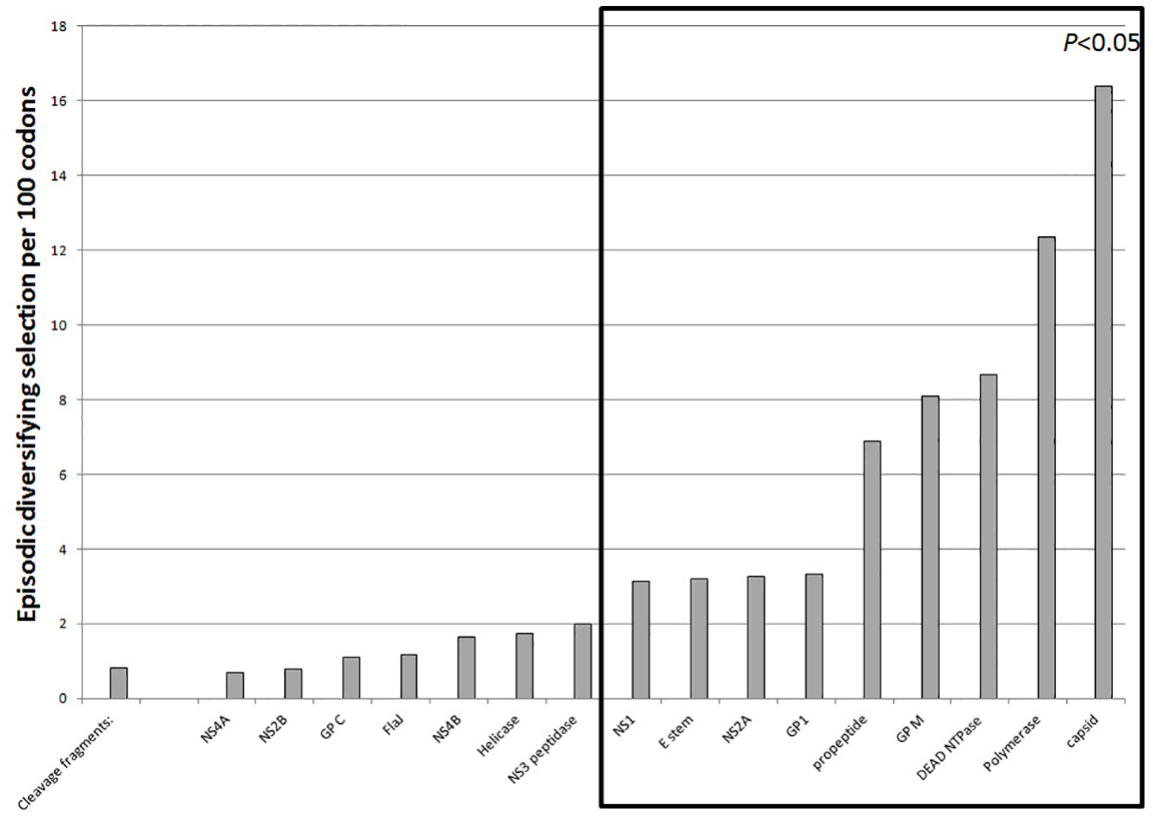
Episodic Diversifying Selection in ZIKV. Individual proteins and/or functional domains varied in the proportion of amino acid sites under episodic diversifying selection. Many proteins or functional domains differed significantly (*P*<0.05; boxed) from the background rate of selection derived from cleavage fragment sequences.

Derivation of the complete selectome is an ‘omics’ approach to identify distinct evolutionary pressures acting on different features of an organism. Global diversifying selection acting on ZIKV proteins was not apparent using the M8 model, despite the rate of point mutations. However, 170 discrete sites were under episodic diversifying selection using the mixed effects model MEME. Diversity was not uniform across proteins, and ranged from 0.70 sites per 100 codons in the NS4A to 16.38 sites per 100 codons in the capsid (Fig 5). A small number of residues under diversifying selection were in predicted to occur in intrinsically disordered regions, but the majority were not. All selection scores are available in our Supporting Information.

## Discussion

The recent emergence of ZIKV in the Western Hemisphere has resulted in major expansions of clinical and molecular data relative to this previously understudied Flavivirus. We examined sequence diversity across 33 strains of ZIKV and commonly detected point mutations across the entire polyprotein-encoding sequence. Insertions and deletions were less common, small in size, and always in-frame. The mean rate of SNPs across the ZIKV genome was 22.5 substitutions per 100 nucleotides. Though higher than previous reports of this measurement in other organisms with DNA genomes, it is comparable to other RNA viruses [34–35]. The mean rate of amino acid substitutions per 100 residues was far lower, at 9.1. Structural predictions revealed at least one intrinsically disordered region in 6 of 11 ZIKV proteins.

Resolution of N- and O-glycotypes for each strain indicated an additional mechanism for generating ZIKV diversity. O-linked glycosylation was highly variable, with most African and Asian strains presenting a completely unique O-glycotype. While American strains were predictably more homogenous, distinct O-glycotypes were displayed by the earliest examined strain (Haiti2014, ID#19-collection date December 2014) and the most recent examined strain (GD01, ID#33-collection date January 2016), suggesting a radiation in O-glycotype diversity is underway. N-linked glycosylation was far more conserved, and all Asian and American strains examined presented a single N-glycotype. Nine of 11 African isolates shared a single N-glycotype. Interestingly, 10 of 11 African strains lacked N-linked glycosylation of ASN_154_ in the E protein, whereas all Asian and American strains were predicted to include this modification. N-linked glycosylation of ASN_154_, which was predicted for ZIKV in this study and confirmed by cryo-electron microscopy for H/PF/2013 (Asian clade; ID#17 in this study) [7], is found in neurotropic flaviviruses such as West Nile virus, and is not predicted for other flaviviruses such as Dengue. ASN_154_ is associated with neuroinvasion [36–37], suggesting that viruses with this particular modification will display a prominent tropism for neurological tissues. ASN_154_ is notably part of an intrinsically disordered region, which have been linked to novel virus/host interactions due to their inherent flexibility [38]. One of the more surprising elements of emergent ZIKV disease in the Western Hemisphere is the association of microcephaly, Guillan-Barré syndrome, and encephalopathies with infection. The question as to whether this association was due to the infrastructural capacity to detect it or a novel element of ZIKV itself has been the subject of extensive debate. While replication of the African isolate MR_766 was recently reported in fibroblast-derived human neural progenitor cells [39], we propose that the predicted difference in N-glycotype between African and Asian/American strains, particularly as it applies to ASN_154_, contributes to the emerging neuropathology seen in ZIKV disease.

Diversity of ZIKV across strains has major implications for molecular diagnostics. Of the nine molecular diagnostic methods evaluated *in silico*, none shared 100% identity for reagent sequences across all 33 ZIKV strains examined. The ‘Faye 2013’ method had the fewest number of escaping strains, and targeted NS5-encoding sequence employing a one-step, real-time strategy with degenerate primers [40]. Remaining strategies employed a mix of real-time and endpoint amplification strategies and utilized both degenerate and non-degenerate primers. No discernible pattern was apparent for predicting loss of reagent identity across strains based on amplification techniques. Divergence in primer or probe sequences relative to the derived consensus sequences substantially elevates the proportion of strains predicted to escape detection (*e*.*g*. ‘Tappe’ exhibits 100% reagent identity only with MR_766 and ArD158084). Given the propensity for certain ZIKV proteins such as NS2B, NS3, and NS4A to favor synonymous nucleotide changes that do not result in amino acid substitutions, diagnostic strategies focused on antigen detection interrogating these targets may provide improved detection across heterologous strains.

ZIKV phylogeny across the 33 strains explored was derived using the entire genome as opposed to a single site. ZIKV strains still resolved clearly into African and Asian clades, and the Western hemisphere isolates formed a single group most closely derived from the Asian clade. Small branches are evident from the root node of the Western Hemisphere strains, potentially marking the emergence of an American clade.

Derivation of the complete selectome, or calculation of ω value for each codon in a genome, of ZIKV added context to the observed genomic diversity. Inferring selection based on ω values has certain limitations such as discounting differences in expression levels. Like other Flaviviruses, all ZIKV genes are expressed as a single unit that are post-translationally cleaved into function proteins, thus eliminating changes in expression as a confounding factor. It is plausible that differential posttranslational modifications affect viral fitness, and are not accounted for in the selection analysis alone. To account for the potential variability between inferences of selection, we employed three different models to calculate diversifying selection (global or episodic). Despite the amount of SNP-level diversity, global evidence of diversifying selection was not observed in ZIKV. However, numerous sites appeared to be under episodic diversifying selection. This is consistent with the inclusion of many strains from a newly emerging population within the analysis. Rates of SNPs were only notably different from background for portions of genome encoding gpM and DEAD NTPase. However, the capsid, propeptide, gpM, gp1, gpC, DEAD NTPase, and helicase were all significantly different from background rates of amino acid substitutions per 100 residues. This indicates that these proteins favor SNPs encoding nonsynonymous substitutions above the rate dictated by random drift for ZIKV. Predictably, the rate of silent mutations across encoded protein sequences negatively correlates with clustering of residues under episodic diversifying selection. Overlap between proteins with significant elevations in amino acid substitutions per 100 residues and those with a significantly higher proportion of sites under episodic diversifying selection per 100 residues (relative to background) was substantial, but not absolute. Helicase and gpC showed significantly higher rates of amino acid substitution, but not rates of sites under selection. Conversely, NS1, E, NS2A, and NS5 all had significantly higher rates of sites under episodic diversifying selection, but were not elevated in their amino acid substitution rate. This indicates that helicase and gpC have a higher number of changes, but those changes are far less likely to impact protein function in a way that contributes to ZIKV fitness, while NS1, E, NS2A, and NS5 have fewer amino acid changes, but they are much more likely to contribute to ZIKV fitness. Differences in N- or O-glycotypes appear in E, propeptide, NS1, NS2A, NS3, and NS5, and may be further contributing to ZIKV fitness. Without the ability to ascribe key functional motifs to many ZIKV protein sites, evaluation of the potential role of relaxed constraint on the generation of diversity is challenging. However, sites under episodic diversifying selection were more no more likely to occur in intrinsically disordered regions, indicating that diversity is not driven exclusively by random flexibility in protein structure. The high rate of diversity in the capsid protein and the glycoproteins gpM and gp1 suggest that cell-surface interactions and/or adaptive immunity in the form of neutralizing antibodies are likely candidates for factors driving ZIKV plasticity.

Our finding of significant episodic diversifying selection [41] supports the assertion of Faye *et al*. that conditions in Southeast Asia and the Americas may be putting selective pressures not previously experienced [11], perhaps due to novel vector associations, a immunologically naïve host population, and tropism for a new host body site (*i*.*e*., neurological tissues). It would thus be predictable that similar radiating episodes may occur following ZIKV introduction to new geographic locations featuring different competent vectors [42–44]. These findings warrant major consideration during ongoing efforts toward vaccine and diagnostic test development to ensure heterologous protection and detection across strains.

## Materials and Methods

### Experimental Design

#### ZIKV Strains, Nucleotide Sequences, and Diagnostic Reagents

Nucleotide sequences encoding the complete polyprotein of ZIKV were mined from GenBank. Strain descriptions including geographic origin, year of isolation, source of isolation, and genome accession numbers are reported in Table 1[11, 13, 45–53]. Isolates represent a temporal span of 69 years; African, Asian, and emerging American clades; and sources including patient serum, fetal brain, diverse mosquito vectors, and animal reservoirs. Sequences for diagnostic reagents including PCR primers and probes were described previously [37, 50, 54–58].

#### Nucleotide and Amino Acid Sequence Analysis

Nucleotide sequences encoding the complete polyprotein from 33 ZIKV strains were aligned using Sequencher version 8.0 (GeneCodes) and a consensus sequence was generated. Nucleotide sequences were individually translated using the ExPASy Translate Tool [59]. Consensus sequence generation and mapping of amino acid substitutions among strains were mapped using ClustalΩ alignments [60]. Nucleotide and amino acid substitutions relative to each consensus sequence were tabulated across strains for: a.) each cleaved, mature protein by functional domain (if applicable) and the remaining cleavage fragments; and b.) their encoding nucleotide sequences. Results are reported as relevant functional domains where appropriate (*i*.*e*., E = gp1/c/E stem; NS3 = DEAD NTPase/helicase; NS5 = FtsJ/polymerase). Substitution totals for each feature were normalized to 100 residues to enable direct comparisons. Changes across strains relative to consensus sequences representing diagnostic primer and/or probe sequences were compiled for each individual reagent, combined for each method, and reported as a total of changes per total nucleotide. Prediction of N-, O-, and C-linked glycosylation patterns was made using GlycoEP under permissive settings (Binary profile of patterns; SVM threshold = 0.01) [61]. Prediction of intrinsically disordered regions in protein sequences were made for one strain from each clade (MR_766, FSS13025, ZikaSPH2015) was made with IUPred [62].

#### Phylogenetic Analysis

Phylogenetic relationships among the 33 examined strains were derived using the complete coding sequences. A consensus tree was generated following 100 bootstrapped replicates of a neighbor-joining tree with a Jukes-Cantor correction using MEGA 6.0 [63]. The resultant consensus tree was visualized via Phylo.io [64].

#### Evolutionary Selection Analysis

Detection of diversifying, neutral, or purifying selection acting globally across the ZIKV polyprotein was made with Bayesian models of sequence evolution using the Selecton v2.4 software suite and the HyPhy software suite [65–66]. Aligned sequences were examined for global inferences using the M8 model [67–68], and episodic diversifying selection was detected using the mixed-effect model of evolution (MEME). The M8 model prioritizes probabilities for transitions and transversions, codon bias, and among-site rate variation by differentially weighting these factors in the generation of a d_n_/d_s_ (ω) value. The MEME model differentially weighs diversity within phylogenetic branches differently than diversity among different branches, and represents a measure of radiating selection following rapid change in habitat [41].

#### Statistical Analysis

We defined background rates of nucleotide and amino acid substitution rates, and rates of sites under diversifying selection, as rates observed for sequences of or encoding nonfunctional cleavage fragments that presumably are not selected in any way. Nucleotide and amino acid substation rates and rates of sites under episodic diversifying selection for all proteins were evaluated for deviation from background by χ^2^ goodness-of-fit analysis. Indications of significance imply *P* < 0.05. All statistical analyses were performed using GraphPad Prism version 6.01.

## Supporting Information

S1 Data ZIKV Multiple Nucleotide Sequence Alignments. (PDF)

S2 Data ZIKV Multiple Amino Acid Sequence Alignments. (PDF)

S3 Data ZIKV Predicted N-Linked Glycosylation Sites. (PDF)

S4 Data ZIKV Predicted O-Linked Glycosylation Sites. (PDF)

S5 Data Selection (ω) Values calculated using the M8 Model. (PDF)

S6 Data Selection (ω) Values calculated using the MEME Model. (PDF)

S1 Fig Prediction of Intrinsically Disordered Regions in ZIKV Proteins. Disordered scores for 3 ZIKV strains are displayed for each amino acid site (X axis). The cutoff value between predicted disorder and structure is indicated at 0.4. The boundaries of mature proteins are indicated by black lines. Strains MR_766, Fss13025, and ZikaSPH2015 were utilized as representatives of the African, Asian, and American clades, respectively.
(TIF)
