# Supplementary material for: A Comprehensive Systems Biology Approach to Studying Zika Virus
Source: PLoS One. 2016 Sep 1;11(9):e0161355. doi: 10.1371/journal.pone.0161355 (PMC5008700; doi:10.1371/journal.pone.0161355)
Supplement: S1 Data — (PDF) [file pone.0161355.s001.pdf]

Predicted O-Linked Glycosylation

MR_766		IBH30656		ArD7117	
119 T	0.087597	119 T	0.196298	119 T	0.13153
126 T	0.146983	126 T	0.114449	126 T	0.146983
[redacted]					
194 T	0.183575	194 T	0.183575	194 T	0.130168
[redacted]					
330 T	0.240569	330 T	0.240569	330 T	0.240569
337 T	0.019643	337 T	0.019643	337 T	0.019643
356 S	0.047111	356 S	0.047111	356 S	0.047111
362 S	0.037459	362 S	0.037459	362 S	0.037459
410 T	0.079493	410 T	0.079493	410 T	0.079493
[redacted]					
460 T	0.106758	460 T	0.099872	460 T	0.141079
469 T	0.005823	469 T	0.005823	469 T	0.005823
[redacted]					
521 T	0.003083	521 T	0.022791	521 T	0.003083
523 T	0.01593	[redacted]		523 T	0.01593
599 T	0.069612	599 T	0.069612	[redacted]	
603 T	0.149639	603 T	0.149639	603 T	0.095881
605 T	0.35319	605 T	0.35319	[redacted]	
615 T	0.022632	[redacted]		615 T	0.017665
617 T	0.005151			617 T	0.005151
625 T	0.065249			625 T	0.120725
		641 T	0.005224	641 T	0.005224
650 T	0.177479	650 T	0.177479	650 T	0.177479
656 T	0.043733	656 T	0.043733	656 T	0.043733
[redacted]					
687 T	0.015988	[redacted]		687 T	0.015988
704 T	0.060959			704 T	0.060959
923 T	0.029399	923 T	0.029399	923 T	0.029399
980 T	0.030193	980 T	0.028349	980 T	0.028349
1087 T	0.319752	1087 T	0.319654	1087 T	0.319654
1091 S	0.077365	1091 S	0.077365	1091 S	0.077365
1095 T	0.139694	1095 T	0.139694	1095 T	0.139694
1096 T	0.104991	1096 T	0.104991	1096 T	0.104991
1145 T	0.098793	1145 T	0.098793	1145 T	0.098793
1176 T	0.065706	1176 T	0.006664	1176 T	0.065706
[redacted]					
1210 T	0.039207	1210 T	0.039207	1210 T	0.039207
[redacted]					
1305 T	0.051617	1305 T	0.010263	1305 T	0.010263
1440 T	0.066466	1440 T	0.066466	1440 T	0.066466

1529 T	0.039022	1529 T	0.039022	1529 T	0.039022
1555 T	0.107913	1555 T	0.107913	1555 T	0.107913
[redacted]					
1636 T	0.157228	1636 T	0.157228	1636 T	0.157228
1668 T	0.045227	1668 T	0.045227		
[redacted]					
1748 T	0.363795	1748 T	0.363795	1748 T	0.230319
1757 T	0.055463	1757 T	0.055463	1757 T	0.055463
[redacted]		1792 T	0.007829	[redacted]	
1818 T	0.404692	1818 T	0.404692	1818 T	0.404692
1820 T	0.334611	1820 T	0.334611	1820 T	0.334611
1824 T	0.356232	1824 T	0.356232	1824 T	0.356232
1855 T	0.165054	1855 T	0.193824	1855 T	0.193824
[redacted]		1951 T	0.067627	1879 T	0.043564
1951 T	0.067627	2057 T	0.13787	1951 T	0.067627
2057 T	0.13787	2131 T	0.023703	2057 T	0.13787
2131 T	0.027071	2173 T	0.02712	2131 T	0.023703
2173 T	0.02712	2316 T	0.162652	2173 T	0.02712
2316 T	0.162652	2317 T	0.278955	2316 T	0.162652
2317 T	0.278955	2320 T	0.208466	2317 T	0.278955
2320 T	0.208466	2328 T	0.216736	2320 T	0.208466
2328 T	0.216736	2430 T	0.002411	2328 T	0.216736
2430 T	0.002411	[redacted]		2430 T	0.002411
[redacted]		2457 T	0.02554	2469 T	0.104993
		2469 T	0.104993	2472 T	0.225078
2469 T	0.104993	2472 T	0.225078	[redacted]	
2472 T	0.225078	[redacted]			
2474 T	0.075947	2474 T	0.075947	2474 T	0.075947
2479 S	0.116964	2479 S	0.116964	2479 S	0.116964
2488 T	0.088617	2488 T	0.088617	2488 T	0.088617
2511 T	0.140249	2511 T	0.140249	2511 T	0.140249
2553 T	0.013941	2553 T	0.013941	2553 T	0.013941
2613 T	0.10376	2613 T	0.10376	2613 T	0.10376
[redacted]					
2679 T	0.084516	2679 T	0.084516	2679 T	0.084516
[redacted]					
2829 S	0.068872	2829 S	0.068872	2829 S	0.068872
2834 T	0.025182	2834 T	0.025182	2834 T	0.025182
2857 T	0.134428	2857 T	0.134428	2857 T	0.134428
2860 T	0.326454	2860 T	0.326454	2860 T	0.326454
2865 T	0.248714	2865 T	0.248714	2865 T	0.248714

3128	T	0.074139	3128	T	0.074139	3128	T	0.074139
3312	T	0.124135	3312	T	0.15859	3312	T	0.124135
3316	T	0.006967	3316	T	0.096034	3316	T	0.006967
3328	T	0.028224	[redacted]			3328	T	0.028224
[redacted]								
3356	T	0.056919	3356	T	0.0408	3356	T	0.056919
3380	T	0.048615	3380	T	0.048615	3380	T	0.048615
3419	T	0.096559	3419	T	0.096559	3419	T	0.096559

ArB7701			ArB15076			ArB13565		
119	T	0.087597	119	T	0.087597	119	T	0.087597
126	T	0.146983	126	T	0.146983	126	T	0.146983
[redacted]								
194	T	0.183575	194	T	0.183575	194	T	0.183575
[redacted]								
330	T	0.240569	330	T	0.240569	330	T	0.240569
337	T	0.019643	337	T	0.019643	337	T	0.019643
356	S	0.047111	356	S	0.047111	356	S	0.047111
362	S	0.037459	362	S	0.037459	362	S	0.037459
410	T	0.079493	410	T	0.079493	410	T	0.079493
[redacted]								
460	T	0.141079	460	T	0.099872	460	T	0.141079
469	T	0.005823	469	T	0.005823	469	T	0.005823
[redacted]								
521	T	0.003083	521	T	0.003083	521	T	0.003083
523	T	0.01593	523	T	0.01593	523	T	0.01593
599	T	0.069612	599	T	0.069612	599	T	0.069612
603	T	0.149639	603	T	0.149639	603	T	0.149639
605	T	0.35319	605	T	0.35319	605	T	0.35319
615	T	0.022632	615	T	0.022632	615	T	0.022632
617	T	0.005151	617	T	0.005151	617	T	0.005151
625	T	0.120725	625	T	0.120725	625	T	0.120725
641	T	0.005224	641	T	0.005224	641	T	0.005224
650	T	0.177479	650	T	0.177479	650	T	0.177479
656	T	0.043733	656	T	0.043733	656	T	0.043733
[redacted]								
687	T	0.015988	687	T	0.015988	687	T	0.015988
704	T	0.060959	704	T	0.060959	704	T	0.060959
923	T	0.029399				923	T	0.029399
980	T	0.028349	980	T	0.028349	980	T	0.028349
1087	T	0.319654	1087	T	0.319654	1087	T	0.319654
1091	S	0.077365	1091	S	0.077365	1091	S	0.077365
1095	T	0.139694	1095	T	0.139694	1095	T	0.139694
1096	T	0.104991	1096	T	0.104991	1096	T	0.104991
1145	T	0.098793	1145	T	0.098793	1145	T	0.098793
1176	T	0.123543	1176	T	0.065706	1176	T	0.065706
1182	T	0.014446	1182	T	0.014446	1182	T	0.014446
1210	T	0.039207	1210	T	0.039207	1210	T	0.039207
[redacted]								
1305	T	0.010263	1305	T	0.077429	1305	T	0.010263
1440	T	0.066466	1440	T	0.066466	1440	T	0.066466

1529	T	0.039022	1529	T	0.039022	1529	T	0.039022
1555	T	0.107913	1555	T	0.107913	1555	T	0.107913
[redacted]								
1636	T	0.157228	1636	T	0.157228	1636	T	0.157228
1668	T	0.045227	1668	T	0.045227	1668	T	0.045227
[redacted]								
1748	T	0.363795	1748	T	0.363795	1748	T	0.363795
1757	T	0.055463	1757	T	0.055463	1757	T	0.055463
1792	T	0.007829	1792	T	0.007829	1792	T	0.007829
1818	T	0.404692	1818	T	0.404692	1818	T	0.404692
1820	T	0.334611	1820	T	0.334611	1820	T	0.334611
1824	T	0.356232	1824	T	0.356232	1824	T	0.356232
1855	T	0.165054	1855	T	0.165054	1855	T	0.165054
[redacted]								
1951	T	0.067627	1951	T	0.067627	1951	T	0.067627
2057	T	0.13787	2057	T	0.13787	2057	T	0.13787
2131	T	0.032549	2131	T	0.027071	2131	T	0.032549
2173	T	0.02712	2173	T	0.02712	2173	T	0.02712
2316	T	0.162652	2316	T	0.162652	2316	T	0.162652
2317	T	0.278955	2317	T	0.278955	2317	T	0.278955
2320	T	0.208466	2320	T	0.208466	2320	T	0.208466
2328	T	0.216736	2328	T	0.216736	2328	T	0.216736
2430	T	0.002411	2430	T	0.002411	2430	T	0.002411
[redacted]								
2469	T	0.104993	2469	T	0.104993	2469	T	0.104993
2472	T	0.225078	2472	T	0.225078	2472	T	0.225078
2474	T	0.075947	2474	T	0.075947	2474	T	0.075947
2479	S	0.116964	2479	S	0.116964	2479	S	0.116964
2488	T	0.088617	2488	T	0.088617	2488	T	0.088617
2511	T	0.140249	2511	T	0.140249	2511	T	0.140249
2553	T	0.013941	2553	T	0.013941	2553	T	0.013941
2613	T	0.086657	2613	T	0.086657	2613	T	0.086657
[redacted]								
2679	T	0.084516	2679	T	0.084516	2679	T	0.084516
[redacted]								
2829	S	0.068872	2829	S	0.068872	2829	S	0.068872
2834	T	0.025182	2834	T	0.025182	2834	T	0.025182
2857	T	0.134428	2857	T	0.134428	2857	T	0.134428
2860	T	0.326454	2860	T	0.326454	2860	T	0.326454
2865	T	0.248714	2865	T	0.248714	2865	T	0.248714

3127	T	0.074139	3128	T	0.074139	3127	T	0.074139
3311	T	0.124135	3312	T	0.124135	3311	T	0.124135
3315	T	0.006967	3316	T	0.006967	3315	T	0.006967
3327	T	0.028224	3328	T	0.028224	3327	T	0.028224
[redacted]								
3355	T	0.056919	3356	T	0.056919	3355	T	0.056919
3379	T	0.048615	3380	T	0.048615	3379	T	0.048615
3418	T	0.096559	3419	T	0.096559	3418	T	0.096559

ArD41519			ArD128000			ArD157995		
119	T	0.13153	119	T	0.087597	119	T	0.087597
126	T	0.146983	126	T	0.146983	126	T	0.146983
[redacted]								
194	T	0.183575	194	T	0.183575	194	T	0.183575
[redacted]								
330	T	0.240569	330	T	0.240569	330	T	0.149494
337	T	0.019643	337	T	0.019643	[redacted]		
356	S	0.047111	356	S	0.047111			
362	S	0.037459	362	S	0.037459	362	S	0.144697
410	T	0.079493	410	T	0.079493	[redacted]		
460	T	0.141079	460	T	0.141079	460	T	0.141079
469	T	0.005823	469	T	0.005823	469	T	0.005823
[redacted]								
521	T	0.003083	521	T	0.003083	521	T	0.003083
523	T	0.01593	523	T	0.01593	523	T	0.01593
599	T	0.069612	599	T	0.069612	599	T	0.069612
603	T	0.149639	603	T	0.149639	603	T	0.149639
605	T	0.35319	605	T	0.35319	605	T	0.35319
615	T	0.022632	615	T	0.022632	615	T	0.097263
617	T	0.005151	617	T	0.005151	617	T	0.053284
625	T	0.120725	625	T	0.120725	625	T	0.128582
641	T	0.005224	641	T	0.005224	641	T	0.005224
650	T	0.177479	650	T	0.177479	650	T	0.177479
656	T	0.043733	656	T	0.043733	656	T	0.043733
[redacted]								
687	T	0.015988				687	T	0.015988
704	T	0.060959	704	T	0.025264	704	T	0.060959
923	T	0.029399	923	T	0.029399	923	T	0.029399
980	T	0.028349	980	T	0.028349	980	T	0.030193
1087	T	0.319654	1087	T	0.319752	1087	T	0.319654
1091	S	0.077365	1091	S	0.077365	1091	S	0.077365
1095	T	0.139694	1095	T	0.139694	1095	T	0.139694
1096	T	0.104991	1096	T	0.104991	1096	T	0.104991
1145	T	0.098793	1145	T	0.098793	1145	T	0.098793
1176	T	0.065706	1176	T	0.065706	1176	T	0.065706
[redacted]								
1210	T	0.039207	1210	T	0.039207	1210	T	0.039207
[redacted]								
			1300	T	0.183825			
1305	T	0.010263	1305	T	0.134386	1305	T	0.010263
1440	T	0.066466	1440	T	0.066466	1440	T	0.066466

1529 T	0.039022	1529 T	0.039022	1529 T	0.039022
1555 T	0.107913	1555 T	0.107913	1555 T	0.107913
[redacted]					
1636 T	0.157228	1636 T	0.157228	1636 T	0.157228
[redacted]				1668 T	0.045227
				[redacted]	
				1727 T	0.000243
				1734 S	0.005669
				1745 T	0.025873
				1747 T	0.229348
1748 T	0.363795	1748 T	0.363795	1748 T	0.338569
1757 T	0.055463	1757 T	0.055463	1757 T	0.055463
1792 T	0.007829	[redacted]			
1818 T	0.404692	1818 T	0.404692	1818 T	0.404692
1820 T	0.334611	1820 T	0.334611	1820 T	0.334611
1824 T	0.356232	1824 T	0.356232	1824 T	0.356232
1855 T	0.193824	1855 T	0.193824	1855 T	0.165054
1951 T	0.067627	[redacted]		1879 T	0.043564
2057 T	0.13787	1951 T	0.067627	1951 T	0.067627
2131 T	0.023703	2057 T	0.13787	2057 T	0.13787
2173 T	0.02712	2131 T	0.023703	2131 T	0.027071
2316 T	0.162652	2173 T	0.02712	2173 T	0.02712
2317 T	0.278955	2316 T	0.162652	2316 T	0.162652
2320 T	0.208466	2317 T	0.278955	2317 T	0.278955
2328 T	0.216736	2320 T	0.208466	2320 T	0.208466
2430 T	0.002411	2328 T	0.216736	2328 T	0.216736
[redacted]		2430 T	0.002411	2430 T	0.002411
		2469 T	0.16471	2469 T	0.104993
2469 T	0.104993	2472 T	0.225078	2472 T	0.225078
2472 T	0.225078	[redacted]			
2474 T	0.075947	2474 T	0.075947	2474 T	0.075947
2479 S	0.116964	2479 S	0.116964	2479 S	0.116964
2488 T	0.088617	2488 T	0.088617	2488 T	0.088617
2511 T	0.140249	2511 T	0.140249	2511 T	0.140249
2553 T	0.013941	2553 T	0.013941	2553 T	0.013941
2613 T	0.10376	2613 T	0.10376	2613 T	0.10376
[redacted]					
2679 T	0.084516	2679 T	0.042554	2679 T	0.084516
[redacted]					
2829 S	0.068872	2829 S	0.068872	2829 S	0.068872
2834 T	0.025182	2834 T	0.025182	2834 T	0.025182
2857 T	0.134428	2857 T	0.134428	2857 T	0.134428
2860 T	0.326454	2860 T	0.326454	2860 T	0.326454
2865 T	0.248714	2865 T	0.248714	2865 T	0.248714

3128 T	0.074139	3128 T	0.074139	3128 T	0.074139
3312 T	0.124135	3312 T	0.124135	3312 T	0.124135
3316 T	0.006967	3316 T	0.006967	3316 T	0.006967
3328 T	0.028224	3328 T	0.028224	3328 T	0.028224
[redacted]		3350 T	0.069573	[redacted]	
3356 T	0.056919	3356 T	0.239134	3356 T	0.056919
3380 T	0.048615	3380 T	0.034982	3380 T	0.048615
3419 T	0.096559	3419 T	0.096559	3419 T	0.096559

**ArB1362**			**P6-740**			**Yap2007**		
119	T	0.087597	119	T	0.146075	119	T	0.146075
126	T	0.146983	126	T	0.123209	126	T	0.123209
[redacted]			149	T	0.05768	149	T	0.038609
[redacted]								
194	T	0.183575	194	T	0.183575	194	T	0.183575
[redacted]								
330	T	0.240569	330	T	0.240569			
337	T	0.019643	337	T	0.019643			
356	S	0.047111	356	S	0.047111	356	S	0.047111
362	S	0.037459	362	S	0.037459	362	S	0.037459
410	T	0.079493	[redacted]					
[redacted]								
460	T	0.186651	460	T	0.103195	460	T	0.103195
469	T	0.005823	[redacted]					
[redacted]								
521	T	0.003083	521	T	0.003083	521	T	0.003083
523	T	0.01593	523	T	0.01593	523	T	0.01593
599	T	0.069612	599	T	0.082564	599	T	0.082564
603	T	0.149639	603	T	0.127233	603	T	0.127233
605	T	0.35319	605	T	0.338661	605	T	0.338661
615	T	0.022632	615	T	0.026037	615	T	0.026037
617	T	0.005151	[redacted]					
625	T	0.120725	625	T	0.120725	625	T	0.120725
641	T	0.005224	641	T	0.005224	641	T	0.005224
650	T	0.177479	650	T	0.177479	650	T	0.177479
656	T	0.043733	656	T	0.043733	656	T	0.043733
[redacted]								
687	T	0.015988	687	T	0.023841	687	T	0.018232
704	T	0.060959	704	T	0.060959	704	T	0.060959
923	T	0.029399	923	T	0.029399	923	T	0.029399
980	T	0.028349	980	T	0.016033	980	T	0.016033
1087	T	0.319654	1087	T	0.319654	1087	T	0.319654
1091	S	0.077365	1091	S	0.077365	1091	S	0.077365
1095	T	0.139694	1095	T	0.139694	1095	T	0.139694
1096	T	0.104991	1096	T	0.104991	1096	T	0.104991
1145	T	0.098793	1145	T	0.098793	1145	T	0.098793
1176	T	0.070649	1176	T	0.038825	1176	T	0.038825
1182	T	0.001445	1182	T	0.007262	1182	T	0.007262
1210	T	0.039207	1210	T	0.057918	1210	T	0.057918
[redacted]			1297	T	0.073487	1297	T	0.100822
[redacted]								
						1302	T	0.120207
1305	T	0.010263	1305	T	0.074391	1305	T	0.015145
1440	T	0.066466	1440	T	0.066466	1440	T	0.066466

1529	T	0.039022	1529	T	0.039022	1529	T	0.039022
1555	T	0.107913	1555	T	0.078221	1555	T	0.078221
[redacted]						1613	T	0.066101
1636	T	0.157228	1636	T	0.157228	1636	T	0.157228
1668	T	0.045227	1668	T	0.052721	1668	T	0.052721
[redacted]			1717	T	0.032852	1717	T	0.032852
[redacted]								
1748	T	0.363795	1748	T	0.363795	1748	T	0.363795
1757	T	0.055463	1757	T	0.055463	1757	T	0.055463
[redacted]			1792	T	0.007829	1792	T	0.007829
1818	T	0.404692	1818	T	0.404692	1818	T	0.404692
1820	T	0.334611	1820	T	0.334611	1820	T	0.334611
1824	T	0.356232	1824	T	0.356232	1824	T	0.356232
1855	T	0.165054	1855	T	0.165054	1855	T	0.165054
1875	T	0.009831	1951	T	0.067627	1951	T	0.067627
1951	T	0.067627	2057	T	0.13787	2057	T	0.140228
2057	T	0.13787	2131	T	0.060709	2131	T	0.060709
2131	T	0.027071	2173	T	0.02712	2173	T	0.02712
2173	T	0.07093	2316	T	0.162652	2316	T	0.194878
2316	T	0.162652	2317	T	0.278955	2317	T	0.340633
2317	T	0.278955	2320	T	0.208466	2320	T	0.184781
2320	T	0.208466	2328	T	0.216736	2328	T	0.274861
2328	T	0.216736	2430	T	0.002411	[redacted]		
2430	T	0.002411	[redacted]			2455	S	0.003898
2469	T	0.104993				2457	T	0.042948
2472	T	0.225078	2469	T	0.104993	2469	T	0.104993
[redacted]			2472	T	0.225078	2472	T	0.225078
[redacted]								
2474	T	0.075947	2474	T	0.075947	2474	T	0.075947
2479	S	0.116964	2479	S	0.116964	2479	S	0.116964
2488	T	0.088617	2488	T	0.088617	2488	T	0.088617
2511	T	0.140249	2511	T	0.140249	2511	T	0.140249
2553	T	0.013941	2553	T	0.013941	2553	T	0.013941
2613	T	0.086657	2613	T	0.086657	2613	T	0.086657
[redacted]			2634	T	0.010892	[redacted]		
2679	T	0.084516	[redacted]					
[redacted]						2747	S	0.016844
						2754	S	0.03286
						2756	T	0.054297
2829	S	0.068872	2829	S	0.068872	2829	S	0.068872
2834	T	0.025182	2834	T	0.038833	2834	T	0.038833
2857	T	0.134428	2857	T	0.134428	2857	T	0.134428
2860	T	0.326454	2860	T	0.326454	2860	T	0.326454
2865	T	0.248714	2865	T	0.248714	2865	T	0.248714

3128 T	0.074139	3128 T	0.074139	3128 T	0.074139
3312 T	0.124135	3312 T	0.12971	3312 T	0.12971
3316 T	0.006967	3316 T	0.006967	3316 T	0.006967
3328 T	0.028224	3328 T	0.004495	3328 T	0.004495
[redacted]					
3356 T	0.056919	3356 T	0.056919	3356 T	0.056919
3380 T	0.048615	3380 T	0.048615	3380 T	0.065263
3419 T	0.096559	3419 T	0.096559	3419 T	0.096559

FSS13025			CPC-0740			PLCAL_zv		
119	T	0.146075	119	T	0.146075	119	T	0.094216
126	T	0.050191	126	T	0.123209	126	T	0.194089
149	T	0.08141	149	T	0.038609	149	T	0.038609
150	T	0.037097	[redacted]					
194	T	0.183575	194	T	0.183575	194	T	0.183575
[redacted]								
330	T	0.240569	330	T	0.240569	330	T	0.240569
337	T	0.019643	337	T	0.019643	337	T	0.019643
356	S	0.047111	356	S	0.047111	356	S	0.047111
362	S	0.037459	362	S	0.037459	362	S	0.037459
[redacted]								
460	T	0.103195	460	T	0.103195	460	T	0.103195
[redacted]								
521	T	0.003083	521	T	0.003083	521	T	0.003083
523	T	0.01593	523	T	0.01593	523	T	0.01593
599	T	0.082564	599	T	0.082564	599	T	0.082564
603	T	0.127233	603	T	0.127233	603	T	0.127233
605	T	0.338661	605	T	0.338661	605	T	0.338661
615	T	0.026037	615	T	0.026037	615	T	0.026037
[redacted]								
625	T	0.120725	625	T	0.120725	625	T	0.120725
641	T	0.005224	641	T	0.005224	641	T	0.005224
650	T	0.177479	650	T	0.177479	650	T	0.177479
656	T	0.043733	656	T	0.043733	656	T	0.043733
[redacted]								
687	T	0.018232	687	T	0.018232	687	T	0.018232
704	T	0.060959	704	T	0.060959	704	T	0.060959
923	T	0.029399	923	T	0.029399	923	T	0.029399
980	T	0.016033	980	T	0.016033			
1087	T	0.319654	1087	T	0.319654	1087	T	0.319654
1091	S	0.077365	1091	S	0.077365	1091	S	0.077365
1095	T	0.139694	1095	T	0.139694	1095	T	0.139694
1096	T	0.104991	1096	T	0.104991	1096	T	0.104991
1145	T	0.098793	1145	T	0.098793	1145	T	0.098793
1176	T	0.038825	1176	T	0.038825	1176	T	0.038825
1182	T	0.007262	1182	T	0.007262	1182	T	0.007262
1210	T	0.057918	1210	T	0.057918	1210	T	0.057918
[redacted]								
1305	T	0.074391	1305	T	0.074391	1305	T	0.074391
1440	T	0.066466	1440	T	0.020976	1440	T	0.066466

1529	T	0.039022	1529	T	0.039022	1529	T	0.039022
1555	T	0.078221	1555	T	0.078221	1555	T	0.078221
[redacted]			1613	T	0.05618			
1636	T	0.157228	1636	T	0.157228	1636	T	0.157228
1668	T	0.052721	1668	T	0.052721	1668	T	0.052721
1717	T	0.032852	1717	T	0.032852	1717	T	0.032852
[redacted]								
1748	T	0.363795	1748	T	0.363795	1748	T	0.363795
1757	T	0.055463	1757	T	0.055463	1757	T	0.055463
1792	T	0.007829	1792	T	0.007829	1792	T	0.007829
1818	T	0.404692	1818	T	0.404692	1818	T	0.404692
1820	T	0.334611	1820	T	0.334611	1820	T	0.334611
1824	T	0.356232	1824	T	0.356232	1824	T	0.356232
1855	T	0.165054	1855	T	0.16374	1855	T	0.165054
1951	T	0.067627	[redacted]					
2057	T	0.13787	1951	T	0.067627	1951	T	0.067627
2131	T	0.060709	2057	T	0.13787	2057	T	0.13787
2173	T	0.02712	2131	T	0.032598	2131	T	0.060709
2316	T	0.194878	2173	T	0.02712	2173	T	0.02712
2317	T	0.340633	2316	T	0.194878	2316	T	0.194878
2320	T	0.184781	2317	T	0.340633	2317	T	0.340633
2328	T	0.274861	2320	T	0.184781	2320	T	0.184781
2430	T	0.002411	2328	T	0.274861	2328	T	0.274861
2455	S	0.003898	2430	T	0.002411	2430	T	0.002411
2457	T	0.042948	2455	S	0.003898	2455	S	0.003898
2469	T	0.104993	2457	T	0.042948	2457	T	0.042948
2472	T	0.225078	2469	T	0.104993	2469	T	0.104993
[redacted]			2472	T	0.225078	2472	T	0.225078
2474	T	0.075947	2474	T	0.075947	2474	T	0.075947
2479	S	0.116964	2479	S	0.116964	2479	S	0.116964
2488	T	0.088617	2488	T	0.088617	2488	T	0.088617
2511	T	0.140249	2511	T	0.140249	2511	T	0.140249
2553	T	0.013941	2553	T	0.013941	2553	T	0.013941
2613	T	0.086657	2613	T	0.086657	2613	T	0.086657
[redacted]								
2747	S	0.016844	2747	S	0.016844	2747	S	0.016844
2754	S	0.03286	2754	S	0.03286	2754	S	0.03286
2756	T	0.054297	2756	T	0.054297	2756	T	0.054297
2829	S	0.068872	2829	S	0.068872	2829	S	0.068872
2834	T	0.038833	2834	T	0.038833	2834	T	0.038833
2857	T	0.134428	2857	T	0.134428	2857	T	0.134428
2860	T	0.326454	2860	T	0.326454	2860	T	0.326454
2865	T	0.248714	2865	T	0.248714	2865	T	0.248714

3128	T	0.074139	3128	T	0.074139	3128	T	0.074139
3312	T	0.12971	3312	T	0.12971	3312	T	0.12971
3316	T	0.006967	3316	T	0.006967	3316	T	0.006967
3328	T	0.004495	3328	T	0.004495	3328	T	0.004495
[redacted]								
3356	T	0.056919	3356	T	0.056919	3356	T	0.056919
3380	T	0.065263	3380	T	0.065263	3380	T	0.065263
3419	T	0.096559	3419	T	0.096559	[redacted]		

H/PF/2013		SV0127		Haiti 2014	
119 T	0.094216	119 T	0.094216	119 T	0.094216
126 T	0.194089	126 T	0.194089	126 T	0.194089
[redacted]		149 T	0.038609	[redacted]	
[redacted]					
194 T	0.183575	194 T	0.183575	194 T	0.183575
[redacted]					
330 T	0.240569	330 T	0.240569	330 T	0.240569
337 T	0.019643	337 T	0.019643	337 T	0.019643
356 S	0.047111	356 S	0.047111	356 S	0.047111
362 S	0.037459	362 S	0.037459	362 S	0.037459
[redacted]					
460 T	0.103195	460 T	0.103195	460 T	0.103195
[redacted]					
[redacted]		517 T	0.103335	[redacted]	
521 T	0.003083	[redacted]		521 T	0.003083
523 T	0.01593	523 T	0.017171	523 T	0.01593
599 T	0.082564	599 T	0.082564	599 T	0.082564
603 T	0.127233	603 T	0.127233	603 T	0.127233
605 T	0.338661	605 T	0.338661	605 T	0.338661
615 T	0.026037	615 T	0.026037	615 T	0.026037
[redacted]					
625 T	0.120725	625 T	0.120725	625 T	0.120725
641 T	0.005224	641 T	0.005224	641 T	0.005224
650 T	0.177479	650 T	0.20583	650 T	0.177479
656 T	0.043733	656 T	0.008746	656 T	0.043733
[redacted]		659 T	0.005595	[redacted]	
687 T	0.018232	687 T	0.018232	687 T	0.018232
704 T	0.060959	704 T	0.060959	704 T	0.060959
923 T	0.029399	923 T	0.029399	923 T	0.032965
[redacted]					
1087 T	0.319654	1087 T	0.319654	1087 T	0.319654
1091 S	0.077365	1091 S	0.077365	1091 S	0.077365
1095 T	0.139694	1095 T	0.139694	1095 T	0.139694
1096 T	0.104991	1096 T	0.104991	1096 T	0.104991
1145 T	0.098793	1145 T	0.098793	1145 T	0.098793
1176 T	0.038825	1176 T	0.038825	1176 T	0.038825
1182 T	0.007262	1182 T	0.007262	1182 T	0.007262
1210 T	0.057918	1210 T	0.057918	1210 T	0.057918
[redacted]					
1305 T	0.074391	1305 T	0.074391	1305 T	0.074391
1440 T	0.066466	1440 T	0.066466	1440 T	0.066466

1529 T	0.039022	1529 T	0.039022	1529 T	0.039022
1555 T	0.078221	1555 T	0.078221	1555 T	0.078221
[redacted]					
1636 T	0.157228	1636 T	0.157228	1636 T	0.157228
1668 T	0.052721	1668 T	0.052721	1668 T	0.052721
1717 T	0.032852	1717 T	0.032852	1717 T	0.032852
[redacted]					
1748 T	0.363795	1748 T	0.363795	1748 T	0.363795
1757 T	0.055463	1757 T	0.055463	1757 T	0.055463
1792 T	0.007829	1792 T	0.007829	1792 T	0.007829
1818 T	0.404692	1818 T	0.404692	1818 T	0.404692
1820 T	0.334611	1820 T	0.334611	1820 T	0.334611
1824 T	0.356232	1824 T	0.356232	1824 T	0.356232
1855 T	0.165054	1855 T	0.165054	1855 T	0.130557
[redacted]		1951 T	0.067627	[redacted]	
1951 T	0.067627	2057 T	0.13787	1951 T	0.067627
2057 T	0.13787	2131 T	0.060709	2057 T	0.13787
2131 T	0.060709	2173 T	0.02712	2131 T	0.060709
2173 T	0.02712	2316 T	0.194878	2173 T	0.02712
2316 T	0.194878	2317 T	0.340633	2316 T	0.194878
2317 T	0.340633	2320 T	0.184781	2317 T	0.340633
2320 T	0.184781	2328 T	0.274861	2320 T	0.184781
2328 T	0.274861	2430 T	0.002411	2328 T	0.274861
2430 T	0.002411	2455 S	0.003898	2430 T	0.002411
2455 S	0.003898	2457 T	0.042948	[redacted]	
2457 T	0.042948	2469 T	0.104993	2457 T	0.042948
2469 T	0.104993	2472 T	0.225078	2469 T	0.104993
2472 T	0.225078	[redacted]		2472 T	0.225078
2474 T	0.075947	2474 T	0.075947	2474 T	0.075947
2479 S	0.116964	2479 S	0.116964	2479 S	0.116964
2488 T	0.088617	2488 T	0.088617	2488 T	0.088617
2511 T	0.140249	2511 T	0.140249	2511 T	0.140249
2553 T	0.013941	2553 T	0.013941	2553 T	0.013941
2613 T	0.086657	2613 T	0.086657	2613 T	0.086657
[redacted]					
2747 S	0.016844	2747 S	0.016844	2747 S	0.016844
2754 S	0.03286	2754 S	0.03286	2754 S	0.03286
2756 T	0.054297	2756 T	0.054297	2756 T	0.054297
2829 S	0.068872	2829 S	0.068872	2829 S	0.068872
2834 T	0.038833	2834 T	0.038833	2834 T	0.038833
2857 T	0.134428	2857 T	0.134428	2857 T	0.134428
2860 T	0.326454	2860 T	0.326454	2860 T	0.326454
2865 T	0.248714	2865 T	0.248714	2865 T	0.248714

3128 T	0.074139	3128 T	0.074139	3128 T	0.074139
3312 T	0.12971	3312 T	0.12971	3312 T	0.12971
3316 T	0.006967	3316 T	0.006967	3316 T	0.006967
3328 T	0.004495	3328 T	0.004495	3328 T	0.004495
[redacted]					
3356 T	0.056919	3356 T	0.056919	3356 T	0.056919
3380 T	0.065263	3380 T	0.065263	3380 T	0.065263
3419 T	0.096559	3419 T	0.096559	3419 T	0.096559

SSABr1			OPY_Martinique			103344		
119	T	0.094216	119	T	0.094216	119	T	0.094216
126	T	0.194089	126	T	0.194089	126	T	0.194089
[redacted]								
194	T	0.183575	194	T	0.183575	194	T	0.183575
[redacted]								
330	T	0.240569	330	T	0.240569	330	T	0.240569
337	T	0.019643	337	T	0.019643	337	T	0.074055
356	S	0.047111	356	S	0.047111	356	S	0.007333
362	S	0.037459	362	S	0.037459	362	S	0.037459
[redacted]								
460	T	0.103195	460	T	0.103195	460	T	0.103195
[redacted]								
521	T	0.003083	521	T	0.003083	521	T	0.003083
523	T	0.01593	523	T	0.01593	523	T	0.01593
599	T	0.082564	599	T	0.082564	599	T	0.082564
603	T	0.127233	603	T	0.127233	603	T	0.127233
605	T	0.338661	605	T	0.338661	605	T	0.338661
615	T	0.026037	615	T	0.026037	615	T	0.026037
[redacted]								
625	T	0.120725	625	T	0.120725	625	T	0.120725
641	T	0.005224	641	T	0.005224	641	T	0.005224
650	T	0.177479	650	T	0.177479	650	T	0.177479
656	T	0.043733	656	T	0.043733	656	T	0.043733
[redacted]								
687	T	0.018232	687	T	0.018232	687	T	0.018232
704	T	0.060959	704	T	0.060959	704	T	0.060959
923	T	0.029399	923	T	0.029399	923	T	0.029399
[redacted]								
1087	T	0.319654	1087	T	0.319654	1087	T	0.319654
1091	S	0.077365	1091	S	0.077365	1091	S	0.077365
1095	T	0.139694	1095	T	0.139694	1095	T	0.139694
1096	T	0.104991	1096	T	0.104991	1096	T	0.104991
1145	T	0.098793	1145	T	0.098793	1145	T	0.098793
1176	T	0.038825	1176	T	0.038825	1176	T	0.038825
1182	T	0.007262	1182	T	0.007262	1182	T	0.007262
1210	T	0.057918	1210	T	0.057918	1210	T	0.057918
[redacted]								
1305	T	0.074391	1305	T	0.074391	1305	T	0.074391
1440	T	0.066466	1440	T	0.066466	1440	T	0.066466

1529	T	0.039022	1529	T	0.039022	1529	T	0.039022
1555	T	0.078221	1555	T	0.078221	1555	T	0.078221
[redacted]								
1636	T	0.157228	1636	T	0.157228	1636	T	0.157228
1668	T	0.052721	1668	T	0.052721	1668	T	0.052721
1717	T	0.032852	1717	T	0.032852	1717	T	0.032852
[redacted]								
1748	T	0.363795	1748	T	0.363795	1748	T	0.363795
1757	T	0.055463	1757	T	0.055463	1757	T	0.055463
1792	T	0.007829	1792	T	0.007829	1792	T	0.007829
1818	T	0.404692	1818	T	0.404692	1818	T	0.404692
1820	T	0.334611	1820	T	0.334611	1820	T	0.334611
1824	T	0.356232	1824	T	0.356232	1824	T	0.356232
1855	T	0.165054	1855	T	0.165054	1855	T	0.165054
1951	T	0.067627	[redacted]					
2057	T	0.13787	1951	T	0.067627	1951	T	0.067627
2131	T	0.060709	2057	T	0.13787	2057	T	0.13787
2173	T	0.02712	2131	T	0.060709	2131	T	0.060709
2316	T	0.194878	2173	T	0.02712	2173	T	0.02712
2317	T	0.340633	2316	T	0.194878	2316	T	0.194878
2320	T	0.184781	2317	T	0.340633	2317	T	0.340633
2328	T	0.274861	2320	T	0.184781	2320	T	0.184781
2430	T	0.002411	2328	T	0.274861	2328	T	0.274861
2455	S	0.003898	2430	T	0.002411	2430	T	0.002411
2457	T	0.042948	2455	S	0.003898	2455	S	0.003898
2469	T	0.104993	2457	T	0.042948	2457	T	0.042948
2472	T	0.225078	2469	T	0.104993	2469	T	0.104993
[redacted]			2472	T	0.225078	2472	T	0.225078
2474	T	0.075947	2474	T	0.075947	2474	T	0.075947
2479	S	0.116964	2479	S	0.116964	2479	S	0.116964
2488	T	0.088617	2488	T	0.088617	2488	T	0.088617
2511	T	0.140249	2511	T	0.140249	2511	T	0.140249
2553	T	0.013941	2553	T	0.013941	2553	T	0.013941
2613	T	0.086657	2613	T	0.086657	2613	T	0.086657
[redacted]								
2747	S	0.016844	2747	S	0.016844	2747	S	0.016844
2754	S	0.03286	2754	S	0.03286	2754	S	0.03286
2756	T	0.054297	2756	T	0.054297	2756	T	0.054297
2829	S	0.068872	2829	S	0.068872	2829	S	0.068872
2834	T	0.038833	2834	T	0.038833	2834	T	0.038833
2857	T	0.134428	2857	T	0.134428	2857	T	0.134428
2860	T	0.326454	2860	T	0.326454	2860	T	0.326454
2865	T	0.248714	2865	T	0.248714	2865	T	0.248714

3128 T	0.074139	3128 T	0.074139	3128 T	0.074139
3312 T	0.12971	3312 T	0.12971	3312 T	0.12971
3316 T	0.006967	3316 T	0.006967	3316 T	0.006967
3328 T	0.004495	3328 T	0.004495	3328 T	0.004495
[redacted]					
3356 T	0.056919	3356 T	0.107531	3356 T	0.056919
3380 T	0.065263	3380 T	0.065263	3380 T	0.065263
3419 T	0.096559	3419 T	0.096559	3419 T	0.096559

8375			PRVABC59			BeH815744		
119	T	0.094216	119	T	0.094216	119	T	0.094216
126	T	0.194089	126	T	0.194089	126	T	0.194089
[redacted]								
194	T	0.183575	194	T	0.183575	194	T	0.183575
[redacted]								
330	T	0.240569	330	T	0.240569	330	T	0.240569
337	T	0.074055	337	T	0.019643	337	T	0.019643
356	S	0.007333	356	S	0.047111	356	S	0.047111
362	S	0.037459	362	S	0.037459	362	S	0.037459
[redacted]								
460	T	0.103195	460	T	0.103195	460	T	0.103195
[redacted]								
521	T	0.003083	521	T	0.003083	521	T	0.003083
523	T	0.01593	523	T	0.01593	523	T	0.01593
599	T	0.082564	599	T	0.082564	599	T	0.082564
603	T	0.127233	603	T	0.127233	603	T	0.127233
605	T	0.338661	605	T	0.338661	605	T	0.338661
615	T	0.026037	615	T	0.026037	615	T	0.026037
[redacted]								
625	T	0.120725	625	T	0.120725	625	T	0.120725
641	T	0.005224	641	T	0.005224	641	T	0.005224
650	T	0.177479	650	T	0.177479	650	T	0.177479
656	T	0.043733	656	T	0.043733	656	T	0.043733
[redacted]								
687	T	0.018232	687	T	0.018232	687	T	0.018232
704	T	0.060959	704	T	0.060959	704	T	0.060959
923	T	0.029399	923	T	0.029399	923	T	0.029399
[redacted]								
1087	T	0.319654	1087	T	0.319654	1087	T	0.319654
1091	S	0.077365	1091	S	0.077365	1091	S	0.077365
1095	T	0.139694	1095	T	0.139694	1095	T	0.139694
1096	T	0.104991	1096	T	0.104991	1096	T	0.104991
1145	T	0.098793	1145	T	0.098793	1145	T	0.098793
1176	T	0.038825	1176	T	0.038825	1176	T	0.038825
1182	T	0.007262	1182	T	0.007262	1182	T	0.007262
1210	T	0.057918	1210	T	0.057918	1210	T	0.057918
[redacted]								
1305	T	0.074391	1305	T	0.074391	1305	T	0.074391
1440	T	0.066466	1440	T	0.066466	1440	T	0.066466

1529	T	0.039022	1529	T	0.039022	1529	T	0.039022
1555	T	0.078221	1555	T	0.078221	1555	T	0.078221
[redacted]								
1636	T	0.157228	1636	T	0.157228	1636	T	0.157228
1668	T	0.052721	1668	T	0.052721	1668	T	0.052721
1717	T	0.032852	1717	T	0.032852	1717	T	0.032852
[redacted]								
1748	T	0.363795	1748	T	0.363795	1748	T	0.363795
1757	T	0.055463	1757	T	0.055463	1757	T	0.055463
1792	T	0.007829	1792	T	0.007829	1792	T	0.007829
1818	T	0.404692	1818	T	0.404692	1818	T	0.404692
1820	T	0.334611	1820	T	0.334611	1820	T	0.334611
1824	T	0.356232	1824	T	0.356232	1824	T	0.356232
1855	T	0.165054	1855	T	0.165054	1855	T	0.165054
[redacted]								
1951	T	0.067627	1951	T	0.067627	1951	T	0.067627
2057	T	0.13787	2057	T	0.13787	2057	T	0.13787
2131	T	0.060709	2131	T	0.060709	2131	T	0.060709
2173	T	0.02712	2173	T	0.02712	2173	T	0.02712
2316	T	0.194878	2316	T	0.194878	2316	T	0.194878
2317	T	0.340633	2317	T	0.340633	2317	T	0.340633
2320	T	0.184781	2320	T	0.184781	2320	T	0.184781
2328	T	0.274861	2328	T	0.274861	2328	T	0.274861
2430	T	0.002411	2430	T	0.002411	2430	T	0.002411
2455	S	0.003898	2455	S	0.003898	2455	S	0.003898
2457	T	0.042948	2457	T	0.042948	2457	T	0.042948
2469	T	0.104993	2469	T	0.104993	2469	T	0.104993
2472	T	0.225078	2472	T	0.225078	2472	T	0.225078
2474	T	0.075947	2474	T	0.075947	2474	T	0.075947
2479	S	0.116964	2479	S	0.116964	2479	S	0.116964
2488	T	0.088617	2488	T	0.088617	2488	T	0.088617
2511	T	0.140249	2511	T	0.140249	2511	T	0.140249
2553	T	0.013941	2553	T	0.013941	2553	T	0.013941
2613	T	0.086657	2613	T	0.053028	2613	T	0.086657
[redacted]								
2747	S	0.016844	2747	S	0.016844	2747	S	0.016844
2754	S	0.03286	2754	S	0.03286	2754	S	0.03286
2756	T	0.054297	2756	T	0.054297	2756	T	0.054297
2829	S	0.068872	2829	S	0.068872	2829	S	0.068872
2834	T	0.038833	2834	T	0.038833	2834	T	0.038833
2857	T	0.134428	2857	T	0.134428	2857	T	0.134428
2860	T	0.326454	2860	T	0.326454	2860	T	0.326454
2865	T	0.248714	2865	T	0.248714	2865	T	0.248714

3128 T	0.074139	3128 T	0.074139	3128 T	0.074139
3312 T	0.12971	3312 T	0.12971	3312 T	0.12971
3316 T	0.006967	3316 T	0.006967	3316 T	0.006967
3328 T	0.004495	3328 T	0.004495	3328 T	0.004495
[redacted]					
3356 T	0.056919	3356 T	0.056919	3356 T	0.056919
3380 T	0.065263	3380 T	0.065263	3380 T	0.065263
3419 T	0.096559	3419 T	0.096559	3419 T	0.096559

BeH819966			BeH819015			BEH818995		
119	T	0.094216	119	T	0.094216	119	T	0.094216
126	T	0.194089	126	T	0.194089	126	T	0.194089
[redacted]								
194	T	0.183575	194	T	0.183575	194	T	0.183575
[redacted]								
330	T	0.240569	330	T	0.240569	330	T	0.240569
337	T	0.019643	337	T	0.019643	337	T	0.019643
356	S	0.047111	356	S	0.047111	356	S	0.047111
362	S	0.037459	362	S	0.037459	362	S	0.037459
[redacted]								
460	T	0.103195	460	T	0.103195	460	T	0.103195
[redacted]								
521	T	0.003083	521	T	0.003083	521	T	0.003083
523	T	0.01593	523	T	0.01593	523	T	0.01593
599	T	0.082564	599	T	0.082564	599	T	0.082564
603	T	0.127233	603	T	0.127233	603	T	0.127233
605	T	0.338661	605	T	0.338661	605	T	0.338661
615	T	0.026037	615	T	0.026037	615	T	0.026037
[redacted]								
625	T	0.120725	625	T	0.120725	625	T	0.120725
641	T	0.005224	641	T	0.005224	641	T	0.005224
650	T	0.177479	650	T	0.177479	650	T	0.177479
656	T	0.043733	656	T	0.043733	656	T	0.043733
[redacted]								
687	T	0.018232	687	T	0.018232	687	T	0.018232
704	T	0.060959	704	T	0.060959	704	T	0.060959
923	T	0.029399	923	T	0.029399	923	T	0.029399
[redacted]								
1087	T	0.319654	1087	T	0.319654	1087	T	0.319654
1091	S	0.077365	1091	S	0.077365	1091	S	0.077365
1095	T	0.139694	1095	T	0.139694	1095	T	0.139694
1096	T	0.104991	1096	T	0.104991	1096	T	0.104991
1145	T	0.098793	1145	T	0.098793	1145	T	0.098793
1176	T	0.038825	1176	T	0.038825	1176	T	0.038825
1182	T	0.007262	1182	T	0.007262	1182	T	0.007262
1210	T	0.057918	1210	T	0.057918	1210	T	0.057918
[redacted]								
1305	T	0.074391	1305	T	0.074391	1305	T	0.074391
1440	T	0.066466	1440	T	0.066466	1440	T	0.066466

1529	T	0.039022	1529	T	0.039022	1529	T	0.039022
1555	T	0.078221	1555	T	0.078221	1555	T	0.078221
[redacted]								
1636	T	0.157228	1636	T	0.157228	1636	T	0.157228
1668	T	0.052721	1668	T	0.052721	1668	T	0.052721
1717	T	0.032852	1717	T	0.032852	1717	T	0.032852
[redacted]								
1748	T	0.363795	1748	T	0.363795	1748	T	0.363795
1757	T	0.055463	1757	T	0.055463	1757	T	0.055463
1792	T	0.007829	1792	T	0.007829	1792	T	0.007829
1818	T	0.404692	1818	T	0.404692	1818	T	0.404692
1820	T	0.334611	1820	T	0.334611	1820	T	0.334611
1824	T	0.356232	1824	T	0.356232	1824	T	0.356232
1855	T	0.165054	1855	T	0.165054	1855	T	0.165054
[redacted]								
1951	T	0.067627	1951	T	0.067627	1951	T	0.067627
2057	T	0.13787	2057	T	0.13787	2057	T	0.13787
2131	T	0.060709	2131	T	0.060709	2131	T	0.060709
2173	T	0.02712	2173	T	0.02712	2173	T	0.02712
2316	T	0.194878	2316	T	0.194878	2316	T	0.194878
2317	T	0.340633	2317	T	0.340633	2317	T	0.340633
2320	T	0.184781	2320	T	0.184781	2320	T	0.184781
2328	T	0.274861	2328	T	0.274861	2328	T	0.274861
2430	T	0.002411	2430	T	0.002411	2430	T	0.002411
2455	S	0.003898	2455	S	0.003898	2455	S	0.003898
2457	T	0.042948	2457	T	0.042948	2457	T	0.042948
2469	T	0.104993	2469	T	0.104993	2469	T	0.104993
2472	T	0.225078	2472	T	0.225078	2472	T	0.225078
2474	T	0.075947	2474	T	0.075947	2474	T	0.075947
2479	S	0.116964	2479	S	0.116964	2479	S	0.116964
2488	T	0.088617	2488	T	0.088617	2488	T	0.088617
2511	T	0.140249	2511	T	0.140249	2511	T	0.140249
2553	T	0.013941	2553	T	0.013941	2553	T	0.013941
2613	T	0.086657	2613	T	0.086657	2613	T	0.086657
[redacted]								
2747	S	0.016844	2747	S	0.016844	2747	S	0.016844
2754	S	0.03286	2754	S	0.03286	2754	S	0.03286
2756	T	0.054297	2756	T	0.054297	2756	T	0.054297
2829	S	0.068872	2829	S	0.068872	2829	S	0.068872
2834	T	0.038833	2834	T	0.038833	2834	T	0.038833
2857	T	0.134428	2857	T	0.134428	2857	T	0.134428
2860	T	0.326454	2860	T	0.326454	2860	T	0.326454
2865	T	0.248714	2865	T	0.248714	2865	T	0.248714

3128 T	0.074139	3128 T	0.074139	3128 T	0.074139
3312 T	0.12971	3312 T	0.12971	3312 T	0.12971
3316 T	0.006967	3316 T	0.006967	3316 T	0.006967
3328 T	0.004495	3328 T	0.004495	3328 T	0.004495
[redacted]					
3356 T	0.056919	3356 T	0.056919	3356 T	0.056919
3380 T	0.065263	3380 T	0.065263	3380 T	0.065263
3419 T	0.096559	3419 T	0.096559	3419 T	0.096559

Z1106033			ZikaSPH2015			Brasil_Zikv2015		
119	T	0.094216	119	T	0.094216	119	T	0.094216
126	T	0.194089	126	T	0.194089	126	T	0.194089
[redacted]								
194	T	0.183575	194	T	0.183575	194	T	0.183575
[redacted]								
[redacted]			322	T	0.005585	[redacted]		
330	T	0.240569	330	T	0.240569	330	T	0.240569
337	T	0.019643	337	T	0.019643	337	T	0.019643
356	S	0.047111	356	S	0.047111	356	S	0.047111
362	S	0.037459	362	S	0.037459	362	S	0.037459
[redacted]								
460	T	0.103195	460	T	0.103195	460	T	0.103195
[redacted]								
521	T	0.003083	521	T	0.003083	521	T	0.003083
523	T	0.01593	523	T	0.01593	523	T	0.01593
599	T	0.082564	599	T	0.082564	599	T	0.082564
603	T	0.127233	603	T	0.127233	603	T	0.127233
605	T	0.338661	605	T	0.338661	605	T	0.338661
615	T	0.026037	615	T	0.026037	615	T	0.026037
[redacted]								
625	T	0.120725	625	T	0.120725	625	T	0.120725
641	T	0.005224	641	T	0.005224	641	T	0.005224
650	T	0.177479	650	T	0.177479	650	T	0.177479
656	T	0.043733	656	T	0.043733	656	T	0.043733
[redacted]								
687	T	0.018232	687	T	0.018232	687	T	0.018232
704	T	0.060959	704	T	0.060959	704	T	0.060959
923	T	0.029399	923	T	0.032965	923	T	0.029399
[redacted]								
1087	T	0.319654	1087	T	0.319654	1087	T	0.319654
1091	S	0.077365	1091	S	0.077365	1091	S	0.077365
1095	T	0.139694	1095	T	0.139694	1095	T	0.139694
1096	T	0.104991	1096	T	0.104991	1096	T	0.104991
1145	T	0.098793	1145	T	0.098793	1145	T	0.098793
1176	T	0.038825	1176	T	0.038825	1176	T	0.038825
1182	T	0.007262	1182	T	0.007262	1182	T	0.007262
1210	T	0.057918	1210	T	0.057918	1210	T	0.057918
[redacted]								
1305	T	0.074391	1305	T	0.074391	1305	T	0.074391
1440	T	0.066466	1440	T	0.066466	1440	T	0.066466

1529 T	0.039022	1529 T	0.039022	1529 T	0.039022
1555 T	0.078221	1555 T	0.078221	1555 T	0.078221
[redacted]					
1636 T	0.157228	1636 T	0.157228	1636 T	0.157228
1668 T	0.052721	1668 T	0.052721	1668 T	0.052721
1717 T	0.032852	1717 T	0.032852	1717 T	0.032852
[redacted]					
1748 T	0.363795	1748 T	0.363795	1748 T	0.363795
1757 T	0.055463	1757 T	0.055463	1757 T	0.055463
1792 T	0.007829	1792 T	0.007829	1792 T	0.007829
1818 T	0.404692	1818 T	0.404692	1818 T	0.404692
1820 T	0.334611	1820 T	0.334611	1820 T	0.334611
1824 T	0.356232	1824 T	0.356232	1824 T	0.356232
1855 T	0.165054	1855 T	0.130557	1855 T	0.165054
[redacted]					
1951 T	0.067627	1951 T	0.067627	1951 T	0.067627
2057 T	0.13787	2057 T	0.13787	2057 T	0.13787
2131 T	0.060709	2131 T	0.060709	2131 T	0.060709
2173 T	0.02712	2173 T	0.02712	2173 T	0.02712
2316 T	0.194878	2316 T	0.194878	2316 T	0.194878
2317 T	0.340633	2317 T	0.340633	2317 T	0.340633
2320 T	0.184781	2320 T	0.184781	2320 T	0.184781
2328 T	0.274861	2328 T	0.274861	2328 T	0.274861
2430 T	0.002411	2430 T	0.002411	2430 T	0.002411
2455 S	0.003898	[redacted]		2455 S	0.003898
2457 T	0.042948	2457 T	0.042948	2457 T	0.042948
2469 T	0.104993	2469 T	0.104993	2469 T	0.104993
2472 T	0.225078	2472 T	0.225078	2472 T	0.225078
2474 T	0.075947	2474 T	0.075947	2474 T	0.075947
2479 S	0.116964	2479 S	0.116964	2479 S	0.116964
2488 T	0.088617	2488 T	0.088617	2488 T	0.088617
2511 T	0.140249	2511 T	0.140249	2511 T	0.140249
2553 T	0.013941	2553 T	0.013941	2553 T	0.013941
2613 T	0.086657	2613 T	0.086657	2613 T	0.086657
[redacted]					
2747 S	0.016844	2747 S	0.016844	2747 S	0.016844
2754 S	0.03286	2754 S	0.03286	2754 S	0.03286
2756 T	0.054297	2756 T	0.054297	2756 T	0.054297
2829 S	0.068872	2829 S	0.068872	2829 S	0.119285
2834 T	0.038833	2834 T	0.038833	2834 T	0.030935
2857 T	0.134428	2857 T	0.134428	2857 T	0.134428
2860 T	0.326454	2860 T	0.326454	2860 T	0.326454
2865 T	0.248714	2865 T	0.248714	2865 T	0.248714

3128 T	0.074139	3128 T	0.074139	3128 T	0.074139
3312 T	0.12971	3312 T	0.12971	3312 T	0.12971
3316 T	0.006967	3316 T	0.006967	3316 T	0.006967
3328 T	0.004495	3328 T	0.004495	3328 T	0.004495
[redacted]					
3356 T	0.056919	3356 T	0.056919	3356 T	0.056919
3380 T	0.065263	3380 T	0.065263	3380 T	0.065263
3419 T	0.096559	3419 T	0.096559	3419 T	0.096559

GD01

119	T	0.094216
126	T	0.194089
[redacted]		
194	T	0.183575
[redacted]		
330	T	0.240569
337	T	0.019643
356	S	0.047111
362	S	0.037459
[redacted]		
446	T	0.048597
460	T	0.103195
[redacted]		
521	T	0.003083
523	T	0.01593
599	T	0.082564
603	T	0.127233
605	T	0.338661
615	T	0.026037
[redacted]		
625	T	0.120725
641	T	0.005224
650	T	0.177479
656	T	0.043733
[redacted]		
687	T	0.018232
704	T	0.060959
923	T	0.029399
[redacted]		
1087	T	0.319654
1091	S	0.077365
1095	T	0.139694
1096	T	0.104991
1145	T	0.098793
1176	T	0.038825
1182	T	0.007262
1210	T	0.057918
[redacted]		
1305	T	0.074391
1440	T	0.066466

1529	T	0.039022
1555	T	0.078221
[redacted]		
1636	T	0.157228
1668	T	0.052721
1717	T	0.032852
[redacted]		
1748	T	0.363795
1757	T	0.055463
1792	T	0.007829
1818	T	0.404692
1820	T	0.334611
1824	T	0.356232
1855	T	0.165054
[redacted]		
1951	T	0.067627
2057	T	0.13787
2131	T	0.060709
2173	T	0.02712
2316	T	0.194878
2317	T	0.340633
2320	T	0.184781
2328	T	0.274861
2430	T	0.002411
2455	S	0.003898
2457	T	0.042948
2469	T	0.104993
2472	T	0.225078
2474	T	0.075947
2479	S	0.116964
2488	T	0.088617
2511	T	0.140249
2553	T	0.013941
2613	T	0.086657
[redacted]		
2829	S	0.068872
2834	T	0.038833
2857	T	0.134428
2860	T	0.326454
2865	T	0.248714

3128	T	0.074139
3312	T	0.12971
3316	T	0.006967
3328	T	0.004495
[redacted]		
3356	T	0.056919
3380	T	0.065263
3419	T	0.096559
