# Supplementary material for: A Comprehensive Systems Biology Approach to Studying Zika Virus
Source: PLoS One. 2016 Sep 1;11(9):e0161355. doi: 10.1371/journal.pone.0161355 (PMC5008700; doi:10.1371/journal.pone.0161355)
Supplement: S2 Data — (PDF) [file pone.0161355.s002.pdf]

Predicted N-linked Glycosylation

MR_766			IBH30656			ArD7117		
192	NTT	0.986334	192	NTT	0.986334	192	NTT	0.986334
[redacted]								
767	NGS	1.075813	765	NGS	1.075813	771	NGS	1.075813
920	NNS	1.376734	918	NNS	1.376734	924	NNS	1.376734
997	NDT	1.021465	995	NDT	1.021465	1001	NDT	1.092811
1240	NWT	0.563446	1238	NWT	0.563446	1244	NWT	0.563446
[redacted]								
1656	NGS	1.027645	1654	NGS	1.027645	1660	NGS	1.027645
1747	NVT	1.082205	1745	NVT	1.082205	1751	NVT	1.143663
2066	NNT	1.069336	2064	NNT	1.069336	2070	NNT	1.069336
2329	NYS	0.981235	2327	NYS	0.981235	2333	NYS	0.981235
2481	NSS	1.269934	2479	NSS	1.269934	2485	NSS	1.269934
2730	NST	0.688944	2728	NST	0.688944	2734	NST	0.688944

ArB7701			ArB15076			ArB13565		
192	NTT	0.986334	192	NTT	0.986334	192	NTT	0.986334
[redacted]								
771	NGS	1.075813	765	NGS	1.075813	771	NGS	1.075813
924	NNS	1.376734	[redacted]			924	NNS	1.376734
1001	NDT	1.021465	995	NDT	1.021465	1001	NDT	1.021465
1244	NWT	0.563446	1238	NWT	0.563446	1244	NWT	0.563446
[redacted]								
1660	NGS	1.027645	1654	NGS	1.027645	1660	NGS	1.027645
1751	NVT	1.082205	1745	NVT	1.082205	1751	NVT	1.082205
2070	NNT	1.069336	2064	NNT	1.069336	2070	NNT	1.069336
2333	NYS	0.981235	2327	NYS	0.981235	2333	NYS	0.981235
2485	NSS	1.269934	2479	NSS	1.269934	2485	NSS	1.269934
2734	NST	0.688944	2728	NST	0.688944	2734	NST	0.688944

ArD41519			ArD128000			ArD157995		
192	NTT	0.986334	192	NTT	0.986334	192	NTT	0.986334
444	NDT	0.654125	[redacted]					
771	NGS	1.075813	771	NGS	1.075813	771	NGS	1.075813
924	NNS	1.376734	924	NNS	1.376734	924	NNS	1.376734
1001	NDT	1.092811	1001	NDT	1.090305	1001	NDT	1.021465
1244	NWT	0.563446	1244	NWT	0.563446	1244	NWT	0.535177
[redacted]								
1660	NGS	1.027645	1660	NGS	1.027645	1660	NGS	1.027645
1751	NVT	1.082205	1751	NVT	1.082205	1751	NVT	1.000052
2070	NNT	1.069336	2070	NNT	1.069336	2070	NNT	1.069336
2333	NYS	0.981235	2333	NYS	0.981235	2333	NYS	0.981235
2485	NSS	1.269934	2485	NSS	1.269934	2485	NSS	1.269934
2734	NST	0.688944	2734	NST	0.688944	2734	NST	0.688944

ArB1362			P6-740			Yap2007		
192	NTT	0.986334	192	NTT	0.986334	192	NTT	0.986334
[redacted]			444	NDT	0.654125	444	NDT	0.654125
771	NGS	1.075813	771	NGS	1.075813	771	NGS	1.075813
924	NNS	1.376734	924	NNS	1.376734	924	NNS	1.376734
1001	NDT	1.021465	1001	NDT	1.021465	1001	NDT	1.021465
1244	NWT	0.563446	1244	NWT	0.563446	1244	NWT	0.563446
[redacted]			1295	NIT	0.963005	1295	NIT	1.110413
1660	NGS	1.027645	1660	NGS	1.027645	1660	NGS	1.027645
1751	NVT	1.082205	1751	NVT	1.082205	1751	NVT	1.082205
2070	NNT	1.069336	2070	NNT	1.069336	2070	NNT	1.125815
2333	NYS	0.981235	2333	NYS	0.981235	2333	NYS	0.981235
2485	NSS	1.269934	2485	NSS	1.269934	2485	NSS	1.269934
2734	NST	0.688944	2734	NST	0.688944	2734	NST	0.688944

FSS13025			CPC-0740			PLCAL_zv		
192	NTT	0.986334	192	NTT	0.986334	155	NTT	0.986334
444	NDT	0.654125	444	NDT	0.654125	407	NDT	0.654125
771	NGS	0.91811	771	NGS	1.075813	734	NGS	0.878675
924	NNS	1.376734	924	NNS	1.376734	887	NNS	1.376734
1001	NDT	1.021465	1001	NDT	1.021465	964	NDT	1.021465
1244	NWT	0.563446	1244	NWT	0.563446	1207	NWT	0.563446
1295	NIT	1.02583	1295	NIT	1.02583	1258	NIT	1.02583
1660	NGS	1.027645	1660	NGS	1.027645	1623	NGS	1.027645
1751	NVT	1.082205	1751	NVT	1.082205	1714	NVT	1.082205
2070	NNT	1.069336	2070	NNT	1.021644	2033	NNT	1.069336
2333	NYS	0.981235	2333	NYS	0.981235	2296	NYS	0.981235
2485	NSS	1.269934	2485	NSS	1.269934	2448	NSS	1.269934
2734	NST	0.688944	2734	NST	0.688944	2697	NST	0.688944

H/PF/2013			SV0127			Haiti 2014		
192	NTT	0.986334	192	NTT	0.986334	192	NTT	0.986334
444	NDT	0.654125	444	NDT	0.654125	444	NDT	0.654125
771	NGS	0.878675	771	NGS	0.878675	771	NGS	0.878675
924	NNS	1.376734	924	NNS	1.376734	924	NNS	1.30725
1001	NDT	1.021465	1001	NDT	1.092811	1001	NDT	1.021465
1244	NWT	0.563446	1244	NWT	0.563446	1244	NWT	0.563446
1295	NIT	1.02583	1295	NIT	1.02583	1295	NIT	1.02583
1660	NGS	1.027645	1660	NGS	1.027645	1660	NGS	1.027645
1751	NVT	1.082205	1751	NVT	1.082205	1751	NVT	1.082205
2070	NNT	1.069336	2070	NNT	1.021644	2070	NNT	1.069336
2333	NYS	0.981235	2333	NYS	0.981235	2333	NYS	0.981235
2485	NSS	1.269934	2485	NSS	1.269934	2485	NSS	1.269934
2734	NST	0.688944	2734	NST	0.688944	2734	NST	0.688944

SSABr1			OPY_Martinique			103344		
192	NTT	0.986334	192	NTT	0.986334	192	NTT	0.986334
444	NDT	0.654125	444	NDT	0.654125	444	NDT	0.654125
771	NGS	0.878675	771	NGS	0.878675	771	NGS	0.878675
924	NNS	1.376734	924	NNS	1.376734	924	NNS	1.376734
1001	NDT	1.021465	1001	NDT	1.021465	1001	NDT	1.021465
1244	NWT	0.563446	1244	NWT	0.563446	1244	NWT	0.563446
1295	NIT	1.02583	1295	NIT	1.02583	1295	NIT	1.02583
1660	NGS	1.027645	1660	NGS	1.027645	1660	NGS	1.027645
1751	NVT	1.082205	1751	NVT	1.082205	1751	NVT	1.082205
2070	NNT	1.069336	2070	NNT	1.069336	2070	NNT	1.112465
2333	NYS	0.981235	2333	NYS	0.981235	2333	NYS	0.981235
2485	NSS	1.269934	2485	NSS	1.269934	2485	NSS	1.269934
2734	NST	0.688944	2734	NST	0.688944	2734	NST	0.688944

8375			PRVABC59			BeH815744		
192	NTT	0.986334	192	NTT	0.986334	192	NTT	0.986334
444	NDT	0.654125	444	NDT	0.654125	444	NDT	0.654125
771	NGS	0.878675	771	NGS	0.878675	771	NGS	0.878675
924	NNS	1.376734	924	NNS	1.376734	924	NNS	1.376734
1001	NDT	1.021465	1001	NDT	1.021465	1001	NDT	1.021465
1244	NWT	0.563446	1244	NWT	0.563446	1244	NWT	0.563446
1295	NIT	1.02583	1295	NIT	1.02583	1295	NIT	1.02583
1660	NGS	1.027645	1660	NGS	1.027645	1660	NGS	1.027645
1751	NVT	1.082205	1751	NVT	1.082205	1751	NVT	1.082205
2070	NNT	1.112465	2070	NNT	1.069336	2070	NNT	1.069336
2333	NYS	0.981235	2333	NYS	0.981235	2333	NYS	0.981235
2485	NSS	1.269934	2485	NSS	1.269934	2485	NSS	1.269934
2734	NST	0.688944	2734	NST	0.688944	2734	NST	0.688944

BeH819966			BeH819015			BEH818995		
192	NTT	0.986334	192	NTT	0.986334	192	NTT	0.986334
444	NDT	0.654125	444	NDT	0.654125	444	NDT	0.654125
771	NGS	0.878675	771	NGS	0.878675	771	NGS	0.878675
924	NNS	1.376734	924	NNS	1.376734	924	NNS	1.376734
1001	NDT	1.021465	1001	NDT	1.021465	1001	NDT	1.021465
1244	NWT	0.563446	1244	NWT	0.563446	1244	NWT	0.563446
1295	NIT	1.02583	1295	NIT	1.02583	1295	NIT	1.02583
1660	NGS	1.027645	1660	NGS	1.027645	1660	NGS	1.027645
1751	NVT	1.082205	1751	NVT	1.082205	1751	NVT	1.082205
2070	NNT	1.069336	2070	NNT	1.069336	2070	NNT	1.069336
2333	NYS	0.981235	2333	NYS	0.981235	2333	NYS	0.981235
2485	NSS	1.269934	2485	NSS	1.269934	2485	NSS	1.269934
2734	NST	0.688944	2734	NST	0.688944	2734	NST	0.688944

Z1106033			ZikaSPH2015			Brasil_Zikv2015		
			Position	Residue	Score			
192	NTT	0.986334	192	NTT	0.986334	192	NTT	0.986334
444	NDT	0.654125	444	NDT	0.654125	444	NDT	0.654125
771	NGS	0.979703	771	NGS	0.878675	771	NGS	0.878675
924	NNS	1.376734	924	NNS	1.30725	924	NNS	1.376734
1001	NDT	1.021465	1001	NDT	1.021465	1001	NDT	1.021465
1244	NWT	0.563446	1244	NWT	0.563446	1244	NWT	0.563446
1295	NIT	1.02583	1295	NIT	1.02583	1295	NIT	1.02583
1660	NGS	1.027645	1660	NGS	1.027645	1660	NGS	1.027645
1751	NVT	1.082205	1751	NVT	1.082205	1751	NVT	1.082205
2070	NNT	1.069336	2070	NNT	1.069336	2070	NNT	1.069336
2333	NYS	0.981235	2333	NYS	0.981235	2333	NYS	0.981235
2485	NSS	1.269934	2485	NSS	1.269934	2485	NSS	1.269934
2734	NST	0.688944	2734	NST	0.688944	2734	NST	0.688944

GD01

192	NTT	0.986334
444	NGT	0.648744
771	NGS	0.878675
924	NNS	1.376734
1001	NDT	1.021465
1244	NWT	0.563446
1295	NIT	1.040512
1660	NGS	1.027645
1751	NVT	1.082205
2070	NNT	1.069336
2333	NYS	0.981235
2485	NSS	1.269934
2734	NST	0.688944
