# Supplementary material for: A Comprehensive Systems Biology Approach to Studying Zika Virus
Source: PLoS One. 2016 Sep 1;11(9):e0161355. doi: 10.1371/journal.pone.0161355 (PMC5008700; doi:10.1371/journal.pone.0161355)
Supplement: S3 Data — (PDF) [file pone.0161355.s003.pdf]

CLUSTAL O(1.2.1) multiple sequence alignment

```
P6-740_1            MKNPKKKSGGFRIVNMLKRGVARVSPFGGLKRLPAGLLLGHGPIRMVLAILAFLRFTAIK      60
P6740               MKNPKKKSGGFRIVNMLKRGVARVSPFGGLKRLPAGLLLGHGPIRMVLAILAFLRFTAIK      60
Yap2007             MKNPKEEIRRIRIVNMLKRGVARVSPFGGLKRLPAGLLLGHGPIRMVLAILAFLRFTAIK      60
CPC0740_1           MKNPKKKSGGFRIVNMLKRGVARVSPFGGLKRLPAGLLLGHGPIRMVLAILAFLRFTAIK      60
SV0127/14_1         MKNPKKKSGGFRIVNMLKRGVARVSPFGGLKRLPAGLLLGHGPIRMVLAILAFLRFTAIK      60
FSS13025_1          MKNPKKKSGGFRIVNMLKRGVARVSPFGGLKRLPAGLLLGHGPIRMVLAILAFLRFTAIK      60
8375_1              MKNPKKKSGGFRIVNMLKRGVARVSPFGGLKRLPAGLLLGHGPIRMVLAILAFLRFTAIK      60
103344_1            MKNPKKKSGGFRIVNMLKRGVARVSPFGGLKRLPAGLLLGHGPIRMVLAILAFLRFTAIK      60
GD01_1              MKNPKKKSGGFRIVNMLKRGVARVSPFGGLKRLPAGLLLGHGPIRMVLAILAFLRFTAIK      60
SPH2015_1           MKNPKKKSGGFRIVNMLKRGVARVSPFGGLKRLPAGLLLGHGPIRMVLAILAFLRFTAIK      60
Haiti2014_1         MKNPKKKSGGFRIVNMLKRGVARVSPFGGLKRLPAGLLLGHGPIRMVLAILAFLRFTAIK      60
PLCal_ZV_1          ------------------------------------LLGHGPIRMVLAILAFLRFTAIK      23
Martinique          MKNPKKKSGGFRIVNMLKRGVARVSPFGGLKRLPAGLLLGHGPIRMVLAILAFLRFTAIK      60
NatalRGN            MKNPKKKSGGFRIVNMLKRGVARVSPFGGLKRLPAGLLLGHGPIRMVLAILAFLRFTAIK      60
BrasilZKV2015_1     MKNPKKKSGGFRIVNMLKRGVARVSPFGGLKRLPAGLLLGHGPIRMVLAILAFLRFTAIK      60
Z1106033_1          MKNPKKKSGGFRIVNMLKRGVARVSPFGGLKRLPAGLLLGHGPIRMVLAILAFLRFTAIK      60
PRVABC59_1          MKNPKKKSGGFRIVNMLKRGVARVSPFGGLKRLPAGLLLGHGPIRMVLAILAFLRFTAIK      60
HPF2013_1           MKNPKKKSGGFRIVNMLKRGVARVSPFGGLKRLPAGLLLGHGPIRMVLAILAFLRFTAIK      60
Beh815744_1         MKNPKKKSGGFRIVNMLKRGVARVSPFGGLKRLPAGLLLGHGPIRMVLAILAFLRFTAIK      60
BEH818995_1         MKNPKKKSGGFRIVNMLKRGVARVSPFGGLKRLPAGLLLGHGPIRMVLAILAFLRFTAIK      60
BEH819966_1         MKNPKKKSGGFRIVNMLKRGVARVSPFGGLKRLPAGLLLGHGPIRMVLAILAFLRFTAIK      60
BeH819015_1         MKNPKKKSGGFRIVNMLKRGVARVSPFGGLKRLPAGLLLGHGPIRMVLAILAFLRFTAIK      60
SSABr_1             MKNPKKKSGGFRIVNMLKRGVARVSPFGGLKRLPAGLLLGHGPIRMVLAILAFLRFTAIK      60
ARD157995_1         MKNPKKKSGRFRIVNMLKRGVARVNPLGGLKRLPAGLLLGHGPIRMVLAILAFLRFTAIK      60
MR_766_1            MKNPKEEIRRIRIVNMLKRGVARVNPLGGLKRLPAGLLLGHGPIRMVLAILAFLRFTAIK      60
ARD158084_1         MKNPKKKSGGFRIVNMLKRGVARVNPLGGLKRLPAGLLLGHGPIRMVLAILAFLRFTAIK      60
ArB1362_1           MKNPKKKSGGFRIVNMLKRGVARVNPLGGLKRLPAGLLLGHGPIRMVLAILAFLRFTAIK      60
ARB15076_1          MKNPKKKSGGFRIVNMLKRGVARVNPLGGLKRLPAGLLLGHGPIRMVLAILAFLRFTAIK      60
ARB13565_1          MKNPKKKSGGFRIVNMLKRGVARVNPLGGLKRLPAGLLLGHGPIRMVLAILAFLRFTAIK      60
ARB7701_1           MKNPKKKSGGFRIVNMLKRGVARVNPLGGLKRLPAGLLLGHGPIRMVLAILAFLRFTAIK      60
IbH30656_1          MKNPKKKSGGFRIVNMLKRGVARVNPLGGLKRLPAGLLLGHGPIRMVLAILAFLRFTAIK      60
ArD128000_1         MKNPKRKSGGFRIVNMLKRGVARVNPLGGLKRLPAGLLLGHGPIRMVLAILAFLRFTAIK      60
ArD7117_1           MKNPKKRSGGFRIVNMLKRGVARVNPLGGLKRLPAGLLLGHGPIRMVLAILAFLRFTAIK      60
ARD_41519_1         MKNPKRKSGGFRIVNMLKRGVARVNPLGGLKRLPAGLLLGHGPIRMVLAILAFLRFTAIK      60
                                                        ************************

P6-740_1            PSLGLINRWGSVGKKEAMEIIKKFKKDLAAMLRIINARKEKKRRGADTSVGIVGLLLTTA      120
P6740               PSLGLINRWGSVGKKEAMEIIKKFKKDLAAMLRIINARKEKKRRGADTSVGIVGLLLTTA      120
Yap2007             PSLGLINRWGSVGKKEAMEIIKKFKKDLAAMLRIINARKEKKRRGTDTSVGIVGLLLTTA      120
CPC0740_1           PSLGLINRWGSVGKKEAMEIIKKFKKDLAAMLRIINARKEKKRRGADTSVGIVGLLLTTA      120
SV0127/14_1         PSLGLINRWGSVGKKEAMEIIKKFKKDLAAMLRIINARKEKKRRGTDTSVGIVGLLLTTA      120
FSS13025_1          PSLGLINRWGSVGKKEAMEIIKKFKKDLAAMLRIINARKEKKRRGTDTSVGIVGLLLTTA      120
8375_1              PSLGLINRWGSVGKKEAMEIIKKFKKDLAAMLRIINARKEKKRRGADTSVGIVGLLLTTA      120
103344_1            PSLGLINRWGSVGKKEAMEIIKKFKKDLAAMLRIINARKEKKRRGADTSVGIVGLLLTTA      120
GD01_1              PSLGLINRWGSVGKKEAMEIIKKFKKDLAAMLRIINARKEKKRRGAETSVGIVGLLLTTA      120
SPH2015_1           PSLGLINRWGSVGKKEAMEIIKKFKKDLAAMLRIINARKEKKRRGADTSVGIVGLLLTTA      120
Haiti2014_1         PSLGLINRWGSVGKKEAMEIIKKFKKDLAAMLRIINARKEKKRRGADTSVGIVGLLLTTA      120
PLCal_ZV_1          PSLGLINRWGSVGKKEAMEIIKKFKKDLAAMLRIINARKEKKRRGTDTSVGIVGLLLTTA      83
Martinique          PSLGLINRWGSVGKKEAMEIIKKFKKDLAAMLRIINARKEKKRRGAETSVGIVGLLLTTA      120
NatalRGN            PSLGLINRWGSVGKKEAMEIIKKFKKDLAAMLRIINARKEKKRRGADTSVGIVGLLLTTA      120
BrasilZKV2015_1     PSLGLINRWGSVGKKEAMEIIKKFKKDLAAMLRIINARKEKKRRGADTSVGIVGLLLTTA      120
Z1106033_1          PSLGLINRWGSVGKKEAMEIIKKFKKDLAAMLRIINARKEKKRRGADTSVGIVGLLLTTA      120
PRVABC59_1          PSLGLINRWGSVGKKEAMETIKKFKKDLAAMLRIINARKEKKRRGADTSVGIVGLLLTTA      120
HPF2013_1           PSLGLINRWGSVGKKEAMEIIKKFKKDLAAMLRIINARKEKKRRGADTSVGIVGLLLTTA      120
Beh815744_1         PSLGLINRWGSVGKKEAMEIIKKFKKDLAAMLRIINARKEKKRRGADTSVGIVGLLLTTA      120
BEH818995_1         PSLGLINRWGSVGKKEAMEIIKKFKKDLAAMLRIINARKEKKRRGADTSVGIVGLLLTTA      120
BEH819966_1         PSLGLINRWGSVGKKEAMEIIKKFKKDLAAMLRIINARKEKKRRGADTSVGIVGLLLTTA      120
BeH819015_1         PSLGLINRWGSVGKKEAMEIIKKFKKDLAAMLRIINARKEKKRRGADTSVGIVGLLLTTA      120
SSABr_1             PSLGLINRWGSVGKKEAMEIIKKFKKDLAAMLRIINARKEKKRRGADTSVGIVGLLLTTA      120
ARD157995_1         PSLGLINRWGSVGKKEAMEIIKKFKKDLAAMLRIINARKERKRRGADTSIGIIGLLLTTA      120
MR_766_1            PSLGLINRWGSVGKKEAMEIIKKFKKDLAAMLRIINARKERKRRGADTSIGIIGLLLTTA      120
ARD158084_1         PSLGLINRWGSVGKKEAMEIIKKFKKDLAAMLRIINARKERKRRGADTSIGIIGLLLTTA      120
ArB1362_1           PSLGLINRWGSVGKKEAMEIIKKFKKDLAAMLRIINARKERKRRGADTSIGIIGLLLTTA      120
```

```
ARB15076_1          PSLGLINRWGSVGKKEAMEIIKKFKKDLAAMLRIINARKERKRRGADTSIGIIGLLLTTA    120
ARB13565_1          PSLGLINRWGSVGKKEAMEIIKKFKKDLAAMLRIINARKERKRRGADTSIGIIGLLLTTA    120
ARB7701_1           PSLGLINRWGSVGKKEAMEIIKKFKKDLAAMLRIINARKERKRRGADTSIGIIGLLLTTA    120
IbH30656_1          PSLGLINRWGSVGKKEAMEIIKKFKKDLAAMLRIINARKERKRRGADASIGIVSLLLTTV    120
ArD128000_1         PSLGLINRWGSVGKKEAMEIIKKFKKDLAAMLRIINARKERKRRGADTSIGIIGLLLTTA    120
ArD7117_1           PSLGLINRWGSVGKKEAMEIIKKFKKDLAAMLRIINARKERKRRGADTSIGIVGLLLTTA    120
ARD_41519_1         PSLGLINRWGSVGKKEAMEIIKKFKKDLAAMLRIINARKERKRRGADTSIGIVGLLLTTA    120
                    ******************* ********************:****:::*:**:.*****.

P6-740_1            MAVEVTRRGSAYYMYLDRSDAGEAISFPTTLGVNKCYIQIMDLGHMCDATMSYECPMLDE    180
P6740               MAVEVTRRGSAYYMYLDRSDAGEAISFPTTLGVNKCYIQIMDLGHMCDATMSYECPMLDE    180
Yap2007             MAVEVTRRGSAYYMYLDRSDAGEAISFPTTLGMNKCYIQIMDLGHMCDATMSYECPMLDE    180
CPC0740_1           MAVEVTRRGSAYYMYLDRSDAGEAISFPTTLGMNKCYIQIMDLGHMCDATMSYECPMLDE    180
SV0127/14_1         MAAEVTRRGSAYYMYLDRSDAGEAISFPTTLGMNKCYIQIMDLGHMCDATMSYECPMLDE    180
FSS13025_1          MAVEVTRRGNAYYMYLDRSDAGEAISFPTTMGMNKCYIQIMDLGHMCDATMSYECPMLDE    180
8375_1              MAAEVTRRGSAYYMYLDRNDAGEAISFPTTLGMNKCYIQIMDLGHMCDATMSYECPMLDE    180
103344_1            MAAEVTRRGSAYYMYLDRNDAGEAISFPTTLGMNKCYIQIMDLGHMCDATMSYECPMLDE    180
GD01_1              MAAEVTRRGSAYYMYLDRNDAGEAISFPTTLGMNKCYIQIMDLGHMCDATMSYECPMLDE    180
SPH2015_1           MAAEVTRRGSAYYMYLDRNDAGEAISFPTTLGMNKCYIQIMDLGHMCDATMSYECPMLDE    180
Haiti2014_1         MAAEVTRRGSAYYMYLDRNDAGEAISFPTTLGMNKCYIQIMDLGHMCDATMSYECPMLDE    180
PLCal_ZV_1          MAAEVTRRGSAYYMYLDRSDAGEAISFPTTLGMNKCYIQIMDLGHMCDATMSYECPMLDE    143
Martinique          MAAEVTRRGSAYYMYLDRNDAGEAISFPTTLGMNKCYIQIMDLGHMCDATMSYECPMLDE    180
NatalRGN            MAAEVTRRGSAYYMYLDRNDAGEAISFPTTLGMNKCYIQIMDLGHMCDATMSYECPMLDE    180
BrasilZKV2015_1     MAAEVTRRGSAYYMYLDRNDAGEAISFPTTLGMNKCYIQIMDLGHMCDATMSYECPMLDE    180
Z1106033_1          MAAEVTRRGSAYYMYLDRNDAGEAISFPTTLGMNKCYIQIMDLGHTCDATMSYECPMLDE    180
PRVABC59_1          MAAEVTRRGSAYYMYLDRNDAGEAISFPTTLGMNKCYIQIMDLGHMCDATMSYECPMLDE    180
HPF2013_1           MAAEVTRRGSAYYMYLDRNDAGEAISFPTTLGMNKCYIQIMDLGHMCDATMSYECPMLDE    180
Beh815744_1         MAAEVTRRGSAYYMYLDRNDAGEAISFPTTLGMNKCYIQIMDLGHMCDATMSYECPMLDE    180
BEH818995_1         MAAEVTRRGSAYYMYLDRNDAGEAISFPTTLGMNKCYIQIMDLGHMCDATMSYECPMLDE    180
BEH819966_1         MAAEVTRRGSAYYMYLDRNDAGEAISFPTTLGMNKCYIQIMDLGHMCDATMSYECPMLDE    180
BeH819015_1         MAAEVTRRGSAYYMYLDRNDAGEAISFPTTLGMNKCYIQIMDLGHMCDATMSYECPMLDE    180
SSABr_1             MAAEVTRRGSAYYMYLDRNDAGEAISFPTTLGMNKCYIQIMDLGHMCDATMSYECPMLDE    180
ARD157995_1         MAAEITRRGSAYYMYLDRSDAGKAISFATTLGVNKCHVQIMDLGHMCDATMSYECPMLDE    180
MR_766_1            MAAEITRRGSAYYMYLDRSDAGKAISFATTLGVNKCHVQIMDLGHMCDATMSYECPMLDE    180
ARD158084_1         MAAEITRRGSAYYMYLDRSDAGKAISFATTLGVNKCHVQIMDLGHMCDATMSYECPMLDE    180
ArB1362_1           MAAEITRRGSAYYMYLDRSDAGKAISFATTLGVNKCHVQIMDLGHMCDATMSYECPMLDE    180
ARB15076_1          MAAEITRRGSAYYMYLDRSDAGKAISFATNLGVNKCHVQIMDLGHMCDATMSYECPMLDE    180
ARB13565_1          MAAEITRRGSAYYMYLDRSDAGKAISFATNLGVNKCHVQIMDLGHMCDATMSYECPMLDE    180
ARB7701_1           MAAEITRRGSAYYMYLDRSDAGKAISFATNLGVNKCHVQIMDLGHMCDATMSYECPMLDE    180
IbH30656_1          MAAEITRRGSAYYMYLDRSDAGKAISFVTTLGVNKCHVQIMDLGHMCDATMSYECPMLDE    180
ArD128000_1         MAAEITRRGSAYYMYLDRSDAGKAISFATTLGVNKCHVQIMDLGHMCDATMSYECPMLDE    180
ArD7117_1           MAAEITRRGSAYYMYLDRSDAGKAISFATTLGVNKCHVQIMDLGHMCDATMSYECPMLDE    180
ARD_41519_1         MAAEITRRGSAYYMYLDRSDAGKAISFATTLGVNKCHVQIMDLGHMCDATMSYECPMLDE    180
                    **.*:****.********.***:**** *.:*:***::******* **************

P6-740_1            GVEPDDVDCWCNTTSTWVVYGTCHHKKGEARRSRRAVTLPSHSTRKLQTRSQTWLESREY    240
P6740               GVEPDDVDCWCNTTSTWVVYGTCHHKKGEARRSRRAVTLPSHSTRKLQTRSQTWLESREY    240
Yap2007             GVEPDDVDCWCNTTSTWVVYGTCHHKKGEARRSRRAVTLPSHSTRKLQTRSQTWLESREY    240
CPC0740_1           GVEPDDVDCWCNTTSTWVVYGTCHHKKGEARRSRRAVTLPSHSTRKLQTRSQTWLESREY    240
SV0127/14_1         GVEPDDVDCWCNTTSTWVVYGTCHHKKGEARRSRRAVTLPSHSTRKLQTRSQTWLESREY    240
FSS13025_1          GVEPDDVDCWCNTTSTWVVYGTCHHKKGEARRSRRAVTLPSHSTRKLQTRSQTWLESREY    240
8375_1              GVEPDDVDCWCNTTSTWVVYGTCHHKKGEARRSRRAVTLPSHSTRKLQTRSQTWLESREY    240
103344_1            GVEPDDVDCWCNTTSTWVVYGTCHHKKGEARRSRRAVTLPSHSTRKLQTRSQTWLESREY    240
GD01_1              GVEPDDVDCWCNTTSTWVVYGTCHHKKGEARRSRRAVTLPSHSTRKLQTRSQTWLESREY    240
SPH2015_1           GVEPDDVDCWCNTTSTWVVYGTCHHKKGEARRSRRAVTLPSHSTRKLQTRSQTWLESREY    240
Haiti2014_1         GVEPDDVDCWCNTTSTWVVYGTCHHKKGEARRSRRAVTLPSHSTRKLQTRSQTWLESREY    240
PLCal_ZV_1          GVEPDDVDCWCNTTSTWVVYGTCHHKKGEARRSRRAVTLPSHSTRKLQTRSQTWLESREY    203
Martinique          GVEPDDVDCWCNTTSTWVVYGTCHHKKGEARRSRRAVTLPSHSTRKLQTRSQTWLESREY    240
NatalRGN            GVEPDDVDCWCNTTSTWVVYGTCHHKKGEARRSRRAVTLPSHSTRKLQTRSQTWLESREY    240
BrasilZKV2015_1     GVEPDDVDCWCNTTSTWVVYGTCHHKKGEARRSRRAVTLPSHSTRKLQTRSQTWLESREY    240
Z1106033_1          GVEPDDVDCWCNTTSTWVVYGTCHHKKGEARRSRRAVTLPSHSTRKLQTRSQTWLESREY    240
PRVABC59_1          GVEPDDVDCWCNTTSTWVVYGTCHHKKGEARRSRRAVTLPSHSTRKLQTRSQTWLESREY    240
HPF2013_1           GVEPDDVDCWCNTTSTWVVYGTCHHKKGEARRSRRAVTLPSHSTRKLQTRSQTWLESREY    240
Beh815744_1         GVEPDDVDCWCNTTSTWVVYGTCHHKKGEARRSRRAVTLPSHSTRKLQTRSQTWLESREY    240
BEH818995_1         GVEPDDVDCWCNTTSTWVVYGTCHHKKGEARRSRRAVTLPSHSTRKLQTRSQTWLESREY    240
BEH819966_1         GVEPDDVDCWCNTTSTWVVYGTCHHKKGEARRSRRAVTLPSHSTRKLQTRSQTWLESREY    240
BeH819015_1         GVEPDDVDCWCNTTSTWVVYGTCHHKKGEARRSRRAVTLPSHSTRKLQTRSQTWLESREY    240
SSABr_1             GVEPDDVDCWCNTTSTWVVYGTCHHKKGEARRSRRAVTLPSHSTRKLQTRSQTWLESREY    240
```

```
ARD157995_1         GVEPDDVDCWCNTTSTWVVYGTCHHKKGETRRSRRSVSLRYHYTRKLQTRSQTWLESREY      240
MR_766_1            GVEPDDVDCWCNTTSTWVVYGTCHHKKGEARRSRRAVTLPSHSTRKLQTRSQTWLESREY      240
ARD158084_1         GVEPDDVDCWCNTTSTWVVYGTCHHKKGEARRSRRAVTLPSHSTRKLQTRSQTWLESREY      240
ArB1362_1           GVEPDDVDCWCNTTSTWVVYGTCHHKKGEARRSRRAVTLPSHSTRKLQTRSQTWLESREY      240
ARB15076_1          GVEPDDVDCWCNTTSTWVVYGTCHHKKGEARRSRRAVTLPSHSTRKLQTRSQTWLESREY      240
ARB13565_1          GVEPDDVDCWCNTTSTWVVYGTCHHKKGEARRSRRAVTLPSHSTRKLQTRSQTWLESREY      240
ARB7701_1           GVEPDDVDCWCNTTSTWVVYGTCHHKKGEARRSRRAVTLPSHSTRKLQTRSQTWLESREY      240
IbH30656_1          GVEPDDVDCWCNTTSTWVVYGTCHHKKGEARRSRRAVTLPSHSTRKLQTRSQTWLESREY      240
ArD128000_1         GVEPDDVDCWCNTTSTWVVYGTCHHKKGEARRSRRAVTLPSHSTRKLQTRSQTWLESREY      240
ArD7117_1           GVEPDDVDCWCNTTSTWVVYGTCQHKKGEARRSRRAVTLPSHSTRKLQTRSQTWLESREY      240
ARD_41519_1         GVEPDDVDCWCNTTSTWVVYGTCHHKKGEARRSRRAVTLPSHSTRKLQTRSQTWLESREY      240
                    ***********************:*****:*****:*:*  * *****************

P6-740_1            TKHLIRVENWIFRNPGFALAAAAIAWLLGSSTSQKVIYLVMILLIAPAYSIRCIGVSNRD      300
P6740               TKHLIRVENWIFRNPGFALAAAAIAWLLGSSTSQKVIYLVMILLIAPAYSIRCIGVSNRD      300
Yap2007             TKHLIRVENWIFRNPGFALAAAAIAWLLGSSTSQKVIYLVMILLIAPAYSIRCIGVSNRD      300
CPC0740_1           TKHLIRVENWIFRNPGFALAAAVIAWLLGSSTSQKVIYLVMILLIAPAYSIRCIGVSNRD      300
SV0127/14_1         TKHLIRVENWIFRNPGFALAAAAIAWLLGSSTSQKVIYLVMILLIAPAYSIRCIGVSNRD      300
FSS13025_1          TKHLIRVENWIFRNPGFALAAAAIAWLLGSSTSQKVIYLVMILLIAPAYSIRCIGVSNRD      300
8375_1              TKHLIRVENWIFRNPGFALAAAAIAWLLGSSTSQKVIYLVMILLIAPAYSIRCIGVSNRD      300
103344_1            TKHLIRVENWIFRNPGFALAAAAIAWLLGSSTSQKVIYLVMILLIAPAYSIRCIGVSNRD      300
GD01_1              TKHLIRVENWIFRNPGFALAAAAIAWLLGSSTSQKVIYLVMILLIAPAYSIRCIGVSNRD      300
SPH2015_1           TKHLIRVENWIFRNPGFALAAAAIAWLLGSSTSQKVIYLVMILLIAPAYSIRCIGVSNRD      300
Haiti2014_1         TKHLIRVENWIFRNPGFALAAAAIAWLLGSSTSQKVIYLVMILLIAPAYSIRCIGVSNRD      300
PLCal_ZV_1          TKHLIRVENWIFRNPGFALAAAAIAWLLGSSTSQKVIYLVMILLIAPAYSIRCIGVSNRD      263
Martinique          TKHLIRVENWIFRNPGFALAAAAIAWLLGSSTSQKVIYLVMILLIAPAYSIRCIGVSNRD      300
NatalRGN            TKHLIRVENWIFRNPGFALAAAAIAWLLGSSTSQKVIYLVMILLIAPAYSIRCIGVSNRD      300
BrasilZKV2015_1     TKHLIRVENWIFRNPGFALAAAAIAWLLGSSTSQKVIYLVMILLIAPAYSIRCIGVSNRD      300
Z1106033_1          TKHLIRVENWIFRNPGFALAAAAIAWLLGSSTSQKVIYLVMILLIAPAYSIRCIGVSNRD      300
PRVABC59_1          TKHLIRVENWIFRNPGFALAAAAIAWLLGSSTSQKVIYLVMILLIAPAYSIRCIGVSNRD      300
HPF2013_1           TKHLIRVENWIFRNPGFALAAAAIAWLLGSSTSQKVIYLVMILLIAPAYSIRCIGVSNRD      300
Beh815744_1         TKHLIRVENWIFRNPGFALAAAAIAWLLGSSTSQKVIYLVMILLIAPAYSIRCIGVSNRD      300
BEH818995_1         TKHLIRVENWIFRNPGFALAAAAIAWLLGSSTSQKVIYLVMILLIAPAYSIRCIGVSNRD      300
BEH819966_1         TKHLIRVENWIFRNPGFALAAAAIAWLLGSSTSQKVIYLVMILLIAPAYSIRCIGVSNRD      300
BeH819015_1         TKHLIRVENWIFRNPGFALAAAAIAWLLGSSTSQKVIYLVMILLIAPAYSIRCIGVSNRD      300
SSABr_1             TKHLIRVENWIFRNPGFALAAAAIAWLLGSSTSQKVIYLVMILLIAPAYSIRCIGVSNRD      300
ARD157995_1         KKHLIMVENWIFRNPGFAIVSVAITWLMGSLTSQKVIYLVMIVLIVPAYSISCIGVSNRD      300
MR_766_1            TKHLIKVENWIFRNPGFALVAVAIAWLLGSSTSQKVIYLVMILLIAPAYSIRCIGVSNRD      300
ARD158084_1         TKHLIKVENWIFRNPGFALVAVAIAWLLGSSTSQKVIYLVMILLIAPAYSIRCIGVSNRD      300
ArB1362_1           TKHLIKVENWIFRNPGFALAAVAIAWLLGSSTSQKVIYLIMILLIAPAYSIRCIGVSNRD      300
ARB15076_1          TKHLIKVENWIFRNPGFALAAVAIAWLLGSSTSQKVIYLVMILLIAPAYSIRCIGVSNRD      300
ARB13565_1          TKHLIKVENWIFRNPGFALAAVAIAWLLGSSTSQKVIYLVMILLIAPAYSIRCIGVSNRD      300
ARB7701_1           TKHLIKVENWIFRNPGFALAAVAIAWLLGSSTSQKVIYLVMILLIAPAYSIRCIGVSNRD      300
IbH30656_1          TKHLIKVENWIFRNPGFALVAVAIAWLLGSSTSQKVIYLVMILLIAPAYSIRCIGVSNRD      300
ArD128000_1         TKHLIKVENWIFRNPGFALAAVAIAWLLGSSTSQKVIYLVMILLIAPAYSIRCIGVSNRD      300
ArD7117_1           TKHLIKVENWIFRNPGFALVAVAIAWLLGSSTSQKVIYLVMILLIAPAYSIRCIGVSNRD      300
ARD_41519_1         TKHLIKVENWIFRNPGFALVAVAIAWLLGSSTSQKVIYLVMILLIAPAYSIRCIGVSNRD      300
                    .**** ************:.:..*:**:** ********:**:**.***** ********

P6-740_1            FVEGMSGGTWVDVVLEHGGCVTVMAQDKPTVDIELVTTTVSNMAEVRSYCYEASISDMAS      360
P6740               FVEGMSGGTWVDVVLEHGGCVTVMAQDKPTVDIELVTTTVSNMAEVRSYCYEASISDMAS      360
Yap2007             FVEGMSGGTWVDVVLEHGGCVTVMAQDKPAVDIELVTTTVSNMAEVRSYCYEASISDMAS      360
CPC0740_1           FVEGMSGGTWVDVVLEHGGCVTVMAQDKPTVDIELVTTTVSNMAEVRSYCYEASISDMAS      360
SV0127/14_1         FVEGMSGGTWVDVVLEHGGCVTVMAQDKPTVDIELVTTTVSNMAEVRSYCYEASISDMAS      360
FSS13025_1          FVEGMSGGTWVDVVLEHGGCVTVMAQDKPTVDIELVTTTVSNMAEVRSYCYEASISDMAS      360
8375_1              FVEGMSGGTWVDVVLEHGGCVTVMAQDKPTVDIELVTTTVSNMAEIRSYCYEASISDMAS      360
103344_1            FVEGMSGGTWVDVVLEHGGCVTVMAQDKPTVDIELVTTTVSNMAEIRSYCYEASISDMAS      360
GD01_1              FVEGMSGGTWVDVVLEHGGCVTVMAQDKPTVDIELVTTTVSNMAEVRSYCYEASISDMAS      360
SPH2015_1           FVEGMSGGTWVDIVLEHGGCVTVMAQDKPTVDIELVTTTVSNMAEVRSYCYEASISDMAS      360
Haiti2014_1         FVEGMSGGTWVDVVLEHGGCVTVMAQDKPTVDIELVTTTVSNMAEVRSYCYEASISDMAS      360
PLCal_ZV_1          FVEGMSGGTWVDVVLEHGGCVTVMAQDKPTVDIELVTTTVSNMAEVRSYCYEASISDMAS      323
Martinique          FVEGMSGGTWVDVVLEHGGCVTVMAQDKPTVDIELVTTTVSNMAEVRSYCYEASISDMAS      360
NatalRGN            FVEGMSGGTWVDVVLEHGGCVTVMAQDKPTVDIELVTTTVSNMAEVRSYCYEASISDMAS      360
BrasilZKV2015_1     FVEGMSGGTWVDVVLEHGGCVTVMAQDKPTVDIELVTTTVSNMAEVRSYCYEASISDMAS      360
Z1106033_1          FVEGMSGGTWVDVVLEHGGCVTVMAQDKPTVDIELVTTTVSNMAEVRSYCYEASISDMAS      360
PRVABC59_1          FVEGMSGGTWVDVVLEHGGCVTVMAQDKPTVDIELVTTTVSNMAEVRSYCYEASISDMAS      360
HPF2013_1           FVEGMSGGTWVDVVLEHGGCVTVMAQDKPTVDIELVTTTVSNMAEVRSYCYEASISDMAS      360
Beh815744_1         FVEGMSGGTWVDVVLEHGGCVTVMAQDKPTVDIELVTTTVSNMAEVRSYCYEASISDMAS      360
```

```
BEH818995_1          FVEGMSGGTWVDVVLEHGGCVTVMAQDKPTVDIELVTTTVSNMAEVRSYCYEASISDMAS       360
BEH819966_1          FVEGMSGGTWVDVVLEHGGCVTVMAQDKPTVDIELVTTTVSNMAEVRSYCYEASISDMAS       360
BeH819015_1          FVEGMSGGTWVDVVLEHGGCVTVMAQDKPTVDIELVTTTVSNMAEVRSYCYEASISDMAS       360
SSABr_1              FVEGMSGGTWVDVVLEHGGCVTVMAQDKPTVDIELVTTTVSNMAEVRSYCYEASISDMAS       360
ARD157995_1          LVEGMSGGTWVDVVLEHGGCVTEMAQDKPTVDIELVTMTVSNMAEVRSYCYEASLSDMAS       360
MR_766_1             FVEGMSGGTWVDVVLEHGGCVTVMAQDKPTVDIELVTTTVSNMAEVRSYCYEASISDMAS       360
ARD158084_1          FVEGMSGGTWVDVVLEHGGCVTVMAQDKPTVDIELVTTTVSNMAEVRSYCYEASISDMAS       360
ArB1362_1            FVEGMSGGTWVDVVLEHGGCVTVMAQDKPTVDIELVTTTVSNMAEVRSYCYEASISDMAS       360
ARB15076_1           FVEGMSGGTWVDVVLEHGGCVTVMAQDKPTVDIELVTTTVSNMAEVRSYCYEASISDMAS       360
ARB13565_1           FVEGMSGGTWVDVVLEHGGCVTVMAQDKPTVDIELVTTTVSNMAEVRSYCYEASISDMAS       360
ARB7701_1            FVEGMSGGTWVDVVLEHGGCVTVMAQDKPTVDIELVTTTVSNMAEVRSYCYEASISDMAS       360
IbH30656_1           FVEGMSGGTWVDVVLEHGGCVTVMAQDKPTVDIELVTTTVSNMAEVRSYCYEASISDMAS       360
ArD128000_1          FVEGMSGGTWVDVVLEHGGCVTVMAQDKPTVDIELVTTTVSNMAEVRSYCYEASISDMAS       360
ArD7117_1            FVEGMSGGTWVDVVLEHGGCVTVMAQDKPTVDIELVTTTVSNMAEVRSYCYEASISDMAS       360
ARD_41519_1          FVEGMSGGTWVDVVLEHGGCVTVMAQDKPTVDIELVTTTVSNMAEVRSYCYEASISDMAS       360
                     :***********:********* ******:******* *******:********:*****

P6-740_1             DSRCPTQGEAYLDKQSDTQYVCKRTLVDRGWGNGCGLFGKGSLVTCAKFACSKKMTGKSI       420
P6740                DSRCPTQGEAYLDKQSDTQYVCKRTLVDRGWGNGCGLFGKGSLVTCAKFACSKKMTGKSI       420
Yap2007              DSRCPTQGEAYLDKQSDTQYVCKRTLVDRGWGNGCGLFGKGSLVTCAKFACSKKMTGKSI       420
CPC0740_1            DSRCPTQGEAYLDKQSDTQYVCKRTLVDRGWGNGCGLFGKGSLVTCAKFACSKKMTGKSI       420
SV0127/14_1          DSRCPTQGEAYLDKQSDTQYVCKRTLVDRGWGNGCGLFGKGSLVTCAKFACSKKMTGKSI       420
FSS13025_1           DSRCPTQGEAYLDKQSDTQYVCKRTLVDRGWGNGCGLFGKGSLVTCAKFACSKKMTGKSI       420
8375_1               DSRCPTQGEAYLDKQSDTQYVCKRTLVDRGWGNGCGLFGKGSLVTCAKFACSKKMTGKSI       420
103344_1             DSRCPTQGEAYLDKQSDTQYVCKRTLVDRGWGNGCGLFGKGSLVTCAKFACSKKMTGKSI       420
GD01_1               DSRCPTQGEAYLDKQSDTQYVCKRTLVDRGWGNGCGLFGKGSLVTCAKFACSKKMTGKSI       420
SPH2015_1            DSRCPTQGEAYLDKQSDTQYVCKRTLVDRGWGNGCGLFGKGSLVTCAKFACSKKMTGKSI       420
Haiti2014_1          DSRCPTQGEAYLDKQSDTQYVCKRTLVDRGWGNGCGLFGKGSLVTCAKFACSKKMTGKSI       420
PLCal_ZV_1           DSRCPTQGEAYLDKQSDTQYVCKRTLVDRGWGNGCGLFGKGSLVTCAKFACSKKMTGKSI       383
Martinique           DSRCPTQGEAYLDKQSDTQYVCKRTLVDRGWGNGCGLFGKGSLVTCAKFACSKKMTGKSI       420
NatalRGN             DSRCPTQGEAYLDKQSDTQYVCKRTLVDRGWGNGCGLFGKGSLVTCAKFACSKKMTGKSI       420
BrasilZKV2015_1      DSRCPTQGEAYLDKQSDTQYVCKRTLVDRGWGNGCGLFGKGSLVTCAKFACSKKMTGKSI       420
Z1106033_1           DSRCPTQGEAYLDKQSDTQYVCKRTLVDRGWGNGCGLFGKGSLVTCAKFACSKKMTGKSI       420
PRVABC59_1           DSRCPTQGEAYLDKQSDTQYVCKRTLVDRGWGNGCGLFGKGSLVTCAKFACSKKMTGKSI       420
HPF2013_1            DSRCPTQGEAYLDKQSDTQYVCKRTLVDRGWGNGCGLFGKGSLVTCAKFACSKKMTGKSI       420
Beh815744_1          DSRCPTQGEAYLDKQSDTQYVCKRTLVDRGWGNGCGLFGKGSLVTCAKFACSKKMTGKSI       420
BEH818995_1          DSRCPTQGEAYLDKQSDTQYVCKRTLVDRGWGNGCGLFGKGSLVTCAKFACSKKMTGKSI       420
BEH819966_1          DSRCPTQGEAYLDKQSDTQYVCKRTLVDRGWGNGCGLFGKGSLVTCAKFACSKKMTGKSI       420
BeH819015_1          DSRCPTQGEAYLDKQSDTQYVCKRTLVDRGWGNGCGLFGKGSLVTCAKFACSKKMTGKSI       420
SSABr_1              DSRCPTQGEAYLDKQSDTQYVCKRTLVDRGWGNGCGLFGKGSLVTCAKFACSKKMTGKSI       420
ARD157995_1          ASRCPTQGEPSLDKQSDTQSVCKRTLGDRGWGNGCGIFGKGSLVTCSKFTCCKKMPGKSI       420
MR_766_1             DSRCPTQGEAYLDKQSDTQYVCKRTLVDRGWGNGCGLFGKGSLVTCAKFTCSKKMTGKSI       420
ARD158084_1          DSRCPTQGEAYLDKQSDTQYVCKRTLVDRGWGNGCGLFGKGSLVTCAKFTCSKKMTGKSI       420
ArB1362_1            DSRCPTQGEAYLDKQSDTQYVCKRTLVDRGWGNGCGLFGKGSLVTCAKFTCSKKMTGKSI       420
ARB15076_1           DSRCPTQGEAYLDKQSDTQYVCKRTLVDRGWGNGCGLFGKGSLVTCAKFTCSKKMTGKSI       420
ARB13565_1           DSRCPTQGEAYLDKQSDTQYVCKRTLVDRGWGNGCGLFGKGSLVTCAKFTCSKKMTGKSI       420
ARB7701_1            DSRCPTQGEAYLDKQSDTQYVCKRTLVDRGWGNGCGLFGKGSLVTCAKFTCSKKMTGKSI       420
IbH30656_1           DSRCPTQGEAYLDKQSDTQYVCKRTLVDRGWGNGCGLFGKGSLVTCAKFTCSKKMTGKSI       420
ArD128000_1          DSRCPTQGEAYLDKQSDTQYVCKRTLVDRGWGNGCGLFGKGSLVTCAKFTCSKKMTGKSI       420
ArD7117_1            DSRCPTQGEAYLDKQSDTQYVCKRTLVDRGWGNGCGLFGKGSLVTCAKFTCSKKMTGKSI       420
ARD_41519_1          DSRCPTQGEAYLDKQSDTQYVCKRTLVDRGWGNGCGLFGKGSLVTCAKFTCSKKMTGKSI       420
                      ********  ******** ****** *********:*********:**:*.*** ****

P6-740_1             QPENLEYRIMLSVHGSQHSGMIVNDTGHETDENRAKVEITPNSPRAEATLGGFGSLGLDC       480
P6740                QPENLEYRIMLSVHGSQHSGMIVNDXGHETDENRAKVEITPNSPRAEATLGGFGSLGLDC       480
Yap2007              QPENLEYRIMLSVHGSQHSGMIVNDTGHETDENRAKVEITPNSPRAEATLGGFGSLGLDC       480
CPC0740_1            QPENLEYRIMLSVHGSQHSGMIVNDTGHETDENRAKVEITPNSPRAEATLGGFGSLGLDC       480
SV0127/14_1          QPENLEYRIMLSVHGSQHSGMIVNDTGHETDENRAKVEITPNSPRAEATLGGFGSLGLDC       480
FSS13025_1           QPENLEYRIMLSVHGSQHSGMIVNDTGHETDENRAKVEITPNSPRAEATLGGFGSLGLDC       480
8375_1               QPENLEYRIMLSVHGSQHSGMIVNDTGHETDENRAKVEITPNSPRAEATLGGFGSLGLDC       480
103344_1             QPENLEYRIMLSVHGSQHSGMIVNDTGHETDENRAKVEITPNSPRAEATLGGFGSLGLDC       480
GD01_1               QPENLEYRIMLSVHGSQHSGMIVNGTGHETDENRAKVEITPNSPRAEATLGGFGSLGLDC       480
SPH2015_1            QPENLEYRIMLSVHGSQHSGMIVNDTGHETDENRAKVEITPNSPRAEATLGGFGSLGLDC       480
Haiti2014_1          QPENLEYRIMLSVHGSQHSGMIVNDTGHETDENRAKVEITPNSPRAEATLGGFGSLGLDC       480
PLCal_ZV_1           QPENLEYRIMLSVHGSQHSGMIVNDTGHETDENRAKVEITPNSPRAEATLGGFGSLGLDC       443
Martinique           QPENLEYRIMLSVHGSQHSGMIVNDTGHETDENRAKVEITPNSPRAEATLGGFGSLGLDC       480
NatalRGN             QPENLEYRIMLSVHGSQHSGMIVNDTGHETDENRAKVEITPNSPRAEATLGGFGSLGLDC       480
BrasilZKV2015_1      QPENLEYRIMLSVHGSQHSGMIVNDTGHETDENRAKVEITPNSPRAEATLGGFGSLGLDC       480
```

```
Z1106033_1          QPENLEYRIMLSVHGSQHSGMIVNDTGHETDENRAKVEITPNSPRAEATLGGFGSLGLDC     480
PRVABC59_1          QPENLEYRIMLSVHGSQHSGMIVNDTGHETDENRAKVEITPNSPRAEATLGGFGSLGLDC     480
HPF2013_1           QPENLEYRIMLSVHGSQHSGMIVNDTGHETDENRAKVEITPNSPRAEATLGGFGSLGLDC     480
Beh815744_1         QPENLEYRIMLSVHGSQHSGMIVNDTGHETDENRAKVEITPNSPRAEATLGGFGSLGLDC     480
BEH818995_1         QPENLEYRIMLSVHGSQHSGMIVNDTGHETDENRAKVEITPNSPRAEATLGGFGSLGLDC     480
BEH819966_1         QPENLEYRIMLSVHGSQHSGMIVNDTGHETDENRAKVEITPNSPRAEATLGGFGSLGLDC     480
BeH819015_1         QPENLEYRIMLSVHGSQHSGMIVNDTGHETDENRAKVEITPNSPRAEATLGGFGSLGLDC     480
SSABr_1             QPENLEYRIMLSVHGSQHSGMIVNDTGHETDENRAKVEITPNSPRAEATLGGFGSLGLDC     480
ARD157995_1         QPENLEYRIMLPVHGSQHSGMIVNDIGHETDENRAKVEVTPNSPRAEATLGGFGSLGLDC     480
MR_766_1            QPENLEYRIMLSVHGSQHSGMI----GYETDEDRAKVEVTPNSPRAEATLGGFGSLGLDC     476
ARD158084_1         QPENLEYRIMLSVHGSQHSGMIVNDIGHETDENRAKVEVTPNSPRAEATLGGFGSLGLDC     480
ArB1362_1           QPENLEYRIMLSVHGSQHSGMIVNDIGHETDENRAEVEVTPNSPRAEATLGGFGSLGLDC     480
ARB15076_1          QPENLEYRIMLSVHGSQHSGMIVND------ENRAKVEVTPNSPRAEATLGGFGSLGLDC     474
ARB13565_1          QPENLEYRIMLSVHGSQHSGMIVNDIGHETDENRAKVEVTPNSPRAEATLGGFGSLGLDC     480
ARB7701_1           QPENLEYRIMLSVHGSQHSGMIVNDIGHETDENRAKVEVTPNSPRAEATLGGFGSLGLDC     480
IbH30656_1          QPENLEYRIMLSVHGSQHSGMIVND------ENRAKVEVTPNSPRAEATLGGFGSLGLDC     474
ArD128000_1         QPENLEYRIMLSVHGSQHSGMIVNDIGHETDENRAKVEVTPNSPRAEATLGGFGSLGLDC     480
ArD7117_1           QPENLEYRIMLSVHGSQHSGMIVNDIGHETDENRAKVEVTPNSPRAEATLGGFGSLGLDC     480
ARD_41519_1         QPENLEYRIMLSVHGSQHSGMIVNDTGHETDENRAKVEVTPNSPRAEATLGGFGSLGLDC     480
                    *********** **********         *:**:**:*********************

P6-740_1            EPRTGLDFSDLYYLTMNNKHWLVHKEWFHDIPLPWHAGADTGTPHWNNKEALVEFKDAHA     540
P6740               EPRTGLDFSDLYYLTMNNKHWLVHKEWFHDIPLPWHAGADTGTPHWNNKEALVEFKDAHA     540
Yap2007             EPRTGLDFSDLYYLTMNNKHWLVHKEWFHDIPLPWHAGADTGTPHWNNKEALVEFKDAHA     540
CPC0740_1           EPRTGLDFSDLYYLTMNNKHWLVHKEWFHDIPLPWHAGADTGTPHWNNKEALVEFKDAHA     540
SV0127/14_1         EPRTGLDFSDLYYLTMNNKHWLVHKEWFHDIPLPWHTGADTGTPHWNNKEALVEFKDAHA     540
FSS13025_1          EPRTGLDFSDLYYLTMNNKHWLVHKEWFHDIPLPWHAGADTGTPHWNNKEALVEFKDAHA     540
8375_1              EPRTGLDFSDLYYLTMNNKHWLVHKEWFHDIPLPWHAGADTGTPHWNNKEALVEFKDAHA     540
103344_1            EPRTGLDFSDLYYLTMNNKHWLVHKEWFHDIPLPWHAGADTGTPHWNNKEALVEFKDAHA     540
GD01_1              EPRTGLDFSDLYYLTMNNKHWLVHKEWFHDIPLPWHAGADTGTPHWNNKEALVEFKDAHA     540
SPH2015_1           EPRTGLDFSDLYYLTMNNKHWLVHKEWFHDIPLPWHAGADTGTPHWNNKEALVEFKDAHA     540
Haiti2014_1         EPRTGLDFSDLYYLTMNNKHWLVHKEWFHDIPLPWHAGADTGTPHWNNKEALVEFKDAHA     540
PLCal_ZV_1          EPRTGLDFSDLYYLTMNNKHWLVHKEWFHDIPLPWHAGADTGTPHWNNKEALVEFKDAHA     503
Martinique          EPRTGLDFSDLYYLTMNNKHWLVHKEWFHDIPLPWHAGADTGTPHWNNKEALVEFKDAHA     540
NatalRGN            EPRTGLDFSDLYYLTMNNKHWLVHKEWFHDIPLPWHAGADTGTPHWNNKEALVEFKDAHA     540
BrasilZKV2015_1     EPRTGLDFSDLYYLTMNNKHWLVHKEWFHDIPLPWHAGADTGTPHWNNKEALVEFKDAHA     540
Z1106033_1          EPRTGLDFSDLYYLTMNNKHWLVHKEWFHDIPLPWHAGADTGTPHWNNKEALVEFKDAHA     540
PRVABC59_1          EPRTGLDFSDLYYLTMNNKHWLVHKEWFHDIPLPWHAGADTGTPHWNNKEALVEFKDAHA     540
HPF2013_1           EPRTGLDFSDLYYLTMNNKHWLVHKEWFHDIPLPWHAGADTGTPHWNNKEALVEFKDAHA     540
Beh815744_1         EPRTGLDFSDLYYLTMNNKHWLVHKEWFHDIPLPWHAGADTGTPHWNNKEALVEFKDAHA     540
BEH818995_1         EPRTGLDFSDLYYLTMNNKHWLVHKEWFHDIPLPWHAGADTGTPHWNNKEALVEFKDAHA     540
BEH819966_1         EPRTGLDFSDLYYLTMNNKHWLVHKEWFHDIPLPWHAGADTGTPHWNNKEALVEFKDAHA     540
BeH819015_1         EPRTGLDFSDLYYLTMNNKHWLVHKEWFHDIPLPWHAGADTGTPHWNNKEALVEFKDAHA     540
SSABr_1             EPRTGLDFSDLYYLTMNNKHWLVHKEWFHDIPLPWHAGADTGTPHWNNKEALVEFKDAHA     540
ARD157995_1         EPRTGLDFSDLYYLTMNNKHWLVHKEWFHDIPLPWHAGADTGTPHWNNKEALVEFKDAHA     540
MR_766_1            EPRTGLDFSDLYYLTMNNKHWLVHKEWFHDIPLPWHAGADTGTPHWNNKEALVEFKDAHA     536
ARD158084_1         EPRTGLDFSDLYYLTMNNKHWLVHKEWFHDIPLPWHAGADTGTPHWNNKEALVEFKDAHA     540
ArB1362_1           EPRTGLDFSDLYYLTMNNKHWLVHKEWFHDIPLPWHAGADTGTPHWNNKEALVEFKDAHA     540
ARB15076_1          EPRTGLDFSDLYYLTMNNKHWLVHKEWFHDIPLPWHAGADTGTPHWNNKEALVEFKDAHA     534
ARB13565_1          EPRTGLDFSDLYYLTMNNKHWLVHKEWFHDIPLPWHAGADTGTPHWNNKEALVEFKDAHA     540
ARB7701_1           EPRTGLDFSDLYYLTMNNKHWLVHKEWFHDIPLPWHAGADTGTPHWNNKEALVEFKDAHA     540
IbH30656_1          EPRTGLDFSDLYYLTMNNKHWLVHKEWFHDIPLPWHSGADTETPHWNNKEALVEFKDAHA     534
ArD128000_1         EPRTGLDFSDLYYLTMNNKHRLVRKEWFHDIPLPWHAGADTGTPHWNNKEALVEFKDAHA     540
ArD7117_1           EPRTGLDFSDLYYLTMNNKHWLVHKEWFHDIPLPWHAGADTGTPHWNNKEALVEFKDAHA     540
ARD_41519_1         EPRTGLDFSDLYYLTMNNKHWLVHKEWFHDIPLPWHAGADTGTPHWNNKEALVEFKDAHA     540
                    ******************** **:************:**** ******************

P6-740_1            KRQTVVVLGSQEGAVHTALAGALEAEMDGAKGRLSSGHLKCRLKMDKLRLKGVSYSLCTA     600
P6740               KRQTVVVLGSQEGAVHTALAGALEAEMDGAKGRLSSGHLKCRLKMDKLRLKGVSYSLCTA     600
Yap2007             KRQTVVVLGSQEGAVHTALAGALEAEMDGAKGRLSSGHLKCRLKMDKLRLKGVSYSLCTA     600
CPC0740_1           KRQTVVVLGSQEGAVHTALAGALEAEMDGAKGRLSSGHLKCRLKMDKLRLKGVSYSLCTA     600
SV0127/14_1         KRQTVVVLGSQEGAVHTALAGALEAEMDGAKGRLSSGHLKCRLKMDKLRLKGVSYSLCTA     600
FSS13025_1          KRQTVVVLGSQEGAVHTALAGALEAEMDGAKGRLSSGHLKCRLKMDKLRLKGVSYSLCTA     600
8375_1              KRQTVVVLGSQEGAVHTALAGALEAEMDGAKGRLSSGHLKCRLKMDKLRLKGVSYSLCTA     600
103344_1            KRQTVVVLGSQEGAVHTALAGALEAEMDGAKGRLSSGHLKCRLKMDKLRLKGVSYSLCTA     600
GD01_1              KRQTVVVLGSQEGAVHTALAGALEAEMDGAKGRLSSGHLKCRLKMDKLRLKGVSYSLCTA     600
SPH2015_1           KRQTVVVLGSQEGAVHTALAGALEAEMDGAKGRLSSGHLKCRLKMDKLRLKGVSYSLCTA     600
Haiti2014_1         KRQTVVVLGSQEGAVHTALAGALEAEMDGAKGRLSSGHLKCRLKMDKLRLKGVSYSLCTA     600
```

```
PLCal_ZV_1          KRQTVVVLGSQEGAVHTALAGALEAEMDGAKGRLSSGHLKCRLKMDKLRLKGVSYSLCTA    563
Martinique          KRQTVVVLGSQEGAVHTALAGALEAEMDGAKGRLSSGHLKCRLKMDKLRLKGVSYSLCTA    600
NatalRGN            KRQTVVVLGSQEGAVHTALAGALEAEMDGAKGRLSSGHLKCRLKMDKLRLKGVSYSLCTA    600
BrasilZKV2015_1     KRQTVVVLGTQEGAVHTALAGALEAEMDGAKGRLSSGHLKCRLKMDKLRLKGVSYSLCTA    600
Z1106033_1          KRQTVVVLGSQEGAVHTALAGALEAEMDGAKGRLSSGHLKCRLKMDKLRLKGVSYSLCTA    600
PRVABC59_1          KRQTVVVLGSQEGAVHTALAGALEAEMDGAKGRLSSGHLKCRLKMDKLRLKGVSYSLCTA    600
HPF2013_1           KRQTVVVLGSQEGAVHTALAGALEAEMDGAKGRLSSGHLKCRLKMDKLRLKGVSYSLCTA    600
Beh815744_1         KRQTVVVLGSQEGAVHTALAGALEAEMDGAKGRLSSGHLKCRLKMDKLRLKGVSYSLCTA    600
BEH818995_1         KRQTVVVLGSQEGAVHTALAGALEAEMDGAKGRLSSGHLKCRLKMDKLRLKGVSYSLCTA    600
BEH819966_1         KRQTVVVLGSQEGAVHTALAGALEAEMDGAKGRLSSGHLKCRLKMDKLRLKGVSYSLCTA    600
BeH819015_1         KRQTVVVLGSQEGAVHTALAGALEAEMDGAKGRLSSGHLKCRLKMDKLRLKGVSYSLCTA    600
SSABr_1             KRQTVVVLGSQEGAVHTALAGALEAEMDGAKGRLSSGHLKCRLKMDKLRLKGVSYSLCTA    600
ARD157995_1         KRQTVVVLGSQEGAVHTALAGALEAEMDGAKGRLFSGHLKCRLKMDKLRLKGVSYSLCTA    600
MR_766_1            KRQTVVVLGSQEGAVHTALAGALEAEMDGAKGRLFSGHLKCRLKMDKLRLKGVSYSLCTA    596
ARD158084_1         KRQTVVVLGSQEGAVHTALAGALEAEMDGAKGRLFSGHLKCRLKMDKLRLKGVSYSLCTA    600
ArB1362_1           KRQTVVVLGSQEGAVHTALAGALEAEMDGAKGRLFSGHLKCRLKMDKLRLKGVSYSLCTA    600
ARB15076_1          KRQTVVVLGSQEGAVHTALAGALEAEMDGAKGRLFSGHLKCRLKMDKLRLKGVSYSLCTA    594
ARB13565_1          KRQTVVVLGSQEGAVHTALAGALEAEMDGAKGRLFSGHLKCRLKMDKLRLKGVSYSLCTA    600
ARB7701_1           KRQTVVVLGSQEGAVHTALAGALEAEMDGAKGRLFSGHLKCRLKMDKLRLKGVSYSLCTA    600
IbH30656_1          KRQTVVVLGSQEGAVHTALAGALEAEMDGAKGRLSSGHLKCRLKMDKLRLKGVSYSLCTA    594
ArD128000_1         KRQTVVVLGSQEGAVHTALAGALEAEMDGAKGRLFSGHLKCRLKMDKLRLKGVSYSLCTA    600
ArD7117_1           KRQTVVVLGSQEGAVHTALAGALEAEMDGAKGRLFSGHLKCRLKMDKLRLKGVSYSLCTA    600
ARD_41519_1         KRQTVVVLGSQEGAVHTALAGALEAEMDGAKGRLFSGHLKCRLKMDKLRLKGVSYSLCTA    600
                    *********:************************ *************************

P6-740_1            AFTFTKIPAETLHGTVTVEVQYAGTDGPCKVPAQMAVDMQTLTPVGRLITANPVITESTE    660
P6740               AFTFTKIPAETLHGTVTVEVQYAGTDGPCKVPAQMAVDMQTLTPVGRLITANPVITESTE    660
Yap2007             AFTFTKIPAETLHGTVTVEVQYAGTDGPCKVPAQMAVDMQTLTPVGRLITANPVITESTE    660
CPC0740_1           AFTFTKIPAETLHGTVTVEVQYAGTDGPCKVPAQMAVDMQTLTPVGRLITANPVITESTE    660
SV0127/14_1         AFTFTKIPAETLHGTVTVEVQYAGTDGPCKVPAQMAVDMQTLTPVGRLITANPVITEGTE    660
FSS13025_1          AFTFTKIPAETLHGTVTVEVQYAGTDGPCKVPAQMAVDMQTLTPVGRLITANPVITESTE    660
8375_1              AFTFTKIPAETLHGTVTVEVQYAGTDGPCKVPAQMAVDMQTLTPVGRLITANPVITESTE    660
103344_1            AFTFTKIPAETLHGTVTVEVQYAGTDGPCKVPAQMAVDMQTLTPVGRLITANPVITESTE    660
GD01_1              AFTFTKIPAETLHGTVTVEVQYAGTDGPCKVPAQMAVDMQTLTPVGRLITANPVITESTE    660
SPH2015_1           AFTFTKIPAETLHGTVTVEVQYAGTDGPCKVPAQMAVDMQTLTPVGRLITANPVITESTE    660
Haiti2014_1         AFTFTKIPAETLHGTVTVEVQYAGTDGPCKVPAQMAVDMQTLTPVGRLITANPVITESTE    660
PLCal_ZV_1          AFTFTKIPAETLHGTVTVEVQYAGTDGPCKVPAQMAVDMQTLTPVGRLITANPVITESTE    623
Martinique          AFTFTKIPAETLHGTVTVEVQYAGTDGPCKVPAQMAVDMQTLTPVGRLITANPVITESTE    660
NatalRGN            AFTFTKIPAETLHGTVTVEVQYAGTDGPCKVPAQMAVDMQTLTPVGRLITANPVITESTE    660
BrasilZKV2015_1     AFTFTKIPAETLHGTVTVEVQYAGTDGPCKVPAQMAVDMQTLTPVGRLITANPVITESTE    660
Z1106033_1          AFTFTKIPAETLHGTVTVEVQYAGTDGPCKVPAQMAVDMQTLTPVGRLITANPVITESTE    660
PRVABC59_1          AFTFTKIPAETLHGTVTVEVQYAGTDGPCKVPAQMAVDMQTLTPVGRLITANPVITESTE    660
HPF2013_1           AFTFTKIPAETLHGTVTVEVQYAGTDGPCKVPAQMAVDMQTLTPVGRLITANPVITESTE    660
Beh815744_1         AFTFTKIPAETLHGTVTVEVQYAGTDGPCKVPAQMAVDMQTLTPVGRLITANPVITESTE    660
BEH818995_1         AFTFTKIPAETLHGTVTVEVQYAGTDGPCKVPAQMAVDMQTLTPVGRLITANPVITESTE    660
BEH819966_1         AFTFTKIPAETLHGTVTVEVQYAGTDGPCKVPAQMAVDMQTLTPVGRLITANPVITESTE    660
BeH819015_1         AFTFTKIPAETLHGTVTVEVQYAGTDGPCKVPAQMAVDMQTLTPVGRLITANPVITESTE    660
SSABr_1             AFTFTKIPAETLHGTVTVEVQYAGTDGPCKVPAQMAVDMQTLTPVGRLITANPVITESTE    660
ARD157995_1         AFTFTKVPAETLHGTVTVEVQSAGTDGPCKVPAQMAVDMQTLTPVGRLITANPVITESTE    660
MR_766_1            AFTFTKVPAETLHGTVTVEVQYAGTDGPCKIPVQMAVDMQTLTPVGRLITANPVITESTE    656
ARD158084_1         AFTFTKVPAETLHGTVTVEVQYAGTDGPCKVPAQMAVDMQTLTPVGRLITANPVITESTE    660
ArB1362_1           AFTFTKVPAETLHGTVTVEVQYAGTDGPCKVPAQMAVDMQTLTPVGRLITANPVITESTE    660
ARB15076_1          AFTFTKVPAETLHGTVTVEVQYAGTDGPCKVPAQMAVDMQTLTPVGRLITANPVITESTE    654
ARB13565_1          AFTFTKVPAETLHGTVTVEVQYAGTDGPCKVPAQMAVDMQTLTPVGRLITANPVITESTE    660
ARB7701_1           AFTFTKVPAETLHGTVTVEVQYAGTDGPCKVPAQMAVDMQTLTPVGRLITANPVITESTE    660
IbH30656_1          AFTFTKVPAETLHGTVTVEVQYAGRDGPCKVPAQMAVDMQTLTPVGRLITANPVITESTE    654
ArD128000_1         AFTFTKVPAETLHGTVTVEVQYAGTDGPCKVPAQMAVDMQTLTPVGRLITANPVITESTE    660
ArD7117_1           VCTAAKVPAETLHGTVTVEVQYAGTDGPCKVPAQMAVDMQTLTPVGRLITANPVITESTE    660
ARD_41519_1         AFTFTKVPAETLHGTVTVEVQYAGTDGPCKVPAQMAVDMQTLTPVGRLITANPVITESTE    660
                    . * :*:************** ** *****:*.************************.**

P6-740_1            NSKMMLELDPPFGDSYIVIGVGDKKITHHWHRSGSTIGKAFEATVRGAKRMAVLGDTAWD    720
P6740               NSKMMLELDPPFGDSYIVIGVGDKKITHHWXRSGSTIGKAFEATVRGAKRMAVLGDTAWD    720
Yap2007             NSKMMLELDPPFGDSYIVIGVGEKKITHHWHRSGSTIGKAFEATVRGAKRMAVLGDTAWD    720
CPC0740_1           NSKMMLELDPPFGDSYIVIGVGEKKITHHWHRSGSTIGKAFEATVRGAKRMAVLGDTAWD    720
SV0127/14_1         NSKMMLELDPPFGDSYIVIGVGEKKITHHWHRSGSTIGKAFEATVRGAKRMAVLGDTAWD    720
FSS13025_1          NSKMMLELDPPFGDSYIVIGVGEKKITHHWHRSGSTIGKAFEATVRGAKRMAVLGDTAWD    720
8375_1              NSKMMLELDPPFGDSYIVIGVGEKKITHHWHRSGSTIGKAFEATVRGAKRMAVLGDTAWD    720
```

```
103344_1          NSKMMLELDPPFGDSYIVIGVGEKKITHHWHRSGSTIGKAFEATVRGAKRMAVLGDTAWD    720
GD01_1            NSKMMLELDPPFGDSYIVIGVGEKKITHHWHRSGSTIGKAFEATVRGAKRMAVLGDTAWD    720
SPH2015_1         NSKMMLELDPPFGDSYIVIGVGEKKITHHWHRSGSTIGKAFEATVRGAKRMAVLGDTAWD    720
Haiti2014_1       NSKMMLELDPPFGDSYIVIGVGEKKITHHWHRSGSTIGKAFEATVRGAKRMAVLGDTAWD    720
PLCal_ZV_1        NSKMMLELDPPFGDSYIVIGVGEKKITHHWHRSGSTIGKAFEATVRGAKRMAVLGDTAWD    683
Martinique        NSKMMLELDPPFGDSYIVIGVGEKKITHHWHRSGSTIGKAFEATVRGAKRMAVLGDTAWD    720
NatalRGN          NSKMMLELDPPFGDSYIVIGVGEKKITHHWHRSGSTIGKAFEATVRGAKRMAVLGDTAWD    720
BrasilZKV2015_1   NSKMMLELDPPFGDSYIVIGVGEKKITHHWHRSGSTIGKAFEATVRGAKRMAVLGDTAWD    720
Z1106033_1        NSKMMLELDPPFGDSYIVIGVGEKKITHHWHRSGSTIGKAFEATVRGAKRMAVLGDTAWD    720
PRVABC59_1        NSKMMLELDPPFGDSYIVIGVGEKKITHHWHRSGSTIGKAFEATVRGAKRMAVLGDTAWD    720
HPF2013_1         NSKMMLELDPPFGDSYIVIGVGEKKITHHWHRSGSTIGKAFEATVRGAKRMAVLGDTAWD    720
Beh815744_1       NSKMMLELDPPFGDSYIVIGVGEKKITHHWHRSGSTIGKAFEATVRGAKRMAVLGDTAWD    720
BEH818995_1       NSKMMLELDPPFGDSYIVIGVGEKKITHHWHRSGSTIGKAFEATVRGAKRMAVLGDTAWD    720
BEH819966_1       NSKMMLELDPPFGDSYIVIGVGEKKITHHWHRSGSTIGKAFEATVRGAKRMAVLGDTAWD    720
BeH819015_1       NSKMMLELDPPFGDSYIVIGVGEKKITHHWHRSGSTIGKAFEATVRGAKRMAVLGDTAWD    720
SSABr_1           NSKMMLELDPPFGDSYIVIGVGEKKITHHWHRSGSTIGKAFEATVRGAKRMAVLGDTAWD    720
ARD157995_1       NSKMMLELDPPFGDSYIVIGVGDKKITHHWHRSGSTIGKAFEATVRGAKRMAVLGDTAWD    720
MR_766_1          NSKMMLELDPPFGDSYIVIGVGDKKITHHWHRSGSTIGKAFEATVRGAKRMAVLGDTAWD    716
ARD158084_1       NSKMMLELDPPFGDSYIVIGVGDKKITHHWHRSGSTIGKAFEATVRGAKRMAVLGDTAWD    720
ArB1362_1         NSKMMLELDPPFGDSYIVIGVGDKKITHHWHRSGSTIGKAFEATVRGAKRMAVLGDTAWD    720
ARB15076_1        NSKMMLELDPPFGDSYIVIGVGDKKITHHWHRSGSTIGKAFEATVRGAKRMAVLGDTAWD    714
ARB13565_1        NSKMMLELDPPFGDSYIVIGVGDKKITHHWHRSGSTIGKAFEATVRGAKRMAVLGDTAWD    720
ARB7701_1         NSKMMLELDPPFGDSYIVIGVGDKKITHHWHRSGSTIGKAFEATVRGAKRMAVLGDTAWD    720
IbH30656_1        NSKMMLELDPPFGDSYIVIGVGDKKITHHWHRSGSIIGKAFEATVRGAKRMAVLGDTAWD    714
ArD128000_1       NSKMMLELDPPFGDSYIVIGVGDKKITHHWLKKGSSIGKAFEATVRGAKRMAVLGDTAWD    720
ArD7117_1         NSKMMLELDPPFGDSYIVIGVGDKKITHHWHRSGSTIGKAFEATVRGAKRMAVLGDTAWD    720
ARD_41519_1       NSKMMLELDPPFGDSYIVIGVGDKKITHHWHRSGSTIGKAFEATVRGAKRMAVLGDTAWD    720
                  **********************:******* :.** ************************

P6-740_1          FGSVGGALNSLGKGIHQIFGAAFKSLFGGMSWFSQILIGTLLVWLGLNTKNGSISLTCLA    780
P6740             FGSVGGALNSLGKGIHQIFGAAFKSLFGGMSWFSQILIGTLLVWLGLNTKNGSISLTCLA    780
Yap2007           FGSVGGALNSLGKGIHQIFGAAFKSLFGGMSWFSQILIGTLLVWLGLNTKNGSISLTCLA    780
CPC0740_1         FGSVGGALNSLGKGIHQIFGAAFKSLFGGMSWFSQILIGTLLVWLGLNTKNGSISLTCLA    780
SV0127/14_1       FGSVGGVLNSLGKGIHQIFGAAFKSLFGGMSWFSQILIGTLLMWLGLNTKNGSISLMCLA    780
FSS13025_1        FGSVGGALNSLGKGIHQIFGAAFKSLFGGMSWFSQILIGTLLVWLGLNTKNGSISLMCLA    780
8375_1            FGSVGGALNSLGKGIHQIFGAAFKSLFGGMSWFSQILIGTLLMWLGLNTKNGSISLMCLA    780
103344_1          FGSVGGALNSLGKGIHQIFGAAFKSLFGGMSWFSQILIGTLLMWLGLNTKNGSISLMCLA    780
GD01_1            FGSVGGALNSLGKGIHQIFGAAFKSLFGGMSWFSQILIGTLLMWLGLNTKNGSISLMCLA    780
SPH2015_1         FGSVGGALNSLGKGIHQIFGAAFKSLFGGMSWFSQILIGTLLMWLGLNTKNGSISLMCLA    780
Haiti2014_1       FGSVGGALNSLGKGIHQIFGAAFKSLFGGMSWFSQILIGTLLMWLGLNTKNGSISLMCLA    780
PLCal_ZV_1        FGSVGGALNSLGKGIHQIFGAAFKSLFGGMSWFSQILIGTLLMWLGLNTKNGSISLMCLA    743
Martinique        FGSVGGALNSLGKGIHQIFGAAFKSLFGGMSWFSQILIGTLLMWLGLNTKNGSISLMCLA    780
NatalRGN          FGSVGGALNSLGKGIHQIFGAAFKSLFGGMSWFSQILIGTLLMWLGLNTKNGSISLMCLA    780
BrasilZKV2015_1   FGSVGGALNSLGKGIHQIFGAAFKSLFGGMSWFSQILIGTLLMWLGLNTKNGSISLMCLA    780
Z1106033_1        FGSVGGALNSLGKGIHQIFGAAFKSLFGGMSWFSQILIGTLLMWLGLNAKNGSISLMCLA    780
PRVABC59_1        FGSVGGALNSLGKGIHQIFGAAFKSLFGGMSWFSQILIGTLLMWLGLNTKNGSISLMCLA    780
HPF2013_1         FGSVGGALNSLGKGIHQIFGAAFKSLFGGMSWFSQILIGTLLMWLGLNTKNGSISLMCLA    780
Beh815744_1       FGSVGGALNSLGKGIHQIFGAAFKSLFGGMSWFSQILIGTLLMWLGLNTKNGSISLMCLA    780
BEH818995_1       FGSVGGALNSLGKGIHQIFGAAFKSLFGGMSWFSQILIGTLLMWLGLNTKNGSISLMCLA    780
BEH819966_1       FGSVGGALNSLGKGIHQIFGAAFKSLFGGMSWFSQILIGTLLMWLGLNTKNGSISLMCLA    780
BeH819015_1       FGSVGGALNSLGKGIHQIFGAAFKSLFGGMSWFSQILIGTLLMWLGLNTKNGSISLMCLA    780
SSABr_1           FGSVGGALNSLGKGIHQIFGAAFKSLFGGMSWFSQILIGTLLMWLGLNTKNGSISLMCLA    780
ARD157995_1       FGSVGGVFNSLGKGIHQIFGAAFKSLFGGMSWFSQILIGTLLVWLGLNTKNGSISLTCLA    780
MR_766_1          FGSVGGVFNSLGKGIHQIFGAAFKSLFGGMSWFSQILIGTLLVWLGLNTKNGSISLTCLA    776
ARD158084_1       FGSVGGVFNSLGKGIHQIFGAAFKSLFGGMSWFSQILIGTLLVWLGLNTKNGSISLTCLA    780
ArB1362_1         FGSVGGVFNSLGKGIHQIFGAAFKSLFGGMSWFSQILIGTLLVWLGLNTKNGSISLTCLA    780
ARB15076_1        FGSVGGVFNSLGKGIHQIFGAAFKSLFGGMSWFSQILIGTLLVWLGLNTKNGSISLTCLA    774
ARB13565_1        FGSVGGVFNSLGKGVHQIFGAAFKSLFGGMSWFSQILIGTLLVWLGLNTKNGSISLTCLA    780
ARB7701_1         FGSVGGVFNSLGKGVHQIFGAAFKSLFGGMSWFSQILIGTLLVWLGLNTKNGSISLTCLA    780
IbH30656_1        FGSVGGVFNSLGKGIHQIFGAAFKSLFGGMSWFSQILIGTLLVWLGLNTKNGSISLTCLA    774
ArD128000_1       FGSVGGVFNSLGKGVHQIFGAAFKSLFGGMSWFSQILIGTLLVWLGLNTKNGSISLTCLA    780
ArD7117_1         FGSVGGVFNSLGKGIHQIFGAAFKSLFGGMSWFSQILIGTLLVWLGLNTKNGSISLTCLA    780
ARD_41519_1       FGSVGGVFNSLGKGIHQIFGAAFKSLFGGMSWFSQILIGTLLVWLGLNTKNGSISLTCLA    780
                  ******.:******:****************************:*****:******* ***

P6-740_1          LGGVLIFLSTAVSADVGCSVDFSKKETRCGTGVFVYNDVEAWRDRYKYHPDSPRRLAAAV    840
P6740             LGGVLIFLSTAVSADVGCSVDFSKKETRCGTGVFVYNDVEAWRDRYKYHPDSPRRLAAAV    840
Yap2007           LGGVLIFLSTAVSADVGCSVDFSKKETRCGTGVFVYNDVDAWRDRYKYHPDSPRRLAAAV    840
```

```
CPC0740_1          LGGVLIFLSTAVSADVGCSVDFSKKETRCGTGVFVYNDVEAWRDRYKYHPDSPRRLAAAV      840
SV0127/14_1        LGGVLIFLSTAVSADVGCSVDFSKKETRCGTGVFVYNDVEAWRDRYKYHPDSPRRLAAVV      840
FSS13025_1         LGGVLIFLSTAVSADVGCSVDFSKKETRCGTGVFVYNDVEAWRDRYKYHPDSPRRLAAAV      840
8375_1             LGGVLIFLSTAVSADVGCSVDFSKKETRCGTGVFVYNDVEAWRDRYKYHPDSPRRLAAAV      840
103344_1           LGGVLIFLSTAVSADVGCSVDFSKKETRCGTGVFVYNDVEAWRDRYKYHPDSPRRLAAAV      840
GD01_1             LGGVLIFLSTAVSADVGCSVDFSKKETRCGTGVFVYNDVEAWRDRYKYHPDSPRRLAAAV      840
SPH2015_1          LGGVLIFLSTAVSADVGCSVDFSKKETRCGTGVFVYNDVEAWRDRYKYHPDSPRRLAAAV      840
Haiti2014_1        LGGVLIFLSTAVSADVGCSVDFSKKETRCGTGVFVYNDVEAWRDRYKYHPDSPRRLAAAV      840
PLCal_ZV_1         LGGVLIFLSTAVSADVGCSVDFSKKETRCGTGVFVYNDVEAWRDRYKYHPDSPRRLAAAV      803
Martinique         LGGVLIFLSTAVSADVGCSVDFSKKETRCGTGVFVYNDVEAWRDRYKYHPDSPRRLAAAV      840
NatalRGN           LGGVLIFLSTAVSADVGCSVDFSKKETRCGTGVFVYNDVEAWRDRYKYHPDSPRRLAAAV      840
BrasilZKV2015_1    LGGVLIFLSTAVSADVGCSVDFSKKETRCGTGVFVYNDVEAWRDRYKYHPDSPRRLAAAV      840
Z1106033_1         LGGVLIFLSTAVSADVGCSVDFSKKETRCGTGVFVYNDVEAWRDRYKYHPDSPRRLAAAV      840
PRVABC59_1         LGGVLIFLSTAVSADVGCSVDFSKKETRCGTGVFVYNDVEAWRDRYKYHPDSPRRLAAAV      840
HPF2013_1          LGGVLIFLSTAVSADVGCSVDFSKKETRCGTGVFVYNDVEAWRDRYKYHPDSPRRLAAAV      840
Beh815744_1        LGGVLIFLSTAVSADVGCSVDFSKKETRCGTGVFVYNDVEAWRDRYKYHPDSPRRLAAAV      840
BEH818995_1        LGGVLIFLSTAVSADVGCSVDFSKKETRCGTGVFVYNDVEAWRDRYKYHPDSPRRLAAAV      840
BEH819966_1        LGGVLIFLSTAVSADVGCSVDFSKKETRCGTGVFVYNDVEAWRDRYKYHPDSPRRLAAAV      840
BeH819015_1        LGGVLIFLSTAVSADVGCSVDFSKKETRCGTGVFVYNDVEAWRDRYKYHPDSPRRLAAAV      840
SSABr_1            LGGVLIFLSTAVSADVGCSVDFSKKETRCGTGVFVYNDVEAWRDRYKYHPDSPRRLAAAV      840
ARD157995_1        LGGVMIFLSTAVSADVGCSVDFSKKETRCGTGVFIYNDVEAWRDRYKYHPDSPRRLAAAV      840
MR_766_1           LGGVMIFLSTAVSADVGCSVDFSKKETRCGTGVFIYNDVEAWRDRYKYHPDSPRRLAAAV      836
ARD158084_1        LGGVMIFLSTAVSADVGCSVDFSKRETRCGTGVFIYNDVEAWRDRYKYHPDSPRRLAAAV      840
ArB1362_1          LGGVMIFLSTAVSADVGCSVDFSKRETRCGTGVFVYNDVEAWRDRYKYHPDSPRRLAAAV      840
ARB15076_1         LGGVMIFLSTAVSADVGCSVDFSKRETRCGTGVFVYNDVEAWRDRYKYHPDSPRRLAAAV      834
ARB13565_1         LGGVMIFLSTAVSADVGCSVDFSKRETRCGTGVFVYNDVEAWRDRYKYHPDSPRRLAAAV      840
ARB7701_1          LGGVMIFLSTAVSADVGCSVDFSKRETRCGTGVFVYNDVEAWRDRYKYHPDSPRRLAAAV      840
IbH30656_1         LGGVMIFLSTAVSADVGCSVDFSKKETRCGTGVFIYNDVEAWRDRYRYHPDSPRRLAAAV      834
ArD128000_1        LGGVMIFLSTAVSADVGCSVDFSKKETRCGTGVFVYNDVEAWRDRYKYHPDSPRRLAAAV      840
ArD7117_1          LGGVMIFLSTAVSADVGCSVDFSKKETRCGTGVFVYNDVEAWRDRYKYHPDSPRRLAAAV      840
ARD_41519_1        LGGVMIFLSTAVSADVGCSVDFSKKETRCGTGVFVYNDVEAWRDRYKYHPDSPRRLAAAV      840
                   ****:*******************:*********:****:******:***********.*

P6-740_1           KQAWEDGICGISSVSRMENIMWRSVEGELNAILEENGVQLTVVVGSVKNPMWRGPQRLPV      900
P6740              KQAWEDGICGISSVSRMENIMWRSVEGELNAILEENGVQLTVVVGSVKNPMWRGPQRLPV      900
Yap2007            KQAWEDGICGISSVSRMENIMWRSVEGELNAILEENGVQLTVVVGSVKNPMWRGPQRLPV      900
CPC0740_1          KQAWEDGICGISSVSRMENIMWRSVEGELNAILEENGVQLTVVVGSVKNPMWRGPQRLPV      900
SV0127/14_1        KQAWEDGICGISSVSRMENIMWRSVEGELNAILEENGVQLTVVVGSVKNPMWRGPQRLPV      900
FSS13025_1         KQAWEDGICGISSVSRMENIMWRSVEGELNAILEENGVQLTVVVGSVKNPMWRGPQRLPV      900
8375_1             KQAWEDGICGISSVSRMENIMWRSVEGELNAILEENGVQLTVVVGSVKNPMWRAPQRLPV      900
103344_1           KQAWEDGICGISSVSRMENIMWRSVEGELNAILEENGVQLTVVVGSVKNPMWRAPQRLPV      900
GD01_1             KQAWEDGICGISSVSRMENIMWRSVEGELNAILEENGVQLTVVVGSVKNPMWRGPQRLPV      900
SPH2015_1          KQAWEDGICGISSVSRMENIMWRSVEGELNAILEENGVQLTVVVGSVKNPMWRGPQRLPV      900
Haiti2014_1        KQAWEDGICGISSVSRMENIMWRSVEGELNAILEENGVQLTVVVGSVKNPMWRGPQRLPV      900
PLCal_ZV_1         KQAWEDGICGISSVSRMENIMWRSVEGELNAILEENGVQLTVVVGSVKNPMWRGPQRLPV      863
Martinique         KQAWEDGICGISSVSRMENIMWRSVEGELNAILEENGVQLTVVVGSVKNPMWRGPQRLPV      900
NatalRGN           KQAWEDGICGISSVSRMENIMWRSVEGELNAILEENGVQLTVVVGSVKNPMWRGPQRLPV      900
BrasilZKV2015_1    KQAWEDGICGISSVSRMENIMWRSVEGELNAILEENGVQLTVVVGSVKNPMWRGPQRLPV      900
Z1106033_1         KQAWEDGICGISSVSRMENIMWRSVEGELNAILEENGVQLTVVVGSVKNPMWRGPQRLPV      900
PRVABC59_1         KQAWEDGICGISSVSRMENIMWRSVEGELNAILEENGVQLTVVVGSVKNPMWRGPQRLPV      900
HPF2013_1          KQAWEDGICGISSVSRMENIMWRSVEGELNAILEENGVQLTVVVGSVKNPMWRGPQRLPV      900
Beh815744_1        KQAWEDGICGISSVSRMENIMWRSVEGELNAILEENGVQLTVVVGSVKNPMWRGPQRLPV      900
BEH818995_1        KQAWEDGICGISSVSRMENIMWRSVEGELNAILEENGVQLTVVVGSVKNPMWRGPQRLPV      900
BEH819966_1        KQAWEDGICGISSVSRMENIMWRSVEGELNAILEENGVQLTVVVGSVKNPMWRGPQRLPV      900
BeH819015_1        KQAWEDGICGISSVSRMENIMWRSVEGELNAILEENGVQLTVVVGSVKNPMWRGPQRLPV      900
SSABr_1            KQAWEDGICGISSVSRMENIMWRSVEGELNAILEENGVQLTVVVGSVKNPMWRGPQRLPV      900
ARD157995_1        KQAWEEGICGISSVSRMENIMWKSVEGELNAILEENGVQLTVVVGSVKNPMWRGPQRLPV      900
MR_766_1           KQAWEEGICGISSVSRMENIMWKSVEGELNAILEENGVQLTVVVGSVKNPMWRGPQRLPV      896
ARD158084_1        KQAWEEGICGISSVSRMENIMWKSVEGELNAILEENGVQLTVVVGSVKNPMWRGPQRLPV      900
ArB1362_1          KQAWEEGICGISSVSRMENIMWKSVEGELNAILEENGVQLTVVVGSVKNPMWRGPQRLPV      900
ARB15076_1         KQAWEEGICGISSVSRMENIMWKSVEGELNAILEENGVQLTVVVGSVKNPMWRGPQRLPV      894
ARB13565_1         KQAWEEGICGISSVSRMENIMWKSVEGELNAILEENGVQLTVVVGSVKNPMWRGPQRLPV      900
ARB7701_1          KQAWEEGICGISSVSRMENIMWKSVEGELNAILEENGVQLTVVVGSVKNPMWRGPQRLPV      900
IbH30656_1         KQAWEEGICGISSVSRMENIMWKSVEGELNAILEENGVQLTVVVGSVKNPMWRGPRRLPV      894
ArD128000_1        KQAWEEGICGISSVSRMENIMWKSVEGELNAILEENGVQLTVVVGSVKNPMWRGPQRLPV      900
ArD7117_1          KQAWEEGICGISSVSRMENIMWKSVEGELNAILEENGVQLTVVVGSVKNPMWRGPQRLPV      900
ARD_41519_1        KQAWEEGICGISSVSRMENIMWKSVEGELNAILEENGVQLTVVVGSVKNPMWRGPQRLPV      900
                   *****:****************:******************************.*:****
```

```
P6-740_1          PVNELPHGWKAWGKSYFVRAAKTNNSFVVDGDTLKECPLKHRAWNSFLVEDHGFGVFHTS    960
P6740             PVNELPHGWKAWGKSYFVRAAKTNNSFVVDGDTLKECPLKHRAWNSFLVEDHGFGVFHTS    960
Yap2007           PVNELPHGWKAWGKSYFVRAAKTNNSFVVDGDTLKECPLKHRAWNSFLVEDHGFGVFHTS    960
CPC0740_1         PVNELPHGWKAWGKSYFVRAAKTNNSFVVDGDTLKECPLKHRAWNSFLVEDHGFGVFHTS    960
SV0127/14_1       PVNELPHGWKAWGKSYFVRAAKTNNSFVVDGDTLKECPLKHRAWNSFLVEDHGFGVFHTS    960
FSS13025_1        PVNELPHGWKAWGKSYFVRAAKTNNSFVVDGDTLKECPLKHRAWNSFLVEDHGFGVFHTS    960
8375_1            PVNELPHGWKAWGKSYFVRAAKTNNSFVVDGDTLKECPLKHRAWNSFLVEDHGFGVFHTS    960
103344_1          PVNELPHGWKAWGKSYFVRAAKTNNSFVVDGDTLKECPLKHRAWNSFLVEDHGFGVFHTS    960
GD01_1            PVNELPHGWKAWGKSYFVRAAKTNNSFVVDGDTLKECPLKHRAWNSFLVEDHGFGVFHTS    960
SPH2015_1         PVNELPHGWKAWGKSHFVRAAKTNNSFVVDGDTLKECPLKHRAWNSFLVEDHGFGVFHTS    960
Haiti2014_1       PVNELPHGWKAWGKSHFVRAAKTNNSFVVDGDTLKECPLKHRAWNSFLVEDHGFGVFHTS    960
PLCal_ZV_1        PVNELPHGWKAWGKSYFVRAAKTNNSFVVDGDTLKECPLKHRAWNSFLVEDHGFGVFHTS    923
Martinique        PVNELPHGWKAWGKSYFVRAAKTNNSFVVDGDTLKECPLKHRAWNSFLVEDHGFGVFHTS    960
NatalRGN          PVNELPHGWKAWGKSYFVRAAKTNNSFVVDGDTLKECPLEHRAWNSFLVEDHGFGVFHTS    960
BrasilZKV2015_1   PVNELPHGWKAWGKSYFVRAAKTNNSFVVDGDTLKECPLKHRAWNSFLVEDHGFGVFHTS    960
Z1106033_1        PVNELPHGWKAWGKSYFVRAAKTNNSFVVDGDTLKECPLKHRAWNSFLVEDHGFGVFHTS    960
PRVABC59_1        PVNELPHGWKAWGKSYFVRAAKTNNSFVVDGDTLKECPLKHRAWNSFLVEDHGFGVFHTS    960
HPF2013_1         PVNELPHGWKAWGKSYFVRAAKTNNSFVVDGDTLKECPLKHRAWNSFLVEDHGFGVFHTS    960
Beh815744_1       PVNELPHGWKAWGKSYFVRAAKTNNSFVVDGDTLKECPLKHRAWNSFLVEDHGFGVFHTS    960
BEH818995_1       PVNELPHGWKAWGKSYFVRAAKTNNSFVVDGDTLKECPLKHRAWNSFLVEDHGFGVFHTS    960
BEH819966_1       PVNELPHGWKAWGKSYFVRAAKTNNSFVVDGDTLKECPLKHRAWNSFLVEDHGFGVFHTS    960
BeH819015_1       PVNELPHGWKAWGKSYFVRAAKTNNSFVVDGDTLKECPLKHRAWNSFLVEDHGFGVFHTS    960
SSABr_1           PVNELPHGWKAWGKSYFVRAAKTNNSFVVDGDTLKECPLKHRAWNSFLVEDHGFGVFHTS    960
ARD157995_1       PVNELPHGWKAWGKSYFVRAAKTNNSFVVDGDTLKECPLKHRAWNSFLVEDHGFGVFHTS    960
MR_766_1          PVNELPHGWKAWGKSYFVRAAKTNNSFVVDGDTLKECPLEHRAWNSFLVEDHGFGVFHTS    956
ARD158084_1       PVNGLPHGWKAWGKSYFVRAAKTNNSFVVDGDTLKECPLKHRAWNSFLVEDHGFGVFHTS    960
ArB1362_1         PVNELPHGWKAWGKSYFVRAAKTNNSFVVDGDTLKECPLKHRAWNSFLVEDHGFGVFHTS    960
ARB15076_1        PVNELPHGWKAWGKSYFVRAAKTNNRFVVDGDTLKECPLKHRAWNSFLVEDHGFGVFHTS    954
ARB13565_1        PVNELPHGWKAWGKSYFVRAAKTNNSFVVDGDTLKECPLKHRAWNSFLVEDHGFGVFHTS    960
ARB7701_1         PVNELPHGWKAWGKSYFVRAAKTNNSFVVDGDTLKECPLKHRAWNSFLVEDHGFGVFHTS    960
IbH30656_1        PVNELPHGWKAWGKSYFVRAAKTNNSFVVDGDTLKECPLKHRAWNSFLVEDHGFGVFHTS    954
ArD128000_1       PVNELPHGWKAWGKSYFVRAAKTNNSFVVDGDTLKECPLKHRAWNSFLVEDHGFGIFHTS    960
ArD7117_1         PVNELPHGWKAWGKSYFVRAAKTNNSFVVDGDTLKECPLKHRAWNSFLVEDHGFGIFHTS    960
ARD_41519_1       PVNELPHGWKAWGKSYFVRAAKTNNSFVVDGDTLKECPLKHRAWNSFLVEDHGFGIFHTS    960
                  *** ***********:********* *************:***************:****

P6-740_1          VWLKVREDYSLECDPAVIGTAAKGKEAVHSDLGYWIESEKNDTWRLKRAHLIEMKTCEWP    1020
P6740             VWLKVREDYSLECDPAVIGTAAKGKEAVHSDLGYWIESEKNDTWRLKRAHLIEMKTCEWP    1020
Yap2007           VWLKVREDYSLECDPAVIGTAAKGKEAVHSDLGYWIESEKNDTWRLKRAHLIEMKTCEWP    1020
CPC0740_1         VWLKVREDYSLECDPAVIGTAAKGKEAVHSDLGYWIESEKNDTWRLKRAHLIEMKTCEWP    1020
SV0127/14_1       VWLKVREDYSLECDPAVIGTAVKGKEAVHSDLGYWIESEKNDTWRLRRAHLIEMKTCEWP    1020
FSS13025_1        VWLKVREDYSLECDPAVIGTAAKGKEAVHSDLGYWIESEKNDTWRLKRAHLIEMKTCEWP    1020
8375_1            VWLKVREDYSLECDPAVIGTAVKGKEAVHSDLGYWIESEKNDTWRLKRAHLIEMKTCEWP    1020
103344_1          VWLKVREDYSLECDPAVIGTAVKGKEAVHSDLGYWIESEKNDTWRLKRAHLIEMKTCEWP    1020
GD01_1            VWLKVREDYSLECDPAVIGTAVKGKEAVHSDLGYWIESEKNDTWRLKRAHLIEMKTCEWP    1020
SPH2015_1         VWLKVREDYSLECDPAVIGTAVKGKEAVHSDLGYWIESEKNDTWRLKRAHLIEMKTCEWP    1020
Haiti2014_1       VWLKVREDYSLECDPAVIGTAVKGKEAVHSDLGYWIESEKNDTWRLKRAHLIEMKTCEWP    1020
PLCal_ZV_1        VWLKVREDYSLECDPAVIGTAVKGKEAVHSDLGYWIESEKNDTWRLKRAHLIEMKTCEWP    983
Martinique        VWLKVREDYSLECDPAVIGTAVKGKEAVHSDLGYWIESEKNDTWRLKRAHLIEMKTCEWP    1020
NatalRGN          VWLKVREDYSLECDPAVIGTAVKGKEAVHSDLGYWIESEKNDTWRLKRAHLIEMKTCEWP    1020
BrasilZKV2015_1   VWLKVREDYSLECDPAVIGTAVKGKEAVHSDLGYWIESEKNDTWRLKRAHLIEMKTCEWP    1020
Z1106033_1        VWLKVREDYSLECDPAVIGTAVKGKEAVHSDLGYWIESEKNDTWRLKRAHLIEMKTCEWP    1020
PRVABC59_1        VWLKVREDYSLECDPAVIGTAVKGKEAVHSDLGYWIESEKNDTWRLKRAHLIEMKTCEWP    1020
HPF2013_1         VWLKVREDYSLECDPAVIGTAVKGKEAVHSDLGYWIESEKNDTWRLKRAHLIEMKTCEWP    1020
Beh815744_1       VWLKVREDYSLECDPAVIGTAVKGKEAVHSDLGYWIESEKNDTWRLKRAHLIEMKTCEWP    1020
BEH818995_1       VWLKVREDYSLECDPAVIGTAVKGKEAVHSDLGYWIESEKNDTWRLKRAHLIEMKTCEWP    1020
BEH819966_1       VWLKVREDYSLECDPAVIGTAVKGKEAVHSDLGYWIESEKNDTWRLKRAHLIEMKTCEWP    1020
BeH819015_1       VWLKVREDYSLECDPAVIGTAVKGKEAVHSDLGYWIESEKNDTWRLKRAHLIEMKTCEWP    1020
SSABr_1           VWLKVREDYSLECDPAVIGTAVKGKEAVHSDLGYWIESEKNDTWRLKRAHLIEMKTCEWP    1020
ARD157995_1       VWLKVREDYSLECDPAVIGTAVKGREAAHSDLGYWIESEKNDTWRLKRAHLIEMKTCEWP    1020
MR_766_1          VWLKVREDYSLECDPAVIGTAVKGREAAHSDLGYWIESEKNDTWRLKRAHLIEMKTCEWP    1016
ARD158084_1       VWLKVREDYSLECDPAVIGTAVKGREAAHSDLGYWIESEKNDTWRLKRAHLIEMKTCEWP    1020
ArB1362_1         VWLKVREDYSLECDPAVIGTAVKGKEAAHSDLGYWIESEKNDTWRLKRAHLIEMKTCEWP    1020
ARB15076_1        VWLKVREDYSLECDPAVIGTAVKGKEAAHSDLGYWIESEKNDTWRLKRAHLIEMKTCEWP    1014
ARB13565_1        VWLKVREDYSLECDPAVIGTAVKGKEAAHSDLGYWIESEKNDTWRLKRAHLIEMKTCEWP    1020
ARB7701_1         VWLKVREDYSLECDPAVIGTAVKGKEAAHSDLGYWIESEKNDTWRLKRAHLIEMKTCEWP    1020
IbH30656_1        VWLKVREDYSLECDPAVIGTAVKGKEAAHSDLGYWIESEKNDTWRLKRAHLIEMKTCEWP    1014
```

```
ArD128000_1         VWLKVREDYSLECDPAVIGTAVKGKEAAHSDLCYWIESEKNDTWRLRRAHLIEMKTCEWP    1020
ArD7117_1           VWLKVREDYSLECDPAVIGTAVKGKEAAHSDLGYWIESEKNDTWRLRRAHLIEMKTCEWP    1020
ARD_41519_1         VWLKVREDYSLECDPAVIGTAVKGKEAAHSDLGYWIESEKNDTWRLRRAHLIEMKTCEWP    1020
                    *********************.**:**.**** *************:*************

P6-740_1            KSHTLWTDGIEESDLIIPKSLAGPLSHHNTREGYRTQVKGPWHSEELEIRFEECPGTKVH    1080
P6740               KSHTLWTDGIEESDLIIPKSLAGPLSHHNTREGYRTQVKGPWHSEELEIRFEECPGTKVH    1080
Yap2007             KSHTLWTDGIEESDLIIPKSLAGPLSHHNTREGYRTQMKGPWHSEELEIRFEECPGTKVH    1080
CPC0740_1           KSHTLWTDGVEESDLIIPKSLAGPLSHHNTREGYRTQMKGPWHSEELEIRFEECPGTKVH    1080
SV0127/14_1         KSHTLWTDGIEESDLIIPKSLAGPLSHHNTREGYRTQMKGPWHSEELEIRFEECPGTKVH    1080
FSS13025_1          KSHTLWTDGIEESDLIIPKSLAGPLSHHNTREGYRTQMKGPWHSEELEIRFEECPGTKVH    1080
8375_1              KSHTLWTDGIEESDLIIPKSLAGPLSHHNTREGYRTQMKGPWHSEELEIRFEECPGTKVH    1080
103344_1            KSHTLWTDGIEESDLIIPKSLAGPLSHHNTREGYRTQMKGPWHSEELEIRFEECPGTKVH    1080
GD01_1              KSHTLWTDGIEESDLIIPKSLAGPLSHHNTREGYRTQMKGPWHSEELEIRFEECPGTKVH    1080
SPH2015_1           KSHTLWTDGIEESDLIIPKSLAGPLSHHNTREGYRTQMKGPWHSEELEIRFEECPGTKVH    1080
Haiti2014_1         KSHTLWTDGIEESDLIIPKSLAGPLSHHNTREGYRTQMKGPWHSEELEIRFEECPGTKVH    1080
PLCal_ZV_1          KSHTLWTDGIEESDLIIPKSLAGPLSHHNTREGYRTQMKGPWHSEELEIRFEECPGTKVH    1043
Martinique          KSHTLWTDGIEESDLIIPKSLAGPLSHHNTREGYRTQMKGPWHSEELEIRFEECPGTKVH    1080
NatalRGN            KSHTLWADGIEESDLIIPKSLAGPLSHHNTREGYRTQMKGPWHSEELEIRFEECPGTKVH    1080
BrasilZKV2015_1     KSHTLWTDGIEESDLIIPKSLAGPLSHHNTREGYRTQMKGPWHSEELEIRFEECPGTKVH    1080
Z1106033_1          KSHTLWTDGIEESDLIIPKSLAGPLSHHNTREGYRTQMKGPWHSEELEIRFEECPGTKVH    1080
PRVABC59_1          KSHTLWTDGIEESDLIIPKSLAGPLSHHNTREGYRTQMKGPWHSEELEIRFEECPGTKVH    1080
HPF2013_1           KSHTLWTDGIEESDLIIPKSLAGPLSHHNTREGYRTQMKGPWHSEELEIRFEECPGTKVH    1080
Beh815744_1         KSHTLWTDGIEESDLIIPKSLAGPLSHHNTREGYRTQMKGPWHSEELEIRFEECPGTKVH    1080
BEH818995_1         KSHTLWTDGIEESDLIIPKSLAGPLSHHNTREGYRTQMKGPWHSEELEIRFEECPGTKVH    1080
BEH819966_1         KSHTLWTDGIEESDLIIPKSLAGPLSHHNTREGYRTQMKGPWHSEELEIRFEECPGTKVH    1080
BeH819015_1         KSHTLWTDGIEESDLIIPKSLAGPLSHHNTREGYRTQMKGPWHSEELEIRFEECPGTKVH    1080
SSABr_1             KSHTLWTDGIEESDLIIPKSLAGPLSHHNTREGYRTQMKGPWHSEELEIRFEECPGTKVH    1080
ARD157995_1         KSHTLWTDGVEESDLIIPKSLAGPLSHHNTREGYRTQVKGPWHSEELEIRFEECPGTKVH    1080
MR_766_1            KSHTLWTDGVEESDLIIPKSLAGPLSHHNTREGYRTQVKGPWHSEELEIRFEECPGTKVY    1076
ARD158084_1         KSHTLWTDGVEESDLIIPKSLAGPLSHHNTREGYRTQVKGPWHSEELEIRFEECPGTKVH    1080
ArB1362_1           KSHTLWTDGVEESDLIIPKSLAGPLSHHNTREGYRTQAKGPWHSEELEIRFEECPGTKVH    1080
ARB15076_1          KSHTLWTDGVEESDLIIPKSLAGPLSHHNTREGYRTQAKGPWHSEELEIRFEECPGTKVH    1074
ARB13565_1          KSHTLWTDGVEESDLIIPKSLAGPLSHHNTREGYRTQAKGPWHSEELEIRFEECPGTKVH    1080
ARB7701_1           KSHTLWTDGVEESDLIIPKSLAGPLSHHNTREGYRTQAKGPWHSEELEIRFEECPGTKVH    1080
IbH30656_1          KSHTLWTDGVEESDLIIPKSLAGPLSHHNTREGYRTQVKGPWHSEELEIRFEECPGTKVH    1074
ArD128000_1         KSHTLWTDGVEESDLIIPKSLAGPLSHHNTREGYRTQVKGPWHSEELEIRFEECPGTKVY    1080
ArD7117_1           KSHTLWTDGVEESDLIIPKSLAGPLSHHNTREGYRTQVKGPWHSEELEIRFEECPGTKVH    1080
ARD_41519_1         KSHTLWTDGVEESDLIIPKSLAGPLSHHNTREGYRTQLKGPWHSEELEIRFEECPGTKVH    1080
                    ******:**:*************************** **********************:

P6-740_1            VEETCGTRGPSLRSTTASGRVIEEWCCRECTMPPLSFRAKDGCWYGMEIRPRKEPESNLV    1140
P6740               VEETCGTRGPSLRSTTASGRVIEEWCCRECTMPPLSFRAKDGCWYGMEIRPRKEPESNLV    1140
Yap2007             VEETCGTRGPSLRSTTASGRVIEEWCCRECTMPPLSFRAKDGCWYGMEIRPRKEPESNLV    1140
CPC0740_1           VEETCGTRGPSLRSTTASGRVIEEWCCRECTMPPLSFRAKDGCWYGMEIRPRKEPESNLV    1140
SV0127/14_1         VEETCGTRGPSLRSTTASGRVIEEWCCRECTMPPLSFRAKDGCWYGMEIRPRKEPESNLV    1140
FSS13025_1          VEETCGTRGPSLRSTTASGRVIEEWCCRECTMPPLSFRAKDGCWYGMEIRPRKEPESNLV    1140
8375_1              VEETCGTRGPSLRSTTASGRVIEEWCCRECTMPPLSFRAKDGCWYGMEIRPRKEPESNLV    1140
103344_1            VEETCGTRGPSLRSTTASGRVIEEWCCRECTMPPLSFRAKDGCWYGMEIRPRKEPESNLV    1140
GD01_1              VEETCGTRGPSLRSTTASGRVIEEWCCRECTMPPLSFRAKDGCWYGMEIRPRKEPESNLV    1140
SPH2015_1           VEETCGTRGPSLRSTTASGRVIEEWCCRECTMPPLSFRAKDGCWYGMEIRPRKEPESNLV    1140
Haiti2014_1         VEETCGTRGPSLRSTTASGRVIEEWCCRECTMPPLSFRAKDGCWYGMEIRPRKEPESNLV    1140
PLCal_ZV_1          VEETCGTRGPSLRSTTASGRVIEEWCCRECTMPPLSFRAKDGCWYGMEIRPRKEPESNLV    1103
Martinique          VEETCGTRGPSLRSTTASGRVIEEWCCRECTMPPLSFWAKDGCWYGMEIRPRKEPESNLV    1140
NatalRGN            VEETCGTRGPSLRSTTASGRVIEEWCCRECTMPPLSFRAKDGCWYGMEIRPRKEPESNLV    1140
BrasilZKV2015_1     VEETCGTRGPSLRSTTASGRVIEEWCCRECTMPPLSFRAKDGCWYGMEIRPRKEPESNLV    1140
Z1106033_1          VEETCGTRGPSLRSTTASGRVIEEWCCRECTMPPLSFRAKDGCWYGMEIRPRKEPESNLV    1140
PRVABC59_1          VEETCGTRGPSLRSTTASGRVIEEWCCRECTMPPLSFRAKDGCWYGMEIRPRKEPESNLV    1140
HPF2013_1           VEETCGTRGPSLRSTTASGRVIEEWCCRECTMPPLSFRAKDGCWYGMEIRPRKEPESNLV    1140
Beh815744_1         VEETCGTRGPSLRSTTASGRVIEEWCCRECTMPPLSFRAKDGCWYGMEIRPRKEPESNLV    1140
BEH818995_1         VEETCGTRGPSLRSTTASGRVIEEWCCRECTMPPLSFRAKDGCWYGMEIRPRKEPESNLV    1140
BEH819966_1         VEETCGTRGPSLRSTTASGRVIEEWCCRECTMPPLSFRAKDGCWYGMEIRPRKEPESNLV    1140
BeH819015_1         VEETCGTRGPSLRSTTASGRVIEEWCCRECTMPPLSFRAKDGCWYGMEIRPRKEPESNLV    1140
SSABr_1             VEETCGTRGPSLRSTTASGRVIEEWCCRECTMPPLSFRAKDGCWYGMEIRPRKEPESNLV    1140
ARD157995_1         VEETCGTRGPSLRSTTASGRVIEEWCCRECTMPPLSFRAKDGCWYGMEIRPRKEPESNLV    1140
MR_766_1            VEETCGTRGPSLRSTTASGRVIEEWCCRECTMPPLSFRAKDGCWYGMEIRPRKEPESNLV    1136
ARD158084_1         VEETCGTRGPSLRSTTASGRVIEEWCCRECTMPPLSFRAKDGCWYGMEIRPRKEPESNLV    1140
ArB1362_1           VEETCGTRGPSLRSTTASGRVIEEWCCRECTMPPLSFRAKDGCWYGMEIRPRKEPESNLV    1140
```

```
ARB15076_1          VEETCGTRGPSLRSTTASGRVIEEWCCRECTMPPLSFRAKDGCWYGMEIRPRKEPESNLV       1134
ARB13565_1          VEETCGTRGPSLRSTTASGRVIEEWCCRECTMPPLSFRAKDGCWYGMEIRPRKEPESNLV       1140
ARB7701_1           VEETCGTRGPSLRSTTASGRVIEEWCCRECTMPPLSFRAKDGCWYGMEIRPRKEPESNLV       1140
IbH30656_1          VEETCGTRGPSLRSTTASGRVIEEWCCRECTMPPLSFRAKDGCWYGMEIRPRKEPESNLV       1134
ArD128000_1         VEETCGTRGPSLRSTTASGRVIEEWCCRECTMPPLSFRAKDGCWYGMEIRPRKEPESNLV       1140
ArD7117_1           VEETCGTRGPSLRSTTASGRVIEEWCCRECTMPPLSFRAKDGCWYGMEIRPRKEPESNLV       1140
ARD_41519_1         VEETCGTRGPSLRSTTASGRVIEEWCCRECTMPPLSFRAKDGCWYGMEIRPRKEPESNLV       1140
                    ************************************* **********************

P6-740_1            RSMVTAGSTDHMDHFSLGVLVILLMVQEGLKKRMTTKIIISTSMAVLVAMILGGFSMSDL       1200
P6740               RSMVTAGSTDHMDHFSLGVLVILLMVQEGLKKRMTTKIIISTSMAVLVAMILGGFSMSDL       1200
Yap2007             RSMVTAGSTDHMDHFSLGVLVILLMVQEGLKKRMTTKIIISTSMAVLVAMILGGFSMSDL       1200
CPC0740_1           RSMVTAGSTDHMDHFSLGVLVILLMVQEGLKKRMTTKIIISTSMAVLVAMILGGFSMSDL       1200
SV0127/14_1         RSMVTAGSTDHMDHFSLGVLVILLMVQEGLKKRMTTKIIISTSMAVLVAMILGGFSMSDL       1200
FSS13025_1          RSMVTAGSTDHMDHFSLGVLVILLMVQEGLKKRMTTKIIISTSMAVLVAMILGGFSMSDL       1200
8375_1              RSMVTAGSTDHMDHFSLGVLVILLMVQEGLKKRMTTKIIISTSMAVLVAMILGGFSMSDL       1200
103344_1            RSMVTAGSTDHMDHFSLGVLVILLMVQEGLKKRMTTKIIISTSMAVLVAMILGGFSMSDL       1200
GD01_1              RSMVTAGSTDHMDHFSLGVLVILLMVQEGLKKRMTTKIIISTSMAVLVAMILGGFSMSDL       1200
SPH2015_1           RSMVTAGSTDHMDHFSLGVLVILLMVQEGLKKRMTTKIIISTSMAVLVAMILGGFSMSDL       1200
Haiti2014_1         RSMVTAGSTDHMDHFSLGVLVILLMVQEGLKKRMTTKIIISTSMAVLVAMILGGFSMSDL       1200
PLCal_ZV_1          RSMVTAGSTDHMDHFSLGVLVILLMVQEGLKKRMTTKIIISTSMAVLVAMILGGFSMSDL       1163
Martinique          RSMVTAGSTDHMDHFSLGVLVILLMVQEGLKKRMTTKIIISTSMAVLVAMILGGFSMSDL       1200
NatalRGN            RSVVTAGSTDHMDHFSLGVLVILLMVQEGLKKRMTTKIIISTSMAVLVAMILGGFSMSDL       1200
BrasilZKV2015_1     RSMVTAGSTDHMDHFSLGVLVILLMVQEGLKKRMTTKIIISTSMAVLVAMILGGFSMSDL       1200
Z1106033_1          RSMVTAGSTDHMDHFSLGVLVILLMVQEGLKKRMTTKIIISTSMAVLVAMILGGFSMSDL       1200
PRVABC59_1          RSMVTAGSTDHMDHFSLGVLVILLMVQEGLKKRMTTKIIISTSMAVLVAMILGGFSMSDL       1200
HPF2013_1           RSMVTAGSTDHMDHFSLGVLVILLMVQEGLKKRMTTKIIISTSMAVLVAMILGGFSMSDL       1200
Beh815744_1         RSMVTAGSTDHMDHFSLGVLVILLMVQEGLKKRMTTKIIISTSMAVLVAMILGGFSMSDL       1200
BEH818995_1         RSMVTAGSTDHMDHFSLGVLVILLMVQEGLKKRMTTKIIISTSMAVLVAMILGGFSMSDL       1200
BEH819966_1         RSMVTAGSTDHMDHFSLGVLVILLMVQEGLKKRMTTKIIISTSMAVLVAMILGGFSMSDL       1200
BeH819015_1         RSMVTAGSTDHMDHFSLGVLVILLMVQEGLKKRMTTKIIISTSMAVLVAMILGGFSMSDL       1200
SSABr_1             RSMVTAGSTDHMDHFSLGVLVILLMVQEGLKKRMTTKIIISTSMAVLVAMILGGFSMSDL       1200
ARD157995_1         RSMVTAGSTDHMDHFSLGVLVILLMVQEGLKKRMTTKIIMSTSMAVLVVMILGGFSMSDL       1200
MR_766_1            RSMVTAGSTDHMDHFSLGVLVILLMVQEGLKKRMTTKIIMSTSMAVLVVMILGGFSMSDL       1196
ARD158084_1         RSMVTAGSTDHMDHFSLGVLVILLMVQEGLKKRMTTKIIMSTSMAVLVVMILGGFSMSDL       1200
ArB1362_1           RSMVTAGSTDHMDHFSLGVLVILLMVQEGLKKRMTTKIIVSTSMAVLVAMILGGFSMSDL       1200
ARB15076_1          RSMVTAGSTDHMDHFSLGVLVILLMVQEGLKKRMTTKIIMSTSMAVLVAMILGGFSMSDL       1194
ARB13565_1          RSMVTAGSTDHMDHFSLGVLVILLMVQEGLKKRMTTKIIMSTSMAVLVAMILGGFLMSDL       1200
ARB7701_1           RSMVTAGSTDHMDHFSLGVLVILLMVXEGLKKRMTTKIIMSTSMAVLVAMILGGFLMSDL       1200
IbH30656_1          RSMVTAGSTDHMDHFSLGVLVILLMVQEGLKKRMTTKIIMSTSMAMLVAMVLGGFSMSDL       1194
ArD128000_1         RSMVTAGSTDHMDHFSLGVLVILLMVQEGLKKRMTTKIIMSTSMAVLVAMVLGGFSMSDL       1200
ArD7117_1           RSMVTAGSTDHMDHFSLGVLVILLMVQEGLKKRMTTKIIMSTSMAVLVAMVLGGFSMSDL       1200
ARD_41519_1         RSMVTAGSTDHMDHFSLGVLVILLMVQEGLKKRMTTKIIMSTSMAVLVAMVLGGFSMSDL       1200
                    **:*********************** ************:*****:**.*:**** ****

P6-740_1            AKLAILMGATFAEMNTGGDVAHLALIAAFKVRPALLVSFIFRANWTPRESMLLALASCLL       1260
P6740               AKLAILMGATFAEMNTGGDVAHLALIAAFKVRPALLVSFIFRANWTPRESMLLALASCLL       1260
Yap2007             AKLAILMGATFAEMNTGGDVAHLALIAAFKVRPALLVSFIFRANWTPRESMLLALASCLL       1260
CPC0740_1           AKLAILMGATFAEMNTGGDVAHLALIAAFKVRPALLVSFIFRANWTPRESMLLALASCLL       1260
SV0127/14_1         AKLAILMGATFAEMNTGGDVAHLALVAAFKVRPALLVSFIFRANWTPRESMLLALASCLL       1260
FSS13025_1          AKLAILMGATFAEMNTGGDVAHLALIAAFKVRPALLVSFIFRANWTPRESMLLALASCLL       1260
8375_1              AKLAILMGATFAEMNTGGDVAHLALIAAFKVRPALLVSFIFRANWTPRESMLLALASCLL       1260
103344_1            AKLAILMGATFAEMNTGGDVAHLALIAAFKVRPALLVSFIFRANWTPRESMLLALASCLL       1260
GD01_1              AKLAILMGATFAEMNTGGDVAHLALIAAFKVRPALLVSFIFRANWTPRESMLLALASCLL       1260
SPH2015_1           AKLAILMGATFAEMNTGGDVAHLALIAAFKVRPALLVSFIFRANWTPRESMLLALASCLL       1260
Haiti2014_1         AKLAILMGATFAEMNTGGDVAHLALIAAFKVRPALLVSFIFRANWTPRESMLLALASCLL       1260
PLCal_ZV_1          AKLAILMGATFAEMNTGGDVAHLALIAAFKVRPALLVSFIFRANWTPRESMLLALASCLL       1223
Martinique          AKLAILMGATFAEMNTGGDVAHLALTAAFKVRPALLVSFIFRANWTPRESMLLALASCLL       1260
NatalRGN            AKLAILMGATFAEMNTGGDVAHLALIAAFKVRPALLVSFIFRANWTPRESMLLALASCLL       1260
BrasilZKV2015_1     AKLAILMGATFAEMNTGGDVAHLALIAAFKVRPALLVSFIFRANWTPRESMLLALASCFL       1260
Z1106033_1          AKLAILMGATFAEMNTGGDVAHLALIAAFKVRPALLVSFIFRANWTPRESMLLALASCLL       1260
PRVABC59_1          AKLAILMGATFAEMNTGGDVAHLALIAAFKVRPALLVSFIFRANWTPRESMLLALASCLL       1260
HPF2013_1           AKLAILMGATFAEMNTGGDVAHLALIAAFKVRPALLVSFIFRANWTPRESMLLALASCLL       1260
Beh815744_1         AKLAILMGATFAEMNTGGDVAHLALIAAFKVRPALLVSFIFRANWTPRESMLLALASCLL       1260
BEH818995_1         AKLAILMGATFAEMNTGGDVAHLALIAAFKVRPALLVSFIFRANWTPRESMLLALASCLL       1260
BEH819966_1         AKLAILMGATFAEMNTGGDVAHLALIAAFKVRPALLVSFIFRANWTPRESMLLALASCLL       1260
BeH819015_1         AKLAILMGATFAEMNTGGDVAHLALIAAFKVRPALLVSFIFRANWTPRESMLLALASCLL       1260
SSABr_1             AKLAILMGATFAEMNTGGDVAHLALIAAFKVRPALLVSFIFRANWTPRESMLLALASCLL       1260
```

```
ARD157995_1         AKLVILMGATFAEMNTGGDVAHLALVAAFKVRPALLVSFILRANWTPRESMLLALASCLL      1260
MR_766_1            AKLVILMGATFAEMNTGGDVAHLALVAAFKVRPALLVSFIFRANWTPRESMLLALASCLL      1256
ARD158084_1         AKLVILMGATFAEMNTGGDVAHLALVAAFKVRPALLVSFILRANWTPRESMLLALASCLL      1260
ArB1362_1           AKLVILMGATFAEMNTGGDVAHLALVAAFKVRPALLVSFIFRANWTPRESMLLALASCLL      1260
ARB15076_1          AKLVILMGATFAEMNTGGDVAHLALVAAFKVRPALLVSFIFRANWTPRESMLLALASCLL      1254
ARB13565_1          AKLVILMGATFAEMNTGGDVAHLALVAAFKVRPALLVSFIFRANWTPRESMLLALASCLL      1260
ARB7701_1           AKLVILMGATFAEMNTGGDVAHLALVAAFKVRPALLVSFIFRANWTPRESMLLALASCLL      1260
IbH30656_1          AKLVILMGATFAEMNTGGDVAHLALVAAFKVRPALLVSFIFRANWTPRESMLLALASCLL      1254
ArD128000_1         AKLVILMGATFAEMNTGGDVAHLALVAAFKVRPALLVSFIFRANWTPRESMLLALASCLL      1260
ArD7117_1           AKLVILMGATFAEMNTGGDVAHLALVAAFKVRPALLVSFIFRANWTPRESMLLALASCLL      1260
ARD_41519_1         AKLVILMGATFAEMNTGGDVAHLALVAAFKVRPALLVSFIFRANWTPRESMLLALASCLL      1260
                    ***.******************** ***************:*****************:*

P6-740_1            QTAISALEGDLMVLINGFALAWLAIRAMAVPRTDNITLAILAALTPLARGTLLVAWRAGL      1320
P6740               QTXISALEGDLMVLINGFALAWLAIRAMAVPRTDNITLAILAALTPLARGTLLVAWRAGL      1320
Yap2007             QTAISALEGDLMVLINGFALAWLAIRAMVVPRTDNITLAILTALTPLARGTLLVAWRAGL      1320
CPC0740_1           QTAISALEGDLMVLINGFALAWLAIRAMVVPRTDNITLAILAALTPLARGTLLVAWRAGL      1320
SV0127/14_1         QTAISALEGDLMVLINGFALAWLAIRAMVVPRTDNITLAILAALTPLARGTLLVAWRAGL      1320
FSS13025_1          QTAISALEGDLMVPINGFALAWLAIRAMVVPRTDNITLAILAALTPLARGTLLVAWRAGL      1320
8375_1              QTAISALEGDLMVLINGFALAWLAIRAMVVPRTDNITLAILAALTPLARGTLLVAWRAGL      1320
103344_1            QTAISALEGDLMVLINGFALAWLAIRAMVVPRTDNITLAILAALTPLARGTLLVAWRAGL      1320
GD01_1              QTAISALEGDLMVLINGFALAWLAVRAMVVPRTDNITLAILAALTPLARGTLLVAWRAGL      1320
SPH2015_1           QTAISALEGDLMVLINGFALAWLAIRAMVVPRTDNITLAILAALTPLARGTLLVAWRAGL      1320
Haiti2014_1         QTAISALEGDLMVLINGFALAWLAIRAMVVPRTDNITLAILAALTPLARGTLLVAWRAGL      1320
PLCal_ZV_1          QTAISALEGDLMVLINGFALAWLAIRAMVVPRTDNITLAILAALTPLARGTLLVAWRAGL      1283
Martinique          QTAISALEGDLMVLINGFALAWLAIRAMVVPRTDNITLAILAALTPLARGTLLVAWRAGL      1320
NatalRGN            QTAISALEGDLMVLINGFALAWLAIRAMVVPRTDNITLAILAALTPLARGTLLVAWRAGL      1320
BrasilZKV2015_1     QTAISALEGDLMVLINGFALAWLAIRAMVVPRTDNITLAILAALTPLARGTLLVAWRAGL      1320
Z1106033_1          QTAISALEGDLMVLINGFALAWLAIRAMVVPRTDNITLAILAALTPLARGTLLVAWRAGL      1320
PRVABC59_1          QTAISALEGDLMVLINGFALAWLAIRAMVVPRTDNITLAILAALTPLARGTLLVAWRAGL      1320
HPF2013_1           QTAISALEGDLMVLINGFALAWLAIRAMVVPRTDNITLAILAALTPLARGTLLVAWRAGL      1320
Beh815744_1         QTAISALEGDLMVLINGFALAWLAIRAMVVPRTDNITLAILAALTPLARGTLLVAWRAGL      1320
BEH818995_1         QTAISALEGDLMVLINGFALAWLAIRAMVVPRTDNITLAILAALTPLARGTLLVAWRAGL      1320
BEH819966_1         QTAISALEGDLMVLINGFALAWLAIRAMVVPRTDNITLAILAALTPLARGTLLVAWRAGL      1320
BeH819015_1         QTAISALEGDLMVLINGFALAWLAIRAMVVPRTDNITLAILAALTPLARGTLLVAWRAGL      1320
SSABr_1             QTAISALEGDLMVLINGFALAWLAIRAMVVPRTDNITLAILAALTPLARGTLLVAWRAGL      1320
ARD157995_1         QTAISALEGDLMVLVNGFALAWLAIRAMAVPRTDNIALAILAALTPLARGTLLVAWRAGL      1320
MR_766_1            QTAISALEGDLMVLINGFALAWLAIRAMAVPRTDNIALPILAALTPLARGTLLVAWRAGL      1316
ARD158084_1         QTAISALEGDLMVLVNGFALAWLAIRAMAVPRTDNIALAILAALTPLARGTLLVAWRAGL      1320
ArB1362_1           QTAISALEGDLMVLVNGFALAWLAIRAMAVPRTDNIALAILAALTPLARGTLLVAWRAGL      1320
ARB15076_1          QTAISALEGDLMVLVNGFALAWLAIRAMAVPRTDNIALAVLAALTPLARGTLLVAWRAGL      1314
ARB13565_1          QTAISALEGDLMVLVNGFALAWLAIRAMAVPRTDNIALAILAALTPLARGTLLVAWRAGL      1320
ARB7701_1           QTAISALEGDLMVLVNGFALAWLAIRAMAVPRTDNIALAILAALTPLARGTLLVAWRAGL      1320
IbH30656_1          QTAISALEGELMVLVNGFALAWLAIRAMAVPRTDNIALAILAALTPLARGTLLVAWRAGL      1314
ArD128000_1         QTAISALEGELMVLVNGFALAWLAIRAMAVPRTDNIALATLAALTPLARGTLLVAWRAGL      1320
ArD7117_1           QTAISALEGELMVLVNGFALAWLAIRAMAVPRTDNIALAILAALTPLARGTLLVAWRAGL      1320
ARD_41519_1         QTAISALEGELMVLVNGFALAWLAIRAMAVPRTDNIALAILAALTPLARGTLLVAWRAGL      1320
                    ** ******:*** :*********:***.*******:*  *:******************

P6-740_1            ATCGGFMLLSLKGKGSVKKNLPFVMALGLTAVRLVDPINVVGLLLLTRSGKRSWPPSEVL      1380
P6740               ATCGGFMLLSLKGKGSVKKNLPFVMALGLTAVRLVDPINVVGLLLLTRSGKRSWPPSEVL      1380
Yap2007             ATCGGFMLLSLKGKGSVKKNLPFVMALGLTAVRLVDPINVVGLLLLTRSGKRSWPPSEVL      1380
CPC0740_1           ATCGGFMLLSLKGKGSVKKNLPFVMALGLTAVRLVDPINVVGLLLLTRSGKRSWPPSEVL      1380
SV0127/14_1         ATCGGFMLLSLKGKGSVKKNLPFVMALGLTAVRLVDPINVVGLLLLTRSGKRSWPPSEVL      1380
FSS13025_1          ATCGGFMLLSLKGKGSVKKNLPFVMALGLTAVRLVDPINVVGLLLLTRSGKRSWPPSEVL      1380
8375_1              ATCGGFMLLSLKGKGSVKKNLPFVMALGLTAVRLVDPINVVGLLLLTRSGKRSWPPSEVL      1380
103344_1            ATCGGFMLLSLKGKGSVKKNLPFVMALGLTAVRLVDPINVVGLLLLTRSGKRSWPPSEVL      1380
GD01_1              ATCGGFMLLSLKGKGSVKKNLPFVMALGLTAVRLVDPINVVGLLLLTRSGKRSWPPSEVL      1380
SPH2015_1           ATCGGFMLLSLKGKGSVKKNLPFVMALGLTAVRLVDPINVVGLLLLTRSGKRSWPPSEVL      1380
Haiti2014_1         ATCGGFMLLSLKGKGSVKKNLPFVMALGLTAVRLVDPINVVGLLLLTRSGKRSWPPSEVL      1380
PLCal_ZV_1          ATCGGFMLLSLKGKGSVKKNLPFVMALGLTAVRLVDPINVVGLLLLTRSGKRSWPPSEVL      1343
Martinique          ATCGGFMLLSLKGKGSVKKNLPFVMALGLTAVRLVDPINVVGLLLLTRSGKRSWPPSEVL      1380
NatalRGN            ATCGGFMLLSLKGKGSVKKNLPFVMALGLTAVRLVDPINVVGLLLLTRSGKRSWPPSEVL      1380
BrasilZKV2015_1     ATCGGFMLLSLKGKGSVKKNLPFVMALGLTAVRLVDPINVVGLLLLTRSGKRSWPPSEVL      1380
Z1106033_1          ATCGGFMLLSLKGKGSVKKNLPFVMALGLTAVRLVDPINVVGLLLLTRSGKRSWPPSEVL      1380
PRVABC59_1          ATCGGFMLLSLKGKGSVKKNLPFVMALGLTAVRLVDPINVVGLLLLTRSGKRSWPPSEVL      1380
HPF2013_1           ATCGGFMLLSLKGKGSVKKNLPFVMALGLTAVRLVDPINVVGLLLLTRSGKRSWPPSEVL      1380
Beh815744_1         ATCGGFMLLSLKGKGSVKKNLPFVMALGLTAVRLVDPINVVGLLLLTRSGKRSWPPSEVL      1380
```

```
BEH818995_1        ATCGGFMLLSLKGKGSVKKNLPFVMALGLTAVRLVDPINVVGLLLLTRSGKRSWPPSEVL    1380
BEH819966_1        ATCGGFMLLSLKGKGSVKKNLPFVMALGLTAVRLVDPINVVGLLLLTRSGKRSWPPSEVL    1380
BeH819015_1        ATCGGFMLLSLKGKGSVKKNLPFVMALGLTAVRLVDPINVVGLLLLTRSGKRSWPPSEVL    1380
SSABr_1            ATCGGFMLLSLKGKGSVKKNLPFVMALGLTAVRLVDPINVVGLLLLTRSGKRSWPPSEVL    1380
ARD157995_1        ATCGGFMLLSLKGKGSVKKNLPFVMALGLTAVRVVDPINVVGLLLLTRSGKRSWPPSEVL    1380
MR_766_1           ATCGGIMLLSLKGKGSVKKNLPFVMALGLTAVRVVDPINVVGLLLLTRSGKRSWPPSEVL    1376
ARD158084_1        ATCGGFMLLSLKGKGSVKKNLPFVMALGLTAVRVVDPINVVGLLLLTRSGKRSWPPSEVL    1380
ArB1362_1          ATCGGFMLLSLKGKGSVKKNLPFVMALGMTAVRIVDPINVVGLLLLTRSGKRSWPPSEVL    1380
ARB15076_1         ATCGGFMLLSLKGKGSVKKNLPFVMALGLTAVRIVDPINVVGLLLLTRSGKRSWPPSEVL    1374
ARB13565_1         ATCGGFMLLSLKGKGSVKKNLPFVMALGLTAVRIVDPINVVGLLLLTRSGKRSWPPSEVL    1380
ARB7701_1          ATCGGFMLLSLKGKGSVKKNLPFVMALGLTAVRIVDPINVVGLLLLTRSGKRSWPPSEVL    1380
IbH30656_1         ATCGGFMLISLKGKGSVKKNLPFVMALGLTAVRIVDPINVVGLLLLTRSGKRSWPPSEVL    1374
ArD128000_1        ATCGGFMLLSLKGKGSVKKNLPFVMALGLAAVRIVDPINVVGLLLLTRSGKRSWPPSEVL    1380
ArD7117_1          ATCGGFMLLSLKGKGSVKKNLPFVMALGLTAVRIVDPINVVGLLLLTRSGKRSWPPSEVL    1380
ARD_41519_1        ATCGGFMLLSLKGKGSVKKNLPFVMALGLTAVRIVDPINVVGLLLLTRSGKRSWPPSEVL    1380
                   *****:**:*******************::***:**************************

P6-740_1           TAVGLICALAGGFAKADIEMAGPMAAVGLLIVSYVVSGKSVDMYIERAGDITWEKDAEVT    1440
P6740              TAVGLICALAGGFAKADIEMAGPMAAVGLLIVSYVVSGKSVDMYIERAGDITWEKDAEVT    1440
Yap2007            TAVGLICALAGGFAKADIEMAGPMAAVGLLIVSYVVSGKSVDMYIERAGDITWEKDAEVT    1440
CPC0740_1          TAVGLICALAGGFAKADIEMAGPMAAVGLLIVSYVVSGKSVDMYIERAGDITWEKDAEIT    1440
SV0127/14_1        TAVGLICALAGGFAKADIEMAGPMAAVGLLIVSYVVSGKSVDMYIERAGDITWEKDAEVT    1440
FSS13025_1         TAVGLICALAGGFAKADIEMAGPMAAVGLLIVSYVVSGKSVDMYIERAGDITWEKDAEVT    1440
8375_1             TAVGLICALAGGFAKADIEMAGPMAAVGLLIVSYVVSGKSVDMYIERAGDITWEKDAEVT    1440
103344_1           TAVGLICALAGGFAKADIEMAGPMAAVGLLIVSYVVSGKSVDMYIERAGDITWEKDAEVT    1440
GD01_1             TAVGLICALAGGFAKADIEMAGPMAAVGLLIVSYVVSGKSVDMYIERAGDITWEKDAEVT    1440
SPH2015_1          TAVGLICALAGGFAKADIEMAGPMAAVGLLIVSYVVSGKSVDMYIERAGDITWEKDAEVT    1440
Haiti2014_1        TAVGLICALAGGFAKADIEMAGPMAAVGLLIVSYVVSGKSVDMYIERAGDITWEKDAEVT    1440
PLCal_ZV_1         TAVGLICALAGGFAKADIEMAGPMAAVGLLIVSYVVSGKSVDMYIERAGDITWEKDAEVT    1403
Martinique         TAVGLICALAGGFAKADIEMAGPMAAVGLLIVSYVVSGKSVDMYIERAGDITWEKDAEVT    1440
NatalRGN           TAVGLICALAGGFAKADIEMAGPMAAVGLLIVSYVVSGKSVDMYIERAGDITWEKDAEVT    1440
BrasilZKV2015_1    TAVGLICALAGGFAKADIEMAGPMAAVGLLIVSYVVSGKSVDMYIERAGDITWEKDAEVT    1440
Z1106033_1         TAVGLICALAGGFAKADIEMAGPMAAVGLLIVSYVVSGKSVDMYIERAGDITWEKDAEVT    1440
PRVABC59_1         TAVGLICALAGGFAKADIEMAGPMAAVGLLIVSYVVSGKSVDMYIERAGDITWEKDAEVT    1440
HPF2013_1          TAVGLICALAGGFAKADIEMAGPMAAVGLLIVSYVVSGKSVDMYIERAGDITWEKDAEVT    1440
Beh815744_1        TAVGLICALAGGFAKADIEMAGPMAAVGLLIVSYVVSGKSVDMYIERAGDITWEKDAEVT    1440
BEH818995_1        TAVGLICALAGGFAKADIEMAGPMAAVGLLIVSYVVSGKSVDMYIERAGDITWEKDAEVT    1440
BEH819966_1        TAVGLICALAGGFAKADIEMAGPMAAVGLLIVSYVVSGKSVDMYIERAGDITWEKDAEVT    1440
BeH819015_1        TAVGLICALAGGFAKADIEMAGPMAAVGLLIVSYVVSGKSVDMYIERAGDITWEKDAEVT    1440
SSABr_1            TAVGLICALAGGFAKADIEMAGPMAAVGLLIVSYVVSGKSVDMYIERAGDITWEKDAEVT    1440
ARD157995_1        TAVGLICALAGGFAKADIEMAGPMAAVGLLIVSYVVSGKSVDMYIERAGDITWEKDAEVT    1440
MR_766_1           TAVGLICALAGGFAKADIEMAGPMAAVGLLIVSYVVSGKSVDMYIERAGDITWEKDAEVT    1436
ARD158084_1        TAVGLICALAGGFAKADIEMAGPMAAVGLLIVSYVVSGKSVDMYIERAGDITWEKDAEVT    1440
ArB1362_1          TAVGLICALAGGFAKADIEMAGPMAAVGLLIVSYVVSGKSVDMYIERAGDITWEKDAEVT    1440
ARB15076_1         TAVGLICALAGGFAKADIEMAGPMAAVGLLIVSYVVSGKSVDMYIERAGDITWEKDAEVT    1434
ARB13565_1         TAVGLICALAGGFAKADIEMAGPMAAVGLLIVSYVVSGKSVDMYIERAGDITWEKDAEVT    1440
ARB7701_1          TAVGLICALAGGFAKADIEMAGPMAAVGLLIVSYVVSGKSVDMYIERAGDITWEKDAEVT    1440
IbH30656_1         TAVGLICALAGGFAKADIEMAGPMAAVGLLIVSYVVTGKSVDMYIERAGDITWEKDAEVT    1434
ArD128000_1        TAVGLICALAGGFAKADIEMAGPMAAVGLLIVSYVVSGKSVDMYIERAGDITWEKDAEVT    1440
ArD7117_1          TAVGLICALAGGFAKADIEMAGPMAAVGLLIVSYVVSGKSVDMYIERAGDITWEKDAEVT    1440
ARD_41519_1        TAVGLICALAGGFAKADIEMAGPMAAVGLLIVSYVVSGKSVDMYIERAGDITWEKDAEVT    1440
                   ************************************:*********************:*

P6-740_1           GNSPRLDVALDESGDFSLVEDDGPPMREIILKVVLMTICGMNPIAIPFAAGAWYVYVKTG    1500
P6740              GNSPRLDVALDESGDFSLVEDDGPPMREIILKVVLMTICGMNPIAIPFAAGAWYVYVKTG    1500
Yap2007            GNSPRLDVALDESGDFSLVEDDGPPMREIILKVVLMTICGMNPIAIPFAAGAWYVYVKTG    1500
CPC0740_1          GNSPRLDVALDESGDFSLVEDDGPPMREIILKVVLMTICGMNPIAIPFAAGAWYVYVKTG    1500
SV0127/14_1        GNSPRLDVALDESGDFSLVEDDGPPMREIILKVVLMTICGMNPIAIPFAAGAWYVYVKTG    1500
FSS13025_1         GNSPRLDVALDESGDFSLVEDDGPPMREIILKVVLMTICGMNPIAIPFAAGAWYVYVKTG    1500
8375_1             GNSPRLDVALDESGDFSLVEDDGPPMREIILKVVLMTICGMNPIAIPFAAGAWYVYVKTG    1500
103344_1           GNSPRLDVALDESGDFSLVEDDGPPMREIILKVVLMTICGMNPIAIPFAAGAWYVYVKTG    1500
GD01_1             GNSPRLDVALDESGDFSLVEDDGPPMREIILKVVLMTICGMNPIAIPFAAGAWYVYVKTG    1500
SPH2015_1          GNSPRLDVALDESGDFSLVEDDGPPMREIILKVVLMTICGMNPIAIPFAAGAWYVYVKTG    1500
Haiti2014_1        GNSPRLDVALDESGDFSLVEDDGPPMREIILKVVLMTICGMNPIAIPFAAGAWYVYVKTG    1500
PLCal_ZV_1         GNSPRLDVALDESGDFSLVEDDGPPMREIILKVVLMTICGMNPIAIPFAAGAWYVYVKTG    1463
Martinique         GNSPRLDVALDESGDFSLVEDDGPPMREIILKVVLMTICGMNPIAIPFAAGAWYVYVKTG    1500
NatalRGN           GNSPRLDVALDESGDFSLVEDDGPPMREIILKVVLMTICGMNPIAIPFAAGAWYVYVKTG    1500
BrasilZKV2015_1    GNSPRLDVALDESGDFSLVEDDGPPMREIILKVVLMTICGMNPIAIPFAAGAWYVYVKTG    1500
```

```
Z1106033_1          GNSPRLDVALDESGDFSLVEDDGPPMREIILKVVLMTICGMNPIAIPFAAGAWYVYVKTG     1500
PRVABC59_1          GNSPRLDVALDESGDFSLVEDDGPPMREIILKVVLMTICGMNPIAIPFAAGAWYVYVKTG     1500
HPF2013_1           GNSPRLDVALDESGDFSLVEDDGPPMREIILKVVLMTICGMNPIAIPFAAGAWYVYVKTG     1500
Beh815744_1         GNSPRLDVALDESGDFSLVEDDGPPMREIILKVVLMTICGMNPIAIPFAAGAWYVYVKTG     1500
BEH818995_1         GNSPRLDVALDESGDFSLVEDDGPPMREIILKVVLMTICGMNPIAIPFAAGAWYVYVKTG     1500
BEH819966_1         GNSPRLDVALDESGDFSLVEDDGPPMREIILKVVLMTICGMNPIAIPFAAGAWYVYVKTG     1500
BeH819015_1         GNSPRLDVALDESGDFSLVEDDGPPMREIILKVVLMTICGMNPIAIPFAAGAWYVYVKTG     1500
SSABr_1             GNSPRLDVALDESGDFSLVEDDGPPMREIILKVVLMTICGMNPIAIPFAAGAWYVYVKTG     1500
ARD157995_1         GNSPRLDVALDESGDFSLVEEDGPPMREIILKVVLMAICGMNPIAIPFAAGAWYVYVKTG     1500
MR_766_1            GNSPRLDVALDESGDFSLVEEDGPPMREIILKVVLMAICGMNPIAIPFAAGAWYVYVKTG     1496
ARD158084_1         GNSPRLDVALDESGDFSLVEEDGPPMREIILKVVLMAICGMNPIAIPFAAGAWYVYVKTG     1500
ArB1362_1           GNSPRLDVALDESGDFSLVEEDGPPMREIILKVVLMAICGMNPIAIPFAAGAWYVYVKTG     1500
ARB15076_1          GNSPRLDVALDESGDFSLVEEDGPPMREIILKVVLMAICGMNPIAIPFAAGAWYVYVKTG     1494
ARB13565_1          GNSPRLDVALDESGDFSLVEEDGPPMREIILKVVLMAICGMNPIAIPFAAGAWYVYVKTG     1500
ARB7701_1           GNSPRLDVALDESGDFSLVEEDGPPMREIILKVVLMAICGMNPIAIPFAAGAWYVYVKTG     1500
IbH30656_1          GNSPRLDVALDESGDFSLVEEDGPPMREIILKVVLMAICGMNPIAIPFAAGAWYVYVKTG     1494
ArD128000_1         GNSPRLDVALDESGDFSLVEEDGPPMREIILKVVLMAICGMNPIAIPFAAGAWYVYVKTG     1500
ArD7117_1           GNSPRLDVALDESGDFSLVEEDGPPMREIILKVVLMAICGMNPIAIPFAAGAWYVYVKTG     1500
ARD_41519_1         GNSPRLDVALDESGDFSLVEEDGPPMREIILKVVLMAICGMNPIAIPFAAGAWYVYVKTG     1500
                    ********************:***************:***********************

P6-740_1            KRSGALWDVPAPKEVKKGETTDGVYRVMTRRLLGSTQVGVGVMQEGVFHTMWHVTKGSAL     1560
P6740               KRSGALWDVPAPKEVKKGETTDGVYRVMTRRLLGSTQVGVGVMQEGVFHTMWHVTKGSAL     1560
Yap2007             KRSGALWDVPAPKEVKKGETTDGVYRVMTRRLLGSTQVGVGVMQEGVFHTMWHVTKGSAL     1560
CPC0740_1           KRSGALWDVPAPKEVKKGETTDGVYRVMTRRLLGSTQVGVGVMQEGVFHTMWHVTKGSAL     1560
SV0127/14_1         KRSGALWDVPAPKEVKKGETTDGVYRVMTRRLLGSTQVGVGVMQEGVFHTMWHVTKGSAL     1560
FSS13025_1          KRSGALWDVPAPKEVKKGETTDGVYRVMTRRLLGSTQVGVGVMQEGVFHTMWHVTKGSAL     1560
8375_1              KRSGALWDVPAPKEVKKGETTDGVYRVMTRRLLGSTQVGVGVMQEGVFHTMWHVTKGSAL     1560
103344_1            KRSGALWDVPAPKEVKKGETTDGVYRVMTRRLLGSTQVGVGVMQEGVFHTMWHVTKGSAL     1560
GD01_1              KRSGALWDVPAPKEVKKGETTDGVYRVMTRRLLGSTQVGVGVMQEGVFHTMWHVTKGSAL     1560
SPH2015_1           KRSGALWDVPAPKEVKKGETTDGVYRVMTRRLLGSTQVGVGVMQEGVFHTMWHVTKGSAL     1560
Haiti2014_1         KRSGALWDVPAPKEVKKGETTDGVYRVMTRRLLGSTQVGVGVMQEGVFHTMWHVTKGSAL     1560
PLCal_ZV_1          KRSGALWDVPAPKEVKKGETTDGVYRVMTRRLLGSTQVGVGVMQEGVFHTMWHVTKGSAL     1523
Martinique          KRSGALWDVPAPKEVKKGETTDGVYRVMTRRLLGSTQVGVGVMQEGVFHTMWHVTKGSAL     1560
NatalRGN            KRSGALWDVPAPKEVKKGETTDGVYRVMTRRLLGSTQVGVGVMQEGVFHTMWHVTKGSAL     1560
BrasilZKV2015_1     KRSGALWDVPAPKEVKKGETTDGVYRVMTRRLLGSTQVGVGVMQEGVFHTMWHVTKGSAL     1560
Z1106033_1          KRSGALWDVPAPKEVKKGETTDGVYRVMTRRLLGSTQVGVGVMQEGVFHTMWHVTKGSAL     1560
PRVABC59_1          KRSGALWDVPAPKEVKKGETTDGVYRVMTRRLLGSTQVGVGVMQEGVFHTMWHVTKGSAL     1560
HPF2013_1           KRSGALWDVPAPKEVKKGETTDGVYRVMTRRLLGSTQVGVGVMQEGVFHTMWHVTKGSAL     1560
Beh815744_1         KRSGALWDVPAPKEVKKGETTDGVYRVMTRRLLGSTQVGVGVMQEGVFHTMWHVTKGSAL     1560
BEH818995_1         KRSGALWDVPAPKEVKKGETTDGVYRVMTRRLLGSTQVGVGVMQEGVFHTMWHVTKGSAL     1560
BEH819966_1         KRSGALWDVPAPKEVKKGETTDGVYRVMTRRLLGSTQVGVGVMQEGVFHTMWHVTKGSAL     1560
BeH819015_1         KRSGALWDVPAPKEVKKGETTDGVYRVMTRRLLGSTQVGVGVMQEGVFHTMWHVTKGSAL     1560
SSABr_1             KRSGALWDVPAPKEVKKGETTDGVYRVMTRRLLGSTQVGVGVMQEGVFHTMWHVTKGSAL     1560
ARD157995_1         KRSGALWDVPAPKEVKKGETTDGVYRVMTRRLLGSTQVGVGVMQEGVFHTMWHVTKGAAL     1560
MR_766_1            KRSGALWDVPAPKEVKKGETTDGVYRVMTRRLLGSTQVGVGVMQEGVFHTMWHVTKGAAL     1556
ARD158084_1         KRSGALWDVPAPKEVKKGETTDGVYRVMTRRLLGSTQVGVGVMQEGVFHTMWHVTKGAAL     1560
ArB1362_1           KRSGALWDVPAPKEVKKGETTDGVYRVMTRRLLGSTQVGVGVMQEGVFHTMWHVTKGAAL     1560
ARB15076_1          KRSGALWDVPAPKEVKKGETTDGVYRVMTRRLLGSTQVGVGVMQEGVFHTMWHVTKGAAL     1554
ARB13565_1          KRSGALWDVPAPKEVKKGETTDGVYRVMTRRLLGSTQVGVGVMQEGVFHTMWHVTKGAAL     1560
ARB7701_1           KRSGALWDVPAPKEVKKGETTDGVYRVMTRRLLGSTQVGVGVMQEGVFHTMWHVTKGAAL     1560
IbH30656_1          KRSGALWDVPAPKEVKKGETTDGVYRVMTRRLLGSTQVGVGVMQEGVFHTMWHVTKGAAL     1554
ArD128000_1         KRSGALWDVPAPKEVKKGETTDGVYRVMTRRLLGSTQVGVGVMQEGVFHTMWHVTKGAAL     1560
ArD7117_1           KRSGALWDVPAPKEVKKGETTDGVYRVMTRRLLGSTQVGVGVMQEGVFHTMWHVTKGAAL     1560
ARD_41519_1         KRSGALWDVPAPKEVKKGETTDGVYRVMTRRLLGSTQVGVGVMQEGVFHTMWHVTKGAAL     1560
                    *********************************************************:**

P6-740_1            RSGEGRLDPYWGDVKQDLVSYCGPWKLDAAWDGHSEVQLLAVPPGERARNIQTLPGIFKT     1620
P6740               RSGEGRLDPYWGDVKQDLVSYCGPWKLDAAWDGHSEVQLLAVPPGERARNIQTLPGIFKT     1620
Yap2007             RSGEGRLDPYWGDVKQDLVSYCGPWKLDAAWDGHSEVQLLAVPPGERARNIQTPPGIFKT     1620
CPC0740_1           RSGEGRLDPYWGDVKQDLVSYCGPWKLDAAWDGHSEVQLLAVPPGERARNIQTLPGTFKT     1620
SV0127/14_1         RSGEGRLDPYWGDVKQDLVSYCGPWKLDAAWDGHSEVQLLAVPPGERARNIQTLPGIFKT     1620
FSS13025_1          RSGEGRLDPYWGDVKQDLVSYCGPWKLDAAWDGHSEVQLLAVPPGERARNIQTLPGIFKT     1620
8375_1              RSGEGRLDPYWGDVKQDLVSYCGPWKLDAAWDGHSEVQLLAVPPGERARNIQTLPGIFKT     1620
103344_1            RSGEGRLDPYWGDVKQDLVSYCGPWKLDAAWDGHSEVQLLAVPPGERARNIQTLPGIFKT     1620
GD01_1              RSGEGRLDPYWGDVKQDLVSYCGPWKLDAAWDGHSEVQLLAVPPGERARNIQTLPGIFKT     1620
SPH2015_1           RSGEGRLDPYWGDVKQDLVSYCGPWKLDAAWDGHSEVQLLAVPPGERARNIQTLPGIFKT     1620
Haiti2014_1         RSGEGRLDPYWGDVKQDLVSYCGPWKLDAAWDGHSEVQLLAVPPGERARNIQTLPGIFKT     1620
```

```
PLCal_ZV_1          RSGEGRLDPYWGDVKQDLVSYCGPWKLDAAWDGHSEVQLLAVPPGERARNIQTLPGIFKT     1583
Martinique          RSGEGRLDPYWGDVKQDLVSYCGPWKLDAAWDGHSEVQLLAVPPGERARNIQTLPGIFKT     1620
NatalRGN            RSGEGRLDPYWGDVKQDLVSYCGPWKLDAAWDGHSEVQLLAVPPGERARNIQTLPGIFKT     1620
BrasilZKV2015_1     RSGEGRLDPYWGDVKQDLVSYCGPWKLDAAWDGHSEVQLLAVPPGERARNIQTLPGIFKT     1620
Z1106033_1          RSGEGRLDPYWGDVKQDLVSYCGPWKLDAAWDGHSEVQLLAVPPGERARNIQTLPGIFKT     1620
PRVABC59_1          RSGEGRLDPYWGDVKQDLVSYCGPWKLDAAWDGHSEVQLLAVPPGERARNIQTLPGIFKT     1620
HPF2013_1           RSGEGRLDPYWGDVKQDLVSYCGPWKLDAAWDGHSEVQLLAVPPGERARNIQTLPGIFKT     1620
Beh815744_1         RSGEGRLDPYWGDVKQDLVSYCGPWKLDAAWDGHSEVQLLAVPPGERARNIQTLPGIFKT     1620
BEH818995_1         RSGEGRLDPYWGDVKQDLVSYCGPWKLDAAWDGHSEVQLLAVPPGERARNIQTLPGIFKT     1620
BEH819966_1         RSGEGRLDPYWGDVKQDLVSYCGPWKLDAAWDGHSEVQLLAVPPGERARNIQTLPGIFKT     1620
BeH819015_1         RSGEGRLDPYWGDVKQDLVSYCGPWKLDAAWDGHSEVQLLAVPPGERARNIQTLPGIFKT     1620
SSABr_1             RSGEGRLDPYWGDVKQDLVSYCGPWKLDAAWDGHSEVQLLAVPPGERARNIQTLPGIFKT     1620
ARD157995_1         RSGEGRLDPYWGDVKQDLVSYCGPWKLDAAWDGLSEVQLLAVPPGERARNIQTLPGIFKT     1620
MR_766_1            RSGEGRLDPYWGDVKQDLVSYCGPWKLDAAWDGLSEVQLLAVPPGERARNIQTLPGIFKT     1616
ARD158084_1         RSGEGRLDPYWGDVKQDLVSYCGPWKLDAAWDGLSEVQLLAVPPGERARNIQTLPGIFKT     1620
ArB1362_1           RSGEGRLDPYWGDVKQDLVSYCGPWKLDAAWDGLSEVQLLAVPPGERARNIQTLPGIFKT     1620
ARB15076_1          RSGEGRLDPYWGDVKQDLVSYCGPWKLDAAWDGLSEVQLLAVPPGERARNIQTLPGIFKT     1614
ARB13565_1          RSGEGRLDPYWGDVKQDLVSYCGPWKLDAAWDGLSEVQLLAVPPGERARNIQTLPGIFKT     1620
ARB7701_1           RSGEGRLDPYWGDVKQDLVSYCGPWKLDAAWDGLSEVQLLAVPPGERARNIQTLPGIFKT     1620
IbH30656_1          RSGEGRLDPYWGDVKQDLVSYCGPWKLDAAWDGLSEVQLLAVPPGERAKNIQTLPGIFKT     1614
ArD128000_1         RSGEGRLDPYWGDVKQDLVSYCGPWKLDATWDGLSEVQLLAVPPGERARNIQTLPGIFKT     1620
ArD7117_1           RSGEGRLDPYWGDVKQDLVSYCGPWKLDAAWDGLSEVQLLAVPPGERARNIQTLPGIFKT     1620
ARD_41519_1         RSGEGRLDPYWGDVKQDLVSYCGPWKLDAAWDGLSEVQLLAVPPGERARNIQTLPGIFKT     1620
                    *****************************:*** **************:**** ** ***

P6-740_1            KDGDIGAVALDYPAGTSGSPILDKCGRVIGLYGNGVVIKNGSYVSAITQGRREEETPVEC     1680
P6740               KDGDIGAVALDYPAGTSGSPILDKCGRVIGLYGNGVVIKNGSYVSAITQGRREEETPVEC     1680
Yap2007             KDGDIGAVALDYPAGTSGSPILDKCGRVIGLYGNGVVIKNGSYVSAITQGRREEETPVEC     1680
CPC0740_1           KDGDIGAVALDYPAGTSGSPILDKCGRVIGLYGNGVVIKNGSYVSAITQGRREEETPVEC     1680
SV0127/14_1         KDGDIGAVALDYPAGTSGSPILDKCGRVIGLYGNGVVIKNGSYVSAITQGRREEETPVEC     1680
FSS13025_1          KDGDIGAVALDYPAGTSGSPILDKCGRVIGLYGNGVVIKNGSYVSAITQGRREEETPVEC     1680
8375_1              KDGDIGAVALDYPAGTSGSPILDKCGRVIGLYGNGVVIKNGSYVSAITQGRREEETPVEC     1680
103344_1            KDGDIGAVALDYPAGTSGSPILDKCGRVIGLYGNGVVIKNGSYVSAITQGRREEETPVEC     1680
GD01_1              KDGDIGAVALDYPAGTSGSPILDKCGRVIGLYGNGVVIKNGSYVSAITQGRREEETPVEC     1680
SPH2015_1           KDGDIGAVALDYPAGTSGSPILDKCGRVIGLYGNGVVIKNGSYVSAITQGRREEETPVEC     1680
Haiti2014_1         KDGDIGAVALDYPAGTSGSPILDKCGRVIGLYGNGVVIKNGSYVSAITQGRREEETPVEC     1680
PLCal_ZV_1          KDGDIGAVALDYPAGTSGSPILDKCGRVIGLYGNGVVIKNGSYVSAITQGRREEETPVEC     1643
Martinique          KDGDIGAVALDYPAGTSGSPILDKCGRVIGLYGNGVVIKNGSYVSAITQGRREEETPVEC     1680
NatalRGN            KDGDIGAVALDYPAGTSGSPILDKCGRVIGLYGNGVVIKNGSYVSAITQGRREEETPVEC     1680
BrasilZKV2015_1     KDGDIGAVALDYPAGTSGSPILDKCGRVIGLYGNGVVIKNGSYVSAITQGRREEETPVEC     1680
Z1106033_1          KDGDIGAVALDYPAGTSGSPILDKCGRVIGLYGNGVVIKNGSYVSAITQGRREEETPVEC     1680
PRVABC59_1          KDGDIGAVALDYPAGTSGSPILDKCGRVIGLYGNGVVIKNGSYVSAITQGRREEETPVEC     1680
HPF2013_1           KDGDIGAVALDYPAGTSGSPILDKCGRVIGLYGNGVVIKNGSYVSAITQGRREEETPVEC     1680
Beh815744_1         KDGDIGAVALDYPAGTSGSPILDKCGRVIGLYGNGVVIKNGSYVSAITQGRREEETPVEC     1680
BEH818995_1         KDGDIGAVALDYPAGTSGSPILDKCGRVIGLYGNGVVIKNGSYVSAITQGRREEETPVEC     1680
BEH819966_1         KDGDIGAVALDYPAGTSGSPILDKCGRVIGLYGNGVVIKNGSYVSAITQGRREEETPVEC     1680
BeH819015_1         KDGDIGAVALDYPAGTSGSPILDKCGRVIGLYGNGVVIKNGSYVSAITQGRREEETPVEC     1680
SSABr_1             KDGDIGAVALDYPAGTSGSPILDKCGRVIGLYGNGVVIKNGSYVSAITQGRREEETPVEC     1680
ARD157995_1         KDGDIGAVALDYPAGTSGSPILDKCGRVIGLYGNGVVIKNGSYVSAITQGKREEETPVEC     1680
MR_766_1            KDGDIGAVALDYPAGTSGSPILDKCGRVIGLYGNGVVIKNGSYVSAITQGKREEETPVEC     1676
ARD158084_1         KDGDIGAVALDYPAGTSGSPILDKCGRVIGLYGNGVVIKNGSYVSAITQGKREEETPVEC     1680
ArB1362_1           KDGDIGAVALDYPAGTSGSPILDKCGRVIGLYGNGVVIKNGSYVSAITQGKREEETPVEC     1680
ARB15076_1          KDGDIGAVALDYPAGTSGSPILDKCGRVIGLYGNGVVIKNGSYVSAITQGKREEETPVEC     1674
ARB13565_1          KDGDIGAVALDYPAGTSGSPILDKCGRVIGLYGNGVVIKNGSYVSAITQGKREEETPVEC     1680
ARB7701_1           KDGDIGAVALDYPAGTSGSPILDKCGRVIGLYGNGVVIKNGSYVSAITQGKREEETPVEC     1680
IbH30656_1          KDGDIGAVALDYPAGTSGSPILDKCGRVIGLYGNGVVIKNGSYVSAITQGKREEETPVEC     1674
ArD128000_1         KDGDIGAVALDYPAGTSGSPILDKCGRVIGLYGNGVVIKNGSYVSAITQGKREEEAPVEC     1680
ArD7117_1           KDGDIGAVALDYPAGTSGSPILDKCGRVIGLYGNGVVIKNGSYVSAITQGKREEEAPVEC     1680
ARD_41519_1         KDGDIGAVALDYPAGTSGSPILDKCGRVIGLYGNGVVIKNGSYVSAITQGKREEEAPVEC     1680
                    **************************************************:****:****

P6-740_1            FEPSMLKKKQLTVLDLHPGAGKTRRVLPEIVREAIKTRLRTVILAPTRVVAAEMEEALRG     1740
P6740               FEPSMLKKKQLTVLDLHPGAGKTRRVLPEIVREAIKTRLRTVILAPTRVVAAEMEEALRG     1740
Yap2007             FEPSMLKKKQLTVLDLHPGAGKTRRVLPEIVREAIKTRLRTVILAPTRVVAAEMEEALRG     1740
CPC0740_1           FEPSMLKKKQLTVLDLHPGAGKTRRVLPEIVREAIKTRLRTVILAPTRVVAAEMEEALRG     1740
SV0127/14_1         FEPSMLKKKQLTVLDLHPGAGKTRRVLPEIVREAIKTRLRTVILAPTRVVAAEMEEALRG     1740
FSS13025_1          FEPSMLKKKQLTVLDLHPGAGKTRRVLPEIVREAIKTRLRTVILAPTRVVAAEMEEALRG     1740
8375_1              FEPSMLKKKQLTVLDLHPGAGKTRRVLPEIVREAIKTRLRTVILAPTRVVAAEMEEALRG     1740
```

```
103344_1             FEPSMLKKKQLTVLDLHPGAGKTRRVLPEIVREAIKTRLRTVILAPTRVVAAEMEEALRG    1740
GD01_1               FEPSMLKKKQLTVLDLHPGAGKTRRVLPEIVREAIKTRLRTVILAPTRVVAAEMEEALRG    1740
SPH2015_1            FEPSMLKKKQLTVLDLHPGAGKTRRVLPEIVREAIKTRLRTVILAPTRVVAAEMEEALRG    1740
Haiti2014_1          FEPSMLKKKQLTVLDLHPGAGKTRRVLPEIVREAIKTRLRTVILAPTRVVAAEMEEALRG    1740
PLCal_ZV_1           FEPSMLKKKQLTVLDLHPGAGKTRRVLPEIVREAIKTRLRTVILAPTRVVAAEMEEALRG    1703
Martinique           FEPSMLKKKQLTVLDLHPGAGKTRRVLPEIVREAIKTRLRTVILAPTRVVAAEMEEALRG    1740
NatalRGN             FEPSMLKKKQLTVLDLHPGAGKTRRVLPEIVREAIKTRLRTVILAPTRVVAAEMEEALRG    1740
BrasilZKV2015_1      FEPSMLKKKQLTVLDLHPGAGKTRRVLPEIVREAIKTRLRTVILAPTRVVAAEMEEALRG    1740
Z1106033_1           FEPSMLKKKQLTVLDLHPGAGKTRRVLPEIVREAIKTRLRTVILAPTRVVAAEMEEALRG    1740
PRVABC59_1           FEPSMLKKKQLTVLDLHPGAGKTRRVLPEIVREAIKTRLRTVILAPTRVVAAEMEEALRG    1740
HPF2013_1            FEPSMLKKKQLTVLDLHPGAGKTRRVLPEIVREAIKTRLRTVILAPTRVVAAEMEEALRG    1740
Beh815744_1          FEPSMLKKKQLTVLDLHPGAGKTRRVLPEIVREAIKTRLRTVILAPTRVVAAEMEEALRG    1740
BEH818995_1          FEPSMLKKKQLTVLDLHPGAGKTRRVLPEIVREAIKTRLRTVILAPTRVVAAEMEEALRG    1740
BEH819966_1          FEPSMLKKKQLTVLDLHPGAGKTRRVLPEIVREAIKTRLRTVILAPTRVVAAEMEEALRG    1740
BeH819015_1          FEPSMLKKKQLTVLDLHPGAGKTRRVLPEIVREAIKTRLRTVILAPTRVVAAEMEEALRG    1740
SSABr_1              FEPSMLKKKQLTVLDLHPGAGKTRRVLPEIVREAIKTRLRTVILAPTRVVAAEMEEALRG    1740
ARD157995_1          FEPSMLKKKQLTVLDLHPGAGKTRRVLPEIVREAIKKRLRTVILAPTPVERSYSRWSLWP    1740
MR_766_1             FEPSMLKKKQLTVLDLHPGAGKTRRVLPEIVREAIKKRLRTVILAPTRVVAAEMEEALRG    1736
ARD158084_1          FEPSMLKKKQLTVLDLHPGAGKTRRVLPEIVREAIKKRLRTVILAPTRVVAAEMEEALRG    1740
ArB1362_1            FEPSMLKKKQLTVLDLHPGAGKTRRVLPEIVREAIKKRLRTVILAPTRVVAAEMEEALRG    1740
ARB15076_1           FEPSMLKKKQLTVLDLHPGAGKTRRVLPEIVREAIKKRLRTVILAPTRVVAAEMEEALRG    1734
ARB13565_1           FEPSMLKKKQLTVLDLHPGAGKTRRVLPEIVREAIKKRLRTVILAPTRVVAAEMEEALRG    1740
ARB7701_1            FEPSMLKKKQLTVLDLHPGAGKTRRVLPEIVREAIKKRLRTVILAPTRVVAAEMEEALRG    1740
IbH30656_1           FEPSMLRKKQLTVLDLHPGAGKTRRVLPEIVREAIKKRLRTVILAPTRVVAAEMEEALRG    1734
ArD128000_1          FEPSMLRKKQLTVLDLHPGAGKTRRVLPEIVREAIKKRLRTVILAPTRVVAAEMEEALRG    1740
ArD7117_1            FEPSMLRKKQLTVLDLHPGAGKTRRVLPEIVREAIKKRLRTLILAPTRVVAAEMEEALRG    1740
ARD_41519_1          FEPSMLRKKQLTVLDLHPGAGKTRRVLPEIVREAIKKRLRTVILAPTRVVAAEMEEALRG    1740
                     ******:*****************************.****:***** *  :  . :*

P6-740_1             LPVRYMTTAVNVTHSGTEIVDLMCHATFTSRLLQPIRVPNYNLYIMDEAHFTDPSSIAAR    1800
P6740                LPVRYMTTAVNVTHSGTEIVDLMCHATFTSRLLQPIRVPNYNLYIMDEAHFTDPSSIAAR    1800
Yap2007              LPVRYMTTAVNVTHSGTEIVDLMCHATFTSRLLQPIRVPNYNLYIMDEAHFTDPSSIAAR    1800
CPC0740_1            LPVRYMTTAVNVTHSGTEIVDLMCHATFTSRLLQPIRVPNYNLYIMDEAHFTDPSSIAAR    1800
SV0127/14_1          LPVRYMTTAVNVTHSGTEIVDLMCHATFTSRLLQPIRVPNYNLYIMDEAHFTDPSSIAAR    1800
FSS13025_1           LPVRYMTTAVNVTHSGTEIVDLMCHATFTSRLLQPIRVPNYNLYIMDEAHFTDPSSIAAR    1800
8375_1               LPVRYMTTAVNVTHSGTEIVDLMCHATFTSRLLQPIRVPNYNLYIMDEAHFTDPSSIAAR    1800
103344_1             LPVRYMTTAVNVTHSGTEIVDLMCHATFTSRLLQPIRVPNYNLYIMDEAHFTDPSSIAAR    1800
GD01_1               LPVRYMTTAVNVTHSGTEIVDLMCHATFTSRLLQPIRVPNYNLYIMDEAHFTDPSSIAAR    1800
SPH2015_1            LPVRYMTTAVNVTHSGTEIVDLMCHATFTSRLLQPIRVPNYNLYIMDEAHFTDPSSIAAR    1800
Haiti2014_1          LPVRYMTTAVNVTHSGTEIVDLMCHATFTSRLLQPIRVPNYNLYIMDEAHFTDPSSIAAR    1800
PLCal_ZV_1           LPVRYMTTAVNVTHSGTEIVDLMCHATFTSRLLQPIRVPNYNLYIMDEAHFTDPSSIAAR    1763
Martinique           LPVRYMTTAVNVTHSGTEIVDLMCHATFTSRLLQPIRVPNYNLYIMDEAHFTDPSSIAAR    1800
NatalRGN             LPVRYMTTAVNVTHSGTEIVDLMCHATFTSRLLQPIRVPNYNLYIMDEAHFTDPSSIAAR    1800
BrasilZKV2015_1      LPVRYMTTAVNVTHSGTEIVDLMCHATFTSRLLQPIRVPNYNLYIMDEAHFTDPSSIAAR    1800
Z1106033_1           LPVRYMTTAVNVTHSGTEIVDLMCHATFTSRLLQPIRVPNYNLYIMDEAHFTDPSSIAAR    1800
PRVABC59_1           LPVRYMTTAVNVTHSGTEIVDLMCHATFTSRLLQPIRVPNYNLYIMDEAHFTDPSSIAAR    1800
HPF2013_1            LPVRYMTTAVNVTHSGTEIVDLMCHATFTSRLLQPIRVPNYNLYIMDEAHFTDPSSIAAR    1800
Beh815744_1          LPVRYMTTAVNVTHSGTEIVDLMCHATFTSRLLQPIRVPNYNLYIMDEAHFTDPSSIAAR    1800
BEH818995_1          LPVRYMTTAVNVTHSGTEIVDLMCHATFTSRLLQPIRVPNYNLYIMDEAHFTDPSSIAAR    1800
BEH819966_1          LPVRYMTTAVNVTHSGTEIVDLMCHATFTSRLLQPIRVPNYNLYIMDEAHFTDPSSIAAR    1800
BeH819015_1          LPVRYMTTAVNVTHSGTEIVDLMCHATFTSRLLQPIRVPNYNLYIMDEAHFTDPSSIAAR    1800
SSABr_1              LPVRYMTTAVNVTHSGTEIVDLMCHATFTSRLLQPIRVPNYNLYIMDEAHFTDPSSIAAR    1800
ARD157995_1          SVARTQTTAVNVTHSGTEIVDLMCHATFTSRLLQPIRVPNYNLNIMDEAHFTDPSSIAAR    1800
MR_766_1             LPVRYMTTAVNVTHSGTEIVDLMCHATFTSRLLQPIRVPNYNLNIMDEAHFTDPSSIAAR    1796
ARD158084_1          LPVRYMTTAVNVTHSGTEIVDLMCHATFTSRLLQPIRVPNYNLNIMDEAHFTDPSSIAAR    1800
ArB1362_1            LPVRYMTTAVNVTHSGTEIVDLMCHATFTSRLLQPIRVPNYNLNIMDEAHFTDPSSIAAR    1800
ARB15076_1           LPVRYMTTAVNVTHSGTEIVDLMCHATFTSRLLQPIRVPNYNLYIMDEAHFTDPSSIAAR    1794
ARB13565_1           LPVRYMTTAVNVTHSGTEIVDLMCHATFTSRLLQPIRVPNYNLYIMDEAHFTDPSSIAAR    1800
ARB7701_1            LPVRYMTTAVNVTHSGTEIVDLMCHATFTSRLLQPIRVPNYNLYIMDEAHFTDPSSIAAR    1800
IbH30656_1           LPVRYMTTAVNVTHSGTEIVDLMCHATFTSRLLQPIRVPNYNLYIMDEAHFTDPSSIAAR    1794
ArD128000_1          LPVRYMTTAVNVTHSGTEIVDLMCHATFTSRLLQPIRVPNYNLNIMDEAHFTDPSSIAAR    1800
ArD7117_1            LQVRYMTTAVNVTHSGTEIVDLMCHATFTSRLLQPIRVPNYNLNIMDEAHFTDPSSIAAR    1800
ARD_41519_1          LPVRYMTTAVNVTHSGTEIVDLMCHATFTSRLLQPIRVPNYNLYIMDEAHFTDPSSIAAR    1800
                       .*  ************************************* ****************

P6-740_1             GYISTRVEMGEAAAIFMTATPPGTRDAFPDSNSPIMDTEVEVPERAWSSGFDWVTDHSGK    1860
P6740                GYISTRVEMGEAAAIFMTATPPGTRDAFPDSNSPIMDTEVEVPERAWSSGFDWVTDHSGK    1860
Yap2007              GYISTRVEMGEAAAIFMTATPPGTRDAFPDSNSPIMDTEVEVPERAWSSGFDWVTDHSGK    1860
```

```
CPC0740_1           GYISTRVEMGEAAAIFMTATPPGTRDAFPDSNSPIMDTEVEVPERAWSTGFDWVTDHSGK      1860
SV0127/14_1         GYISTRVEMGEAAAIFMTATPPGTRDAFPDSNSPIMDTEVEVPERAWSSGFDWVTDHSGK      1860
FSS13025_1          GYISTRVEMGEAAAIFMTATPPGTRDAFPDSNSPIMDTEVEVPERAWSSGFDWVTDHSGK      1860
8375_1              GYISTRVEMGEAAAIFMTATPPGTRDAFPDSNSPIMDTEVEVPERAWSSGFDWVTDHSGK      1860
103344_1            GYISTRVEMGEAAAIFMTATPPGTRDAFPDSNSPIMDTEVEVPERAWSSGFDWVTDHSGK      1860
GD01_1              GYISTRVEMGEAAAIFMTATPPGTRDAFPDSNSPIMDTEVEVPERAWSSGFDWVTDHSGK      1860
SPH2015_1           GYISTRVEMGEAAAIFMTATPPGTRDAFPDSNSPIMDTEVEVPERAWSSGFDWVTDYSGK      1860
Haiti2014_1         GYISTRVEMGEAAAIFMTATPPGTRDAFPDSNSPIMDTEVEVPERAWSSGFDWVTDYSGK      1860
PLCal_ZV_1          GYISTRVEMGEAAAIFMTATPPGTRDAFPDSNSPIMDTEVEVPERAWSSGFDWVTDHSGK      1823
Martinique          GYISTRVEMGEAAAIFMTATPPGTRDAFPDSNSPIMDTEVEVPERAWSSGFDWVTDHSGK      1860
NatalRGN            GYISTRVEMGEAAAIFMTATPPGTRDAFPDSNSPIMDTEVEVPERAWSSGFDWVTDHSGK      1860
BrasilZKV2015_1     GYISTRVEMGEAAAIFMTATPPGTRDAFPDSNSPIMDTEVEVPERAWSSGFDWVTDHSGK      1860
Z1106033_1          GYISTRVEMGEAAAIFMTATPPGTRDAFPDSNSPIMDTEVEVPERAWSSGFDWVTDHSGK      1860
PRVABC59_1          GYISTRVEMGEAAAIFMTATPPGTRDAFPDSNSPIMDTEVEVPERAWSSGFDWVTDHSGK      1860
HPF2013_1           GYISTRVEMGEAAAIFMTATPPGTRDAFPDSNSPIMDTEVEVPERAWSSGFDWVTDHSGK      1860
Beh815744_1         GYISTRVEMGEAAAIFMTATPPGTRDAFPDSNSPIMDTEVEVPERAWSSGFDWVTDHSGK      1860
BEH818995_1         GYISTRVEMGEAAAIFMTATPPGTRDAFPDSNSPIMDTEVEVPERAWSSGFDWVTDHSGK      1860
BEH819966_1         GYISTRVEMGEAAAIFMTATPPGTRDAFPDSNSPIMDTEVEVPERAWSSGFDWVTDHSGK      1860
BeH819015_1         GYISTRVEMGEAAAIFMTATPPGTRDAFPDSNSPIMDTEVEVPERAWSSGFDWVTDHSGK      1860
SSABr_1             GYISTRVEMGEAAAIFMTATPPGTRDAFPDSNSPIMDTEVEVPERAWSSGFDWVTDHSGK      1860
ARD157995_1         GYISTRVEMGEAAAIFMTATPPGTRDAFPDSNSPIMDTEVEVPERAWSSGFDWVTDHSGK      1860
MR_766_1            GYISTRVEMGEAAAIFMTATPPGTRDAFPDSNSPIMDTEVEVPERAWSSGFDWVTDHSGK      1856
ARD158084_1         GYISTRVEMGEAAAIFMTATPPGTRDAFPDSNSPIMDTEVEVPERAWSSGFDWVTDHSGK      1860
ArB1362_1           GYISTRVEMGEAAAIFMTATPPGTRDAFPDSNSPIMDTEVEVPERAWSSGFDWVTDHSGK      1860
ARB15076_1          GYISTRVEMGEAAAIFMTATPPGTRDAFPDSNSPIMDTEVEVPERAWSSGFDWVTDHSGK      1854
ARB13565_1          GYISTRVEMGEAAAIFMTATPPGTRDAFPDSNSPIMDTEVEVPERAWSSGFDWVTDHSGK      1860
ARB7701_1           GYISTRVEMGEAAAIFMTATPPGTRDAFPDSNSPIMDTEVEVPERAWSSGFDWVTDHSGK      1860
IbH30656_1          GYISTRVEMGEAAAIFMTATPPGTRDAFPDSNSPIMDTEVEVPERAWSSGFDWVTDHSGK      1854
ArD128000_1         GYISTRVEMGEAAAIFMTATPPGTRDAFPDSNSPIMDTEVEVPERAWSSGFDWVTDHSGK      1860
ArD7117_1           GYISTRVEMGEAAAIFMTATPPGTRDAFPDSNSPIMDTEVEVPERAWSSGFDWVTDHSGK      1860
ARD_41519_1         GYISTRVEMGEAAAIFMTATPPGTRDAFPDSNSPIMDTEVEVPERAWSSGFDWVTDHSGK      1860
                    ************************************************:*******:***

P6-740_1            TVWFVPSVRNGNEIAACLTKAGKRVIQLSRKTFETEFQKTKNQEWDFVVTTDISEMGANF      1920
P6740               TVWFVPSVRNGNEIAACLTKAGKRVIQLSRKTFETEFQKTKNQEWDFVVTTDISEMGANF      1920
Yap2007             TVWFVPSVRNGNEIAACLTKAGKRVIQLSRKTFETEFLKTKNQEWDFVVTTDISEMGANF      1920
CPC0740_1           TVWFVPSVRNGNEIAACLTKAGKRVIQLSRKTFETEFQKTKNQEWDFVVTTDISEMGANF      1920
SV0127/14_1         TVWFVPSVRNGNEIAACLTKAGKRVIQLSRKTFETEFQKTKHQEWDFVVTTDISEMGANF      1920
FSS13025_1          TVWFVPSVRNGNEIAACLTKAGKRVIQLSRKTFETEFQKTKHQEWDFVVTTDISEMGANF      1920
8375_1              TVWFVPSVRNGNEIAACLTKAGKRVIQLSRKTFETEFQKTKHQEWDFVVTTDISEMGANF      1920
103344_1            TVWFVPSVRNGNEIAACLTKAGKRVIQLSRKTFETEFQKTKHQEWDFVVTTDISEMGANF      1920
GD01_1              TVWFVPSVRNGNEIAACLTKAGKRVIQLSRKTFETEFQKTKHQEWDFVVTTDISEMGANF      1920
SPH2015_1           TVWFVPSVRNGNEIAACLTKAGKRVIQLSRKTFETEFQKTKHQEWDFVVTTDISEMGANF      1920
Haiti2014_1         TVWFVPSVRNGNEIAACLTKAGKRVIQLSRKTFETEFQKTKHQEWDFVVTTDISEMGANF      1920
PLCal_ZV_1          TVWFVPSVRNGNEIAACLTKAGKRVIQLSRKTFETEFQKTKHQEWDFVVTTDISEMGANF      1883
Martinique          TVWFVPSVRNGNEIAACLTKAGKRVIQLSRKTFETEFQKTKHQEWDFVVTTDISEMGANF      1920
NatalRGN            TVWFVPSVRNGNEIAACLTKAGKRVIQLSRKTFETEFQKTKHQEWDFVVTTDISEMGANF      1920
BrasilZKV2015_1     TVWFVPSVRNGNEIAACLTKAGKRVIQLSRKTFETEFQKTKHQEWDFVVTTDISEMGANF      1920
Z1106033_1          TVWFVPSVRNGNEIAACLTKAGKRVIQLSRKTFETEFQKTKHQEWDFVVTTDISEMGANF      1920
PRVABC59_1          TVWFVPSVRNGNEIAACLTKAGKRVIQLSRKTFETEFQKTKHQEWDFVVTTDISEMGANF      1920
HPF2013_1           TVWFVPSVRNGNEIAACLTKAGKRVIQLSRKTFETEFQKTKHQEWDFVVTTDISEMGANF      1920
Beh815744_1         TVWFVPSVRNGNEIAACLTKAGKRVIQLSRKTFETEFQKTKHQEWDFVVTTDISEMGANF      1920
BEH818995_1         TVWFVPSVRNGNEIAACLTKAGKRVIQLSRKTFETEFQKTKHQEWDFVVTTDISEMGANF      1920
BEH819966_1         TVWFVPSVRNGNEIAACLTKAGKRVIQLSRKTFETEFQKTKHQEWDFVVTTDISEMGANF      1920
BeH819015_1         TVWFVPSVRNGNEIAACLTKAGKRVIQLSRKTFETEFQKTKHQEWDFVVTTDISEMGANF      1920
SSABr_1             TVWFVPSVRNGNEIAACLTKAGKRVIQLSRKTFETEFQKTKHQEWDFVVTTDISEMGANF      1920
ARD157995_1         TVWFVPSVRSGEESAACLTKAGKRVIQLSRKTFETEFQKTKNQEWDFVITTDISEMGANF      1920
MR_766_1            TVWFVPSVRNGNEIAACLTKAGKRVIQLSRKTFETEFQKTKNQEWDFVITTDISEMGANF      1916
ARD158084_1         TVWFVPSVRNGNEIAACLTKAGKRVIQLSRKTFETEFQKTKNQEWDFVITTDISEMGANF      1920
ArB1362_1           TVWFVPSVRNGNEITACLTKAGKRVIQLSRKTFETEFQKTKNQEWDFVITTDISEMGANF      1920
ARB15076_1          TVWFVPSVRNGNEIAACLTKAGKRVIQLSRKTFETEFQKTKNQEWDFVITTDISEMGANF      1914
ARB13565_1          TVWFVPSVRNGNEIAACLTKAGKRVIQLSRKTFETEFQKTKNQEWDFVITTDISEMGANF      1920
ARB7701_1           TVWFVPSVRNGNEIAACLTKAGKRVIQLSRKTFETEFQKTKNQEWDFVITTDISEMGANF      1920
IbH30656_1          TIWFVPSVRNGNEIAACLTKAGKRVIQLSRKTFETEFQKTKNQEWDFVITTDISEMGANF      1914
ArD128000_1         TIWFVPSVRNGNEIAACLTKAGKRVIQLSRKTFETEFQKTKNQEWDFVITTDISEMGANF      1920
ArD7117_1           TIWFVPSVRSGEESAACLTKAGKRVIQLSRKTFETEFQKTKNQEWDFVITTDISEMGANF      1920
ARD_41519_1         TIWFVPSVRNGNEIAACLTKAGKRVIQLSRKTFETEFQKTKNQEWDFVITTDISEMGANF      1920
                    *:*******.*:* :********************** ***.******:***********
```

```
P6-740_1            KADRVIDSRRCLKPVILDGERVILAGPMPVTHASAAQRRGRIGRNPNKPGDEYMYGGGCA    1980
P6740               KADRVIDSRRCLKPVILDGERVILAGPMPVTHASAAQRRGRIGRNPNKPGDEYMYGGGCA    1980
Yap2007             KADRVIDSRRCLKPVILDGERVILAGPMPVTHASAAQRRGRIGRNPNKPGDEYLYGGGCA    1980
CPC0740_1           KADRVIDSRRCLKPVILDGERVILAGPMPVTHASAAQRRGRIGRNPNKPGDEYLYGGGCA    1980
SV0127/14_1         KADRVIDSRRCLKPVILDGERVILAGPMPVTHASAAQRRGRIGRNPNKPGDEYLYGGGCA    1980
FSS13025_1          KADRVIDSRRCLKPVILDGERVILAGPMPVTHASAAQRRGRIGRNPNKPGDEYLYGGGCA    1980
8375_1              KADRVIDSRRCLKPVILDGERVILAGPMPVTHASAAQRRGRIGRNPNKPGDEYLYGGGCA    1980
103344_1            KADRVIDSRRCLKPVILDGERVILAGPMPVTHASAAQRRGRIGRNPNKPGDEYLYGGGCA    1980
GD01_1              KADRVIDSRRCLKPVILDGERVILAGPMPVTHASAAQRRGRIGRNPNKPGDEYLYGGGCA    1980
SPH2015_1           KADRVIDSRRCLKPVILDGERVILAGPMPVTHASAAQRRGRIGRNPNKPGDEYLYGGGCA    1980
Haiti2014_1         KADRVIDSRRCLKPVILDGERVILAGPMPVTHASAAQRRGRIGRNPNKPGDEYLYGGGCA    1980
PLCal_ZV_1          KADRVIDSRRCLKPVILDGERVILAGPMPVTHASAAQRRGRIGRNPNKPGDEYLYGGGCA    1943
Martinique          KADRVIDSRRCLKPVILDGERVILAGPMPVTHASAAQRRGRIGRNPNKPGDEYLYGGGCA    1980
NatalRGN            KADRVIDSRRCLKPVILDGERVILAGPMPVTHASAAQRRGRIGRNPNKPGDEYLYGGGCA    1980
BrasilZKV2015_1     KADRVIDSRRCLKPVILDGERVILAGPMPVTHASAAQRRGRIGRNPNKPGDEYLYGGGCA    1980
Z1106033_1          KADRVIDSRRCLKPVILDGERVILAGPMPVTHASAAQRRGRIGRNPNKPGDEYLYGGGCA    1980
PRVABC59_1          KADRVIDSRRCLKPVILDGERVILAGPMPVTHASAAQRRGRIGRNPNKPGDEYLYGGGCA    1980
HPF2013_1           KADRVIDSRRCLKPVILDGERVILAGPMPVTHASAAQRRGRIGRNPNKPGDEYLYGGGCA    1980
Beh815744_1         KADRVIDSRRCLKPVILDGERVILAGPMPVTHASAAQRRGRIGRNPNKPGDEYLYGGGCA    1980
BEH818995_1         KADRVIDSRRCLKPVILDGERVILAGPMPVTHASAAQRRGRIGRNPNKPGDEYLYGGGCA    1980
BEH819966_1         KADRVIDSRRCLKPVILDGERVILAGPMPVTHASAAQRRGRIGRNPNKPGDEYLYGGGCA    1980
BeH819015_1         KADRVIDSRRCLKPVILDGERVILAGPMPVTHASAAQRRGRIGRNPNKPGDEYLYGGGCA    1980
SSABr_1             KADRVIDSRRCLKPVILDGERVILAGPMPVTHASAAQRRGRIGRNPNKPGDEYLYGGGCA    1980
ARD157995_1         KADRVIDSRRCLKPVILDGERVILAGPMPVTHASAAQRRGRIGRNPNKPGDEYMYGGGCA    1980
MR_766_1            KADRVIDSRRCLKPVILDGERVILAGPMPVTHASAAQRRGRIGRNPNKPGDEYMYGGGCA    1976
ARD158084_1         KADRVIDSRRCLKPVILDGERVILAGPMPVTHASAAQRRGRIGRNPNKPGDEYMYGGGCA    1980
ArB1362_1           KADRVIDSRRCLKPVILDGERVILAGPMPVTHASAAQRRGRIGRNPNKPGDEYMYGGGCA    1980
ARB15076_1          KADRVIDSRRCLKPVILDGERVILAGPMPVTHASAAQRRGRIGRNPNKPGDEYMYGGGCA    1974
ARB13565_1          KADRVIDSRRCLKPVILDGERVILAGPMPVTHASAAQRRGRIGRNPNKPGDEYMYGGGCA    1980
ARB7701_1           KADRVIDSRRCLKPVILDGERVILAGPMPVTHASAAQRRGRIGRNPNKPGDEYMYGGGCA    1980
IbH30656_1          KADRVIDSRRCLKPVILDGERVILAGPMPVTHASAAQRRGRIGRNPNKPGDEYMYGGGCA    1974
ArD128000_1         KADRVIDSRRCLKPVILDGERVILAGPMPVTHASAAQRRGRIGRNPNKPGDEYMYGGGCA    1980
ArD7117_1           KADRVIDSRRCLKPVILDGERVILAGPMPVTHASAAQRRGRIGRNPNKPGDEYMYGGGCA    1980
ARD_41519_1         KADRVIDSRRCLKPVILDGERVILAGPMPVTHASAAQRRGRVGRNPNKPGDEYMYGGGCA    1980
                    *****************************************:***********:******

P6-740_1            ETDEDHAHWLEARMLLDNIYLQDGLIASLYRPEADKVAAIEGEFKLRTEQRKTFVELMKR    2040
P6740               ETDEDHAHWLEARMLLDNIYLQDGLIASLYRPEADKVAAIEGEFKLRTEQRKTFVELMKR    2040
Yap2007             ETDEDHAHWLEARMLLDNIYLQDGLIASLYRPEADKVAAIEGEFKLRTEQRKTFVELMKR    2040
CPC0740_1           ETDEDHAHWLEARMLLDNIYLQDGLIASLYRPEADKVAAIEGEFKLRTEQRKTFVELMKR    2040
SV0127/14_1         ETDEDHAHWLEARMLLDNIYLQDGLIASLYRPEADKVAAIEGEFKLRTEQRKTFVELMKR    2040
FSS13025_1          ETDEDHAHWLEARMLLDNIYLQDGLIASLYRPEADKVAAIEGEFKLRTEQRKTFVELMKR    2040
8375_1              ETDEDHAHWLEARMLLDNIYLQDGLIASLYRPEADKVAAIEGEFKLRTEQRKTFVELMKR    2040
103344_1            ETDEDHAHWLEARMLLDNIYLQDGLIASLYRPEADKVAAIEGEFKLRTEQRKTFVELMKR    2040
GD01_1              ETDEDHAHWLEARMLLDNIYLQDGLIASLYRPEADKVAAIEGEFKLRTEQRKTFVELMKR    2040
SPH2015_1           ETDEDHAHWLEARMLLDNIYLQDGLIASLYRPEADKVAAIEGEFKLRTEQRKTFVELMKR    2040
Haiti2014_1         ETDEDHAHWLEARMLLDNIYLQDGLIASLYRPEADKVAAIEGEFKLRTEQRKTFVELMKR    2040
PLCal_ZV_1          ETDEDHAHWLEARMLLDNIYLQDGLIASLYRPEADKVAAIEGEFKLRTEQRKTFVELMKR    2003
Martinique          ETDEDHAHWLEARMLLDNIYLQDGLIASLYRPEADKVAAIEGEFKLRTEQRKTFVELMKR    2040
NatalRGN            ETDEDHAHWLEARMLLDNIYLQDGLIASLYRPEADKVAAIEGEFKLRTEQRKTFVELMKR    2040
BrasilZKV2015_1     ETDEDHAHWLEARMLLDNIYLQDGLIASLYRPEADKVAAIEGEFKLRTEQRKTFVELMKR    2040
Z1106033_1          ETDEDHAHWLEARMLLDNIYLQDGLIASLYRPEADKVAAIEGEFKLRTEQRKTFVELMKR    2040
PRVABC59_1          ETDEDHAHWLEARMLLDNIYLQDGLIASLYRPEADKVAAIEGEFKLRTEQRKTFVELMKR    2040
HPF2013_1           ETDEDHAHWLEARMLLDNIYLQDGLIASLYRPEADKVAAIEGEFKLRTEQRKTFVELMKR    2040
Beh815744_1         ETDEDHAHWLEARMLLDNIYLQDGLIASLYRPEADKVAAIEGEFKLRTEQRKTFVELMKR    2040
BEH818995_1         ETDEDHAHWLEARMLLDNIYLQDGLIASLYRPEADKVAAIEGEFKLRTEQRKTFVELMKR    2040
BEH819966_1         ETDEDHAHWLEARMLLDNIYLQDGLIASLYRPEADKVAAIEGEFKLRTEQRKTFVELMKR    2040
BeH819015_1         ETDEDHAHWLEARMLLDNIYLQDGLIASLYRPEADKVAAIEGEFKLRTEQRKTFVELMKR    2040
SSABr_1             ETDEDHAHWLEARMLLDNIYLQDGLIASLYRPEADKVAAIEGEFKLRTEQRKTFVELMKR    2040
ARD157995_1         ETDEDHAHWLEARMLLDNIYLQDGLIASLYRPEADKVAAIEGEFKLRTEQRKTFVELMKR    2040
MR_766_1            ETDEGHAHWLEARMLLDNIYLQDGLIASLYRPEADKVAAIEGEFKLRTEQRKTFVELMKR    2036
ARD158084_1         ETDEGHAHWLEARMLLDNIYLQDGLIASLYRPEADKVAAIEGEFKLRTEQRKTFVELMKR    2040
ArB1362_1           ETDEDHAHWLEARMLLDNIYLQDGLIASLYRPEADKVAAIEGEFKLRTEQRKTFVELMKR    2040
ARB15076_1          ETDEDHAHWLEARMLLDNIYLQDGLIASLYRPEADKVAAIEGEFKLRTEQRKTFVELMKR    2034
ARB13565_1          ETDEDHAHWLEARMLLDNIYLQDGLIASLYRPEADKVAAIEGEFKLRTEQRKTFVELMKR    2040
ARB7701_1           ETDEDHAHWLEARMLLDNIYLQDGLIASLYRPEADKVAAIEGEFKLRTEQRKTFVELMKR    2040
IbH30656_1          ETDEDHAHWLEARMLLDNIYLQDGLIASLYRPEADKVAAIEGEFKLRTEQRKTFVELMKR    2034
```

```
ArD128000_1         ETDEDHAHWLEARMLLDNIYLQDGLIASLYRPEADKVAAIEGEFKLRTEQRKTFVELMKR    2040
ArD7117_1           ETDEDHAHWLEARMLLDNIYLQDGLIASLYRPEADKVAAIEGEFKLRTEQRKTFVELMKR    2040
ARD_41519_1         ETDEDHAHWLEARMLLDNIYLQDGLIASLYRPEADKVAAIEGEFKLRTEQRKTFVELMKR    2040
                    **** *******************************************************

P6-740_1            GDLPVWLAYQVASAGITYTDRRWCFDGTTNNTIMEDSVPAEVWTRYGEKRVLKPRWMDAR    2100
P6740               GDLPVWLAYQVASAGITYTDRRWCFDGTTNNTIMEDSVPAEVWTRYGEKRVLKPRWMDAR    2100
Yap2007             GDLPVWLAYQVASAGITYTDRKWCFDGTTNNTIMEDSVPAEVWTRYGEKRVLKPRWMDAR    2100
CPC0740_1           GDLPVWLAYQVASAGITYTDRRWCFDGMTNNTIMEDSVPAEVWTRYGEKRVLKPRWMDAR    2100
SV0127/14_1         GDLPVWLAYQVASAGITYTDRRWCFDGMTNNTIMEDSVPAEVWTRHGEKRVLKPRWMDAR    2100
FSS13025_1          GDLPVWLAYQVASAGITYTDRRWCFDGTTNNTIMEDSVPAEVWTRYGEKRVLKPRWMDAR    2100
8375_1              GDLPVWLAYQVASAGITYTDRRWCFDGTTNNTILEDSVPAEVWTRHGEKRVLKPRWMDAR    2100
103344_1            GDLPVWLAYQVASAGITYTDRRWCFDGTTNNTILEDSVPAEVWTRHGEKRVLKPRWMDAR    2100
GD01_1              GDLPVWLAYQVASAGITYTDRRWCFDGTTNNTIMEDSVPAEVWTRHGEKRVLKPRWMDAR    2100
SPH2015_1           GDLPVWLAYQVASAGITYTDRRWCFDGTTNNTIMEDSVPAEVWTRHGEKRVLKPRWMDAR    2100
Haiti2014_1         GDLPVWLAYQVASAGITYTDRRWCFDGTTNNTIMEDSVPAEVWTRHGEKRVLKPRWMDAR    2100
PLCal_ZV_1          GDLPVWLAYQVASAGITYTDRRWCFDGTTNNTIMEDSVPAEVWTRYGEKRVLKPRWMDAR    2063
Martinique          GDLPVWLAYQVASAGITYTDRRWCFDGTTNNTIMEDSVPAEVWTRHGEKRVLKPRWMDAR    2100
NatalRGN            GDLPVWLAYQVASAGITYTDRRWCFDGTTNNTIMEDSVPAEVWTRHGEKRVLKPRWMDAR    2100
BrasilZKV2015_1     GDLPVWLAYQVASAGITYTDRRWCFDGTTNNTIMEDSVPAEVWTRHGEKRVLKPRWMDAR    2100
Z1106033_1          GDLPVWLAYQVASAGITYTDRRWCFDGTTNNTIMEDSVPAEVWTRHGEKRVLKPRWMDAR    2100
PRVABC59_1          GDLPVWLAYQVASAGITYTDRRWCFDGTTNNTIMEDSVPAEVWTRHGEKRVLKPRWMDAR    2100
HPF2013_1           GDLPVWLAYQVASAGITYTDRRWCFDGTTNNTIMEDSVPAEVWTRHGEKRVLKPRWMDAR    2100
Beh815744_1         GDLPVWLAYQVASAGITYTDRRWCFDGTTNNTIMEDSVPAEVWTRHGEKRVLKPRWMDAR    2100
BEH818995_1         GDLPVWLAYQVASAGITYTDRRWCFDGTTNNTIMEDSVPAEVWTRHGEKRVLKPRWMDAR    2100
BEH819966_1         GDLPVWLAYQVASAGITYTDRRWCFDGTTNNTIMEDSVPAEVWTRHGEKRVLKPRWMDAR    2100
BeH819015_1         GDLPVWLAYQVASAGITYTDRRWCFDGTTNNTIMEDSVPAEVWTRHGEKRVLKPRWMDAR    2100
SSABr_1             GDLPVWLAYQVASAGITYTDRRWCFDGTTNNTIMEDSVPAEVWTRHGEKRVLKPRWMDAR    2100
ARD157995_1         GDLPVWLAYQVASAGITYTDRRWCFDGTTNNTIMEDSVPAEVWTKYGEKRVLKPRWMDAR    2100
MR_766_1            GDLPVWLAYQVASAGITYTDRRWCFDGTTNNTIMEDSVPAEVWTKYGEKRVLKPRWMDAR    2096
ARD158084_1         GDLPVWLAYQVASAGITYTDRRWCFDGTTNNTIMEDSVPAEVWTKYGEKRVLKPRWMDAR    2100
ArB1362_1           GDLPVWLAYQVASAGITYTDRRWCFDGTTNNTIMEDSVPAEVWTKYGEKRVLKPRWMDAR    2100
ARB15076_1          GDLPVWLAYQVASAGITYTDRRWCFDGTTNNTIMEDSVPAEVWTKYGEKRVLKPRWMDAR    2094
ARB13565_1          GDLPVWLAYQVASAGITYTDRRWCFDGTTNNTIMEDSVPAEVWTKYGEKRVLKPRWMDAR    2100
ARB7701_1           GDLPVWLAYQVASAGITYTDRRWCFDGTTNNTIMEDSVPAEVWTKYGEKRVLKPRWMDAR    2100
IbH30656_1          GDLPVWLAYQVASAGITYTDRRWCFDGTTNNTIMEDSVPAEVWTKYGEKRVLKPRWMDAR    2094
ArD128000_1         GDLPVWLAYQVASAGITYTDRRWCFDGTTNNTIMEDSVPAEVWTKYGEKRVLKPRWMDAR    2100
ArD7117_1           GDLPVWLAYQVASAGITYTDRRWCFDGTTNNTIMEDSVPAEVWTKYGEKRVLKPRWMDAR    2100
ARD_41519_1         GDLPVWLAYQVASAGITYTDRRWCFDGTTNNTIMEDSVPAEVWTKYGEKRVLKPRWMDAR    2100
                    *********************:***** *****:**********::**************

P6-740_1            VCSDHAALKSFKEFAAGKRGAAFGVMEALGTLPGHMTERFQEAIDNLAVLMRAETGSRPY    2160
P6740               VCSDHAALKSFKEFAAGKRGAAFGVMEALGTLPGHMTERFQEAIDNLAVLMRAETGSRPY    2160
Yap2007             VCSDHAALKSFKEFAAGKRGAAFGVMEALGTLPGHMTERFQEAIDNLAVLMRAETGSRPY    2160
CPC0740_1           VCSDHAALKSFKEFAAGKRGAAFGVIEALGTLPGHMTERFQEAIDNLAVLMRAETGSRPY    2160
SV0127/14_1         VCSDHAALKSFKEFAAGKRGAAFGVMEALGTLPGHMTERFQEAIDNLAVLMRAETGSRPY    2160
FSS13025_1          VCSDHAALKSFKEFAAGKRGAAFGVMEALGTLPGHMTERFQEAIDNLAVLMRAETGSRPY    2160
8375_1              VCSDHAALKSFKEFAAGKRGAAFGVMEALGTLPGHMTERFQEAIDNLAVLMRAETGSRPY    2160
103344_1            VCSDHAALKSFKEFAAGKRGAAFGVMEALGTLPGHMTERFQEAIDNLAVLMRAETGSRPY    2160
GD01_1              VCSDHAALKSFKEFAAGKRGAAFGVMEALGTLPGHMTERFQEAIDNLAVLMRAETGSRPY    2160
SPH2015_1           VCSDHAALKSFKEFAAGKRGAAFGVMEALGTLPGHMTERFQEAIDNLAVLMRAETGSRPY    2160
Haiti2014_1         VCSDHAALKSFKEFAAGKRGAAFGVMEALGTLPGHMTERFQEAIDNLAVLMRAETGSRPY    2160
PLCal_ZV_1          VCSDHAALKSFKEFAAGKRGAAFGVMEALGTLPGHMTERFQEAIDNLAVLMRAETGSRPY    2123
Martinique          VCSDHAALKSFKEFAAGKRGAAFGVMEALGTLPGHMTERFQEAIDNLAVLMRAETGSRPY    2160
NatalRGN            VCSDHAALKSFKEFAAGKRGAAFGVMEALGTLPGHMTERFQEAIDNLAVLMRAETGSRPY    2160
BrasilZKV2015_1     VCSDHAALKSFKEFAAGKRGAAFGVMEALGTLPGHMTERFQEAIDNLAVLMRAETGSRPY    2160
Z1106033_1          VCSDHAALKSFKEFAAGKRGAAFGVMEALGTLPGHMTERFQEAIDNLAVLMRAETGSRPY    2160
PRVABC59_1          VCSDHAALKSFKEFAAGKRGAAFGVMEALGTLPGHMTERFQEAIDNLAVLMRAETGSRPY    2160
HPF2013_1           VCSDHAALKSFKEFAAGKRGAAFGVMEALGTLPGHMTERFQEAIDNLAVLMRAETGSRPY    2160
Beh815744_1         VCSDHAALKSFKEFAAGKRGAAFGVMEALGTLPGHMTERFQEAIDNLAVLMRAETGSRPY    2160
BEH818995_1         VCSDHAALKSFKEFAAGKRGAAFGVMEALGTLPGHMTERFQEAIDNLAVLMRAETGSRPY    2160
BEH819966_1         VCSDHAALKSFKEFAAGKRGAAFGVMEALGTLPGHMTERFQEAIDNLAVLMRAETGSRPY    2160
BeH819015_1         VCSDHAALKSFKEFAAGKRGAAFGVMEALGTLPGHMTERFQEAIDNLAVLMRAETGSRPY    2160
SSABr_1             VCSDHAALKSFKEFAAGKRGAAFGVMEALGTLPGHMTERFQEAIDNLAVLMRAETGSRPY    2160
ARD157995_1         VCSDHAALKSFKEFAAGKRGAALGVMEALGTLPGHMTERFQEAIDNLAVLMRAETGSRPY    2160
MR_766_1            VCSDHAALKSFKEFAAGKRGAALGVMEALGTLPGHMTERFQEAIDNLAVLMRAETGSRPY    2156
ARD158084_1         VCSDHAALKSFKEFAAGKRGAALGVMEALGTLPGHMTERFQEAIDNLAVLMRAETGSRPY    2160
ArB1362_1           VCSDHAALKSFKEFAAGKRGAALGVMEALGTLPGHMTERFQEAIDNLAVLMRAETGSRPY    2160
```

```
ARB15076_1          VCSDHAALKSFKEFAAGKRGAALGVMEALGTLPGHMTERFQEAIDNLAVLMRAETGSRPY       2154
ARB13565_1          VCSDHAALKSFKEFAAGKRGVALGVMEALGTLPGHMTERFQEAIDNLAVLMRAETGSRPY       2160
ARB7701_1           VCSDHAALKSFKEFAAGKRGVALGVMEALGTLPGHMTERFQEAIDNLAVLMRAETGSRPY       2160
IbH30656_1          VCSDHAALKSFKEFAAGKRGAALGVMDALGTLPGHMTERFQEAIDNLAVLMRAETGSRPY       2154
ArD128000_1         VCSDHAALKSFKEFAAGKRGAALGVMDALGTLPGHMTERFQEAIDNLAVLMRAETGSRPY       2160
ArD7117_1           VCSDHAALKSFKEFAAGKRGAALGVMDALGTLPGHMTERFQEAIDNLAVLMRAETGSRPY       2160
ARD_41519_1         VCSDHAALKSFKEFAAGKRGAALGVMDALGTLPGHMTERFQEAIDNLAVLMRAETGSRPY       2160
                    ********************.*:**::*********************************

P6-740_1            KAAAAQLPETLETIMLLGLLGTVSLGIFFVLMRNKGIGKMGFGMVTLGASAWLMWLSEIE       2220
P6740               KAAAAQLPETLETIMLLGLLGTVSLGIFFVLMRNKGIGKMGFGMVTLGASAWLMWLSEIE       2220
Yap2007             EAAAAQLPETLETIMLLGLLGTVSLGIFFVLMRNKGIGKMGFGMVTLGASAWLMWLSEIE       2220
CPC0740_1           KAAAAQLPETLETIMLLGLLGTVSLGIFFVLMRNKGMGKMGFGMVTLGASAWLMWLSEIE       2220
SV0127/14_1         KAAAAQLPETLETIMLLGLLGTVSLGIFFVLMRNKGIGKMGFGMVTLGASAWLMWLSEIE       2220
FSS13025_1          KAAAAQLPETLETIMLLGLLGTVSLGIFFVLMRNKGIGKMGFGMVTLGASAWLMWLSEIE       2220
8375_1              KAAAAQLPETLETIMLLGLLGTVSLGIFFVLMRNKGIGKMGFGMVTLGASAWLMWLSEIE       2220
103344_1            KAAAAQLPETLETIMLLGLLGTVSLGIFFVLMRNKGIGKMGFGMVTLGASAWLMWLSEIE       2220
GD01_1              KAAAAQLPETLETIMLLGLLGTVSLGIFFVLMRNKGIGKMGFGMVTLGASAWLMWLSEIE       2220
SPH2015_1           KAAAAQLPETLETIMLLGLLGTVSLGIFFVLMRNKGIGKMGFGMVTLGASAWLMWLSEIE       2220
Haiti2014_1         KAAAAQLPETLETIMLLGLLGTVSLGIFFVLMRNKGIGKMGFGMVTLGASAWLMWLSEIE       2220
PLCal_ZV_1          KAAAAQLPETLETIMLLGLLGTVSLGIFFVLMRNKGIGKMGFGMVTLGASAWLMWLSEIE       2183
Martinique          KAAAAQLPETLETIMLLGLLGTVSLGIFFVLMRNKGIGKMGFGMVTLGASAWLMWLSEIE       2220
NatalRGN            KAAAAQLPETLETIMLLGLLGTVSLGIFFVLMRNKGIGKMGFGMVTLGASAWLMWLSEIE       2220
BrasilZKV2015_1     KAAAAQLPETLETIMLLGLLGTVSLGIFFVLMRNKGIGKMGFGMVTLGASAWLMWLSEIE       2220
Z1106033_1          KAAAAQLPETLETIMLLGLLGTVSLGIFFVLMRNKGIGKMGFGMVTLGASAWLMWLSEIE       2220
PRVABC59_1          KAAAAQLPETLETIMLLGLLGTVSLGIFFVLMRNKGIGKMGFGMVTLGASAWLMWLSEIE       2220
HPF2013_1           KAAAAQLPETLETIMLLGLLGTVSLGIFFVLMRNKGIGKMGFGMVTLGASAWLMWLSEIE       2220
Beh815744_1         KAAAAQLPETLETIMLLGLLGTVSLGIFFVLMRNKGIGKMGFGMVTLGASAWLMWLSEIE       2220
BEH818995_1         KAAAAQLPETLETIMLLGLLGTVSLGIFFVLMRNKGIGKMGFGMVTLGASAWLMWLSEIE       2220
BEH819966_1         KAAAAQLPETLETIMLLGLLGTVSLGIFFVLMRNKGIGKMGFGMVTLGASAWLMWLSEIE       2220
BeH819015_1         KAAAAQLPETLETIMLLGLLGTVSLGIFFVLMRNKGIGKMGFGMVTLGASAWLMWLSEIE       2220
SSABr_1             KAAAAQLPETLETIMLLGLLGTVSLGIFFVLMRNKGIGKMGFGMVTLGASAWLMWLSEIE       2220
ARD157995_1         KAAAAQLPETLETIMLLGLLGTVSLGIFFVLMRNKGIGKMGFGMVTLGASAWLMWLSEIE       2220
MR_766_1            KAAAAQLPETLETIMLLGLLGTVSLGIFFVLMRNKGIGKMGFGMVTLGASAWLMWLSEIE       2216
ARD158084_1         KAAAAQLPETLETIMLLGLLGTVSLGIFFVLMRNKGIGKMGFGMVTLGASAWLMWLSEIE       2220
ArB1362_1           KAAAAPLPETLETIMLLGLLGTVSLGIFFVLMRNKGIGKMGFGMVTLGASAWLMWLSEIE       2220
ARB15076_1          KAAAAQLPETLETIMLLGLLGTVSLGIFFVLMRNKGIGKMGFGMVTLGASAWLMWLSEIE       2214
ARB13565_1          KAAAAQLPETLETIMLLGLLGTVSLGIFFVLMRNKGIGKMGFGMVTLGASAWLMWLSEIE       2220
ARB7701_1           KAAAAQLPETLETIMLLGLLGTVSLGIFFVLMRNKGIGKMGFGMVTLGASAWLMWLSEIE       2220
IbH30656_1          KAAAAQLPETLETIMLLGLLGTVSLGIFFVLMRNKGIGKMGFGMVTLGASAWLMWLSEIE       2214
ArD128000_1         KAAAAQLPETLETIMLLGLLGTVSLGIFFVLMRNKGIGKMGFGMVTLGASAWLMWLSEIE       2220
ArD7117_1           KAAAAQLPETLETIMLLGLLGTVSLGIFFVLMRNKGIGKMGFGMVTLGASAWLMWLSEIE       2220
ARD_41519_1         KAAAAQLPETLETIMLLGLLGTVSLGIFFVLMRNKGIGKMGFGMVTLGASAWLMWLSEIE       2220
                    :**** ******************************:***********************

P6-740_1            PARIACVLIVVFLLLVVLIPEPEKQRSPQDNQMAIIIMVAVGLLGLITANELGWLERTKS       2280
P6740               PARIACVLIVVFLLLVVLIPEPEKQRSPQDNQMAIIIMVAVGLLGLITANELGWLERTKS       2280
Yap2007             PARIACVLIVVFLLLVVLIPEPEKQRSPQDNQMAIIIMIAVGLLGLITANELGWLERTKS       2280
CPC0740_1           PARIACVLIVVFLLLVVLIPEPEKQRSPQDNQMAIIIMVAVGLLGLITANELGWLERTKS       2280
SV0127/14_1         PARIACVLIVVFLLLVVLIPEPEKQRSPQDNQMAIIIMVAVGLLGLITANELGWLERTKS       2280
FSS13025_1          PARIACVLIVVFLLLVVLIPEPEKQRSPQDNQMAIIIMVAVGLLGLITANELGWLERTKS       2280
8375_1              PARIACVLIVVFLLLVVLIPEPEKQRSPQDNQMAIIIMVAVGLLGLITANELGWLERTKS       2280
103344_1            PARIACVLIVVFLLLVVLIPEPEKQRSPQDNQMAIIIMVAVGLLGLITANELGWLERTKS       2280
GD01_1              PARIACVLIVVFLLLVVLIPEPEKQRSPQDNQMAIIIMVAVGLLGLITANELGWLERTKS       2280
SPH2015_1           PARIACVLIVVFLLLVVLIPEPEKQRSPQDNQMAIIIMVAVGLLGLITANELGWLERTKS       2280
Haiti2014_1         PARIACVLIVVFLLLVVLIPEPEKQRSPQDNQMAIIIMVAVGLLGLITANELGWLERTKS       2280
PLCal_ZV_1          PARIACVLIVVFLLLVVLIPEPEKQRSPQDNQMAIIIMVAVGLLGLITANELGWLERTKS       2243
Martinique          PARIACVLIVVFLLLVVLIPEPEKQRSPQDNQMAIIIMVAVGLLGLITANELGWLERTKS       2280
NatalRGN            PARIACVLIVVFLLLVVLIPEPEKQRSPQDNQMAIIIMVAVGLLGLITANELGWLERTKS       2280
BrasilZKV2015_1     PARIACVLIVVFLLLVVLIPEPEKQRSPQDNQMAIIIMVAVGLLGLITANELGWLERTKS       2280
Z1106033_1          PARIACVLIVVFLLLVVLIPEPEKQRSPQDNQMAIIIMVAVGLLGLITANELGWLERTKS       2280
PRVABC59_1          PARIACVLIVVFLLLVVLIPEPEKQRSPQDNQMAIIIMVAVGLLGLITANELGWLERTKS       2280
HPF2013_1           PARIACVLIVVFLLLVVLIPEPEKQRSPQDNQMAIIIMVAVGLLGLITANELGWLERTKS       2280
Beh815744_1         PARIACVLIVVFLLLVVLIPEPEKQRSPQDNQMAIIIMVAVGLLGLITANELGWLERTKS       2280
BEH818995_1         PARIACVLIVVFLLLVVLIPEPEKQRSPQDNQMAIIIMVAVGLLGLITANELGWLERTKS       2280
BEH819966_1         PARIACVLIVVFLLLVVLIPEPEKQRSPQDNQMAIIIMVAVGLLGLITANELGWLERTKS       2280
BeH819015_1         PARIACVLIVVFLLLVVLIPEPEKQRSPQDNQMAIIIMVAVGLLGLITANELGWLERTKS       2280
SSABr_1             PARIACVLIVVFLLLVVLIPEPEKQRSPQDNQMAIIIMVAVGLLGLITANELGWLERTKS       2280
```

```
ARD157995_1         PARIACVLIVVFLLLVVLIPEPEKQRSPQDNQMAIIMVAVGLLGLITANELGWLERTKN       2280
MR_766_1            PARIACVLIVVFLLLVVLIPEPEKQRSPQDNQMAIIMVAVGLLGLITANELGWLERTKN       2276
ARD158084_1         PARIACVLIVVFLLLVVLIPEPEKQRSPQDNQMAIIMVAVGLLGLITANELGWLERTKN       2280
ArB1362_1           PARIACVLIVVFLLLVVLIPEPEKQRSPQDNQMAIIMVAVGLLGLITANELGWLERTKN       2280
ARB15076_1          PARIACVLIVVFLLLVVLIPEPEKQRSPQDNQMAIIMVAVGLLGLITANELGWLERTKN       2274
ARB13565_1          PARIACVLIVVFLLLVVLIPEPEKQRSPQDNQMAIIMVAVGLLGLITANELGWLERTKN       2280
ARB7701_1           PARIACVLIVVFLLLVVLIPEPEKQRSPQDNQMAIIMVAVGLLGLITANELGWLERTKN       2280
IbH30656_1          PARIACVLIVVFLLLVVLIPEPEKQRSPQDNQMAIIMVAVGLLGLITANELGWLERTKS       2274
ArD128000_1         PARIACVLIVVFLLLVVLIPEPEKQRSPQDNQMAIIMVAVGLLGLITANELGWLERTKS       2280
ArD7117_1           PARIACVLIVVFLLLVVLIPEPEKQRSPQDNQMAIIMVAVGLLGLITANELGWLERTKS       2280
ARD_41519_1         PARIACVLIVVFLLLVVLIPEPEKQRSPQDNQMAIIMVAVGLLGLITANELGWLERTKS       2280
                    **************************************:********************.

P6-740_1            DLGHLMGRREEGATMGFSMDIDLRPASAWAIYAALTTLITPAVQHAVTTSYNNYSLMAMA       2340
P6740               DLGHLMGRREEGATMGFSMDIDLRPASAWAIYAALTTLITPAVQHAVTTSYNNYSLMAMA       2340
Yap2007             DLSHLMGRREEGATIGFSMDIDLRPASAWAIYAALTTFITPAVQHAVTTSYNNYSLMAMA       2340
CPC0740_1           DLSHLMGRREEGATTGFSMDIDLRPASAWAIYAALTTFITPAVQHAVTTSYNNYSLMAMA       2340
SV0127/14_1         DLSHLMGRREEGATIGFSMDIDLRPASAWAIYAALTTFITPAVQHAVTTSYNNYSLMAMA       2340
FSS13025_1          DLSHLMGRREEGATIGFSMDIDLRPASAWAIYAALTTFITPAVQHAVTTSYNNYSLMAMA       2340
8375_1              DLSHLMGRREEGATIGFSMDIDLRPASAWAIYAALTTFITPAVQHAVTTSYNNYSLMAMA       2340
103344_1            DLSHLMGRREEGATIGFSMDIDLRPASAWAIYAALTTFITPAVQHAVTTSYNNYSLMAMA       2340
GD01_1              DLSHLMGRREEGATIGFSMDIDLRPASAWAIYAALTTFITPAVQHAVTTSYNNYSLMAMA       2340
SPH2015_1           DLSHLMGRREEGATMGFSMDIDLRPASAWAIYAALTTFITPAVQHAVTTSYNNYSLMAMA       2340
Haiti2014_1         DLSHLMGRREEGATMGFSMDIDLRPASAWAIYAALTTFITPAVQHAVTTSYNNYSLMAMA       2340
PLCal_ZV_1          DLSHLMGRREEGATIGFSMDIDLRPASAWAIYAALTTFITPAVQHAVTTSYNNYSLMAMA       2303
Martinique          DLSHLMGRREEGATIGFSMDIDLRPASAWAIYAALTTFITPAVQHAVTTSYNNYSLMAMA       2340
NatalRGN            DLSHLMGRREEGATIGFSMDIDLRPASAWAIYAALTTFITPAVQHAVTTSYNNYSLMAMA       2340
BrasilZKV2015_1     DLSHLMGRREEGATIGFSMDIDLRPASAWAIYAALTTFITPAVQHAVTTSYNNYSLMAMA       2340
Z1106033_1          DLSHLMGRREEGATIGFSMDIDLRPASAWAIYAALTTFITPAVQHAVTTSYNNYSLMAMA       2340
PRVABC59_1          DLSHLMGRREEGATIGFSMDIDLRPASAWAIYAALTTFITPAVQHAVTTSYNNYSLMAMA       2340
HPF2013_1           DLSHLMGRREEGATIGFSMDIDLRPASAWAIYAALTTFITPAVQHAVTTSYNNYSLMAMA       2340
Beh815744_1         DLSHLMGRREEGATIGFSMDIDLRPASAWAIYAALTTFITPAVQHAVTTSYNNYSLMAMA       2340
BEH818995_1         DLSHLMGRREEGATIGFSMDIDLRPASAWAIYAALTTFITPAVQHAVTTSYNNYSLMAMA       2340
BEH819966_1         DLSHLMGRREEGATIGFSMDIDLRPASAWAIYAALTTFITPAVQHAVTTSYNNYSLMAMA       2340
BeH819015_1         DLSHLMGRREEGATIGFSMDIDLRPASAWAIYAALTTFITPAVQHAVTTSYNNYSLMAMA       2340
SSABr_1             DLSHLMGRREEGATIGFSMDIDLRPASAWAIYAALTTFITPAVQHAVTTSYNNYSLMAMA       2340
ARD157995_1         DIAHLMGRREEGATMGFSMDIDLRPASAWAIYAALTTLITPAVQHAVTTSYNNYSLMAMA       2340
MR_766_1            DIAHLMGRREEGATMGFSMDIDLRPASAWAIYAALTTLITPAVQHAVTTSYNNYSLMAMA       2336
ARD158084_1         DIAHLMGRREEGATMGFSMDIDLRPASAWAIYAALTTLITPAVQHAVTTSYNNYSLMAMA       2340
ArB1362_1           DIAHLMGRREEGVTMGFSMDIDLRPASAWAIYAALTTLITPAVQHAVTTSYNNYSLMAMA       2340
ARB15076_1          DIAHLMGRREEGVTMGFSMDIDLRPASAWAIYAALTTLITPAVQHAVTTSYNNYSLMAMA       2334
ARB13565_1          DIAHLMGRREEGVTMGFSMDIDLRPASAWAIYAALTTLITPAVQHAVTTSYNNYSLMAMA       2340
ARB7701_1           DIAHLMGRREEGVTMGFSMDIDLRPASAWAIYAALTTLITPAVQHAVTTSYNNYSLMAMA       2340
IbH30656_1          DIAHLMGRKEEGTTVGFSMDIDLRPASAWAIYAALTTLITPAVQHAVTTSYNNYSLMAMA       2334
ArD128000_1         DIAYLMGRKEEGTTIGFSMDIDLRPASAWAIYAALTTLITPAVQHAVTTSYNNYSLMAMA       2340
ArD7117_1           DIAHLMGRKEEGTTIGFSMDIDLRPASAWAIYAALTTLITPAVQHAVTTSYNNYSLMAMA       2340
ARD_41519_1         DIAHLMGRKEEGTTMGFSMDIDLRPASAWAIYAALTTLITPAVQHAVTTSYNNYSLMAMA       2340
                    *:.:****:***.* **********************:**********************

P6-740_1            TQAGVLFGMGKGMPFYAWDFGVPLLMMGCYSQLTPLTLIVAIILLVAHYMYLIPGLQAAA       2400
P6740               TQAGVLFGMGKGMPFYAWDFGVPLLMMGCYSQLTPLTLIVAIILLVAHYMYLIPGLQAAA       2400
Yap2007             TQAGVLFGMGKGMPFYAWDFGVPLLMMGCYSQLTPLTLIVAIILLVAHYMYLIPGLQAAA       2400
CPC0740_1           TQAGVLFGMGKGMPFYAWDFGVPLLMMGCYSQLTPLTLIVAIILLVAHYMYLIPGLQAAA       2400
SV0127/14_1         TQAGVLFGMGKGMPFYAWDFGVPLLMIGCYSQLTPLTLIVAIILLVAHYMYLIPGLQAAA       2400
FSS13025_1          TQAGVLFGMGKGMPFYAWDFGVPLLMIGCYSQLTPLTLIVAIILLVAHYMYLIPGLQAAA       2400
8375_1              TQAGVLFGMGKGMPFYAWDFGVPLLMIGCYSQLTPLTLIVAIILLVAHYMYLIPGLQAAA       2400
103344_1            TQAGVLFGMGKGMPFYAWDFGVPLLMIGCYSQLTPLTLIVAIILLVAHYMYLIPGLQAAA       2400
GD01_1              TQAGVLFGMGKGMPFYAWDFGVPLLMIGCYSQLTPLTLIVAIILLVAHYMYLIPGLQAAA       2400
SPH2015_1           TQAGVLFGMGKGMPFYAWDFGVPLLMIGCYSQLTPLTLIVAIILLVAHYMYLIPGLQAAA       2400
Haiti2014_1         TQAGVLFGMGKGMPFYAWDFGVPLLMIGCYSQLTPLTLIVAIILLVAHYMYLIPGLQAAA       2400
PLCal_ZV_1          TQAGVLFGMGKGMPFYAWDFGVPLLMIGCYSQLTPLTLIVAIILLVAHYMYLIPGLQAAA       2363
Martinique          TQAGVLFGMGKGMPFYAWDFGVPLLMIGCYSQLTPLTLIVAIILLVAHYMYLIPGLQAAA       2400
NatalRGN            TQAGVLFGMGKGMPFYAWDFGVPLLMIGCYSQLTPLTLIVAIILLVAHYMYLIPGLQAAA       2400
BrasilZKV2015_1     TQAGVLFGMGKGMPFYAWDFGVPLLMIGCYSQLTPLTLIVAIILLVAHYMYLIPGLQAAA       2400
Z1106033_1          TQAGVLFGMGKGMPFYAWDFGVPLLMIGCYSQLTPLTLIVAIILLVAHYMYLIPGLQAAA       2400
PRVABC59_1          TQAGVLFGMGKGMPFYAWDFGVPLLMIGCYSQLTPLTLIVAIILLVAHYMYLIPGLQAAA       2400
HPF2013_1           TQAGVLFGMGKGMPFYAWDFGVPLLMIGCYSQLTPLTLIVAIILLVAHYMYLIPGLQAAA       2400
Beh815744_1         TQAGVLFGMGKGMPFYAWDFGVPLLMIGCYSQLTPLTLIVAIILLVAHYMYLIPGLQAAA       2400
```

```
BEH818995_1         TQAGVLFGMGKGMPFYAWDFGVPLLMIGCYSQLTPLTLIVAIILLVAHYMYLIPGLQAAA      2400
BEH819966_1         TQAGVLFGMGKGMPFYAWDFGVPLLMIGCYSQLTPLTLIVAIILLVAHYMYLIPGLQAAA      2400
BeH819015_1         TQAGVLFGMGKGMPFYAWDFGVPLLMIGCYSQLTPLTLIVAIILLVAHYMYLIPGLQAAA      2400
SSABr_1             TQAGVLFGMGKGMPFYAWDFGVPLLMIGCYSQLTPLTLIVAIILLVAHYMYLIPGLQAAA      2400
ARD157995_1         TQAGVLFGMGKGMPFMHGDLGVPLLMMGCYSQLTPLTLIVAIILLVAHYMYLIPGLQAAA      2400
MR_766_1            TQAGVLFGMGKGMPFMHGDLGVPLLMMGCYSQLTPLTLIVAIILLVAHYMYLIPGLQAAA      2396
ARD158084_1         TQAGVLFGMGKGMPFYAWDFGVPLLMMGCYSQLTPLTLIVAIILLVAHYMYLIPGLQAAA      2400
ArB1362_1           TQAGVLFGMGKGMPFYAWDFGVPLLMMGCYSQLTPLTLIVAIILLVAHYMYLIPGLQAAA      2400
ARB15076_1          TQAGVLFGMGKGMPFYAWDFGVPLLMVGCYSQLTPLTLIVAIILLVAHYMYLIPGLQAAA      2394
ARB13565_1          TQAGVLFGMGKGMPFYAWDFGVPLLMMGCYSQLTPLTLIVAIILLVAHYMYLIPGLQAAA      2400
ARB7701_1           TQAGVLFGMGKGMPFYAWDFGVPLLMMGCYSQLTPLTLIVAIILLVAHYMYLIPGLQAAA      2400
IbH30656_1          TQAGVLFGMGKGMPFYAWDFGVPLLMMGCYSQLTPLTLIVAIILLVAHYMYLIPGLQAAA      2394
ArD128000_1         TQAGVLFGMGKGMPFYAWDFGVPLLMIGCYSQLTPLTLIVAIILLVAHYMYLIPGLQAAA      2400
ArD7117_1           TQAGVLFGMGKGMPFYAWDFGVPLLMMGCYSQLTPLTLIVAIILLVAHYMYLIPGLQAAA      2400
ARD_41519_1         TQAGVLFGMGKGMPFYAWDFGVPLLMMGCYSQLTPLTLIVAIILLVAHYMYLIPGLQAAA      2400
                    ***************   *:******:*********************************

P6-740_1            ARAAQKRTAAGIMKNPVVDGIVVTDIDTMTIDPQVEKKMGQVLLIAVAISSAVLLRTAWG      2460
P6740               ARAAQKRTAAGIMKNPVVDGIVVTDIDTMTIDPQVEKKMGQVLLIAVAISSAVLLRTAWG      2460
Yap2007             ARAAQKRTAAGIMKNPVVDGIVVTDIDTMTIDHRVEKKMGQVLLIAVAVSSAILSRTAWG      2460
CPC0740_1           ARAAQKRTAAGIMKNPVVDGIVVTDIDTMTIDPQVEKKMGQVLLIAVAVSSAILSRTAWG      2460
SV0127/14_1         ARAAQKRTAAGIMKNPVVDGIVVTDIDTMTIDPQVEKKMGQVLLIAVAVSSAILSRTAWG      2460
FSS13025_1          ARAAQKRTAAGIMKNPVVDGIVVTDIDTMTIDPQVEKKMGQVLLIAVAVSSAILSRTAWG      2460
8375_1              ARAAQKRTAAGIMKNPVVDGIVVTDIDTMTIDPQVEKKMGQVLLIAVAVSSAILSRTAWG      2460
103344_1            ARAAQKRTAAGIMKNPVVDGIVVTDIDTMTIDPQVEKKMGQVLLIAVAVSSAILSRTAWG      2460
GD01_1              ARAAQKRTAAGIMKNPVVDGIVVTDIDTMTIDPQVEKKMGQVLLIAVAVSSAILSRTAWG      2460
SPH2015_1           ARAAQKRTAAGIMKNPVVDGIVVTDIDTMTIDPQVEKKMGQVLLMAVAVSSAILSRTAWG      2460
Haiti2014_1         ARAAQKRTAAGIMKNPVVDGIVVTDIDTMTIDPQVEKKMGQVLLMAVAVSSAILSRTAWG      2460
PLCal_ZV_1          ARAAQKRTAAGIMKNPVVDGIVVTDIDTMTIDPQVEKKMGQVLLIAVAVSSAILSRTAWG      2423
Martinique          ARAAQKRTAAGIMKNPVVDGIVVTDIDTMTIDPQVEKKMGQVLLIAVAVSSAILSRTAWG      2460
NatalRGN            ARAAQKRTAAGIMKNPVVDGIVVTDIDTMTIDPQVEKKMGQVLLIAVAVSSAILSRTAWG      2460
BrasilZKV2015_1     ARAAQKRTAAGIMKNPVVDGIVVTDIDTMTIDPQVEKKMGQVLLIAVAVSSAILSRTAWG      2460
Z1106033_1          ARAAQKRTAAGIMKNPVVDGIVVTDIDTMTIDPQVEKKMGQVLLIAVAVSSAILSRTAWG      2460
PRVABC59_1          ARAAQKRTAAGIMKNPVVDGIVVTDIDTMTIDPQVEKKMGQVLLIAVAVSSAILSRTAWG      2460
HPF2013_1           ARAAQKRTAAGIMKNPVVDGIVVTDIDTMTIDPQVEKKMGQVLLIAVAVSSAILSRTAWG      2460
Beh815744_1         ARAAQKRTAAGIMKNPVVDGIVVTDIDTMTIDPQVEKKMGQVLLIAVAVSSAILSRTAWG      2460
BEH818995_1         ARAAQKRTAAGIMKNPVVDGIVVTDIDTMTIDPQVEKKMGQVLLIAVAVSSAILSRTAWG      2460
BEH819966_1         ARAAQKRTAAGIMKNPVVDGIVVTDIDTMTIDPQVEKKMGQVLLIAVAVSSAILSRTAWG      2460
BeH819015_1         ARAAQKRTAAGIMKNPVVDGIVVTDIDTMTIDPQVEKKMGQVLLIAVAVSSAILSRTAWG      2460
SSABr_1             ARAAQKRTAAGIMKNPVVDGIVVTDIDTMTIDPQVEKKMGQVLLIAVAVSSAILSRTAWG      2460
ARD157995_1         ARAAQKRTAAGIMKNPVVDGIVVTDIDTMTIDPQVEKKMGQVLLIAVAISSAVLLRTAWG      2460
MR_766_1            ARAAQKRTAAGIMKNPVVDGIVVTDIDTMTIDPQVEKKMGQVLLIAVAISSAVLLRTAWG      2456
ARD158084_1         ARAAQKRTAAGIMKNPVVDGIVVTDIDTMTIDPQVEKKMGQVLLIAVAISSAVLLRTAWG      2460
ArB1362_1           ARAAQKRTAAGIMKNPVVDGIVVTDIDTMTIDPQVEKKMGQVLLIAVAISSAVLLRTAWG      2460
ARB15076_1          ARAAQKRTAAGIMKNPVVDGIVVTDIDTMTIDPQVEKKMGQVLLIAVAISSAVLLRTAWG      2454
ARB13565_1          ARAAQKRTAAGIMKNPVVDGIVVTDIDTMTIDPQVEKKMGQVLLIAVAISSAVLLRTAWG      2460
ARB7701_1           ARAAQKRTAAGIMKNPVVDGIVVTDIDTMTIDPQVEKKMGQVLLIAVAISSAVLLRTAWG      2460
IbH30656_1          ARAAQKRTAAGIMKNPVVDGIVVTDIDTMTIDPQVEKKMGQVLLIAVAASSAVLLRTAWG      2454
ArD128000_1         ARAAQKRTAAGIMKNPVVDGIVVTDIDTMTIDPQVEKKMGQVLLIAVAVSSAVLLRTAGG      2460
ArD7117_1           ARAAQKRTAAGIMKNPVVDGIVVTDIDTMTIDPQVEKKMGQVLLIAVAVSSAVLLRTAWG      2460
ARD_41519_1         ARAAQKRTAAGIMKNPVVDGIVVTDIDTMTIDPQVEKKMGQVLLIAVAVSSAVLLRTAWG      2460
                    ******************************** :**********:*** ***:* *** *

P6-740_1            WGEAGALITAATSTLWEGSPNKYWNSSTATSLCNIFRGSYLAGASLIYTVTRNAGLVKRR      2520
P6740               WGEAGALITAATSTLWEGSPNKYWNSSTATSLCNIFRGSYLAGASLIYTVTRNAGLVKRR      2520
Yap2007             WGEAGALITAATSTLWEGSPNKYWNSSTATSLCNIFRGSYLAGASLIYTVTRNAGLVKRR      2520
CPC0740_1           WGEAGALITAATSTLWEGSPNKYWNSSTATSLCNIFRGSYLAGASLIYTVTRNAGLVKRR      2520
SV0127/14_1         WGEAGALITAATSTLWEGSPNKYWNSSTATSLCNIFRGSYLAGASLIYTVTRNAGLVKRR      2520
FSS13025_1          WGEAGALITAATSTLWEGSPNKYWNSSTATSLCNIFRGSYLAGASLIYTVTRNAGLVKRR      2520
8375_1              WGEAGALITAATSTLWEGSPNKYWNSSTATSLCNIFRGSYLAGASLIYTVTRNAGLVKRR      2520
103344_1            WGEAGALITAATSTLWEGSPNKYWNSSTATSLCNIFRGSYLAGASLIYTVTRNAGLVKRR      2520
GD01_1              WGEAGALITAATSTLWEGSPNKYWNSSTATSLCNIFRGSYLAGASLIYTVTRNAGLVKRR      2520
SPH2015_1           WGEAGALITAATSTLWEGSPNKYWNSSTATSLCNIFRGSYLAGASLIYTVTRNAGLVKRR      2520
Haiti2014_1         WGEAGALITAATSTLWEGSPNKYWNSSTATSLCNIFRGSYLAGASLIYTVTRNAGLVKRR      2520
PLCal_ZV_1          WGEAGALITAATSTLWEGSPNKYWNSSTATSLCNIFRGSYLAGASLIYTVTRNAGLVKRR      2483
Martinique          WGEAGALITAATSTLWEGSPNKYWNSSTATSLCNIFRGSYLAGASLIYTVTRNAGLVKRR      2520
NatalRGN            WGEAGALITAATSTLWEGSPNKYWNSSTATSLCNIFRGSYLAGASLIYIVTRNAGLVKRR      2520
BrasilZKV2015_1     WGEAGALITAATSTLWEGSPNKYWNSSTATSLCNIFRGSYLAGASLIYTVTRNAGLVKRR      2520
```

```
Z1106033_1          WGEAGALITAATSTLWEGSPNKYWNSSTATSLCNIFRGSYLAGASLIYTVTRNAGLVKRR      2520
PRVABC59_1          WGEAGALITAATSTLWEGSPNKYWNSSTATSLCNIFRGSYLAGASLIYTVTRNAGLVKRR      2520
HPF2013_1           WGEAGALITAATSTLWEGSPNKYWNSSTATSLCNIFRGSYLAGASLIYTVTRNAGLVKRR      2520
Beh815744_1         WGEAGALITAATSTLWEGSPNKYWNSSTATSLCNIFRGSYLAGASLIYTVTRNAGLVKRR      2520
BEH818995_1         WGEAGALITAATSTLWEGSPNKYWNSSTATSLCNIFRGSYLAGASLIYTVTRNAGLVKRR      2520
BEH819966_1         WGEAGALITAATSTLWEGSPNKYWNSSTATSLCNIFRGSYLAGASLIYTVTRNAGLVKRR      2520
BeH819015_1         WGEAGALITAATSTLWEGSPNKYWNSSTATSLCNIFRGSYLAGASLIYTVTRNAGLVKRR      2520
SSABr_1             WGEAGALITAATSTLWEGSPNKYWNSSTATSLCNIFRGSYLAGASLIYTVTRNAGLVKRR      2520
ARD157995_1         WGEAGALITAATSTLWEGSPNKYWNSSTATSLCNIFRGSYLAGASLIYTVTRNAGLVKRR      2520
MR_766_1            WGEAGALITAATSTLWEGSPNKYWNSSTATSLCNIFRGSYLAGASLIYTVTRNAGLVKRR      2516
ARD158084_1         WGEAGALITAATSTLWEGSPNKYWNSSTATSLCNIFRGSYLAGASLIYTVTRNAGLVKRR      2520
ArB1362_1           WGEAGALITAATSTLWEGSPNKYWNSSTATSLCNIFRGSYLAGASLIYTVTRNAGLVKRR      2520
ARB15076_1          WGEAGALITAATSTLWEGSPNKYWNSSTATSLCNIFRGSYLAGASLIYTVTRNAGLVKRR      2514
ARB13565_1          WGEAGALITAATSTLWEGSPNKYWNSSTATSLCNIFRGSYLAGASLIYTVTRNAGLVKRR      2520
ARB7701_1           WGEAGALITAATSTLWEGSPNKYWNSSTATSLCNIFRGSYLAGASLIYTVTRNAGLVKRR      2520
IbH30656_1          WGEAGALITAATSTLWEGSPNKYWNSSTATSLCNIFRGSYLAGASLIYTVTRNAGLVKRR      2514
ArD128000_1         WGEAGALITAATSTLWEGSPNKYWNSSTATSLCNIFRGSYLAGASLIYTVTRNAGLVKRR      2520
ArD7117_1           WGEAGALITAATSTLWEGSPNKYWNSSTATSLCNIFRGSYLAGASLIYTVTRNAGLVKRR      2520
ARD_41519_1         WGEAGALITAATSTLWEGSPNKYWNSSTATSLCNIFRGSYLAGASLIYTVTRNAGLVKRR      2520
                    ************************************************ ***********

P6-740_1            GGGTGETLGEKWKARLNQMSALEFYSYKKSGITEVCREEARRALKDGVATGGHAVSRGSA      2580
P6740               GGGTGETLGEKWKARLNQMSALEFYSYKKSGITEVCREEARRALKDGVATGGHAVSRGSA      2580
Yap2007             GGGTGETLGEKWKARLNQMSALEFYSYKKSGITEVCREEARRALKDGVATGGHAVSRGSA      2580
CPC0740_1           GGGTGETLGEKWKARLNQMSALEFYSYKKSGITEVCREEARRALKDGVATGGHAVSRGSA      2580
SV0127/14_1         GGGTGETLGEKWKARLNQMSALEFYSYKKSGITEVCREEARRALKDGVATGGHAVSRGSA      2580
FSS13025_1          GGGTGETLGEKWKARLNQMSALEFYSYKKSGITEVCREEARRALKDGVATGGHAVSRGSA      2580
8375_1              GGGTGETLGEKWKARLNQMSALEFYSYKKSGITEVCREEARRALKDGVATGGHAVSRGSA      2580
103344_1            GGGTGETLGEKWKARLNQMSALEFYSYKKSGITEVCREEARRALKDGVATGGHAVSRGSA      2580
GD01_1              GGGTGETLGEKWKARLNQMSALEFYSYKKSGITEVCREEARRALKDGVATGGHAVSRGSA      2580
SPH2015_1           GGGTGETLGEKWKARLNQMSALEFYSYKKSGITEVCREEARRALKDGVATGGHAVSRGSA      2580
Haiti2014_1         GGGTGETLGEKWKARLNQMSALEFYSYKKSGITEVCREEARRALKDGVATGGHAVSRGSA      2580
PLCal_ZV_1          GGGTGETLGEKWKARLNQMSALEFYSYKKSGITEVCREEARRALKDGVATGGHAVSRGSA      2543
Martinique          GGGTGETLGEKWKARLNQMSALEFYSYKKSGITEVCREEARRALKDGVATGGHAVSRGSA      2580
NatalRGN            GGGTGETLGEKWKARLNQMSALEFYSYKKSGITEVCREEARRALKDGVATGGHAVSRGSA      2580
BrasilZKV2015_1     GGGTGETLGEKWKARLNQMSALEFYSYKKSGITEVCREEARRALKDGVATGGHAVSRGSA      2580
Z1106033_1          GGGTGETLGEKWKARLNQMSALEFYSYKKSGITEVCREEARRALKDGVATGGHAVSRGSA      2580
PRVABC59_1          GGGTGETLGEKWKARLNQMSALEFYSYKKSGITEVCREEARRALKDGVATGGHAVSRGSA      2580
HPF2013_1           GGGTGETLGEKWKARLNQMSALEFYSYKKSGITEVCREEARRALKDGVATGGHAVSRGSA      2580
Beh815744_1         GGGTGETLGEKWKARLNQMSALEFYSYKKSGITEVCREEARRALKDGVATGGHAVSRGSA      2580
BEH818995_1         GGGTGETLGEKWKARLNQMSALEFYSYKKSGITEVCREEARRALKDGVATGGHAVSRGSA      2580
BEH819966_1         GGGTGETLGEKWKARLNQMSALEFYSYKKSGITEVCREEARRALKDGVATGGHAVSRGSA      2580
BeH819015_1         GGGTGETLGEKWKARLNQMSALEFYSYKKSGITEVCREEARRALKDGVATGGHAVSRGSA      2580
SSABr_1             GGGTGETLGEKWKARLNQMSALEFYSYKKSGITEVCREEARRALKDGVATGGHAVSRGSA      2580
ARD157995_1         GGGTGETLGEKWKARLNQMSALEFYSYKKSGITEVCREEARRALKDGVATGGHAVSRGSA      2580
MR_766_1            GGGTGETLGEKWKARLNQMSALEFYSYKKSGITEVCREEARRALKDGVATGGHAVSRGSA      2576
ARD158084_1         GGGTGETLGEKWKARLNQMSALEFYSYKKSGITEVCREEARRALKDGVATGGHAVSRGSA      2580
ArB1362_1           GGGTGETLGEKWKARLNQMSALEFYSYKKSGITEVCREEARRALKDGVATGGHAVSRGSA      2580
ARB15076_1          GGGTGETLGEKWKARLNQMSALEFYSYKKSGITEVCREEARRALKNGVATGGHAVSRGSA      2574
ARB13565_1          GGGTGETLGEKWKARLNQMSALEFYSYKKSGITEVCREEARRALKDGVATGGHAVSRGSA      2580
ARB7701_1           GGGTGETLGEKWKARLNQMSALEFYSYKKSGITEVCREEARRALKDGVATGGHAVSRGSA      2580
IbH30656_1          GGGTGETLGEKWKARLNQMSALEFYSYKKSGITEVCREEARRALKDGVATGGHAVSRGSA      2574
ArD128000_1         GGGTGETLGEKWKARLNQMSALEFYSYKKSGITEVCREEARRALKDGVATGGHAVSRGSA      2580
ArD7117_1           GGGTGETLGEKWKARLNQMSALEFYSYKKSGITEVCREEARRALKDGVATGGHAVSRGSA      2580
ARD_41519_1         GGGTGETLGEKWKARLNQMSALEFYSYKKSGITEVCREEARRALKDGVATGGHAVSRGSA      2580
                    *********************************************:**************

P6-740_1            KLRWLVERGYLQPYGKVIDLGCGRGGWSYYAATIRKVQEVKGYTKGGPGHEEPTLVQSYG      2640
P6740               KLRWLVERGYLQPYGKVIDLGCGRGGWSYYAATIRKVQEVKGYTKGGPGHEEPTLVQSYG      2640
Yap2007             KLRWLVERGYLQPYGKVIDLGCGRGGWSYYAATIRKVQEVKGYTKGGPGHEEPMLVQSYG      2640
CPC0740_1           KLRWLVERGYLQPYGKVIDLGCGRGGWSYYAATIRKVQEVKGYTKGGPGHEEPMLVQSYG      2640
SV0127/14_1         KLRWLVERGYLQPYGKVIDLGCGRGGWSYYAATIRKVQEVKGYTKGGPGHEEPMLVQSYG      2640
FSS13025_1          KLRWLVERGYLQPYGKVIDLGCGRGGWSYYAATIRKVQEVKGYTKGGPGHEEPMLVQSYG      2640
8375_1              KLRWLVERGYLQPYGKVIDLGCGRGGWSYYAATIRKVQEVKGYTKGGPGHEEPVLVQSYG      2640
103344_1            KLRWLVERGYLQPYGKVIDLGCGRGGWSYYAATIRKVQEVKGYTKGGPGHEEPVLVQSYG      2640
GD01_1              KLRWLVERGYLQPYGKVIDLGCGRGGWSYYAATIRKVQEVKGYTKGGPGHEEPVLVQSYG      2640
SPH2015_1           KLRWLVERGYLQPYGKVIDLGCGRGGWSYYAATIRKVQEVKGYTKGGPGHEEPVLVQSYG      2640
Haiti2014_1         KLRWLVERGYLQPYGKVIDLGCGRGGWSYYAATIRKVQEVKGYTKGGPGHEEPVLVQSYG      2640
```

```
PLCal_ZV_1          KLRWLVERGYLQPYGKVIDLGCGRGGWSYYAATIRKVQEVKGYTKGGPGHEEPMLVQSYG      2603
Martinique          KLRWLVERGYLQPYGKVIDLGCGRGGWSYYAATIRKVQEVKGYTKGGPGHEEPVLVQSYG      2640
NatalRGN            KLRWLVERGYLQPYGKVIDLGCGRGGWSYYAATIRKVQEVKGYTKGGPGHEEPVLVQSYG      2640
BrasilZKV2015_1     KLRWLVERGYLQPYGKVIDLGCGRGGWSYYAATIRKVQEVKGYTKGGPGHEEPVLVQSYG      2640
Z1106033_1          KLRWLVERGYLQPYGKVIDLGCGRGGWSYYAATIRKVQEVKGYTKGGPGHEEPVLVQSYG      2640
PRVABC59_1          KLRWLVERGYLQPYGKVIDLGCGRGGWSYYVATIRKVQEVKGYTKGGPGHEEPVLVQSYG      2640
HPF2013_1           KLRWLVERGYLQPYGKVIDLGCGRGGWSYYAATIRKVQEVKGYTKGGPGHEEPMLVQSYG      2640
Beh815744_1         KLRWLVERGYLQPYGKVIDLGCGRGGWSYYAATIRKVQEVKGYTKGGPGHEEPVLVQSYG      2640
BEH818995_1         KLRWLVERGYLQPYGKVIDLGCGRGGWSYYAATIRKVQEVKGYTKGGPGHEEPVLVQSYG      2640
BEH819966_1         KLRWLVERGYLQPYGKVIDLGCGRGGWSYYAATIRKVQEVKGYTKGGPGHEEPVLVQSYG      2640
BeH819015_1         KLRWLVERGYLQPYGKVIDLGCGRGGWSYYAATIRKVQEVKGYTKGGPGHEEPVLVQSYG      2640
SSABr_1             KLRWLVERGYLQPYGKVIDLGCGRGGWSYYAATIRKVQEVKGYTKGGPGHEEPVLVQSYG      2640
ARD157995_1         KLRWLVERGYLQPHGKVVDLGCGRGGWSYYAATIRKVQEVRGYTKGGPGHEEPMLVQSYG      2640
MR_766_1            KIRWLEERGYLQPYGKVVDLGCGRGGWSYYAATIRKVQEVRGYTKGGPGHEEPMLVQSYG      2636
ARD158084_1         KLRWLVERGYLQPHGKVVDLGCGRGGWSYYAATIRKVQEVRGYTKGGPGHEEPMLVQSYG      2640
ArB1362_1           KLRWLVERGYLQPHGKVVDLGCGRGGWSYYAATIRKVQEVKGYTKGGPGHEEPMLVQSYG      2640
ARB15076_1          KLRWLVERGYLQPHGKVVDLGCGRGGWSYYAATIRKVQEVKGYTKGGPGHEEPMLVQSYG      2634
ARB13565_1          KLRWLVERGYLQPHGKVVDLGCGRGGWSYYAATIRKVQEVKGYTKGGPGHEEPMLVQSYG      2640
ARB7701_1           KLRWLVERGYLQPHGKVVDLGCGRGGWSYYAATIRKVQEVKGYTKGGPGHEEPMLVQSYG      2640
IbH30656_1          KLRWLVERGYLQPHGKVVDLGCGRGGWSYYAATIRKVQEVRGYTKGGPGHEEPMLVQSYG      2634
ArD128000_1         KLRWLVERGYLQPHGKVVDLGCGRGGWSYYAATIRKVQEVRGYTKGGPGHEEPMLVQSYG      2640
ArD7117_1           KLRWLVERGYLQPHGKVVDLGCGRGGWSYYAATIRKVQEVRGYTKGGPGHEEPMLVQSYG      2640
ARD_41519_1         KLRWLVERGYLQPHGKVVDLGCGRGGWSYYAATIRKVQEVRGYTKGGPGHEEPMLVQSYG      2640
                    *:*** *******:***:************.*********:************ ******

P6-740_1            WNIVRLKSGVDVFHMAAESCDTLLCDIGESSSSPEVEEARTLRVLSMVGDWLEKRPGAFC      2700
P6740               WNIVRLKSGVDVFHMAAESCDTLLCDIGESSSSPEVEEARTLRVLSMVGDWLEKRPGAFC      2700
Yap2007             WNIVRLKSGVDVFHMAAEPCDTLLCDIGESSSSPEVEEARTLRVLSMVGDWLEKRPGAFC      2700
CPC0740_1           WNIVRLKSGVDVFHMAAEPCDTLLCDIGESSSSPEVEEARTLRVLSMVGDWLEKRPGAFC      2700
SV0127/14_1         WNIVRLKSGVDVFHMAAEPCDTLLCDIGESSSSPEVEEARTLRVLSMVGDWLEKRPGAFC      2700
FSS13025_1          WNIVRLKSGVDVFHMAAEPCDTLLCDIGESSSSPEVEEARTLRVLSMVGDWLEKRPGAFC      2700
8375_1              WNIVRLKSGVDVFHMAAEPCDTLLCDIGESSSSPEVEEARTLRVLSMVGDWLERRPGAFC      2700
103344_1            WNIVRLKSGVDVFHMAAEPCDTLLCDIGESSSSPEVEEARTLRVLSMVGDWLERRPGAFC      2700
GD01_1              WNIVRLKSGVDVFHMAAEPCDTLLCDIGESSSSPEVEEARTLRVLSMVGDWLEKRPGAFC      2700
SPH2015_1           WNIVRLKSGVDVFHMAAEPCDTLLCDIGESSSSPEVEEARTLRVLSMVGDWLEKRPGAFC      2700
Haiti2014_1         WNIVRLKSGVDVFHMAAEPCDTLLCDIGESSSSPEVEEARTLRVLSMVGDWLEKRPGAFC      2700
PLCal_ZV_1          WNIVRLKSGVDVFHMAAEPCDTLLCDIGESSSSPEVEEARTLRVLSMVGDWLEKRPGAFC      2663
Martinique          WNIVRLKSGVDVFHMAAEPCDTLLCDIGESSSSPEVEEARTLRVLSMVGDWLEKRPGAFC      2700
NatalRGN            WNIVRLKSGVDVFHMAAEPCDTLLCDIGESSSSPEVEEARTLRVLSMVGDWLEKRPGAFC      2700
BrasilZKV2015_1     WNIVRLKSGVDVFHMAAEPCDTLLCDIGESSSSPEVEEARTLRVLSMVGDWLEKRPGAFC      2700
Z1106033_1          WNIVRLKSGVDVFHMAAEPCDTLLCDIGESSSSPEVEEARTLRVLSMVGDWLEKRPGAFC      2700
PRVABC59_1          WNIVRLKSGVDVFHMAAEPCDTLLCDIGESSSSPEVEEARTLRVLSMVGDWLEKRPGAFC      2700
HPF2013_1           WNIVRLKSGVDVFHMAAEPCDTLLCDIGESSSSPEVEEARTLRVLSMVGDWLEKRPGAFC      2700
Beh815744_1         WNIVRLKSGVDVFHMAAEPCDTLLCDIGESSSSPEVEEARTLRVLSMVGDWLEKRPGAFC      2700
BEH818995_1         WNIVRLKSGVDVFHMAAEPCDTLLCDIGESSSSPEVEEARTLRVLSMVGDWLEKRPGAFC      2700
BEH819966_1         WNIVRLKSGVDVFHMAAEPCDTLLCDIGESSSSPEVEEARTLRVLSMVGDWLEKRPGAFC      2700
BeH819015_1         WNIVRLKSGVDVFHMAAEPCDTLLCDIGESSSSPEVEEARTLRVLSMVGDWLEKRPGAFC      2700
SSABr_1             WNIVRLKSGVDVFHMAAEPCDTLLCDIGESSSSPEVEEARTLRVLSMVGDWLEKRPGAFC      2700
ARD157995_1         WNIVRLKSGVDVFHMAAEPCDTLLCDIGESSSSPEVEETRTLRVLSMVGDWLEKRPGAFC      2700
MR_766_1            WNIVRLKSGVDVFHMAAEPCDTLLCDIGESSSSPEVEETRTLRVLSMVGDWLEKRPGAFC      2696
ARD158084_1         WNIVRLKSGVDVFHMAAEPCDTLLCDIGESSSSPEVEETRTLRVLSMVGDWLEKRPGAFC      2700
ArB1362_1           WNIVRLKSGVDVFHMAAEPCDTLLCDIGESSSSPEVEETRTLRVLSMVGDWLEKRPGAFC      2700
ARB15076_1          WNIVRLKSGVDVFHMAAEPCDTLLCDIGESSSSPEVEETRTLRVLSMVGDWLEKRPGAFC      2694
ARB13565_1          WNIVRLKSGVDVFHMAAEPCDTLLCDIGESSSSPEVEETRTLRVLSMVGDWLEKRPGAFC      2700
ARB7701_1           WNIVRLKSGVDVFHMAAEPCDTLLCDIGESSSSPEVEETRTLRVLSMVGDWLEKRPGAFC      2700
IbH30656_1          WNIVRLKSGVDVFHMAAEPCDTLLCDIGESSSSPEVEETRTLRVLSMVGDWLEKRPGAFC      2694
ArD128000_1         WNIVRLKSGVDVFHMAAEPCDTLLCDIGESSSSPEVEETRTLRVLSMVEDWLEKRPGAFC      2700
ArD7117_1           WNIVRLKSGVDVFHMAAEPCDTLLCDIGESSSSPEVEETRTLRVLSMVGDWLEKRPGAFC      2700
ARD_41519_1         WNIVRLKSGVDVFHMAAEPCDTLLCDIGESSSSPEVEETRTLRVLSMVGDWLEKRPGAFC      2700
                    ****************** *******************:********* ****:******

P6-740_1            IKVLCPYTSTMMETLERLQRRYGGGLVRVPLSRNSTHEMYWVSGAKSNIIKSVSTTSQLL      2760
P6740               IKVLCPYTSTMMETLERLQRRYGGGLVRVPLSRNSTHEMYWVSGAKSNIIKSVSTTSQLL      2760
Yap2007             IKVLCPYTSTMMETLERLQRRYGGGLVRVPLSRNSTHEMYWVSGAKSNTIKSVSTTSQLL      2760
CPC0740_1           IKVLCPYTSTMMETLERLQRRYGGGLVRVPLSRNSTHEMYWVSGAKSNTIKSVSTTSQLL      2760
SV0127/14_1         VKVLCPYTSTMMETLERLQRRYGGGLVRVPLSRNSTHEMYWVSGAKSNTIKSVSTTSQLL      2760
FSS13025_1          IKVLCPYTSTMMETLERLQRRYGGGLVRVPLSRNSTHEMYWVSGAKSNTIKSVSTTSQLL      2760
8375_1              IKVLCPYTSTMMETLERLQRRYGGGLVRVPLSRNSTHEMYWVSGAKSNTIKSVSTTSQLL      2760
```

```
103344_1            IKVLCPYTSTMMETLERLQRRYGGGLVRVPLSRNSTHEMYWVSGAKSNTIKSVSTTSQLL    2760
GD01_1              IKVLCPYTSTMMETLERLQRRYGGGLVRVPLSRNSTHEMYWVSGAKSNIIKSVSTTSQLL    2760
SPH2015_1           IKVLCPYTSTMMETLERLQRRYGGGLVRVPLSRNSTHEMYWVSGAKSNTIKSVSTTSQLL    2760
Haiti2014_1         IKVLCPYTSTMMETLERLQRRYGGGLVRVPLSRNSTHEMYWVSGAKSNTIKSVSTTSQLL    2760
PLCal_ZV_1          IKVLCPYTSTMMETLERLQRRYGGGLVRVPLSRNSTHEMYWVSGAKSNTIKSVSTTSQLL    2723
Martinique          IKVLCPYTSTMMETLERLQRRYGGGLVRVPLSRNSTHEMYWVSGAKSNTIKSVSTTSQLL    2760
NatalRGN            IKVLCPYTSTMMETLERLQRRYGGGLVRVPLSRNSTHEMYWVSGAKSNTIKSVSTTSQLL    2760
BrasilZKV2015_1     IKVLCPYTSTMMETLERLQRRYGGGLVRVPLSRNSTHEMYWVSGAKSNTIKSVSTTSQLL    2760
Z1106033_1          IKVLCPYTSTMMETLERLQRRYGGGLVRVPLSRNSTHEMYWVSGAKSNTIKSVSTTSQLL    2760
PRVABC59_1          IKVLCPYTSTMMETLERLQRRYGGGLVRVPLSRNSTHEMYWVSGAKSNTIKSVSTTSQLL    2760
HPF2013_1           IKVLCPYTSTMMETLERLQRRYGGGLVRVPLSRNSTHEMYWVSGAKSNTIKSVSTTSQLL    2760
Beh815744_1         IKVLCPYTSTMMETLERLQRRYGGGLVRVPLSRNSTHEMYWVSGAKSNTIKSVSTTSQLL    2760
BEH818995_1         IKVLCPYTSTMMETLERLQRRYGGGLVRVPLSRNSTHEMYWVSGAKSNTIKSVSTTSQLL    2760
BEH819966_1         IKVLCPYTSTMMETLERLQRRYGGGLVRVPLSRNSTHEMYWVSGAKSNTIKSVSTTSQLL    2760
BeH819015_1         IKVLCPYTSTMMETLERLQRRYGGGLVRVPLSRNSTHEMYWVSGAKSNTIKSVSTTSQLL    2760
SSABr_1             IKVLCPYTSTMMETLERLQRRYGGGLVRVPLSRNSTHEMYWVSGAKSNTIKSVSTTSQLL    2760
ARD157995_1         IKVLCPYTSTMMETMERLQRRHGGGLVRVPLSRNSTHEMYWVSGAKSNIIKSVSTTSQLL    2760
MR_766_1            IKVLCPYTSTMMETMERLQRRHGGGLVRVPLCRNSTHEMYWVSGAKSNIIKSVSTTSQLL    2756
ARD158084_1         IKVLCPYTSTMMETMERLQRRHGGGLVRVPLSRNSTHEMYWVSGAKSNIIKSVSTTSQLL    2760
ArB1362_1           IKVLCPYTSTMMETMERLQRRYGGGLVRVPLSRNSTHEMYWVSGAKSNIIKSVSTTSQLL    2760
ARB15076_1          IKVLCPYTSTMMETMERLQRRYGGGLVRVPLSRNSTHEMYWVSGAKSNIIKSVSTTSQLL    2754
ARB13565_1          IKVLCPYTSTMMETMERLQRRYGGGLVRVPLSRNSTHEMYWVSGAKSNVIKSVSTTSQLL    2760
ARB7701_1           IKVLCPYTSTMMETMERLQRRYGGGLVRVPLSRNSTHEMYWVSGAKSNVIKSVSTTSQLL    2760
IbH30656_1          IKVLCPYTSTMMETMERLQRRYGGGLVRVPLSRNSTHEMYWVSGAKSNIIKSVSTTSQLL    2754
ArD128000_1         IKVLCPYTSTMMETMERLQRRHGGGLVRVPLSRNSTHEMYWVSGAKSNIIKSVSTTSQLL    2760
ArD7117_1           IKVLCPYTSTMMETMERLQRRHGGGLVRVPLSRNSTHEMYWVSGAKSNIIKSVSTTSQLL    2760
ARD_41519_1         IKVLCPYTSTMMETMERLQRRHGGGLVRVPLSRNSTHEMYWVSGAKSNIIKSVSTTSQLL    2760
                    :*************:******:*********.**************** ***********

P6-740_1            LGRMDGPRRPVKYEEDVNLGSGTRAVASCAEAPNLKIIGNRVERIRSEHAETWFFDENHP    2820
P6740               LGRMDGPRRPVKYEEDVNLGSGTRAVASCAEAPNLKIIGNRVERIRSEHAETWFFDENHP    2820
Yap2007             LGRMDGPRRPVKYEEDVNLGSGTRAVVSCAEAPNMKIIGNRIERIRSEHAETWFFDENHP    2820
CPC0740_1           LGRMDGPRRPVKYEEDVNLGSGTRAVVSCAEAPNMKIIGNRIERIRSEHAETWFFDENHP    2820
SV0127/14_1         LGRMDGPRRPVKYEEDVNLGSGTRAVVSCAEAPNMKIIGNRIERIRSEHAETWFFDENHP    2820
FSS13025_1          LGRMDGPRRPVKYEEDVNLGSGTRAVVSCAEAPNMKIIGNRIERIRSEHAETWFFDENHP    2820
8375_1              LGRMDGPRRPVKYEEDVNLGSGTRAVVSCAEAPNMKIIGNRIERIRSEHAETWFFDENHP    2820
103344_1            LGRMDGPRRPVKYEEDVNLGSGTRAVVSCAEAPNMKIIGNRIERIRSEHAETWFFDENHP    2820
GD01_1              LGRMDGPRRPVKYEEDVNLGSGTRAVASCAEAPNMKIIGNRIERIRSEHAETWFFDENHP    2820
SPH2015_1           LGRMDGPRRPVKYEEDVNLGSGTRAVVSCAEAPNMKIIGNRIERIRSEHAETWFFDENHP    2820
Haiti2014_1         LGRMDGPRRPVKYEEDVNLGSGTRAVVSCAEAPNMKIIGNRIERIRSEHAETWFFDENHP    2820
PLCal_ZV_1          LGRMDGPRRPVKYEEDVNLGSGTRAVVSCAEAPNMKIIGNRIERIRSEHAETWFFDENHP    2783
Martinique          LGRMDGPRRPVKYEEDVNLGSGTRAVVSCAEAPNMKIIGNRIERIRSEHAETWFFDENHP    2820
NatalRGN            LGRMDGPRRPVKYEEDVNLGSGTRAVVSCAEAPNMKIIGNRIERIRSEHAETWFFDENHP    2820
BrasilZKV2015_1     LGRMDGPRRPVKYEEDVNLGSGTRAVVSCAEAPNMKIIGNRIERIRSEHAETWFFDENHP    2820
Z1106033_1          LGRMDGPRRPVKYEEDVNLGSGTRAVVSCAEAPNMKIIGNRIERIRSEHAETWFFDENHP    2820
PRVABC59_1          LGRMDGPRRPVKYEEDVNLGSGTRAVVSCAEAPNMKIIGNRIERIRSEHAETWFFDENHP    2820
HPF2013_1           LGRMDGPRRPVKYEEDVNLGSGTRAVVSCAEAPNMKIIGNRIERIRSEHAETWFFDENHP    2820
Beh815744_1         LGRMDGPRRPVKYEEDVDLGSGTRAVVSCAEAPNMKIIGNRIERIRSEHAETWFFDENHP    2820
BEH818995_1         LGRMDGPRRPVKYEEDVDLGSGTRAVVSCAEAPNMKIIGNRIERIRSEHAETWFFDENHP    2820
BEH819966_1         LGRMDGPRRPVKYEEDVNLGSGTRAVVSCAEAPNMKIIGNRIERIRSEHAETWFFDENHP    2820
BeH819015_1         LGRMDGPRRPVKYEEDVNLGSGTRAVVSCAEAPNMKIIGNRIERIRSEHAETWFFDENHP    2820
SSABr_1             LGRMDGPRRPVKYEEDVNLGSGTRAVVSCAEAPNMKIIGNRIERIRSEHAETWFFDENHP    2820
ARD157995_1         LGRMDGPRRPVKYEEDVNLGSGTRAVASCAEAPNMKIIGRRIERIRNEHAETWFLDENHP    2820
MR_766_1            LGRMDGPRRPVKYEEDVNLGSGTRAVASCAEAPNMKIIGRRIERIRNEHAETWFLDENHP    2816
ARD158084_1         LGRMDGPRRPVKYEEDVNLGSGTRAVASCAEAPNMKIIGRRIERIRNEHAETWFLDENHP    2820
ArB1362_1           LGRMDGPRRPVKYEEDVNLGSGTRAVASCAEAPNMKIIGKRIERIRSEHAETWFFDENHP    2820
ARB15076_1          LGRMDGPRRPVKYEEDVNLGSGTRAVASCAEAPNMKIIGKRIERIRSEHAETWFFDENHP    2814
ARB13565_1          LGRMDGPRRPVKYEEDVNLGSGTRAVASCAEAPNMKVIGKRIERIRSEHAETWFFDENHP    2820
ARB7701_1           LGRMDGPRRPVKYEEDVNLGSGTRAVASCAEAPNMKVIGKRIERIRSEHAETWFFDENHP    2820
IbH30656_1          LGRMDGPRRPVKYEEDVNLGSGTRAVASCAEAPNMKIIGRRIERIRNEHAETWFFDENHP    2814
ArD128000_1         LGRMDGPRRPVKYEEDVNLGSGTRAVASCAEAPNMKIIGRRIERIRNEHAETWFFDENHP    2820
ArD7117_1           LGRMDGPRRPVKYEEDVNLGSGTRAVASCAEAPNMKIIGRRIERIRNEHAETWFFDENHP    2820
ARD_41519_1         LGRMEGPRRPVKYEEDVNLGSGTRAVASCAEAPNMKIIGRRIERIRNEHAETWFFDENHP    2820
                    ****:************:********.*******:*:**.*:****.*******:*****

P6-740_1            YRTWAYHGSYEAPTQGSASSLINGVVRLLSKPWDVVTGVTGIAMTDTTPYGQQRVFKEKV    2880
P6740               YRTWAYHGSYEAPTQGSASSLINGVVRLLSKPWDVVTGVTGIAMTDTTPYGQQRVFKEKV    2880
Yap2007             YRTWAYHGSYEAPTQGSASSLINGVVRLLSKPWDVVTGVTGIAMTDTTPYGQQRVFKEKV    2880
```

```
CPC0740_1           YRTWAYHGSYEAPTQGSASSLINGVVRLLSKPWDVVTGVTGIAMTDTTPYGQQRVFKEKV    2880
SV0127/14_1         YRTWAYHGSYEAPTQGSASSLINGVVRLLSKPWDVVTGVTGIAMTDTTPYGQQRVFKEKV    2880
FSS13025_1          YRTWAYHGSYEAPTQGSASSLINGVVRLLSKPWDVVTGVTGIAMTDTTPYGQQRVFKEKV    2880
8375_1              YRTWAYHGSYEAPTQGSASSLINGVVRLLSKPWDVVTGVTGIAMTDTTPYGQQRVFKEKV    2880
103344_1            YRTWAYHGSYEAPTQGSASSLINGVVRLLSKPWDVVTGVTGIAMTDTTPYGQQRVFKEKV    2880
GD01_1              YRTWAYHGSYEAPTQGSASSLINGVVRLLSKPWDVVTGVTGIAMTDTTPYGQQRVFKEKV    2880
SPH2015_1           YRTWAYHGSYEAPTQGSASSLINGVVRLLSKPWDVVTGVTGIAMTDTTPYGQQRVFKEKV    2880
Haiti2014_1         YRTWAYHGSYEAPTQGSASSLINGVVRLLSKPWDVVTGVTGIAMTDTTPYGQQRVFKEKV    2880
PLCal_ZV_1          YRTWAYHGSYEAPTQGSASSLINGVVRLLSKPWDVVTGVTGIAMTDTTPYGQQRVFKEKV    2843
Martinique          YRTWAYHGSYEAPTQGSASSLINGVVRLLSKPWDVVTGVTGIAMTDTTPYGQQRVFKEKV    2880
NatalRGN            YRTWAYHGSYEAPTQGSASSLINGVVRLLSKPWDVVTGVTGIAMTDTTPYGQQRVFKEKV    2880
BrasilZKV2015_1     YRTWAYHGSYVAPTQGSASSLINGVVRLLSKPWDVVTGVTGIAMTDTTPYGQQRVFKEKV    2880
Z1106033_1          YRTWAYHGSYEAPTQGSASSLINGVVRLLSKPWDVVTGVTGIAMTDTTPYGQQRVFKEKV    2880
PRVABC59_1          YRTWAYHGSYEAPTQGSASSLINGVVRLLSKPWDVVTGVTGIAMTDTTPYGQQRVFKEKV    2880
HPF2013_1           YRTWAYHGSYEAPTQGSASSLINGVVRLLSKPWDVVTGVTGIAMTDTTPYGQQRVFKEKV    2880
Beh815744_1         YRTWAYHGSYEAPTQGSASSLINGVVRLLSKPWDVVTGVTGIAMTDTTPYGQQRVFKEKV    2880
BEH818995_1         YRTWAYHGSYEAPTQGSASSLINGVVRLLSKPWDVVTGVTGIAMTDTTPYGQQRVFKEKV    2880
BEH819966_1         YRTWAYHGSYEAPTQGSASSLINGVVRLLSKPWDVVTGVTGIAMTDTTPYGQQRVFKEKV    2880
BeH819015_1         YRTWAYHGSYEAPTQGSASSLINGVVRLLSKPWDVVTGVTGIAMTDTTPYGQQRVFKEKV    2880
SSABr_1             YRTWAYHGSYEAPTQGSASSLINGVVRLLSKPWDVVTGVTGIAMTDTTPYGQQRVFKEKV    2880
ARD157995_1         YRTWAYHGSYEAPTQGSASSLVNGVVRLLSKPWDVVTGVTGIAMTDTTPYGQQRVFKEKV    2880
MR_766_1            YRTWAYHGSYEAPTQGSASSLVNGVVRLLSKPWDVVTGVTGIAMTDTTPYGQQRVFKEKV    2876
ARD158084_1         YRTWAYHGSYEAPTQGSASSLVNGVVRLLSKPWDVVTGVTGIAMTDTTPYGQQRVFKEKV    2880
ArB1362_1           YRTWAYHGSYEAPTQGSASSLVNGVVRLLSKPWDVVTGVTGIAMTDTTPYGQQRVFKEKV    2880
ARB15076_1          YRTWAYHGSYEAPTQGSASSLVNGVVRLLSKPWDVVTGVTGIAMTDTTPYGQQRVFKEKV    2874
ARB13565_1          YRTWAYHGSYEAPTQGSASSLVNGVVRLLSKPWDVVTGVTGIAMTDTTPYGQQRVFKEKV    2880
ARB7701_1           YRTWAYHGSYEAPTQGSASSLVNGVVRLLSKPWDVVTGVTGIAMTDTTPYGQQRVFKEKV    2880
IbH30656_1          YRTWAYHGSYEAPTQGSASSLVNGVVRLLSKPWDVVTGVTGIAMTDTTPYGQQRVFKEKV    2874
ArD128000_1         YRTWAYHGSYEAPTQGSASSLVNGVVRLLSKPWDVVTGVTGIAMTDTTPYGQQRVFKEKV    2880
ArD7117_1           YRTWAYHGSYEAPTQGSASSLVNGVVRLLSKPWDVVTGVTGIAMTDTTPYGQQRVFKEKV    2880
ARD_41519_1         YRTWAYHGSYEAPTQGSASSLVNGVVRLLSKPWDVVTGVTGIAMTDTTPYGQQRVFKEKV    2880
                    ********** **********:**************************************

P6-740_1            DTRVPDPQEGTRQVMNMVSSWLWKELGKHKRPRVCTKEEFINKVRSNAALGAIFEEEKEW    2940
P6740               DTRVPDPQEGTRQVMNMVSSWLWKELGKHKRPRVCTKEEFINKVRSNAALGAIFEEEKEW    2940
Yap2007             DTRVPDPQEGTRQVMSMVSSWLWKELGKHKRPRVCTKEEFINKVRSNAALGAIFEEEKEW    2940
CPC0740_1           DTRVPDPQEGTRQVMSMVSSWLWKELGKHKRPRVCTKEEFINKVRSNAALGAIFEEEKEW    2940
SV0127/14_1         DTRVPDPQEGTRQVMSMVSSWLWKELGKHKRPRVCTKEEFINKVRSNAALGAIFEEEKEW    2940
FSS13025_1          DTRVPDPQEGTRQVMSMVSSWLWKELGKHKRPRVCTKEEFINKVRSNAALGAIFEEEKEW    2940
8375_1              DTRVPDPQEGTRQVMSMVSSWLWKELGKHKRPRVCTKEEFINKVRSNAALGAIFEEEKEW    2940
103344_1            DTRVPDPQEGTRQVMSMVSSWLWKELGKHKRPRVCTKEEFINKVRSNAALGAIFEEEKEW    2940
GD01_1              DTRVPDPQEGTRQVMSMVSSWLWKELGKHKRPRVCTKEEFINKVRSNAALGAIFEEEKEW    2940
SPH2015_1           DTRVPDPQEGTRQVMSMVSSWLWKELGKHKRPRVCTKEEFINKVRSNAALGAIFEEEKEW    2940
Haiti2014_1         DTRVPDPQEGTRQVMSMVSSWLWKELGKHKRPRVCTKEEFINKVRSNAALGAIFEEEKEW    2940
PLCal_ZV_1          DTRVPDPQEGTRQIMSMVSSWLWKELGKHKRPRVCTKEEFINKVRSNAALGAIFEEEKEW    2903
Martinique          DTRVPDPQEGTRQVMSMVSSWLWKELGKHKRPRVCTKEEFINKVRSNAALGAIFEEEKEW    2940
NatalRGN            DTRVPDPQEGTRQVMSMVSSWLWKELGKHKRPRVCTKEEFINKVRSNAALGAIFEEEKEW    2940
BrasilZKV2015_1     DTRVPDPQEGTRQVMSMVSSWLWKELGKHKRPRVCTKEEFINKVRSNAALGAIFEEEKEW    2940
Z1106033_1          DTRVPDPQEGTRQVMSMVSSWLWKELGKHKRPRVCTKEEFINKVRSNAALGAIFEEEKEW    2940
PRVABC59_1          DTRVPDPQEGTRQVMSMVSSWLWKELGKHKRPRVCTKEEFINKVRSNAALGAIFEEEKEW    2940
HPF2013_1           DTRVPDPQEGTRQVMSMVSSWLWKELGKHKRPRVCTKEEFINKVRSNAALGAIFEEEKEW    2940
Beh815744_1         DTRVPDPQEGTRQVMSMVSSWLWKELGKHKRPRVCTKEEFINKVRSNAALGAIFEEEKEW    2940
BEH818995_1         DTRVPDPQEGTRQVMSMVSSWLWKELGKHKRPRVCTKEEFINKVRSNAALGAIFEEEKEW    2940
BEH819966_1         DTRVPDPQEGTRQVMSMVSSWLWKELGKHKRPRVCTKEEFINKVRSNAALGAIFEEEKEW    2940
BeH819015_1         DTRVPDPQEGTRQVMSMVSSWLWKELGKHKRPRVCTKEEFINKVRSNAALGAIFEEEKEW    2940
SSABr_1             DTRVPDPQEGTRQVMSMVSSWLWKELGKHKRPRVCTKEEFINKVRSNAALGAIFEEEKEW    2940
ARD157995_1         DTRVPDPQEGTRQVMNMVSSWLWKELGKRKRPRVCTKEEFINKVRSNAALGAIFEEEKEW    2940
MR_766_1            DTRVPDPQEGTRQVMNIVSSWLWKELGKRKRPRVCTKEEFINKVRSNAALGAIFEEEKEW    2936
ARD158084_1         DTRVPDPQEGTRQVMNMVSSWLWKELGKRKRPRVCTKEEFINKVRSNAALGAIFEEEKEW    2940
ArB1362_1           DTRVPDPQEGTRQTMNMVSSWLWKELGKRKRPRVCTKEEFINKVRSNAALGAIFEEEKEW    2940
ARB15076_1          DTRVPDPQEGTRQTMNMVSSWLWKELGKRKRPRVCTKEEFINKVRSNAALGAIFEEEKEW    2934
ARB13565_1          DTRVPDPQEGTRQAMNMVSSWLWKELGKRKRPRVCTKEEFINKVRSNAALGAIFEEEKEW    2940
ARB7701_1           DTRVPDPQEGTRQAMNMVSSWLWKELGKRKRPRVCTKEEFINKVRSNAALGAIFEEEKEW    2940
IbH30656_1          DTRVPDPQEGTRRVMNMVSSWLWKELGKRKRPRVCTKEEFINKVRSNAALGAIFEEEKEW    2934
ArD128000_1         DTRVPDPQEGTRQVMNIVSSWLWKELGKRKRPRVCTKEEFINKVRSNAALGAIFEEEKEW    2940
ArD7117_1           DTRVPDPQEGTRQVMNMVSSWLWKELGKRKRPRVCTKEEFINKVRSNAALGAIFEEEKEW    2940
ARD_41519_1         DTRVPDPQEGTRQVMNMVSSWLWKELGKRKRPRVCTKEEFINKVRSNAALGAIFEEEKEW    2940
                    ************: *.:***********:*******************************
```

```
P6-740_1          KTAVEAVNDPRFWALVDKEREHHLRGECQSCVYNMMGKREKKQGEFGKAKGSRAIWYMWL   3000
P6740             KTAVEAVNDPRFWALVDKEREHHLRGECQSCVYNMMGKREKKQGEFGKAKGSRAIWYMWL   3000
Yap2007           KTAVEAVNDPRFWALVDKEREHHLRGECQSCVYNMMGKREKKQGEFGKAKGSRAIWYMWL   3000
CPC0740_1         KTAVEAVNDPRFWALVDKEREHHLRGECQSCVYNMMGKREKKQGEFGKAKGSRAIWYMWL   3000
SV0127/14_1       KTAVEAVNDPRFWALVDKEREHHLRGECQSCVYNMMGKREKKQGEFGKAKGSRAIWYMWL   3000
FSS13025_1        KTAVEAVNDPRFWALVDKEREHHLRGECQSCVYNMMGKREKKQGEFGKAKGSRAIWYMWL   3000
8375_1            KTAVEAVNDPRFWALVDKEREHHLRGECQSCVYNMMGKREKKQGEFGKAKGSRAIWYMWL   3000
103344_1          KTAVEAVNDPRFWALVDKEREHHLRGECQSCVYNMMGKREKKQGEFGKAKGSRAIWYMWL   3000
GD01_1            KTAVEAVNDPRFWALVDKEREHHLRGECQSCVYNMMGKREKKQGEFGKAKGSRAIWYMWL   3000
SPH2015_1         KTAVEAVNDPRFWALVDKEREHHLRGECQSCVYNMMGKREKKQGEFGKAKGSRAIWYMWL   3000
Haiti2014_1       KTAVEAVNDPRFWALVDKEREHHLRGECQSCVYNMMGKREKKQGEFGKAKGSRAIWYMWL   3000
PLCal_ZV_1        KTAVEAVNDPRFWALVDKEREHHLRGECQSCVYNMMGKREKKQGEFGKAKGSRAIWYMWL   2963
Martinique        KTAVEAVNDPRFWALVDKEREHHLRGECQSCVYNMMGKREKKQGEFGKAKGSRAIWYMWL   3000
NatalRGN          KTAVEAVNDPRFWALVDKEREHHLRGECQSCVYNMMGKREKKQGEFGKAKGSRAIWYMWL   3000
BrasilZKV2015_1   KTAVEAVNDPRFWALVDKEREHHLRGECQSCVYNMMGKREKKQGEFGKAKGSRAIWYMWL   3000
Z1106033_1        KTAVEAVNDPRFWALVDKEREHHLRGECQSCVYNMMGKREKKQGEFGKAKGSRAIWYMWL   3000
PRVABC59_1        KTAVEAVNDPRFWALVDKEREHHLRGECQSCVYNMMGKREKKQGEFGKAKGSRAIWYMWL   3000
HPF2013_1         KTAVEAVNDPRFWALVDKEREHHLRGECQSCVYNMMGKREKKQGEFGKAKGSRAIWYMWL   3000
Beh815744_1       KTAVEAVNDPRFWALVDKEREHHLRGECQSCVYNMMGKREKKQGEFGKAKGSRAIWYMWL   3000
BEH818995_1       KTAVEAVNDPRFWALVDKEREHHLRGECQSCVYNMMGKREKKQGEFGKAKGSRAIWYMWL   3000
BEH819966_1       KTAVEAVNDPRFWALVDKEREHHLRGECQSCVYNMMGKREKKQGEFGKAKGSRAIWYMWL   3000
BeH819015_1       KTAVEAVNDPRFWALVDKEREHHLRGECQSCVYNMMGKREKKQGEFGKAKGSRAIWYMWL   3000
SSABr_1           KTAVEAVNDPRFWALVDKEREHHLRGECQSCVYNMMGKREKKQGEFGKAKGSRAIWYMWL   3000
ARD157995_1       KTAVEAVNDPRFWALVDREREHHLRGECHSCVYNMMGKREKKQGEFGKAKGSRAIWYMWL   3000
MR_766_1          KTAVEAVNDPRFWALVDREREHHLRGECHSCVYNMMGKREKKQGEFGKAKGSRAIWYMWL   2996
ARD158084_1       KTAVEAVNDPRFWALVDREREHHLRGECHSCVYNMMGKREKKQGEFGKAKGSRAIWYMWL   3000
ArB1362_1         KTAVEAVNDPRFWALVDKEREHHLRGECHSCVYNMMGKREKKQGEFGKAKGSRAIWYMWL   3000
ARB15076_1        KTAVEAVNDPRFWALVDKEREHHLRGECHSCVYNMMGKREKKQGEFGKAKGSRAIWYMWL   2994
ARB13565_1        KTAVEAVNDPRFWALVDKEREHHLRGECHSCVYNMMGKREKKQGEFGKAKGSRAIWYMWL   3000
ARB7701_1         KTAVEAVNDPRFWALVDKEREHHLRGECHSCVYNMMGKREKKQGEFGKAKGSRAIWYMWL   3000
IbH30656_1        KTAVEAVNDPRFWALVDKEREHHLRGECHSCVYNMMGKREKKQGEFGKAKGSRAIWYMWL   2994
ArD128000_1       KTAVEAVNDPRFWALVDKEREHHLRGECHSCVYNMMGKREKKQGEFGKAKGSRAIWYMWL   3000
ArD7117_1         KTAVEAVNDPRFWALVDKEREHHLRGECHSCVYNMMGKREKKQGEFGKAKGSRAIWYMWL   3000
ARD_41519_1       KTAVEAVNDPRFWALVDKEREHHLRGECHSCVYNMMGKREKKQGEFGKAKGSRAIWYMWL   3000
                  *****************:**********:*******************************

P6-740_1          GARFLEFEALGFLNEDHWMGRENSGGGVEGLGLQRLGYVLEEMSRTPGGKMYADDTAGWD   3060
P6740             GARFLEFEALGFLNEDHWMGRENSGGGVEGLGLQRLGYVLEEMSRTPGGKMYADDTAGWD   3060
Yap2007           GARFLEFEALGFLNEDHWMGRENSGGGVEGLGLQRLGYVLEEMSRIPGGRMYADDTAGWD   3060
CPC0740_1         GARFLEFEALGFLNEDHWMGRENSGGGVEGLGLQRLGYVLEEMSRIPGGRMYADDTAGWD   3060
SV0127/14_1       GARFLEFEALGFLNEDHWMGRENSGGGVEGLGLQRLGYVLEEMSRIPGGRMYADDTAGWD   3060
FSS13025_1        GARFLEFEALGFLNEDHWMGRENSGGGVEGLGLQRLGYVLEEMSRIPGGRMYADDTAGWD   3060
8375_1            GARFLEFEALGFLNEDHWMGRENSGGGVEGLGLQRLGYVLEEMSCIPGGRMYADDTAGWD   3060
103344_1          GARFLEFEALGFLNEDHWMGRENSGGGVEGLGLQRLGYVLEEMSCIPGGRMYADDTAGWD   3060
GD01_1            GARFLEFEALGFLNEDHWMGRENSGGGVEGLGLQRLGYVLEEMSRIPGGRMYADDTAGWD   3060
SPH2015_1         GARFLEFEALGFLNEDHWMGRENSGGGVEGLGLQRLGYVLEEMSRIPGGRMYADDTAGWD   3060
Haiti2014_1       GARFLEFEALGFLNEDHWMGRENSGGGVEGLGLQRLGYVLEEMSRIPGGRMYADDTAGWD   3060
PLCal_ZV_1        GARFLEFEALGFLNEDHWMGRENSGGGVEGLGLQRLGYVLEEMSRIPGGRMYADDTAGWD   3023
Martinique        GARFLEFEALGFLNEDHWMGRENSGGGVEGLGLQRLGYVLEEMSRIPGGRMYADDTAGWD   3060
NatalRGN          GARFLEFEALGFLNEDHWMGRENSGGGVEGLGLQRLGYVLEEMSRIPGGRMYADDTAGWD   3060
BrasilZKV2015_1   GARFLEFEALGFLNEDHWMGRENSGGGVEGLGLQRLGYVLEEMSRIPGGRMYADDTAGWD   3060
Z1106033_1        GARFLEFEALGFLNEDHWMGRENSGGGVEGLGLQRLGYVLEEMSRIPGGRMYADDTAGWD   3060
PRVABC59_1        GARFLEFEALGFLNEDHWMGRENSGGGVEGLGLQRLGYVLEEMSRIPGGRMYADDTAGWD   3060
HPF2013_1         GARFLEFEALGFLNEDHWMGRENSGGGVEGLGLQRLGYVLEEMSRIPGGRMYADDTAGWD   3060
Beh815744_1       GARFLEFEALGFLNEDHWMGRENSGGGVEGLGLQRLGYVLEEMSRIPGGRMYADDTAGWD   3060
BEH818995_1       GARFLEFEALGFLNEDHWMGRENSGGGVEGLGLQRLGYVLEEMSRIPGGRMYADDTAGWD   3060
BEH819966_1       GARFLEFEALGFLNEDHWMGRENSGGGVEGLGLQRLGYVLEEMSRIPGGRMYADDTAGWD   3060
BeH819015_1       GARFLEFEALGFLNEDHWMGRENSGGGVEGLGLQRLGYVLEEMSRIPGGRMYADDTAGWD   3060
SSABr_1           GARFLEFEALGFLNEDHWMGRENSGGGVEGLGLQRLGYVLEEMSRIPGGRMYADDTAGWD   3060
ARD157995_1       GARFLEFESLGFLNEDHWMGRENSGGGVEGLGLQRLGYVLEEMSQAPGGKMYADDTAGWD   3060
MR_766_1          GARFLEFEALGFLNEDHWMGRENSGGGVEGLGLQRLGYILEEMNRAPGGKMYADDTAGWD   3056
ARD158084_1       GARFLEFEALGFLNEDHWMGRENSGGGVEGLGLQRLGYVLEEMSQAPGGKMYADDTAGWD   3060
ArB1362_1         GARFLEFESLGFLNEDHWMGRENSGGGVEGLGLQRLGYILEEMNRAPGGKMYADDTAGWD   3060
ARB15076_1        GARFLEFEALGFLNEDHWMGRENSGGGVEGLGLQRLGYILEEMNRAPGGKMYADDTAGWD   3054
ARB13565_1        GARFLEFEALGFLNEDHWMGRENSGGGVEGLGLQRLGYILEEMNRAPGGKMYADDTAGWD   3060
ARB7701_1         GARFLEFEALGFLNEDHWMGRENSGGGVEGLGLQRLGYILEEMNRAPGGKMYADDTAGWD   3060
IbH30656_1        GARFLEFEALGFLNEDHWMGRENSGGGVEGLGLQRLGYILEEMNRAPGGKMYADDTAGWD   3054
```

```
ArD128000_1        GARFLEFEALGFLNEDHWMGRENSGGGVEGLGLQRLGYILEEMNRAPGGKMYADDTAGWD    3060
ArD7117_1          GARFLEFEALGFLNEDHWMGRENSGGGVEGLGLQRLGYILEEMNRAPGGKMYADDTAGWD    3060
ARD_41519_1        GARFLEFEALGFLNEDHWMGRENSGGGVEGLGLQRLGYILEEMNRAPGGKMYADDTAGWD    3060
                   ********:*****************************:****.  ***:**********

P6-740_1           TRISRFDLENEALITNQMEKGHRALALAIIKYTYQNKVVKVLRPAERGKTVMDIISRQDQ    3120
P6740              TRISRFDLENEALITNQMEKGHRALALAIIKYTYQNKVVKVLRPAERGKTVMDIISRQDQ    3120
Yap2007            TRISRFDLENEALITNQMEKGHRALALAIIKYTYQNKVVKVLRPAEKGKTVMDIISRQDQ    3120
CPC0740_1          TRISRFDLENEALITNQMEKGHRALALAIIKYTYQNKVVKVLRPAEKGKTVMDIISRQDQ    3120
SV0127/14_1        TRISRFDLENEALITNQMEKGHRALALAIIKYTYQNKVVKVLRPAEKGKTVMDIISRQDQ    3120
FSS13025_1         TRISRFDLENEALITNQMEKGHRALALAIIKYTYQNKVVKVLRPAEKGKTVMDIISRQDQ    3120
8375_1             TRISRFDLENEALITNQMEKGHRALALAIIKYTYQNKVVKVLRPAEKGKTVMDIISRQDQ    3120
103344_1           TRISRFDLENEALITNQMEKGHRALALAIIKYTYQNKVVKVLRPAEKGKTVMDIISRQDQ    3120
GD01_1             TRISRFDLENEALITNQMEKGHRALALAIIKYTYQNKVVKVLRPAEKGKTVMDIISRQDQ    3120
SPH2015_1          TRISRFDLENEALITNQMEKGHRALALAIIKYTYQNKVVKVLRPAEKGKTVMDIISRQDQ    3120
Haiti2014_1        TRISRFDLENEALITNQMEKGHRALALAIIKYTYQNKVVKVLRPAEKGKTVMDIISRQDQ    3120
PLCal_ZV_1         TRISRFDLENEALITNQMEKGHRALALAIIKYTYQNKVVKVLRPAEKGKTVMDIISRQDQ    3083
Martinique         TRISRFDLENEALITNQMEKGHRALALAIIKYTYQNKVVKVLRPAEKGKTVMDIISRQDQ    3120
NatalRGN           TRISRFDLENEALITNQMEKGHRALALAIIKYTYQNKVVKVLRPAEKGKTVMDIISRQDQ    3120
BrasilZKV2015_1    TRISRFDLENEALITNQMEKGHRALALAIIKYTYQNKVVKVLRPAEKGKTVMDIISRQDQ    3120
Z1106033_1         TRISRFDLENEALITNQMEKGHRALALAIIKYTYQNKVVKVLRPAEKGKTVMDIISRQDQ    3120
PRVABC59_1         TRISRFDLENEALITNQMEKGHRALALAIIKYTYQNKVVKVLRPAEKGKTVMDIISRQDQ    3120
HPF2013_1          TRISRFDLENEALITNQMEKGHRALALAIIKYTYQNKVVKVLRPAEKGKTVMDIISRQDQ    3120
Beh815744_1        TRISRFDLENEALITNQMEKGHRALALAIIKYTYQNKVVKVLRPAEKGKTVMDIISRQDQ    3120
BEH818995_1        TRISRFDLENEALITNQMEKGHRALALAIIKYTYQNKVVKVLRPAEKGKTVMDIISRQDQ    3120
BEH819966_1        TRISRFDLENEALITNQMEKGHRALALAIIKYTYQNKVVKVLRPAEKGKTVMDIISRQDQ    3120
BeH819015_1        TRISRFDLENEALITNQMEKGHRALALAIIKYTYQNKVVKVLRPAEKGKTVMDIISRQDQ    3120
SSABr_1            TRISRFDLENEALITNQMEKGHRALALAIIKYTYQNKVVKVLRPAEKGKTVMDIISRQDQ    3120
ARD157995_1        TRISKFDLENEALITNQMEEGHRTLALAVIKYTYQNKVVKVLRPAEGGKTVMDIISRQDQ    3120
MR_766_1           TRISKFDLENEALITNQMEEGHRTLALAVIKYTYQNKVVKVLRPAEGGKTVMDIISRQDQ    3116
ARD158084_1        TRISKFDLENEALITNQMEEGHRTLALAVIKYTYQNKVVKVLRPAEGGKTVMDIISRQDQ    3120
ArB1362_1          TRISKFDLENEALITNQMEEGHRALALAVIKYTYQNKVVKVLRPAEGGKTVMDIISRQDQ    3120
ARB15076_1         TRISKFDLENEALITNQMEEGHRALALAVIKYTYQNKVVKVLRPAEGGKTVMDIISRQDQ    3114
ARB13565_1         TRISKFDLENEALITNQMEEGHRALALAVIKYT-KNKVVKVLRPAEGGKTVMDIISRQDQ    3119
ARB7701_1          TRISKFDLENEALITNQMEEGHRALALAVIKYT-KNKVVKVLRPAEGGKTVMDIISRQDQ    3119
IbH30656_1         TRISRFDLENEALITNQMEEGHRALALAVIKYTYQNKVVKVLRPAEGGKTVMDIISRQDQ    3114
ArD128000_1        TRISKFDLENEALITNQMEEGHRALALAVIKYTYQNKVVKVLRPAEGGKTVMDIISRQDQ    3120
ArD7117_1          TRISKFDLENEALITNQMEEGHRALALAVIKYTYQNKVVKVLRPAEGGKTVMDIISRQDQ    3120
ARD_41519_1        TRISKFDLENEALITNQMEEGHRALALAVIKYTYQNKVVKVLRPAEGGKTVMDIISRQDQ    3120
                   ****:**************:***:****:**** :*********** *************

P6-740_1           RGSGQVVTYALNTFTNLVVQLIRNMEAEEVLEMQDLWLLRRPEKVTSWLQSNGWDRLKRM    3180
P6740              RGSGQVVTYALNTFTNLVVQLIRNMEAEEVLEMQDLWLLRRPEKVTSWLQSNGWDRLKRM    3180
Yap2007            RGSGQVVTYALNTFTNLVVQLIRNMEAEEVLEMQDLWLLRRSEKVTNWLQSNGWDRLKRM    3180
CPC0740_1          RGSGQVVTYALNTFTNLVVQLIRNMEAEEVLEMQDLWLLRRPEKVTNWLQSNGWDRLKRM    3180
SV0127/14_1        RGSGQVVTYALNTFTNLVVQLIRNMEAEEVLEMQDLWLLRRSEKVTNWLQSNGWDRLKRM    3180
FSS13025_1         RGSGQVVTYALNTFTNLVVQLIRNMEAEEVLEMQDLWLLRRSEKVTNWLQSNGWDRLKRM    3180
8375_1             RGSGQVVTYALNTFTNLVVQLIRNMEAEEVLEMQDLWLLRRSEKVTNWLQSNGWDRLKRM    3180
103344_1           RGSGQVVTYALNTFTNLVVQLIRNMEAEEVLEMQDLWLLRRSEKVTNWLQSNGWDRLKRM    3180
GD01_1             RGSGQVVTYALNTFTNLVVQLIRNMEAEEVLEMQDLWLLRRSEKVTNWLQSNGWDRLKRM    3180
SPH2015_1          RGSGQVVTYALNTFTNLVVQLIRNMEAEEVLEMQDLWLLRRSEKVTNWLQSNGWDRLKRM    3180
Haiti2014_1        RGSGQVVTYALNTFTNLVVQLIRNMEAEEVLEMQDLWLLRRSEKVTNWLQSNGWDRLKRM    3180
PLCal_ZV_1         RGSGQVVTYALNTFTNLVVQLIRNMEAEEVLEMQDLWLLRRSEKVTNWLQSNGWDRLKRM    3143
Martinique         RGSGQVVTYALNTFTNLVVQLIRNMEAEEVLEMQDLWLLRRSEKVTNWLQSNGWDRLKRM    3180
NatalRGN           RGSGQVVTYALNTFTNLVVQLIRNMEAEEVLEMQDLWLLRRSEKVTNWLQSNGWDRLKRM    3180
BrasilZKV2015_1    RGSGQVVTYALNTFTNLVVQLIRNMEAEEVLEMQDLWLLRRSEKVTNWLQSNGWDRLKRM    3180
Z1106033_1         RGSGQVVTYALNTFTNLVVQLIRNMEAEEVLEMQDLWLLRRSEKVTNWLQSNGWDRLKRM    3180
PRVABC59_1         RGSGQVVTYALNTFTNLVVQLIRNMEAEEVLEMQDLWLLRRSEKVTNWLQSNGWDRLKRM    3180
HPF2013_1          RGSGQVVTYALNTFTNLVVQLIRNMEAEEVLEMQDLWLLRRSEKVTNWLQSNGWDRLKRM    3180
Beh815744_1        RGSGQVVTYALNTFTNLVVQLIRNMEAEEVLEMQDLWLLRRSEKVTNWLQSNGWDRLKRM    3180
BEH818995_1        RGSGQVVTYALNTFTNLVVQLIRNMEAEEVLEMQDLWLLRRSEKVTNWLQSNGWDRLKRM    3180
BEH819966_1        RGSGQVVTYALNTFTNLVVQLIRNMEAEEVLEMQDLWLLRRSEKVTNWLQSNGWDRLKRM    3180
BeH819015_1        RGSGQVVTYALNTFTNLVVQLIRNMEAEEVLEMQDLWLLRRSEKVTNWLQSNGWDRLKRM    3180
SSABr_1            RGSGQVVTYALNTFTNLVVQLIRNMEAEEVLEMQDLWLLRRSEKVTNWLQSNGWDRLKRM    3180
ARD157995_1        RGSGQVVTYALNTFTNLVVQLIRNMEAEEVLEMQDLWLLRKPEKVTRWLQSNGWDRLKRM    3180
MR_766_1           RGSGQVVTYALNTFTNLVVQLIRNMEAEEVLEMQDLWLLRKPEKVTRWLQSNGWDRLKRM    3176
ARD158084_1        RGSGQVVTYALNTFTNLVVQLIRNMEAEEVLEMQDLWLLRKPEKVTRWLQSNGWDGLKRM    3180
ArB1362_1          RGSGQVVTYALNTFTNLVVQLIRNMEAEEVLEMQDLWLLRKPEKVTRWLQSNGWDRLKRM    3180
```

```
ARB15076_1          RGSGQVVTYALNTFTNLVVQLIRNMEAEEVLEMQDLWLLRKPEKVTRWLQCNGWDRLKRM       3174
ARB13565_1          RGSGQVVTYALNTFTNLVVQLIRNMEAEEVLEMQDLWLLRKPEKVTRWLQCNGWDRLKRM       3179
ARB7701_1           RGSGQVVTYALNTFTNLVVQLIRNMEAEEVLEMQDLWLLRKPEKVTRWLQCNGWDRLKRM       3179
IbH30656_1          RGSGQVVTYALNTFTNLVVQLIRNMEAEEVLEMHDLWLLRKPEKVTRWLQSNGWDRLKRM       3174
ArD128000_1         RGSGQVVTYALNTFTNLVVQLIRNMEAEEVLEMHDLWLLRKPEKVTRWLQSNGWDRLKRM       3180
ArD7117_1           RGSGQVVTYALNTFTNLVVQLIRNMEAEEVLEMHDLWLLRKPEKVTRWLQSNGWDRLKRM       3180
ARD_41519_1         RGSGQVVTYALNTFTNLVVQLIRNMEAEEVLEMHDLWLLRKPEKVTRWLQSNGWDRLKRM       3180
                    *********************************:******: **** ***.**** ****

P6-740_1            AVSGDDCVVKPIDDRFAHALRFLNDMGKVRKDTQEWKPSTGWSNWEEVPFCSHHFNKLYL       3240
P6740               AVSGDDCVVKPIDDRFAHALRFLNDMGKVRKDTQEWKPSTGWSNWEEVPFCSHHFNKLYL       3240
Yap2007             AVSGDDCVVKPIDDRFAHALRFLNDMGKVRKDTQEWKPSTGWDNWEEVPFCSHHFNKLHL       3240
CPC0740_1           AVSGDDCVVKPIDDRFAHALRFLNDMGKVRKDTQEWKPSTGWDNWEEVPFCSHHFNKLHL       3240
SV0127/14_1         AVSGDDCVVKPIDDRFAHALRFLNDMGKVRKDTQEWKPSTGWDNWEEVPFCSHHFNKLHL       3240
FSS13025_1          AVSGDDCVVKPIDDRFAHALRFLNDMGKVRKDTQEWKPSTGWDNWEEVPFCSHHFNKLHL       3240
8375_1              AVSGDDCVVKPIDDRFAHALRFLNDMGKVRKDTQEWKPSTGWDNWEEVPFCSHHFNKLHL       3240
103344_1            AVSGDDCVVKPIDDRFAHALRFLNDMGKVRKDTQEWKPSTGWDNWEEVPFCSHHFNKLHL       3240
GD01_1              AVSGDDCVVKPIDDRFAHALRFLNDMGKVRKDTQEWKPSTGWDNWEEVPFCSHHFNKLHL       3240
SPH2015_1           AVSGDDCVVKPIDDRFAHALRFLNDMGKVRKDTQEWKPSTGWDNWEEVPFCSHHFNKLHL       3240
Haiti2014_1         AVSGDDCVVKPIDDRFAHALRFLNDMGKVRKDTQEWKPSTGWDNWEEVPFCSHHFNKLHL       3240
PLCal_ZV_1          AVSGDDCVVKPIDDRFAHALRFLNDMGKVRKDTQEWKPSTGWDNWEEVPFCSHHFNKLHL       3203
Martinique          AVSGDDCVVKPIDDRFAHALRFLNDMGKVRKDTQEWKPSTGWDNWEEVPFCSHHFNKLHL       3240
NatalRGN            AVSGDDCVVKPIDDRFAHALRFLNDMGKVRKDTQEWKPSTGWDNWEEVPFCSHHFNKLHL       3240
BrasilZKV2015_1     AVSGDDCVVKPIDDRFAHALRFLNDMGKVRKDTQEWKPSTGWDNWEEVPFCSHHFNKLHL       3240
Z1106033_1          AVSGDDCVVKPIDDRFAHALRFLNDMGKVRKDTQEWKPSTGWDNWEEVPFCSHHFNKLHL       3240
PRVABC59_1          AVSGDDCVVKPIDDRFAHALRFLNDMGKVRKDTQEWKPSTGWDNWEEVPFCSHHFNKLHL       3240
HPF2013_1           AVSGDDCVVKPIDDRFAHALRFLNDMGKVRKDTQEWKPSTGWDNWEEVPFCSHHFNKLHL       3240
Beh815744_1         AVSGDDCVVKPIDDRFAHALRFLNDMGKVRKDTQEWKPSTGWDNWEEVPFCSHHFNKLHL       3240
BEH818995_1         AVSGDDCVVKPIDDRFAHALRFLNDMGKVRKDTQEWKPSTGWDNWEEVPFCSHHFNKLHL       3240
BEH819966_1         AVSGDDCVVKPIDDRFAHALRFLNDMGKVRKDTQEWKPSTGWDNWEEVPFCSHHFNKLHL       3240
BeH819015_1         AVSGDDCVVKPIDDRFAHALRFLNDMGKVRKDTQEWKPSTGWDNWEEVPFCSHHFNKLHL       3240
SSABr_1             AVSGDDCVVKPIDDRFAHALRFLNDMGKVRKDTQEWKPSTGWDNWEEVPFCSHHFNKLHL       3240
ARD157995_1         AVSGDDCVVKPIDDRFAHALRFLNDMGKVRKDTQEWKPSTGWSNWEEVPFCSHHFNKLYL       3240
MR_766_1            AVSGDDCVVKPIDDRFAHALRFLNDMGKVRKDTQEWKPSTGWSNWEEVPFCSHHFNKLYL       3236
ARD158084_1         AVSGDDCVVKPIDDRFAHALRFLNDMGKVRKDTQEWKPSTGWSNWEEVPFCSHHFNKLYL       3240
ArB1362_1           AVSGDDCVVKPIDDRFACCLRFLNDMGKVRKDTQEWKPSTGWSNWEEVPFCSHHFNKLHL       3240
ARB15076_1          AVSGDDCVVKPIDDRFAHALRFLNDMGKVRKDTQEWKPSTGWSNWEEVPFCSHHFNKLHL       3234
ARB13565_1          AVSGDDCVVKPIDDRFAHALRFLNDMGKVRKDTQEWKPSTGWSNWEEVPFCSHHFNKLHL       3239
ARB7701_1           AVSGDDCVVKPIDDRFAHALRFLNDMGKVRKDTQEWKPSTGWSNWEEVPFCSHHFNKLHL       3239
IbH30656_1          AVSGDDCVVKPIDDRFAHALRFLNDMGKVRKDTQEWKPSTGWSNWEEVPFCSHHFNKLHL       3234
ArD128000_1         AVSGDDCVVKPIDDRFAHALRFLNDMGKVRKDTQEWKPSTGWSNWEEVPFCSHHFNKLHL       3240
ArD7117_1           AVSGDDCVVKPIDDRFAHALRFLNDMGKVRKDTQEWKPSTGWSNWEEVPFCSHHFNKLHL       3240
ARD_41519_1         AVSGDDCVVKPIDDRFAHALRFLNDMGKVRKDTQEWKPSTGWSNWEEVPFCSHHFNKLHL       3240
                    ***************** .***********************.***************:*

P6-740_1            KDGRSIVVPCRHQDELIGRARVSPGAGWSIRETACLAKSYAQMWQLLYFHRRDLRLMANA       3300
P6740               KDGRSIVVPCRHQDELIGRARVSPGAGWSIRETACLAKSYAQMWQLLYFHRRDLRLMANA       3300
Yap2007             KDGRSIVVPCRHQDELIGRARVSPGAGWSIRETACLAKSYAQMWQLLYFHRRDLRLMANA       3300
CPC0740_1           KDGRSIVVPCRHQDELIGRARVSPGAGWSIRETACLAKSYAQMWQLLYFHRRDLRLMANA       3300
SV0127/14_1         KDGRSIVVPCRHQDELIGRARVSPGAGWSIRETACLAKSYAQMWQLLYFHRRDLRLMANA       3300
FSS13025_1          KDGRSIVVPCRHQDELIGRARVSPGAGWSIRETACLAKSYAQMWQLLYFHRRDLRLMANA       3300
8375_1              KDGRSIVVPCRHQDELIGRARVSPGAGWSIRETACLAKSYAQMWQLLYFHRRDLRLMANA       3300
103344_1            KDGRSIVVPCRHQDELIGRARVSPGAGWSIRETACLAKSYAQMWQLLYFHRRDLRLMANA       3300
GD01_1              KDGRSIVVPCRHQDELIGRARVSPGAGWSIRETACLAKSYAQMWQLLYFHRRDLRLMANA       3300
SPH2015_1           KDGRSIVVPCRHQDELIGRARVSPGAGWSIRETACLAKSYAQMWQLLYFHRRDLRLMANA       3300
Haiti2014_1         KDGRSIVVPCRHQDELIGRARVSPGAGWSIRETACLAKSYAQMWQLLYFHRRDLRLMANA       3300
PLCal_ZV_1          KDGRSIVVPCRHQDELIGRARVSPGAGWSIRETACLAKSYAQMWQLLYFHRRDLRLMANA       3263
Martinique          KDGRSIVVPCRHQDELIGRARVSPGAGWSIRETACLAKSYAQMWQLLYFHRRDLRLMANA       3300
NatalRGN            KDGRSIVVPCRHQDELIGRARVSPGAGWSIRETACLAKSYAQMWQLLYFHRRDLRLMANA       3300
BrasilZKV2015_1     KDGRSIVVPCRHQDELIGRARVSPGAGWSIRETACLAKSYAQMWQLLYFHRRDLRLMANA       3300
Z1106033_1          KDGRSIVVPCRHQDELIGRARVSPGAGWSIRETACLAKSYAQMWQLLYFHRRDLRLMANA       3300
PRVABC59_1          KDGRSIVVPCRHQDELIGRARVSPGAGWSIRETACLAKSYAQMWQLLYFHRRDLRLMANA       3300
HPF2013_1           KDGRSIVVPCRHQDELIGRARVSPGAGWSIRETACLAKSYAQMWQLLYFHRRDLRLMANA       3300
Beh815744_1         KDGRSIVVPCRHQDELIGRARVSPGAGWSIRETACLAKSYAQMWQLLYFHRRDLRLMANA       3300
BEH818995_1         KDGRSIVVPCRHQDELIGRARVSPGAGWSIRETACLAKSYAQMWQLLYFHRRDLRLMANA       3300
BEH819966_1         KDGRSIVVPCRHQDELIGRARVSPGAGWSIRETACLAKSYAQMWQLLYFHRRDLRLMANA       3300
BeH819015_1         KDGRSIVVPCRHQDELIGRARVSPGAGWSIRETACLAKSYAQMWQLLYFHRRDLRLMANA       3300
SSABr_1             KDGRSIVVPCRHQDELIGRARVSPGAGWSIRETACLAKSYAQMWQLLYFHRRDLRLMANA       3300
```

```
ARD157995_1         KDGRSIVVPCRHQDELIGRARVSPGAGWSIRETACLAKSYAQMWQLLYFHRRDLRLMANA       3300
MR_766_1            KDGRSIVVPCRHQDELIGRARVSPGAGWSIRETACLAKSYAQMWQLLYFHRRDLRLMANA       3296
ARD158084_1         KDGRSIVVPCRHQDELIGRARVSPGAGWSIRETACLAKSYAQMWQLLYFHRRDLRLMANA       3300
ArB1362_1           KDGRSIVVPCRHQDELIGRARVSPGAGWSIRETACLAKSYAQMWQLLYFHRRDLRLMANA       3300
ARB15076_1          KDGRSIVVPCRHQDELIGRARVSPGAGWSIRETACLAKSYAQMWQLLYFHRRDLRLMANA       3294
ARB13565_1          KDGRSIVVPCRHQDELIGRARVSPGAGWSIRETACLAKSYAQMWQLLYFHRRDLRLMANA       3299
ARB7701_1           KDGRSIVVPCRHQDELIGRARVSPGAGWSIRETACLAKSYAQMWQLLYFHRRDLRLMANA       3299
IbH30656_1          KDGRSIVVPCRHQDELIGRARVSPGAGWSIRETACLAKSYAQMWQLLYFHRRDLRLMANA       3294
ArD128000_1         KDGRSIVVPCRHQDELIGRARVSPGAGWSIRETACLAKSYAQMWQLLYFHRRDLRLMANA       3300
ArD7117_1           KDGRSIVVPCRHQDELIGRARVSPGAGWSIRETACLAKSYAQMWQLLYFHKRDLRLMANA       3300
ARD_41519_1         KDGRSIVVPCRHQDELIGRARVSPGAGWSIRETACLAKSYAQMWQLLYFHRRDLRLMANA       3300
                    **************************************************:*********

P6-740_1            ICSSVPVDWVPTGRTTWSIHGKGEWMTTEDMLVVWNRVWIEENDHMEDKTPVTKWTDIPY       3360
P6740               ICSSVPVDWVPTGRTTWSIHGKGEWMTTEDMLVVWNRVWIEENDHMEDKTPVTKWTDIPY       3360
Yap2007             ICSSVPVDWVPTGRTTWSIHGKGEWMTTEDMLVVWNRVWIEENDHMEDKTPVTKWTDIPY       3360
CPC0740_1           ICSSVPVDWVPTGRTTWSIHGKGEWMTTEDMLVVWNRVWIEENDHMEDKTPVTKWTDIPY       3360
SV0127/14_1         ICSSVPVDWVPTGRTTWSIHGKGEWMTTEDMLVVWNRVWIEENDHMEDKTPVTKWTDIPY       3360
FSS13025_1          ICSSVPVDWVPTGRTTWSIHGKGEWMTTEDMLVVWNRVWIEENDHMEDKTPVTKWTDIPY       3360
8375_1              ICSSVPVDWVPTGRTTWSIHGKGEWMTTEDMLVVWNRVWIEENDHMEDKTPVTKWTDIPY       3360
103344_1            ICSSVPVDWVPTGRTTWSIHGKGEWMTTEDMLVVWNRVWIEENDHMEDKTPVTKWTDIPY       3360
GD01_1              ICSSVPVDWVPTGRTTWSIHGKGEWMTTEDMLVVWNRVWIEENDHMEDKTPVTKWTDIPY       3360
SPH2015_1           ICSSVPVDWVPTGRTTWSIHGKGEWMTTEDMLVVWNRVWIEENDHMEDKTPVTKWTDIPY       3360
Haiti2014_1         ICSSVPVDWVPTGRTTWSIHGKGEWMTTEDMLVVWNRVWIEENDHMEDKTPVTKWTDIPY       3360
PLCal_ZV_1          ICSSVPVDWVPTGRTTWSIHGKGEWMTTEDMLVVWNRVWIEENDHMEDKTPVTKWTDIPY       3323
Martinique          ICSSVPVDWVPTGRTTWSIHGKGEWMTTEDMLVVWNRVWIEENDHMEDKTPVAKWTDIPY       3360
NatalRGN            ICSSVPVDWVPTGRTTWSIHGKGEWMTTEDMLVVWNRVWIEENDHMEDKTPVTKWTDIPY       3360
BrasilZKV2015_1     ICSSVPVDWVPTGRTTWSIHGKGEWMTTEDMLVVWNRVWIEENDHMEDKTPVTKWTDIPY       3360
Z1106033_1          ICSSVPVDWVPTGRTTWSIHGKGEWMTTEDMLVVWNRVWIEENDHMEDKTPVTKWTDIPY       3360
PRVABC59_1          ICSSVPVDWVPTGRTTWSIHGKGEWMTTEDMLVVWNRVWIEENDHMEDKTPVTKWTDIPY       3360
HPF2013_1           ICSSVPVDWVPTGRTTWSIHGKGEWMTTEDMLVVWNRVWIEENDHMEDKTPVTKWTDIPY       3360
Beh815744_1         ICSSVPVDWVPTGRTTWSIHGKGEWMTTEDMLVVWNRVWIEENDHMEDKTPVTKWTDIPY       3360
BEH818995_1         ICSSVPVDWVPTGRTTWSIHGKGEWMTTEDMLVVWNRVWIEENDHMEDKTPVTKWTDIPY       3360
BEH819966_1         ICSSVPVDWVPTGRTTWSIHGKGEWMTTEDMLVVWNRVWIEENDHMEDKTPVTKWTDIPY       3360
BeH819015_1         ICSSVPVDWVPTGRTTWSIHGKGEWMTTEDMLVVWNRVWIEENDHMEDKTPVTKWTDIPY       3360
SSABr_1             ICSSVPVDWVPTGRTTWSIHGKGEWMTTEDMLVVWNRVWIEENDHMEDKTPVTKWTDIPY       3360
ARD157995_1         ICSAVPVDWVPTGRTTWSIHGKGEWMTTEDMLMVWNRVWIEENDHMEDKTPVTKWTDIPY       3360
MR_766_1            ICSAVPVDWVPTGRTTWSIHGKGEWMTTEDMLMVWNRVWIEENDHMEDKTPVTKWTDIPY       3356
ARD158084_1         ICSAVPVDWVPTGRTTWSIHGKGEWMTTEDMLMVWNRVWIEENDHMEDKTPVTKWTDIPY       3360
ArB1362_1           ICSAVPVDWVPTGRTTWSIHGKGEWMTTEDMLMVWNRVWIEENDHMEDKTPVTKWTDIPY       3360
ARB15076_1          ICSAVPVDWVPTGRTTWSIHGKGEWMTTEDMLMVWNRVWIEENDHMEDKTPVTKWTDIPY       3354
ARB13565_1          ICSAVPVDWVPTGRTTWSIHGKGEWMTTEDMLMVWNRVWIEENDHMEDKTPVTKWTDIPY       3359
ARB7701_1           ICSAVPVDWVPTGRTTWSIHGKGEWMTTEDMLMVWNRVWIEENDHMEDKTPVTKWTDIPY       3359
IbH30656_1          ICSAVPADWVPTGRTTWSIHGKGEWMTNEDMLMVWNRVWIEENDHMGDKTPVTKWTDIPY       3354
ArD128000_1         ICSAVPVDWVPTGRTTWSIHGKGEWMTTEDMLMVWNRVWIEENDHMEDKTPVPKWTEIPY       3360
ArD7117_1           ICSAVPVDWVPTGRTTWSIHGKGEWMTTEDMLMVWNRVWIEENDHMEDKTPVTKWTDIPY       3360
ARD_41519_1         ICSAVPVDWVPTGRTTWSIHGKGEWMTTEDMLMVWNRVWIEENDHMEDKTPVTKWTDIPY       3360
                    ***:**.*******************.*****:************* ***** ***:***

P6-740_1            LGKREDLWCGSLIGHRPRTTWAENIKDTVNMVRRIIGDEEKYMDYLSTQVRYLGEEGSTP       3420
P6740               LGKREDLWCGSLIGHRPRTTWAENIKDTVNMVRRIIGDEEKYMDYLSTQVRYLGEEGSTP       3420
Yap2007             LGKREDLWCGSLIGHRPRTTWAENIKNTVNMMRRIIGDEEKYMDYLSTQVRYLGEEGSTP       3420
CPC0740_1           LGKREDLWCGSLIGHRPRTTWAENIKNTVNMMRRIIGDEEKYMDYLSTQVRYLGEEGSTP       3420
SV0127/14_1         LGKREDLWCGSLIGHRPRTTWAENIKNTVNMVRRIIGDEEKYMDYLSTQVRYLGEEGSTP       3420
FSS13025_1          LGKREDLWCGSLIGHRPRTTWAENIKNTVNMMRRIIGDEEKYVDYLSTQVRYLGEEGSTP       3420
8375_1              LGKREDLWCGSLIGHRPRTTWAENIKNTVNMVRRIIGDEEKYMDYLSTQVRYLGEEGSTP       3420
103344_1            LGKREDLWCGSLIGHRPRTTWAENIKNTVNMVRRIIGDEEKYMDYLSTQVRYLGEEGSTP       3420
GD01_1              LGKREDLWCGSLIGHRPRTTWAENIKNTVNMVRRIIGDEEKYMDYLSTQVRYLGEEGSTP       3420
SPH2015_1           LGKREDLWCGSLIGHRPRTTWAENIKNTVNMVRRIIGDEEKYMDYLSTQVRYLGEEGSTP       3420
Haiti2014_1         LGKREDLWCGSLIGHRPRTTWAENIKNTVNMVRRIIGDEEKYMDYLSTQVRYLGEEGSTP       3420
PLCal_ZV_1          LGKREDLWCGSLIGHRPRTTWAENIKNTVNMVRRIIGDEEKYMDYLSTQVRYLGEEG---       3380
Martinique          LGKREDLWCGSLIGHRPRTTWAENIKNTVNMVRRIIGDEEKYMDYLSTQVRYLGEEGSTP       3420
NatalRGN            LGKREDLWCGSLIGHRPRTTWAENIKNTVNMVRRIIGDEEKYMDYLSTQVRYLGEEGSTP       3420
BrasilZKV2015_1     LGKREDLWCGSLIGHRPRTTWAENIKNTVNMVRRIIGDEEKYMDYLSTQVRYLGEEGSTP       3420
Z1106033_1          LGKREDLWCGSLIGHRPRTTWAENIKNTVNMVRRIIGDEEKYMDYLSTQVRYLGEEGSTP       3420
PRVABC59_1          LGKREDLWCGSLIGHRPRTTWAENIKNTVNMVRRIIGDEEKYMDYLSTQVRYLGEEGSTP       3420
HPF2013_1           LGKREDLWCGSLIGHRPRTTWAENIKNTVNMVRRIIGDEEKYMDYLSTQVRYLGEEGSTP       3420
Beh815744_1         LGKREDLWCGSLIGHRPRTTWAENIKNTVNMVRRIIGDEEKYMDYLSTQVRYLGEEGSTP       3420
```

```
BEH818995_1         LGKREDLWCGSLIGHRPRTTWAENIKNTVNMVRRIIGDEEKYMDYLSTQVRYLGEEGSTP       3420
BEH819966_1         LGKREDLWCGSLIGHRPRTTWAENIKNTVNMVRRIIGDEEKYMDYLSTQVRYLGEEGSTP       3420
BeH819015_1         LGKREDLWCGSLIGHRPRTTWAENIKNTVNMVRRIIGDEEKYMDYLSTQVRYLGEEGSTP       3420
SSABr_1             LGKREDLWCGSLIGHRPRTTWAENIKNTVNMVRRIIGDEEKYMDYLSTQVRYLGEEGSTP       3420
ARD157995_1         LGKREDLWCGSLIGHRPRTTWAENIKDTVNMVRRIIGDEEKYMDYLSTQVRYLGEEGSTP       3420
MR_766_1            LGKREDLWCGSLIGHRPRTTWAENIKDTVNMVRRIIGDEEKYMDYLSTQVRYLGEEGSTP       3416
ARD158084_1         LGKREDLWCGSLIGHRPRTTWAENIKDTVNMVRRIIGDEEKYMDYLSTQVRYLGEEGSTP       3420
ArB1362_1           LGKREDLWCGSLIGHRPRTTWAENIKDTVNMVRRIIGDEEKYMDYLSTQVRYLGEEGSTP       3420
ARB15076_1          LGKREDLWCGSLIGHRPRTTWAENIKDTVNMVRRIIGDEEKYMDYLSTQVRYLGEEGSTP       3414
ARB13565_1          LGKREDLWCGSLIGHRPRTTWAENIKDTVNMVRRIIGDEEKYMDYLSTQVRYLGEEGSTP       3419
ARB7701_1           LGKREDLWCGSLIGHRPRTTWAENIKDTVNMVRRIIGDEEKYMDYLSTQVRYLGEEGSTP       3419
IbH30656_1          LGKREDLWCGSLIGHRPRTTWAENIKDTVNMVRRIIGDEERYMDYLSTQVRYLGEEGSTP       3414
ArD128000_1         LGKRESLWCGYIIGHRPRTTWAENIKDTVNMVLRLIGDEEKYMDYLSTQVRYLGEEGSTP       3420
ArD7117_1           LGKREDLWCGSLIGHRPRTTWAENIKDTVNMVRRIIGDEEKYMDYLSTQVRYLGEEGSTP       3420
ARD_41519_1         LGKREDLWCGSLIGHRPRTTWAENIKDTVNMVRRIIGDEEKYMDYLSTQVRYLGEEGSTP       3420
                    *****.**** :**************:****: *:*****:*:**************

P6-740_1            GVL        3423
P6740               GVL        3423
Yap2007             GVL        3423
CPC0740_1           GVL        3423
SV0127/14_1         GVL        3423
FSS13025_1          GVL        3423
8375_1              GVL        3423
103344_1            GVL        3423
GD01_1              GVL        3423
SPH2015_1           GVL        3423
Haiti2014_1         GVL        3423
PLCal_ZV_1          ---        3380
Martinique          GVL        3423
NatalRGN            GVL        3423
BrasilZKV2015_1     GVL        3423
Z1106033_1          GVL        3423
PRVABC59_1          GVL        3423
HPF2013_1           GVL        3423
Beh815744_1         GVL        3423
BEH818995_1         GVL        3423
BEH819966_1         GVL        3423
BeH819015_1         GVL        3423
SSABr_1             GVL        3423
ARD157995_1         GVL        3423
MR_766_1            GVL        3419
ARD158084_1         GVL        3423
ArB1362_1           GVL        3423
ARB15076_1          GVL        3417
ARB13565_1          GVL        3422
ARB7701_1           GVL        3422
IbH30656_1          GVL        3417
ArD128000_1         GVL        3423
ArD7117_1           GVL        3423
ARD_41519_1         GVL        3423
```

PLEASE NOTE: Showing colors on large alignments is slow.
