# Supplementary material for: A Comprehensive Systems Biology Approach to Studying Zika Virus
Source: PLoS One. 2016 Sep 1;11(9):e0161355. doi: 10.1371/journal.pone.0161355 (PMC5008700; doi:10.1371/journal.pone.0161355)
Supplement: S4 Data — (PDF) [file pone.0161355.s004.pdf]

CLUSTAL O(1.2.1) multiple sequence alignment

```
P6-740              ATGAAAAACCCAAAAAAGAAATCCGGAGGATTCCGGATTGTCAATATGCTAAAACGCGGA 60
CPC0740             ATGAAAAACCCAAAAAAGAAATCCGGAGGATTCCGGATTGTCAATATGCTAAAACGCGGA 60
YAP2007             ATGAAAAACCCCAAAGAAGAAATCCGGAGGATCCGGATTGTCAATATGCTAAAACGCGGA 60
FSS13025            ATGAAAAACCCAAAGAAGAAATCCGGAGGATTCCGGATTGTCAATATGCTAAAACGCGGA 60
PLCal_ZV            ------------------------------------------------------------ 0
SV0127/14           ATGAAAAACCCAAAAAAGAAATCCGGAGGATTCCGGATTGTCAATATGCTAAAACGCGGA 60
8375                ATGAAAAACCCAAAAAAGAAATCCGGAGGATTCCGGATTGTCAATATGCTAAAACGCGGA 60
103344              ATGAAAAACCCAAAAAAGAAATCCGGAGGATTCCGGATTGTCAATATGCTAAAACGCGGA 60
BrasilZKV2015       ATGAAAAACCCAAAAAAGAAATCCGGAGGATTCCGGATTGTCAATATGCTAAAACGCGGA 60
GD01                ATGAAAAACCCAAAAAAGAAATCCGGAGGATTCCGGATTGTCAATATGCTAAAACGCGGA 60
Martinique          ATGAAAAACCCAAAAAAGAAATCCGGAGGATTCCGGATTGTCAATATGCTAAAACGCGGA 60
NatalRGN            ATGAAAAACCCAAAAAAGAAATCCGGAGGATTCCGGATTGTCAATATGCTAAAACGCGGA 60
HPF2013             ATGAAAAACCCAAAAAAGAAATCCGGAGGATTCCGGATTGTCAATATGCTAAAACGCGGA 60
SPH2015             ATGAAAAACCCAAAAAAGAAATCCGGAGGATTCCGGATTGTCAATATGCTAAAACGCGGA 60
Haiti2014           ATGAAAAACCCAAAAAAGAAATCCGGAGGATTCCGGATTGTCAATATGCTAAAACGCGGA 60
PRVABC59            ATGAAAAACCCAAAAAAGAAATCCGGAGGATTCCGGATTGTCAATATGCTAAAACGCGGA 60
BeH819015           ATGAAAAACCCAAAAAAGAAATCCGGAGGATTCCGGATTGTCAATATGCTAAAACGCGGA 60
Z1106033            ATGAAAAACCCAAAAAAGAAATCCGGAGGATTCCGGATTGTCAATATGCTAAAACGCGGA 60
BEH819966           ATGAAAAACCCAAAAAAGAAATCCGGAGGATTCCGGATTGTCAATATGCTAAAACGCGGA 60
SSABr               ATGAAAAACCCAAAAAAGAAATCCGGAGGATTCCGGATTGTCAATATGCTAAAACGCGGA 60
Beh815744           ATGAAAAACCCAAAAAAGAAATCCGGAGGATTCCGGATTGTCAATATGCTAAAACGCGGA 60
BEH818995           ATGAAAAACCCAAAAAAGAAATCCGGAGGATTCCGGATTGTCAATATGCTAAAACGCGGA 60
IbH30656            ATGAAAAACCCAAAGAAGAAATCCGGAGGATTCCGGATTGTCAATATGCTAAAACGCGGA 60
ArD128000           ATGAAAAACCCAAAGAGGAAATCCGGAGGATTCCGGATTGTCAATATGCTAAAACGCGGA 60
ArD7117             ATGAAAAACCCAAAGAAGAGATCCGGAGGATTCCGGATTGTCAATATGCTAAAACGCGGA 60
ARD_41519           ATGAAAAACCCAAAGAGGAAATCCGGAGGATTCCGGATTGTCAATATGCTAAAACGCGGA 60
ARD157995           ATGAAAAACCCAAAGAAGAAATCCGGAAGATTCCGGATTGTCAATATGCTAAAACGCGGA 60
MR_766              ATGAAAAACCCCAAAGAAGAAATCCGGAGGATCCGGATTGTCAATATGCTAAAACGCGGA 60
ARD158084           ATGAAAAACCCAAAGAAGAAATCCGGAGGATTCCGGATTGTCAATATGCTAAAACGCGGA 60
ArB1362             ATGAAAAACCCAAAGAAGAAATCCGGAGGATTCCGGATTGTCAATATGCTAAAACGCGGA 60
ARB13565            ATGAAAAACCCAAAGAAGAAATCCGGAGGATTCCGGATTGTCAATATGCTAAAACGCGGA 60
ARB7701             ATGAAAAACCCAAAGAAGAAATCCGGAGGATTCCGGATTGTCAATATGCTAAAACGCGGA 60
ARB15076            ATGAAAAACCCAAAGAAGAAATCCGGAGGATTCCGGATTGTCAATATGCTAAAACGCGGA 60


P6-740              GTAGCCCGTGTGAGCCCCTTTGGGGGCTTGAAGAGGCTACCAGCTGGACTTCTGCTGGGT 120
CPC0740             GTAGCCCGTGTGAGCCCCTTTGGGGGCTTGAAGAGGCTGCCAGCCGGACTTCTGCTGGGC 120
YAP2007             GTAGCCCGTGTGAGCCCCTTTGGGGGCTTGAAGAGGCTGCCAGCCGGACTTCTGCTGGGT 120
FSS13025            GTAGCCCGTGTGAGCCCCTTTGGGGGCTTGAAGAGGCTGCCAGCCGGACTTCTGCTGGGT 120
PLCal_ZV            ---------------------------------------------------CTGCTGGGT 9
SV0127/14           GTAGCCCGTGTGAGCCCCTTTGGGGGCTTGAAGAGGCTGCCAGCCGGACTTCTGCTGGGT 120
8375                GTAGCCCGTGTGAGCCCCTTTGGGGGCTTGAAGAGGCTGCCAGCCGGACTTCTGCTGGGT 120
103344              GTAGCCCGTGTGAGCCCCTTTGGGGGCTTGAAGAGGCTGCCAGCCGGACTTCTGCTGGGT 120
BrasilZKV2015       GTAGCCCGTGTGAGCCCCTTTGGGGGCTTGAAGAGGCTGCCAGCCGGACTTCTGCTGGGT 120
GD01                GTAGCCCGTGTGAGCCCCTTTGGGGGCTTGAAGAGGCTGCCAGCCGGACTTCTGCTGGGT 120
Martinique          GTAGCCCGTGTGAGCCCCTTTGGGGGCTTGAAGAGGCTGCCAGCCGGACTTCTGCTGGGT 120
NatalRGN            GTAGCCCGTGTGAGCCCCTTTGGGGGCTTGAAGAGGCTGCCAGCCGGACTTCTGCTGGGT 120
HPF2013             GTAGCCCGTGTGAGCCCCTTTGGGGGCTTGAAGAGGCTGCCAGCCGGACTTCTGCTGGGT 120
SPH2015             GTAGCCCGTGTGAGCCCCTTTGGGGGCTTGAAGAGGCTGCCAGCCGGACTTCTGCTGGGT 120
Haiti2014           GTAGCCCGTGTGAGCCCCTTTGGGGGCTTGAAGAGGCTGCCAGCCGGACTTCTGCTGGGT 120
PRVABC59            GTAGCCCGTGTGAGCCCCTTTGGGGGCTTGAAGAGGCTGCCAGCCGGACTTCTGCTGGGT 120
BeH819015           GTAGCCCGTGTGAGCCCCTTTGGGGGCTTGAAGAGGCTGCCAGCCGGACTTCTGCTGGGT 120
Z1106033            GTAGCCCGTGTGAGCCCCTTTGGGGGCTTGAAGAGGCTGCCAGCCGGACTTCTGCTGGGT 120
BEH819966           GTAGCCCGTGTGAGCCCCTTTGGGGGCTTGAAGAGGCTGCCAGCCGGACTTCTGCTGGGT 120
SSABr               GTAGCCCGTGTGAGCCCCTTTGGGGGCTTGAAGAGGCTGCCAGCCGGACTTCTGCTGGGT 120
Beh815744           GTAGCCCGTGTGAGCCCCTTTGGGGGCTTGAAGAGGCTGCCAGCCGGACTTCTGCTGGGT 120
BEH818995           GTAGCCCGTGTGAGCCCCTTTGGGGGCTTGAAGAGGCTGCCAGCCGGACTTCTGCTGGGT 120
IbH30656            GTAGCCCGTGTAAACCCCTTGGGGGGTTTGAAGAGGCTGCCGGCCGGACTCCTGCTGGGC 120
ArD128000           GTAGCCCGTGTAAACCCCTTGGGGGGTTTGAAGAGGCTGCCAGCCGGACTTCTGCTGGGT 120
ArD7117             GTAGCCCGTGTAAACCCCTTGGGGGGTTTGAAGAGGCTGCCGGCCGGACTTCTGCTGGGC 120
ARD_41519           GTAGCCCGTGTAAACCCCTTGGGGGGTTTGAAGAGGCTGCCGGCCGGACTTCTGCTGGGC 120
ARD157995           GTAGCCCGTGTAAACCCCTTGGGAGGTTTGAAGAGGTTGCCAGCCGGACTTCTGCTGGGT 120
MR_766              GTAGCCCGTGTAAACCCCTTGGGAGGTTTGAAGAGGTTGCCAGCCGGACTTCTGCTGGGT 120
```

```
ARD158084          GTAGCCCGTGTAAACCCCTTGGGAGGTTTGAAGAGGTTGCCAGCCGGACTTCTGCTGGGT 120
ArB1362            GTAGCCCGTGTAAACCCCTTGGGAGGTTTGAAGAGGCTGCCAGCCGGACTTCTGCTGGGT 120
ARB13565           GTAGCCCGTGTAAACCCCTTGGGAGGTTTGAAGAGGCTGCCAGCCGGACTTCTGCTGGGT 120
ARB7701            GTAGCCCGTGTAAACCCCTTGGGAGGTTTGAAGAGGCTGCCAGCCGGACTTCTGCTGGGT 120
ARB15076           GTAGCCCGTGTAAACCCCTTGGGAGGTTTGAAGAGGCTGCCAGCCGGACTCCTGCTGGGT 120
                                                                      ********

P6-740             CATGGACCCATCAGGATGGTCTTGGCGATACTAGCCTTCTTGAGATTCACGGCAATCAAG 180
CPC0740            CATGGGCCCATCAGGATGGTCTTGGCGATACTAGCCTTTTTGAGATTCACGGCAATCAAG 180
YAP2007            CATGGGCCCATCAGGATGGTCTTGGCGATACTAGCCTTTTTGAGATTCACGGCAATCAAG 180
FSS13025           CATGGGCCCATCAGGATGGTCTTGGCGATTCTAGCCTTTTTGAGATTCACGGCAATCAAG 180
PLCal_ZV           CATGGGCCCATCAGGATGGTCTTGGCGATTCTAGCCTTTTTGAGATTCACGGCAATCAAG 69
SV0127/14          CATGGGCCCATCAGGATGGTCTTGGCGATTCTAGCCTTTTTGAGATTCACGGCAATCAAG 180
8375               CATGGGCCCATCAGGATGGTCTTGGCGATTCTAGCCTTTTTGAGATTCACGGCAATCAAG 180
103344             CATGGGCCCATCAGGATGGTCTTGGCGATTCTAGCCTTTTTGAGATTCACGGCAATCAAG 180
BrasilZKV2015      CATGGGCCCATCAGGATGGTCTTGGCGATTCTAGCCTTTTTGAGATTCACGGCAATCAAG 180
GD01               CATGGGCCCATCAGGATGGTCTTGGCGATTCTAGCCTTTTTGAGATTCACGGCAATCAAG 180
Martinique         CATGGGCCCATCAGGATGGTCTTGGCGATTCTAGCCTTTTTGAGATTCACGGCAATCAAG 180
NatalRGN           CATGGGCCCATCAGGATGGTCTTGGCAATTCTAGCCTTTTTGAGATTCACGGCAATCAAG 180
HPF2013            CATGGGCCCATCAGGATGGTCTTGGCGATTCTAGCCTTTTTGAGATTCACGGCAATCAAG 180
SPH2015            CATGGGCCCATCAGGATGGTCTTGGCAATTCTAGCCTTTTTGAGATTCACGGCAATCAAG 180
Haiti2014          CATGGGCCCATCAGGATGGTCTTGGCAATTCTAGCCTTTTTGAGATTCACGGCAATCAAG 180
PRVABC59           CATGGGCCCATCAGGATGGTCTTGGCGATTCTAGCCTTTTTGAGATTCACGGCAATCAAG 180
BeH819015          CATGGGCCCATCAGGATGGTCTTGGCGATTCTAGCCTTTTTGAGATTCACGGCAATCAAG 180
Z1106033           CATGGGCCCATCAGGATGGTCTTGGCGATTCTAGCCTTTTTGAGATTCACGGCAATCAAG 180
BEH819966          CATGGGCCCATCAGGATGGTCTTGGCGATTCTAGCCTTTTTGAGATTCACGGCAATCAAG 180
SSABr              CATGGGCCCATCAGGATGGTCTTGGCGATTCTAGCCTTTTTGAGATTCACGGCAATCAAG 180
Beh815744          CATGGGCCCATCAGGATGGTCTTGGCGATTCTAGCCTTTTTGAGATTCACGGCAATCAAG 180
BEH818995          CATGGGCCCATCAGGATGGTCTTGGCGATTCTAGCCTTTTTGAGATTCACGGCAATCAAG 180
IbH30656           CATGGACCCATCAGAATGGTTTTGGCGATACTAGCCTTCTTGAGATTCACAGCAATCAAG 180
ArD128000          CATGGACCCATTAGAATGGTTTTGGCGATACTAGCCTTTTTGAGATTTACAGCAATCAAG 180
ArD7117            CATGGACCCATCAGAATGGTTTTGGCGATACTAGCCTTTTTGAGATTCACAGCAATCAAG 180
ARD_41519          CATGGACCCATCAGAATGGTTTTGGCGATACTAGCCTTCTTGAGATTCACAGCAATCAAG 180
ARD157995          CATGGACCCATCAGAATGGTTTTGGCGATACTAGCCTTTTTGAGATTTACAGCAATCAAG 180
MR_766             CATGGACCCATCAGAATGGTTTTGGCGATACTAGCCTTTTTGAGATTTACAGCAATCAAG 180
ARD158084          CATGGACCCATCAGAATGGTTTTGGCGATACTAGCCTTTTTGAGATTTACAGCAATCAAG 180
ArB1362            CATGGACCCATTAGAATGGTTTTGGCGATACTAGCCTTTTTGAGATTTACAGCAATCAAG 180
ARB13565           CATGGACCCATCAGAATGGTTTTGGCAATACTAGCCTTTTTGAGATTTACAGCAATCAAG 180
ARB7701            CATGGACCCATCAGAATGGTTTTGGCAATACTAGCCTTTTTGAGATTTACAGCAATCAAG 180
ARB15076           CATGGACCCATCAGAATGGTTTTGGCAATACTAGCCTTTTTGAGATTTACAGCAATCAAG 180
                   ***** ***** ** ***** ***** ** ******** ******** ** *********

P6-740             CCATCACTGGGTCTCATCAATAGATGGGGTTCCGTGGGGAAAAAAGAGGCTATGGAAATA 240
CPC0740            CCATCACTGGGTCTCATCAATAGATGGGGTTCAGTGGGGAAAAAAGAGGCTATGGAAATA 240
YAP2007            CCATCACTGGGTCTCATCAATAGATGGGGTTCAGTGGGGAAAAAAGAGGCTATGGAAATA 240
FSS13025           CCATCACTGGGTCTCATCAATAGATGGGGTTCAGTGGGGAAAAAAGAGGCTATGGAAATA 240
PLCal_ZV           CCATCACTGGGTCTCATCAATAGATGGGGTTCAGTGGGGAAAAAAGAGGCTATGGAAATA 129
SV0127/14          CCATCACTGGGTCTCATCAATAGATGGGGTTCAGTGGGAAAAAAAGAGGCTATGGAAATA 240
8375               CCATCACTGGGTCTCATCAATAGATGGGGTTCAGTGGGGAAAAAAGAGGCTATGGAAATA 240
103344             CCATCACTGGGTCTCATCAATAGATGGGGTTCAGTGGGGAAAAAAGAGGCTATGGAAATA 240
BrasilZKV2015      CCATCACTGGGTCTCATCAATAGATGGGGTTCAGTGGGGAAAAAAGAGGCTATGGAAATA 240
GD01               CCATCACTGGGTCTCATCAATAGATGGGGTTCAGTGGGGAAAAAAGAGGCTATGGAAATA 240
Martinique         CCATCACTGGGTCTCATCAATAGATGGGGTTCAGTGGGGAAAAAAGAGGCTATGGAAATA 240
NatalRGN           CCATCACTGGGTCTCATCAATAGATGGGGTTCAGTGGGGAAAAAAGAGGCTATGGAAATA 240
HPF2013            CCATCACTGGGTCTCATCAATAGATGGGGTTCAGTGGGGAAAAAAGAGGCTATGGAAATA 240
SPH2015            CCATCACTGGGTCTCATCAATAGATGGGGTTCAGTGGGGAAAAAAGAGGCTATGGAAATA 240
Haiti2014          CCATCACTGGGTCTCATCAATAGATGGGGTTCAGTGGGGAAAAAAGAGGCTATGGAAATA 240
PRVABC59           CCATCACTGGGTCTCATCAATAGATGGGGTTCAGTGGGGAAAAAAGAGGCTATGGAAACA 240
BeH819015          CCATCACTGGGTCTCATCAATAGATGGGGTTCAGTTGGGAAAAAAGAGGCTATGGAAATA 240
Z1106033           CCATCACTGGGTCTCATCAATAGATGGGGTTCAGTGGGGAAAAAAGAGGCTATGGAAATA 240
BEH819966          CCATCACTGGGTCTCATCAATAGATGGGGTTCAGTGGGGAAAAAAGAGGCTATGGAAATA 240
SSABr              CCATCACTGGGTCTCATCAATAGATGGGGTTCAGTGGGGAAAAAAGAGGCTATGGAAATA 240
Beh815744          CCATCACTGGGTCTCATCAATAGATGGGGTTCAGTGGGGAAAAAAGAGGCTATGGAAATA 240
BEH818995          CCATCACTGGGTCTCATCAATAGATGGGGTTCAGTGGGGAAAAAAGAGGCTATGGAAATA 240
IbH30656           CCATCACTGGGCCTCATCAATAGATGGGGTTCCGTGGGGAAGAAGGAGGCTATGGAAATA 240
ArD128000          CCATCACTGGGCCTCATCAATAGATGGGGTTCAGTGGGGAAAAAAGAGGCTATGGAGATA 240
ArD7117            CCATCACTGGGCCTCATCAATAGATGGGGTTCCGTGGGGAAGAAGGAGGCTATGGAAATA 240
ARD_41519          CCATCACTGGGCCTCATCAATAGATGGGGTTCCGTGGGGAAGAAGGAGGCTATGGAAATA 240
```

```
ARD157995        CCATCACTGGGCCTTATCAACAGATGGGGTTCCGTGGGGAAAAAAGAGGCTATGGAAATA 240
MR_766           CCATCACTGGGCCTTATCAACAGATGGGGTTCCGTGGGGAAAAAAGAGGCTATGGAAATA 240
ARD158084        CCATCACTGGGCCTTATCAACAGATGGGGTTCCGTGGGGAAAAAAGAGGCTATGGAAATA 240
ArB1362          CCATCACTGGGCCTCATCAATAGATGGGGTTCAGTGGGGAAAAAAGAGGCTATGGAGATA 240
ARB13565         CCATCACTGGGCCTCATCAACAGATGGGGTTCTGTGGGGAAAAAAGAGGCTATGGAAATA 240
ARB7701          CCATCACTGGGCCTCATCAACAGATGGGGTTCTGTGGGGAAAAAAGAGGCTATGGAAATA 240
ARB15076         CCATCACTGGGCCTCATCAACAGATGGGGTTCAGTGGGGAAAAAAGAGGCTATGGAAATA 240
                 *********** ** ***** *********** ** ** ** ** *********** * *

P6-740           ATAAAGAAGTTCAAGAAAGATCTGGCTGCCATGCTGAGAATAATCAATGCTAGGAAGGAG 300
CPC0740          ATAAAGAAGTTCAAGAAAGATCTGGCTGCCATGCTGAGAATAATCAATGCTAGGAAGGAG 300
YAP2007          ATAAAGAAGTTCAAGAAAGATCTGGCTGCCATGCTGAGAATAATCAATGCTAGGAAGGAG 300
FSS13025         ATAAAGAAGTTTAAGAAAGATCTGGCTGCCATGCTGAGAATAATCAATGCTAGGAAGGAG 300
PLCal_ZV         ATAAAGAAGTTCAAGAAAGATCTGGCTGCCATGCTGAGAATAATCAATGCCAGGAAGGAG 189
SV0127/14        ATAAAGAAGTTCAAGAAAGATCTGGCTGCCATGCTGAGAATAATCAATGCTAGGAAGGAG 300
8375             ATAAAGAAGTTCAAGAAAGATCTGGCTGCCATGCTGAGAATAATCAATGCTAGGAAGGAG 300
103344           ATAAAGAAGTTCAAGAAAGATCTGGCTGCCATGCTGAGAATAATCAATGCTAGGAAGGAG 300
BrasilZKV2015    ATAAAGAAGTTCAAGAAAGATCTGGCTGCCATGCTGAGAATAATCAATGCCAGGAAGGAG 300
GD01             ATAAAGAAGTTCAAGAAAGATCTGGCTGCCATGCTGAGAATAATCAATGCTAGGAAGGAG 300
Martinique       ATAAAGAAGTTCAAGAAAGATCTGGCTGCCATGCTGAGAATAATCAATGCTAGGAAGGAG 300
NatalRGN         ATAAAGAAGTTCAAGAAAGATCTGGCTGCCATGCTGAGAATAATCAATGCTAGGAAGGAG 300
HPF2013          ATAAAGAAGTTCAAGAAAGATCTGGCTGCCATGCTGAGAATAATCAATGCTAGGAAGGAG 300
SPH2015          ATAAAGAAGTTCAAGAAAGATCTGGCTGCCATGCTGAGAATAATCAATGCTAGGAAGGAG 300
Haiti2014        ATAAAGAAGTTCAAGAAAGATCTGGCTGCCATGCTGAGAATAATCAATGCTAGGAAGGAG 300
PRVABC59         ATAAAGAAGTTCAAGAAAGATCTGGCTGCCATGCTGAGAATAATCAATGCTAGGAAGGAG 300
BeH819015        ATAAAGAAGTTCAAGAAAGATCTGGCTGCCATGCTGAGAATAATCAATGCTAGGAAGGAG 300
Z1106033         ATAAAGAAGTTCAAGAAAGATCTGGCTGCCATGCTGAGAATAATCAATGCTAGGAAGGAG 300
BEH819966        ATAAAGAAGTTCAAGAAAGATCTGGCTGCCATGCTGAGAATAATCAATGCTAGGAAGGAG 300
SSABr            ATAAAGAAGTTCAAGAAAGATCTGGCTGCCATGCTGAGAATAATCAATGCTAGGAAGGAG 300
Beh815744        ATAAAGAAGTTCAAGAAAGATCTGGCTGCCATGCTGAGAATAATCAATGCTAGGAAGGAG 300
BEH818995        ATAAAGAAGTTCAAGAAAGATCTGGCTGCCATGCTGAGAATAATCAATGCTAGGAAGGAG 300
IbH30656         ATAAAAAAGTTCAAGAAAGATCTTGCTGCCATGTTGAGAATAATCAATGCTAGGAAGGAG 300
ArD128000        ATAAAGAAGTTTAAGAAAGATCTTGCTGCCATGTTGAGAATAATCAATGCTAGGAAGGAG 300
ArD7117          ATAAAAAAGTTCAAGAAAGACCTTGCTGCCATGTTGAGAATTATCAATGCTAGGAAGGAG 300
ARD_41519        ATAAAAAAGTTCAAGAAAGACCTTGCTGCCATGTTGAGAATCATCAATGCTAGGAAGGAG 300
ARD157995        ATAAAGAAGTTCAAGAAAGATCTTGCTGCCATGTTGAGAATAATCAATGCTAGGAAAGAG 300
MR_766           ATAAAGAAGTTCAAGAAAGATCTTGCTGCCATGTTGAGAATAATCAATGCTAGGAAAGAG 300
ARD158084        ATAAAGAAGTTCAAGAAAGATCTTGCTGCCATGTTGAGAATAATCAATGCTAGGAAAGAG 300
ArB1362          ATAAAGAAGTTTAAGAAAGATCTTGCTGCCATGTTGAGAATAATCAATGCTAGGAAGGAG 300
ARB13565         ATAAAGAAGTTTAAGAAAGATCTTGCTGCCATGTTGAGAATAATCAATGCTAGGAAGGAG 300
ARB7701          ATAAAGAAGTTTAAGAAAGATCTTGCTGCCATGTTGAGAATAATCAATGCTAGGAAGGAG 300
ARB15076         ATAAAGAAGTTTAAGAAAGATCTTGCTGCCATGTTGAGAATAATCAATGCTAGGAAGGAG 300
                 ***** ***** ******** ** ********* ******* ******** ***** ***

P6-740           AAGAAGAGACGTGGCGCAGACACCAGTGTCGGAATTGTTGGCCTCCTGCTGACCACAGCC 360
CPC0740          AAGAAGAGACGAGGCGCAGATACTAGCGTCGGAATTGTTGGCCTCCTCCTGACCACAGCC 360
YAP2007          AAGAAGAGACGAGGCACAGATACTAGTGTCGGAATTGTTGGCCTCCTGCTGACCACAGCC 360
FSS13025         AAGAAGAGACGAGGCACAGATACTAGTGTCGGAATTGTTGGCCTCCTGCTGACCACAGCC 360
PLCal_ZV         AAGAAGAGACGAGGCACAGACACTAGTGTCGGAATTGTTGGCCTCCTGCTGACCACAGCC 249
SV0127/14        AAGAAGAGACGAGGCACAGATACTAGTGTCGGAATTGTTGGCCTCCTGCTGACCACAGCT 360
8375             AAGAAGAGACGAGGCGCAGATACTAGTGTCGGAATTGTTGGCCTCCTGCTGACCACAGCT 360
103344           AAGAAGAGACGAGGCGCAGATACTAGTGTCGGAATTGTTGGCCTCCTGCTGACCACAGCT 360
BrasilZKV2015    AAGAAGAGACGAGGCGCAGATACTAGTGTCGGAATCGTTGGCCTCCTGCTGACCACAGCT 360
GD01             AAGAAGAGACGAGGCGCAGAAACTAGTGTCGGAATTGTTGGCCTCCTGCTGACCACAGCT 360
Martinique       AAGAAGAGACGAGGCGCAGAAACTAGTGTCGGAATTGTTGGCCTCCTGCTGACCACAGCT 360
NatalRGN         AAGAAGAGACGAGGCGCAGATACTAGTGTCGGAATTGTTGGCCTCCTGCTGACCACAGCT 360
HPF2013          AAGAAGAGACGAGGCGCAGATACTAGTGTCGGAATTGTTGGCCTCCTGCTGACCACAGCT 360
SPH2015          AAGAAGAGACGGGGCGCAGATACTAGTGTCGGAATTGTTGGCCTCCTGCTGACCACAGCT 360
Haiti2014        AAGAAGAGACGAGGCGCAGATACTAGTGTCGGAATTGTTGGCCTCCTGCTGACCACAGCT 360
PRVABC59         AAGAAGAGACGAGGCGCAGATACTAGTGTCGGAATTGTTGGCCTCCTGCTGACCACAGCT 360
BeH819015        AAGAAGAGACGAGGCGCAGATACTAGTGTCGGAATTGTTGGCCTCCTGCTGACCACAGCT 360
Z1106033         AAGAAGAGACGAGGCGCAGATACTAGTGTCGGAATTGTTGGCCTCCTGCTGACCACAGCT 360
BEH819966        AAGAAGAGACGAGGCGCAGATACTAGTGTCGGAATTGTTGGCCTCCTGCTGACCACAGCT 360
SSABr            AAGAAGAGACGAGGCGCAGATACTAGTGTCGGAATTGTTGGCCTCCTGCTGACCACAGCT 360
Beh815744        AAGAAGAGACGAGGCGCAGATACTAGTGTCGGAATTGTTGGCCTCCTGCTGACCACAGCT 360
BEH818995        AAGAAGAGACGAGGCGCAGATACTAGTGTCGGAATTGTTGGCCTCCTGCTGACCACAGCT 360
IbH30656         AGGAAGAGACGTGGAGCTGATGCCAGCATCGGAATCGTCAGCCTCCTGCTGACTACAGTC 360
ArD128000        AGGAAGAGACGTGGTGCAGACACCAGTATCGGAATCATTGGCCTCCTGCTGACTACAGCC 360
```

```
ArD7117             AGGAAGAGACGTGGCGCTGACACCAGCATCGGAATCGTCGGCCTCCTGCTGACCACAGCC 360
ARD_41519           AGGAAGAGACGTGGCGCTGACACCAGCATCGGAATCGTCGGCCTCCTGCTGACCACAGCC 360
ARD157995           AGGAAGAGACGTGGCGCAGACACCAGCATCGGAATCATTGGCCTCCTGCTGACTACAGCC 360
MR_766              AGGAAGAGACGTGGCGCAGACACCAGCATCGGAATCATTGGCCTCCTGCTGACTACAGCC 360
ARD158084           AGGAAGAGACGTGGCGCAGACACCAGCATCGGAATCATTGGCCTCCTGCTGACTACAGCC 360
ArB1362             AGGAAGAGACGTGGTGCAGACACCAGTATCGGAATCATTGGCCTCCTGCTGACTACAGCC 360
ARB13565            AGGAAGAGACGTGGTGCAGACACCAGCATCGGAATCATTGGTCTCCTGCTGACTACAGCC 360
ARB7701             AGGAAGAGACGTGGTGCAGACACCAGCATCGGAATCATTGGTCTCCTGCTGACTACAGCC 360
ARB15076            AGGAAGAGACGTGGTGCAGACACCAGCATCGGAATCATTGGTCTCCTGCTGACTACAGCC 360
                    * ********* **  * **  * **  *******  *  * ***** ***** ****

P6-740              ATGGCAGTGGAGGTCACCAGACGTGGGAGTGCATACTATATGTACTTAGACAGAAGCGAT 420
CPC0740             ATGGCAGTAGAGGTCACTAGACGTGGGAGTGCATACTATATGTACTTGGACAGAAGCGAT 420
YAP2007             ATGGCAGTGGAGGTCACTAGACGTGGGAGTGCATACTATATGTACTTGGACAGAAGCGAT 420
FSS13025            ATGGCAGTGGAGGTCACTAGACGTGGGAATGCATACTATATGTACTTGGACAGAAGCGAT 420
PLCal_ZV            ATGGCAGCGGAGGTCACTAGACGTGGGAGTGCATACTATATGTACTTGGACAGAAGCGAT 309
SV0127/14           ATGGCAGCGGAGGTCACTAGACGTGGGAGTGCATACTATATGTACTTGGACAGAAGCGAT 420
8375                ATGGCAGCGGAGGTCACTAGACGTGGGAGTGCATACTATATGTACTTGGACAGAAACGAT 420
103344              ATGGCAGCGGAGGTCACTAGACGTGGGAGTGCATACTATATGTACTTGGACAGAAACGAT 420
BrasilZKV2015       ATGGCAGCGGAGGTCACTAGACGTGGGAGTGCATACTATATGTACTTGGACAGAAACGAT 420
GD01                ATGGCAGCGGAGGTCACTAGACGTGGGAGTGCATACTATATGTACTTGGACAGAAACGAT 420
Martinique          ATGGCAGCGGAGGTCACTAGACGTGGGAGTGCATACTATATGTACTTGGACAGAAACGAT 420
NatalRGN            ATGGCAGCGGAGGTCACTAGACGTGGGAGTGCATACTATATGTACTTGGACAGAAACGAT 420
HPF2013             ATGGCAGCGGAGGTCACTAGACGTGGGAGTGCATACTATATGTACTTGGACAGAAACGAC 420
SPH2015             ATGGCAGCGGAGGTCACTAGACGTGGGAGTGCATACTATATGTACTTGGACAGAAACGAT 420
Haiti2014           ATGGCAGCGGAGGTCACTAGACGTGGGAGTGCATACTATATGTACTTGGACAGAAACGAT 420
PRVABC59            ATGGCAGCGGAGGTCACTAGACGTGGGAGTGCATACTATATGTACTTGGACAGAAACGAT 420
BeH819015           ATGGCAGCGGAGGTCACTAGACGTGGGAGTGCATACTATATGTACTTGGACAGAAACGAT 420
Z1106033            ATGGCAGCGGAGGTCACTAGACGTGGGAGTGCATACTATATGTACTTGGACAGAAACGAT 420
BEH819966           ATGGCAGCGGAGGTCACTAGACGTGGGAGTGCATACTATATGTACTTGGACAGAAACGAT 420
SSABr               ATGGCAGCGGAGGTCACTAGACGTGGGAGTGCATACTATATGTACTTGGACAGAAACGAT 420
Beh815744           ATGGCAGCGGAGGTCACTAGACGTGGGAGTGCATACTATATGTACTTGGACAGAAACGAT 420
BEH818995           ATGGCAGCGGAGGTCACTAGACGTGGGAGTGCATACTATATGTACTTGGACAGAAACGAT 420
IbH30656            ATGGCAGCAGAGATCACTAGACGCGGGAGTGCATACTACATGTACTTGGACAGGAGCGAT 420
ArD128000           ATGGCAGCAGAGATTACTAGACGAGGAAGTGCATACTACATGTACTTGGACAGGAGCGAT 420
ArD7117             ATGGCAGCCGAGATCACCAGACGTGGGAGTGCATACTACATGTACTTGGACAGGAGCGAT 420
ARD_41519           ATGGCAGCCGAGATCACTAGACGTGGGAGTGCATACTACATGTACTTGGACAGGAGCGAT 420
ARD157995           ATGGCAGCAGAGATCACTAGACGCGGGAGTGCATACTACATGTACTTGGATAGGAGCGAT 420
MR_766              ATGGCAGCAGAGATCACTAGACGCGGGAGTGCATACTACATGTACTTGGATAGGAGCGAT 420
ARD158084           ATGGCAGCAGAGATCACTAGACGCGGGAGTGCATACTACATGTACTTGGATAGGAGCGAT 420
ArB1362             ATGGCAGCAGAGATTACTAGACGAGGAAGTGCATACTACATGTACTTGGACAGGAGCGAT 420
ARB13565            ATGGCAGCGGAGATTACCAGACGTGGAAGTGCATACTACATGTACTTGGACAGGAGCGAT 420
ARB7701             ATGGCAGCGGAGATTACCAGACGTGGAAGTGCATACTACATGTACTTGGACAGGAGCGAT 420
ARB15076            ATGGCAGCAGAGATTACCAGACGTGGAAGTGCATACTACATGTACTTGGACAGGAGCGAT 420
                    *******  *** * ** ***** ** * ********* ******** ** ** * ***

P6-740              GCTGGGGAGGCCATATCTTTTCCAACCACACTGGGGGTGAATAAGTGTTACATACAGATC 480
CPC0740             GCTGGGGAGGCCATATCTTTTCCAACCACACTGGGGATGAATAAGTGTTACATACAAATC 480
YAP2007             GCTGGGGAGGCCATATCTTTTCCAACCACACTGGGGATGAACAAGTGTTACATACAGATC 480
FSS13025            GCTGGGGAGGCCATATCTTTTCCAACCACAATGGGGATGAATAAGTGTTATATACAGATC 480
PLCal_ZV            GCTGGGGAGGCCATATCTTTTCCAACCACACTGGGGATGAATAAGTGTTATATACAGATC 369
SV0127/14           GCTGGGGAGGCCATATCTTTTCCAACCACACTGGGGATGAATAAGTGTTATATACAGATC 480
8375                GCTGGGGAGGCCATATCTTTTCCAACCACATTGGGGATGAATAAGTGTTATATACAGATC 480
103344              GCTGGGGAGGCCATATCTTTTCCAACCACATTGGGGATGAATAAGTGTTATATACAGATC 480
BrasilZKV2015       GCTGGGGAGGCCATATCTTTTCCAACCACATTGGGGATGAATAAGTGTTATATACAGATC 480
GD01                GCTGGGGAGGCCATATCTTTTCCAACCACATTGGGGATGAATAAGTGTTATATACAGATC 480
Martinique          GCTGGGGAGGCCATATCTTTTCCAACCACATTGGGGATGAATAAGTGTTATATACAGATC 480
NatalRGN            GCTGGGGAGGCCATATCTTTTCCAACCACATTGGGGATGAATAAGTGTTATATACAGATC 480
HPF2013             GCTGGGGAGGCCATATCTTTTCCAACCACATTGGGGATGAATAAGTGTTATATACAGATC 480
SPH2015             GCTGGGGAGGCCATATCTTTTCCAACCACATTGGGGATGAATAAGTGTTATATACAGATC 480
Haiti2014           GCTGGGGAGGCCATATCTTTTCCAACCACATTGGGGATGAATAAGTGTTATATACAGATC 480
PRVABC59            GCTGGGGAGGCCATATCTTTTCCAACCACATTGGGGATGAATAAGTGTTATATACAGATC 480
BeH819015           GCTGGGGAGGCCATATCTTTTCCAACCACATTGGGGATGAATAAGTGTTATATACAGATC 480
Z1106033            GCTGGGGAGGCCATATCTTTTCCAACCACATTGGGGATGAATAAGTGTTATATACAGATC 480
BEH819966           GCTGGGGAGGCCATATCTTTTCCAACCACATTGGGGATGAATAAGTGTTATATACAGATC 480
SSABr               GCTGGGGAGGCCATATCTTTTCCAACCACATTGGGGATGAATAAGTGTTATATACAGATC 480
Beh815744           GCTGGGGAGGCCATATCTTTTCCAACCACATTGGGGATGAATAAGTGTTATATACAGATC 480
BEH818995           GCTGGGGAGGCCATATCTTTTCCAACCACATTGGGGATGAATAAGTGTTATATACAGATC 480
```

```
IbH30656           GCTGGTAAGGCCATTTCTTTCGTTACCACACTGGGGGTGAACAAATGCCATGTGCAGATC 480
ArD128000          GCTGGGAAGGCCATTTCCTTTGCTACCACATTGGGAGTGAACAAATGCCATGTACAGATC 480
ArD7117            GCTGGTAAGGCCATTTCTTTTGCTACCACATTGGGGGTGAACAAATGCCATGTACAGATC 480
ARD_41519          GCTGGTAAGGCCATTTCTTTTGCCACCACATTGGGGGTGAACAAATGCCATGTACAGATC 480
ARD157995          GCCGGGAAGGCCATTTCGTTTGCTACCACATTGGGAGTGAACAAGTGCCACGTACAGATC 480
MR_766             GCCGGGAAGGCCATTTCGTTTGCTACCACATTGGGAGTGAACAAGTGCCACGTACAGATC 480
ARD158084          GCCGGGAAGGCCATTTCGTTTGCTACCACATTGGGAGTGAACAAGTGCCACGTACAGATC 480
ArB1362            GCTGGGAAGGCCATTTCCTTTGCTACCACATTGGGAGTGAACAAATGCCATGTACAGATC 480
ARB13565           GCTGGGAAGGCCATCTCCTTTGCTACCAACTTGGGAGTTAACAAATGCCATGTACAGATC 480
ARB7701            GCTGGGAAGGCCATCTCCTTTGCTACCAACTTGGGAGTTAACAAATGCCATGTACAGATC 480
ARB15076           GCTGGGAAGGCCATCTCCTTTGCTACCAATTTGGGAGTTAACAAATGCCATGTACAGATC 480
                   ** **  ******* ** **    ****   ****  * ** ** **  *  * ** ***

P6-740             ATGGATCTTGGACACATGTGTGATGCCACAATGAGCTATGAATGCCCTATGTTGGATGAG 540
CPC0740            ATGGATCTTGGACACATGTGTGATGCCACCATGAGCTATGAATGCCCTATGTTGGATGAG 540
YAP2007            ATGGATCTTGGACACATGTGTGATGCCACCATGAGCTATGAATGCCCTATGTTGGATGAG 540
FSS13025           ATGGATCTTGGACACATGTGTGATGCCACCATGAGCTATGAATGCCCTATGCTGGATGAG 540
PLCal_ZV           ATGGATCTTGGACACATGTGTGATGCCACCATGAGCTATGAATGCCCTATGCTGGATGAG 429
SV0127/14          ATGGATCTTGGACACATGTGTGATGCCACCATGAGCTATGAATGCCCTATGCTGGATGAG 540
8375               ATGGATCTTGGACACATGTGTGATGCCACCATGAGCTATGAATGCCCTATGCTGGATGAG 540
103344             ATGGATCTTGGACACATGTGTGATGCCACCATGAGCTATGAATGCCCTATGCTGGATGAG 540
BrasilZKV2015      ATGGATCTTGGACACATGTGTGATGCCACCATGAGCTATGAATGCCCTATGCTGGATGAG 540
GD01               ATGGATCTTGGACACATGTGTGATGCCACCATGAGCTATGAATGCCCTATGCTGGATGAG 540
Martinique         ATGGATCTTGGACACATGTGTGATGCCACCATGAGCTATGAATGCCCTATGCTGGATGAG 540
NatalRGN           ATGGATCTTGGACACATGTGTGATGCCACCATGAGCTATGAATGCCCTATGCTGGATGAG 540
HPF2013            ATGGATCTTGGACACATGTGTGATGCCACCATGAGCTATGAATGCCCTATGCTGGATGAG 540
SPH2015            ATGGATCTTGGACACATGTGTGATGCCACCATGAGCTATGAATGCCCTATGCTGGATGAG 540
Haiti2014          ATGGATCTTGGACACATGTGTGATGCCACCATGAGCTATGAATGCCCTATGCTGGATGAG 540
PRVABC59           ATGGATCTTGGACACATGTGTGATGCCACCATGAGCTATGAATGCCCTATGCTGGATGAG 540
BeH819015          ATGGATCTTGGACACATGTGTGATGCCACCATGAGCTATGAATGCCCTATGCTGGATGAG 540
Z1106033           ATGGATCTTGGACACACGTGTGATGCCACCATGAGCTATGAATGCCCTATGCTGGATGAG 540
BEH819966          ATGGATCTTGGACACATGTGTGATGCCACCATGAGCTATGAATGCCCTATGCTGGATGAG 540
SSABr              ATGGATCTTGGACACATGTGTGATGCCACCATGAGCTATGAATGCCCTATGCTGGATGAG 540
Beh815744          ATGGATCTTGGACACATGTGTGATGCCACCATGAGCTATGAATGCCCTATGCTGGATGAG 540
BEH818995          ATGGATCTTGGACACATGTGTGATGCCACCATGAGCTATGAATGCCCTATGCTGGATGAG 540
IbH30656           ATGGACCTCGGGCATATGTGTGACGCCACCATGAGTTATGAGTGCCCCATGCTGGACGAG 540
ArD128000          ATGGATCTCGGGCACATGTGTGACGCCACCATGAGTTATGAGTGCCCTATGCTAGACGAA 540
ArD7117            ATGGACCTCGGGCACATGTGTGACGCCACCATGAGTTATGAGTGCCCCATGCTGGACGAG 540
ARD_41519          ATGGACCTCGGGCACATGTGTGACGCCACCATGAGTTATGAGTGCCCCATGCTAGACGAG 540
ARD157995          ATGGACCTCGGGCACATGTGTGACGCCACCATGAGTTATGAGTGCCCTATGCTGGATGAG 540
MR_766             ATGGACCTCGGGCACATGTGTGACGCCACCATGAGTTATGAGTGCCCTATGCTGGATGAG 540
ARD158084          ATGGACCTCGGGCACATGTGTGACGCCACCATGAGTTATGAGTGCCCTATGCTGGATGAG 540
ArB1362            ATGGATCTCGGGCACATGTGTGACGCCACCATGAGTTATGAGTGCCCTATGCTAGACGAA 540
ARB13565           ATGGATCTCGGGCACATGTGTGACGCCACCATGAGTTATGAGTGCCCTATGTTGGACGAG 540
ARB7701            ATGGATCTCGGGCACATGTGTGACGCCACCATGAGTTATGAGTGCCCTATGTTGGACGAG 540
ARB15076           ATGGATCTCGGGCACATGTGTGACGCCACCATGAGTTATGAGTGTCCTATGTTGGACGAG 540
                   ***** ** ** ** * ****** ***** ***** ***** ** ** *** * ** **

P6-740             GGGGTAGAACCAGATGACGTCGATTGCTGGTGCAACACGACATCGACTTGGGTTGTGTAC 600
CPC0740            GGGGTAGAACCAGATGACGTCGATTGCTGGTGCAACACGACATCAACTTGGGTTGTGTAT 600
YAP2007            GGGGTAGAACCAGATGACGTCGATTGTTGGTGCAACACGACATCAACTTGGGTTGTGTAC 600
FSS13025           GGGGTAGAACCAGATGACGTCGATTGTTGGTGCAACACGACGTCAACTTGGGTTGTGTAC 600
PLCal_ZV           GGGGTAGAACCAGATGACGTCGATTGTTGGTGCAACACGACGTCAACTTGGGTTGTGTAC 489
SV0127/14          GGGGTAGAACCAGATGACGTCGATTGTTGGTGCAACACGACGTCAACTTGGGTTGTGTAC 600
8375               GGGGTGGAACCAGATGACGTCGATTGTTGGTGCAACACGACGTCAACTTGGGTTGTGTAC 600
103344             GGGGTGGAACCAGATGACGTCGATTGTTGGTGCAACACGACGTCAACTTGGGTTGTGTAC 600
BrasilZKV2015      GGGGTGGAACCAGATGACGTCGATTGTTGGTGCAACACGACGTCAACTTGGGTTGTGTAC 600
GD01               GGGGTGGAACCAGATGACGTCGATTGTTGGTGCAACACGACGTCAACTTGGGTTGTGTAC 600
Martinique         GGGGTGGAACCAGATGACGTCGATTGTTGGTGCAACACGACGTCAACTTGGGTTGTGTAC 600
NatalRGN           GGGGTGGAACCAGATGACGTCGATTGTTGGTGCAACACGACGTCAACTTGGGTTGTGTAC 600
HPF2013            GGGGTGGAACCAGATGACGTCGATTGTTGGTGCAACACGACGTCAACTTGGGTTGTGTAC 600
SPH2015            GGGGTGGAACCAGATGACGTCGATTGTTGGTGCAACACGACGTCAACTTGGGTTGTGTAC 600
Haiti2014          GGGGTGGAACCAGATGACGTCGATTGTTGGTGCAACACGACGTCAACTTGGGTTGTGTAC 600
PRVABC59           GGGGTGGAACCAGATGACGTCGATTGTTGGTGCAACACGACGTCAACTTGGGTTGTGTAC 600
BeH819015          GGGGTGGAACCAGATGACGTCGATTGTTGGTGCAACACGACGTCAACTTGGGTTGTGTAC 600
Z1106033           GGGGTGGAACCAGATGACGTCGATTGTTGGTGCAACACGACGTCAACTTGGGTTGTGTAC 600
BEH819966          GGGGTGGAACCAGATGACGTCGATTGTTGGTGCAACACGACGTCAACTTGGGTTGTGTAC 600
SSABr              GGGGTGGAACCAGATGACGTCGATTGTTGGTGCAACACGACGTCAACTTGGGTTGTGTAC 600
```

```
Beh815744         GGGGTGGAACCAGATGACGTCGATTGTTGGTGCAACACGACGTCAACTTGGGTTGTGTAC 600
BEH818995         GGGGTGGAACCAGATGACGTCGATTGTTGGTGCAACACGACGTCAACTTGGGTTGTGTAC 600
IbH30656          GGAGTGGAGCCAGATGACGTCGATTGCTGGTGCAACACGACATCAACTTGGGTTGTGTAC 600
ArD128000         GGAGTGGAACCAGATGATGTCGATTGCTGGTGCAACACGACATCAACTTGGGTTGTGTAC 600
ArD7117           GGAGTGGAGCCAGATGACGTCGATTGCTGGTGCAACACGACATCAACTTGGGTTGTGTAC 600
ARD_41519         GGAGTGGAGCCAGATGACGTCGATTGCTGGTGCAACACGACATCGACTTGGGTTGTGTAC 600
ARD157995         GGAGTGGAACCAGATGATGTCGATTGCTGGTGCAACACGACATCAACTTGGGTTGTGTAC 600
MR_766            GGAGTGGAACCAGATGATGTCGATTGCTGGTGCAACACGACATCAACTTGGGTTGTGTAC 600
ARD158084         GGAGTGGAACCAGATGATGTCGATTGCTGGTGCAACACGACATCAACTTGGGTTGTGTAC 600
ArB1362           GGAGTGGAACCAGATGATGTCGATTGCTGGTGCAACACGACATCAACTTGGGTTGTGTAC 600
ARB13565          GGAGTGGAACCAGATGATGTCGATTGCTGGTGCAACACGACATCAACTTGGGTTGTGTAC 600
ARB7701           GGAGTGGAACCAGATGATGTCGATTGCTGGTGCAACACGACATCAACTTGGGTTGTGTAC 600
ARB15076          GGAGTGGAACCAGATGATGTCGATTGCTGGTGCAACACGACATCAACTTGGGTTGTGTAC 600
                  ** ** ** ******** ******** ************** ** **************

P6-740            GGAACCTGCCATCACAAAAAAGGTGAGGCACGGAGATCTAGAAGAGCTGTGACGCTCCCC 660
CPC0740           GGAACCTGCCACCACAAAAAAGGTGAAGCACGGAGATCTAGAAGAGCTGTGACGCTCCCC 660
YAP2007           GGAACCTGCCACCACAAAAAAGGTGAAGCACGGAGATCTAGAAGAGCTGTGACGCTCCCC 660
FSS13025          GGAACCTGCCACCACAAAAAAGGTGAAGCACGGAGATCTAGAAGAGCTGTGACGCTCCCC 660
PLCal_ZV          GGAACCTGCCACCACAAAAAAGGTGAAGCACGGAGATCTAGAAGAGCTGTGACGCTCCCC 549
SV0127/14         GGAACCTGCCATCACAAAAAAGGTGAAGCACGGAGATCCAGAAGAGCTGTGACGCTCCCC 660
8375              GGAACCTGCCATCACAAAAAAGGTGAAGCACGGAGATCTAGAAGAGCTGTGACGCTCCCC 660
103344            GGAACCTGCCATCACAAAAAAGGTGAAGCACGGAGATCTAGAAGAGCTGTGACGCTCCCC 660
BrasilZKV2015     GGAACCTGCCATCACAAAAAAGGTGAAGCACGGAGATCTAGAAGAGCTGTGACGCTCCCC 660
GD01              GGAACCTGCCATCACAAAAAAGGTGAAGCACGGAGATCTAGAAGAGCTGTGACGCTCCCT 660
Martinique        GGAACCTGCCATCACAAAAAAGGTGAAGCACGGAGATCTAGAAGAGCCGTGACGCTCCCC 660
NatalRGN          GGAACCTGCCATCACAAAAAAGGTGAAGCACGGAGATCTAGAAGAGCTGTGACGCTCCCC 660
HPF2013           GGAACCTGCCATCACAAAAAAGGTGAAGCACGGAGATCTAGAAGAGCTGTGACGCTCCCC 660
SPH2015           GGAACCTGCCATCACAAAAAAGGTGAAGCACGGAGATCTAGAAGAGCTGTGACGCTCCCC 660
Haiti2014         GGAACCTGCCATCACAAAAAAGGTGAAGCACGGAGATCTAGAAGAGCTGTGACGCTCCCC 660
PRVABC59          GGAACCTGCCATCACAAAAAAGGTGAAGCACGGAGATCTAGAAGAGCTGTGACGCTCCCC 660
BeH819015         GGAACCTGCCATCACAAAAAAGGTGAAGCACGGAGATCTAGAAGAGCTGTGACGCTCCCC 660
Z1106033          GGAACCTGCCATCACAAAAAAGGTGAAGCACGGAGATCTAGAAGAGCTGTGACGCTCCCC 660
BEH819966         GGAACCTGCCATCACAAAAAAGGTGAAGCACGGAGATCTAGAAGAGCTGTGACGCTCCCC 660
SSABr             GGAACCTGCCATCACAAAAAAGGTGAAGCACGGAGATCTAGAAGAGCTGTGACGCTCCCC 660
Beh815744         GGAACCTGCCATCACAAAAAAGGTGAAGCACGGAGATCTAGAAGAGCTGTGACGCTCCCC 660
BEH818995         GGAACCTGCCATCACAAAAAAGGTGAAGCACGGAGATCTAGAAGAGCTGTGACGCTCCCC 660
IbH30656          GGAACCTGTCATCATAAAAAAGGTGAAGCACGACGATCCAGAAGAGCCGTGACGCTTCCT 660
ArD128000         GGAACCTGTCATCACAAAAAAGGTGAAGCACGGCGATCTAGAAGAGCTGTGACGCTCCCT 660
ArD7117           GGAACCTGTCAACATAAAAAAGGTGAAGCACGACGATCCAGAAGAGCCGTGACGCTTCCT 660
ARD_41519         GGAACCTGTCATCATAAAAAAGGTGAAGCACGACGATCCAGAAGAGCCGTGACGCTTCCT 660
ARD157995         GGAACCTGTCATCACAAAAAAGGTGAGACACGGCGATCTAGAAGATCTGTGTCGCTCCGT 660
MR_766            GGAACCTGTCATCACAAAAAAGGTGAGGCACGGCGATCTAGAAGAGCCGTGACGCTCCCT 660
ARD158084         GGAACCTGTCATCACAAAAAAGGTGAGGCACGGCGATCTAGAAGAGCCGTGACGCTCCCT 660
ArB1362           GGAACCTGTCATCACAAAAAAGGTGAAGCACGGCGATCTAGAAGAGCTGTGACGCTCCCT 660
ARB13565          GGAACCTGTCATCACAAAAAAGGTGAAGCACGGCGATCTAGAAGAGCTGTGACGCTCCCT 660
ARB7701           GGAACCTGTCATCACAAAAAAGGTGAAGCACGGCGATCTAGAAGAGCTGTGACGCTCCCT 660
ARB15076          GGAACCTGTCATCACAAAAAAGGTGAAGCACGGCGATCTAGAAGAGCTGTGACGCTCCCT 660
                  ******** ** ** ***********  ****  **** ****** * *** **** *

P6-740            TCTCATTCCACTAGGAAGCTGCAAACGCGGTCGCAGACCTGGTTGGAATCAAGAGAATAC 720
CPC0740           TCCCATTCCACTAGGAAGCTGCAAACGCGGTCGCAGACCTGGTTGGAATCAAGAGAATAC 720
YAP2007           TCCCATTCCACTAGGAAGCTGCAAACGCGGTCGCAGACCTGGTTGGAATCAAGAGAATAT 720
FSS13025          TCCCATTCCACTAGGAAGCTGCAAACGCGGTCGCAGACCTGGTTGGAATCAAGAGAATAC 720
PLCal_ZV          TCCCACTCCACTAGGAAGCTGCAAACGCGGTCGCAGACCTGGTTGGAATCAAGAGAATAC 609
SV0127/14         TCCCATTCCACTAGGAAGCTGCAAACGCGGTCGCAGACCTGGTTGGAATCAAGAGAATAC 720
8375              TCCCATTCCACTAGGAAGCTGCAAACGCGGTCGCAAACCTGGTTGGAATCAAGAGAATAC 720
103344            TCCCATTCCACTAGGAAGCTGCAAACGCGGTCGCAAACCTGGTTGGAATCAAGAGAATAC 720
BrasilZKV2015     TCCCATTCCACTAGGAAGCTGCAAACGCGGTCGCAAACCTGGTTGGAATCAAGAGAATAC 720
GD01              TCCCATTCCACTAGGAAGCTGCAAACGCGGTCGCAAACCTGGTTGGAATCAAGAGAATAC 720
Martinique        TCCCATTCCACTAGGAAGCTGCAAACGCGGTCGCAAACCTGGTTGGAATCAAGAGAATAC 720
NatalRGN          TCCCATTCCACTAGGAAGCTGCAAACGCGGTCGCAAACCTGGTTGGAATCAAGAGAATAC 720
HPF2013           TCCCATTCCACTAGGAAGCTGCAAACGCGGTCGCAAACCTGGTTGGAATCAAGAGAATAC 720
SPH2015           TCCCATTCCACTAGGAAGCTGCAAACGCGGTCGCAAACCTGGTTGGAATCAAGAGAATAC 720
Haiti2014         TCCCATTCCACTAGGAAGCTGCAAACGCGGTCGCAAACCTGGTTGGAATCAAGAGAATAC 720
PRVABC59          TCCCATTCCACCAGGAAGCTGCAAACGCGGTCGCAAACCTGGTTGGAATCAAGAGAATAC 720
BeH819015         TCCCATTCCACTAGGAAGCTGCAAACGCGGTCGCAAACCTGGTTGGAATCAAGAGAATAC 720
Z1106033          TCCCATTCCACTAGGAAGCTGCAAACGCGGTCGCAAACCTGGTTGGAATCAAGAGAATAC 720
```

```
BEH819966          TCCCATTCCACTAGGAAGCTGCAAACGCGGTCGCAAACCTGGTTGGAATCAAGAGAATAC 720
SSABr              TCCCATTCCACTAGGAAGCTGCAAACGCGGTCGCAAACCTGGTTGGAATCAAGAGAATAC 720
Beh815744          TCCCATTCCACTAGGAAGCTGCAAACGCGGTCGCAAACCTGGTTGGAATCAAGAGAATAC 720
BEH818995          TCCCATTCCACTAGGAAGCTGCAAACGCGGTCGCAAACCTGGTTGGAATCAAGAGAATAC 720
IbH30656           TCTCACTCTACAAGGAAGTTGCAAACGCGATCGCAGACTTGGCTAGAATCAAGAGAATAC 720
ArD128000          TCTCACTCCACAAGGAAGTTGCAAACGCGGTCGCAGACCTGGTTAGAATCAAGAGAATAC 720
ArD7117            TCTCACTCCACAAGGAAGCTGCAAACGCGATCACAGACTTGGCTAGAATCAAGAGAATAC 720
ARD_41519          TCTCACTCCACGAGGAAGCTGCAAACGCGATCGCAGACTTGGCTAGAGTCAAGAGAATAC 720
ARD157995          TATCACTATACAAGGAAGTTGCAAACGCGGTCGCAGACATGGTTAGAATCAAGAGAATAC 720
MR_766             TCTCACTCTACAAGGAAGTTGCAAACGCGGTCGCAGACCTGGTTAGAATCAAGAGAATAC 720
ARD158084          TCTCACTCTACAAGGAAGTTGCAAACGCGGTCGCAGACCTGGTTAGAATCAAGAGAATAC 720
ArB1362            TCTCACTCCACAAGGAAGTTGCAAACGCGGTCGCAGACCTGGTTAGAATCAAGAGAATAC 720
ARB13565           TCTCACTCTACAAGGAAGTTGCAAACGCGGTCGCAGACCTGGTTAGAATCAAGAGAATAC 720
ARB7701            TCTCACTCTACAAGGAAGTTGCAAACGCGGTCGCAGACCTGGTTAGAATCAAGAGAATAC 720
ARB15076           TCTCACTCTACAAGGAAGCTGCAAACGCGGTCGCAGACTTGGTTAGAATCAAGAGAATAC 720
                   *  ** *  ** ****** ********** ** ** ** *** * ** ***********

P6-740             ACAAAGCACTTGATCAGAGTCGAAAATTGGATATTCAGGAACCCTGGCTTTGCGTTGGCA 780
CPC0740            ACAAAGCACCTGATTAGAGTTGAAAATTGGATATTCAGGAACCCTGGCTTCGCGTTAGCA 780
YAP2007            ACAAAGCACCTGATTAGAGTCGAAAATTGGATATTCAGGAACCCTGGCTTCGCGTTAGCA 780
FSS13025           ACAAAGCACCTGATTAGAGTCGAAAATTGGATATTCAGGAACCCTGGCTTCGCGTTAGCA 780
PLCal_ZV           ACAAAGCACTTGATTAGAGTCGAAAATTGGATATTCAGGAACCCTGGCTTCGCGTTAGCA 669
SV0127/14          ACAAAGCACTTGATTAGAGTCGAAAATTGGATATTCAGGAACCCTGGCTTCGCGTTAGCA 780
8375               ACAAAGCACTTGATTAGAGTCGAAAATTGGATATTCAGGAACCCTGGCTTCGCGTTAGCA 780
103344             ACAAAGCACTTGATTAGAGTCGAAAATTGGATATTCAGGAACCCTGGCTTCGCGTTAGCA 780
BrasilZKV2015      ACAAAGCACTTGATTAGAGTCGAAAATTGGATATTCAGGAACCCTGGCTTCGCGTTAGCA 780
GD01               ACAAAGCACTTGATTAGAGTCGAAAATTGGATATTCAGGAACCCTGGCTTCGCGTTAGCA 780
Martinique         ACAAAGCACTTGATTAGAGTCGAAAATTGGATATTCAGGAACCCTGGTTTCGCTTTAGCA 780
NatalRGN           ACAAAGCACTTGATTAGAGTCGAAAATTGGATATTCAGGAACCCTGGCTTCGCGTTAGCA 780
HPF2013            ACAAAGCACTTGATTAGAGTCGAAAATTGGATATTCAGGAACCCTGGCTTCGCGTTAGCA 780
SPH2015            ACAAAGCACTTGATTAGAGTCGAAAATTGGATATTCAGGAACCCTGGCTTCGCGTTAGCA 780
Haiti2014          ACAAAGCACTTGATTAGAGTCGAAAATTGGATATTCAGGAACCCTGGCTTCGCGTTAGCA 780
PRVABC59           ACAAAGCACTTGATTAGAGTCGAAAATTGGATATTCAGGAACCCTGGCTTCGCGTTAGCA 780
BeH819015          ACAAAGCACTTGATTAGAGTCGAAAATTGGATATTCAGGAACCCTGGCTTCGCGTTAGCA 780
Z1106033           ACAAAGCACTTGATTAGAGTCGAAAATTGGATATTCAGGAACCCTGGCTTCGCGTTAGCA 780
BEH819966          ACAAAGCACTTGATTAGAGTCGAAAATTGGATATTCAGGAACCCTGGCTTCGCGTTAGCA 780
SSABr              ACAAAGCACTTGATTAGAGTCGAAAATTGGATATTCAGGAACCCTGGCTTCGCGTTAGCA 780
Beh815744          ACAAAGCACTTGATTAGAGTCGAAAATTGGATATTTAGGAACCCTGGCTTCGCGTTAGCA 780
BEH818995          ACAAAGCACTTGATTAGAGTCGAAAATTGGATATTTAGGAACCCTGGCTTCGCGTTAGCA 780
IbH30656           ACAAAGCACCTGATCAAGGTTGAGAATTGGATATTCAGGAACCCCGGGTTTGCGCTAGTG 780
ArD128000          ACGAAGCACTTGATCAAGGTTGAAAACTGGATATTCAGGAACCCCGGGTTTGCGCTAGCG 780
ArD7117            ACAAAGCACCTGATCAAGGTTGAGAATTGGATATTCAGGAACCCCGGGTTTGCGCTAGTG 780
ARD_41519          ACAAAGCACCTGATCAAGGTTGAAAATTGGATATTTAGGAACCCCGGGTTTGCGCTAGTG 780
ARD157995          AAGAAGCACTTGATCATGGTCGAAAACTGGATATTCAGGAACCCCGGGTTTGCCATAGTG 780
MR_766             ACGAAGCACTTGATCAAGGTTGAAAACTGGATATTCAGGAACCCCGGGTTTGCGCTAGTG 780
ARD158084          ACGAAGCACTTGATCAAGGTTGAAAACTGGATATTCAGGAACCCCGGGTTTGCGCTAGTG 780
ArB1362            ACGAAGCACTTGATCAAGGTTGAAAACTGGATATTCAGGAACCCCGGGTTTGCGCTAGCG 780
ARB13565           ACGAAGCACCTGATCAAGGTTGAAAACTGGATATTTAGGAACCCCGGGTTTGCGCTCGCA 780
ARB7701            ACGAAGCACCTGATCAAGGTTGAAAACTGGATATTTAGGAACCCCGGGTTTGCGCTCGCA 780
ARB15076           ACGAAGCACTTGATCAAGGTTGAAAACTGGATATTCAGGAACCCCGGGTTTGCGCTCGCA 780
                   *  ****** **** *  ** ** ** ******** ******** ** ** **   * *

P6-740             GCAGCTGCCATTGCTTGGCTTTTGGGAAGCTCAACGAGCCAAAAAGTCATATACTTGGTC 840
CPC0740            GCAGCTGTCATCGCTTGGCTTTTGGGAAGTTCAACGAGCCAAAAAGTCATATATCTGGTC 840
YAP2007            GCAGCTGCCATCGCCTGGCTTTTGGGAAGTTCAACGAGCCAAAAAGTCATATACTTGGTC 840
FSS13025           GCAGCTGCCATCGCTTGGCTTTTGGGAAGCTCAACGAGCCAAAAAGTCATATACTTGGTC 840
PLCal_ZV           GCAGCTGCCATCGCTTGGCTCTTGGGAAGCTCAACGAGCCAAAAAGTCATATACTTGGTC 729
SV0127/14          GCAGCTGCCATCGCTTGGCTTTTGGGAAGCTCAACGAGCCAAAAAGTCATATACTTGGTC 840
8375               GCAGCTGCCATCGCTTGGCTTTTGGGAAGCTCAACGAGCCAAAAAGTCATATACTTGGTC 840
103344             GCAGCTGCCATCGCTTGGCTTTTGGGAAGCTCAACGAGCCAAAAAGTCATATACTTGGTC 840
BrasilZKV2015      GCAGCTGCCATCGCTTGGCTTTTGGGAAGCTCAACGAGCCAAAAAGTCATATACTTGGTC 840
GD01               GCAGCTGCTATCGCTTGGCTTTTGGGAAGCTCAACGAGCCAAAAAGTCATATACTTGGTC 840
Martinique         GCAGCTGCCATCGCGTGGCTTTTGGGAAGCTCAACGAGCCAAAAAGTCATATACTTGGTC 840
NatalRGN           GCAGCTGCCATCGCTTGGCTTTTGGGAAGCTCAACGAGCCAAAAAGTCATATACTTGGTC 840
HPF2013            GCAGCTGCCATCGCTTGGCTTTTGGGAAGCTCAACGAGCCAAAAAGTCATATACTTGGTC 840
SPH2015            GCAGCTGCCATCGCTTGGCTTTTGGGAAGCTCAACGAGCCAAAAAGTCATATACTTGGTC 840
Haiti2014          GCAGCTGCCATCGCTTGGCTTTTGGGAAGCTCAACGAGCCAAAAAGTCATATACTTGGTC 840
PRVABC59           GCAGCTGCCATCGCTTGGCTTTTGGGAAGCTCAACGAGCCAAAAAGTCATATACTTGGTC 840
```

```
BeH819015          GCAGCTGCCATCGCTTGGCTTTTGGGAAGCTCAACGAGCCAAAAAGTCATATACTTGGTC 840
Z1106033           GCAGCTGCCATCGCTTGGCTTTTGGGAAGCTCAACGAGCCAAAAAGTCATATACTTGGTC 840
BEH819966          GCAGCTGCCATCGCTTGGCTTTTGGGAAGCTCAACGAGCCAAAAAGTCATATACTTGGTC 840
SSABr              GCAGCTGCCATCGCTTGGCTTTTGGGAAGCTCAACGAGCCAAAAAGTCATATACTTGGTC 840
Beh815744          GCAGCTGCCATCGCTTGGCTTTTGGGAAGCTCAACGAGCCAAAAAGTCATATACTTGGTC 840
BEH818995          GCAGCTGCCATCGCTTGGCTTTTGGGAAGCTCAACGAGCCAAAAAGTCATATACTTGGTC 840
IbH30656           GCTGTAGCTATTGCCTGGCTCCTGGGAAGCTCGACGAGCCAAAAAGTCATATACTTGGTC 840
ArD128000          GCTGTTGCCATTGCTTGGCTTCTGGGAAGCTCGACGAGCCAAAAAGTCATATACTTGGTC 840
ArD7117            GCTGTAGCTATTGCCTGGCTCCTGGGAAGCTCGACGAGCCAAAAAGTCATATACTTGGTA 840
ARD_41519          GCTGTAGCTATAGCCTGGCTCCTGGGAAGCTCGACGAGCCAAAAAGTTATATACTTGGTC 840
ARD157995          TCCGTTGCCATTACCTGGCTGATGGGAAGCTTGACGAGCCAAAAAGTCATATACTTGGTC 840
MR_766             GCCGTTGCCATTGCCTGGCTTTTGGGAAGCTCGACGAGCCAAAAAGTCATATACTTGGTC 840
ARD158084          GCCGTTGCCATTGCCTGGCTTTTGGGAAGCTCGACGAGCCAAAAAGTCATATACTTGGTC 840
ArB1362            GCTGTTGCCATTGCTTGGCTTCTGGGAAGTTCGACGAGCCAAAAAGTCATATACTTGATC 840
ARB13565           GCTGTTGCCATTGCCTGGCTTTTGGGAAGCTCGACGAGCCAAAAAGTCATATACTTGGTT 840
ARB7701            GCTGTTGCCATTGCCTGGCTTTTGGGAAGCTCGACGAGCCAAAAAGTCATATACTTGGTT 840
ARB15076           GCTGTTGCCATTGCCTGGCTTTTGGGAAGCTCGACGAGCCAAAAAGTCATATACTTGGTC 840
                    * *  *  **  * *****  ******* *  ************** *****  ** *

P6-740             ATGATACTGTTGATTGCCCCGGCATACAGTATCAGGTGCATAGGAGTCAGCAATAGGGAT 900
CPC0740            ATGATACTGCTGATTGCCCCGGCATACAGCATCAGGTGCATAGGAGTCAGCAATAGGGAC 900
YAP2007            ATGATACTGCTGATTGCCCCGGCATACAGCATCAGGTGCATAGGAGTCAGCAATAGGGAC 900
FSS13025           ATGATACTGCTGATTGCCCCGGCATACAGCATCAGGTGCATAGGAGTCAGCAATAGGGAC 900
PLCal_ZV           ATGATACTGCTGATTGCTCCGGCATATAGCATCAGGTGCATAGGAGTCAGCAATAGGGAC 789
SV0127/14          ATGATACTGCTGATTGCCCCGGCATACAGCATCAGGTGCATAGGAGTCAGTAATAGGGAC 900
8375               ATGATACTGCTGATTGCCCCGGCATACAGCATCAGGTGCATAGGAGTCAGCAATAGGGAC 900
103344             ATGATACTGCTGATTGCCCCGGCATACAGCATCAGGTGCATAGGAGTCAGCAATAGGGAC 900
BrasilZKV2015      ATGATACTGCTGATTGCCCCGGCATACAGCATCAGGTGCATAGGAGTCAGCAATAGGGAC 900
GD01               ATGATACTGCTGATTGCCCCGGCATACAGCATCAGGTGCATAGGAGTCAGCAATAGGGAC 900
Martinique         ATGATACTGCTGATTGCCCCGGCATACAGCATCAGGTGCATAGGAGTCAGCAATAGGGAC 900
NatalRGN           ATGATACTGCTGATTGCCCCGGCATACAGCATCAGGTGCATAGGAGTCAGCAATAGGGAC 900
HPF2013            ATGATACTGCTGATTGCCCCGGCATACAGCATCAGGTGCATAGGAGTCAGCAATAGGGAC 900
SPH2015            ATGATACTGCTGATTGCCCCGGCATACAGCATCAGGTGCATAGGAGTCAGCAATAGGGAC 900
Haiti2014          ATGATACTGCTGATTGCCCCGGCATACAGCATCAGGTGCATAGGAGTCAGCAATAGGGAC 900
PRVABC59           ATGATACTGCTGATTGCCCCGGCATACAGCATCAGGTGCATAGGAGTCAGCAATAGGGAC 900
BeH819015          ATGATACTGCTGATTGCCCCGGCATACAGCATCAGGTGCATAGGAGTCAGCAATAGGGAC 900
Z1106033           ATGATACTGCTGATTGCCCCGGCATACAGCATCAGGTGCATAGGAGTCAGCAATAGGGAC 900
BEH819966          ATGATACTGCTGATTGCCCCGGCATACAGCATCAGGTGCATAGGAGTCAGCAATAGGGAC 900
SSABr              ATGATACTGCTGATTGCCCCGGCATACAGCATCAGGTGCATAGGAGTCAGCAATAGGGAC 900
Beh815744          ATGATACTGCTGATTGCCCCGGCATACAGCATCAGGTGCATAGGAGTCAGCAATAGGGAC 900
BEH818995          ATGATACTGCTGATTGCCCCGGCATACAGCATCAGGTGCATAGGAGTCAGCAATAGGGAC 900
IbH30656           ATGATATTGTTGATTGCCCCGGCATACAGTATCAGGTGCATAGGAGTTAGCAATAGAGAC 900
ArD128000          ATGATATTGTTGATTGCCCCGGCATACAGCATCAGGTGCATAGGAGTTAGCAATAGAGAT 900
ArD7117            ATGATATTGTTGATTGCCCCGGCATACAGCATCAGGTGCATAGGAGTTAGCAATAGAGAC 900
ARD_41519          ATGATATTGTTGATTGCCCCGGCATACAGCATTAGGTGCATAGGAGTTAGCAATAGAGAC 900
ARD157995          ATGATAGTGTTGATTGTCCCGGCATACAGTATCAGCTGCATTGGAGTCAGCAATAGAGAC 900
MR_766             ATGATACTGCTGATTGCCCCGGCATACAGTATCAGGTGCATTGGAGTCAGCAATAGAGAC 900
ARD158084          ATGATACTGCTGATTGCCCCGGCATACAGTATCAGGTGCATTGGAGTCAGCAATAGAGAC 900
ArB1362            ATGATACTGCTGATTGCCCCGGCATACAGCATCAGGTGCATTGGAGTCAGCAACAGAGAC 900
ARB13565           ATGATACTGCTGATTGCCCCGGCATACAGCATCAGGTGCATTGGAGTCAGCAATAGAGAC 900
ARB7701            ATGATACTGCTGATTGCCCCGGCATACAGCATCAGGTGCATTGGAGTCAGCAATAGAGAC 900
ARB15076           ATGATACTGCTGATTGCCCCGGCATACAGCATCAGGTGCATTGGAGTCAGCAATAGAGAC 900
                   ****** ** ******  ******** ** ** ** ***** ***** ** ** ** **

P6-740             TTTGTGGAAGGTATGTCAGGTGGGACCTGGGTTGATGTTGTCTTGGAACATGGAGGTTGT 960
CPC0740            TTTGTGGAAGGTATGTCAGGTGGGACTTGGGTTGATGTTGTCTTGGAACATGGAGGTTGT 960
YAP2007            TTTGTGGAAGGTATGTCAGGTGGGACTTGGGTTGATGTTGTCTTGGAACATGGAGGTTGT 960
FSS13025           TTTGTGGAAGGTATGTCAGGTGGGACTTGGGTTGATGTTGTCTTGGAACATGGAGGTTGT 960
PLCal_ZV           TTTGTGGAAGGTATGTCAGGTGGGACTTGGGTTGATGTTGTCTTGGAACATGGAGGTTGT 849
SV0127/14          TTTGTGGAAGGTATGTCAGGTGGGACTTGGGTTGATGTTGTCTTGGAACATGGAGGTTGT 960
8375               TTTGTGGAAGGTATGTCAGGTGGGACTTGGGTTGATGTTGTCTTGGAACATGGAGGTTGT 960
103344             TTTGTGGAAGGTATGTCAGGTGGGACTTGGGTTGATGTTGTCTTGGAACATGGAGGTTGT 960
BrasilZKV2015      TTTGTGGAAGGTATGTCAGGTGGGACTTGGGTTGATGTTGTCTTGGAACATGGGGGTTGT 960
GD01               TTTGTGGAAGGTATGTCAGGTGGGACTTGGGTTGATGTTGTCTTGGAACATGGAGGTTGT 960
Martinique         TTTGTGGAAGGTATGTCAGGTGGGACTTGGGTTGATGTTGTCTTGGAACATGGAGGTTGT 960
NatalRGN           TTTGTGGAAGGTATGTCAGGTGGGACTTGGGTTGATGTTGTCTTGGAACATGGAGGTTGT 960
HPF2013            TTTGTGGAAGGTATGTCAGGTGGGACTTGGGTTGATGTTGTCTTGGAACATGGAGGTTGT 960
SPH2015            TTTGTGGAAGGTATGTCAGGTGGGACTTGGGTTGATATTGTCTTGGAACATGGAGGTTGT 960
```

```
Haiti2014          TTTGTGGAAGGTATGTCAGGTGGGACTTGGGTTGATGTTGTCTTGGAACATGGAGGTTGT 960
PRVABC59           TTTGTGGAAGGTATGTCAGGTGGGACTTGGGTTGATGTTGTCTTGGAACATGGAGGTTGT 960
BeH819015          TTTGTGGAAGGTATGTCAGGTGGGACTTGGGTTGATGTTGTCTTGGAACATGGAGGTTGT 960
Z1106033           TTTGTGGAAGGTATGTCAGGTGGGACTTGGGTTGATGTTGTCTTGGAACATGGAGGTTGT 960
BEH819966          TTTGTGGAAGGTATGTCAGGTGGGACCTGGGTTGATGTTGTCTTGGAACATGGAGGTTGT 960
SSABr              TTTGTGGAAGGTATGTCAGGTGGGACCTGGGTTGATGTTGTCTTGGAACATGGAGGTTGT 960
Beh815744          TTTGTGGAAGGTATGTCAGGTGGGACCTGGGTTGATGTTGTCTTGGAACATGGAGGTTGT 960
BEH818995          TTTGTGGAAGGTATGTCAGGTGGGACCTGGGTTGATGTTGTCTTGGAACATGGAGGTTGT 960
IbH30656           TTCGTGGAGGGCATGTCAGGTGGGACCTGGGTTGATGTTGTCTTGGAACATGGAGGTTGT 960
ArD128000          TTCGTAGAGGGCATGTCAGGTGGGACCTGGGTTGATGTCGTCTTGGAACATGGGGGTTGC 960
ArD7117            TTCGTGGAGGGCATGTCAGGTGGGACCTGGGTTGATGTTGTCTTGGAACATGGGGGTTGC 960
ARD_41519          TTCGTGGAGGGCATGTCAGGTGGGACCTGGGTTGATGTTGTCTTGGAACATGGGGGTTGC 960
ARD157995          TTAGTGGAGGGCATGTCAGGTGGGACCTGGGTTGATGTTGTCTTGGAACATGGAGGGTGC 960
MR_766             TTCGTGGAGGGCATGTCAGGTGGGACCTGGGTTGATGTTGTCTTGGAACATGGAGGCTGC 960
ARD158084          TTCGTGGAGGGCATGTCAGGTGGGACCTGGGTTGATGTTGTCTTGGAACATGGAGGCTGC 960
ArB1362            TTCGTGGAAGGCATGTCAGGTGGGACCTGGGTTGATGTCGTCTTGGAACATGGAGGTTGC 960
ARB13565           TTCGTGGAAGGCATGTCAGGTGGGACCTGGGTTGATGTCGTCTTGGAACATGGAGGTTGC 960
ARB7701            TTCGTGGAAGGCATGTCAGGTGGGACCTGGGTTGATGTCGTCTTGGAACATGGAGGTTGC 960
ARB15076           TTCGTGGAAGGCATGTCAGGTGGGACCTGGGTTGATGTCGTCTTGGAACATGGAGGTTGC 960
                   ** ** ** ** ************** ********* * ************** ** **

P6-740             GTTACCGTAATGGCACAGGACAAGCCAACTGTTGATATAGAGTTGGTCACAACAACGGTT 1020
CPC0740            GTTACCGTAATGGCACAGGACAAACCGACTGTCGACATAGAGCTGGTTACAACAACAGTC 1020
YAP2007            GTTACCGTAATGGCACAGGACAAACCGGCTGTCGACATAGAGCTGGTTACAACAACAGTC 1020
FSS13025           GTTACCGTAATGGCACAGGACAAACCGACTGTCGACATAGAGCTGGTTACAACAACAGTC 1020
PLCal_ZV           GTCACCGTAATGGCACAGGACAAACCGACTGTCGACATAGAGCTGGTTACAACAACAGTC 909
SV0127/14          GTCACCGTAATGGCACAGGACAAACCGACTGTCGACATAGAGCTGGTTACAACAACAGTC 1020
8375               GTCACCGTAATGGCACAGGACAAACCGACTGTCGACATAGAGCTGGTTACAACAACAGTC 1020
103344             GTCACCGTAATGGCACAGGACAAACCGACTGTCGACATAGAGCTGGTTACAACAACAGTC 1020
BrasilZKV2015      GTCACCGTAATGGCACAGGACAAACCGACTGTCGACATAGAGCTGGTTACAACAACAGTC 1020
GD01               GTCACCGTAATGGCACAGGACAAACCGACTGTCGACATAGAGCTGGTTACAACAACAGTC 1020
Martinique         GTCACCGTAATGGCACAGGACAAACCGACTGTCGACATAGAGCTGGTTACAACAACAGTC 1020
NatalRGN           GTCACCGTAATGGCACAGGACAAACCGACTGTCGACATAGAGCTGGTTACAACAACAGTC 1020
HPF2013            GTCACCGTAATGGCACAGGACAAACCGACTGTCGACATAGAGCTGGTTACAACAACAGTC 1020
SPH2015            GTCACCGTAATGGCACAGGACAAACCGACTGTCGACATAGAGCTGGTTACAACAACAGTC 1020
Haiti2014          GTCACCGTAATGGCACAGGACAAACCGACTGTCGACATAGAGCTGGTTACAACAACAGTC 1020
PRVABC59           GTCACCGTAATGGCACAGGACAAACCGACTGTCGACATAGAGCTGGTTACAACAACAGTC 1020
BeH819015          GTCACCGTAATGGCACAGGACAAACCGACTGTCGACATAGAGCTGGTTACAACAACAGTC 1020
Z1106033           GTCACTGTAATGGCACAGGACAAACCGACTGTCGACATAGAGCTGGTTACAACAACAGTC 1020
BEH819966          GTCACCGTAATGGCACAGGACAAACCGACTGTCGACATAGAGCTGGTTACAACAACAGTC 1020
SSABr              GTCACCGTAATGGCACAGGACAAACCGACTGTCGACATAGAGCTGGTTACAACAACAGTC 1020
Beh815744          GTCACCGTAATGGCACAGGACAAACCGACTGTCGACATAGAGCTGGTTACAACAACAGTC 1020
BEH818995          GTCACCGTAATGGCACAGGACAAACCGACTGTCGACATAGAGCTGGTTACAACAACAGTC 1020
IbH30656           GTCACCGTGATGGCACAGGACAAGCCAACAGTTGACATAGAGTTGGTCACGACAACGGTT 1020
ArD128000          GTCACCGTGATGGCACAGGACAAGCCAACAGTTGACATCGAGTTGGTCACGACAACGGTT 1020
ArD7117            GTCACCGTGATGGCACAGGACAAGCCAACAGTTGACATCGAGTTGGTCACGACAACGGTT 1020
ARD_41519          GTCACCGTGATGGCACAGGACAAGCCAACAGTTGATATCGAGTTGGTCACGACAACGGTT 1020
ARD157995          GTTACCGAGATGGCACAGGACAAGCCAACAGTTGACATAGAGTTGGTCACGATGACGGTT 1020
MR_766             GTTACCGTGATGGCACAGGACAAGCCAACAGTCGACATAGAGTTGGTCACGACGACGGTT 1020
ARD158084          GTTACCGTGATGGCACAGGACAAGCCAACAGTTGACATAGAGTTGGTCACGACGACGGTT 1020
ArB1362            GTCACCGTAATGGCACAGGACAAGCCAACAGTTGACATAGAGTTGGTCACGACAACGGTT 1020
ARB13565           GTCACCGTTATGGCACAGGACAAGCCAACAGTTGACATAGAGTTGGTCACGACAACGGTT 1020
ARB7701            GTCACCGTTATGGCACAGGACAAGCCAACAGTTGACATAGAGTTGGTCACGACAACGGTT 1020
ARB15076           GTCACCGTAATGGCACAGGACAAGCCAACAGTTGACATAGAGTTGGTCACGACAACGGTC 1020
                   ** ** *  ************** **  * ** ** ** *** **** ** *  ** **

P6-740             AGCAACATGGCGGAGGTAAGATCCTACTGCTACGAGGCATCAATATCGGACATGGCTTCG 1080
CPC0740            AGCAACATGGCGGAGGTAAGATCCTACTGCTATGAGGCATCAATATCGGATATGGCTTCG 1080
YAP2007            AGCAACATGGCGGAGGTAAGATCCTATTGCTATGAGGCATCAATATCGGACATGGCTTCG 1080
FSS13025           AGCAACATGGCGGAGGTAAGATCCTACTGCTATGAGGCATCAATATCGGACATGGCTTCG 1080
PLCal_ZV           AGCAACATGGCGGAGGTAAGATCCTACTGCTATGAGGCATCAATATCGGACATGGCTTCG 969
SV0127/14          AGCAACATGGCGGAGGTAAGATCCTACTGCTATGAGGCATCAATATCGGACATGGCTTCG 1080
8375               AGCAACATGGCGGAGATAAGATCCTACTGCTATGAGGCATCAATATCAGACATGGCTTCG 1080
103344             AGCAACATGGCGGAGATAAGATCCTACTGCTATGAGGCATCAATATCAGACATGGCTTCG 1080
BrasilZKV2015      AGCAACATGGCGGAGGTAAGATCCTACTGCTATGAGGCATCAATATCAGACATGGCTTCG 1080
GD01               AGCAACATGGCGGAGGTAAGATCCTACTGCTATGAGGCATCAATATCAGACATGGCTTCG 1080
Martinique         AGCAACATGGCGGAGGTAAGATCCTACTGCTATGAGGCATCAATATCAGACATGGCTTCG 1080
NatalRGN           AGCAACATGGCGGAGGTAAGATCCTACTGCTATGAGGCATCAATATCAGACATGGCTTCG 1080
```

```
HPF2013          AGCAACATGGCGGAGGTAAGATCCTACTGCTATGAGGCATCAATATCGGACATGGCTTCG 1080
SPH2015          AGCAACATGGCGGAGGTAAGATCCTACTGCTATGAGGCATCAATATCAGACATGGCTTCG 1080
Haiti2014        AGCAACATGGCGGAGGTAAGATCCTACTGCTATGAGGCATCAATATCAGACATGGCTTCG 1080
PRVABC59         AGCAACATGGCGGAGGTAAGATCCTACTGCTATGAGGCATCAATATCAGACATGGCTTCT 1080
BeH819015        AGCAACATGGCGGAGGTAAGATCCTACTGCTATGAGGCATCAATATCAGACATGGCTTCG 1080
Z1106033         AGCAACATGGCGGAGGTAAGATCCTACTGCTATGAGGCATCAATATCAGACATGGCTTCG 1080
BEH819966        AGCAACATGGCGGAGGTAAGATCCTACTGCTATGAGGCATCAATATCAGACATGGCTTCG 1080
SSABr            AGCAACATGGCGGAGGTAAGATCCTACTGCTATGAGGCATCAATATCAGACATGGCTTCG 1080
Beh815744        AGCAACATGGCGGAGGTAAGATCCTACTGCTATGAGGCATCAATATCAGACATGGCTTCG 1080
BEH818995        AGCAACATGGCGGAGGTAAGATCCTACTGCTATGAGGCATCAATATCAGACATGGCTTCG 1080
IbH30656         AGCAACATGGCCGAGGTGAGATCCTACTGCTACGAGGCATCAATATCGGACATGGCTTCG 1080
ArD128000        AGCAACATGGCCGAGGTAAGATCCTACTGCTATGAGGCATCAATATCGGACATGGCCTCG 1080
ArD7117          AGCAACATGGCCGAGGTAAGATCCTACTGCTATGAGGCATCAATATCGGACATGGCTTCG 1080
ARD_41519        AGCAACATGGCCGAGGTAAGATCCTACTGCTATGAGGCATCAATATCGGACATGGCTTCG 1080
ARD157995        AGTAACATGGCCGAGGTAAGATCCTATTGCTACGAGGCATCGTTATCCGACATGGCTTCG 1080
MR_766           AGTAACATGGCCGAGGTAAGATCCTATTGCTACGAGGCATCGATATCGGACATGGCTTCG 1080
ARD158084        AGTAACATGGCCGAGGTAAGATCCTATTGCTACGAGGCATCGATATCGGACATGGCTTCG 1080
ArB1362          AGCAACATGGCTGAGGTAAGATCCTACTGCTACGAGGCATCAATATCGGACATGGCTTCG 1080
ARB13565         AGTAACATGGCCGAGGTAAGATCCTACTGTTATGAGGCATCAATATCAGACATGGCTTCG 1080
ARB7701          AGTAACATGGCCGAGGTAAGATCCTACTGTTATGAGGCATCAATATCAGACATGGCTTCG 1080
ARB15076         AGTAACATGGCCGAGGTAAGATCCTACTGTTATGAGGCATCAATATCGGACATGGCTTCG 1080
                 ** ******** *** * ******** ** ** ********  **** ** ***** **

P6-740           GACAGCCGCTGCCCAACACAAGGTGAAGCCTACCTTGACAAGCAGTCAGACACTCAATAT 1140
CPC0740          GACAGCCGCTGCCCAACACAAGGTGAGGCCTACCTTGACAAGCAGTCAGACACTCAATAT 1140
YAP2007          GACAGCCGCTGCCCAACACAAGGTGAAGCCTACCTTGACAAGCAGTCAGACACTCAATAT 1140
FSS13025         GACAGCCGCTGCCCAACACAAGGTGAAGCCTACCTTGACAAGCAATCAGACACTCAATAT 1140
PLCal_ZV         GACAGCCGCTGCCCAACACAAGGCGAAGCCTACCTTGACAAGCAATCAGACACTCAATAT 1029
SV0127/14        GACAGCCGCTGCCCAACACAAGGTGAAGCCTACCTTGACAAGCAATCAGACACTCAATAT 1140
8375             GACAGCCGCTGCCCAACACAAGGTGAAGCCTACCTTGACAAGCAATCAGACACTCAATAT 1140
103344           GACAGCCGCTGCCCAACACAAGGTGAAGCCTACCTTGACAAGCAATCAGACACTCAATAT 1140
BrasilZKV2015    GACAGCCGCTGCCCAACACAAGGTGAAGCCTACCTTGACAAGCAATCAGACACTCAATAT 1140
GD01             GACAGCCGCTGCCCAACACAAGGTGAAGCCTACCTTGACAAGCAATCAGACACTCAATAT 1140
Martinique       GACAGCCGCTGCCCAACACAAGGTGAAGCCTACCTTGACAAGCAATCAGACACTCAATAT 1140
NatalRGN         GACAGCCGCTGCCCAACACAAGGTGAAGCCTACCTTGACAAGCAATCAGACACTCAATAT 1140
HPF2013          GACAGCCGCTGCCCAACACAAGGTGAAGCCTACCTTGACAAGCAATCAGACACTCAATAT 1140
SPH2015          GACAGCCGCTGCCCAACACAAGGTGAAGCCTACCTTGACAAGCAATCAGACACTCAATAT 1140
Haiti2014        GACAGCCGCTGCCCAACACAAGGTGAAGCCTACCTTGACAAGCAATCAGACACTCAATAT 1140
PRVABC59         GACAGCCGCTGCCCAACACAAGGTGAAGCCTACCTTGACAAGCAATCAGACACTCAATAT 1140
BeH819015        GACAGCCGCTGCCCAACACAAGGTGAAGCCTACCTTGACAAGCAATCAGACACTCAATAT 1140
Z1106033         GACAGCCGCTGCCCAACACAAGGTGAAGCCTACCTTGACAAGCAATCAGACACTCAATAT 1140
BEH819966        GACAGCCGCTGCCCAACACAAGGTGAAGCCTACCTTGACAAGCAATCAGACACTCAATAT 1140
SSABr            GACAGCCGCTGCCCAACACAAGGTGAAGCCTACCTTGACAAGCAATCAGACACTCAATAT 1140
Beh815744        GACAGCCGCTGCCCAACACAAGGTGAAGCCTACCTTGACAAGCAATCAGACACTCAATAT 1140
BEH818995        GACAGCCGCTGCCCAACACAAGGTGAAGCCTACCTTGACAAGCAATCAGACACTCAATAT 1140
IbH30656         GACAGTCGCTGCCCAACACAAGGTGAAGCCTACCTTGACAAGCAGTCAGACACTCAATAT 1140
ArD128000        GACAGCCGCTGTCCAACACAAGGTGAAGCCTACCTTGACAAGCAATCAGACACTCAATAT 1140
ArD7117          GACAGCCGCTGTCCAACACAAGGTGAAGCCTACCTTGACAAGCAGTCAGACACTCAATAT 1140
ARD_41519        GACAGCCGTTGTCCAACACAAGGTGAAGCCTACCTTGACAAGCAGTCAGACACTCAATAT 1140
ARD157995        GCCAGTCGTTGCCCAACACAAGGCGAACCCTCCCTCGACAAGCAATCAGACACTCAATCT 1140
MR_766           GACAGTCGTTGCCCAACACAAGGTGAAGCCTACCTTGACAAGCAATCAGACACTCAATAT 1140
ARD158084        GACAGTCGTTGCCCAACACAAGGTGAAGCCTACCTTGACAAGCAATCAGACACTCAATAT 1140
ArB1362          GATAGTCGCTGCCCAACACAAGGTGAAGCCTACCTTGACAAGCAATCAGACACTCAATAT 1140
ARB13565         GACAGTCGCTGCCCAACACAAGGTGAAGCCTACCTTGATAAGCAATCAGACACCCAATAT 1140
ARB7701          GACAGTCGCTGCCCAACACAAGGTGAAGCCTACCTTGATAAGCAATCAGACACCCAATAT 1140
ARB15076         GACAGTCGCTGCCCAACACAAGGTGAAGCCTACCTTGACAAGCAATCAGACACTCAATAT 1140
                 *  ** ** ** *********** **  *** *** ** ***** ******** **** *

P6-740           GTTTGCAAAAGAACGTTAGTGGACAGAGGTTGGGGAAATGGATGTGGACTCTTTGGCAAA 1200
CPC0740          GTCTGCAAAAGAACGTTAGTGGACAGAGGCTGGGGAAATGGATGTGGACTTTTTGGCAAA 1200
YAP2007          GTCTGCAAAAGAACGTTAGTGGACAGAGGCTGGGGAAATGGATGTGGACTTTTTGGCAAA 1200
FSS13025         GTCTGCAAAAGAACGTTAGTGGACAGAGGCTGGGGAAATGGATGTGGACTTTTTGGCAAA 1200
PLCal_ZV         GTCTGCAAAAGAACGTTAGTGGACAGAGGCTGGGGAAATGGATGTGGACTTTTTGGCAAA 1089
SV0127/14        GTCTGCAAAAGAACGTTAGTGGACAGAGGCTGGGGAAATGGATGTGGACTTTTTGGCAAA 1200
8375             GTCTGCAAAAGAACGTTAGTGGACAGAGGCTGGGGAAATGGATGTGGACTTTTTGGCAAA 1200
103344           GTCTGCAAAAGAACGTTAGTGGACAGAGGCTGGGGAAATGGATGTGGACTTTTTGGCAAA 1200
BrasilZKV2015    GTCTGCAAAAGAACGTTAGTGGACAGAGGCTGGGGAAATGGATGTGGACTTTTTGGCAAA 1200
GD01             GTCTGCAAAAGAACGTTAGTGGACAGAGGCTGGGGAAATGGATGTGGACTTTTTGGCAAA 1200
```

```
Martinique          GTCTGCAAAAGAACGTTAGTGGACAGAGGCTGGGGAAATGGATGTGGACTTTTTGGCAAA 1200
NatalRGN            GTCTGCAAAAGAACGTTAGTGGACAGAGGCTGGGGAAATGGATGTGGACTTTTTGGCAAA 1200
HPF2013             GTCTGCAAAAGAACGTTAGTGGACAGAGGCTGGGGAAATGGATGTGGACTTTTTGGCAAA 1200
SPH2015             GTCTGCAAAAGAACGTTAGTGGACAGAGGCTGGGGAAATGGATGTGGACTTTTTGGCAAA 1200
Haiti2014           GTCTGCAAAAGAACGTTAGTGGACAGAGGCTGGGGAAATGGATGTGGACTTTTTGGCAAA 1200
PRVABC59            GTCTGCAAAAGAACGTTAGTGGACAGAGGCTGGGGAAATGGATGTGGACTTTTTGGCAAA 1200
BeH819015           GTCTGCAAAAGAACGTTAGTGGACAGAGGCTGGGGAAATGGATGTGGACTTTTTGGCAAA 1200
Z1106033            GTCTGCAAAAGAACGTTAGTGGACAGAGGCTGGGGAAATGGATGTGGACTTTTTGGCAAA 1200
BEH819966           GTCTGCAAAAGAACGTTAGTGGACAGAGGCTGGGGAAATGGATGTGGACTTTTTGGCAAA 1200
SSABr               GTCTGCAAAAGAACGTTAGTGGACAGAGGCTGGGGAAATGGATGTGGACTTTTTGGCAAA 1200
Beh815744           GTCTGCAAAAGAACGTTAGTGGACAGAGGCTGGGGAAATGGATGTGGACTTTTTGGCAAA 1200
BEH818995           GTCTGCAAAAGAACGTTAGTGGACAGAGGCTGGGGAAATGGATGTGGACTTTTTGGCAAA 1200
IbH30656            GTCTGTAAAAGAACATTGGTGGACAGAGGTTGGGGAAATGGGTGTGGACTTTTTGGCAAG 1200
ArD128000           GTCTGCAAAAGAACATTAGTGGACAGAGGTTGGGGAAACGGTTGTGGACTTTTTGGCAAA 1200
ArD7117             GTCTGCAAGAGAACATTGGTGGATAGAGGTTGGGGAAATGGGTGTGGACTTTTTGGCAAA 1200
ARD_41519           GTCTGCAAGAGAACATTGGTGGATAGAGGTTGGGGAAATGGGTGTGGACTTTTTGGCAAA 1200
ARD157995           GTATGCAAAAGAACATTAGGAGACAGAGGTTGGGGAAATGGTTGTGGGATTTTTGGCAAA 1200
MR_766              GTCTGCAAAAGAACATTAGTGGACAGAGGTTGGGGAAACGGTTGTGGACTTTTTGGCAAA 1200
ARD158084           GTCTGCAAAAGAACATTAGTGGACAGAGGTTGGGGAAATGGTTGTGGACTTTTTGGCAAA 1200
ArB1362             GTTTGCAAAAGAACATTGGTGGACAGAGGTTGGGGAAATGGGTGTGGACTTTTTGGCAAA 1200
ARB13565            GTGTGCAAAAGAACATTGGTGGACAGAGGTTGGGGAAATGGGTGTGGACTTTTTGGCAAA 1200
ARB7701             GTGTGCAAAAGAACATTGGTGGACAGAGGTTGGGGAAATGGGTGTGGACTTTTTGGCAAA 1200
ARB15076            GTATGCAAAAGAACATTGGTGGACAGAGGTTGGGGAAATGGGTGTGGACTTTTTGGCAAA 1200
                    ** ** ** ***** ** *  ** ***** ******** ** *****  * ********

P6-740              GGGAGCCTGGTGACATGCGCCAAGTTTGCATGCTCCAAGAAAATGACTGGGAAGAGCATC 1260
CPC0740             GGGAGCCTGGTGACATGCGCTAAGTTTGCATGCTCCAAGAAAATGACCGGGAAGAGCATC 1260
YAP2007             GGGAGCCTGGTGACATGCGCTAAGTTTGCATGCTCCAAGAAAATGACCGGGAAGAGCATC 1260
FSS13025            GGGAGCCTGGTGACATGCGCTAAGTTTGCTTGCTCTAAGAAAATGACCGGGAAGAGCATC 1260
PLCal_ZV            GGGAGCCTGGTGACATGCGCCAAGTTTGCATGCTCCAAGAAAATGACCGGGAAGAGCATC 1149
SV0127/14           GGGAGCCTGGTGACATGCGCTAAGTTTGCATGCTCCAAGAAAATGACCGGGAAGAGCATC 1260
8375                GGGAGCCTGGTGACATGCGCTAAGTTTGCATGCTCCAAGAAAATGACCGGGAAGAGCATC 1260
103344              GGGAGCCTGGTGACATGCGCTAAGTTTGCATGCTCCAAGAAAATGACCGGGAAGAGCATC 1260
BrasilZKV2015       GGGAGCCTGGTGACATGCGCTAAGTTTGCATGCTCCAAGAAAATGACCGGGAAGAGCATC 1260
GD01                GGGAGCCTGGTGACATGCGCTAAGTTTGCATGCTCCAAGAAAATGACCGGGAAAAGCATC 1260
Martinique          GGGAGCCTGGTGACATGCGCTAAGTTTGCATGCTCCAAGAAAATGACCGGGAAGAGCATC 1260
NatalRGN            GGGAGCCTGGTGACATGCGCTAAGTTTGCATGCTCCAAGAAAATGACCGGGAAGAGCATC 1260
HPF2013             GGGAGCCTGGTGACATGCGCTAAGTTTGCATGCTCCAAGAAAATGACCGGGAAGAGCATC 1260
SPH2015             GGGAGTCTGGTGACATGCGCTAAGTTTGCATGCTCCAAGAAAATGACCGGGAAGAGCATC 1260
Haiti2014           GGGAGTCTGGTGACATGCGCTAAGTTTGCATGCTCCAAGAAAATGACCGGGAAGAGCATC 1260
PRVABC59            GGGAGCCTGGTGACATGCGCTAAGTTTGCATGCTCCAAGAAAATGACCGGGAAGAGCATC 1260
BeH819015           GGGAGCCTGGTGACATGCGCTAAGTTTGCATGCTCCAAGAAAATGACCGGGAAGAGCATC 1260
Z1106033            GGGAGCCTGGTGACATGCGCTAAGTTTGCATGCTCCAAGAAAATGACCGGGAAGAGCATC 1260
BEH819966           GGGAGCCTGGTGACATGCGCTAAGTTTGCATGCTCCAAGAAAATGACCGGGAAGAGCATC 1260
SSABr               GGGAGCCTGGTGACATGCGCTAAGTTTGCATGCTCCAAGAAAATGACCGGGAAGAGCATC 1260
Beh815744           GGGAGCCTGGTGACATGCGCTAAGTTTGCATGCTCCAAGAAAATGACCGGGAAGAGCATC 1260
BEH818995           GGGAGCCTGGTGACATGCGCTAAGTTTGCATGCTCCAAGAAAATGACCGGGAAGAGCATC 1260
IbH30656            GGGAGCTTGGTGACGTGTGCCAAGTTTACATGCTCCAAGAAAATGACAGGGAAGAGCATC 1260
ArD128000           GGGAGCTTGGTGACATGTGCCAAGTTTACGTGTTCTAAGAAGATGACCGGGAAGAGCATT 1260
ArD7117             GGGAGCTTGGTGACATGTGCCAAGTTTACGTGCTCCAAGAAAATGACAGGCAAGAGCATC 1260
ARD_41519           GGGAGCTTGGTGACATGTGCCAAGTTTACGTGCTCCAAGAAAATGACAGGCAAGAGCATC 1260
ARD157995           GGGAGCTTGGTGACATGTTCCAAGTTCACGTGTTGTAAGAAGATGCCCGGGAAGAGCATT 1260
MR_766              GGGAGCTTGGTGACATGTGCCAAGTTTACGTGTTCTAAGAAGATGACCGGGAAGAGCATT 1260
ARD158084           GGGAGCTTGGTGACATGTGCCAAGTTCACGTGTTCTAAGAAGATGACCGGGAAGAGCATT 1260
ArB1362             GGGAGCCTGGTGACATGTGCCAAGTTCACGTGTTCCAAGAAGATGACCGGGAAGAGCATC 1260
ARB13565            GGGAGCCTGGTGACATGTGCCAAGTTCACGTGTTCCAAGAAGATGACTGGGAAGAGCATT 1260
ARB7701             GGGAGCCTGGTGACATGTGCCAAGTTCACGTGTTCCAAGAAGATGACTGGGAAGAGCATT 1260
ARB15076            GGGAGCCTGGTGACATGTGCCAAGTTCACGTGTTCCAAGAAGATGACCGGGAAGAGCATT 1260
                    *****  ******* **  * *****  * ** *  ***** *** * ** ** *****

P6-740              CAGCCAGAGAACCTGGAGTACCGGATAATGCTGTCAGTTCATGGCTCCCAGCACAGTGGG 1320
CPC0740             CAGCCAGAGAATCTGGAGTACCGGATAATGCTGTCAGTTCATGGCTCCCAGCACAGTGGG 1320
YAP2007             CAGCCAGAGAATCTGGAGTACCGGATAATGCTGTCAGTTCATGGCTCCCAGCACAGTGGG 1320
FSS13025            CAGCCAGAGAATCTGGAGTACCGGATAATGCTGTCAGTTCATGGCTCCCAGCACAGTGGG 1320
PLCal_ZV            CAGCCAGAGAATCTGGAGTACCGAATAATGCTGTCAGTTCATGGCTCCCAGCACAGTGGG 1209
SV0127/14           CAGCCAGAGAATCTGGAGTACCGGATAATGCTGTCAGTTCATGGCTCCCAGCACAGTGGG 1320
8375                CAGCCAGAGAATCTGGAGTACCGGATAATGCTGTCAGTTCATGGCTCCCAGCACAGTGGG 1320
103344              CAGCCAGAGAATCTGGAGTACCGGATAATGCTGTCAGTTCATGGCTCCCAGCACAGTGGG 1320
```

```
BrasilZKV2015      CAGCCAGAGAATCTGGAGTACCGGATAATGCTGTCAGTTCATGGCTCCCAGCACAGTGGG 1320
GD01               CAGCCAGAGAATCTGGAGTACCGGATAATGCTGTCAGTTCATGGCTCCCAGCACAGTGGG 1320
Martinique         CAGCCAGAGAATCTGGAGTACCGGATAATGTTGTCAGTTCATGGCTCCCAGCACAGTGGG 1320
NatalRGN           CAGCCAGAGAATCTGGAGTACCGGATAATGCTGTCAGTTCATGGCTCCCAGCACAGTGGG 1320
HPF2013            CAGCCAGAGAATCTGGAGTACCGGATAATGCTGTCAGTTCATGGCTCCCAGCACAGTGGG 1320
SPH2015            CAGCCAGAGAATCTGGAGTACCGGATAATGCTGTCAGTTCATGGCTCCCAGCACAGTGGG 1320
Haiti2014          CAGCCAGAGAATCTGGAGTACCGGATAATGCTGTCAGTTCATGGCTCCCAGCACAGTGGG 1320
PRVABC59           CAGCCAGAGAATCTGGAGTACCGGATAATGCTGTCAGTTCATGGCTCCCAGCACAGTGGG 1320
BeH819015          CAGCCAGAGAATCTGGAGTACCGGATAATGCTGTCAGTTCATGGCTCCCAGCACAGTGGG 1320
Z1106033           CAGCCAGAGAATCTGGAGTACCGGATAATGCTGTCAGTTCATGGCTCCCAGCACAGTGGG 1320
BEH819966          CAGCCAGAGAATCTGGAGTACCGGATAATGCTGTCAGTTCATGGCTCCCAGCACAGTGGG 1320
SSABr              CAGCCAGAGAATCTGGAGTACCGGATAATGCTGTCAGTTCATGGCTCCCAGCACAGTGGG 1320
Beh815744          CAGCCAGAGAATCTGGAGTACCGGATAATGCTGTCAGTTCATGGCTCCCAGCACAGTGGG 1320
BEH818995          CAGCCAGAGAATCTGGAGTACCGGATAATGCTGTCAGTTCATGGCTCCCAGCACAGTGGG 1320
IbH30656           CAGCCGGAGAACTTGGAGTACCGGATAATGCTATCAGTGCATGGATCCCAGCACAGTGGG 1320
ArD128000          CAGCCGGAGAACTTGGAGTACCGGATAATGCTATCAGTGCATGGATCCCAGCACAGTGGG 1320
ArD7117            CAGCCGGAGAACTTGGAGTACCGGATAATGCTATCAGTGCATGGATCCCAGCACAGTGGG 1320
ARD_41519          CAGCCGGAGAACTTGGAGTACCGGATAATGCTATCAGTGCATGGATCCCAGCACAGTGGG 1320
ARD157995          CAACCGGAAAATCTGGAGTATCGGATAATGCTCCCAGTGCATGGCTCCCAGCATAGCGGG 1320
MR_766             CAACCGGAAAATCTGGAGTATCGGATAATGCTATCAGTGCATGGCTCCCAGCATAGCGGG 1320
ARD158084          CAACCGGAAAATCTGGAGTATCGGATAATGCTATCAGTGCATGGCTCCCAGCATAGCGGG 1320
ArB1362            CAGCCGGAGAATCTGGAGTATCGAATAATGCTATCGGTGCATGGCTCCCAGCACAGCGGG 1320
ARB13565           CAGCCGGAGAATCTGGAGTATCGAATAATGCTATCAGTGCATGGCTCCCAGCACAGCGGG 1320
ARB7701            CAGCCGGAGAATCTGGAGTATCGAATAATGCTATCAGTGCATGGCTCCCAGCACAGCGGG 1320
ARB15076           CAGCCGGAGAATCTGGAGTATCGAATAATGCTATCAGTGCATGGCTCCCAGCACAGCGGG 1320
                   ** ** ** **  ******* ** ****** *  * ** ***** ******** ** ***

P6-740             ATGATTGTTAATGACAcAGGACATGAAACTGATGAGAATAGAGCGAAGGTTGAGATAACG 1380
CPC0740            ATGATCGTTAATGACACAGGACATGAAACTGATGAGAATAGAGCGAAGGTTGAGATAACG 1380
YAP2007            ATGATCGTTAATGACACAGGACATGAAACTGATGAGAATAGAGCGAAGGTTGAGATAACG 1380
FSS13025           ATGATCGTTAATGATACAGGACATGAAACTGATGAGAATAGAGCGAAGGTTGAGATAACG 1380
PLCal_ZV           ATGATCGTTAATGACACAGGACATGAAACTGATGAGAATAGAGCGAAGGTTGAGATAACG 1269
SV0127/14          ATGATCGTTAATGACACAGGACATGAAACTGATGAGAATAGAGCGAAGGTTGAGATAACG 1380
8375               ATGATCGTTAATGACACAGGACATGAAACTGATGAGAATAGAGCGAAGGTTGAGATAACG 1380
103344             ATGATCGTTAATGACACAGGACATGAAACTGATGAGAATAGAGCGAAGGTTGAGATAACG 1380
BrasilZKV2015      ATGATCGTTAATGACACAGGACATGAAACTGATGAGAATAGAGCGAAGGTTGAGATAACG 1380
GD01               ATGATTGTTAATGGCACAGGACATGAAACTGATGAGAATAGAGCGAAAGTTGAGATAACG 1380
Martinique         ATGATCGTTAATGACACAGGACATGAAACTGATGAGAATAGAGCGAAGGTTGAGATAACG 1380
NatalRGN           ATGATCGTTAATGACACAGGACATGAAACTGATGAGAATAGAGCGAAGGTTGAGATAACG 1380
HPF2013            ATGATCGTTAATGACACAGGACATGAAACTGATGAGAATAGAGCGAAGGTTGAGATAACG 1380
SPH2015            ATGATCGTTAATGACACAGGACATGAAACTGATGAGAATAGAGCGAAGGTTGAGATAACG 1380
Haiti2014          ATGATCGTTAATGACACAGGACATGAAACTGATGAGAATAGAGCGAAGGTTGAGATAACG 1380
PRVABC59           ATGATCGTTAATGACACAGGACATGAAACTGATGAGAATAGAGCGAAAGTTGAGATAACG 1380
BeH819015          ATGATCGTTAATGACACAGGACATGAAACTGATGAGAATAGAGCGAAAGTTGAGATAACG 1380
Z1106033           ATGATCGTTAATGACACAGGACATGAAACTGATGAGAATAGAGCGAAAGTTGAGATAACG 1380
BEH819966          ATGATTGTTAATGACACAGGACATGAAACTGATGAGAATAGAGCGAAAGTTGAGATAACG 1380
SSABr              ATGATTGTTAATGACACAGGACATGAAACTGATGAGAATAGAGCGAAAGTTGAGATAACG 1380
Beh815744          ATGATTGTTAATGACACAGGACATGAAACTGATGAGAATAGAGCGAAAGTTGAGATAACG 1380
BEH818995          ATGATTGTTAATGACACAGGACATGAAACTGATGAGAATAGAGCGAAAGTTGAGATAACG 1380
IbH30656           ATGATTGTGAATGA------------------CGAAAACAGAGCAAAAGTCGAGGTTACA 1362
ArD128000          ATGattgttaatgacataGGACATGAAACTGACGAAAACAGAGCAAAGGTCGAGGTCACA 1380
ArD7117            ATGATTGTGAATGACATAGGACATGAAACTGACGAAAACAGAGCAAAAGTCGAGGTCACA 1380
ARD_41519          ATGATTGTGAATGACACAGGACATGAAACTGACGAAAACAGAGCAAAAGTCGAGGTCACA 1380
ARD157995          ATGATTGTGAATGACATAGGACATGAAACTGACGAAAACAGAGCGAAAGTCGAGGTCACA 1380
MR_766             ATGATTGGAT------------ATGAAACTGACGAAGATAGAGCGAAAGTCGAGGTTACG 1368
ARD158084          ATGATTGTCAATGATATAGGACATGAAACTGACGAAAACAGAGCGAAAGTCGAGGTTACG 1380
ArB1362            ATGATTGTTAATGACataggacatgaaactgacgaaAACAGAGCGGAAGTCGAGGTCACG 1380
ARB13565           ATGATTGTTAATGACATAGGACATGAAACTGACGAAAACAGAGCGAAAGTCGAGGTCACG 1380
ARB7701            ATGATTGTTAATGACATAGGACATGAAACTGACGAAAACAGAGCGAAAGTCGAGGTCACG 1380
ARB15076           ATGATTGTTAATGAC------------------GAAAATAGGGCGAAAGTCGAGGTCACG 1362
                   ***** *                          **   * ** **  * ** *** * **

P6-740             CCCAATTCACCAAGAGCCGAAGCCACCCTGGGAGGTTTTGGAAGCCTAGGACTTGATTGT 1440
CPC0740            CCCAATTCACCAAGAGCCGAAGCCACCCTGGGGGGTTTTGGGAGCCTAGGACTTGATTGT 1440
YAP2007            CCCAATTCACCAAGAGCTGAAGCCACCCTGGGGGGTTTTGGAAGCCTAGGACTTGATTGT 1440
FSS13025           CCCAATTCACCAAGAGCCGAAGCCACCCTGGGGGGTTTTGGAAGCCTAGGACTTGATTGT 1440
PLCal_ZV           CCCAATTCACCAAGAGCCGAAGCCACCCTGGGAGGTTTTGGAAGCCTAGGACTTGATTGT 1329
SV0127/14          CCCAATTCACCAAGAGCCGAAGCCACCCTGGGGGGTTTTGGAAGCCTAGGACTTGATTGT 1440
```

```
8375               CCCAATTCACCAAGAGCCGAAGCCACCCTGGGGGGTTTTGGAAGCCTAGGACTTGATTGT 1440
103344             CCCAATTCACCAAGAGCCGAAGCCACCCTGGGGGGTTTTGGAAGCCTAGGACTTGATTGT 1440
BrasilZKV2015      CCCAATTCACCAAGAGCCGAAGCCACCCTGGGGGGTTTTGGAAGCTTAGGACTTGATTGT 1440
GD01               CCCAATTCACCAAGAGCCGAAGCCACCCTGGGGGGTTTTGGAAGCCTAGGACTTGATTGT 1440
Martinique         CCCAATTCACCAAGAGCCGAAGCCACCCTGGGGGGTTTTGGAAGCCTAGGACTTGATTGT 1440
NatalRGN           CCCAATTCACCAAGAGCCGAAGCCACCCTGGGGGGTTTTGGAAGCCTAGGACTTGATTGT 1440
HPF2013            CCCAATTCACCAAGAGCCGAAGCCACCCTGGGGGGTTTTGGAAGCCTAGGACTTGATTGT 1440
SPH2015            CCCAATTCACCAAGAGCCGAAGCCACCCTGGGGGGTTTTGGAAGCCTAGGACTTGATTGT 1440
Haiti2014          CCCAATTCACCAAGAGCCGAAGCCACCCTGGGGGGTTTTGGAAGCCTAGGACTTGATTGT 1440
PRVABC59           CCCAATTCACCGAGAGCCGAAGCCACCCTGGGGGGTTTTGGAAGCCTAGGACTTGATTGT 1440
BeH819015          CCCAATTCACCAAGAGCCGAAGCCACCCTGGGGGGTTTTGGAAGCCTAGGACTTGATTGT 1440
Z1106033           CCCAATTCACCAAGAGCCGAAGCCACCCTGGGGGGGTTTGGAAGCCTAGGACTTGATTGT 1440
BEH819966          CCCAATTCACCAAGAGCCGAAGCCACCCTGGGGGGTTTTGGAAGCCTAGGACTTGATTGT 1440
SSABr              CCCAATTCACCAAGAGCCGAAGCCACCCTGGGGGGTTTTGGAAGCCTAGGACTTGATTGT 1440
Beh815744          CCCAATTCACCAAGAGCCGAAGCCACCCTGGGGGGTTTTGGAAGCCTAGGACTTGATTGT 1440
BEH818995          CCCAATTCACCAAGAGCCGAAGCCACCCTGGGGGGTTTTGGAAGCCTAGGACTTGATTGT 1440
IbH30656           CCCAATTCACCAAGAGCAGAAGCAACCTTGGGAGGTTTTGGAAGCCTGGGACTTGATTGT 1422
ArD128000          CCCAATTCACCAAGAGCAGAAGCAACCTTGGGAGGTTTTGGAAGCTTGGGACTTGACTGT 1440
ArD7117            CCCAATTCACCAAGAGCAGAAGCAACCTTGGGAGGTTTTGGAAGCTTGGGACTTGACTGT 1440
ARD_41519          CCCAATTCACCAAGAGCAGAAGCAACCTTGGGAGGTTTTGGAAGCTTGGGACTTGACTGT 1440
ARD157995          CCCAATTCACCAAGAGCAGAAGCAACCTTGGGAGGTTTTGGAAGCTTGGGACTTGACTGT 1440
MR_766             CCTAATTCACCAAGAGCGGAAGCAACCTTGGGAGGCTTTGGAAGCTTAGGACTTGACTGT 1428
ARD158084          CCTAATTCACCAAGAGCGGAAGCAACCTTGGGAGGCTTTGGAAGCTTAGGACTTGACTGT 1440
ArB1362            CCTAATTCACCAAGAGCAGAAGCAACCTTGGGAGGCTTTGGAAGTTTAGGACTTGATTGT 1440
ARB13565           CCCAATTCACCAAGAGCAGAAGCAACCTTGGGAGGCTTTGGAAGCTTAGGACTTGATTGT 1440
ARB7701            CCCAATTCACCAAGAGCAGAAGCAACCTTGGGAGGCTTTGGAAGCTTAGGACTTGATTGT 1440
ARB15076           CCCAATTCACCAAGAGCAGAAGCAACCTTGGGAGGCTTTGGAAGCTTAGGACTTGATTGT 1422
                   ** ******** ***** ***** *** **** ** ***** **  * ******** ***

P6-740             GAACCGAGGACAGGCCTTGACTTTTCAGATTTGTATTACTTGACTATGAATAACAAGCAT 1500
CPC0740            GAACCGAGGACAGGCCTTGACTTTTCAGATTTGTATTACCTGACTATGAATAACAAGCAC 1500
YAP2007            GAACCGAGGACAGGCCTTGACTTTTCAGATTTGTATTACTTGACTATGAATAACAAGCAC 1500
FSS13025           GAACCGAGGACAGGCCTTGACTTTTCAGATTTGTATTACTTGACTATGAATAACAAGCAC 1500
PLCal_ZV           GAACCGAGGACAGGCCTTGACTTTTCAGATTTGTATTACTTGACTATGAATAACAAGCAC 1389
SV0127/14          GAACCGAGGACAGGCCTTGACTTTTCAGATTTGTATTACTTGACTATGAACAACAAGCAC 1500
8375               GAACCGAGGACAGGCCTTGACTTTTCAGATTTGTATTACTTGACTATGAATAACAAGCAC 1500
103344             GAACCGAGGACAGGCCTTGACTTTTCAGATTTGTATTACTTGACTATGAATAACAAGCAC 1500
BrasilZKV2015      GAACCGAGGACAGGCCTTGACTTTTCAGATTTGTATTACTTGACTATGAATAACAAGCAC 1500
GD01               GAACCGAGGACAGGCCTTGACTTTTCAGATTTGTATTACTTGACTATGAATAACAAGCAC 1500
Martinique         GAACCGAGGACAGGCCTTGACTTTTCAGATTTGTATTACTTGACTATGAATAACAAGCAC 1500
NatalRGN           GAACCGAGGACAGGCCTTGACTTTTCAGATTTGTATTACTTGACTATGAATAACAAGCAC 1500
HPF2013            GAACCGAGGACAGGCCTTGACTTTTCAGATTTGTATTACTTGACTATGAATAACAAGCAC 1500
SPH2015            GAACCGAGGACAGGCCTTGACTTTTCAGATTTGTATTACTTGACTATGAATAACAAGCAC 1500
Haiti2014          GAACCGAGGACAGGCCTTGACTTTTCAGATTTGTATTACTTGACTATGAATAACAAGCAC 1500
PRVABC59           GAACCGAGGACAGGCCTTGACTTTTCAGATTTGTATTACTTGACTATGAATAACAAGCAC 1500
BeH819015          GAACCGAGGACAGGCCTTGACTTTTCAGATTTGTATTACTTGACTATGAATAACAAGCAC 1500
Z1106033           GAACCGAGGACAGGCCTTGACTTTTCAGATTTGTATTACTTGACTATGAATAACAAGCAC 1500
BEH819966          GAACCGAGGACAGGCCTTGACTTTTCAGATTTGTATTACTTGACTATGAATAACAAGCAC 1500
SSABr              GAACCGAGGACAGGCCTTGACTTTTCAGATTTGTATTACTTGACTATGAATAACAAGCAC 1500
Beh815744          GAACCGAGGACAGGCCTTGACTTTTCAGATTTGTATTACTTGACTATGAATAACAAGCAC 1500
BEH818995          GAACCGAGGACAGGCCTTGACTTTTCAGATTTGTATTACTTGACTATGAATAACAAGCAC 1500
IbH30656           GAACCAAGGACAGGCCTTGACTTTTCAGATCTGTATTACCTGACCATGAACAATAAGCAT 1482
ArD128000          GAACCAAGGACAGGCCTTGACTTCTCAGATCTGTATTATCTGACCATGAACAACAAGCAT 1500
ArD7117            GAACCAAGGACAGGCCTTGACTTCTCAGATCTGTATTACCTGACCATGAACAATAAGCAT 1500
ARD_41519          GAACCAAGGACAGGCCTTGACTTCTCAGATCTGTATTACCTGACCATGAACAATAAGCAT 1500
ARD157995          GAACCAAGGACAGGCCTTGACTTCTCAGATCTGTATTATCTGACCATGAACAACAAGCAT 1500
MR_766             GAACCAAGGACAGGCCTTGACTTTTCAGATCTGTATTACCTGACCATGAACAATAAGCAT 1488
ARD158084          GAACCAAGGACAGGCCTTGACTTTTCAGATCTGTATTACCTGACCATGAACAATAAGCAT 1500
ArB1362            GAACCAAGGACAGGCCTTGACTTTTCAGATTTGTATTACCTGACCATGAACAATAAGCAC 1500
ARB13565           GAACCAAGGACAGGCCTGGACTTTTCAGATTTGTACTACTTGACCATGAACAACAAGCAC 1500
ARB7701            GAACCAAGGACAGGCCTGGACTTTTCAGATTTGTACTACTTGACCATGAACAACAAGCAC 1500
ARB15076           GAACCAAGGACAGGCCTGGACTTTTCAGATTTGTACTACTTGACCATGAACAATAAGCAC 1482
                   ***** *********** ***** ****** **** **  **** ***** ** *****

P6-740             TGGTTGGTGCACAAGGAGTGGTTCCATGACATTCCACTACCTTGGCATGCTGGGGCAGAC 1560
CPC0740            TGGTTGGTTCACAAGGAGTGGTTCCACGACATTCCATTACCTTGGCATGCTGGGGCAGAC 1560
YAP2007            TGGTTGGTTCACAAGGAGTGGTTCCACGACATTCCATTACCTTGGCATGCTGGGGCAGAC 1560
FSS13025           TGGTTGGTTCACAAGGAGTGGTTCCACGACATTCCATTACCTTGGCATGCTGGGGCAGAC 1560
```

```
PLCal_ZV         TGGTTGGTTCACAAGGAGTGGTTCCACGACATTCCATTACCTTGGCACGCTGGGGCAGAC 1449
SV0127/14        TGGTTGGTTCACAAGGAGTGGTTCCACGACATTCCATTACCTTGGCACACTGGGGCAGAC 1560
8375             TGGTTGGTTCACAAGGAGTGGTTCCACGACATTCCATTACCTTGGCACGCTGGGGCAGAC 1560
103344           TGGTTGGTTCACAAGGAGTGGTTCCACGACATTCCATTACCTTGGCACGCTGGGGCAGAC 1560
BrasilZKV2015    TGGTTGGTTCACAAGGAGTGGTTCCACGACATTCCATTACCTTGGCACGCTGGGGCAGAC 1560
GD01             TGGTTGGTTCACAAGGAGTGGTTCCACGACATTCCATTACCTTGGCACGCTGGGGCAGAC 1560
Martinique       TGGTTGGTTCACAAGGAGTGGTTCCACGACATTCCATTACCTTGGCACGCTGGGGCAGAC 1560
NatalRGN         TGGTTGGTCCACAAGGAGTGGTTCCACGACATTCCATTACCTTGGCACGCTGGGGCAGAC 1560
HPF2013          TGGTTGGTTCACAAGGAGTGGTTCCACGACATTCCATTACCTTGGCACGCTGGGGCAGAC 1560
SPH2015          TGGTTGGTTCACAAGGAGTGGTTCCACGACATTCCATTACCTTGGCACGCTGGGGCAGAC 1560
Haiti2014        TGGTTGGTTCACAAGGAGTGGTTCCACGACATTCCATTACCTTGGCACGCTGGGGCAGAC 1560
PRVABC59         TGGTTGGTTCACAAGGAGTGGTTCCACGACATTCCATTACCTTGGCACGCTGGGGCAGAC 1560
BeH819015        TGGTTGGTTCACAAGGAGTGGTTCCACGACATTCCATTACCTTGGCACGCTGGGGCAGAC 1560
Z1106033         TGGCTGGTTCACAAGGAGTGGTTCCACGACATTCCATTACCTTGGCACGCTGGGGCAGAC 1560
BEH819966        TGGTTGGTTCACAAGGAGTGGTTCCACGACATTCCATTACCTTGGCACGCTGGGGCAGAC 1560
SSABr            TGGTTGGTTCACAAGGAGTGGTTCCACGACATTCCATTACCTTGGCACGCTGGGGCAGAC 1560
Beh815744        TGGTTGGTTCACAAGGAGTGGTTCCACGACATTCCATTACCTTGGCACGCTGGGGCAGAC 1560
BEH818995        TGGTTGGTTCACAAGGAGTGGTTCCACGACATTCCATTACCTTGGCACGCTGGGGCAGAC 1560
IbH30656         TGGTTGGTGCACAAAGAGTGGTTTCATGACATCCCATTACCTTGGCATTCTGGTGCAGAC 1542
ArD128000        CGGTTGGTGCGCAAGGAGTGGTTTCATGACATCCCATTACCTTGGCATGCTGGTGCAGAC 1560
ArD7117          TGGTTGGTGCACAAGGAGTGGTTTCATGACATCCCATTACCTTGGCATGCTGGTGCAGAC 1560
ARD_41519        TGGTTGGTGCACAAGGAGTGGTTTCATGACATCCCATTACCTTGGCATGCTGGTGCAGAC 1560
ARD157995        TGGTTGGTGCACAAGGAGTGGTTTCATGACATCCCATTACCTTGGCATGCTGGTGCAGAC 1560
MR_766           TGGTTGGTGCACAAAGAGTGGTTTCATGACATCCCATTGCCTTGGCATGCTGGGGCAGAC 1548
ARD158084        TGGTTGGTGCACAAAGAGTGGTTTCATGACATCCCATTGCCTTGGCATGCTGGGGCAGAC 1560
ArB1362          TGGTTAGTCCACAAAGAGTGGTTTCATGACATCCCATTGCCTTGGCATGCTGGGGCAGAC 1560
ARB13565         TGGTTGGTGCACAAAGAGTGGTTTCATGACATCCCATTGCCTTGGCATGCTGGGGCAGAT 1560
ARB7701          TGGTTGGTGCACAAAGAGTGGTTTCATGACATCCCATTGCCTTGGCATGCTGGGGCAGAT 1560
ARB15076         TGGTTAGTGCACAAAGAGTGGTTTCATGACATCCCATTGCCCTGGCATGCTGGGGCAGAC 1542
                  ** * ** * *** ******** ** ***** *** * ** *****  **** *****

P6-740           ACCGGAACTCCACATTGGAACAACAAAGAAGCATTGGTAGAGTTCAAGGACGCACATGCC 1620
CPC0740          ACTGGAACTCCACATTGGAACAACAAAGAAGCACTGGTAGAGTTCAAGGACGCACATGCA 1620
YAP2007          ACCGGAACTCCACATTGGAACAACAAAGAAGCATTGGTAGAGTTCAAGGACGCACATGCC 1620
FSS13025         ACCGGAACTCCACACTGGAACAACAAAGAAGCACTGGTAGAGTTCAAGGACGCACATGCC 1620
PLCal_ZV         ACCGGAACTCCACACTGGAACAACAAAGAAGCACTGGTAGAATTCAAGGATGCACATGCC 1509
SV0127/14        ACCGGAACTCCACACTGGAACAACAAAGAAGCACTGGTAGAGTTCAAGGACGCACATGCC 1620
8375             ACCGGAACTCCACACTGGAACAACAAAGAAGCACTGGTGGAGTTCAAGGACGCACATGCC 1620
103344           ACCGGAACTCCACACTGGAACAACAAAGAAGCACTGGTGGAGTTCAAGGACGCACATGCC 1620
BrasilZKV2015    ACCGGAACTCCACACTGGAACAACAAAGAAGCACTGGTAGAGTTCAAGGACGCACATGCC 1620
GD01             ACCGGAACTCCACACTGGAACAACAAAGAAGCACTGGTAGAGTTCAAGGACGCACATGCC 1620
Martinique       ACCGGAACTCCACACTGGAACAACAAAGAAGCACTGGTAGAGTTCAAGGACGCACATGCC 1620
NatalRGN         ACCGGAACTCCACACTGGAACAACAAAGAAGCACTGGTAGAGTTCAAGGACGCACATGCC 1620
HPF2013          ACCGGAACTCCACACTGGAACAACAAAGAAGCACTGGTAGAGTTCAAGGACGCACATGCC 1620
SPH2015          ACCGGAACTCCACACTGGAACAACAAAGAAGCACTGGTAGAGTTCAAGGACGCACATGCC 1620
Haiti2014        ACCGGAACTCCACACTGGAACAACAAAGAAGCACTGGTAGAGTTCAAGGACGCACATGCC 1620
PRVABC59         ACCGGAACTCCACACTGGAACAACAAAGAAGCACTGGTAGAGTTCAAGGACGCACATGCC 1620
BeH819015        ACCGGAACTCCACACTGGAACAACAAAGAAGCACTGGTAGAGTTCAAGGACGCACATGCC 1620
Z1106033         ACCGGAACTCCACACTGGAACAACAAAGAAGCACTGGTAGAGTTCAAGGACGCACATGCC 1620
BEH819966        ACCGGAACTCCACACTGGAACAACAAAGAAGCACTGGTAGAGTTCAAGGACGCACATGCC 1620
SSABr            ACCGGAACTCCACACTGGAACAACAAAGAAGCACTGGTAGAGTTCAAGGACGCACATGCC 1620
Beh815744        ACCGGAACTCCACACTGGAACAACAAAGAAGCACTGGTAGAGTTCAAGGACGCACATGCC 1620
BEH818995        ACCGGAACTCCACACTGGAACAACAAAGAAGCACTGGTAGAGTTCAAGGACGCACATGCC 1620
IbH30656         ACTGAAACTCCACACTGGAACAACAAAGAGGCACTGGTGGAGTTCAAGGACGCCCACGCC 1602
ArD128000        ACTGGAACTCCACACTGGAACAACAAAGAGGCATTGGTGGAGTTCAAGGACGCTCACGCC 1620
ArD7117          ACTGGAACTCCACACTGGAACAACAAAGAGGCATTGGTGGAGTTTAAGGACGCCCACGCC 1620
ARD_41519        ACTGGAACTCCACACTGGAACAACAAAGAGGCATTGGTGGAGTTCAAGGACGCCCACGCC 1620
ARD157995        ACTGGAACTCCACACTGGAACAACAAAGAGGCATTGGTGGAGTTCAAGGACGCCCACGCC 1620
MR_766           ACCGGAACTCCACACTGGAACAACAAAGAGGCATTGGTAGAATTCAAGGATGCCCACGCC 1608
ARD158084        ACCGGAACTCCACACTGGAACAACAAAGAGGCATTGGTAGAATTCAAGGATGCCCACGCC 1620
ArB1362          ACCGGAACTCCACACTGGAACAACAAAGAGGCATTGGTAGAGTTCAAGGATGCCCACGCC 1620
ARB13565         ACCGGAACTCCACACTGGAACAACAAGGAGGCATTGGTAGAGTTCAAAGATGCCCACGCC 1620
ARB7701          ACCGGAACTCCACACTGGAACAACAAGGAGGCATTGGTAGAGTTCAAAGATGCCCACGCC 1620
ARB15076         ACCGGAACTCCACATTGGAACAACAAGGAGGCATTGGTAGAGTTCAAAGATGCCCACGCT 1602
                 ** * ********* *********** ** *** **** ** ** ** ** ** ** **

P6-740           AAAAGGCAAACTGTCGTGGTTCTAGGGAGTCAAGAAGGAGCCGTTCACACGGCTCTTGCT 1680
CPC0740          AAAAGGCAAACTGTCGTGGTTCTAGGGAGTCAAGAAGGAGCAGTTCACACGGCCCTTGCT 1680
```

```
YAP2007             AAAAGGCAAACTGTCGTGGTTCTAGGGAGTCAAGAAGGAGCAGTTCACACGGCCCTTGCT 1680
FSS13025            AAAAGGCAGACTGTCGTGGTTCTAGGGAGTCAAGAAGGAGCAGTTCACACGGCCCTTGCT 1680
PLCal_ZV            AAAAGGCAAACTGTCGTGGTTCTAGGGAGTCAAGAGGGAGCAGTTCACACGGCCCTTGCT 1569
SV0127/14           AAAAGGCAAACTGTCGTGGTTCTAGGGAGTCAAGAAGGAGCAGTTCACACGGCCCTTGCT 1680
8375                AAAAGGCAAACTGTCGTGGTTCTAGGGAGTCAAGAAGGAGCAGTTCACACGGCCCTTGCT 1680
103344              AAAAGGCAAACTGTCGTGGTTCTAGGGAGTCAAGAAGGAGCAGTTCACACGGCCCTTGCT 1680
BrasilZKV2015       AAAAGGCAAACTGTCGTGGTTCTAGGGACTCAAGAAGGAGCAGTTCACACGGCCCTTGCT 1680
GD01                AAAAGGCAAACTGTCGTGGTTCTAGGGAGTCAAGAAGGAGCAGTTCACACGGCCCTTGCT 1680
Martinique          AAAAGGCAAACTGTCGTGGTTCTAGGGAGTCAAGAAGGAGCAGTTCACACGGCCCTTGCT 1680
NatalRGN            AAAAGGCAAACTGTCGTGGTTCTAGGGAGTCAAGAAGGAGCAGTTCACACGGCCCTTGCT 1680
HPF2013             AAAAGGCAAACTGTCGTGGTTCTAGGGAGTCAAGAAGGAGCAGTTCACACGGCCCTTGCT 1680
SPH2015             AAAAGGCAAACTGTCGTGGTTCTAGGGAGTCAAGAAGGAGCAGTTCACACGGCCCTTGCT 1680
Haiti2014           AAAAGGCAAACTGTCGTGGTTCTAGGGAGTCAAGAAGGAGCAGTTCACACGGCCCTTGCT 1680
PRVABC59            AAAAGGCAAACTGTCGTGGTTCTAGGGAGTCAAGAAGGAGCAGTTCACACGGCCCTTGCT 1680
BeH819015           AAAAGGCAAACTGTCGTGGTTCTAGGGAGTCAAGAAGGAGCAGTTCACACGGCCCTTGCT 1680
Z1106033            AAAAGGCAAACTGTCGTGGTTCTAGGGAGTCAAGAAGGAGCAGTTCACACGGCCCTTGCT 1680
BEH819966           AAAAGGCAAACTGTCGTGGTTCTAGGGAGTCAAGAAGGAGCAGTTCACACGGCCCTTGCT 1680
SSABr               AAAAGGCAAACTGTCGTGGTTCTAGGGAGTCAAGAAGGAGCAGTTCACACGGCCCTTGCT 1680
Beh815744           AAAAGGCAAACTGTCGTGGTTCTAGGGAGTCAAGAAGGAGCAGTTCACACGGCCCTTGCT 1680
BEH818995           AAAAGGCAAACTGTCGTGGTTCTAGGGAGTCAAGAAGGAGCAGTTCACACGGCCCTTGCT 1680
IbH30656            AAGAGGCAAACTGTTGTGGTTCTGGGGAGCCAAGAAGGAGCCGTTCACACGGCTCTCGCT 1662
ArD128000           AAGAGGCAAACTGTTGTGGTTCTGGGGAGCCAAGAGGGAGCTGTTCACACGGCCCTCGCT 1680
ArD7117             AAGAGGCAAACTGTTGTGGTTCTGGGGAGCCAAGAGGGAGCTGTTCACACGGCCCTCGCT 1680
ARD_41519           AAGAGGCAAACTGTTGTGGTTCTGGGGAGCCAAGAGGGAGCTGTTCATACGGCCCTCGCT 1680
ARD157995           AAGAGGCAAACTGTTGTGGTTCTGGGGAGCCAAGAGGGAGCTGTTCACACGGCCCTCGCT 1680
MR_766              AAGAGGCAAACCGTCGTCGTTCTGGGGAGCCAGGAAGGAGCCGTTCACACGGCTCTCGCT 1668
ARD158084           AAGAGGCAAACCGTCGTCGTTCTGGGGAGCCAGGAAGGAGCCGTTCACACGGCTCTCGCT 1680
ArB1362             AAGAGGCAAACCGTCGTCGTTCTGGGGAGCCAGGAAGGAGCTGTTCACACGGCTCTTGCT 1680
ARB13565            AAGAGGCAAACCGTCGTTGTTCTAGGGAGCCAGGAAGGAGCCGTTCACACGGCTCTTGCT 1680
ARB7701             AAGAGGCAAACCGTCGTTGTTCTAGGGAGCCAGGAAGGAGCCGTTCACACGGCTCTTGCT 1680
ARB15076            AAGAGGCAAACCGTCGTTGTTCTAGGGAGCCAGGAAGGAGCCGTTCACACGGCTCTTGCT 1662
                    ** ***** ** ** ** ***** ****  ** ** ***** ***** ***** ** ***

P6-740              GGAGCCCTGGAGGCTGAGATGGATGGTGCAAAGGGAAGGCTGTCCTCTGGCCACTTGAAA 1740
CPC0740             GGAGCTCTGGAGGCTGAGATGGATGGAGCCAAGGGAAGGCTGTCCTCTGGCCACTTGAAA 1740
YAP2007             GGAGCTCTGGAGGCTGAGATGGATGGTGCAAAGGGAAGGCTGTCCTCTGGCCACTTGAAA 1740
FSS13025            GGAGCTCTGGAGGCTGAGATGGATGGTGCAAAGGGAAGGCTGTCCTCTGGCCACTTGAAA 1740
PLCal_ZV            GGAGCTCTGGAGGCTGAGATGGATGGTGCAAAGGGAAGGCTGTCCTCTGGCCACTTGAAA 1629
SV0127/14           GGAGCTCTGGAGGCTGAGATGGATGGTGCAAAGGGAAGGCTGTCCTCTGGCCACTTGAAA 1740
8375                GGAGCTCTGGAGGCTGAGATGGATGGTGCAAAGGGAAGGCTGTCCTCTGGCCACTTGAAA 1740
103344              GGAGCTCTGGAGGCTGAGATGGATGGTGCAAAGGGAAGGCTGTCCTCTGGCCACTTGAAA 1740
BrasilZKV2015       GGAGCTCTGGAGGCTGAGATGGATGGTGCAAAGGGAAGGCTGTCCTCTGGCCACTTGAAA 1740
GD01                GGAGCTCTGGAGGCTGAGATGGATGGAGCAAAGGGAAGGCTGTCCTCTGGCCACTTGAAA 1740
Martinique          GGAGCTCTGGAGGCTGAGATGGATGGTGCAAAGGGAAGGCTGTCCTCTGGCCACTTGAAA 1740
NatalRGN            GGAGCTCTGGAGGCTGAGATGGATGGTGCAAAGGGAAGGCTGTCCTCTGGCCACTTGAAA 1740
HPF2013             GGAGCTCTGGAGGCTGAGATGGATGGTGCAAAGGGAAGGCTGTCCTCTGGCCACTTGAAA 1740
SPH2015             GGAGCTCTGGAGGCTGAGATGGATGGTGCAAAGGGAAGGCTGTCCTCTGGCCACTTGAAA 1740
Haiti2014           GGAGCTCTGGAGGCTGAGATGGATGGTGCAAAGGGAAGGCTGTCCTCTGGCCACTTGAAA 1740
PRVABC59            GGAGCTCTGGAGGCTGAGATGGATGGTGCAAAGGGAAGGCTGTCCTCTGGCCACTTGAAA 1740
BeH819015           GGAGCTCTGGAGGCTGAGATGGATGGTGCAAAGGGAAGGCTGTCCTCTGGCCACTTGAAA 1740
Z1106033            GGAGCTCTGGAGGCTGAGATGGATGGTGCAAAGGGAAGGCTGTCCTCTGGCCACTTGAAA 1740
BEH819966           GGAGCTCTGGAGGCTGAGATGGATGGTGCAAAGGGAAGGCTGTCCTCTGGCCACTTGAAA 1740
SSABr               GGAGCTCTGGAGGCTGAGATGGATGGTGCAAAGGGAAGGCTGTCCTCTGGCCACTTGAAA 1740
Beh815744           GGAGCTCTGGAGGCTGAGATGGATGGTGCAAAGGGAAGGCTGTCCTCTGGCCACTTGAAA 1740
BEH818995           GGAGCTCTGGAGGCTGAGATGGATGGTGCAAAGGGAAGGCTGTCCTCTGGCCACTTGAAA 1740
IbH30656            GGAGCTCTGGAGGCTGAGATGGATGGTGCGAAGGGAAGGCTATCCTCAGGCCATTTGAAA 1722
ArD128000           GGAGCTCTGGAGGCTGAGATGGATGGTGCAAAGGGAAGGCTATTCTCTGGCCATTTGAAA 1740
ArD7117             GGAGCTTTGGAGGCTGAGATGGATGGTGCAAAGGGAAGGCTATTCTCTGGCCATTTGAAA 1740
ARD_41519           GGAGCTTTGGAGGCTGAGATGGATGGTGCAAAGGGAAGGCTATTCTCTGGCCATTTGAAA 1740
ARD157995           GGAGCTCTGGAGGCTGAGATGGATGGTGCAAAGGGAAGGCTATTCTCTGGCCATTTGAAA 1740
MR_766              GGAGCTCTAGAGGCTGAGATGGATGGTGCAAAGGGAAGGCTGTTCTCTGGCCATTTGAAA 1728
ARD158084           GGAGCTCTAGAGGCTGAGATGGATGGTGCAAAGGGAAGGCTGTTCTCTGGCCATTTGAAA 1740
ArB1362             GGAGCTTTGGAGGCTGAGATGGATGGTGCAAAAGGAAGGCTGTTCTCTGGCCATTTGAAA 1740
ARB13565            GGAGCTCTGGAGGCTGAGATGGATGGTGCAAAAGGAAGGCTGTTCTCCGGCCATTTGAAA 1740
ARB7701             GGAGCTCTGGAGGCTGAGATGGATGGTGCAAAAGGAAGGCTGTTCTCCGGCCATTTGAAA 1740
ARB15076            GGAGCTCTGGAGGCTGAGATGGATGGTGCAAAAGGAAGGTTGTTCTCCGGCCATTTGAAA 1722
                    *****  * ***************** ** ** ****** * * *** ***** ******
```

```
P6-740          TGTCGCTTGAAAATGGACAAACTTAGATTGAAGGGCGTGTCATACTCCTTATGTACCGCG 1800
CPC0740         TGTCGCCTGAAAATGGATAAACTTAGATTGAAGGGCGTGTCATACTCCTTGTGCACTGCA 1800
YAP2007         TGTCGCCTGAAAATGGATAAACTTAGATTGAAGGGCGTGTCATACTCCTTGTGTACCGCA 1800
FSS13025        TGTCGCCTGAAAATGGATAAACTTAGATTGAAGGGCGTGTCATACTCCTTGTGTACCGCA 1800
PLCal_ZV        TGTCGCCTGAAAATGGATAAACTTAGATTGAAGGGCGTGTCATACTCCCTGTGTACCGCA 1689
SV0127/14       TGTCGCCTGAAAATGGATAAACTTAGATTGAAGGGCGTGTCATACTCCTTGTGTACCGCA 1800
8375            TGTCGCCTGAAAATGGATAAACTTAGATTGAAGGGCGTGTCATACTCCTTGTGTACCGCA 1800
103344          TGTCGCCTGAAAATGGATAAACTTAGATTGAAGGGCGTGTCATACTCCTTGTGTACCGCA 1800
BrasilZKV2015   TGTCGCCTGAAAATGGATAAACTTAGATTGAAGGGCGTGTCATACTCCTTGTGTACCGCA 1800
GD01            TGTCGCCTGAAAATGGATAAACTTAGATTGAAGGGCGTGTCATACTCCTTGTGTACTGCA 1800
Martinique      TGTCGCCTGAAAATGGATAAACTTAGATTGAAGGGCGTGTCATACTCCTTGTGTACCGCA 1800
NatalRGN        TGTCGCCTGAAAATGGATAAACTTAGATTGAAGGGCGTGTCATACTCCTTGTGTACCGCA 1800
HPF2013         TGTCGCCTGAAAATGGATAAACTTAGATTGAAGGGCGTGTCATACTCCTTGTGTACCGCA 1800
SPH2015         TGTCGCCTGAAAATGGATAAACTTAGATTGAAGGGCGTGTCATACTCCTTGTGTACCGCA 1800
Haiti2014       TGTCGCCTGAAAATGGATAAACTTAGATTGAAGGGCGTGTCATACTCCTTGTGTACCGCA 1800
PRVABC59        TGTCGCCTGAAAATGGATAAACTTAGATTGAAGGGCGTGTCATACTCCTTGTGTACTGCA 1800
BeH819015       TGTCGCCTGAAAATGGATAAACTTAGATTGAAGGGCGTGTCATACTCCTTGTGTACTGCA 1800
Z1106033        TGTCGCCTGAAAATGGATAAACTTAGATTGAAGGGCGTGTCATACTCCTTGTGTACTGCA 1800
BEH819966       TGTCGCCTGAAAATGGATAAACTTAGATTGAAGGGCGTGTCATACTCCTTGTGTACTGCA 1800
SSABr           TGTCGCCTGAAAATGGATAAACTTAGATTGAAGGGCGTGTCATACTCCTTGTGTACTGCA 1800
Beh815744       TGTCGCCTGAAAATGGATAAACTTAGATTGAAGGGCGTGTCATACTCCTTGTGTACTGCA 1800
BEH818995       TGTCGCCTGAAAATGGATAAACTTAGATTGAAGGGCGTGTCATACTCCTTGTGTACTGCA 1800
IbH30656        TGCCGCCTAAAAATGGACAAGCTTAGGTTGAAGGGTGTGTCATATTCCCTGTGTACCGCA 1782
ArD128000       TGCCGCCTAAAAATGGACAAGCTTAGGTTGAAGGGTGTGTCATATTCCCTGTGTACTGCA 1800
ArD7117         TGCCGCCTAAAAATGGACAAGCTTAGGTTGAAGGGTGTGTCATATTCCCTGTGTACCGCA 1800
ARD_41519       TGCCGCCTAAAAATGGACAAGCTTAGGTTGAAGGGTGTGTCATATTCCCTGTGTACCGCA 1800
ARD157995       TGCCGCCTAAAAATGGACAAGCTTAGGTTGAAGGGTGTGTCATATTCCCTGTGTACTGCA 1800
MR_766          TGCCGCCTAAAAATGGACAAGCTTAGATTGAAGGGCGTGTCATATTCCTTGTGCACTGCG 1788
ARD158084       TGCCGCCTAAAAATGGACAAGCTTAGATTGAAGGGCGTGTCATATTCCTTGTGCACTGCG 1800
ArB1362         TGCCGCCTAAAAATGGACAAGCTTAGATTGAAGGGCGTGTCATATTCCTTGTGCACCGCA 1800
ARB13565        TGCCGCTTAAAAATGGACAAGCTTAGATTGAAGGGCGTGTCATATTCCTTGTGCACCGCA 1800
ARB7701         TGCCGCTTAAAAATGGACAAGCTTAGATTGAAGGGCGTGTCATATTCCTTGTGCACCGCA 1800
ARB15076        TGCCGCTTAAAAATGGACAAGCTTAGATTGAAGGGCGTGTCATATTCCTTGTGCACCGCA 1782
                ** *** * ******** ** ***** ******** ******** *** * ** ** **

P6-740          GCGTTCACATTCACCAAGATCCCGGCTGAAACGCTGCATGGGACAGTCACAGTGGAGGTA 1860
CPC0740         GCGTTCACATTCACCAAGATCCCGGCTGAAACACTGCACGGGACAGTCACAGTGGAGGTA 1860
YAP2007         GCGTTCACATTCACCAAGATCCCGGCTGAAACACTGCACGGGACAGTCACAGTGGAGGTA 1860
FSS13025        GCGTTCACATTCACTAAGATCCCGGCTGAAACACTGCACGGGACAGTCACAGTGGAGGTA 1860
PLCal_ZV        GCGTTCACATTCACTAAGATCCCGGCTGAAACACTGCACGGGACAGTCACAGTGGAGGTA 1749
SV0127/14       GCGTTCACATTCACCAAGATCCCGGCTGAAACACTGCACGGGACAGTCACAGTGGAGGTA 1860
8375            GCGTTCACATTCACCAAGATCCCGGCTGAAACACTGCACGGGACAGTCACAGTGGAGGTA 1860
103344          GCGTTCACATTCACCAAGATCCCGGCTGAAACACTGCACGGGACAGTCACAGTGGAGGTA 1860
BrasilZKV2015   GCGTTCACATTCACCAAGATCCCGGCTGAAACACTGCACGGGACAGTCACAGTGGAGGTA 1860
GD01            GCGTTCACATTCACCAAGATCCCGGCTGAAACACTGCACGGGACAGTCACAGTGGAGGTA 1860
Martinique      GCGTTCACATTCACCAAGATCCCGGCTGAAACACTGCACGGGACAGTCACAGTGGAGGTA 1860
NatalRGN        GCGTTCACATTCACCAAGATCCCGGCTGAAACACTGCACGGGACAGTCACAGTGGAGGTA 1860
HPF2013         GCGTTCACATTCACCAAGATCCCGGCTGAAACACTGCACGGGACAGTCACAGTGGAGGTA 1860
SPH2015         GCGTTCACATTCACCAAGATCCCGGCTGAAACACTGCACGGGACAGTCACAGTGGAGGTA 1860
Haiti2014       GCGTTCACATTCACCAAGATCCCGGCTGAAACACTGCACGGGACAGTCACAGTGGAGGTA 1860
PRVABC59        GCGTTCACATTCACCAAGATCCCGGCTGAAACACTGCACGGGACAGTCACAGTGGAGGTA 1860
BeH819015       GCGTTCACATTCACCAAGATCCCGGCTGAAACACTGCACGGGACAGTCACAGTGGAGGTA 1860
Z1106033        GCGTTCACATTCACCAAGATCCCGGCTGAAACACTGCACGGGACAGTCACAGTGGAGGTA 1860
BEH819966       GCGTTCACATTCACCAAGATCCCGGCTGAAACACTGCACGGGACAGTCACAGTGGAGGTA 1860
SSABr           GCGTTCACATTCACCAAGATCCCGGCTGAAACACTGCACGGGACAGTCACAGTGGAGGTA 1860
Beh815744       GCGTTCACATTCACCAAGATCCCGGCTGAAACACTGCACGGGACAGTCACAGTGGAGGTA 1860
BEH818995       GCGTTCACATTCACCAAGATCCCGGCTGAAACACTGCACGGGACAGTCACAGTGGAGGTA 1860
IbH30656        GCGTTCACATTCACCAAGGTTCCAGCTGAAACATTGCATGGAACAGTCACAGTGGAGGTG 1842
ArD128000       GCGTTCACATTTACCAAGGTCCCAGCTGAAACATTGCATGGAACAGTTACAGTGGAGGTG 1860
ArD7117         GTGTGTACCGCAGCGAAGGTCCCAGCTGAAACATTGCATGGAACAGTCACAGTGGAGGTG 1860
ARD_41519       GCGTTCACATTTACCAAGGTCCCAGCTGAAACATTGCATGGAACAGTCACAGTGGAGGTG 1860
ARD157995       GCGTTCACATTTACCAAGGTCCCAGCTGAAACATTGCATGGAACAGTTACAGTGGAGGTG 1860
MR_766          GCATTCACATTCACCAAGGTCCCAGCTGAAACACTGCATGGAACAGTCACAGTGGAGGTG 1848
ARD158084       GCATTCACATTCACCAAGGTCCCAGCTGAAACACTGCATGGAACAGTCACAGTGGAGGTG 1860
ArB1362         GCGTTCACATTCACCAAGGTCCCAGCTGAAACACTGCATGGAACAGTCACAGTGGAGGTG 1860
ARB13565        GCGTTCACATTTACCAAGGTCCCAGCTGAAACATTGCATGGAACAGTTACAGTGGAGGTG 1860
ARB7701         GCGTTCACATTTACCAAGGTCCCAGCTGAAACATTGCATGGAACAGTTACAGTGGAGGTG 1860
ARB15076        GCGTTCACATTCACCAAGGTCCCAGCTGAAACATTGCATGGAACAGTTACAGTGGAGGTG 1842
```

```
                        *  *  **     * *** * ** ********  **** ** ***** ***********

P6-740                  CAGTATGCAGGGACAGATGGACCCTGCAAGGTTCCAGCTCAGATGGCGGTGGATATGCAA 1920
CPC0740                 CAGTACGCAGGGACAGATGGACCTTGCAAGGTTCCAGCTCAGATGGCGGTGGATATGCAA 1920
YAP2007                 CAGTACGCAGGGACAGATGGACCCTGCAAGGTTCCAGCTCAGATGGCGGTGGACATGCAA 1920
FSS13025                CAGTACGCAGGGACAGATGGACCTTGCAAGGTTCCAGCTCAGATGGCGGTGGACATGCAA 1920
PLCal_ZV                CAGTACGCAGGGACAGATGGACCTTGCAAGGTTCCAGCTCAGATGGCGGTGGACATGCAA 1809
SV0127/14               CAGTACGCAGGGACAGATGGACCTTGCAAGGTTCCAGCTCAGATGGCGGTGGACATGCAA 1920
8375                    CAGTACGCAGGGACAGATGGACCTTGCAAGGTTCCAGCTCAGATGGCGGTGGACATGCAA 1920
103344                  CAGTACGCAGGGACAGATGGACCTTGCAAGGTTCCAGCTCAGATGGCGGTGGACATGCAA 1920
BrasilZKV2015           CAGTACGCAGGGACAGATGGACCTTGCAAGGTTCCAGCTCAGATGGCGGTGGACATGCAA 1920
GD01                    CAGTACGCAGGGACAGATGGACCTTGCAAGGTTCCAGCTCAGATGGCGGTGGACATGCAA 1920
Martinique              CAGTACGCAGGGACAGATGGACCTTGCAAGGTTCCAGCCCAGATGGCGGTGGACATGCAA 1920
NatalRGN                CAGTACGCAGGGACAGATGGACCTTGCAAGGTTCCAGCTCAGATGGCGGTGGACATGCAA 1920
HPF2013                 CAGTACGCAGGGACAGATGGACCTTGCAAGGTTCCAGCTCAGATGGCGGTGGACATGCAA 1920
SPH2015                 CAGTACGCAGGGACAGATGGACCTTGCAAGGTTCCAGCTCAGATGGCGGTGGACATGCAA 1920
Haiti2014               CAGTACGCAGGGACAGATGGACCTTGCAAGGTTCCAGCTCAGATGGCGGTGGACATGCAA 1920
PRVABC59                CAGTACGCAGGGACAGATGGACCTTGCAAGGTTCCAGCTCAGATGGCGGTGGACATGCAA 1920
BeH819015               CAGTACGCAGGGACAGATGGACCTTGCAAGGTTCCAGCTCAGATGGCGGTGGACATGCAA 1920
Z1106033                CAGTACGCAGGGACAGATGGACCTTGCAAGGTTCCAGCTCAGATGGCGGTGGACATGCAA 1920
BEH819966               CAGTACGCAGGGACAGATGGACCTTGCAAGGTTCCAGCTCAGATGGCGGTGGACATGCAA 1920
SSABr                   CAGTACGCAGGGACAGATGGACCTTGCAAGGTTCCAGCTCAGATGGCGGTGGACATGCAA 1920
Beh815744               CAGTACGCAGGGACAGATGGACCTTGCAAGGTTCCAGCTCAGATGGCGGTGGACATGCAA 1920
BEH818995               CAGTACGCAGGGACAGATGGACCTTGCAAGGTTCCAGCTCAGATGGCGGTGGACATGCAA 1920
IbH30656                CAGTATGCAGGGAGGGATGGACCCTGCAAGGTCCCAGCCCAGATGGCGGTGGACATGCAG 1902
ArD128000               CAGTATGCAGGGACAGATGGACCTTGCAAGGTCCCAGCCCAGATGGCGGTGGACATGCAG 1920
ArD7117                 CAGTATGCAGGGACAGATGGACCCTGCAAGGTCCCAGCCCAGATGGCGGTAGACATGCAG 1920
ARD_41519               CAGTATGCAGGGACAGACGGACCCTGCAAAGTCCCAGCCCAGATGGCGGTGGACATGCAG 1920
ARD157995               CAGTCTGCAGGGACAGATGGACCCTGCAAGGTCCCAGCCCAGATGGCGGTGGACATGCAG 1920
MR_766                  CAGTATGCAGGGACAGATGGACCCTGCAAGATCCCAGTCCAGATGGCGGTGGACATGCAG 1908
ARD158084               CAGTATGCAGGGACAGATGGACCCTGCAAGGTCCCAGCCCAGATGGCGGTGGACATGCAG 1920
ArB1362                 CAGTATGCAGGGACAGATGGACCCTGCAAGGTCCCAGCCCAGATGGCGGTGGACATGCAG 1920
ARB13565                CAGTATGCAGGGACAGATGGACCCTGCAAGGTCCCAGCCCAGATGGCGGTGGACATGCAG 1920
ARB7701                 CAGTATGCAGGGACAGATGGACCCTGCAAGGTCCCAGCCCAGATGGCGGTGGACATGCAG 1920
ARB15076                CAGTATGCAGGGACAGATGGACCCTGCAAGGTCCCAGCTCAGATGGCGGTGGACATGCAG 1902
                        ****  *******  ** ***** *****  * ****  *********** ** *****

P6-740                  ACTCTGACCCCAGTTGGGAGGTTGATAACCGCTAACCCTGTGATCACTGAAAGCACTGAG 1980
CPC0740                 ACTCTGACCCCAGTTGGGAGGTTGATAACCGCTAACCCTGTAATCACTGAAAGCACCGAG 1980
YAP2007                 ACTCTGACCCCAGTTGGGAGGCTGATAACCGCTAACCCTGTAATCACTGAAAGCACTGAG 1980
FSS13025                ACTCTGACCCCAGTTGGGAGGTTGATAACCGCTAACCCTGTAATCACTGAAAGCACTGAG 1980
PLCal_ZV                ACTCTGACCCCAGTTGGGAGGTTGATAACCGCTAACCCTGTAATCACTGAAAGCACTGAG 1869
SV0127/14               ACTCTGACCCCAGTTGGGAGGTTGATAACCGCTAACCCCGTAATCACTGAAGGCACTGAG 1980
8375                    ACTCTGACCCCAGTTGGGAGGTTGATAACCGCTAACCCCGTAATCACTGAAAGCACTGAG 1980
103344                  ACTCTGACCCCAGTTGGGAGGTTGATAACCGCTAACCCCGTAATCACTGAAAGCACTGAG 1980
BrasilZKV2015           ACTCTGACCCCAGTTGGGAGGTTGATAACCGCTAACCCCGTAATCACTGAAAGCACTGAG 1980
GD01                    ACTCTGACCCCAGTTGGGAGGTTGATAACCGCTAACCCCGTAATCACTGAAAGCACTGAG 1980
Martinique              ACTCTGACCCCAGTTGGGAGGTTGATAACCGCTAACCCCGTAATCACTGAAAGCACTGAG 1980
NatalRGN                ACTCTGACCCCAGTTGGGAGGTTGATAACCGCTAACCCCGTAATCACTGAAAGCACTGAG 1980
HPF2013                 ACTCTGACCCCAGTTGGGAGGTTGATAACCGCTAACCCCGTAATCACTGAAAGCACTGAG 1980
SPH2015                 ACTCTGACCCCAGTTGGGAGGTTGATAACCGCTAACCCCGTAATCACTGAAAGCACTGAG 1980
Haiti2014               ACTCTGACCCCAGTTGGGAGGTTGATAACCGCTAACCCCGTAATCACTGAAAGCACTGAG 1980
PRVABC59                ACTCTGACCCCAGTTGGGAGGTTGATAACCGCTAACCCCGTAATCACTGAAAGCACTGAG 1980
BeH819015               ACTCTGACCCCAGTTGGGAGGTTGATAACCGCTAACCCCGTAATCACTGAAAGCACTGAG 1980
Z1106033                ACTCTGACCCCAGTTGGGAGGTTGATAACCGCTAACCCCGTAATCACTGAAAGCACTGAG 1980
BEH819966               ACTCTGACCCCAGTTGGGAGGTTGATAACCGCTAACCCCGTAATCACTGAAAGCACTGAG 1980
SSABr                   ACTCTGACCCCAGTTGGGAGGTTGATAACCGCTAACCCCGTAATCACTGAAAGCACTGAG 1980
Beh815744               ACTCTGACCCCAGTTGGGAGGTTGATAACCGCTAACCCCGTAATCACTGAAAGCACTGAG 1980
BEH818995               ACTCTGACCCCAGTTGGGAGGTTGATAACCGCTAACCCCGTAATCACTGAAAGCACTGAG 1980
IbH30656                ACCCTGACCCCAGTTGGAAGGCTGATAACGGCTAACCCTGTGATCACTGAAAGCACTGAG 1962
ArD128000               ACTCTGACCCCAGTTGGAAGGCTGATAACCGCCAACCCTGTGATCACTGAAAGTACTGAG 1980
ArD7117                 ACCCTGACCCCAGTTGGAAGGCTGATAACCGCCAACCCTGTGATCACTGAAAGTACTGAG 1980
ARD_41519               ACCCTGACCCCAGTTGGAAGGCTGATAACCGCCAATCCTGTGATCACTGAAAGTACTGAG 1980
ARD157995               ACCCTGACCCCAGTTGGAAGGCTGATAACCGCCAACCCCGTGATTACTGAAAGCACTGAG 1980
MR_766                  ACCCTGACCCCAGTTGGAAGGCTGATAACCGCCAACCCCGTGATTACTGAAAGCACTGAG 1968
ARD158084               ACCCTGACCCCAGTTGGAAGGCTGATAACCGCCAACCCCGTGATTACTGAAAGCACTGAG 1980
ArB1362                 ACTTTGACCCCAGTCGGAAGGCTGATAACTGCCAACCCCGTGATTACTGAAAGCACTGAA 1980
ARB13565                ACCCTGACCCCAGTCGGGAGGCTGATAACCGCCAACCCTGTGATTACTGAAAGCACTGAG 1980
```

```
ARB7701          ACCCTGACCCCAGTCGGGAGGCTGATAACCGCCAACCCTGTGATTACTGAAAGCACTGAG 1980
ARB15076         ACCCTGACCCCAGTCGGAAGGCTGATAACCGCCAACCCCGTGATTACTGAAAGCACTGAG 1962
                 **  ********** ** *** ******* ** ** ** ** ** ****** * ** **

P6-740           AATTCAAAGATGATGTTGGAACTTGACCCACCATTTGGGGATTCTTACATTGTCATAGGA 2040
CPC0740          AACTCTAAGATGATGCTGGAACTTGATCCACCATTTGGGGACTCTTACATTGTCATAGGA 2040
YAP2007          AACTCTAAGATGATGCTGGAACTTGATCCACCATTTGGGGACTCTTACATTGTCATAGGA 2040
FSS13025         AACTCCAAGATGATGCTGGAACTGGATCCACCATTTGGGGACTCTTACATTGTCATAGGA 2040
PLCal_ZV         AACTCTAAGATGATGCTGGAACTTGATCCACCATTTGGGGACTCTTACATTGTCATAGGA 1929
SV0127/14        AACTCTAAGATGATGCTGGAACTTGATCCACCATTTGGGGACTCTTACATTGTCATAGGA 2040
8375             AACTCTAAGATGATGCTGGAACTTGATCCACCATTTGGGGACTCTTACATTGTCATAGGA 2040
103344           AACTCTAAGATGATGCTGGAACTTGATCCACCATTTGGGGACTCTTACATTGTCATAGGA 2040
BrasilZKV2015    AACTCTAAGATGATGCTGGAACTTGATCCACCATTTGGGGACTCTTACATTGTCATAGGA 2040
GD01             AACTCTAAGATGATGCTGGAACTTGATCCACCATTTGGGGACTCTTACATTGTCATAGGA 2040
Martinique       AACTCTAAGATGATGCTGGAACTTGATCCACCATTTGGGGACTCTTACATTGTCATAGGA 2040
NatalRGN         AACTCTAAGATGATGCTGGAACTTGATCCACCATTTGGGGACTCTTACATTGTCATAGGA 2040
HPF2013          AACTCTAAGATGATGCTGGAACTTGATCCACCATTTGGGGACTCTTACATTGTCATAGGA 2040
SPH2015          AACTCTAAGATGATGCTGGAACTTGATCCACCATTTGGGGACTCTTACATTGTCATAGGA 2040
Haiti2014        AACTCTAAGATGATGCTGGAACTTGATCCACCATTTGGGGACTCTTACATTGTCATAGGA 2040
PRVABC59         AACTCTAAGATGATGCTGGAACTTGATCCACCATTTGGGGACTCTTACATTGTCATAGGA 2040
BeH819015        AACTCTAAGATGATGCTGGAACTTGATCCACCATTTGGGGACTCTTACATTGTCATAGGA 2040
Z1106033         AACTCTAAGATGATGCTGGAACTTGATCCACCATTTGGGGACTCTTACATTGTCATAGGA 2040
BEH819966        AACTCTAAGATGATGCTGGAACTTGATCCACCATTTGGGGACTCTTACATTGTCATAGGA 2040
SSABr            AACTCTAAGATGATGCTGGAACTTGATCCACCATTTGGGGACTCTTACATTGTCATAGGA 2040
Beh815744        AACTCTAAGATGATGCTGGAACTTGATCCACCATTTGGGGACTCTTACATTGTCATAGGA 2040
BEH818995        AACTCTAAGATGATGCTGGAACTTGATCCACCATTTGGGGACTCTTACATTGTCATAGGA 2040
IbH30656         AATTCAAAGATGATGTTGGAGCTCGACCCACCATTTGGGGATTCTTACATTGTCATAGGA 2022
ArD128000        AATTCAAAGATGATGTTGGAACTTGACCCACCATTTGGGGATTCTTACATTGTCATAGGA 2040
ArD7117          AATTCAAAGATGATGTTGGAGCTCGACCCACCATTTGGGGATTCTTACATTGTCATAGGA 2040
ARD_41519        AATTCAAAGATGATGTTGGAGCTCGACCCACCATTTGGGGATTCTTACATTGTCATAGGA 2040
ARD157995        AACTCAAAGATGATGTTGGAGCTTGACCCACCATTTGGGGATTCTTACATTGTCATAGGA 2040
MR_766           AACTCAAAGATGATGTTGGAGCTTGACCCACCATTTGGGGATTCTTACATTGTCATAGGA 2028
ARD158084        AACTCAAAGATGATGTTGGAGCTTGACCCACCATTTGGGGATTCTTACATTGTCATAGGA 2040
ArB1362          AATTCAAAGATGATGTTGGAGCTCGACCCACCATTTGGGGATTCTTACATTGTCATAGGA 2040
ARB13565         AATTCAAAGATGATGTTGGAGCTCGACCCACCATTTGGGGATTCTTACATTGTCATAGGA 2040
ARB7701          AATTCAAAGATGATGTTGGAGCTCGACCCACCATTTGGGGATTCTTACATTGTCATAGGA 2040
ARB15076         AATTCAAAGATGATGTTGGAGCTCGACCCACCATTTGGGGATTCTTACATTGTCATAGGA 2022
                 ** ** ********* **** ** ** ************** ******************

P6-740           GTTGGGGATAAGAAGATCACCCACCACTGGcACAGGAGTGGCAGCACCATCGGAAAAGCA 2100
CPC0740          GTCGGGGAGAAGAAGATCACCCATCACTGGCACAGGAGTGGCAGCACCATTGGAAAAGCA 2100
YAP2007          GTCGGGGAGAAGAAGATCACCCATCACTGGCACAGGAGTGGCAGCACCATTGGAAAAGCA 2100
FSS13025         GTCGGGGAAAAGAAGATCACCCACCACTGGCACAGGAGTGGCAGCACCATTGGAAAAGCA 2100
PLCal_ZV         GTCGGGGAGAAGAAGATCACCCACCACTGGCACAGGAGTGGCAGCACCATTGGAAAAGCA 1989
SV0127/14        GTCGGGGAGAAGAAGATCACCCACCACTGGCACAGGAGTGGCAGCACCATTGGAAAAGCA 2100
8375             GTCGGGGAGAAGAAGATCACCCACCACTGGCACAGGAGTGGCAGCACCATTGGAAAAGCA 2100
103344           GTCGGGGAGAAGAAGATCACCCACCACTGGCACAGGAGTGGCAGCACCATTGGAAAAGCA 2100
BrasilZKV2015    GTCGGGGAGAAGAAGATCACCCACCACTGGCACAGGAGTGGCAGCACCATTGGAAAAGCA 2100
GD01             GTCGGGGAGAAGAAGATCACCCACCACTGGCACAGGAGTGGCAGCACCATTGGAAAAGCA 2100
Martinique       GTCGGGGAGAAGAAGATCACCCACCACTGGCACAGGAGTGGCAGCACCATTGGAAAAGCA 2100
NatalRGN         GTCGGGGAGAAGAAGATCACCCACCACTGGCACAGGAGTGGCAGCACCATTGGAAAAGCA 2100
HPF2013          GTCGGGGAGAAGAAGATCACCCACCACTGGCACAGGAGTGGCAGCACCATTGGAAAAGCA 2100
SPH2015          GTCGGGGAGAAGAAGATCACCCACCACTGGCACAGGAGTGGCAGCACCATTGGAAAAGCA 2100
Haiti2014        GTCGGGGAGAAGAAGATCACCCACCACTGGCACAGGAGTGGCAGCACCATTGGAAAAGCA 2100
PRVABC59         GTCGGGGAGAAGAAGATCACCCACCACTGGCACAGGAGTGGCAGCACCATTGGAAAAGCA 2100
BeH819015        GTCGGGGAGAAGAAGATCACCCACCACTGGCACAGGAGTGGCAGCACCATTGGAAAAGCA 2100
Z1106033         GTCGGGGAGAAGAAGATCACCCACCACTGGCACAGGAGTGGCAGCACCATTGGAAAAGCA 2100
BEH819966        GTCGGGGAGAAGAAGATCACCCACCACTGGCACAGGAGTGGCAGCACCATTGGAAAAGCA 2100
SSABr            GTCGGGGAGAAGAAGATCACCCACCACTGGCACAGGAGTGGCAGCACCATTGGAAAAGCA 2100
Beh815744        GTCGGGGAGAAGAAGATCACCCACCACTGGCACAGGAGTGGCAGCACCATTGGAAAAGCA 2100
BEH818995        GTCGGGGAGAAGAAGATCACCCACCACTGGCACAGGAGTGGCAGCACCATTGGAAAAGCA 2100
IbH30656         GTCGGGGACAAGAAAATCACCCATCACTGGCATCGGAGTGGTAGCATCATCGGAAAGGCA 2082
ArD128000        GTCGGGGACAAGAAAATCACCCATCACTGGCTCAAGAAAGGGAGCTCCATCGGAAAGGCA 2100
ArD7117          GTCGGGGACAAGAAAATCACCCATCACTGGCATCGGAGTGGTAGCACCATCGGAAAGGCA 2100
ARD_41519        GTCGGGGACAAGAAAATCACCCATCACTGGCATCGGAGTGGTAGCACCATCGGAAAGGCA 2100
ARD157995        GTTGGGGACAAGAAAATCACCCACCACTGGCATAGGAGTGGTAGCACCATCGGAAAGGCA 2100
MR_766           GTTGGGGACAAGAAAATCACCCACCACTGGCATAGGAGTGGTAGCACCATCGGAAAGGCA 2088
ARD158084        GTTGGGGACAAGAAAATCACCCACCACTGGCATAGGAGTGGTAGCACCATCGGAAAGGCA 2100
```

```
ArB1362         GTTGGAGATAAGAAAATCACCCACCACTGGCATAGGAGTGGTAGCACCATCGGAAAAGCA 2100
ARB13565        GTTGGGGACAAGAAAATCACCCATCACTGGCATAGGAGTGGTAGCACCATCGGAAAAGCA 2100
ARB7701         GTTGGGGACAAGAAAATCACCCATCACTGGCATAGGAGTGGTAGCACCATCGGAAAAGCA 2100
ARB15076        GTTGGGGACAAGAAAATTACCCACCACTGGCATAGGAGTGGTAGCACCATCGGAAAAGCA 2082
                ** ** ** ***** ** ***** *******    **  ** ***  *** ***** ***

P6-740          TTTGAAGCCACTGTGAGAGGCGCCAAGAGAATGGCAGTCTTGGGAGACACAGCCTGGGAC 2160
CPC0740         TTTGAAGCCACTGTGAGAGGTGCCAAGAGAATGGCAGTCTTGGGAGACACAGCCTGGGAC 2160
YAP2007         TTTGAAGCCACTGTGAGAGGTGCCAAGAGAATGGCAGTCTTGGGAGACACAGCCTGGGAT 2160
FSS13025        TTTGAAGCCACTGTGAGAGGTGCCAAGAGAATGGCAGTCTTGGGAGACACAGCCTGGGAC 2160
PLCal_ZV        TTTGAAGCCACTGTGAGAGGTGCCAAGAGAATGGCAGTCTTGGGAGACACAGCCTGGGAC 2049
SV0127/14       TTTGAAGCCACTGTGAGAGGTGCCAAGAGAATGGCAGTCTTGGGAGACACAGCCTGGGAC 2160
8375            TTTGAAGCCACTGTGAGAGGTGCCAAGAGAATGGCAGTCTTGGGAGACACAGCCTGGGAC 2160
103344          TTTGAAGCCACTGTGAGAGGTGCCAAGAGAATGGCAGTCTTGGGAGACACAGCCTGGGAC 2160
BrasilZKV2015   TTTGAAGCCACTGTGAGAGGTGCCAAGAGAATGGCAGTCTTGGGAGACACAGCCTGGGAC 2160
GD01            TTTGAAGCCACTGTGAGAGGTGCCAAGAGAATGGCAGTCTTGGGAGACACAGCCTGGGAC 2160
Martinique      TTTGAAGCCACTGTGAGAGGTGCCAAGAGAATGGCAGTCTTGGGAGACACAGCCTGGGAC 2160
NatalRGN        TTTGAAGCCACTGTGAGAGGTGCCAAGAGAATGGCAGTCTTGGGAGACACAGCCTGGGAC 2160
HPF2013         TTTGAAGCCACTGTGAGAGGTGCCAAGAGAATGGCAGTCTTGGGAGACACAGCCTGGGAC 2160
SPH2015         TTTGAAGCCACTGTGAGAGGTGCCAAGAGAATGGCAGTCTTGGGAGACACAGCCTGGGAC 2160
Haiti2014       TTTGAAGCCACTGTGAGAGGTGCCAAGAGAATGGCAGTCTTGGGAGACACAGCCTGGGAC 2160
PRVABC59        TTTGAAGCCACTGTGAGAGGTGCCAAGAGAATGGCAGTCTTGGGAGACACAGCCTGGGAC 2160
BeH819015       TTTGAAGCCACTGTGAGAGGTGCCAAGAGAATGGCAGTCTTGGGAGACACAGCCTGGGAC 2160
Z1106033        TTTGAAGCCACTGTGAGAGGTGCCAAGAGAATGGCAGTCTTGGGAGACACAGCCTGGGAC 2160
BEH819966       TTTGAAGCCACTGTGAGAGGTGCCAAGAGAATGGCAGTCTTGGGAGACACAGCCTGGGAC 2160
SSABr           TTTGAAGCCACTGTGAGAGGTGCCAAGAGAATGGCAGTCTTGGGAGACACAGCCTGGGAC 2160
Beh815744       TTTGAAGCCACTGTGAGAGGTGCCAAGAGAATGGCAGTCTTGGGAGACACAGCCTGGGAC 2160
BEH818995       TTTGAAGCCACTGTGAGAGGTGCCAAGAGAATGGCAGTCTTGGGAGACACAGCCTGGGAC 2160
IbH30656        TTTGAAGCCACTGTGAGAGGCGCCAAGAGAATGGCAGTCTTGGGAGACACAGCCTGGGAC 2142
ArD128000       TTTGAAGCCACTGTGAGAGGTGCCAAGAGAATGGCAGTCTTGGGGGACACAGCCTGGGAC 2160
ArD7117         TTTGAAGCCACTGTGAGAGGTGCCAAGAGAATGGCAGTCTTGGGGGACACAGCCTGGGAC 2160
ARD_41519       TTTGAAGCCACTGTGAGAGGTGCCAAGAGAATGGCAGTCTTGGGGGACACAGCCTGGGAC 2160
ARD157995       TTTGAAGCCACTGTGAGAGGCGCCAAGAGAATGGCAGTCCTGGGGGATACAGCCTGGGAC 2160
MR_766          TTTGAGGCCACTGTGAGAGGCGCCAAGAGAATGGCAGTCCTGGGGGATACAGCCTGGGAC 2148
ARD158084       TTTGAAGCCACTGTGAGAGGCGCCAAGAGAATGGCAGTCCTGGGGGATACAGCCTGGGAC 2160
ArB1362         TTTGAGGCCACTGTGAGAGGTGCCAAGAGAATGGCAGTTTTGGGGGACACAGCTTGGGAC 2160
ARB13565        TTTGAGGCCACTGTGAGAGGTGCCAAGAGAATGGCAGTTCTGGGGGATACAGCCTGGGAC 2160
ARB7701         TTTGAGGCCACTGTGAGAGGTGCCAAGAGAATGGCAGTTCTGGGGGATACAGCCTGGGAC 2160
ARB15076        TTTGAGGCCACTGTGAGAGGTGCCAAGAGAATGGCAGTTCTGGGGGACACAGCCTGGGAC 2142
                ***** ************** *****************  **** ** ***** *****

P6-740          TTTGGATCAGTCGGAGGTGCTCTCAACTCATTGGGCAAGGGCATCCATCAAATTTTTGGA 2220
CPC0740         TTTGGATCAGTTGGGGGTGCTCTCAACTCATTGGGCAAGGGCATCCATCAAATTTTTGGA 2220
YAP2007         TTTGGATCAGTTGGAGGTGCTCTCAACTCATTGGGCAAGGGCATCCATCAAATTTTTGGA 2220
FSS13025        TTTGGATCAGTTGGGGGTGCTCTCAACTCACTGGGCAAGGGCATCCATCAAATTTTTGGA 2220
PLCal_ZV        TTTGGATCAGTTGGAGGCGCTCTCAACTCATTGGGCAAGGGCATCCATCAAATTTTTGGA 2109
SV0127/14       TTTGGATCAGTTGGAGGCGTTCTTAACTCATTGGGCAAGGGCATCCATCAAATTTTTGGA 2220
8375            TTTGGATCAGTTGGAGGCGCTCTCAACTCATTGGGCAAGGGCATCCATCAAATTTTTGGA 2220
103344          TTTGGATCAGTTGGAGGCGCTCTCAACTCATTGGGCAAGGGCATCCATCAAATTTTTGGA 2220
BrasilZKV2015   TTTGGATCAGTTGGAGGCGCTCTCAACTCATTGGGCAAGGGCATCCATCAAATTTTTGGA 2220
GD01            TTTGGATCAGTTGGAGGCGCTCTCAACTCATTGGGCAAGGGCATCCATCAAATTTTTGGA 2220
Martinique      TTTGGATCAGTTGGAGGCGCTCTCAACTCATTGGGCAAGGGCATCCATCAAATTTTTGGA 2220
NatalRGN        TTTGGATCAGTTGGAGGCGCTCTCAACTCATTGGGCAAGGGCATCCATCAAATTTTTGGA 2220
HPF2013         TTTGGATCAGTTGGAGGCGCTCTCAACTCATTGGGCAAGGGCATCCATCAAATTTTTGGA 2220
SPH2015         TTTGGATCAGTTGGAGGCGCTCTCAACTCATTGGGCAAGGGCATCCATCAAATTTTTGGA 2220
Haiti2014       TTTGGATCAGTTGGAGGCGCTCTCAACTCATTGGGCAAGGGCATCCATCAAATTTTTGGA 2220
PRVABC59        TTTGGATCAGTTGGAGGCGCTCTCAACTCATTGGGCAAGGGCATCCATCAAATTTTTGGA 2220
BeH819015       TTTGGATCAGTTGGAGGCGCTCTCAACTCATTGGGCAAGGGCATCCATCAAATTTTTGGA 2220
Z1106033        TTTGGATCAGTTGGAGGCGCTCTCAACTCATTGGGCAAGGGCATCCATCAAATCTTTGGA 2220
BEH819966       TTTGGATCAGTTGGAGGCGCTCTCAACTCATTGGGCAAGGGCATCCATCAAATTTTTGGA 2220
SSABr           TTTGGATCAGTTGGAGGCGCTCTCAACTCATTGGGCAAGGGCATCCATCAAATTTTTGGA 2220
Beh815744       TTTGGATCAGTTGGAGGCGCTCTCAACTCATTGGGCAAGGGCATCCATCAAATTTTTGGA 2220
BEH818995       TTTGGATCAGTTGGAGGCGCTCTCAACTCATTGGGCAAGGGCATCCATCAAATTTTTGGA 2220
IbH30656        TTTGGATCAGTTGGGGGTGTGTTTAACTCATTGGGCAAGGGTATTCACCAGATCTTTGGA 2202
ArD128000       TTTGGATCAGTTGGAGGTGTGTTTAATTCATTGGGTAAGGGTGTTCATCAGATCTTTGGA 2220
ArD7117         TTTGGATCAGTTGGGGGTGTGTTTAATTCATTGGGTAAGGGTATTCACCAGATCTTTGGA 2220
ARD_41519       TTTGGATCAGTTGGGGGTGTGTTTAATTCATTGGGTAAGGGTATTCACCAGATCTTTGGA 2220
ARD157995       TTCGGATCAGTCGGGGGTGTGTTCAACTCACTGGGTAAGGGCATTCACCAGATTTTTGGA 2220
```

```
MR_766          TTCGGATCAGTCGGGGGTGTGTTCAACTCACTGGGTAAGGGCATTCACCAGATTTTTGGA 2208
ARD158084       TTCGGATCAGTCGGGGGTGTGTTCAACTCACTGGGTAAGGGCATTCACCAGATTTTTGGA 2220
ArB1362         TTTGGATCCGTCGGGGGTGTGTTCAACTCATTGGGCAAGGGCATCCACCAGATCTTTGGA 2220
ARB13565        TTCGGATCAGTCGGGGGCGTGTTCAACTCATTGGGCAAGGGTGTTCACCAGATTTTTGGA 2220
ARB7701         TTCGGATCAGTCGGGGGCGTGTTCAACTCATTGGGCAAGGGTGTTCACCAGATTTTTGGA 2220
ARB15076        TTTGGATCAGTCGGGGGCGTGTTTAACTCATTGGGCAAGGGCATCCACCAGATTTTTGGA 2202
                ** ***** ** ** ** *   * ** *** **** *****  * ** ** ** ******

P6-740          GCAGCTTTCAAATCATTGTTTGGAGGAATGTCCTGGTTCTCACAAATCCTCATAGGAACG 2280
CPC0740         GCAGCTTTCAAATCATTGTTCGGAGGAATGTCCTGGTTCTCACAAATTCTCATTGGAACG 2280
YAP2007         GCAGCTTTCAAATCATTGTTTGGAGGAATGTCCTGGTTCTCACAAATTCTCATTGGAACG 2280
FSS13025        GCAGCTTTCAAATCATTGTTTGGAGGAATGTCCTGGTTCTCACAAATTCTCATTGGAACG 2280
PLCal_ZV        GCAGCTTTCAAATCATTGTTTGGAGGAATGTCCTGGTTCTCACAAATTCTCATTGGAACG 2169
SV0127/14       GCAGCTTTCAAATCATTGTTTGGAGGAATGTCCTGGTTCTCACAAATTCTCATTGGAACG 2280
8375            GCTGCTTTCAAATCATTGTTTGGAGGAATGTCCTGGTTCTCACAAATTCTCATTGGAACG 2280
103344          GCTGCTTTCAAATCATTGTTTGGAGGAATGTCCTGGTTCTCACAAATTCTCATTGGAACG 2280
BrasilZKV2015   GCAGCTTTCAAATCATTGTTTGGAGGAATGTCCTGGTTCTCACAAATTCTCATTGGAACG 2280
GD01            GCAGCTTTCAAATCATTGTTTGGAGGAATGTCCTGGTTCTCACAAATTCTCATTGGAACG 2280
Martinique      GCAGCTTTCAAATCATTGTTTGGAGGAATGTCCTGGTTCTCACAAATTCTCATTGGAACG 2280
NatalRGN        GCAGCTTTCAAATCATTGTTTGGAGGAATGTCCTGGTTCTCACAAATCCTCATTGGAACG 2280
HPF2013         GCAGCTTTCAAATCATTGTTTGGAGGAATGTCCTGGTTCTCACAAATTCTCATTGGAACG 2280
SPH2015         GCAGCTTTCAAATCATTGTTTGGAGGAATGTCCTGGTTCTCACAAATTCTCATTGGAACG 2280
Haiti2014       GCAGCTTTCAAATCATTGTTTGGAGGAATGTCCTGGTTCTCACAAATTCTCATTGGAACG 2280
PRVABC59        GCAGCTTTCAAATCATTGTTTGGAGGAATGTCCTGGTTCTCACAAATTCTCATTGGAACG 2280
BeH819015       GCAGCTTTCAAATCATTGTTTGGAGGAATGTCCTGGTTCTCACAAATTCTCATTGGAACG 2280
Z1106033        GCAGCTTTCAAATCATTGTTTGGAGGAATGTCCTGGTTCTCACAAATTCTCATTGGAACG 2280
BEH819966       GCAGCTTTCAAATCATTGTTTGGAGGAATGTCCTGGTTCTCACAAATTCTCATTGGAACG 2280
SSABr           GCAGCTTTCAAATCATTGTTTGGAGGAATGTCCTGGTTCTCACAAATTCTCATTGGAACG 2280
Beh815744       GCAGCTTTCAAATCATTGTTTGGAGGAATGTCCTGGTTCTCACAAATTCTCATTGGAACG 2280
BEH818995       GCAGCTTTCAAATCATTGTTTGGAGGAATGTCCTGGTTCTCACAAATTCTCATTGGAACG 2280
IbH30656        GCAGCTTTCAAATCACTGTTCGGAGGAATGTCCTGGTTCTCACAGATCCTCATAGGCACA 2262
ArD128000       GCAGCTTTCAAATCACTGTTTGGAGGAATGTCCTGGTTCTCACAGATCCTCATAGGCACA 2280
ArD7117         GCAGCTTTTAAATCACTGTTTGGAGGAATGTCCTGGTTCTCACAGATCCTCATAGGCACA 2280
ARD_41519       GCAGCTTTCAAATCACTGTTTGGAGGAATGTCCTGGTTCTCACAGATCCTCATAGGCACA 2280
ARD157995       GCAGCCTTCAAATCACTGTTTGGAGGAATGTCCTGGTTCTCACAGATCCTCATAGGCACG 2280
MR_766          GCAGCCTTCAAATCACTGTTTGGAGGAATGTCCTGGTTCTCACAGATCCTCATAGGCACG 2268
ARD158084       GCAGCCTTCAAATCACTGTTTGGAGGAATGTCCTGGTTCTCACAGATCCTCATAGGCACG 2280
ArB1362         GCAGCCTTCAAATCACTGTTTGGAGGAATGTCCTGGTTCTCACAGATCCTCATAGGCACG 2280
ARB13565        GCAGCTTTCAAATCACTGTTTGGAGGAATGTCCTGGTTCTCACAGATCCTCATAGGCACG 2280
ARB7701         GCAGCTTTCAAATCACTGTTTGGAGGAATGTCCTGGTTCTCACAGATCCTCATAGGCACG 2280
ARB15076        GCAGCCTTCAAATCACTGTTTGGAGGAATGTCCTGGTTCTCACAGATCCTCATAGGCACG 2262
                ** ** ** ****** **** *********************** ** ***** ** **

P6-740          TTGCTGGTGTGGTTGGGTCTGAACACAAAGAATGGATCTATTTCCCTTACGTGCTTGGCC 2340
CPC0740         TTGCTGGTGTGGTTGGGTCTGAATACAAAGAATGGATCTATTTCCCTTACGTGCTTGGCC 2340
YAP2007         TTGCTGGTGTGGTTGGGTCTGAATACAAAGAATGGATCTATTTCCCTTACGTGCTTGGCC 2340
FSS13025        TTGCTGGTGTGGTTGGGTCTGAATACAAAGAATGGATCTATTTCCCTTATGTGCTTGGCC 2340
PLCal_ZV        TTGCTGATGTGGTTGGGTCTGAATACAAAGAATGGATCTATTTCCCTTATGTGCTTGGCC 2229
SV0127/14       TTGCTGATGTGGTTGGGTCTGAATACAAAGAATGGATCTATTTCCCTTATGTGCTTGGCC 2340
8375            TTGCTGATGTGGTTGGGTCTGAACACAAAGAATGGATCTATTTCCCTTATGTGCTTGGCC 2340
103344          TTGCTGATGTGGTTGGGTCTGAACACAAAGAATGGATCTATTTCCCTTATGTGCTTGGCC 2340
BrasilZKV2015   TTGCTGATGTGGTTGGGTCTGAACACAAAGAATGGATCTATTTCCCTTATGTGCTTGGCC 2340
GD01            TTGCTGATGTGGTTGGGTCTGAACACAAAGAATGGATCTATTTCCCTTATGTGCTTGGCC 2340
Martinique      TTGCTGATGTGGTTGGGTCTGAACACAAAGAATGGATCTATTTCCCTTATGTGCTTGGCC 2340
NatalRGN        TTGCTGATGTGGTTGGGTCTGAACACAAAGAATGGATCTATTTCCCTTATGTGCTTGGCC 2340
HPF2013         TTGCTGATGTGGTTGGGTCTGAACACAAAGAATGGATCTATTTCCCTTATGTGCTTGGCC 2340
SPH2015         TTGCTGATGTGGTTGGGTCTGAACACAAAGAATGGATCTATTTCCCTTATGTGCTTGGCC 2340
Haiti2014       TTGCTGATGTGGTTGGGTCTGAACACAAAGAATGGATCTATTTCCCTTATGTGCTTGGCC 2340
PRVABC59        TTGCTGATGTGGTTGGGTCTGAACACAAAGAATGGATCTATTTCCCTTATGTGCTTGGCC 2340
BeH819015       TTGCTGATGTGGTTGGGTCTGAACACAAAGAATGGATCTATTTCCCTTATGTGCTTGGCC 2340
Z1106033        TTGCTGATGTGGTTGGGTCTGAACGCAAAGAATGGATCTATTTCCCTTATGTGCTTGGCC 2340
BEH819966       TTGCTGATGTGGTTGGGTCTGAACACAAAGAATGGATCTATTTCCCTTATGTGCTTGGCC 2340
SSABr           TTGCTGATGTGGTTGGGTCTGAACACAAAGAATGGATCTATTTCCCTTATGTGCTTGGCC 2340
Beh815744       TTGCTGATGTGGTTGGGTCTGAACACAAAGAATGGATCTATTTCCCTTATGTGCTTGGCC 2340
BEH818995       TTGCTGATGTGGTTGGGTCTGAACACAAAGAATGGATCTATTTCCCTTATGTGCTTGGCC 2340
IbH30656        CTGTTGGTGTGGTTGGGTCTGAACACAAAGAATGGATCTATCTCCCTCACATGCTTGGCC 2322
ArD128000       CTGTTGGTGTGGTTGGGTCTGAACACAAAGAATGGATCTATCTCCCTCACATGCTTAGCC 2340
ArD7117         CTGTTGGTGTGGTTAGGTCTGAACACAAAGAATGGATCTATCTCCCTCACATGCTTAGCC 2340
```

```
ARD_41519           CTGTTGGTGTGGTTAGGTTTGAACACAAAGAATGGATCTATCTCCCTCACATGCTTAGCC 2340
ARD157995           CTGCTAGTGTGGTTAGGTTTGAACACAAAGAATGGATCTATCTCCCTCACATGCTTGGCC 2340
MR_766              CTGCTAGTGTGGTTAGGTTTGAACACAAAGAATGGATCTATCTCCCTCACATGCTTGGCC 2328
ARD158084           CTGCTAGTGTGGTTAGGTTTGAACACAAAGAATGGATCTATCTCCCTCACATGCTTGGCC 2340
ArB1362             CTGCTAGTGTGGTTGGGCTTGAACACAAAGAATGGATCCATCTCCCTCACATGCTTGGCC 2340
ARB13565            CTGCTGGTGTGGTTGGGTTTGAACACAAAGAATGGATCCATCTCCCTCACATGCTTGGCC 2340
ARB7701             CTGCTGGTGTGGTTGGGTTTGAACACAAAGAATGGATCCATCTCCCTCACATGCTTGGCC 2340
ARB15076            CTGCTGGTGTGGTTGGGTTTGAACACAAAGAATGGATCCATCTCCCTCACATGCTTGGCC 2322
                     ** *  ******* **  ****  ************* ** ***** *  ***** ***

P6-740              TTAGGGGGAGTGTTGATCTTCCTATCTACAGCCGTCTCTGCTGATGTGGGGTGTTCGGTG 2400
CPC0740             TTAGGGGGAGTGTTGATCTTCTTATCCACAGCCGTTTCTGCTGATGTGGGGTGCTCGGTG 2400
YAP2007             TTAGGGGGAGTGTTGATCTTTTTATCCACAGCCGTCTCTGCTGATGTGGGGTGCTCGGTG 2400
FSS13025            TTAGGGGGAGTGTTGATCTTCTTATCCACAGCCGTCTCTGCTGATGTGGGGTGCTCGGTG 2400
PLCal_ZV            TTAGGGGGAGTGTTGATCTTCTTATCCACAGCCGTCTCTGCTGATGTGGGGTGCTCGGTG 2289
SV0127/14           TTAGGGGGAGTGTTGATCTTCTTATCCACAGCCGTCTCCGCTGATGTGGGGTGCTCGGTG 2400
8375                TTAGGGGGAGTGTTGATCTTCTTATCCACAGCCGTCTCTGCTGATGTGGGGTGCTCGGTG 2400
103344              TTAGGGGGAGTGTTGATCTTCTTATCCACAGCCGTCTCTGCTGATGTGGGGTGCTCGGTG 2400
BrasilZKV2015       TTAGGGGGAGTGTTGATCTTCTTATCCACAGCCGTCTCTGCTGATGTGGGGTGCTCGGTG 2400
GD01                TTAGGGGGAGTGTTGATCTTCTTATCCACAGCCGTCTCTGCTGATGTGGGGTGCTCGGTG 2400
Martinique          TTAGGGGGAGTGTTGATCTTCTTATCCACAGCCGTCTCTGCTGATGTGGGGTGCTCGGTG 2400
NatalRGN            TTAGGGGGAGTGTTGATCTTCTTATCCACAGCCGTCTCTGCTGATGTGGGGTGCTCGGTG 2400
HPF2013             TTAGGGGGAGTGTTGATCTTCTTATCCACAGCTGTCTCTGCTGATGTGGGGTGCTCGGTG 2400
SPH2015             TTAGGGGGAGTGTTGATCTTCTTATCCACAGCCGTCTCTGCTGATGTGGGGTGCTCGGTG 2400
Haiti2014           TTAGGGGGAGTGTTGATCTTCTTATCCACAGCCGTCTCTGCTGATGTGGGGTGCTCGGTG 2400
PRVABC59            TTAGGGGGAGTGTTGATCTTCTTATCCACAGCCGTCTCTGCTGATGTGGGGTGCTCGGTG 2400
BeH819015           TTAGGGGGGGTGTTGATCTTCTTATCCACAGCCGTCTCTGCTGATGTGGGGTGCTCGGTG 2400
Z1106033            TTAGGGGGAGTGTTGATCTTCTTATCCACAGCCGTCTCTGCTGATGTGGGGTGCTCGGTG 2400
BEH819966           TTAGGGGGAGTGTTGATCTTCTTATCCACAGCCGTCTCTGCTGATGTGGGGTGCTCGGTG 2400
SSABr               TTAGGGGGAGTGTTGATCTTCTTATCCACAGCCGTCTCTGCTGATGTGGGGTGCTCGGTG 2400
Beh815744           TTAGGGGGAGTGTTGATCTTCTTATCCACAGCCGTCTCTGCTGATGTGGGGTGCTCGGTG 2400
BEH818995           TTAGGGGGAGTGTTGATCTTCTTATCCACAGCCGTCTCTGCTGATGTGGGGTGCTCGGTG 2400
IbH30656            TTGGGAGGAGTGATGATCTTCCTTTCCACGGCTGTTTCTGCTGATGTGGGGTGTTCGGTG 2382
ArD128000           CTGGGGGGAGTGATGATTTTCCTTTCCACGGCTGTTTCTGCTGATGTTGGGTGCTCGGTG 2400
ArD7117             CTGGGGGGAGTGATGATCTTCCTTTCCACGGCTGTTTCTGCTGATGTTGGGTGCTCGGTG 2400
ARD_41519           CTGGGGGGAGTGATGATCTTCCTTTCCACGGCTGTTTCTGCTGATGTTGGGTGCTCGGTG 2400
ARD157995           CTGGGGGGAGTGATGATCTTCCTCTCCACGGCTGTTTCTGCTGACGTGGGGTGCTCAGTG 2400
MR_766              CTGGGGGGAGTGATGATCTTCCTCTCCACGGCTGTTTCTGCTGACGTGGGGTGCTCAGTG 2388
ARD158084           CTGGGGGGAGTGATGATCTTCCTCTCCACGGCTGTTTCTGCTGACGTGGGGTGCTCAGTG 2400
ArB1362             CTGGGGGGAGTGATGATCTTCCTCTCCACGGCTGTTTCTGCTGATGTGGGGTGCTCGGTG 2400
ARB13565            CTGGGGGGAGTGATGATCTTCCTCTCCACGGCTGTCTCTGCTGACGTGGGGTGCTCGGTG 2400
ARB7701             CTGGGGGGAGTGATGATCTTCCTCTCCACGGCTGTCTCTGCTGACGTGGGGTGCTCGGTG 2400
ARB15076            CTGGGGGGAGTGATGATCTTCCTCTCCACGGCTGTCTCTGCTGACGTGGGGTGCTCGGTG 2382
                     * ** ** *** **** **  * ** ** ** ** ** ***** ** ***** ** ***

P6-740              GACTTCTCAAAGAAGGAAACGAGATGCGGTACGGGGGTGTTCGTCTATAACGACGTTGAA 2460
CPC0740             GACTTCTCAAAGAAGGAAACGAGATGCGGTACAGGGGTGTTCGTCTATAACGACGTTGAA 2460
YAP2007             GACTTCTCAAAGAAGGAAACGAGATGCGGTACGGGGGTGTTCGTCTATAACGACGTTGAT 2460
FSS13025            GACTTCTCAAAGAAGGAAACGAGATGCGGTACAGGGGTGTTCGTCTATAACGACGTTGAA 2460
PLCal_ZV            GACTTCTCAAAAAAAGAAACGAGATGCGGCACAGGGGTGTTCGTCTATAACGACGTTGAA 2349
SV0127/14           GACTTCTCAAAGAAGGAAACGAGATGCGGTACAGGGGTGTTCGTCTATAACGACGTTGAA 2460
8375                GACTTCTCAAAGAAGGAGACGAGATGCGGTACAGGGGTGTTCGTCTATAACGACGTTGAA 2460
103344              GACTTCTCAAAGAAGGAGACGAGATGCGGTACAGGGGTGTTCGTCTATAACGACGTTGAA 2460
BrasilZKV2015       GACTTCTCAAAGAAGGAGACGAGATGTGGTACAGGGGTGTTCGTCTATAACGACGTTGAA 2460
GD01                GACTTCTCAAAGAAGGAGACGAGATGCGGTACAGGGGTGTTTGTCTATAACGACGTTGAA 2460
Martinique          GACTTCTCAAAGAAGGAGACGAGATGTGGTACAGGGGTGTTCGTCTATAACGACGTTGAA 2460
NatalRGN            GACTTCTCAAAGAAGGAGACGAGATGCGGTACAGGGGTGTTCGTCTATAACGACGTTGAA 2460
HPF2013             GACTTCTCAAAGAAGGAGACGAGATGCGGTACAGGGGTGTTCGTCTATAACGACGTTGAA 2460
SPH2015             GACTTCTCAAAGAAGGAGACGAGATGCGGTACAGGGGTGTTCGTCTATAACGACGTTGAA 2460
Haiti2014           GACTTCTCAAAGAAGGAGACGAGATGCGGTACAGGGGTGTTCGTCTATAACGACGTTGAA 2460
PRVABC59            GACTTCTCAAAGAAGGAGACGAGATGCGGTACAGGGGTGTTCGTCTATAACGACGTTGAA 2460
BeH819015           GACTTCTCAAAGAAGGAGACGAGATGCGGTACAGGGGTGTTCGTCTATAACGACGTTGAA 2460
Z1106033            GACTTCTCAAAGAAGGAGACGAGATGCGGTACAGGGGTGTTCGTCTATAACGACGTTGAA 2460
BEH819966           GACTTCTCAAAGAAGGAGACGAGATGCGGTACAGGGGTGTTCGTCTATAACGACGTTGAA 2460
SSABr               GACTTCTCAAAGAAGGAGACGAGATGCGGTACAGGGGTGTTCGTCTATAACGACGTTGAA 2460
Beh815744           GACTTCTCAAAGAAGGAGACGAGATGCGGTACAGGGGTGTTCGTCTATAACGACGTTGAA 2460
BEH818995           GACTTCTCAAAGAAGGAGACGAGATGCGGTACAGGGGTGTTCGTCTATAACGACGTTGAA 2460
IbH30656            GACTTCTCAAAAAAGGAAACGAGATGTGGCACGGGGGTGTTCATCTACAATGACGTTGAA 2442
```

```
ArD128000       GACTTCTCAAAAAAGGAAACGAGATGTGGCACGGGGGTGTTCGTCTACAATGACGTTGAA 2460
ArD7117         GACTTCTCAAAAAAGGAAACGAGATGTGGCACGGGGGTGTTCGTCTACAATGACGTTGAA 2460
ARD_41519       GACTTCTCAAAAAAGGAAACGAGATGTGGCACGGGGGTGTTCGTCTACAATGACGTTGAA 2460
ARD157995       GACTTCTCAAAAAAGGAAACGAGATGTGGCACGGGGGTATTCATCTATAATGATGTTGAA 2460
MR_766          GACTTCTCAAAAAAGGAAACGAGATGTGGCACGGGGGTATTCATCTATAATGATGTTGAA 2448
ARD158084       GACTTCTCAAAAAGAGAAACGAGATGTGGCACGGGGGTATTCATCTATAATGATGTTGAA 2460
ArB1362         GACTTCTCAAAAAGAGAAACGAGATGTGGCACGGGGGTGTTCGTCTACAATGACGTTGAA 2460
ARB13565        GACTTCTCAAAAAGAGAAACGAGATGTGGCACGGGGGTGTTCGTCTACAATGATGTTGAA 2460
ARB7701         GACTTCTCAAAAAGAGAAACGAGATGTGGCACGGGGGTGTTCGTCTACAATGATGTTGAA 2460
ARB15076        GACTTCTCAAAAAGAGAAACGAGATGTGGCACGGGGGTGTTCGTCTACAATGATGTTGAA 2442
                *********** *  ** ******** ** ** ***** **  **** ** ** ***** 

P6-740          GCCTGGAGGGACAGGTACAAGTACCATCCTGACTCCCCTCGTAGATTGGCAGCAGCAGTC 2520
CPC0740         GCCTGGAGGGACAGGTACAAGTACCATCCTGACTCCCCTCGTAGATTGGCAGCAGCAGTC 2520
YAP2007         GCCTGGAGGGACAGGTACAAGTACCATCCTGACTCCCCTCGTAGATTAGCAGCAGCAGTC 2520
FSS13025        GCTTGGAGGGACAGGTACAAGTACCATCCTGACTCCCCTCGTAGATTGGCAGCAGCAGTC 2520
PLCal_ZV        GCCTGGAGAGACAGGTACAAGTACCATCCTGACTCCCCTCGTAGATTGGCAGCAGCAGTC 2409
SV0127/14       GCCTGGAGGGACAGGTACAAGTACCATCCTGACTCCCCTCGTAGATTGGCAGCAGTAGTC 2520
8375            GCCTGGAGGGACAGGTACAAGTACCATCCTGACTCCCCCCGTAGATTGGCAGCAGCAGTC 2520
103344          GCCTGGAGGGACAGGTACAAGTACCATCCTGACTCCCCCCGTAGATTGGCAGCAGCAGTC 2520
BrasilZKV2015   GCCTGGAGGGACAGGTACAAGTACCATCCTGACTCTCCCCGTAGATTGGCAGCAGCAGTC 2520
GD01            GCCTGGAGGGACAGGTACAAGTACCATCCTGACTCCCCCCGTAGATTGGCAGCAGCAGTC 2520
Martinique      GCCTGGAGGGACAGGTACAAGTACCATCCTGACTCCCCCCGTAGATTGGCAGCAGCAGTC 2520
NatalRGN        GCCTGGAGGGACAGGTACAAGTACCATCCTGACTCCCCCCGTAGATTGGCAGCAGCAGTC 2520
HPF2013         GCCTGGAGGGACAGGTACAAGTACCATCCTGACTCCCCCCGTAGATTGGCAGCAGCAGTC 2520
SPH2015         GCCTGGAGGGACAGGTACAAGTACCATCCTGACTCCCCCCGTAGATTGGCAGCAGCAGTC 2520
Haiti2014       GCCTGGAGGGACAGGTACAAGTACCATCCTGACTCCCCCCGTAGATTGGCAGCAGCAGTC 2520
PRVABC59        GCCTGGAGGGACAGGTACAAGTACCATCCTGACTCCCCCCGTAGATTGGCAGCAGCAGTC 2520
BeH819015       GCCTGGAGGGACAGGTACAAGTACCATCCTGACTCCCCCCGTAGATTGGCAGCAGCAGTC 2520
Z1106033        GCCTGGAGGGACAGGTACAAGTACCATCCTGACTCCCCCCGTAGATTGGCAGCAGCAGTC 2520
BEH819966       GCCTGGAGGGACAGGTACAAGTACCATCCTGACTCCCCCCGTAGATTGGCAGCAGCAGTC 2520
SSABr           GCCTGGAGGGACAGGTACAAGTACCATCCTGACTCCCCCCGTAGATTGGCAGCAGCAGTC 2520
Beh815744       GCCTGGAGGGACAGGTACAAGTACCATCCTGACTCCCCCCGTAGATTGGCAGCAGCAGTC 2520
BEH818995       GCCTGGAGGGACAGGTACAAGTACCATCCTGACTCCCCCCGTAGATTGGCAGCAGCAGTC 2520
IbH30656        GCCTGGAGGGATCGATACAGATACCATCCTGACTCCCCCCGCAGATTGGCAGCAGCTGTT 2502
ArD128000       GCCTGGAGGGACCGTTACAAGTATCATCCTGACTCCCCCCGCAGATTGGCAGCAGCTGTT 2520
ArD7117         GCCTGGAGGGACCGGTACAAGTACCATCCTGACTCCCCCCGCAGATTGGCAGCAGCTGTC 2520
ARD_41519       GCCTGGAGGGACCGGTACAAGTACCATCCTGACTCCCCCCGCAGATTGGCAGCAGCTGTT 2520
ARD157995       GCCTGGAGGGACCGGTACAAGTACCATCCTGACTCCCCCCGCAGATTGGCAGCAGCAGTC 2520
MR_766          GCCTGGAGGGACCGGTACAAGTACCATCCTGACTCCCCCCGCAGATTGGCAGCAGCAGTC 2508
ARD158084       GCCTGGAGGGACCGGTACAAGTACCATCCTGACTCCCCCCGCAGATTGGCAGCAGCAGTC 2520
ArB1362         GCCTGGAGGGATAGGTACAAGTACCATCCTGACTCCCCCCGCAGATTGGCAGCAGCAGTC 2520
ARB13565        GCCTGGAGGGACAGGTACAAGTACCATCCTGACTCCCCCCGCAGATTGGCAGCAGCAGTC 2520
ARB7701         GCCTGGAGGGACAGGTACAAGTACCATCCTGACTCCCCCCGCAGATTGGCAGCAGCAGTC 2520
ARB15076        GCCTGGAGGGACAGGTACAAGTACCATCCTGACTCCCCCCGCAGATTGGCAGCAGCAGTC 2502
                ** ***** **  * ****  ** *********** ** ** ***** *******  ** 

P6-740          AAGCAGGCCTGGGAAGATGGGATCTGTGGGATCTCCTCTGTTTCAAGAATGGAAAACATT 2580
CPC0740         AAGCAAGCCTGGGAAGATGGGATCTGTGGGATCTCCTCTGTCTCAAGAATGGAAAACATC 2580
YAP2007         AAGCAAGCCTGGGaAGATGGGATCTGTGGGATCTCCTCTGTTTCAAGAATGGAAAACATC 2580
FSS13025        AAGCAAGCCTGGGAAGATGGGATCTGTGGGATCTCCTCTGTTTCAAGAATGGAAAACATC 2580
PLCal_ZV        AAGCAAGCCTGGGAAGATGGGATCTGCGGGATCTCCTCTGTTTCAAGAATGGAAAACATC 2469
SV0127/14       AAGCAAGCCTGGGAAGATGGTATCTGTGGGATCTCCTCTGTTTCAAGAATGGAAAACATC 2580
8375            AAGCAAGCCTGGGAAGATGGTATCTGCGGGATCTCCTCTGTTTCAAGAATGGAAAACATC 2580
103344          AAGCAAGCCTGGGAAGATGGTATCTGCGGGATCTCCTCTGTTTCAAGAATGGAAAACATC 2580
BrasilZKV2015   AAGCAAGCCTGGGAAGATGGTATCTGCGGGATCTCCTCTGTTTCAAGAATGGAAAACATC 2580
GD01            AAGCAAGCCTGGGAAGATGGTATCTGCGGGATCTCCTCTGTTTCAAGAATGGAAAACATC 2580
Martinique      AAGCAAGCCTGGGAAGATGGTATCTGCGGGATCTCCTCTGTTTCAAGAATGGAAAACATC 2580
NatalRGN        AAGCAAGCCTGGGAAGATGGTATCTGCGGGATCTCCTCTGTTTCAAGAATGGAGAACATC 2580
HPF2013         AAGCAAGCCTGGGAAGATGGTATCTGTGGGATCTCCTCTGTTTCAAGAATGGAAAACATC 2580
SPH2015         AAGCAAGCCTGGGAAGATGGTATCTGCGGGATCTCCTCTGTTTCAAGAATGGAAAACATC 2580
Haiti2014       AAGCAAGCCTGGGAAGATGGTATCTGCGGGATCTCCTCTGTTTCAAGAATGGAAAACATC 2580
PRVABC59        AAGCAAGCCTGGGAAGATGGTATCTGCGGGATCTCCTCTGTTTCAAGAATGGAAAACATC 2580
BeH819015       AAGCAAGCCTGGGAAGATGGTATCTGCGGGATCTCCTCTGTTTCAAGAATGGAAAACATC 2580
Z1106033        AAGCAAGCCTGGGAAGATGGTATCTGCGGGATCTCCTCTGTTTCAAGAATGGAAAACATC 2580
BEH819966       AAGCAAGCCTGGGAAGATGGTATCTGCGGGATCTCCTCTGTTTCAAGAATGGAAAACATC 2580
SSABr           AAGCAAGCCTGGGAAGATGGTATCTGCGGGATCTCCTCTGTTTCAAGAATGGAAAACATC 2580
Beh815744       AAGCAAGCCTGGGAAGATGGTATCTGCGGGATCTCCTCTGTTTCAAGAATGGAAAACATC 2580
```

```
BEH818995          AAGCAAGCCTGGGAAGATGGTATCTGCGGGATCTCCTCTGTTTCAAGAATGGAAAACATC 2580
IbH30656           AAGCAGGCTTGGGAAGAGGGGATTTGTGGGATCTCCTCCGTTTCGAGAATGGAAAACATC 2562
ArD128000          AAGCAGGCTTGGGAAGAGGGGATTTGTGGGATCTCCTCCGTTTCGAGAATGGAAAACATC 2580
ArD7117            AAGCAGGCTTGGGAAGAGGGGATTTGTGGGATCTCCTCCGTTTCGAGAATGGAAAACATC 2580
ARD_41519          AAGCAGGCTTGGGAAGAGGGGATTTGTGGGATCTCCTCCGTTTCGAGAATGGAAAACATC 2580
ARD157995          AAGCAGGCCTGGGAAGAGGGGATCTGTGGGATCTCATCCGTTTCAAGAATGGAAAACATC 2580
MR_766             AAGCAGGCCTGGGAAGAGGGGATCTGTGGGATCTCATCCGTTTCAAGAATGGAAAACATC 2568
ARD158084          AAGCAGGCCTGGGAAGAGGGGATCTGTGGGATCTCATCCGTTTCAAGAATGGAAAACATC 2580
ArB1362            AAGCAGGCTTGGGAAGAGGGGATTTGTGGGATCTCCTCCGTTTCAAGAATGGAAAACATC 2580
ARB13565           AAGCAGGCTTGGGAAGAGGGGATTTGTGGGATCTCCTCCGTTTCAAGAATGGAAAACATC 2580
ARB7701            AAGCAGGCTTGGGAAGAGGGGATTTGTGGGATCTCCTCCGTTTCAAGAATGGAAAACATC 2580
ARB15076           AAGCAGGCTTGGGAAGAGGGAATTTGTGGGATCTCCTCCGTTTCAAGAATGGAAAACATC 2562
                   ***** ** ******** ** ** ** ******** ** ** ** ******** *****

P6-740             ATGTGGAGATCAGTAGAAGGGGAGCTCAACGCAATTCTGGAAGAGAATGGAGTTCAACTG 2640
CPC0740            ATGTGGAGATCAGTAGAAGGGGAGCTCAACGCAATCCTGGAAGAGAATGGAGTTCAACTG 2640
YAP2007            ATGTGGAGATCAGTAGAAGGGGAGCTCAACGCAATCCTGGAAGAGAATGGAGTTCAACTG 2640
FSS13025           ATGTGGAGATCAGTAGAAGGGGAGCTCAACGCAATCCTGGAAGAGAATGGAGTTCAACTG 2640
PLCal_ZV           ATGTGGAGATCAGTAGAAGGGGAGCTCAACGCAATCCTGGAAGAGAATGGAGTTCAACTG 2529
SV0127/14          ATGTGGAGATCAGTAGAAGGGGAGCTCAACGCAATCCTGGAAGAGAATGGAGTTCAACTG 2640
8375               ATGTGGAGATCAGTAGAAGGGGAGCTCAACGCAATCCTGGAAGAGAATGGAGTTCAACTG 2640
103344             ATGTGGAGATCAGTAGAAGGGGAGCTCAACGCAATCCTGGAAGAGAATGGAGTTCAACTG 2640
BrasilZKV2015      ATGTGGAGATCAGTAGAAGGGGAGCTTAACGCAATCCTGGAAGAGAATGGAGTTCAACTG 2640
GD01               ATGTGGAGATCAGTAGAAGGGGAGCTCAACGCAATCCTGGAAGAGAATGGAGTTCAACTG 2640
Martinique         ATGTGGAGATCAGTAGAAGGGGAGCTCAACGCAATCCTGGAAGAGAATGGAGTTCAACTG 2640
NatalRGN           ATGTGGAGATCAGTAGAAGGGGAGCTCAACGCAATCTTGGAAGAGAATGGAGTTCAACTG 2640
HPF2013            ATGTGGAGATCAGTAGAAGGGGAGCTCAACGCAATCCTGGAAGAGAATGGAGTTCAACTG 2640
SPH2015            ATGTGGAGATCAGTAGAAGGGGAGCTCAACGCAATCCTGGAAGAGAATGGAGTTCAACTG 2640
Haiti2014          ATGTGGAGATCAGTAGAAGGGGAGCTCAACGCAATCCTGGAAGAGAATGGAGTTCAACTG 2640
PRVABC59           ATGTGGAGATCAGTAGAAGGGGAGCTCAACGCAATCCTGGAAGAGAATGGAGTTCAACTG 2640
BeH819015          ATGTGGAGATCAGTAGAAGGGGAGCTCAACGCAATCCTGGAAGAGAATGGAGTTCAACTG 2640
Z1106033           ATGTGGAGATCAGTAGAAGGGGAGCTCAACGCAATCCTGGAAGAGAATGGAGTTCAACTG 2640
BEH819966          ATGTGGAGATCAGTAGAAGGGGAGCTCAACGCAATCCTGGAAGAGAATGGAGTTCAACTG 2640
SSABr              ATGTGGAGATCAGTAGAAGGGGAGCTCAACGCAATCCTGGAAGAGAATGGAGTTCAACTG 2640
Beh815744          ATGTGGAGATCAGTAGAAGGGGAGCTCAACGCAATCCTGGAAGAGAATGGAGTTCAACTG 2640
BEH818995          ATGTGGAGATCAGTAGAAGGGGAGCTCAACGCAATCCTGGAAGAGAATGGAGTTCAACTG 2640
IbH30656           ATGTGGAAATCAGTGGAAGGGGAGCTTAATGCGATCCTAGAGGAGAATGGAGTCCAACTG 2622
ArD128000          ATGTGGAAATCAGTGGAAGGGGAGCTTAATGCAATCCTAGAGGAGAATGGAGTCCAACTG 2640
ArD7117            ATGTGGAAATCAGTGGAAGGGGAGCTTAATGCAATCCTAGAGGAGAATGGAGTCCAACTG 2640
ARD_41519          ATGTGGAAATCAGTGGAAGGGGAGCTTAATGCAATCCTAGAGGAGAATGGAGTTCAACTG 2640
ARD157995          ATGTGGAAATCAGTAGAAGGGGAGCTCAATGCTATCCTAGAGGAGAATGGAGTTCAACTG 2640
MR_766             ATGTGGAAATCAGTAGAAGGGGAGCTCAATGCTATCCTAGAGGAGAATGGAGTTCAACTG 2628
ARD158084          ATGTGGAAATCAGTAGAAGGGGAGCTCAATGCTATCCTAGAGGAGAATGGAGTTCAACTG 2640
ArB1362            ATGTGGAAATCAGTGGAAGGGGAGCTCAATGCTATCTTAGAGGAGAATGGAGTTCAACTG 2640
ARB13565           ATGTGGAAATCAGTGGAAGGGGAGCTCAATGCTATCTTAGAGGAGAATGGAGTTCAACTG 2640
ARB7701            ATGTGGAAATCAGTGGAAGGGGAGCTCAATGCTATCTTAGAGGAGAATGGAGTTCAACTG 2640
ARB15076           ATGTGGAAATCAGTGGAAGGGGAGCTCAATGCTATCTTAGAGGAGAATGGAGTTCAACTG 2622
                   ******* ****** *********** ** ** **  * ** *********** ******

P6-740             ACGGTCGTTGTGGGATCTGTAAAAAACCCCATGTGGAGAGGTCCGCAGAGGTTGCCTGTG 2700
CPC0740            ACGGTCGTTGTGGGATCTGTAAAAAACCCCATGTGGAGAGGTCCACAGAGATTGCCCGTG 2700
YAP2007            ACGGTCGTTGTGGGATCTGTAAAAAACCCCATGTGGAGAGGTCCACAGAGATTGCCCGTG 2700
FSS13025           ACGGTCGTTGTGGGATCTGTAAAAAACCCCATGTGGAGAGGTCCACAGAGATTGCCCGTG 2700
PLCal_ZV           ACGGTCGTTGTGGGATCTGTAAAAAACCCCATGTGGAGAGGTCCACAGAGATTGCCCGTG 2589
SV0127/14          ACGGTCGTTGTGGGATCTGTAAAAAACCCCATGTGGAGAGGTCCACAGAGATTGCCCGTG 2700
8375               ACGGTCGTTGTGGGATCTGTAAAAAACCCCATGTGGAGAGCTCCACAGAGATTGCCCGTG 2700
103344             ACGGTCGTTGTGGGATCTGTAAAAAACCCCATGTGGAGAGCTCCACAGAGATTGCCCGTG 2700
BrasilZKV2015      ACGGTCGTTGTGGGATCTGTAAAAAACCCCATGTGGAGAGGTCCACAGAGATTGCCCGTG 2700
GD01               ACGGTCGTTGTGGGATCTGTAAAAAACCCCATGTGGAGAGGTCCACAGAGATTGCCCGTG 2700
Martinique         ACGGTCGTTGTGGGATCTGTAAAAAACCCCATGTGGAGAGGTCCACAGAGATTGCCCGTG 2700
NatalRGN           ACGGTCGTTGTGGGATCTGTAAAAAACCCCATGTGGAGAGGTCCACAGAGATTGCCCGTG 2700
HPF2013            ACGGTCGTTGTGGGATCTGTAAAAAACCCCATGTGGAGAGGTCCACAGAGATTGCCCGTG 2700
SPH2015            ACGGTCGTTGTGGGATCTGTAAAAAACCCCATGTGGAGAGGTCCACAGAGATTGCCCGTG 2700
Haiti2014          ACGGTCGTTGTGGGATCTGTAAAAAACCCCATGTGGAGAGGTCCACAGAGATTGCCCGTG 2700
PRVABC59           ACGGTCGTTGTGGGATCTGTAAAAAACCCCATGTGGAGAGGTCCACAGAGATTGCCCGTG 2700
BeH819015          ACGGTCGTTGTGGGATCTGTAAAAAACCCCATGTGGAGAGGTCCACAGAGATTGCCCGTG 2700
Z1106033           ACGGTCGTTGTGGGATCTGTAAAAAACCCCATGTGGAGAGGTCCACAGAGATTGCCCGTG 2700
BEH819966          ACGGTCGTTGTGGGATCTGTAAAAAACCCCATGTGGAGAGGTCCACAGAGATTGCCCGTG 2700
```

```
SSABr           ACGGTCGTTGTGGGATCTGTAAAAAACCCCATGTGGAGAGGTCCACAGAGATTGCCCGTG 2700
Beh815744       ACGGTCGTTGTGGGATCTGTAAAAAACCCCATGTGGAGAGGTCCACAGAGATTGCCCGTG 2700
BEH818995       ACGGTCGTTGTGGGATCTGTAAAAAACCCCATGTGGAGAGGTCCACAGAGATTGCCCGTG 2700
IbH30656        ACAGTTGTAGTGGGGTCTGTAAAAAACCCCATGTGGAGAGGTCCACGAAGATTGCCAGTG 2682
ArD128000       ACAGTTGTAGTGGGGTCTGTAAAAAACCCCATGTGGAGAGGTCCACAAAGATTGCCAGTG 2700
ArD7117         ACAGTTGTAGTGGGGTCTGTAAAAAACCCTATGTGGAGAGGTCCACAAAGATTGCCAGTG 2700
ARD_41519       ACAGTTGTAGTGGGGTCTGTAAAAAACCCTATGTGGAGAGGTCCACAGAGATTGCCAGTG 2700
ARD157995       ACAGTTGTTGTGGGATCTGTAAAAAACCCCATGTGGAGAGGTCCACAAAGATTGCCAGTG 2700
MR_766          ACAGTTGTTGTGGGATCTGTAAAAAACCCCATGTGGAGAGGTCCACAAAGATTGCCAGTG 2688
ARD158084       ACAGTTGTTGTGGGATCTGTAAAAAACCCCATGTGGAGAGGTCCACAAAGATTGCCAGTG 2700
ArB1362         ACAGTTGTTGTGGGATCTGTAAAAAACCCCATGTGGAGAGGTCCACAAAGATTGCCAGTG 2700
ARB13565        ACAGTTGTTGTAGGATCTGTAAAAAACCCCATGTGGAGAGGCCCACAAAGATTGCCAGTG 2700
ARB7701         ACAGTTGTTGTAGGATCTGTAAAAAACCCCATGTGGAGAGGCCCACAAAGATTGCCAGTG 2700
ARB15076        ACAGTTGTTGTAGGATCTGTAAAAAACCCCATGTGGAGAGGTCCACAAAGATTGCCAGTG 2682
                ** ** ** ** ** ************** **********  ** *  ** ***** ***

P6-740          CCTGTGAATGAGCTGCCCCACGGTTGGAAGGCCTGGGGGAAATCGTACTTTGTCAGGGCA 2760
CPC0740         CCTGTGAACGAGCTGCCCCACGGCTGGAAGGCTTGGGGGAAATCGTACTTCGTCAGAGCA 2760
YAP2007         CCTGTGAACGAGCTGCCCCACGGCTGGAAGGCTTGGGGGAAATCGTACTTCGTCAGAGCA 2760
FSS13025        CCTGTGAACGAGCTGCCCCATGGCTGGAAGGCTTGGGGGAAATCGTACTTCGTCAGGGCA 2760
PLCal_ZV        CCTGTGAACGAGCTGCCCCACGGCTGGAAGGCTTGGGGAAAATCGTACTTCGTCAGAGCA 2649
SV0127/14       CCTGTGAACGAGCTGCCCCACGGCTGGAAGGCTTGGGGGAAATCGTACTTCGTCAGAGCA 2760
8375            CCTGTGAACGAGCTGCCCCACGGCTGGAAGGCTTGGGGGAAATCGTACTTCGTCAGAGCA 2760
103344          CCTGTGAACGAGCTGCCCCACGGCTGGAAGGCTTGGGGGAAATCGTACTTCGTCAGAGCA 2760
BrasilZKV2015   CCTGTGAACGAGCTGCCCCACGGCTGGAAGGCTTGGGGGAAATCGTACTTCGTCAGAGCA 2760
GD01            CCTGTGAACGAGCTGCCCCACGGCTGGAAGGCTTGGGGGAAATCGTACTTCGTCAGAGCA 2760
Martinique      CCTGTGAACGAGCTGCCCCACGGCTGGAAGGCTTGGGGGAAATCGTACTTCGTCAGAGCA 2760
NatalRGN        CCTGTGAACGAGCTGCCCCACGGCTGGAAGGCTTGGGGGAAATCGTACTTCGTCAGAGCA 2760
HPF2013         CCTGTGAACGAGCTGCCCCACGGCTGGAAGGCTTGGGGGAAATCGTACTTCGTCAGAGCA 2760
SPH2015         CCTGTGAACGAGCTGCCCCACGGCTGGAAGGCTTGGGGGAAATCGCACTTCGTCAGAGCA 2760
Haiti2014       CCTGTGAACGAGCTGCCCCACGGCTGGAAGGCTTGGGGGAAATCGCACTTCGTCAGAGCA 2760
PRVABC59        CCTGTGAACGAGCTGCCCCACGGCTGGAAGGCTTGGGGGAAATCGTATTTCGTCAGAGCA 2760
BeH819015       CCTGTGAACGAGCTGCCCCACGGCTGGAAGGCTTGGGGGAAATCGTACTTCGTCAGAGCA 2760
Z1106033        CCTGTGAACGAGCTGCCCCACGGCTGGAAGGCTTGGGGGAAATCGTACTTCGTCAGAGCA 2760
BEH819966       CCTGTGAACGAGCTGCCCCACGGCTGGAAGGCTTGGGGGAAATCGTACTTCGTCAGAGCA 2760
SSABr           CCTGTGAACGAGCTGCCCCACGGCTGGAAGGCTTGGGGGAAATCGTACTTCGTCAGAGCA 2760
Beh815744       CCTGTGAACGAGCTGCCCCACGGCTGGAAGGCTTGGGGGAAATCGTACTTCGTCAGAGCA 2760
BEH818995       CCTGTGAACGAGCTGCCCCACGGCTGGAAGGCTTGGGGGAAATCGTACTTCGTCAGAGCA 2760
IbH30656        CCCGTAAATGAGCTGCCCCATGGCTGGAAAGCCTGGGGGAAATCGTACTTTGTTAGGGCG 2742
ArD128000       CCCGTGAATGAGCTGCCCCATGGCTGGAAAGCCTGGGGGAAATCGTACTTTGTCAGAGCG 2760
ArD7117         CCCGTGAATGAGCTGCCCCATGGCTGGAAAGCCTGGGGGAAATCGTACTTTGTCAGAGCG 2760
ARD_41519       CCTGTGAATGAGCTGCCCCATGGCTGGAAAGCCTGGGGGAAATCGTACTTTGTCAGAGCA 2760
ARD157995       CCTGTGAATGAGCTGCCCCATGGCTGGAAAGCCTGGGGGAAATCGTATTTTGTTAGGGCG 2760
MR_766          CCTGTGAATGAGCTGCCCCATGGCTGGAAAGCCTGGGGGAAATCGTATTTTGTTAGGGCG 2748
ARD158084       CCTGTGAATGGGCTGCCCCATGGCTGGAAAGCCTGGGGGAAATCGTATTTTGTTAGGGCG 2760
ArB1362         CCTGTAAATGAGCTGCCCCATGGCTGGAAAGCCTGGGGGAAATCATATTTTGTCAGAGCG 2760
ARB13565        CCTGTGAATGAGCTGCCCCATGGCTGGAAAGCCTGGGGGAAATCATATTTTGTCAGAGCG 2760
ARB7701         CCTGTGAATGAGCTGCCCCATGGCTGGAAAGCCTGGGGGAAATCATATTTTGTCAGAGCG 2760
ARB15076        CCTGTGAATGAGCTGCCCCATGGCTGGAAAGCCTGGGGGAAATCATATTTTGTCAGAGCG 2742
                ** ** ** * ********* ** ***** ** ***** *****  * ** ** ** **

P6-740          GCAAAGACCAACAACAGCTTTGTTGTGGATGGTGACACACTGAAGGAATGCCCGCTCAAA 2820
CPC0740         GCAAAGACAAATAACAGCTTTGTCGTGGATGGTGACACACTGAAGGAATGCCCACTCAAA 2820
YAP2007         GCAAAGACAAATAACAGCTTTGTCGTGGATGGTGACACACTGAAGGAATGCCCACTCAAA 2820
FSS13025        GCAAAGACAAATAACAGCTTTGTCGTGGATGGTGACACACTGAAGGAATGCCCACTCAAA 2820
PLCal_ZV        GCAAAGACAAATAACAGCTTTGTCGTGGATGGTGACACACTGAAGGAATGCCCACTCAAA 2709
SV0127/14       GCAAAGACAAATAACAGCTTTGTCGTGGATGGTGACACACTGAAGGAATGCCCACTCAAA 2820
8375            GCAAAGACAAATAACAGCTTTGTCGTGGATGGTGACACACTGAAGGAATGCCCACTCAAA 2820
103344          GCAAAGACAAATAACAGCTTTGTCGTGGATGGTGACACACTGAAGGAATGCCCACTCAAA 2820
BrasilZKV2015   GCAAAGACAAATAACAGCTTTGTCGTGGATGGTGACACACTGAAGGAATGCCCACTCAAA 2820
GD01            GCAAAGACAAATAACAGCTTTGTCGTGGATGGTGACACACTGAAGGAATGCCCACTCAAA 2820
Martinique      GCAAAGACAAATAACAGCTTTGTCGTGGATGGTGACACACTGAAGGAATGCCCACTCAAA 2820
NatalRGN        GCAAAGACAAATAACAGCTTTGTCGTGGATGGTGACACACTGAAGGAATGCCCACTCGAA 2820
HPF2013         GCAAAGACAAATAACAGCTTTGTCGTGGATGGTGACACACTGAAGGAATGCCCACTCAAA 2820
SPH2015         GCAAAGACAAATAACAGCTTTGTCGTGGATGGTGACACACTGAAGGAATGCCCACTCAAA 2820
Haiti2014       GCAAAGACAAATAACAGCTTTGTCGTGGATGGTGACACACTGAAGGAATGCCCACTCAAA 2820
PRVABC59        GCAAAGACAAATAACAGCTTTGTCGTGGATGGTGACACACTGAAGGAATGCCCACTCAAA 2820
BeH819015       GCAAAGACAAATAACAGCTTTGTCGTGGATGGTGACACACTGAAGGAATGCCCACTCAAA 2820
```

```
Z1106033           GCAAAGACAAATAACAGCTTTGTCGTGGATGGTGACACACTGAAGGAATGCCCACTCAAA 2820
BEH819966          GCAAAGACAAATAACAGCTTTGTCGTGGATGGTGACACACTGAAGGAATGCCCACTCAAA 2820
SSABr              GCAAAGACAAATAACAGCTTTGTCGTGGATGGTGACACACTGAAGGAATGCCCACTCAAA 2820
Beh815744          GCAAAGACAAATAACAGCTTTGTCGTGGATGGTGACACACTGAAGGAATGCCCACTCAAA 2820
BEH818995          GCAAAGACAAATAACAGCTTTGTCGTGGATGGTGACACACTGAAGGAATGCCCACTCAAA 2820
IbH30656           GCAAAGACCAACAACAGTTTTGTTGTCGACGGTGACACACTGAAGGAATGTCCGCTCAAA 2802
ArD128000          GCAAAGACCAACAACAGTTTTGTTGTCGACGGAGACACACTGAAGGAGTGTCCGCTCAAA 2820
ArD7117            GCAAAGACCAACAACAGTTTTGTTGTCGACGGTGACACACTGAAGGAGTGTCCGCTCAAA 2820
ARD_41519          GCAAAGACCAACAACAGTTTTGTTGTCGACGGTGACACACTGAAGGAGTGTCCGCTCAAA 2820
ARD157995          GCAAAGACCAACAACAGTTTTGTTGTCGACGGTGACACACTGAAGGAATGTCCGCTTAAG 2820
MR_766             GCAAAGACCAACAACAGTTTTGTTGTCGACGGTGACACACTGAAGGAATGTCCGCTTGAG 2808
ARD158084          GCAAAGACCAACAACAGTTTTGTTGTCGACGGTGACACACTGAAGGAATGTCCGCTTAAG 2820
ArB1362            GCAAAGACCAACAACAGTTTTGTTGTCGACGGTGACACACTGAAGGAATGTCCGCTCAAA 2820
ARB13565           GCAAAGACCAACAACAGTTTTGTTGTCGATGGTGACACACTGAAGGAATGTCCGCTCAAA 2820
ARB7701            GCAAAGACCAACAACAGTTTTGTTGTCGATGGTGACACACTGAAGGAATGTCCGCTCAAA 2820
ARB15076           GCAAAGACCAACAACAGGTTTGTTGTCGATGGTGACACACTGAAGGAATGTCCGCTCAAA 2802
                   ******** ** ***** ***** ** ** ** ************** ** ** **  * 

P6-740             CACAGAGCATGGAACAGCTTTCTTGTGGAGGATCACGGGTTCGGGGTATTTCACACTAGT 2880
CPC0740            CATAGAGCATGGAACAGCTTTCTTGTGGAGGATCATGGGTTTGGGGTATTTCACACTAGT 2880
YAP2007            CATAGAGCATGGAACAGCTTTCTTGTGGAGGATCATGGGTTCGGGGTATTTCACACTAGT 2880
FSS13025           CATAGAGCATGGAACAGCTTTCTTGTGGAGGATCATGGGTTCGGGGTATTTCACACTAGT 2880
PLCal_ZV           CATAGAGCATGGAACAGCTTTCTTGTGGAGGATCATGGGTTCGGGGTATTTCACACTAGT 2769
SV0127/14          CATAGAGCATGGAACAGCTTTCTTGTGGAGGATCATGGGTTCGGGGTATTTCACACTAGT 2880
8375               CATAGAGCATGGAACAGCTTTCTTGTGGAGGATCATGGGTTCGGGGTATTTCACACTAGT 2880
103344             CATAGAGCATGGAACAGCTTTCTTGTGGAGGATCATGGGTTCGGGGTATTTCACACTAGT 2880
BrasilZKV2015      CATAGAGCATGGAACAGCTTTCTTGTGGAGGATCATGGGTTCGGGGTATTTCACACTAGT 2880
GD01               CATAGAGCATGGAACAGCTTTCTTGTGGAGGATCATGGGTTCGGGGTATTTCACACTAGT 2880
Martinique         CATAGAGCATGGAACAGCTTTCTTGTGGAGGATCATGGGTTCGGGGTATTTCACACTAGT 2880
NatalRGN           CATAGAGCATGGAACAGCTTTCTTGTGGAGGATCATGGGTTCGGGGTATTTCACACTAGT 2880
HPF2013            CATAGAGCATGGAACAGCTTTCTTGTGGAGGATCATGGGTTCGGGGTATTTCACACTAGT 2880
SPH2015            CATAGAGCATGGAACAGCTTTCTTGTGGAGGATCATGGGTTCGGGGTATTTCACACTAGT 2880
Haiti2014          CATAGAGCATGGAACAGCTTTCTTGTGGAGGATCATGGGTTCGGGGTATTTCACACTAGT 2880
PRVABC59           CATAGAGCATGGAACAGCTTTCTTGTGGAGGATCATGGGTTCGGGGTATTTCACACTAGT 2880
BeH819015          CATAGAGCATGGAACAGCTTTCTTGTGGAGGATCATGGGTTCGGGGTATTTCACACTAGT 2880
Z1106033           CATAGAGCATGGAACAGCTTTCTTGTGGAGGATCATGGGTTCGGGGTATTTCACACTAGT 2880
BEH819966          CATAGAGCATGGAACAGCTTTCTTGTGGAGGATCATGGGTTCGGGGTATTTCACACTAGT 2880
SSABr              CATAGAGCATGGAACAGCTTTCTTGTGGAGGATCATGGGTTCGGGGTATTTCACACTAGT 2880
Beh815744          CATAGAGCATGGAACAGCTTTCTTGTGGAGGATCATGGGTTCGGGGTATTTCACACTAGT 2880
BEH818995          CATAGAGCATGGAACAGCTTTCTTGTGGAGGATCATGGGTTCGGGGTATTTCACACTAGT 2880
IbH30656           CATAGAGCATGGAATAGCTTCCTTGTGGAGGATCACGGGTTTGGGGTCTTCCACACCAGT 2862
ArD128000          CATAGAGCATGGAATAGCTTCCTTGTGGAGGATCACGGGTTTGGGATTTTCCACACCAGT 2880
ArD7117            CATAGAGCATGGAATAGCTTCCTTGTGGAGGATCACGGGTTTGGGATCTTCCACACCAGT 2880
ARD_41519          CATAGAGCATGGAATAGCTTCCTTGTGGAGGATCACGGGTTTGGGATCTTCCACACCAGT 2880
ARD157995          CACAGAGCATGGAATAGTTTTCTTGTGGAGGATCACGGGTTTGGAGTCTTCCACACCAGT 2880
MR_766             CACAGAGCATGGAATAGTTTTCTTGTGGAGGATCACGGGTTTGGAGTCTTCCACACCAGT 2868
ARD158084          CACAGAGCATGGAATAGTTTTCTTGTGGAGGATCACGGGTTTGGAGTCTTCCACACCAGT 2880
ArB1362            CACAGAGCATGGAACAGTTTCCTTGTGGAGGATCACGGGTTTGGGGTCTTCCACACTAGT 2880
ARB13565           CACAGAGCATGGAATAGTTTCCTTGTGGAGGATCATGGGTTTGGGGTCTTCCACACCAGT 2880
ARB7701            CACAGAGCATGGAATAGTTTCCTTGTGGAGGATCATGGGTTTGGGGTCTTCCACACCAGT 2880
ARB15076           CACAGAGCATGGAATAGTTTCCTTGTGGAGGATCATGGGTTTGGGGTCTTCCACACCAGT 2862
                   ** *********** ** ** ************** ***** **  * ** ***** *** 

P6-740             GTCTGGCTTAAAGTCAGAGAGGATTACTCATTAGAGTGTGATCCAGCCGTCATAGGAACA 2940
CPC0740            GTCTGGCTCAAGGTTAGAGAAGATTATTCATTAGAGTGTGATCCAGCCGTCATTGGAACA 2940
YAP2007            GTCTGGCTCAAGGTTAGAGAAGATTATTCATTAGAGTGTGATCCAGCCGTTATTGGAACA 2940
FSS13025           GTCTGGCTCAAGGTTAGAGAAGATTATTCATTAGAGTGTGATCCAGCCGTCATTGGAACA 2940
PLCal_ZV           GTCTGGCTCAAGGTTAGAGAGGATTACTCATTAGAGTGTGATCCAGCCGTCATTGGAACA 2829
SV0127/14          GTCTGGCTCAAGGTTAGAGAAGATTATTCACTAGAGTGTGATCCAGCCGTCATTGGAACA 2940
8375               GTCTGGCTCAAGGTTAGAGAAGATTATTCATTAGAGTGTGATCCAGCCGTTATTGGAACA 2940
103344             GTCTGGCTCAAGGTTAGAGAAGATTATTCATTAGAGTGTGATCCAGCCGTTATTGGAACA 2940
BrasilZKV2015      GTCTGGCTCAAGGTTAGAGAAGATTATTCATTAGAGTGTGATCCAGCCGTTATTGGAACA 2940
GD01               GTCTGGCTCAAGGTTAGAGAAGACTATTCATTAGAGTGTGATCCAGCCGTTATTGGAACA 2940
Martinique         GTCTGGCTCAAGGTTAGAGAAGATTATTCATTAGAGTGTGATCCAGCCGTTATTGGAACA 2940
NatalRGN           GTCTGGCTCAAGGTTAGAGAAGATTATTCATTAGAGTGTGATCCAGCCGTTATTGGAACA 2940
HPF2013            GTCTGGCTCAAGGTTAGAGAAGATTATTCATTAGAGTGTGATCCAGCCGTTATTGGAACA 2940
SPH2015            GTCTGGCTCAAGGTTAGAGAAGATTATTCATTAGAGTGTGATCCAGCCGTTATTGGAACA 2940
Haiti2014          GTCTGGCTCAAGGTTAGAGAAGATTATTCATTAGAGTGTGATCCAGCCGTTATTGGAACA 2940
```

```
PRVABC59            GTCTGGCTCAAGGTTAGAGAAGATTATTCATTAGAGTGTGATCCAGCCGTTATTGGAACA 2940
BeH819015           GTCTGGCTCAAGGTTAGAGAAGATTATTCATTAGAGTGTGATCCAGCCGTTATTGGAACA 2940
Z1106033            GTCTGGCTCAAGGTTAGAGAAGATTATTCATTAGAGTGTGATCCAGCCGTTATTGGAACA 2940
BEH819966           GTCTGGCTCAAGGTTAGAGAAGATTATTCATTAGAGTGTGATCCAGCCGTTATTGGAACA 2940
SSABr               GTCTGGCTCAAGGTTAGAGAAGATTATTCATTAGAGTGTGATCCAGCCGTTATTGGAACA 2940
Beh815744           GTCTGGCTCAAGGTTAGAGAAGATTATTCATTAGAGTGTGATCCAGCCGTTATTGGAACA 2940
BEH818995           GTCTGGCTCAAGGTTAGAGAAGATTATTCATTAGAGTGTGATCCAGCCGTTATTGGAACA 2940
IbH30656            GTTTGGCTGAAGGTCAGAGAGGACTATTCATTAGAGTGTGACCCAGCCGTCATAGGAACA 2922
ArD128000           GTTTGGCTGAAGGTCAGAGAGGACTACTCATTAGAGTGTGACCCAGCCGTCATAGGAACA 2940
ArD7117             GTTTGGCTGAAGGTCAGAGAGGACTACTCATTAGAGTGTGACCCAGCCGTCATAGGAACA 2940
ARD_41519           GTTTGGCTGAAGGTCAGAGAGGACTACTCACTAGAGTGTGACCCAGCCGTCATAGGAACA 2940
ARD157995           GTTTGGCTTAAGGTCAGAGAAGATTACTCATTAGAATGTGACCCAGCCGTCATAGGAACA 2940
MR_766              GTCTGGCTTAAGGTCAGAGAAGATTACTCATTAGAATGTGACCCAGCCGTCATAGGAACA 2928
ARD158084           GTTTGGCTTAAGGTCAGAGAAGATTACTCATTAGAATGTGACCCAGCCGTCATAGGAACA 2940
ArB1362             GTTTGGCTTAAGGTTAGAGAAGATTACTCACTAGAGTGTGACCCAGCCGTCATAGGAACA 2940
ARB13565            GTTTGGCTCAAGGTTAGAGAAGACTACTCACTAGAATGTGACCCAGCCGTCATAGGAACA 2940
ARB7701             GTTTGGCTCAAGGTTAGAGAAGACTACTCACTAGAATGTGACCCAGCCGTCATAGGAACA 2940
ARB15076            GTTTGGCTTAAGGTTAGAGAAGATTACTCACTAGAATGTGACCCAGCCGTCATAGGAACA 2922
                    ** ***** ** ** ***** ** ** *** **** ***** ******** ** ******

P6-740              GCTGCTAAGGGAAAGGAGGCCGTGCACAGTGATCTAGGCTACTGGATTGAGAGTGAAAAG 3000
CPC0740             GCTGCTAAGGGAAAGGAGGCTGTGCACAGCGATCTAGGCTACTGGATTGAGAGTGAGAAG 3000
YAP2007             GCTGCTAAGGGAAAGGAGGCTGTGCACAGTGATCTAGGCTACTGGATTGAGAGTGAGAAG 3000
FSS13025            GCCGCTAAGGGAAAGGAGGCTGTGCACAGTGATCTAGGCTACTGGATTGAGAGTGAGAAG 3000
PLCal_ZV            GCTGTTAAGGGAAAGGAGGCTGTACACAGTGATCTAGGCTACTGGATTGAGAGTGAGAAG 2889
SV0127/14           GCTGTTAAGGGAAAGGAGGCTGTACACAGTGATCTAGGCTACTGGATTGAGAGTGAGAAG 3000
8375                GCTGTTAAGGGAAAGGAGGCTGTACACAGTGATCTAGGCTACTGGATTGAGAGTGAGAAG 3000
103344              GCTGTTAAGGGAAAGGAGGCTGTACACAGTGATCTAGGCTACTGGATTGAGAGTGAGAAG 3000
BrasilZKV2015       GCTGTTAAGGGAAAGGAGGCTGTACACAGTGATCTAGGCTACTGGATTGAGAGTGAGAAG 3000
GD01                GCTGTTAAGGGAAAGGAGGCTGTACACAGTGATCTAGGCTACTGGATTGAGAGTGAGAAG 3000
Martinique          GCTGTTAAGGGAAAGGAGGCTGTACACAGTGATCTAGGCTACTGGATTGAGAGTGAGAAG 3000
NatalRGN            GCTGTTAAGGGGAAGGAGGCTGTACACAGTGATCTAGGCTACTGGATTGAGAGTGAGAAG 3000
HPF2013             GCTGTTAAGGGAAAGGAGGCTGTACACAGTGATCTAGGCTACTGGATTGAGAGTGAGAAG 3000
SPH2015             GCTGTTAAGGGAAAGGAGGCTGTACACAGTGATCTAGGCTACTGGATTGAGAGTGAGAAG 3000
Haiti2014           GCTGTTAAGGGAAAGGAGGCTGTACACAGTGATCTAGGCTACTGGATTGAGAGTGAGAAG 3000
PRVABC59            GCTGTTAAGGGAAAGGAGGCTGTACACAGTGATCTAGGCTACTGGATTGAGAGTGAGAAG 3000
BeH819015           GCTGTTAAGGGAAAGGAGGCTGTACACAGTGATCTAGGCTACTGGATTGAGAGTGAGAAG 3000
Z1106033            GCTGTTAAGGGAAAGGAGGCTGTACACAGTGATCTAGGCTACTGGATTGAGAGTGAGAAG 3000
BEH819966           GCTGTTAAGGGAAAGGAGGCTGTACACAGTGATCTAGGCTACTGGATTGAGAGTGAGAAG 3000
SSABr               GCTGTTAAGGGAAAGGAGGCTGTACACAGTGATCTAGGCTACTGGATTGAGAGTGAGAAG 3000
Beh815744           GCTGTTAAGGGAAAGGAGGCTGTACACAGTGATCTAGGCTACTGGATTGAGAGTGAGAAG 3000
BEH818995           GCTGTTAAGGGAAAGGAGGCTGTACACAGTGATCTAGGCTACTGGATTGAGAGTGAGAAG 3000
IbH30656            GCTGTCAAGGGAAAGGAGGCTGCACACAGTGATCTAGGCTATTGGATTGAGAGTGAAAAG 2982
ArD128000           GCTGTCAAGGGAAAGGAAGCTGCACACAGTGATCTATGCTATTGGATTGAGAGTGAAAAG 3000
ArD7117             GCTGTCAAGGGAAAGGAAGCTGCACACAGTGATCTAGGCTATTGGATTGAGAGTGAAAAG 3000
ARD_41519           GCTGTCAAGGGAAAGGAAGCTGCACACAGTGATCTAGGCTATTGGATTGAGAGTGAAAAG 3000
ARD157995           GCTGTTAAGGGAAGGGAGGCCGCGCACAGTGATCTGGGCTATTGGATTGAAAGTGAAAAG 3000
MR_766              GCTGTTAAGGGAAGGGAGGCCGCGCACAGTGATCTGGGCTATTGGATTGAAAGTGAAAAG 2988
ARD158084           GCTGTTAAGGGAAGGGAGGCCGCGCACAGTGATCTGGGCTATTGGATTGAAAGTGAAAAG 3000
ArB1362             GCTGTTAAGGGAAAGGAGGCCGCGCACAGTGATCTAGGCTACTGGATTGAAAGTGAAAAG 3000
ARB13565            GCTGTTAAGGGAAAGGAGGCCGCCCACAGTGACCTGGGCTACTGGATTGAAAGTGAAAAG 3000
ARB7701             GCTGTTAAGGGAAAGGAGGCCGCCCACAGTGACCTGGGCTACTGGATTGAAAGTGAAAAG 3000
ARB15076            GCTGTTAAGGGAAAGGAGGCCGCGCACAGTGACCTAGGCTACTGGATTGAAAGTGAAAAG 2982
                    ** *  ***** * *** ** *  ***** ** **  **** ******** ***** ***

P6-740              AACGACACATGGAGGCTGAAGAGGGCTCACCTGATCGAGATGAAAACATGTGAATGGCCA 3060
CPC0740             AACGACACATGGAGGCTGAAGAGGGCCCACCTGATCGAGATGAAAACATGTGAATGGCCA 3060
YAP2007             AATGACACATGGAGGCTGAAGAGGGCCCACCTGATCGAGATGAAAACATGTGAATGGCCA 3060
FSS13025            AACGACACATGGAGGCTGAAGAGGGCCCACCTGATCGAGATGAAAACATGTGAATGGCCA 3060
PLCal_ZV            AACGACACATGGAGGCTGAAGAGGGCCCACCTGATCGAGATGAAAACATGTGAATGGCCA 2949
SV0127/14           AACGACACATGGAGGCTGAGGAGGGCCCACCTGATCGAGATGAAAACATGTGAATGGCCA 3060
8375                AATGACACATGGAGGCTGAAGAGGGCCCATCTGATCGAGATGAAAACATGTGAATGGCCA 3060
103344              AATGACACATGGAGGCTGAAGAGGGCCCATCTGATCGAGATGAAAACATGTGAATGGCCA 3060
BrasilZKV2015       AATGACACATGGAGGCTGAAGAGGGCCCATCTGATCGAGATGAAAACATGTGAATGGCCA 3060
GD01                AATGACACATGGAGGCTGAAGAGGGCCCATCTGATCGAGATGAAAACATGTGAATGGCCA 3060
Martinique          AATGACACATGGAGGCTGAAGAGGGCCCATCTGATCGAGATGAAAACATGTGAATGGCCA 3060
NatalRGN            AATGACACATGGAGGCTGAAGAGGGCCCATCTAATCGAGATGAAAACATGTGAATGGCCA 3060
HPF2013             AATGACACATGGAGGCTGAAGAGGGCCCATCTGATCGAGATGAAAACATGTGAATGGCCA 3060
```

```
SPH2015          AATGACACATGGAGGCTGAAGAGGGCCCATCTGATCGAGATGAAAACATGTGAATGGCCA 3060
Haiti2014        AATGACACATGGAGGCTGAAGAGGGCCCATCTGATCGAGATGAAAACATGTGAATGGCCA 3060
PRVABC59         AATGACACATGGAGGCTGAAGAGGGCCCATCTGATCGAGATGAAAACATGTGAATGGCCA 3060
BeH819015        AATGACACATGGAGGCTGAAGAGGGCCCATCTGATCGAGATGAAAACATGTGAATGGCCA 3060
Z1106033         AATGACACATGGAGGCTGAAGAGGGCCCATCTGATCGAGATGAAAACATGTGAATGGCCA 3060
BEH819966        AATGACACATGGAGGCTGAAGAGGGCCCATCTGATCGAGATGAAAACATGTGAATGGCCA 3060
SSABr            AATGACACATGGAGGCTGAAGAGGGCCCATCTGATCGAGATGAAAACATGTGAATGGCCA 3060
Beh815744        AATGACACATGGAGGCTGAAGAGGGCCCATCTGATCGAGATGAAAACATGTGAATGGCCA 3060
BEH818995        AATGACACATGGAGGCTGAAGAGGGCCCATCTGATCGAGATGAAAACATGTGAATGGCCA 3060
IbH30656         AATGACACATGGAGGCTGAAGAGGGCTCATCTGATTGAGATGAAGACATGTGAGTGGCCA 3042
ArD128000        AATGACACATGGAGGCTGAGGAGGGCCCACCTGATTGAGATGAAAACATGTGAATGGCCA 3060
ArD7117          AATGACACATGGAGGCTGAGGAGGGCTCATCTGATTGAGATGAAAACATGTGAGTGGCCA 3060
ARD_41519        AATGACACATGGAGGCTGAGGAGGGCTCATCTGATTGAGATGAAAACATGTGAGTGGCCA 3060
ARD157995        AATGACACATGGAGGCTGAAGAGGGCCCACCTGATTGAGATGAAAACATGTGAATGGCCA 3060
MR_766           AATGACACATGGAGGCTGAAGAGGGCCCACCTGATTGAGATGAAAACATGTGAATGGCCA 3048
ARD158084        AATGACACATGGAGGCTGAAGAGGGCCCACCTGATTGAGATGAAAACATGTGAATGGCCA 3060
ArB1362          AATGACACATGGAGGTTGAAGAGGGCCCACCTGATTGAGATGAAAACATGTGAGTGGCCA 3060
ARB13565         AATGACACATGGAGGCTGAAGAGGGCCCATCTGATTGAGATGAAAACATGTGAGTGGCCA 3060
ARB7701          AATGACACATGGAGGCTGAAGAGGGCCCATCTGATTGAGATGAAAACATGTGAGTGGCCA 3060
ARB15076         AATGACACATGGAGGCTGAAGAGGGCCCACCTGATTGAGATGAAAACATGTGAGTGGCCA 3042
                 ** ************ *** ****** ** ** ** ******** ******** ******

P6-740           AAGTCCCACACACTGTGGACAGATGGAATAGAAGAAAGTGATCTGATCATACCTAAGTCT 3120
CPC0740          AAGTCCCACACATTGTGGACAGATGGAGTAGAAGAAAGTGATCTGATCATACCCAAGTCT 3120
YAP2007          AAGTCCCACACATTGTGGACAGATGGAATAGAAGAAAGTGATCTGATCATACCCAAGTCT 3120
FSS13025         AAGTCCCACACATTGTGGACAGATGGAATAGAAGAAAGTGATCTGATCATACCCAAGTCT 3120
PLCal_ZV         AAGTCCCACACATTGTGGACAGATGGAATAGAAGAAAGTGATCTGATCATACCCAAGTCT 3009
SV0127/14        AAGTCCCACACATTGTGGACAGATGGAATAGAAGAGAGTGATCTGATCATACCCAAGTCT 3120
8375             AAGTCCCACACATTGTGGACAGATGGAATAGAAGAGAGTGATCTGATCATACCCAAGTCT 3120
103344           AAGTCCCACACATTGTGGACAGATGGAATAGAAGAGAGTGATCTGATCATACCCAAGTCT 3120
BrasilZKV2015    AAGTCCCACACATTGTGGACAGATGGAATAGAAGAGAGTGATCTGATCATACCCAAGTCT 3120
GD01             AAGTCCCACACATTGTGGACAGATGGAATAGAAGAGAGTGATCTGATCATACCCAAGTCT 3120
Martinique       AAATCCCACACATTGTGGACAGATGGAATAGAAGAGAGTGATCTGATCATACCCAAGTCT 3120
NatalRGN         AAGTCCCACACATTGTGGGCAGATGGAATAGAAGAGAGTGATCTGATCATTCCCAAGTCT 3120
HPF2013          AAGTCCCACACATTGTGGACAGATGGAATAGAAGAGAGTGATCTGATCATACCCAAGTCT 3120
SPH2015          AAGTCCCACACATTGTGGACAGATGGAATAGAAGAGAGTGATCTGATCATACCCAAGTCT 3120
Haiti2014        AAGTCCCACACATTGTGGACAGATGGAATAGAAGAGAGTGATCTGATCATACCCAAGTCT 3120
PRVABC59         AAGTCCCACACATTGTGGACAGATGGAATAGAAGAGAGTGATCTGATCATACCCAAGTCT 3120
BeH819015        AAGTCCCACACATTGTGGACAGATGGAATAGAAGAGAGTGATCTGATCATACCCAAGTCT 3120
Z1106033         AAGTCCCACACATTGTGGACAGATGGAATAGAAGAGAGTGATCTGATCATACCCAAGTCT 3120
BEH819966        AAGTCCCACACATTGTGGACAGATGGAATAGAAGAGAGTGATCTGATCATACCCAAGTCT 3120
SSABr            AAGTCCCACACATTGTGGACAGATGGAATAGAAGAGAGTGATCTGATCATACCCAAGTCT 3120
Beh815744        AAGTCCCACACATTGTGGACAGATGGAATAGAAGAGAGTGATCTGATCATACCCAAGTCT 3120
BEH818995        AAGTCCCACACATTGTGGACAGATGGAATAGAAGAGAGTGATCTGATCATACCCAAGTCT 3120
IbH30656         AAGTCTCACACACTGTGGACAGATGGAGTGGAAGAAAGTGATCTGATCATACCCAAGTCC 3102
ArD128000        AAGTCTCACACATTGTGGACAGATGGAGTAGAAGAAAGTGATCTTATCATACCCAAGTCT 3120
ArD7117          AAGTCTCACACACTGTGGACAGATGGAGTGGAAGAAAGTGATCTGATCATACCCAAGTCC 3120
ARD_41519        AAGTCTCACACACTGTGGACAGATGGAGTGGAAGAAAGTGATCTGATCATACCCAAGTCC 3120
ARD157995        AAGTCTCACACATTGTGGACAGATGGAGTAGAAGAAAGTGATCTTATCATACCCAAGTCT 3120
MR_766           AAGTCTCACACATTGTGGACAGATGGAGTAGAAGAAAGTGATCTTATCATACCCAAGTCT 3108
ARD158084        AAGTCTCACACATTGTGGACAGATGGAGTAGAAGAAAGTGATCTTATCATACCCAAGTCT 3120
ArB1362          AAGTCTCACACACTGTGGACAGATGGAGTAGAAGAAAGTGATCTGATCATACCCAAGTCT 3120
ARB13565         AAGTCTCACACTCTGTGGACAGATGGAGTAGAAGAAAGTGATCTGATCATACCTAAATCT 3120
ARB7701          AAGTCTCACACTCTGTGGACAGATGGAGTAGAAGAAAGTGATCTGATCATACCTAAATCT 3120
ARB15076         AAGTCTCACACTCTGTGGACAGATGGAGTAGAAGAAAGTGATCTGATCATACCTAAATCT 3102
                 ** ** *****  ***** ******** * ***** ******** ***** ** ** **

P6-740           TTAGCTGGGCCACTCAGCCACCACAACACCAGAGAGGGCTACAGGACTCAAGTGAAAGGG 3180
CPC0740          TTAGCTGGGCCACTCAGCCATCACAACACCAGAGAGGGCTACAGGACTCAAATGAAAGGG 3180
YAP2007          TTAGCTGGGCCACTCAGCCATCACAACACCAGAGAGGGCTACAGGACTCAAATGAAAGGG 3180
FSS13025         TTAGCTGGGCCACTCAGCCATCACAACACCAGAGAGGGCTACAGGACCCAAATGAAAGGG 3180
PLCal_ZV         TTAGCTGGGCCACTCAGCCATCACAACACCAGAGAGGGTTACAGGACCCAAATGAAAGGG 3069
SV0127/14        TTAGCTGGGCCACTCAGCCATCACAACACCAGAGAGGGCTACAGGACCCAAATGAAAGGG 3180
8375             TTAGCTGGGCCACTCAGCCATCACAATACCAGAGAGGGCTACAGGACCCAAATGAAAGGG 3180
103344           TTAGCTGGGCCACTCAGCCATCACAATACCAGAGAGGGCTACAGGACCCAAATGAAAGGG 3180
BrasilZKV2015    TTAGCTGGGCCACTCAGCCATCACAATACCAGAGAGGGCTACAGGACCCAAATGAAAGGG 3180
GD01             TTAGCTGGGCCACTCAGCCATCACAATACCAGAGAGGGCTACAGGACCCAAATGAAAGGG 3180
Martinique       TTAGCTGGGCCACTCAGCCATCACAATACCAGAGAGGGCTACAGGACCCAAATGAAAGGG 3180
```

```
NatalRGN          TTAGCTGGGCCACTCAGCCATCACAATACCAGAGAGGGCTACAGGACCCAAATGAAAGGG 3180
HPF2013           TTAGCTGGGCCACTCAGCCATCACAATACCAGAGAGGGCTACAGGACCCAAATGAAAGGG 3180
SPH2015           TTAGCTGGGCCACTCAGCCATCACAATACCAGAGAGGGCTACAGGACCCAAATGAAAGGG 3180
Haiti2014         TTAGCTGGGCCACTCAGCCATCACAATACCAGAGAGGGCTACAGGACCCAAATGAAAGGG 3180
PRVABC59          TTAGCTGGGCCACTCAGCCATCACAATACCAGAGAGGGCTACAGGACCCAAATGAAAGGG 3180
BeH819015         TTAGCTGGGCCACTCAGCCATCACAATACCAGAGAGGGCTACAGGACCCAAATGAAAGGG 3180
Z1106033          TTAGCTGGGCCACTCAGCCATCACAATACCAGAGAGGGCTACAGGACCCAAATGAAAGGG 3180
BEH819966         TTAGCTGGGCCACTCAGCCATCACAATACCAGAGAGGGCTACAGGACCCAAATGAAAGGG 3180
SSABr             TTAGCTGGGCCACTCAGCCATCACAATACCAGAGAGGGCTACAGGACCCAAATGAAAGGG 3180
Beh815744         TTAGCTGGGCCACTCAGCCATCACAATACCAGAGAGGGCTACAGGACCCAAATGAAAGGG 3180
BEH818995         TTAGCTGGGCCACTCAGCCATCACAATACCAGAGAGGGCTACAGGACCCAAATGAAAGGG 3180
IbH30656          TTAGCTGGTCCACTCAGCCACCACAACACCAGAGAGGGTTATAGAACTCAAGTGAAAGGG 3162
ArD128000         TTAGCTGGTCCACTCAGCCACCACAACACCAGAGAGGGTTACAGAACCCAAGTGAAAGGG 3180
ArD7117           TTAGCTGGTCCACTCAGCCACCACAACACCAGAGAGGGTTACAGAACTCAAGTGAAAGGG 3180
ARD_41519         TTAGCTGGTCCACTCAGCCACCACAACACCAGAGAGGGTTACAGAACTCAATTGAAAGGG 3180
ARD157995         TTAGCTGGTCCACTCAGCCACCACAACACCAGAGAGGGTTACAGAACCCAAGTGAAAGGG 3180
MR_766            TTAGCTGGTCCACTCAGCCACCACAACACCAGAGAGGGTTACAGAACCCAAGTGAAAGGG 3168
ARD158084         TTAGCTGGTCCACTCAGCCACCACAACACCAGAGAGGGTTACAGAACCCAAGTGAAAGGG 3180
ArB1362           TTAGCTGGTCCACTCAGCCACCACAACACCAGAGAGGGTTACAGAACTCAGGCAAAAGGG 3180
ARB13565          TTAGCTGGTCCACTCAGCCACCACAACACTAGAGAGGGTTACAGAACTCAAGCAAAAGGG 3180
ARB7701           TTAGCTGGTCCACTCAGCCACCACAACACTAGAGAGGGTTACAGAACTCAAGCAAAAGGG 3180
ARB15076          TTAGCTGGTCCACTCAGCCACCACAACACTAGAGAGGGTTACAGAACTCAAGCAAAAGGG 3162
                  ******** *********** ***** ** ******** ** ** ** **    ******

P6-740            CCGTGGCATAGTGAAGAGCTTGAAATCCGGTTTGAGGAATGTCCAGGCACCAAGGTCCAC 3240
CPC0740           CCATGGCACAGTGAAGAGCTTGAAATTCGGTTTGAGGAATGCCCAGGCACTAAGGTCCAC 3240
YAP2007           CCATGGCACAGTGAAGAGCTTGAAATTCGGTTTGAGGAATGCCCAGGCACTAAGGTCCAC 3240
FSS13025          CCATGGCATAGTGAAGAGCTTGAAATTCGGTTTGAGGAATGCCCAGGCACTAAGGTCCAC 3240
PLCal_ZV          CCATGGCACAGTGAAGAGCTTGAAATTCGGTTTGAGGAATGCCCAGGCACTAAGGTCCAC 3129
SV0127/14         CCATGGCACAGTGAAGAGCTTGAAATTCGGTTTGAGGAATGCCCAGGCACTAAGGTCCAC 3240
8375              CCATGGCACAGTGAAGAGCTTGAAATTCGGTTTGAGGAATGCCCAGGCACTAAGGTCCAC 3240
103344            CCATGGCACAGTGAAGAGCTTGAAATTCGGTTTGAGGAATGCCCAGGCACTAAGGTCCAC 3240
BrasilZKV2015     CCATGGCACAGTGAAGAGCTTGAAATTCGGTTTGAGGAATGCCCAGGCACTAAGGTCCAC 3240
GD01              CCATGGCACAGTGAGGAGCTTGAAATTCGGTTTGAGGAATGCCCAGGCACTAAGGTCCAC 3240
Martinique        CCATGGCACAGTGAAGAGCTTGAAATTCGGTTTGAGGAATGCCCAGGCACTAAGGTCCAC 3240
NatalRGN          CCATGGCACAGTGAAGAGCTTGAAATTCGGTTTGAGGAATGCCCGGGCACTAAGGTCCAC 3240
HPF2013           CCATGGCACAGTGAAGAGCTTGAAATTCGGTTTGAGGAATGCCCAGGCACTAAGGTCCAC 3240
SPH2015           CCATGGCACAGTGAAGAGCTTGAAATTCGGTTTGAGGAATGCCCAGGCACTAAGGTCCAC 3240
Haiti2014         CCATGGCACAGTGAAGAGCTTGAAATTCGGTTTGAGGAATGCCCAGGCACTAAGGTCCAC 3240
PRVABC59          CCATGGCACAGTGAAGAGCTTGAAATTCGGTTTGAGGAATGCCCAGGCACTAAGGTCCAC 3240
BeH819015         CCATGGCACAGTGAAGAGCTTGAAATTCGGTTTGAGGAATGCCCAGGCACTAAGGTCCAC 3240
Z1106033          CCATGGCACAGTGAAGAGCTTGAAATTCGGTTTGAGGAATGCCCAGGCACTAAGGTCCAC 3240
BEH819966         CCATGGCACAGTGAAGAGCTTGAAATTCGGTTTGAGGAATGCCCAGGCACTAAGGTCCAC 3240
SSABr             CCATGGCACAGTGAAGAGCTTGAAATTCGGTTTGAGGAATGCCCAGGCACTAAGGTCCAC 3240
Beh815744         CCATGGCACAGTGAAGAGCTTGAAATTCGGTTTGAGGAATGCCCAGGCACTAAGGTCCAC 3240
BEH818995         CCATGGCACAGTGAAGAGCTTGAAATTCGGTTTGAGGAATGCCCAGGCACTAAGGTCCAC 3240
IbH30656          CCATGGCATAGTGAAGAGCTCGAAATCCGGTTTGAGGAATGCCCAGGCACCAAGGTTCAT 3222
ArD128000         CCATGGCACAGTGAAGAGCTTGAAATCCGGTTTGAGGAATGTCCAGGCACCAAGGTTTAC 3240
ArD7117           CCATGGCATAGTGAAGAGCTCGAAATCCGGTTTGAGGAGTGCCCAGGAACCAAGGTTCAC 3240
ARD_41519         CCATGGCACAGTGAAGAGCTCGAAATCCGGTTTGAGGAGTGCCCAGGAACCAAGGTTCAC 3240
ARD157995         CCATGGCACAGTGAAGAGCTTGAAATCCGGTTTGAGGAATGTCCAGGCACCAAGGTTCAC 3240
MR_766            CCATGGCACAGTGAAGAGCTTGAAATCCGGTTTGAGGAATGTCCAGGCACCAAGGTTTAC 3228
ARD158084         CCATGGCACAGTGAAGAGCTTGAAATCCGGTTTGAGGAATGTCCAGGCACCAAGGTTCAC 3240
ArB1362           CCATGGCATAGTGAAGAGCTTGAAATCCGGTTTGAGGAATGTCCAGGCACCAAGGTTCAC 3240
ARB13565          CCATGGCATAGTGAAGAGCTTGAAATCCGGTTTGAGGAATGTCCAGGCACCAAGGTTCAC 3240
ARB7701           CCATGGCATAGTGAAGAGCTTGAAATCCGGTTTGAGGAATGTCCAGGCACCAAGGTTCAC 3240
ARB15076          CCATGGCATAGTGAAGAGCTTGAAATCCGGTTTGAGGAATGTCCAGGCACCAAGGTTCAT 3222
                  ** ***** ***** ***** ***** *********** ** ** ** ** *****  *

P6-740            GTGGAGGAAACATGTGGAACGAGAGGACCGTCCCTGAGATCAACCACTGCAAGCGGAAGG 3300
CPC0740           GTGGAGGAAACATGTGGGACAAGAGGACCATCCCTGAGATCAACCACTGCAAGCGGAAGG 3300
YAP2007           GTGGAGGAAACATGTGGAACAAGAGGACCATCTCTGAGATCAACCACTGCAAGCGGAAGG 3300
FSS13025          GTGGAGGAAACATGTGGAACAAGAGGACCATCTCTGAGATCAACCACTGCAAGCGGAAGG 3300
PLCal_ZV          GTGGAGGAAACATGTGGAACAAGAGGACCATCTCTGAGATCAACCACTGCAAGCGGAAGG 3189
SV0127/14         GTGGAGGAAACATGTGGAACAAGAGGACCATCTCTGAGATCAACCACTGCAAGCGGAAGG 3300
8375              GTGGAGGAAACATGTGGAACAAGAGGACCATCTCTGAGATCAACCACTGCAAGCGGAAGG 3300
103344            GTGGAGGAAACATGTGGAACAAGAGGACCATCTCTGAGATCAACCACTGCAAGCGGAAGG 3300
BrasilZKV2015     GTGGAGGAAACATGTGGAACAAGAGGACCATCTCTGAGATCAACCACTGCAAGCGGAAGG 3300
```

```
GD01                GTGGAGGAAACATGTGGAACAAGAGGACCATCTCTGAGATCAACCACTGCAAGCGGAAGG 3300
Martinique          GTGGAGGAAACATGTGGAACAAGAGGACCATCTCTGAGATCAACCACTGCAAGCGGAAGG 3300
NatalRGN            GTGGAGGAAACATGTGGAACAAGAGGACCATCTCTGAGATCAACCACTGCAAGCGGAAGG 3300
HPF2013             GTGGAGGAAACATGTGGAACAAGAGGACCATCTCTGAGATCAACCACTGCAAGCGGAAGG 3300
SPH2015             GTGGAGGAAACATGTGGAACAAGAGGACCATCTCTGAGATCAACCACTGCAAGCGGAAGG 3300
Haiti2014           GTGGAGGAAACATGTGGAACAAGAGGACCATCTCTGAGATCAACCACTGCAAGCGGAAGG 3300
PRVABC59            GTGGAGGAAACATGTGGAACAAGAGGACCATCTCTGAGATCAACCACTGCAAGCGGAAGG 3300
BeH819015           GTGGAGGAAACATGTGGAACAAGAGGACCATCTCTGAGATCAACCACTGCAAGCGGAAGG 3300
Z1106033            GTGGAGGAAACATGTGGAACGAGAGGACCATCTCTGAGATCAACCACTGCAAGCGGAAGG 3300
BEH819966           GTGGAGGAAACATGTGGAACAAGAGGACCATCTCTGAGATCAACCACTGCAAGCGGAAGG 3300
SSABr               GTGGAGGAAACATGTGGAACAAGAGGACCATCTCTGAGATCAACCACTGCAAGCGGAAGG 3300
Beh815744           GTGGAGGAAACATGTGGAACAAGAGGACCATCTCTGAGATCAACCACTGCAAGCGGAAGG 3300
BEH818995           GTGGAGGAAACATGTGGAACAAGAGGACCATCTCTGAGATCAACCACTGCAAGCGGAAGG 3300
IbH30656            GTGGAGGAGACATGCGGAACTAGAGGACCATCTTTAAGATCAACCACTGCAAGTGGAAGG 3282
ArD128000           GTGGAGGAGACATGCGGAACTAGAGGACCATCTCTGAGATCAACTACTGCAAGTGGAAGG 3300
ArD7117             GTGGAGGAGACATGCGGAACTAGAGGACCATCTCTGAGATCAACTACTGCAAGTGGAAGG 3300
ARD_41519           GTGGAGGAGACATGCGGAACTAGAGGACCATCTCTGAGATCGACTACTGCAAGTGGAAGG 3300
ARD157995           GTGGAGGAGACATGCGGAACTAGAGGACCATCTCTGAGATCAACTACTGCAAGTGGAAGG 3300
MR_766              GTGGAGGAGACATGCGGAACTAGAGGACCATCTCTGAGATCAACTACTGCAAGTGGAAGG 3288
ARD158084           GTGGAGGAGACATGCGGAACTAGAGGACCATCTCTGAGATCAACTACTGCAAGTGGAAGG 3300
ArB1362             GTGGAGGAGACATGCGGAACTAGAGGACCATCTCTGAGATCAACCACTGCAAGTGGAAGG 3300
ARB13565            GTGGAGGAGACATGCGGAACTAGAGGACCATCTCTGAGATCAACCACTGCAAGTGGAAGG 3300
ARB7701             GTGGAGGAGACATGCGGAACTAGAGGACCATCTCTGAGATCAACCACTGCAAGTGGAAGG 3300
ARB15076            GTGGAGGAGACATGCGGAACTAGAGGACCATCTCTGAGATCAACCACTGCAAGTGGAAGG 3282
                    ******** ***** ** ** ******** **  * ***** ** ******** ******

P6-740              GTGATCGAGGAATGGTGCTGCAGGGAATGCACAATGCCCCCATTGTCGTTCCGGGCAAAA 3360
CPC0740             GTGATCGAGGAATGGTGCTGCAGGGAATGCACAATGCCCCCACTGTCGTTCCGAGCTAAA 3360
YAP2007             GTGATCGAGGAATGGTGCTGCAGGGAATGCACAATGCCCCCACTGTCGTTCCGGGCTAAA 3360
FSS13025            GTGATCGAGGAATGGTGCTGCAGGGAGTGCACAATGCCCCCACTGTCGTTCCGGGCTAAA 3360
PLCal_ZV            GTGATCGAGGAATGGTGCTGCAGGGAGTGCACAATGCCCCCACTGTCGTTCCGGGCTAAA 3249
SV0127/14           GTGATCGAGGAATGGTGCTGCAGGGAGTGCACAATGCCCCCACTGTCGTTCCGGGCTAAA 3360
8375                GTGATCGAGGAATGGTGCTGCAGGGAGTGCACAATGCCCCCACTGTCGTTCCGGGCTAAA 3360
103344              GTGATCGAGGAATGGTGCTGCAGGGAGTGCACAATGCCCCCACTGTCGTTCCGGGCTAAA 3360
BrasilZKV2015       GTGATCGAGGAATGGTGCTGCAGGGAGTGCACAATGCCCCCACTGTCGTTCCGGGCTAAA 3360
GD01                GTGATCGAGGAATGGTGCTGCAGGGAGTGCACAATGCCCCCACTGTCGTTCCGGGCTAAA 3360
Martinique          GTGATCGAGGAATGGTGCTGCAGGGAGTGCACAATGCCCCCACTGTCGTTCTGGGCTAAA 3360
NatalRGN            GTGATCGAGGAATGGTGCTGCAGGGAGTGCACAATGCCCCCACTGTCGTTCCGGGCTAAA 3360
HPF2013             GTGATCGAGGAATGGTGCTGCAGGGAGTGCACAATGCCCCCACTGTCGTTCCGGGCTAAA 3360
SPH2015             GTGATCGAGGAATGGTGCTGCAGGGAGTGCACAATGCCCCCACTGTCGTTCCGGGCTAAA 3360
Haiti2014           GTGATCGAGGAATGGTGCTGCAGGGAGTGCACAATGCCCCCACTGTCGTTCCGGGCTAAA 3360
PRVABC59            GTGATCGAGGAATGGTGCTGCAGGGAGTGCACAATGCCCCCACTGTCGTTCCGGGCTAAA 3360
BeH819015           GTGATCGAGGAATGGTGCTGCAGGGAGTGCACAATGCCCCCACTGTCGTTCCGGGCTAAA 3360
Z1106033            GTGATCGAGGAATGGTGCTGCAGGGAGTGCACAATGCCCCCACTGTCGTTCCGGGCTAAA 3360
BEH819966           GTGATCGAGGAATGGTGCTGCAGGGAGTGCACAATGCCCCCACTGTCGTTCCGGGCTAAA 3360
SSABr               GTGATCGAGGAATGGTGCTGCAGGGAGTGCACAATGCCCCCACTGTCGTTCCGGGCTAAA 3360
Beh815744           GTGATCGAGGAATGGTGCTGCAGGGAGTGCACAATGCCCCCACTGTCGTTCCGGGCTAAA 3360
BEH818995           GTGATCGAGGAATGGTGCTGCAGGGAGTGCACAATGCCCCCACTGTCGTTCCGGGCTAAA 3360
IbH30656            GTCATAGAGGAATGGTGCTGTAGGGAATGCACAATGCCTCCACTATCGTTCCGGGCAAAA 3342
ArD128000           GTCATTGAGGAATGGTGCTGTAGGGAATGCACAATGCCCCCACTATCGTTTCGAGCAAAA 3360
ArD7117             GTCATAGAGGAATGGTGCTGTAGGGAATGCACAATGCCTCCACTATCGTTCCGGGCGAAA 3360
ARD_41519           GTCATAGAGGAATGGTGCTGTAGGGAATGCACAATGCCTCCACTATCGTTCCGGGCAAAA 3360
ARD157995           GTCATTGAGGAATGGTGCTGTAGGGAATGCACAATGCCCCCACTATCGTTTCGAGCAAAA 3360
MR_766              GTCATTGAGGAATGGTGCTGTAGGGAATGCACAATGCCCCCACTATCGTTTCGAGCAAAA 3348
ARD158084           GTCATTGAGGAATGGTGCTGTAGGGAATGCACAATGCCCCCACTATCGTTTCGAGCAAAA 3360
ArB1362             GTCATTGAGGAATGGTGCTGTAGAGAATGCACAATGCCCCCACTATCATTTCGAGCGAAA 3360
ARB13565            GTCATTGAGGAATGGTGCTGTAGAGAATGCACAATGCCCCCACTATCGTTTCGAGCAAAA 3360
ARB7701             GTCATTGAGGAATGGTGCTGTAGAGAATGCACAATGCCCCCACTATCGTTTCGAGCAAAA 3360
ARB15076            GTCATTGAGGAATGGTGCTGTAGAGAATGCACAATGCCCCCACTATCGTTTCGAGCAAAA 3342
                    ** ** ************** ** ** *********** *** * ** **  * ** ***

P6-740              GATGGCTGTTGGTATGGAATGGAGATAAGGCCCAGGAAGGAACCAGAGAGTAACCTAGTA 3420
CPC0740             GATGGCTGTTGGTATGGAATGGAGATAAGGCCCAGGAAAGAACCAGAAAGTAACTTAGTA 3420
YAP2007             GATGGCTGTTGGTATGGAATGGAGATAAGGCCCAGGAAAGAACCAGAAAGTAACTTAGTA 3420
FSS13025            GATGGTTGTTGGTATGGAATGGAGATAAGGCCCAGGAAAGAACCAGAAAGTAACTTAGTA 3420
PLCal_ZV            GATGGCTGTTGGTATGGAATGGAGATAAGGCCCAGGAAAGAACCAGAAAGCAACTTAGTA 3309
SV0127/14           GATGGCTGTTGGTATGGAATGGAGATAAGGCCCAGGAAAGAACCAGAAAGTAACTTAGTA 3420
8375                GATGGCTGTTGGTATGGAATGGAGATAAGGCCCAGGAAAGAACCAGAAAGCAACTTAGTA 3420
```

```
103344          GATGGCTGTTGGTATGGAATGGAGATAAGGCCCAGGAAAGAACCAGAAAGCAACTTAGTA 3420
BrasilZKV2015   GATGGCTGTTGGTATGGAATGGAGATAAGGCCCAGGAAAGAACCAGAAAGCAACTTAGTA 3420
GD01            GATGGCTGTTGGTATGGAATGGAGATAAGGCCCAGGAAAGAACCAGAAAGCAACTTAGTA 3420
Martinique      GATGGCTGTTGGTATGGAATGGAGATAAGGCCCAGGAAAGAACCAGAAAGCAACTTAGTA 3420
NatalRGN        GATGGCTGTTGGTATGGAATGGAGATAAGGCCCAGGAAAGAACCAGAAAGCAACTTAGTA 3420
HPF2013         GATGGCTGTTGGTATGGAATGGAGATAAGGCCCAGGAAAGAACCAGAAAGTAACTTAGTA 3420
SPH2015         GATGGCTGTTGGTATGGAATGGAGATAAGGCCCAGGAAAGAACCAGAAAGCAACTTAGTA 3420
Haiti2014       GATGGCTGTTGGTATGGAATGGAGATAAGGCCCAGGAAAGAACCAGAAAGCAACTTAGTA 3420
PRVABC59        GATGGCTGTTGGTATGGAATGGAGATAAGGCCCAGGAAAGAACCAGAAAGCAACTTAGTA 3420
BeH819015       GATGGCTGTTGGTATGGAATGGAGATAAGGCCCAGGAAAGAACCAGAAAGCAACTTAGTA 3420
Z1106033        GATGGCTGTTGGTATGGAATGGAGATAAGGCCCAGGAAAGAACCAGAAAGCAACTTAGTA 3420
BEH819966       GATGGCTGTTGGTATGGAATGGAGATAAGGCCCAGGAAAGAACCAGAAAGCAACTTAGTA 3420
SSABr           GATGGCTGTTGGTATGGAATGGAGATAAGGCCCAGGAAAGAACCAGAAAGCAACTTAGTA 3420
Beh815744       GATGGCTGTTGGTATGGAATGGAGATAAGGCCCAGGAAAGAACCAGAAAGCAACTTAGTA 3420
BEH818995       GATGGCTGTTGGTATGGAATGGAGATAAGGCCCAGGAAAGAACCAGAAAGCAACTTAGTA 3420
IbH30656        GACGGCTGCTGGTATGGAATGGAGATAAGGCCCAGAAAGGAACCAGAGAGCAACTTAGTG 3402
ArD128000       GACGGCTGCTGGTATGGAATGGAGATAAGGCCCAGGAAAGAACCAGAGAGCAACTTAGTG 3420
ArD7117         GACGGCTGCTGGTATGGAATGGAGATAAGGCCCAGAAAGGAACCAGAGAGCAACTTAGTA 3420
ARD_41519       GACGGCTGCTGGTATGGAATGGAGATAAGGCCCAGAAAGGAACCAGAGAGCAACTTAGTA 3420
ARD157995       GACGGCTGCTGGTATGGAATGGAGATAAGGCCCAGGAAAGAACCAGAGAGCAACTTAGTG 3420
MR_766          GACGGCTGCTGGTATGGAATGGAGATAAGGCCCAGGAAAGAACCAGAGAGCAACTTAGTG 3408
ARD158084       GACGGCTGCTGGTATGGAATGGAGATAAGGCCCAGGAAAGAACCAGAGAGCAACTTAGTG 3420
ArB1362         GACGGCTGCTGGTATGGAATGGAGATAAGGCCCAGGAAAGAACCAGAGAGCAATTTAGTG 3420
ARB13565        GACGGCTGCTGGTATGGAATGGAGATAAGGCCCAGGAAAGAACCAGAGAGCAACTTAGTG 3420
ARB7701         GACGGCTGCTGGTATGGAATGGAGATAAGGCCCAGGAAAGAACCAGAGAGCAACTTAGTG 3420
ARB15076        GATGGCTGCTGGTATGGAATGGAGATAAGGCCCAGGAAAGAACCAGAGAGCAACTTAGTG 3402
                ** ** ** ************************** ** ******** ** **   ****

P6-740          AGGTCAATGGTGACTGCAGGATCAACTGATCACATGGATCACTTCTCCCTTGGAGTGCTT 3480
CPC0740         AGGTCAATGGTGACTGCAGGATCAACTGATCACATGGATCACTTCTCTCTTGGAGTGCTT 3480
YAP2007         AGGTCAATGGTGACTGCAGGATCAACTGATCACATGGATCACTTCTCCCTTGGAGTGCTT 3480
FSS13025        AGGTCAATGGTGACTGCAGGATCAACTGATCACATGGATCACTTCTCCCTTGGAGTGCTT 3480
PLCal_ZV        AGGTCAATGGTGACTGCAGGATCAACTGATCACATGGATCACTTCTCCCTTGGAGTGCTT 3369
SV0127/14       AGGTCAATGGTGACTGCAGGATCAACTGATCACATGGATCACTTTTCCCTTGGAGTGCTT 3480
8375            AGGTCAATGGTGACTGCAGGATCAACTGATCACATGGATCACTTCTCCCTTGGAGTGCTT 3480
103344          AGGTCAATGGTGACTGCAGGATCAACTGATCACATGGATCACTTCTCCCTTGGAGTGCTT 3480
BrasilZKV2015   AGGTCAATGGTGACTGCAGGATCAACTGATCACATGGATCACTTCTCCCTTGGAGTGCTT 3480
GD01            AGGTCAATGGTGACTGCAGGATCAACTGATCACATGGACCACTTCTCCCTTGGAGTGCTT 3480
Martinique      AGGTCAATGGTGACTGCAGGATCAACTGATCACATGGATCACTTCTCCCTTGGAGTGCTT 3480
NatalRGN        AGGTCAGTGGTGACTGCAGGATCAACTGATCACATGGATCACTTCTCCCTTGGAGTGCTT 3480
HPF2013         AGGTCAATGGTGACTGCAGGATCAACTGATCACATGGATCACTTCTCCCTTGGAGTGCTT 3480
SPH2015         AGGTCAATGGTGACTGCAGGATCAACTGATCACATGGATCACTTCTCCCTTGGAGTGCTT 3480
Haiti2014       AGGTCAATGGTGACTGCAGGATCAACTGATCACATGGATCACTTCTCCCTTGGAGTGCTT 3480
PRVABC59        AGGTCAATGGTGACTGCAGGATCAACTGATCACATGGACCACTTCTCCCTTGGAGTGCTT 3480
BeH819015       AGGTCAATGGTGACTGCAGGATCAACTGATCACATGGACCACTTCTCCCTTGGAGTGCTT 3480
Z1106033        AGGTCAATGGTGACTGCAGGATCAACTGATCACATGGACCACTTCTCCCTTGGAGTGCTT 3480
BEH819966       AGGTCAATGGTGACTGCAGGATCAACTGATCACATGGACCACTTCTCCCTTGGAGTGCTT 3480
SSABr           AGGTCAATGGTGACTGCAGGATCAACTGATCACATGGACCACTTCTCCCTTGGAGTGCTT 3480
Beh815744       AGGTCAATGGTGACTGCAGGATCAACTGATCACATGGACCACTTCTCCCTTGGAGTGCTT 3480
BEH818995       AGGTCAATGGTGACTGCAGGATCAACTGATCACATGGACCACTTCTCCCTTGGAGTGCTT 3480
IbH30656        AGGTCTATGGTGACAGCAGGATCAACCGATCACATGGATCACTTCTCTCTTGGAGTGCTT 3462
ArD128000       AGGTCAATGGTGACAGCGGGGTCAACCGATCATATGGACCACTTCTCTCTTGGAGTGCTT 3480
ArD7117         AGGTCTATGGTGACAGCAGGATCAACCGATCATATGGATCACTTCTCTCTTGGAGTGCTT 3480
ARD_41519       AGGTCTATGGTGACAGCAGGATCAACCGATCATATGGATCACTTCTCTCTTGGAGTGCTT 3480
ARD157995       AGGTCAATGGTGACAGCGGGGTCAACCGATCATATGGACCACTTCTCTCTTGGAGTGCTT 3480
MR_766          AGGTCAATGGTGACAGCGGGGTCAACCGATCATATGGACCACTTCTCTCTTGGAGTGCTT 3468
ARD158084       AGGTCAATGGTGACAGCGGGGTCAACCGATCATATGGACCACTTCTCTCTTGGAGTGCTT 3480
ArB1362         AGGTCAATGGTGACAGCGGGATCAACCGATCACATGGACCACTTCTCTCTTGGAGTGCTT 3480
ARB13565        AGGTCAATGGTGACAGCGGGATCAACCGATCACATGGACCACTTCTCTCTTGGAGTGCTT 3480
ARB7701         AGGTCAATGGTGACAGCGGGATCAACCGATCACATGGACCACTTCTCTCTTGGAGTGCTT 3480
ARB15076        AGGTCAATGGTGACAGCGGGATCAACCGATCACATGGACCACTTCTCTCTTGGAGTGCTT 3462
                *****  ******* ** ** ***** ***** ***** ***** ** ************

P6-740          GTGATTCTGCTCATGGTGCAGGAAGGGCTGAAGAAGAGAATGACCACAAAGATCATCATA 3540
CPC0740         GTGATTTTGCTCATGGTGCAGGAAGGGCTGAAGAAGAGAATGACCACAAAGATCATCATA 3540
YAP2007         GTGATTCTGCTCATGGTGCAGGAAGGGCTGAAGAAGAGAATGACCACAAAGATCATCATA 3540
FSS13025        GTGATTCTGCTCATGGTACAGGAAGGGCTAAAGAAGAGAATGACCACAAAGATCATCATA 3540
PLCal_ZV        GTGATTCTGCTCATGGTGCAGGAAGGGCTGAAGAAGAGAATGACCACAAAGATCATCATA 3429
```

```
SV0127/14          GTGATTCTGCTCATGGTGCAGGAAGGGCTGAAGAAGAGAATGACCACAAAGATCATCATA 3540
8375               GTGATTCTGCTCATGGTGCAGGAAGGGCTAAAGAAGAGAATGACCACAAAGATCATCATA 3540
103344             GTGATTCTGCTCATGGTGCAGGAAGGGCTAAAGAAGAGAATGACCACAAAGATCATCATA 3540
BrasilZKV2015      GTGATTCTGCTCATGGTGCAGGAAGGGCTGAAGAAGAGAATGACCACAAAGATCATCATA 3540
GD01               GTGATCCTGCTCATGGTGCAGGAAGGGCTGAAGAAGAGAATGACCACAAAGATCATCATA 3540
Martinique         GTGATTCTGCTCATGGTGCAGGAAGGGCTGAAGAAGAGAATGACCACAAAGATCATCATA 3540
NatalRGN           GTGATTCTGCTCATGGTGCAGGAAGGGCTGAAGAAGAGAATGACCACAAAGATCATCATA 3540
HPF2013            GTGATTCTGCTCATGGTGCAGGAAGGGCTGAAGAAGAGAATGACCACAAAGATCATCATA 3540
SPH2015            GTGATTCTGCTCATGGTGCAGGAAGGGCTGAAGAAGAGAATGACCACAAAGATCATCATA 3540
Haiti2014          GTGATTCTGCTCATGGTGCAGGAAGGGCTGAAGAAGAGAATGACCACAAAGATCATCATA 3540
PRVABC59           GTGATCCTGCTCATGGTGCAGGAAGGGCTGAAGAAGAGAATGACCACAAAGATCATCATA 3540
BeH819015          GTGATCCTGCTCATGGTGCAGGAAGGGCTGAAGAAGAGAATGACCACAAAGATCATCATA 3540
Z1106033           GTGATTCTGCTCATGGTGCAGGAAGGGTTGAAGAAGAGAATGACCACAAAGATCATCATA 3540
BEH819966          GTGATTCTGCTCATGGTGCAGGAAGGGCTGAAGAAGAGAATGACCACAAAGATCATCATA 3540
SSABr              GTGATTCTGCTCATGGTGCAGGAAGGGCTGAAGAAGAGAATGACCACAAAGATCATCATA 3540
Beh815744          GTGATTCTGCTCATGGTGCAGGAAGGGCTGAAGAAGAGAATGACCACAAAGATCATCATA 3540
BEH818995          GTGATTCTGCTCATGGTGCAGGAAGGGCTGAAGAAGAGAATGACCACAAAGATCATCATA 3540
IbH30656           GTGATTCTACTCATGGTGCAGGAAGGTTTGAAGAAGAGAATGACCACAAAGATCATAATG 3522
ArD128000          GTGATTCTACTCATGGTGCAGGAGGGGTTGAAGAAGAGAATGACCACAAAGATCATCATG 3540
ArD7117            GTGATTCTACTCATGGTGCAGGAAGGTTTGAAGAAGAGAATGACCACAAAGATCATAATG 3540
ARD_41519          GTGATTCTACTCATGGTGCAGGAAGGTTTGAAGAAGAGAATGACCACAAAGATCATAATG 3540
ARD157995          GTGATTCTACTCATGGTGCAGGAGGGGTTGAAGAAGAGAATGACCACAAAGATCATCATG 3540
MR_766             GTGATTCTACTCATGGTGCAGGAGGGGTTGAAGAAGAGAATGACCACAAAGATCATCATG 3528
ARD158084          GTGATTCTACTCATGGTGCAGGAGGGGTTGAAGAAGAGAATGACCACAAAGATCATCATG 3540
ArB1362            GTGATTCTACTCATGGTGCAGGAAGGGCTGAAGAAGAGAATGACCACGAAGATCATTGTG 3540
ARB13565           GTGATTCTACTCATGGTGCAGGAAGGGCTGAAGAAGAGAATGACCACAAAGATCATTATG 3540
ARB7701            GTGATTCTACTCATGGTGCNNGAAGGGCTGAAGAAGAGAATGACCACAAAGATCATTATG 3540
ARB15076           GTGATTCTACTCATGGTGCAGGAAGGGTTGAAGAAGAGAATGACCACGAAGATCATTATG 3522
                   *****  * ******** *  ** **  * ***************** ********  *

P6-740             AGCACATCAATGGCAGTGTTGGTAGCTATGATCCTGGGAGGATTTTCAATGAGTGACTTG 3600
CPC0740            AGCACATCAATGGCAGTGCTGGTAGCCATGATCCTGGGAGGATTTTCAATGAGTGACCTG 3600
YAP2007            AGCACATCAATGGCAGTGCTGGTAGCTATGATCCTGGGAGGATTTTCAATGAGTGACCTG 3600
FSS13025           AGCACATCAATGGCAGTGCTGGTAGCTATGATCCTGGGAGGATTTTCAATGAGTGACCTG 3600
PLCal_ZV           AGCACATCAATGGCAGTGCTGGTAGCTATGATCCTGGGAGGATTTTCAATGAGTGACCTG 3489
SV0127/14          AGCACATCAATGGCAGTGCTGGTAGCTATGATCCTGGGAGGATTTTCAATGAGTGATCTG 3600
8375               AGCACATCAATGGCAGTGCTGGTAGCTATGATCCTGGGAGGATTTTCAATGAGTGACCTG 3600
103344             AGCACATCAATGGCAGTGCTGGTAGCTATGATCCTGGGAGGATTTTCAATGAGTGACCTG 3600
BrasilZKV2015      AGCACATCAATGGCAGTGCTGGTAGCTATGATCCTGGGAGGATTTTCAATGAGTGACCTG 3600
GD01               AGCACATCAATGGCAGTGCTGGTAGCTATGATCCTGGGAGGATTTTCAATGAGTGACCTG 3600
Martinique         AGCACATCAATGGCAGTGCTGGTAGCTATGATCCTGGGAGGATTTTCAATGAGTGACCTG 3600
NatalRGN           AGCACATCAATGGCAGTGCTGGTAGCTATGATCCTGGGAGGATTTTCAATGAGTGACCTG 3600
HPF2013            AGCACATCGATGGCAGTGCTGGTAGCTATGATCCTGGGAGGATTTTCAATGAGTGACCTG 3600
SPH2015            AGCACATCAATGGCAGTGCTGGTAGCTATGATCCTGGGAGGATTTTCAATGAGTGACCTG 3600
Haiti2014          AGCACATCAATGGCAGTGCTGGTAGCTATGATCCTGGGAGGATTTTCAATGAGTGACCTG 3600
PRVABC59           AGCACATCAATGGCAGTGCTGGTAGCTATGATCCTGGGAGGATTTTCAATGAGTGACCTG 3600
BeH819015          AGCACATCAATGGCAGTGCTGGTAGCTATGATCCTGGGAGGATTTTCAATGAGTGACCTG 3600
Z1106033           AGCACATCAATGGCAGTGCTGGTAGCTATGATCCTGGGAGGATTTTCAATGAGTGACCTG 3600
BEH819966          AGCACATCAATGGCAGTGCTGGTAGCTATGATCCTGGGAGGATTTTCAATGAGTGACCTG 3600
SSABr              AGCACATCAATGGCAGTGCTGGTAGCTATGATCCTGGGAGGATTTTCAATGAGTGACCTG 3600
Beh815744          AGCACATCAATGGCAGTGCTGGTAGCTATGATCCTGGGAGGATTTTCAATGAGTGACCTG 3600
BEH818995          AGCACATCAATGGCAGTGCTGGTAGCTATGATCCTGGGAGGATTTTCAATGAGTGACCTG 3600
IbH30656           AGCACATCAATGGCAATGCTGGTAGCCATGGTCTTGGGAGGATTCTCAATGAGTGACCTG 3582
ArD128000          AGCACATCAATGGCAGTGCTGGTAGCCATGGTCTTGGGAGGATTCTCAATGAGTGATCTG 3600
ArD7117            AGCACATCAATGGCAGTGCTGGTAGCCATGGTCTTGGGAGGATTCTCAATGAGTGACCTG 3600
ARD_41519          AGCACATCAATGGCAGTGCTGGTAGCCATGGTCTTGGGAGGATTCTCAATGAGTGACCTG 3600
ARD157995          AGCACATCAATGGCAGTGCTGGTAGTCATGATCTTGGGAGGATTTTCAATGAGTGACCTG 3600
MR_766             AGCACATCAATGGCAGTGCTGGTAGTCATGATCTTGGGAGGATTTTCAATGAGTGACCTG 3588
ARD158084          AGCACATCAATGGCAGTGCTGGTAGTCATGATCTTGGGAGGATTTTCAATGAGTGACCTG 3600
ArB1362            AGCACATCAATGGCAGTGCTGGTAGCCATGATCTTGGGAGGATTCTCAATGAGTGACCTG 3600
ARB13565           AGCACATCAATGGCAGTGCTGGTGGCCATGATCCTGGGAGGATTTTTAATGAGTGACTTG 3600
ARB7701            AGCACATCAATGGCAGTGCTGGTGGCCATGATCCTGGGAGGATTTTTAATGAGTGACTTG 3600
ARB15076           AGCACATCAATGGCAGTGCTGGTAGCCATGATCCTGGGAGGATTTTCAATGAGTGACCTG 3582
                   ******** ****** ** **** *  *** ** ********** * *********  **

P6-740             GCTAAGCTTGCAATTCTGATGGGTGCCACCTTCGCGGAAATGAACACTGGAGGAGATGTA 3660
CPC0740            GCTAAGCTTGCAATTTTGATGGGTGCCACCTTCGCGGAAATGAACACTGGAGGAGATGTA 3660
YAP2007            GCCAAGCTTGCAATTTTGATGGGTGCCACCTTTGCGGAAATGAACACTGGAGGAGATGTA 3660
```

```
FSS13025         GCTAAGCTTGCAATTTTGATGGGTGCCACCTTCGCGGAAATGAACACTGGAGGAGATGTT 3660
PLCal_ZV         GCTAAGCTTGCAATTTTGATGGGTGCTACCTTCGCGGAAATGAACACTGGAGGAGATGTA 3549
SV0127/14        GCTAAGCTTGCAATTTTGATGGGTGCCACCTTTGCGGAAATGAACACTGGAGGAGATGTA 3660
8375             GCTAAGCTTGCAATTTTGATGGGTGCCACCTTCGCGGAAATGAACACTGGAGGAGATGTA 3660
103344           GCTAAGCTTGCAATTTTGATGGGTGCCACCTTCGCGGAAATGAACACTGGAGGAGATGTA 3660
BrasilZKV2015    GCTAAGCTTGCAATTTTGATGGGTGCCACCTTCGCGGAAATGAACACTGGAGGAGATGTA 3660
GD01             GCTAAGCTTGCAATTTTGATGGGTGCCACCTTCGCGGAAATGAACACTGGAGGAGATGTA 3660
Martinique       GCTAAGCTTGCAATTTTGATGGGTGCCACCTTCGCGGAAATGAACACTGGAGGAGATGTA 3660
NatalRGN         GCTAAGCTTGCAATTTTGATGGGCGCCACCTTCGCGGAAATGAACACTGGAGGAGATGTA 3660
HPF2013          GCTAAGCTTGCAATTTTGATGGGTGCCACCTTCGCGGAAATGAACACTGGAGGAGATGTA 3660
SPH2015          GCTAAGCTTGCAATTTTGATGGGTGCCACCTTCGCGGAAATGAACACTGGAGGAGATGTA 3660
Haiti2014        GCTAAGCTTGCAATTTTGATGGGTGCCACCTTCGCGGAAATGAACACTGGAGGAGATGTA 3660
PRVABC59         GCTAAGCTTGCAATTTTGATGGGTGCCACCTTCGCGGAAATGAACACTGGAGGAGATGTA 3660
BeH819015        GCTAAGCTTGCAATTTTGATGGGTGCCACCTTCGCGGAAATGAACACTGGAGGAGATGTA 3660
Z1106033         GCTAAGCTTGCAATTTTGATGGGTGCCACCTTCGCGGAAATGAACACTGGAGGAGATGTA 3660
BEH819966        GCTAAGCTTGCAATTTTGATGGGTGCCACCTTCGCGGAAATGAACACTGGAGGAGATGTA 3660
SSABr            GCTAAGCTTGCAATTTTGATGGGTGCCACCTTCGCGGAAATGAACACTGGAGGAGATGTA 3660
Beh815744        GCTAAGCTTGCAATTTTGATGGGTGCCACCTTCGCGGAAATGAACACTGGAGGAGATGTA 3660
BEH818995        GCTAAGCTTGCAATTTTGATGGGTGCCACCTTCGCGGAAATGAACACTGGAGGAGATGTA 3660
IbH30656         GCTAAGCTTGTGATCCTGATGGGTGCCACTTTCGCAGAAATGAACACTGGAGGAGATGTG 3642
ArD128000        GCTAAGCTTGTGATCCTGATGGGTGCCACTTTCGCAGAAATGAACACTGGAGGAGATGTG 3660
ArD7117          GCTAAGCTTGTGATCCTGATGGGTGCCACTTTCGCAGAAATGAACACTGGAGGAGATGTG 3660
ARD_41519        GCTAAGCTCGTGATCCTGATGGGTGCCACTTTCGCAGAAATGAACACTGGAGGAGATGTG 3660
ARD157995        GCCAAGCTTGTGATCCTGATGGGTGCTACTTTCGCAGAAATGAACACTGGAGGAGATGTA 3660
MR_766           GCCAAGCTTGTGATCCTGATGGGTGCTACTTTCGCAGAAATGAACACTGGAGGAGATGTA 3648
ARD158084        GCCAAGCTTGTGATCCTGATGGGTGCTACTTTCGCAGAAATGAACACTGGAGGAGATGTA 3660
ArB1362          GCTAAGCTTGTGATCCTGATGGGTGCTACTTTCGCAGAAATGAATACTGGAGGAGATGTG 3660
ARB13565         GCTAAGCTTGTGATCCTGATGGGTGCCACCTTCGCAGAAATGAACACTGGAGGAGATGTG 3660
ARB7701          GCTAAGCTTGTGATCCTGATGGGTGCCACCTTCGCAGAAATGAACACTGGAGGAGATGTG 3660
ARB15076         GCTAAGCTTGTGATCCTGATGGGTGCCACCTTCGCAGAAATGAACACTGGAGGAGATGTG 3642
                 ** ***** *  **  ******* ** ** ** ** ******** **************

P6-740           GCTCATCTGGCGCTGATAGCGGCATTCAAAGTCAGACCCGCGTTGCTGGTCTCTTTCATC 3720
CPC0740          GCTCATTTGGCGCTGATAGCGGCATTCAAAGTCAGACCTGCGTTGCTGGTATCTTTCATC 3720
YAP2007          GCTCATCTGGCGCTGATAGCGGCATTCAAAGTCAGACCTGCGTTGCTGGTATCTTTCATC 3720
FSS13025         GCTCATCTGGCGCTGATAGCGGCATTCAAAGTCAGACCTGCGTTGCTGGTATCTTTCATT 3720
PLCal_ZV         GCACATCTGGCGCTGATAGCGGCATTCAAAGTCAGACCAGCGTTGCTGGTATCTTTCATC 3609
SV0127/14        GCTCATCTGGCGCTGGTAGCGGCATTCAAAGTCAGACCAGCGTTGCTGGTATCTTTCATC 3720
8375             GCTCATCTGGCGCTGATAGCGGCATTCAAAGTCAGACCAGCGTTGCTGGTATCTTTCATC 3720
103344           GCTCATCTGGCGCTGATAGCGGCATTCAAAGTCAGACCAGCGTTGCTGGTATCTTTCATC 3720
BrasilZKV2015    GCTCATCTGGCGCTGATAGCGGCATTCAAAGTCAGACCAGCGTTGCTGGTATCTTTCATC 3720
GD01             GCTCATCTGGCGCTGATAGCGGCATTCAAAGTCAGACCAGCGTTGCTGGTATCTTTCATC 3720
Martinique       GCTCATCTGGCGCTGACAGCGGCATTCAAAGTCAGACCAGCGTTGCTGGTATCTTTCATC 3720
NatalRGN         GCTCATCTGGCGCTGATAGCGGCATTCAAAGTCAGACCAGCGTTGCTGGTATCTTTCATC 3720
HPF2013          GCTCATCTGGCGCTGATAGCGGCATTCAAAGTCAGACCAGCGTTGCTGGTATCTTTCATC 3720
SPH2015          GCTCATCTGGCGCTGATAGCGGCATTCAAAGTCAGACCAGCGTTGCTGGTATCTTTCATC 3720
Haiti2014        GCTCATCTGGCGCTGATAGCGGCATTCAAAGTCAGACCAGCGTTGCTGGTATCTTTCATC 3720
PRVABC59         GCTCATCTGGCGCTGATAGCGGCATTCAAAGTCAGACCAGCGTTGCTGGTATCTTTCATC 3720
BeH819015        GCTCATCTGGCGCTGATAGCGGCATTCAAAGTCAGACCAGCGTTGCTGGTATCTTTCATC 3720
Z1106033         GCTCATCTGGCGCTGATAGCGGCATTCAAAGTCAGACCAGCGTTGCTGGTATCTTTCATC 3720
BEH819966        GCTCATCTGGCGCTGATAGCGGCATTCAAAGTCAGACCAGCGTTGCTGGTATCTTTCATC 3720
SSABr            GCTCATCTGGCGCTGATAGCGGCATTCAAAGTCAGACCAGCGTTGCTGGTATCTTTCATC 3720
Beh815744        GCTCATCTGGCGCTGATAGCGGCATTCAAAGTCAGACCAGCGTTGCTGGTATCTTTCATC 3720
BEH818995        GCTCATCTGGCGCTGATAGCGGCATTCAAAGTCAGACCAGCGTTGCTGGTATCTTTCATC 3720
IbH30656         GCTCACTTGGCATTGGTAGCGGCATTTAAAGTCAGACCAGCCTTGTTGGTTTCCTTCATC 3702
ArD128000        GCTCACTTGGCATTGGTTGCGGCATTTAAAGTCAGACCAGCCTTGTTGGTTTCCTTTATC 3720
ArD7117          GCTCACTTGGCATTGGTTGCGGCATTTAAAGTCAGACCAGCCTTGTTGGTTTCCTTCATC 3720
ARD_41519        GCTCACTTGGCATTGGTTGCGGCATTTAAAGTCAGACCAGCCTTGTTGGTTTCCTTCATC 3720
ARD157995        GCTCACTTGGCATTGGTAGCGGCATTTAAAGTCAGACCAGCCTTGCTGGTCTCCTTCATT 3720
MR_766           GCTCACTTGGCATTGGTAGCGGCATTTAAAGTCAGACCAGCCTTGCTGGTCTCCTTCATT 3708
ARD158084        GCTCACTTGGCATTGGTAGCGGCATTTAAAGTCAGACCAGCCTTGCTGGTCTCCTTCATT 3720
ArB1362          GCTCACTTGGCATTGGTCGCGGCATTCAAAGTCAGACCAGCCTTGCTGGTCTCCTTCATT 3720
ARB13565         GCTCACTTGGCATTGGTTGCGGCATTTAAAGTCAGACCAGCCTTGCTGGTCTCCTTTATT 3720
ARB7701          GCTCACTTGGCATTGGTTGCGGCATTTAAAGTCAGACCAGCCTTGCTGGTCTCCTTTATT 3720
ARB15076         GCTCACTTGGCATTGGTTGCGGCATTTAAAGTCAGACCAGCCTTGCTGGTCTCCTTTATT 3702
                 ** **  ****  **   ******** *********** ** *** **** ** ** **

P6-740           TTCAGAGCCAATTGGACACCCCGTGAGAGCATGCTGCTGGCCTTGGCCTCGTGCCTTCTG 3780
```

```
CPC0740          TTCAGAGCTAATTGGACACCCCGTGAGAGCATGCTGCTGGCCTTGGCCTCGTGTCTTCTG 3780
YAP2007          TTCAGAGCTAATTGGACACCCCGTGAGAGCATGCTGCTGGCCCTGGCTTCGTGTCTTCTG 3780
FSS13025         TTCAGAGCTAATTGGACACCCCGTGAGAGCATGCTGCTGGCCTTGGCCTCGTGTCTTCTG 3780
PLCal_ZV         TTCAGAGCTAATTGGACACCCCGTGAAAGCATGCTGCTGGCCCTGGCCTCGTGTCTTCTG 3669
SV0127/14        TTCAGAGCTAATTGGACACCCCGTGAAAGCATGCTGCTGGCCTTGGCCTCGTGTCTTTTG 3780
8375             TTCAGAGCTAATTGGACACCCCGTGAAAGCATGCTACTGGCCTTGGCCTCGTGTCTTTTG 3780
103344           TTCAGAGCTAATTGGACACCCCGTGAAAGCATGCTACTGGCCTTGGCCTCGTGTCTTTTG 3780
BrasilZKV2015    TTCAGAGCTAATTGGACACCCCGTGAAAGCATGCTGCTGGCCTTGGCCTCGTGTTTTTTG 3780
GD01             TTCAGAGCTAATTGGACACCCCGTGAAAGCATGCTGCTGGCCTTGGCCTCGTGTCTTTTG 3780
Martinique       TTCAGAGCTAATTGGACACCCCGTGAAAGCATGCTGCTGGCCTTGGCCTCGTGTCTTTTG 3780
NatalRGN         TTCAGAGCTAATTGGACACCCCGTGAAAGCATGCTGCTGGCCTTGGCCTCGTGTCTTTTG 3780
HPF2013          TTCAGAGCTAATTGGACACCCCGTGAAAGCATGCTGCTGGCCTTGGCCTCGTGTCTTTTG 3780
SPH2015          TTCAGAGCTAATTGGACACCCCGTGAAAGCATGCTGCTGGCCTTGGCCTCGTGTCTTTTG 3780
Haiti2014        TTCAGAGCTAATTGGACACCCCGTGAAAGCATGCTGCTGGCCTTGGCCTCGTGTCTTTTG 3780
PRVABC59         TTCAGAGCTAATTGGACACCCCGTGAAAGCATGCTGCTGGCCTTGGCCTCGTGTCTTTTG 3780
BeH819015        TTCAGAGCTAATTGGACACCCCGTGAAAGCATGCTGCTGGCCTTGGCCTCGTGTCTTTTG 3780
Z1106033         TTCAGAGCTAATTGGACACCCCGTGAAAGCATGCTGCTGGCCTTGGCCTCGTGTCTTTTG 3780
BEH819966        TTCAGAGCTAATTGGACACCCCGTGAAAGCATGCTGCTGGCCTTGGCCTCGTGTCTTTTG 3780
SSABr            TTCAGAGCTAATTGGACACCCCGTGAAAGCATGCTGCTGGCCTTGGCCTCGTGTCTTTTG 3780
Beh815744        TTCAGAGCTAATTGGACACCCCGTGAAAGCATGCTGCTGGCCTTGGCCTCGTGTCTTTTG 3780
BEH818995        TTCAGAGCTAATTGGACACCCCGTGAAAGCATGCTGCTGGCCTTGGCCTCGTGTCTTTTG 3780
IbH30656         TTCAGAGCCAACTGGACACCCCGTGAGAGCATGCTGCTAGCCCTGGCTTCGTGTCTCCTG 3762
ArD128000        TTCAGAGCCAACTGGACACCCCGTGAGAGCATGCTGCTAGCCCTGGCTTCGTGTCTCCTG 3780
ArD7117          TTCAGAGCCAACTGGACACCCCGTGAGAGCATGCTGCTAGCCCTGGCTTCGTGTCTCCTG 3780
ARD_41519        TTCAGAGCCAACTGGACACCCCGTGAGAGCATGCTGCTAGCCCTGGCTTCGTGCCTCCTG 3780
ARD157995        CTCAGAGCCAATTGGACACCCCGTGAGAGCATGCTGCTAGCCCTGGCTTCGTGTCTTCTG 3780
MR_766           TTCAGAGCCAATTGGACACCCCGTGAGAGCATGCTGCTAGCCCTGGCTTCGTGTCTTCTG 3768
ARD158084        CTCAGAGCCAATTGGACACCCCGTGAGAGCATGCTGCTAGCCCTGGCTTCGTGTCTTCTG 3780
ArB1362          TTCAGAGCCAATTGGACACCCCGTGAGAGCATGCTGCTAGCCCTGGCTTCGTGTCTTTTG 3780
ARB13565         TTCAGAGCTAATTGGACACCTCGTGAGAGCATGCTGCTAGCCCTGGCTTCGTGTCTTTTG 3780
ARB7701          TTCAGAGCTAATTGGACACCTCGTGAGAGCATGCTGCTAGCCCTGGCTTCGTGTCTTTTG 3780
ARB15076         TTCAGAGCTAATTGGACACCTCGTGAGAGCATGCTGCTAGCCCTGGCTTCGTGTCTTTTA 3762
                  ******* ** ******** ***** ******** ** *** **** *****  *  *

P6-740           CAAACTGCGATCTCCGCCCTGGAAGGCGACCTGATGGTTCTCATCAATGGTTTTGCTTTG 3840
CPC0740          CAAACTGCGATCTCCGCCTTGGAAGGCGACCTGATGGTTCTCATCAATGGTTTTGCTTTG 3840
YAP2007          CAAACTGCGATCTCCGCCTTGGAAGGCGACCTGATGGTTCTCATCAATGGTTTTGCTTTG 3840
FSS13025         CAAACTGCGATCTCCGCCTTGGAAGGCGACCTGATGGTTCCCATCAATGGTTTTGCTTTG 3840
PLCal_ZV         CAAACTGCGATCTCCGCCTTGGAAGGCGACCTGATGGTTCTCATCAATGGTTTTGCTTTG 3729
SV0127/14        CAAACTGCGATCTCCGCCTTGGAAGGCGACCTGATGGTTCTCATCAATGGTTTTGCTTTG 3840
8375             CAAACTGCGATCTCCGCCTTGGAAGGCGACCTGATGGTTCTCATCAATGGTTTTGCTTTG 3840
103344           CAAACTGCGATCTCCGCCTTGGAAGGCGACCTGATGGTTCTCATCAATGGTTTTGCTTTG 3840
BrasilZKV2015    CAAACTGCGATCTCCGCCTTGGAAGGCGACCTGATGGTTCTCATCAATGGTTTTGCTTTG 3840
GD01             CAAACTGCGATCTCCGCCTTGGAAGGCGACCTGATGGTTCTCATCAATGGTTTTGCTTTG 3840
Martinique       CAAACTGCGATCTCTGCCTTGGAAGGCGACCTGATGGTTCTCATCAATGGTTTTGCTTTG 3840
NatalRGN         CAAACTGCGATCTCCGCCTTGGAAGGCGACCTGATGGTTCTCATCAATGGTTTTGCTTTG 3840
HPF2013          CAAACTGCGATCTCCGCCTTGGAAGGCGACCTGATGGTTCTCATCAATGGTTTTGCTTTG 3840
SPH2015          CAAACTGCGATCTCCGCCTTGGAAGGCGACCTGATGGTTCTCATCAATGGTTTTGCTTTG 3840
Haiti2014        CAAACTGCGATCTCCGCCTTGGAAGGCGACCTGATGGTTCTCATCAATGGTTTTGCTTTG 3840
PRVABC59         CAAACTGCGATCTCCGCCTTGGAAGGCGACCTGATGGTTCTCATCAATGGTTTTGCTTTG 3840
BeH819015        CAAACTGCGATCTCCGCCTTGGAAGGCGACCTGATGGTTCTCATCAATGGTTTTGCTTTG 3840
Z1106033         CAAACTGCGATCTCCGCCTTGGAAGGCGACCTGATGGTTCTCATCAATGGTTTTGCTTTG 3840
BEH819966        CAAACTGCGATCTCCGCCTTGGAAGGCGACCTGATGGTTCTCATCAATGGTTTTGCTTTG 3840
SSABr            CAAACTGCGATCTCCGCCTTGGAAGGCGACCTGATGGTTCTCATCAATGGTTTTGCTTTG 3840
Beh815744        CAAACTGCGATCTCCGCCTTGGAAGGCGACCTGATGGTTCTCATCAATGGTTTTGCTTTG 3840
BEH818995        CAAACTGCGATCTCCGCCTTGGAAGGCGACCTGATGGTTCTCATCAATGGTTTTGCTTTG 3840
IbH30656         CAGACTGCGATTTCCGCTCTTGAAGGCGAGCTGATGGTCCTCGTTAATGGATTTGCTTTG 3822
ArD128000        CAGACTGCGATCTCCGCTCTTGAAGGCGAGCTGATGGTCCTCGTTAATGGATTTGCTTTG 3840
ArD7117          CAGACTGCGATCTCCGCTCTTGAAGGCGAGCTGATGGTCCTCGTTAATGGATTTGCTTTG 3840
ARD_41519        CAGACTGCGATCTCTGCTCTTGAAGGCGAGCTGATGGTCCTCGTTAATGGATTTGCTTTG 3840
ARD157995        CAAACTGCGATCTCTGCTCTTGAAGGTGACTTGATGGTCCTCGTTAATGGATTTGCTTTG 3840
MR_766           CAAACTGCGATCTCTGCTCTTGAAGGTGACTTGATGGTCCTCATTAATGGATTTGCTTTG 3828
ARD158084        CAAACTGCGATCTCTGCTCTTGAAGGTGACTTGATGGTCCTCGTTAATGGATTTGCTTTG 3840
ArB1362          CAAACTGCAATTTCAGCTCTTGAAGGTGACTTGATGGTCCTCGTTAATGGATTTGCTTTG 3840
ARB13565         CAAACTGCAATCTCTGCTCTCGAAGGTGACTTGATGGTCCTCGTCAATGGATTTGCTTTG 3840
ARB7701          CAAACTGCAATCTCTGCTCTCGAAGGTGACTTGATGGTCCTCGTCAATGGATTTGCTTTG 3840
ARB15076         CAAACTGCAATCTCTGCTCTTGAAGGTGACTTGATGGTCCTCGTCAATGGATTTGCTTTG 3822
                 ** ***** ** ** **  * ***** **  ******* * * * ***** *********
```

```
P6-740             GCCTGGTTGGCAATACGAGCGATGGCTGTTCCACGCACTGACAACATCACCTTGGCAATC 3900
CPC0740            GCCTGGTTGGCAATACGAGCGATGGTTGTTCCACGCACTGACAACATCACCTTGGCAATC 3900
YAP2007            GCCTGGTTGGCAATACGAGCGATGGTTGTTCCACGCACTGACAACATCACCTTGGCAATC 3900
FSS13025           GCCTGGTTGGCAATACGAGCGATGGTTGTTCCACGCACTGACAACATCACCTTGGCAATC 3900
PLCal_ZV           GCCTGGTTGGCAATACGAGCGATGGTTGTTCCACGCACTGACAACATCACCTTGGCAATC 3789
SV0127/14          GCCTGGTTGGCAATACGAGCGATGGTTGTTCCACGCACTGACAATATCACCTTGGCAATC 3900
8375               GCCTGGTTGGCAATACGAGCGATGGTTGTTCCACGCACTGATAACATCACCTTGGCAATC 3900
103344             GCCTGGTTGGCAATACGAGCGATGGTTGTTCCACGCACTGATAACATCACCTTGGCAATC 3900
BrasilZKV2015      GCCTGGTTGGCAATACGAGCGATGGTTGTTCCACGCACTGACAACATCACCTTGGCAATC 3900
GD01               GCCTGGTTGGCAGTACGAGCGATGGTTGTTCCACGCACTGATAACATCACCTTAGCAATC 3900
Martinique         GCCTGGTTGGCAATACGAGCGATGGTTGTTCCACGCACTGACAACATCACCTTGGCAATC 3900
NatalRGN           GCCTGGTTGGCAATACGAGCGATGGTTGTTCCACGCACTGATAACATCACCTTGGCAATC 3900
HPF2013            GCCTGGTTGGCAATACGAGCGATGGTTGTTCCACGCACTGATAACATCACCTTGGCAATC 3900
SPH2015            GCCTGGTTGGCAATACGAGCGATGGTTGTTCCACGCACTGATAACATCACCTTGGCAATC 3900
Haiti2014          GCCTGGTTGGCAATACGAGCGATGGTTGTTCCACGCACTGATAACATCACCTTGGCAATC 3900
PRVABC59           GCCTGGTTGGCAATACGAGCGATGGTTGTTCCACGCACTGATAACATCACCTTGGCAATC 3900
BeH819015          GCCTGGTTGGCAATACGAGCGATGGTTGTTCCACGCACTGATAACATCACCTTGGCAATC 3900
Z1106033           GCCTGGTTGGCAATACGAGCGATGGTTGTTCCACGCACTGATAACATCACCTTGGCAATC 3900
BEH819966          GCCTGGTTGGCAATACGAGCGATGGTTGTTCCACGCACTGATAACATCACCTTGGCAATC 3900
SSABr              GCCTGGTTGGCAATACGAGCGATGGTTGTTCCACGCACTGATAACATCACCTTGGCAATC 3900
Beh815744          GCCTGGTTGGCAATACGAGCGATGGTTGTTCCACGCACTGATAACATCACCTTGGCAATC 3900
BEH818995          GCCTGGTTGGCAATACGAGCGATGGTTGTTCCACGCACTGATAACATCACCTTGGCAATC 3900
IbH30656           GCCTGGTTGGCAATACGAGCAATGGCCGTGCCACGCACTGATAACATCGCTCTAGCAATT 3882
ArD128000          GCCTGGTTGGCAATACGAGCAATGGCCGTGCCACGCACTGACAATATCGCTCTAGCAACT 3900
ArD7117            GCCTGGTTGGCAATACGAGCAATGGCCGTGCCACGCACTGACAATATCGCTCTAGCAATT 3900
ARD_41519          GCCTGGTTGGCAATACGAGCAATGGCCGTGCCACGCACTGACAATATCGCTCTGGCAATT 3900
ARD157995          GCCTGGTTGGCAATTCGAGCAATGGCCGTGCCACGCACTGACAACATCGCTCTAGCAATC 3900
MR_766             GCCTGGTTGGCAATTCGAGCAATGGCCGTGCCACGCACTGACAACATCGCTCTACCAATC 3888
ARD158084          GCCTGGTTGGCAATTCGAGCAATGGCCGTGCCACGCACTGACAACATCGCTCTAGCAATC 3900
ArB1362            GCCTGGTTGGCAATACGAGCAATGGCCGTGCCACGCACTGACAACATTGCTCTAGCAATC 3900
ARB13565           GCCTGGTTGGCAATACGAGCAATGGCCGTGCCACGCACTGACAACATTGCTCTGGCAATC 3900
ARB7701            GCCTGGTTGGCAATACGAGCAATGGCCGTGCCACGCACTGACAACATTGCTCTGGCAATC 3900
ARB15076           GCCTGGTTGGCAATACGAGCAATGGCCGTGCCACGCACTGACAACATTGCTCTAGCAGTC 3882
                   ************ * ***** ****  ** *********** ** **  *  *  **

P6-740             CTGGCTGCTCTGACACCACTGGCCCGAGGCACACTGCTTGTAGCGTGGAGAGCAGGCCTT 3960
CPC0740            CTGGCTGCTCTGACACCACTGGCCCGGGGCACACTGCTTGTGGCGTGGAGAGCAGGCCTT 3960
YAP2007            CTGACTGCGCTGACACCACTGGCCCGGGGCACGCTGCTTGTGGCGTGGAGAGCAGGCCTT 3960
FSS13025           CTGGCTGCTCTGACACCACTGGCCCGGGGCACACTGCTTGTGGCGTGGAGAGCAGGCCTT 3960
PLCal_ZV           CTGGCTGCTCTGACACCACTGGCCCGGGGCACACTGCTTGTGGCGTGGAGAGCAGGCCTT 3849
SV0127/14          CTGGCTGCTCTGACACCACTGGCCCGGGGCACACTGCTTGTGGCGTGGAGAGCAGGCCTT 3960
8375               CTGGCTGCTCTGACACCACTGGCCCGGGGCACACTGCTTGTGGCGTGGAGAGCAGGCCTT 3960
103344             CTGGCTGCTCTGACACCACTGGCCCGGGGCACACTGCTTGTGGCGTGGAGAGCAGGCCTT 3960
BrasilZKV2015      CTGGCTGCTCTGACACCACTGGCCCGGGGCACACTGCTTGTGGCGTGGAGAGCAGGCCTT 3960
GD01               CTGGCTGCTCTGACACCACTGGCCCGGGGCACACTGCTTGTGGCGTGGAGAGCAGGCCTT 3960
Martinique         CTGGCTGCTCTGACACCACTGGCCCGGGGCACACTGCTTGTGGCGTGGAGAGCAGGCCTT 3960
NatalRGN           CTGGCTGCTCTGACACCACTGGCCCGGGGCACACTGCTTGTGGCGTGGAGAGCAGGCCTT 3960
HPF2013            CTGGCTGCTCTGACACCACTGGCCCGGGGCACACTGCTTGTGGCGTGGAGAGCAGGCCTT 3960
SPH2015            CTGGCTGCTCTGACACCACTGGCCCGGGGCACACTGCTTGTGGCGTGGAGAGCAGGCCTT 3960
Haiti2014          CTGGCTGCTCTGACACCACTGGCCCGGGGCACACTGCTTGTGGCGTGGAGAGCAGGCCTT 3960
PRVABC59           CTGGCTGCTCTGACACCACTGGCCCGGGGCACACTGCTTGTGGCGTGGAGAGCAGGCCTT 3960
BeH819015          CTGGCTGCTCTGACACCACTGGCCCGGGGCACACTGCTTGTGGCGTGGAGAGCAGGCCTT 3960
Z1106033           CTGGCTGCTCTGACACCACTGGCCCGGGGCACACTGCTTGTGGCGTGGAGAGCAGGCCTT 3960
BEH819966          CTGGCTGCTCTGACACCACTGGCCCGGGGCACACTGCTTGTGGCGTGGAGAGCAGGCCTT 3960
SSABr              CTGGCTGCTCTGACACCACTGGCCCGGGGCACACTGCTTGTGGCGTGGAGAGCAGGCCTT 3960
Beh815744          CTGGCTGCTCTGACACCACTGGCCCGGGGCACACTGCTTGTGGCGTGGAGAGCAGGCCTT 3960
BEH818995          CTGGCTGCTCTGACACCACTGGCCCGGGGCACACTGCTTGTGGCGTGGAGAGCAGGCCTT 3960
IbH30656           CTGGCCGCTCTAACACCATTAGCCAGAGGCACACTGCTTGTGGCATGGAGAGCGGGCCTC 3942
ArD128000          CTGGCCGCTTTAACACCATTGGCCAGAGGCACACTGCTCGTGGCATGGAGAGCGGGCCTC 3960
ArD7117            CTGGCTGCTCTAACACCATTAGCCAGAGGCACACTGCTCGTGGCATGGAGAGCGGGCCTC 3960
ARD_41519          CTGGCTGCTCTAACACCATTAGCCAGAGGCACACTGCTCGTGGCATGGAGAGCGGGCCTC 3960
ARD157995          TTGGCTGCTCTAACACCACTAGCTCGAGGCACACTGCTCGTGGCATGGAGAGCGGGCCTG 3960
MR_766             TTGGCTGCTCTAACACCACTAGCTCGAGGCACACTGCTCGTGGCATGGAGAGCGGGCCTG 3948
ARD158084          TTGGCTGCTCTAACACCACTAGCTCGAGGCACACTGCTCGTGGCATGGAGAGCGGGCCTG 3960
ArB1362            CTGGCTGCTCTGACACCACTAGCCCGAGGCACACTGCTCGTGGCATGGAGAGCGGGCCTC 3960
ARB13565           CTGGCTGCTCTGACACCACTAGCCCGAGGTACACTGCTCGTGGCATGGAGAGCGGGCCTC 3960
ARB7701            CTGGCTGCTCTGACACCACTAGCCCGAGGTACACTGCTCGTGGCATGGAGAGCGGGCCTC 3960
```

```
ARB15076           CTGGCTGCTCTGACACCACTAGCCCGAGGCACACTGCTCGTGGCATGGAGAGCGGGCCTC 3942
                    ** * **  * ****** * **  * ** ** ***** ** ** ******** *****

P6-740             GCTACTTGTGGGGGGTTCATGCTCCTCTCTCTGAAGGGGAAAGGTAGTGTGAAGAAGAAC 4020
CPC0740            GCTACTTGCGGGGGGTTCATGCTCCTCTCTCTGAAGGGGAAAGGCAGTGTGAAGAAGAAC 4020
YAP2007            GCTACTTGCGGGGGGTTCATGCTTCTCTCTCTGAAGGGGAAGGGCAGTGTGAAGAAGAAC 4020
FSS13025           GCTACTTGCGGGGGGTTCATGCTCCTTTCTCTGAAGGGGAAAGGCAGTGTGAAGAAGAAC 4020
PLCal_ZV           GCTACTTGTGGGGGGTTCATGCTCCTCTCTCTGAAGGGGAAAGGCAGTGTGAAGAAGAAC 3909
SV0127/14          GCTACTTGCGGGGGGTTCATGCTCCTCTCTCTGAAGGGGAAAGGCAGTGTGAAGAAGAAC 4020
8375               GCTACTTGCGGGGGGTTTATGCTCCTCTCTCTGAAGGGAAAAGGCAGTGTGAAGAAGAAC 4020
103344             GCTACTTGCGGGGGGTTTATGCTCCTCTCTCTGAAGGGAAAAGGCAGTGTGAAGAAGAAC 4020
BrasilZKV2015      GCTACTTGCGGGGGGTTTATGCTCCTCTCTCTGAAGGGAAAAGGCAGTGTGAAGAAGAAC 4020
GD01               GCTACTTGCGGGGGGTTTATGCTCCTCTCTCTGAAGGGAAAAGGCAGTGTGAAGAAGAAC 4020
Martinique         GCTACTTGCGGGGGGTTTATGCTCCTCTCTCTGAAGGGAAAAGGCAGTGTGAAGAAGAAC 4020
NatalRGN           GCTACTTGCGGGGGGTTTATGCTCCTCTCTCTGAAGGGAAAAGGCAGTGTGAAGAAGAAC 4020
HPF2013            GCTACTTGCGGGGGGTTTATGCTCCTCTCTCTGAAGGGAAAAGGCAGTGTGAAGAAGAAC 4020
SPH2015            GCTACTTGCGGGGGGTTTATGCTCCTCTCTCTGAAGGGAAAAGGCAGTGTGAAGAAGAAC 4020
Haiti2014          GCTACTTGCGGGGGGTTTATGCTCCTCTCTCTGAAGGGAAAAGGCAGTGTGAAGAAGAAC 4020
PRVABC59           GCTACTTGCGGGGGGTTTATGCTCCTCTCTCTGAAGGGAAAAGGCAGTGTGAAGAAGAAC 4020
BeH819015          GCTACTTGCGGGGGGTTTATGCTCCTCTCTCTGAAGGGAAAAGGCAGTGTGAAGAAGAAC 4020
Z1106033           GCTACTTGCGGGGGGTTTATGCTCCTCTCTCTGAAGGGAAAAGGCAGTGTGAAGAAGAAC 4020
BEH819966          GCTACTTGCGGGGGGTTTATGCTCCTCTCTCTGAAGGGAAAAGGCAGTGTGAAGAAGAAC 4020
SSABr              GCTACTTGCGGGGGGTTTATGCTCCTCTCTCTGAAGGGAAAAGGCAGTGTGAAGAAGAAC 4020
Beh815744          GCTACTTGCGGGGGGTTTATGCTCCTCTCTCTGAAGGGAAAAGGCAGTGTGAAGAAGAAC 4020
BEH818995          GCTACTTGCGGGGGGTTTATGCTCCTCTCTCTGAAGGGAAAAGGCAGTGTGAAGAAGAAC 4020
IbH30656           GCCACTTGTGGAGGGTTCATGCTCATTTCCCTGAAAGGGAAAGGTAGTGTGAAGAAGAAC 4002
ArD128000          GCCACTTGTGGAGGGTTCATGCTTCTTTCCCTGAAAGGGAAAGGTAGTGTGAAGAAGAAC 4020
ArD7117            GCCACTTGTGGAGGGTTCATGCTCCTTTCCCTGAAAGGGAAAGGTAGTGTGAAGAAGAAC 4020
ARD_41519          GCCACTTGTGGAGGGTTCATGCTCCTTTCCCTGAAAGGGAAAGGTAGTGTGAAGAAGAAC 4020
ARD157995          GCTACTTGTGGAGGGTTCATGCTCCTCTCCCTGAAAGGGAAAGGTAGTGTGAAGAAGAAC 4020
MR_766             GCTACTTGTGGAGGGATCATGCTCCTCTCCCTGAAAGGGAAAGGTAGTGTGAAGAAGAAC 4008
ARD158084          GCTACTTGTGGAGGGTTCATGCTCCTCTCCCTGAAAGGGAAAGGTAGTGTGAAGAAGAAC 4020
ArB1362            GCCACTTGTGGAGGGTTCATGCTCCTCTCCTTGAAAGGAAAAGGTAGTGTGAAGAAGAAC 4020
ARB13565           GCTACTTGTGGAGGGTTTATGCTCCTCTCCCTGAAAGGGAAAGGTAGTGTGAAGAAGAAC 4020
ARB7701            GCTACTTGTGGAGGGTTTATGCTCCTCTCCCTGAAAGGGAAAGGTAGTGTGAAGAAGAAC 4020
ARB15076           GCTACTTGTGGAGGGTTCATGCTCCTTTCCCTGAAGGGGAAAGGTAGTGTGAAGAAGAAC 4002
                   ** ***** ** *** * *****  * **  **** ** ** ** ***************

P6-740             CTACCATTTGTCATGGCCTTGGGACTAACCGCTGTGAGGCTGGTTGACCCCATCAACGTG 4080
CPC0740            CTACCATTTGTCATGGCCTTGGGACTAACTGCTGTGAGGCTGGTCGACCCCATCAACGTG 4080
YAP2007            CTACCATTTGTCATGGCCTTGGGACTCACCGCTGTGAGGCTGGTCGACCCCATCAACGTG 4080
FSS13025           TTACCATTTGTCATGGCCCTGGGACTAACCGCTGTGAGGCTGGTCGACCCCATCAACGTG 4080
PLCal_ZV           TTACCATTTGTCATGGCCCTGGGACTAACCGCTGTGAGGCTGGTCGACCCCATCAACGTG 3969
SV0127/14          TTACCATTTGTCATGGCCCTGGGACTAACCGCTGTGAGGCTGGTCGACCCCATCAACGTG 4080
8375               TTACCATTTGTCATGGCCCTGGGACTAACCGCTGTGAGGCTGGTCGACCCCATCAACGTG 4080
103344             TTACCATTTGTCATGGCCCTGGGACTAACCGCTGTGAGGCTGGTCGACCCCATCAACGTG 4080
BrasilZKV2015      TTACCATTTGTCATGGCCCTGGGACTAACCGCTGTGAGGCTGGTCGACCCCATCAACGTG 4080
GD01               TTACCATTTGTCATGGCCCTGGGACTAACCGCTGTGAGGCTGGTCGACCCCATCAACGTG 4080
Martinique         TTACCATTTGTCATGGCCCTGGGACTAACCGCTGTGAGGCTGGTCGACCCCATCAACGTG 4080
NatalRGN           TTACCATTTGTCATGGCCCTGGGACTAACCGCTGTGAGGCTGGTCGACCCCATCAACGTG 4080
HPF2013            TTACCATTTGTCATGGCCCTGGGACTAACCGCTGTGAGGCTGGTCGACCCCATCAACGTG 4080
SPH2015            TTACCATTTGTCATGGCCCTGGGACTAACCGCTGTGAGGCTGGTCGACCCCATCAACGTG 4080
Haiti2014          TTACCATTTGTCATGGCCCTGGGACTAACCGCTGTGAGGCTGGTCGACCCCATCAACGTG 4080
PRVABC59           TTACCATTTGTCATGGCCCTGGGACTAACCGCTGTGAGGCTGGTCGACCCCATCAACGTG 4080
BeH819015          TTACCATTTGTCATGGCCCTGGGACTAACCGCTGTGAGGCTGGTCGACCCCATCAACGTG 4080
Z1106033           TTACCATTTGTCATGGCCCTGGGACTAACCGCTGTGAGGCTGGTCGACCCCATCAACGTG 4080
BEH819966          TTACCATTTGTCATGGCCCTGGGACTAACCGCTGTGAGGCTGGTCGACCCCATCAACGTG 4080
SSABr              TTACCATTTGTCATGGCCCTGGGACTAACCGCTGTGAGGCTGGTCGACCCCATCAACGTG 4080
Beh815744          TTACCATTTGTCATGGCCCTGGGACTAACCGCTGTGAGGCTGGTCGACCCCATCAACGTG 4080
BEH818995          TTACCATTTGTCATGGCCCTGGGACTAACCGCTGTGAGGCTGGTCGACCCCATCAACGTG 4080
IbH30656           CTGCCATTTGTCATGGCCTTGGGGTTGACCGCTGTGAGGATAGTGGACCCCATTAATGTG 4062
ArD128000          CTGCCTTTTGTCATGGCCTTGGGGTTGGCCGCTGTGAGGATAGTGGACCCCATCAATGTG 4080
ArD7117            CTGCCTTTTGTCATGGCCTTGGGGTTGACCGCTGTGAGGATAGTGGACCCCATCAATGTG 4080
ARD_41519          CTGCCTTTTGTCATGGCCTTGGGGTTGACCGCTGTGAGGATAGTGGACCCCATCAATGTG 4080
ARD157995          CTGCCATTTGTCATGGCCCTGGGATTGACAGCTGTGAGGGTAGTAGACCCTATTAATGTG 4080
MR_766             CTGCCATTTGTCATGGCCCTGGGATTGACAGCTGTGAGGGTAGTAGACCCTATTAATGTG 4068
ARD158084          CTGCCATTTGTCATGGCCCTGGGATTGACAGCTGTGAGGGTAGTAGACCCTATTAATGTG 4080
ArB1362            CTGCCATTTGTCATGGCCTTGGGAATGACCGCTGTGAGGATAGTGGACCCCATTAACGTG 4080
```

```
ARB13565        CTGCCATTTGTCATGGCCTTGGGATTGACTGCTGTGAGGATAGTAGACCCCATTAATGTG 4080
ARB7701         CTGCCATTTGTCATGGCCTTGGGATTGACTGCTGTGAGGATAGTAGACCCCATTAATGTG 4080
ARB15076        CTGCCATTTGTCATGGCCTTGGGATTGACCGCTGTGAGGATAGTAGACCCCATTAATGTG 4062
                 * ** ************ ****  *  * ********* * ** ***** ** ** ***

P6-740          GTGGGACTGCTGTTGCTCACAAGGAGTGGGAAGCGGAGCTGGCCCCCTAGTGAAGTACTC 4140
CPC0740         GTGGGACTGCTGTTGCTCACAAGGAGTGGGAAGCGGAGCTGGCCCCCTAGTGAAGTACTC 4140
YAP2007         GTGGGACTGCTGTTGCTCACAAGGAGTGGGAAGCGGAGCTGGCCCCCTAGTGAAGTACTC 4140
FSS13025        GTGGGACTGCTGTTGCTCACAAGGAGTGGGAAGCGGAGCTGGCCCCCTAGTGAAGTACTC 4140
PLCal_ZV        GTGGGACTGCTGTTGCTCACAAGGAGTGGGAAGCGGAGCTGGCCCCCTAGTGAAGTGCTC 4029
SV0127/14       GTGGGACTGCTGTTGCTCACAAGGAGTGGGAAGCGGAGCTGGCCCCCTAGCGAAGTACTC 4140
8375            GTGGGACTGCTGTTGCTCACAAGGAGTGGGAAGCGGAGCTGGCCCCCTAGCGAAGTACTC 4140
103344          GTGGGACTGCTGTTGCTCACAAGGAGTGGGAAGCGGAGCTGGCCCCCTAGCGAAGTACTC 4140
BrasilZKV2015   GTGGGACTGCTGTTGCTCACAAGGAGTGGGAAGCGGAGCTGGCCCCCTAGCGAAGTACTC 4140
GD01            GTGGGACTGCTGTTGCTCACAAGGAGTGGGAAGCGGAGCTGGCCCCCTAGCGAAGTACTC 4140
Martinique      GTGGGACTGCTGTTGCTCACAAGGAGTGGGAAGCGGAGCTGGCCCCCTAGCGAAGTACTC 4140
NatalRGN        GTGGGACTGCTGTTGCTCACAAGGAGTGGGAAGCGGAGCTGGCCCCCTAGCGAAGTACTC 4140
HPF2013         GTGGGACTGCTGTTGCTCACAAGGAGTGGGAAGCGGAGCTGGCCCCCTAGCGAAGTACTC 4140
SPH2015         GTGGGGCTGCTGTTGCTCACAAGGAGTGGGAAGCGGAGCTGGCCCCCTAGCGAAGTACTC 4140
Haiti2014       GTGGGGCTGCTGTTGCTCACAAGGAGTGGGAAGCGGAGCTGGCCCCCTAGCGAAGTACTC 4140
PRVABC59        GTGGGACTGCTGTTGCTCACAAGGAGTGGGAAGCGGAGCTGGCCCCCTAGCGAAGTACTC 4140
BeH819015       GTGGGACTGCTGTTACTCACAAGGAGTGGGAAGCGGAGCTGGCCCCCTAGCGAAGTACTC 4140
Z1106033        GTGGGACTGCTGTTGCTCACAAGGAGTGGGAAGCGGAGCTGGCCCCCTAGCGAAGTACTC 4140
BEH819966       GTGGGACTGCTGTTGCTCACAAGGAGTGGGAAGCGGAGCTGGCCCCCTAGCGAAGTACTC 4140
SSABr           GTGGGACTGCTGTTGCTCACAAGGAGTGGGAAGCGGAGCTGGCCCCCTAGCGAAGTACTC 4140
Beh815744       GTGGGACTGCTGTTGCTCACAAGGAGTGGGAAGCGGAGCTGGCCCCCTAGCGAAGTACTC 4140
BEH818995       GTGGGACTGCTGTTGCTCACAAGGAGTGGGAAGCGGAGCTGGCCCCCTAGCGAAGTACTC 4140
IbH30656        GTAGGACTACTGTTACTGACAAGGAGTGGGAAACGGAGCTGGCCCCCTAGTGAAGTGCTT 4122
ArD128000       GTAGGACTACTGTTACTCACAAGGAGTGGAAAACGGAGCTGGCCCCCCAGTGAAGTGCTT 4140
ArD7117         GTAGGACTACTGTTACTCACACGGAGTGGAAAACGGAGCTGGCCCCCTAGTGAAGTGCTT 4140
ARD_41519       GTAGGACTACTGTTACTCACAAGGAGTGGAAAACGGAGCTGGCCTCCTAGTGAAGTGCTT 4140
ARD157995       GTAGGACTACTGTTACTCACAAGGAGTGGGAAGCGGAGCTGGCCCCCTAGTGAAGTTCTC 4140
MR_766          GTAGGACTACTGTTACTCACAAGGAGTGGGAAGCGGAGCTGGCCCCCTAGTGAAGTTCTC 4128
ARD158084       GTAGGACTACTGTTACTCACAAGGAGTGGGAAGCGGAGCTGGCCCCCTAGTGAAGTTCTC 4140
ArB1362         GTGGGACTACTACTACTCACAAGGAGTGGGAAGCGGAGCTGGCCCCCCAGTGAAGTGCTT 4140
ARB13565        GTGGGACTACTGTTACTCACAAGGAGTGGGAAGCGGAGCTGGCCCCCTAGTGAAGTGCTT 4140
ARB7701         GTGGGACTACTGTTACTCACAAGGAGTGGGAAGCGGAGCTGGCCCCCTAGTGAAGTGCTT 4140
ARB15076        GTAGGACTACTGTTACTCACAAGGAGCGGGAAGCGGAGCTGGCCCCCTAGTGAAGTGCTT 4122
                ** ** ** **  * ** *** **** ** ** *********** ** ** ***** **

P6-740          ACAGCTGTTGGCCTGATATGTGCACTGGCCGGAGGGTTCGCCAAAGCAGATATAGAGATG 4200
CPC0740         ACAGCTGTTGGCCTGATATGCGCATTGGCTGGAGGGTTCGCCAAGGCGGATATAGAGATG 4200
YAP2007         ACAGCTGTTGGTCTGATATGCGCGTTGGCCGGAGGGTTCGCCAAGGCGGATATAGAGATG 4200
FSS13025        ACAGCTGTTGGCCTGATATGCGCATTGGCTGGAGGGTTCGCCAAGGCGGATATAGAGATG 4200
PLCal_ZV        ACAGCTGTTGGCCTGATATGCGCATTGGCTGGAGGGTTCGCCAAGGCGGATATAGAGATG 4089
SV0127/14       ACAGCTGTTGGCCTGATATGCGCATTGGCTGGAGGGTTCGCCAAGGCAGATATAGAGATG 4200
8375            ACAGCTGTTGGCCTGATATGCGCATTGGCTGGAGGGTTCGCCAAGGCAGATATAGAGATG 4200
103344          ACAGCTGTTGGCCTGATATGCGCATTGGCTGGAGGGTTCGCCAAGGCAGATATAGAGATG 4200
BrasilZKV2015   ACAGCTGTTGGCCTGATATGCGCATTGGCTGGAGGGTTCGCCAAGGCAGATATAGAGATG 4200
GD01            ACAGCTGTTGGCCTGATATGCGCATTGGCTGGAGGGTTCGCCAAGGCAGATATAGAGATG 4200
Martinique      ACAGCTGTTGGCCTGATATGTGCATTGGCTGGAGGGTTCGCCAAGGCAGATATAGAGATG 4200
NatalRGN        ACAGCTGTTGGCCTGATATGCGCATTGGCTGGAGGGTTCGCCAAGGCAGATATAGAGATG 4200
HPF2013         ACAGCTGTTGGCCTGATATGCGCATTGGCTGGAGGGTTCGCCAAGGCAGATATAGAGATG 4200
SPH2015         ACAGCTGTTGGCCTGATATGCGCATTGGCTGGAGGGTTCGCCAAGGCAGATATAGAGATG 4200
Haiti2014       ACAGCTGTTGGCCTGATATGCGCATTGGCTGGAGGGTTCGCCAAGGCAGATATAGAGATG 4200
PRVABC59        ACAGCTGTTGGCCTGATATGCGCATTGGCTGGAGGGTTCGCCAAGGCAGATATAGAGATG 4200
BeH819015       ACAGCTGTTGGCCTGATATGCGCATTGGCTGGAGGGTTCGCCAAGGCAGATATAGAGATG 4200
Z1106033        ACAGCTGTTGGCCTGATATGCGCATTGGCTGGAGGGTTCGCCAAGGCAGATATAGAGATG 4200
BEH819966       ACAGCTGTTGGCCTGATATGCGCATTGGCTGGAGGGTTCGCCAAGGCAGATATAGAGATG 4200
SSABr           ACAGCTGTTGGCCTGATATGCGCATTGGCTGGAGGGTTCGCCAAGGCAGATATAGAGATG 4200
Beh815744       ACAGCTGTTGGCCTGATATGCGCATTGGCTGGAGGGTTCGCCAAGGCAGATATAGAGATG 4200
BEH818995       ACAGCTGTTGGCCTGATATGCGCATTGGCTGGAGGGTTCGCCAAGGCAGATATAGAGATG 4200
IbH30656        ACAGCTGTCGGCCTGATATGTGCACTGGCCGGAGGGTTTGCCAAGGCAGACATAGAGATG 4182
ArD128000       ACAGCTGTTGGCCTGATATGTGCACTGGCCGGAGGGTTTGCCAAGGCAGACATAGAGATG 4200
ArD7117         ACAGCTGTTGGCCTGATATGTGCACTGGCCGGAGGGTTTGCCAAGGCAGATATAGAGATG 4200
ARD_41519       ACAGCTGTTGGCCTGATATGTGCACTGGCCGGAGGGTTTGCCAAGGCAGACATAGAGATG 4200
ARD157995       ACAGCCGTTGGCCTGATATGTGCACTGGCCGGAGGGTTTGCCAAGGCAGACATTGAGATG 4200
MR_766          ACAGCCGTTGGCCTGATATGTGCACTGGCCGGAGGGTTTGCCAAGGCAGACATTGAGATG 4188
```

```
ARD158084        ACAGCCGTTGGCCTGATATGTGCACTGGCCGGAGGGTTTGCCAAGGCAGACATTGAGATG 4200
ArB1362          ACAGCTGTTGGCCTGATATGTGCACTGGCCGGAGGGTTTGCCAAGGCAGACATTGAGATG 4200
ARB13565         ACAGCTGTTGGCCTGATATGTGCACTGGCCGGAGGCTTTGCCAAGGCAGACATTGAGATG 4200
ARB7701          ACAGCTGTTGGCCTGATATGTGCACTGGCCGGAGGCTTTGCCAAGGCAGACATTGAGATG 4200
ARB15076         ACAGCTGTTGGCCTGATATGTGCACTGGCCGGAGGGTTTGCCAAGGCAGACATTGAGATG 4182
                 ***** ** ** ******** **  **** ***** ** ***** ** ** ** ******

P6-740           GCTGGGCCCATGGCTGCAGTTGGCCTGCTAATTGTTAGTTACGTGGTCTCAGGAAAGAGT 4260
CPC0740          GCTGGGCCCATGGCCGCGGTCGGTCTGCTAATTGTCAGTTACGTGGTCTCAGGAAAGAGT 4260
YAP2007          GCTGGGCCCATGGCCGCGGTCGGTCTGCTAATTGTCAGTTACGTGGTCTCAGGAAAGAGT 4260
FSS13025         GCTGGGCCCATGGCCGCGGTCGGTCTGCTAATTGTCAGTTACGTGGTCTCAGGAAAGAGT 4260
PLCal_ZV         GCTGGGCCCATGGCCGCGGTCGGTCTGCTAATTGTCAGTTACGTGGTCTCAGGAAAGAGT 4149
SV0127/14        GCTGGGCCCATGGCCGCGGTCGGTCTGCTAATTGTCAGTTACGTGGTCTCAGGAAAGAGT 4260
8375             GCTGGGCCCATGGCCGCGGTCGGTCTGCTAATTGTCAGTTACGTGGTCTCAGGAAAGAGT 4260
103344           GCTGGGCCCATGGCCGCGGTCGGTCTGCTAATTGTCAGTTACGTGGTCTCAGGAAAGAGT 4260
BrasilZKV2015    GCTGGGCCCATGGCCGCGGTCGGTCTGCTAATTGTCAGTTACGTGGTCTCAGGAAAGAGT 4260
GD01             GCTGGGCCCATGGCCGCGGTCGGTCTGCTAATTGTCAGTTACGTGGTCTCAGGAAAGAGT 4260
Martinique       GCTGGGCCCATGGCCGCGGTCGGTCTGCTAATTGTCAGTTACGTGGTCTCAGGAAAGAGT 4260
NatalRGN         GCTGGGCCCATGGCCGCGGTCGGTCTGCTAATTGTCAGTTACGTGGTCTCAGGAAAGAGT 4260
HPF2013          GCTGGGCCCATGGCCGCGGTCGGTCTGCTAATTGTCAGTTACGTGGTCTCAGGAAAGAGT 4260
SPH2015          GCTGGGCCCATGGCCGCGGTCGGTCTGCTAATTGTCAGTTACGTGGTCTCAGGAAAGAGT 4260
Haiti2014        GCTGGGCCCATGGCCGCGGTCGGTCTGCTAATTGTCAGTTACGTGGTCTCAGGAAAGAGT 4260
PRVABC59         GCTGGGCCCATGGCCGCGGTCGGTCTGCTAATTGTCAGTTACGTGGTCTCAGGAAAGAGT 4260
BeH819015        GCTGGGCCCATGGCCGCGGTCGGTCTGCTAATTGTCAGTTACGTGGTCTCAGGAAAGAGT 4260
Z1106033         GCTGGGCCCATGGCCGCGGTCGGTCTGCTAATTGTCAGTTACGTGGTCTCAGGAAAGAGT 4260
BEH819966        GCTGGGCCCATGGCCGCGGTCGGTCTGCTAATTGTCAGTTACGTGGTCTCAGGAAAGAGT 4260
SSABr            GCTGGGCCCATGGCCGCGGTCGGTCTGCTAATTGTCAGTTACGTGGTCTCAGGAAAGAGT 4260
Beh815744        GCTGGGCCCATGGCCGCGGTCGGTCTGCTAATTGTCAGTTACGTGGTCTCAGGAAAGAGT 4260
BEH818995        GCTGGGCCCATGGCCGCGGTCGGTCTGCTAATTGTCAGTTACGTGGTCTCAGGAAAGAGT 4260
IbH30656         GCTGGGCCCATGGCTGCAGTAGGCCTGCTAATTGTCAGTTATGTGGTCACGGGAAAGAGT 4242
ArD128000        GCTGGGCCCATGGCTGCAGTAGGCCTGCTAATTGTCAGTTATGTGGTCTCGGGAAAGAGT 4260
ArD7117          GCTGGGCCCATGGCTGCAGTAGGCCTGCTAATTGTCAGTTATGTGGTCTCGGGAAAGAGT 4260
ARD_41519        GCTGGGCCCATGGCTGCAGTAGGCCTGCTAATTGTCAGTTATGTGGTCTCGGGAAAGAGT 4260
ARD157995        GCTGGACCCATGGCTGCAGTAGGCTTGCTAATTGTCAGCTATGTGGTCTCGGGAAAGAGT 4260
MR_766           GCTGGACCCATGGCTGCAGTAGGCTTGCTAATTGTCAGCTATGTGGTCTCGGGAAAGAGT 4248
ARD158084        GCTGGACCCATGGCTGCAGTAGGCTTGCTAATTGTCAGCTATGTGGTCTCGGGAAAGAGT 4260
ArB1362          GCTGGGCCCATGGCTGCAGTAGGCTTGCTAATTGTCAGTTATGTGGTCTCAGGAAAGAGT 4260
ARB13565         GCTGGGCCCATGGCTGCAGTAGGCTTGCTGATTGTCAGTTATGTGGTCTCAGGAAAGAGT 4260
ARB7701          GCTGGGCCCATGGCTGCAGTAGGCTTGCTGATTGTCAGTTATGTGGTCTCAGGAAAGAGT 4260
ARB15076         GCTGGGCCCATGGCTGCAGTAGGCTTGCTAATTGTCAGTTATGTGGTCTCAGGAAAGAGT 4242
                 ***** ******** ** ** **  **** ***** ** ** ****** * *********

P6-740           GTGGACATGTACATTGAAAGAGCAGGTGACATCACATGGGAAAAAGATGCGGAAGTTACT 4320
CPC0740          GTGGACATGTACATTGAAAGAGCAGGTGACATCACATGGGAAAAAGATGCGGAAATCACT 4320
YAP2007          GTGGACATGTACATTGAAAGAGCAGGTGACATCACATGGGAAAAAGATGCGGAAGTCACT 4320
FSS13025         GTGGACATGTACATTGAAAGAGCAGGTGACATCACATGGGAAAAAGATGCGGAAGTCACT 4320
PLCal_ZV         GTGGACATGTACATTGAAAGAGCAGGTGACATCACGTGGGAAAAAGATGCGGAAGTCACT 4209
SV0127/14        GTGGACATGTACATTGAAAGAGCAGGTGACATCACATGGGAAAAAGATGCGGAAGTTACT 4320
8375             GTGGACATGTACATTGAAAGAGCAGGTGACATCACATGGGAAAAAGATGCGGAAGTCACT 4320
103344           GTGGACATGTACATTGAAAGAGCAGGTGACATCACATGGGAAAAAGATGCGGAAGTCACT 4320
BrasilZKV2015    GTGGACATGTACATTGAAAGAGCAGGTGACATCACATGGGAAAAAGATGCGGAAGTCACT 4320
GD01             GTGGACATGTACATTGAAAGAGCAGGTGACATCACATGGGAAAAAGATGCGGAAGTCACT 4320
Martinique       GTGGACATGTACATTGAAAGAGCAGGTGACATCACATGGGAAAAAGATGCGGAAGTCACT 4320
NatalRGN         GTGGACATGTACATTGAAAGAGCAGGTGACATCACATGGGAAAAAGATGCGGAAGTCACT 4320
HPF2013          GTGGACATGTACATTGAAAGAGCAGGTGACATCACATGGGAAAAAGATGCGGAAGTCACT 4320
SPH2015          GTGGACATGTACATTGAAAGAGCAGGTGACATCACATGGGAAAAAGATGCGGAAGTCACT 4320
Haiti2014        GTGGACATGTACATTGAAAGAGCAGGTGACATCACATGGGAAAAAGATGCGGAAGTCACT 4320
PRVABC59         GTGGACATGTACATTGAAAGAGCAGGTGACATCACATGGGAAAAAGATGCGGAAGTCACT 4320
BeH819015        GTGGACATGTACATTGAAAGAGCAGGTGACATCACATGGGAAAAAGATGCGGAAGTCACT 4320
Z1106033         GTGGACATGTACATTGAAAGAGCAGGTGACATCACATGGGAAAAAGATGCGGAAGTCACT 4320
BEH819966        GTGGACATGTACATTGAAAGAGCAGGTGACATCACATGGGAAAAAGATGCGGAAGTCACT 4320
SSABr            GTGGACATGTACATTGAAAGAGCAGGTGACATCACATGGGAAAAAGATGCGGAAGTCACT 4320
Beh815744        GTGGACATGTACATTGAAAGAGCAGGTGACATCACATGGGAAAAAGATGCGGAAGTCACT 4320
BEH818995        GTGGACATGTACATTGAAAGAGCAGGTGACATCACATGGGAAAAAGATGCGGAAGTCACT 4320
IbH30656         GTGGACATGTACATTGAAAGAGCAGGTGATATTACATGGGAAAAAGACGCGGAAGTCACT 4302
ArD128000        GTGGACATGTACATCGAAAGAGCAGGTGATATCACATGGGAAAAAGACGCGGAAGTCACT 4320
ArD7117          GTGGACATGTACATCGAAAGAGCAGGTGATATCACATGGGAAAAAGACgCGGAAGTCACT 4320
ARD_41519        GTGGACATGTACATCGAAAGAGCAGGTGATATCACATGGGAAAAAGACGCGGAAGTCACT 4320
```

```
ARD157995           GTGGACATGTACATTGAAAGAGCAGGTGACATCACATGGGAAAAGGACGCGGAAGTCACT 4320
MR_766              GTGGACATGTACATTGAAAGAGCAGGTGACATCACATGGGAAAAGGACGCGGAAGTCACT 4308
ARD158084           GTGGACATGTACATTGAAAGAGCAGGTGACATCACATGGGAAAAGGACGCGGAAGTCACT 4320
ArB1362             GTGGATATGTACATTGAAAGAGCAGGTGACATCACATGGGAAAAAGACGCGGAAGTTACT 4320
ARB13565            GTGGATATGTACATTGAAAGAGCAGGTGACATCACATGGGAAAAGGACGCGGAAGTTACT 4320
ARB7701             GTGGATATGTACATTGAAAGAGCAGGTGACATCACATGGGAAAAGGACGCGGAAGTTACT 4320
ARB15076            GTGGATATGTACATTGAAAGAGCAGGTGACATCACATGGGAAAAGGACGCGGAAGTTACT 4302
                    ***** ******** ************** ** ** ******** ** ****** * ***

P6-740              GGAAACAGCCCCCGGCTCGATGTGGCACTAGATGAGAGTGGTGATTTCTCCCTGGTGGAG 4380
CPC0740             GGAAACAGTCCCCGGCTCGATGTGGCACTAGATGAGAGTGGTGATTTCTCCCTAGTGGAG 4380
YAP2007             GGAAACAGTCCCCGGCTCGATGTGGCACTGGATGAGAGTGGTGATTTCTCCCTAGTGGAG 4380
FSS13025            GGAAACAGTCCCCGGCTCGATGTGGCACTAGATGAGAGTGGTGATTTCTCCCTAGTGGAG 4380
PLCal_ZV            GGAAACAGTCCCCGGCTCGATGTGGCACTAGATGAGAGTGGTGATTTCTCCCTGGTGGAG 4269
SV0127/14           GGAAACAGTCCCCGGCTCGATGTGGCACTAGATGAGAGTGGTGATTTCTCCCTGGTGGAG 4380
8375                GGAAACAGTCCCCGGCTCGATGTGGCGCTAGATGAGAGTGGTGATTTCTCCCTGGTGGAG 4380
103344              GGAAACAGTCCCCGGCTCGATGTGGCGCTAGATGAGAGTGGTGATTTCTCCCTGGTGGAG 4380
BrasilZKV2015       GGAAACAGTCCCCGGCTCGATGTGGCGCTAGATGAGAGTGGTGACTTCTCCCTGGTGGAG 4380
GD01                GGAAACAGTCCCCGGCTCGATGTGGCGCTAGATGAGAGTGGTGATTTCTCCCTGGTGGAG 4380
Martinique          GGAAACAGTCCCCGGCTCGACGTGGCGCTAGATGAGAGTGGTGATTTCTCCCTGGTGGAG 4380
NatalRGN            GGAAACAGTCCCCGGCTCGATGTGGCGCTAGATGAGAGTGGTGATTTCTCCCTGGTGGAG 4380
HPF2013             GGAAACAGTCCCCGGCTCGATGTGGCGCTAGATGAGAGTGGTGATTTCTCCCTGGTGGAG 4380
SPH2015             GGAAACAGTCCCCGGCTCGATGTGGCGCTAGATGAGAGTGGTGATTTCTCCCTGGTGGAG 4380
Haiti2014           GGAAACAGTCCCCGGCTCGATGTGGCGCTAGATGAGAGTGGTGATTTCTCCCTGGTGGAG 4380
PRVABC59            GGAAACAGTCCCCGGCTCGATGTGGCGCTAGATGAGAGTGGTGATTTCTCCCTGGTGGAG 4380
BeH819015           GGAAACAGTCCCCGGCTCGATGTGGCGCTAGATGAGAGTGGTGATTTCTCCCTGGTGGAG 4380
Z1106033            GGAAACAGTCCCCGGCTCGATGTGGCGCTAGATGAGAGTGGTGATTTCTCCCTGGTGGAG 4380
BEH819966           GGAAACAGTCCCCGGCTCGATGTGGCGCTAGATGAGAGTGGTGATTTCTCCCTGGTGGAG 4380
SSABr               GGAAACAGTCCCCGGCTCGATGTGGCGCTAGATGAGAGTGGTGATTTCTCCCTGGTGGAG 4380
Beh815744           GGAAACAGTCCCCGGCTCGATGTGGCGCTAGATGAGAGTGGTGATTTCTCCCTGGTGGAG 4380
BEH818995           GGAAACAGTCCCCGGCTCGATGTGGCGCTAGATGAGAGTGGTGATTTCTCCCTGGTGGAG 4380
IbH30656            GGAAACAGTCCTCGGCTTGACGTGGCACTAGATGAGAGTGGTGATTTCTCTTTGGTAGAG 4362
ArD128000           GGAAACAGTCCCCGGCTTGACGTGGCACTGGATGAGAGTGGTGATTTCTCCTTGGTAGAG 4380
ArD7117             GGAAACAGTCCTCGGCTTGACGTGGCACTAGATGAGAGTGGTGATTTCTCCTTGGTAGAG 4380
ARD_41519           GGAAACAGTCCCCGGCTTGACGTGGCACTGGATGAGAGTGGTGATTTCTCCTTGGTAGAG 4380
ARD157995           GGAAACAGTCCTCGGCTTGACGTGGCACTGGATGAGAGTGGTGATTTCTCCTTGGTAGAG 4380
MR_766              GGAAACAGTCCTCGGCTTGACGTGGCACTGGATGAGAGTGGTGACTTCTCCTTGGTAGAG 4368
ARD158084           GGAAACAGTCCTCGGCTTGACGTGGCACTGGATGAGAGTGGTGATTTCTCCTTGGTAGAG 4380
ArB1362             GGAAATAGTCCTCGGCTTGACGTGGCACTAGATGAGAGTGGTGATTTCTCTCTGGTAGAG 4380
ARB13565            GGAAATAGTCCTCGGCTTGACGTGGCACTAGATGAGAGTGGTGATTTCTCTCTGGTGGAG 4380
ARB7701             GGAAATAGTCCTCGGCTTGACGTGGCACTAGATGAGAGTGGTGATTTCTCTCTGGTGGAG 4380
ARB15076            GGAAATAGTCCTCGGCTTGACGTGGCACTAGATGAGAGTGGTGATTTCTCTCTGGTGGAG 4362
                    ***** ** ** ***** ** ***** ** ************** *****  * ** ***

P6-740              GATGATGGTCCCCCCATGAGAGAGATCATACTCAAGGTGGTCCTGATGACCATCTGTGGC 4440
CPC0740             GATGATGGTCCACCCATGAGAGAGATCATACTCAAAGTGGTCCTGATGACCATCTGCGGC 4440
YAP2007             GATGATGGTCCCCCCATGAGAGAGATCATACTCAAAGTGGTCCTGATGACCATCTGTGGC 4440
FSS13025            GATGATGGTCCCCCCATGAGAGAGATCATACTCAAAGTGGTCCTGATGaCCATCTGTGGC 4440
PLCal_ZV            GATGATGGTCCCCCCATGAGAGAGATCATACTCAAAGTGGTCCTGATGACCATCTGTGGC 4329
SV0127/14           GATGACGGTCCCCCCATGAGAGAGATCATACTCAAAGTGGTCCTGATGACCATCTGTGGC 4440
8375                GATGACGGTCCCCCCATGAGAGAGATCATACTCAAGGTGGTCCTGATGACCATCTGTGGC 4440
103344              GATGACGGTCCCCCCATGAGAGAGATCATACTCAAGGTGGTCCTGATGACCATCTGTGGC 4440
BrasilZKV2015       GATGACGGTCCCCCCATGAGAGAGATCATACTCAAGGTGGTCCTGATGACCATCTGTGGC 4440
GD01                GATGACGGTCCCCCCATGAGAGAGATCATACTCAAGGTGGTCCTGATGACCATCTGTGGC 4440
Martinique          GATGACGGTCCCCCCATGAGAGAGATCATACTCAAGGTGGTCCTGATGACCATCTGTGGC 4440
NatalRGN            GATGACGGTCCCCCCATGAGAGAGATCATACTCAAGGTGGTCCTGATGACCATCTGTGGC 4440
HPF2013             GATGACGGTCCCCCCATGAGAGAGATCATACTCAAGGTGGTCCTGATGACCATCTGTGGC 4440
SPH2015             GATGACGGTCCCCCCATGAGAGAGATCATACTCAAGGTGGTCCTGATGACCATCTGTGGC 4440
Haiti2014           GATGACGGTCCCCCCATGAGAGAGATCATACTCAAGGTGGTCCTGATGACCATCTGTGGC 4440
PRVABC59            GATGACGGTCCCCCCATGAGAGAGATCATACTCAAGGTGGTCCTGATGACCATCTGTGGC 4440
BeH819015           GATGACGGTCCCCCCATGAGAGAGATCATACTCAAGGTGGTCCTGATGACCATCTGTGGC 4440
Z1106033            GATGACGGTCCCCCCATGAGAGAGATCATACTCAAGGTGGTCCTGATGACCATCTGTGGC 4440
BEH819966           GATGACGGTCCCCCCATGAGAGAGATCATACTCAAGGTGGTCCTGATGACCATCTGTGGC 4440
SSABr               GATGACGGTCCCCCCATGAGAGAGATCATACTCAAGGTGGTCCTGATGACCATCTGTGGC 4440
Beh815744           GATGACGGTCCCCCCATGAGAGAGATCATACTCAAGGTGGTCCTGATGACCATCTGTGGC 4440
BEH818995           GATGACGGTCCCCCCATGAGAGAGATCATACTCAAGGTGGTCCTGATGACCATCTGTGGC 4440
IbH30656            GAGGATGGCCCACCCATGAGAGAGATCATACTCAAGGTGGTCCTGATGGCCATCTGTGGC 4422
ArD128000           GAGGATGGCCCACCCATGAGAGAGATCATACTCAAGGTGGTCCTGATGGCCATCTGTGGC 4440
```

```
ArD7117             GAGGATGGCCCACCCATGAGAGAGATCATACTCAAGGTGGTCCTGATGGCCATCTGTGGC 4440
ARD_41519           GAGGATGGCCCACCCATGAGAGAGATCATACTCAAGGTGGTCCTGATGGCCATCTGTGGC 4440
ARD157995           GAAGATGGTCCACCCATGAGAGAGATCATACTCAAGGTGGTCCTGATGGCCATCTGTGGC 4440
MR_766              GAAGATGGTCCACCCATGAGAGAGATCATACTCAAGGTGGTCCTGATGGCCATCTGTGGC 4428
ARD158084           GAAGATGGTCCACCCATGAGAGAGATCATACTCAAGGTGGTCCTGATGGCCATCTGTGGC 4440
ArB1362             GAAGATGGTCCACCCATGAGAGAGATCATACTCAAGGTGGTCCTGATGGCCATCTGTGGC 4440
ARB13565            GAAGATGGTCCACCCATGAGAGAGATCATACTCAAGGTGGTCCTGATGGCCATCTGTGGC 4440
ARB7701             GAAGATGGTCCACCCATGAGAGAGATCATACTCAAGGTGGTCCTGATGGCCATCTGTGGC 4440
ARB15076            GAAGATGGTCCACCCATGAGAGAGATCATACTCAAGGTGGTCCTGATGGCCATCTGTGGC 4422
                    ** ** ** ** *********************** ************ ******* ***

P6-740              ATGAACCCAATAGCCATACCCTTTGCAGCTGGAGCGTGGTATGTGTATGTGAAGACTGGA 4500
CPC0740             ATGAACCCAATAGCCATACCCTTTGCAGCTGGAGCGTGGTACGTGTATGTGAAGACTGGA 4500
YAP2007             ATGAACCCAATAGCCATACCCTTTGCAGCTGGAGCGTGGTACGTGTATGTGAAGACTGGA 4500
FSS13025            ATGAACCCAATAGCCATACCCTTTGCAGCTGGAGCGTGGTACGTGTATGTGAAGACTGGA 4500
PLCal_ZV            ATGAACCCAATAGCCATACCCTTTGCAGCTGGAGCGTGGTACGTATATGTGAAGACTGGA 4389
SV0127/14           ATGAACCCAATAGCCATACCCTTTGCAGCTGGAGCGTGGTACGTATACGTGAAAACTGGA 4500
8375                ATGAACCCAATAGCCATACCCTTTGCAGCTGGAGCGTGGTACGTATACGTGAAGACTGGA 4500
103344              ATGAACCCAATAGCCATACCCTTTGCAGCTGGAGCGTGGTACGTATACGTGAAGACTGGA 4500
BrasilZKV2015       ATGAACCCAATAGCCATACCCTTTGCAGCTGGAGCGTGGTACGTATACGTGAAGACTGGA 4500
GD01                ATGAACCCAATAGCCATACCCTTTGCAGCTGGAGCGTGGTACGTATACGTGAAGACTGGA 4500
Martinique          ATGAACCCAATAGCCATACCCTTTGCAGCTGGAGCGTGGTACGTATACGTGAAGACTGGA 4500
NatalRGN            ATGAACCCAATAGCCATACCCTTTGCAGCTGGAGCGTGGTACGTATACGTGAAGACTGGA 4500
HPF2013             ATGAACCCAATAGCCATACCCTTTGCAGCTGGAGCGTGGTACGTATACGTGAAGACTGGA 4500
SPH2015             ATGAACCCAATAGCCATACCCTTTGCAGCTGGAGCGTGGTACGTATACGTGAAGACTGGA 4500
Haiti2014           ATGAACCCAATAGCCATACCCTTTGCAGCTGGAGCGTGGTACGTATACGTGAAGACTGGA 4500
PRVABC59            ATGAACCCAATAGCCATACCCTTTGCAGCTGGAGCGTGGTACGTATACGTGAAGACTGGA 4500
BeH819015           ATGAATCCAATAGCCATACCCTTTGCAGCTGGAGCGTGGTACGTATACGTGAAGACTGGA 4500
Z1106033            ATGAACCCAATAGCCATACCCTTTGCAGCTGGAGCGTGGTACGTATACGTGAAGACTGGA 4500
BEH819966           ATGAACCCAATAGCCATACCCTTTGCAGCTGGAGCGTGGTACGTATACGTGAAGACTGGA 4500
SSABr               ATGAACCCAATAGCCATACCCTTTGCAGCTGGAGCGTGGTACGTATACGTGAAGACTGGA 4500
Beh815744           ATGAACCCAATAGCCATACCCTTTGCAGCTGGAGCGTGGTACGTATACGTGAAGACTGGA 4500
BEH818995           ATGAACCCAATAGCCATACCCTTTGCAGCTGGAGCGTGGTACGTATACGTGAAGACTGGA 4500
IbH30656            ATGAACCCAATAGCCATACCCTTCGCTGCAGGAGCGTGGTATGTGTATGTAAAGACTGGG 4482
ArD128000           ATGAACCCAATAGCCATACCCTTCGCTGCAGGAGCGTGGTATGTGTATGTAAAGACTGGG 4500
ArD7117             ATGAACCCAATAGCCATACCCTTCGCTGCAGGAGCGTGGTATGTGTATGTAAAGACTGGA 4500
ARD_41519           ATGAACCCAATAGCCATACCCTTCGCTGCAGGAGCGTGGTATGTGTATGTAAAGACTGGA 4500
ARD157995           ATGAACCCAATAGCTATACCTTTTGCTGCAGGAGCGTGGTATGTGTATGTGAAGACTGGG 4500
MR_766              ATGAACCCAATAGCTATACCTTTTGCTGCAGGAGCGTGGTATGTGTATGTGAAGACTGGG 4488
ARD158084           ATGAACCCAATAGCTATACCTTTTGCTGCAGGAGCGTGGTATGTGTATGTGAAGACTGGG 4500
ArB1362             ATGAACCCAATAGCCATACCTTTTGCTGCAGGAGCGTGGTATGTGTATGTGAAGACTGGG 4500
ARB13565            ATGAACCCAATAGCCATACCCTTTGCTGCAGGAGCGTGGTATGTGTATGTGAAGACTGGA 4500
ARB7701             ATGAACCCAATAGCCATACCCTTTGCTGCAGGAGCGTGGTATGTGTATGTGAAGACTGGA 4500
ARB15076            ATGAACCCAATAGCCATACCCTTTGCTGCAGGAGCGTGGTATGTGTATGTGAAGACTGGG 4482
                    ***** ******** ***** ** ** ** *********** ** ** ** ** *****

P6-740              AAGAGGAGTGGTGCTCTATGGGATGTGCCTGCTCCCAAGGAAGTAAAAAAGGGGGAGACC 4560
CPC0740             AAAAGGAGTGGTGCTCTATGGGATGTGCCTGCTCCCAAGGAAGTAAAAAAGGGGGAGACC 4560
YAP2007             AAAAGGAGTGGTGCTCTATGGGATGTGCCTGCTCCCAAGGAAGTAAAAAAGGGGGAGACC 4560
FSS13025            AAAAGGAGTGGTGCTCTATGGGATGTGCCTGCTCCCAAGGAAGTAAAAAAGGGGGAGACC 4560
PLCal_ZV            AAAAGGAGTGGTGCTCTATGGGATGTGCCTGCTCCCAAGGAAGTAAAAAAGGGGGAGACC 4449
SV0127/14           AAAAGGAGTGGTGCTCTATGGGATGTGCCTGCTCCCAAGGAAGTAAAAAAGGGGGAGACC 4560
8375                AAAAGGAGTGGTGCTCTATGGGATGTGCCTGCTCCCAAGGAAGTAAAAAAGGGGGAGACC 4560
103344              AAAAGGAGTGGTGCTCTATGGGATGTGCCTGCTCCCAAGGAAGTAAAAAAGGGGGAGACC 4560
BrasilZKV2015       AAAAGGAGTGGTGCTCTATGGGATGTGCCTGCTCCCAAGGAAGTAAAAAAGGGGGAGACC 4560
GD01                AAAAGGAGTGGTGCTCTATGGGATGTGCCTGCTCCCAAGGAAGTAAAAAAGGGGGAGACC 4560
Martinique          AAAAGGAGTGGTGCTCTATGGGATGTGCCTGCTCCCAAGGAAGTAAAAAAGGGGGAGACC 4560
NatalRGN            AAAAGGAGTGGTGCTCTATGGGATGTGCCTGCTCCCAAGGAAGTAAAAAAGGGGGAGACC 4560
HPF2013             AAAAGGAGTGGTGCTCTATGGGATGTGCCTGCTCCCAAGGAAGTAAAAAAGGGGGAGACC 4560
SPH2015             AAAAGGAGTGGTGCTCTATGGGATGTGCCTGCTCCCAAGGAAGTAAAAAAGGGGGAGACC 4560
Haiti2014           AAAAGGAGTGGTGCTCTATGGGATGTGCCTGCTCCCAAGGAAGTAAAAAAGGGGGAGACC 4560
PRVABC59            AAAAGGAGTGGTGCTCTATGGGATGTGCCTGCTCCCAAGGAAGTAAAAAAGGGGGAGACC 4560
BeH819015           AAAAGGAGTGGTGCTCTATGGGATGTGCCTGCTCCCAAGGAAGTAAAAAAGGGGGAGACC 4560
Z1106033            AAAAGGAGTGGTGCTCTATGGGATGTGCCTGCTCCCAAGGAAGTAAAAAAGGGGGAGACC 4560
BEH819966           AAAAGGAGTGGTGCTCTATGGGATGTGCCTGCTCCCAAGGAAGTAAAAAAGGGGGAGACC 4560
SSABr               AAAAGGAGTGGTGCTCTATGGGATGTGCCTGCTCCCAAGGAAGTAAAAAAGGGGGAGACC 4560
Beh815744           AAAAGGAGTGGTGCTCTATGGGATGTGCCTGCTCCCAAGGAAGTAAAAAAGGGGGAGACC 4560
BEH818995           AAAAGGAGTGGTGCTCTATGGGATGTGCCTGCTCCCAAGGAAGTAAAAAAGGGGGAGACC 4560
```

```
IbH30656          AAAAGGAGCGGTGCCCTCTGGGACGTGCCTGCTCCCAAAGAAGTAAAAAAGGAGAGACT 4542
ArD128000         AAAAGGAGTGGTGCCCTCTGGGACGTGCCTGCTCCCAAAGAAGTGAAAAAAGGAGAGACT 4560
ArD7117           AAAAGGAGTGGTGCCCTCTGGGACGTGCCTGCTCCCAAAGAAGTGAAAAAAGGAGAGACT 4560
ARD_41519         AAAAGGAGTGGTGCCCTCTGGGACGTGCCTGCTCCCAAAGAAGTGAAAAAAGGAGAGACT 4560
ARD157995         AAAAGGAGTGGCGCCCTCTGGGACGTGCCTGCTCCCAAAGAAGTGAAGAAAGGAGAGACC 4560
MR_766            AAAAGGAGTGGCGCCCTCTGGGACGTGCCTGCTCCCAAAGAAGTGAAGAAAGGAGAGACC 4548
ARD158084         AAAAGGAGTGGCGCCCTCTGGGACGTGCCTGCTCCCAAAGAAGTGAAGAAAGGAGAGACC 4560
ArB1362           AAAAGGAGTGGTGCCCTCTGGGATGTGCCAGCTCCTAAAGAAGTGAAAAAAGGAGAGACC 4560
ARB13565          AAAAGGAGTGGTGCCCTCTGGGATGTGCCCGCTCCCAAAGAAGTGAAAAAAGGAGAGACC 4560
ARB7701           AAAAGGAGTGGTGCCCTCTGGGATGTGCCCGCTCCCAAAGAAGTGAAAAAAGGAGAGACC 4560
ARB15076          AAAAGGAGTGGTGCCCTCTGGGATGTGCCCGCTCCCAAAGAAGTGAAAAAAGGAGAGACC 4542
                  ** ***** ** ** ** ***** ***** ***** ** ***** ** ** ** *****

P6-740            ACAGATGGAGTGTATAGAGTGATGACTCGCAGACTGCTAGGTTCAACACAAGTTGGAGTG 4620
CPC0740           ACAGATGGAGTGTACAGAGTAATGACTCGTAGACTGCTTGGTTCAACACAAGTTGGAGTG 4620
YAP2007           ACAGATGGAGTGTACAGAGTAATGACTCGTAGACTGCTTGGTTCAACACAAGTTGGAGTG 4620
FSS13025          ACAGATGGAGTGTACAGAGTAATGACTCGTAGACTGCTAGGTTCAACACAAGTTGGAGTG 4620
PLCal_ZV          ACAGATGGAGTGTACAGAGTAATGACTCGTAGACTGCTAGGTTCAACACAAGTTGGAGTG 4509
SV0127/14         ACAGATGGAGTGTACAGAGTAATGACTCGTAGACTGCTAGGTTCAACACAAGTTGGAGTG 4620
8375              ACAGATGGAGTGTACAGAGTAATGACTCGTAGACTGCTAGGTTCAACACAAGTTGGAGTG 4620
103344            ACAGATGGAGTGTACAGAGTAATGACTCGTAGACTGCTAGGTTCAACACAAGTTGGAGTG 4620
BrasilZKV2015     ACAGATGGAGTGTACAGAGTAATGACTCGTAGACTGCTAGGTTCAACACAAGTTGGAGTG 4620
GD01              ACAGATGGAGTGTACAGAGTAATGACTCGCAGACTGCTAGGTTCAACACAAGTTGGAGTG 4620
Martinique        ACAGATGGAGTGTACAGAGTAATGACTCGTAGACTGCTAGGTTCAACACAAGTTGGAGTG 4620
NatalRGN          ACAGATGGAGTGTACAGAGTAATGACTCGTAGACTGCTAGGTTCAACACAAGTTGGAGTG 4620
HPF2013           ACAGATGGAGTGTACAGAGTAATGACTCGTAGACTGCTAGGTTCAACACAAGTTGGAGTG 4620
SPH2015           ACAGATGGAGTGTACAGAGTAATGACTCGTAGACTGCTAGGTTCAACACAAGTTGGAGTG 4620
Haiti2014         ACAGATGGAGTGTACAGAGTAATGACTCGTAGACTGCTAGGTTCAACACAAGTTGGAGTG 4620
PRVABC59          ACAGATGGAGTGTACAGAGTAATGACTCGTAGACTGCTAGGTTCAACACAAGTTGGAGTG 4620
BeH819015         ACAGATGGAGTGTACAGAGTAATGACTCGTAGACTGCTAGGTTCAACACAAGTTGGAGTG 4620
Z1106033          ACAGATGGAGTGTACAGAGTAATGACTCGTAGACTGCTAGGTTCAACACAAGTTGGAGTG 4620
BEH819966         ACAGATGGAGTGTACAGAGTAATGACTCGTAGACTGCTAGGTTCAACACAAGTTGGAGTG 4620
SSABr             ACAGATGGAGTGTACAGAGTAATGACTCGTAGACTGCTAGGTTCAACACAAGTTGGAGTG 4620
Beh815744         ACAGATGGAGTGTACAGAGTAATGACTCGTAGACTGCTAGGTTCAACACAAGTTGGAGTG 4620
BEH818995         ACAGATGGAGTGTACAGAGTAATGACTCGTAGACTGCTAGGTTCAACACAAGTTGGAGTG 4620
IbH30656          ACAGATGGAGTGTACAGAGTTATGACTCGCAGACTGCTGGGTTCAACACAGGTTGGAGTG 4602
ArD128000         ACAGATGGAGTGTACAGAGTGATGACTCGCAGACTGCTGGGTTCAACACAGGTTGGAGTG 4620
ArD7117           ACAGATGGAGTGTACAGAGTGATGACTCGCAGACTGCTGGGTTCAACACAGGTTGGAGTG 4620
ARD_41519         ACAGATGGAGTGTACAGAGTGATGACTCGCAGACTGCTGGGTTCAACACAGGTTGGAGTG 4620
ARD157995         ACAGATGGAGTGTACAGAGTGATGACTCGCAGACTGCTAGGTTCAACACAGGTTGGAGTG 4620
MR_766            ACAGATGGAGTGTACAGAGTGATGACTCGCAGACTGCTAGGTTCAACACAGGTTGGAGTG 4608
ARD158084         ACAGATGGAGTGTACAGAGTGATGACTCGCAGACTGCTAGGTTCAACACAGGTTGGAGTG 4620
ArB1362           ACAGATGGAGTATACAGAGTGATGACCCGCAGATTGCTGGGTTCAACACAGGTTGGAGTG 4620
ARB13565          ACAGATGGGGTATACAGAGTGATGACCCGCAGACTGCTGGGTTCAACACAGGTTGGAGTG 4620
ARB7701           ACAGATGGGGTATACAGAGTGATGACCCGCAGACTGCTGGGTTCAACACAGGTTGGAGTG 4620
ARB15076          ACAGATGGAGTATACAGAGTGATGACCCGCAGACTGCTGGGTTCAACACAGGTTGGAGTT 4602
                  ******** ** ** ***** ***** ** *** **** *********** ********

P6-740            GGAGTCATGCAAGAGGGGGTCTTCCACACTATGTGGCACGTCACAAAAGGATCCGCGCTG 4680
CPC0740           GGAGTCATGCAAGAGGGGGTCTTCCACACTATGTGGCACGTCACAAAAGGATCCGCGCTG 4680
YAP2007           GGAGTCATGCAAGAGGGGGTCTTCCACACTATGTGGCACGTCACAAAAGGATCCGCGCTG 4680
FSS13025          GGAGTCATGCAAGAGGGGGTCTTCCACACTATGTGGCACGTCACAAAAGGATCCGCGCTG 4680
PLCal_ZV          GGAGTCATGCAAGAGGGGGTCTTTCACACTATGTGGCACGTCACAAAAGGATCCGCGCTG 4569
SV0127/14         GGAGTTATGCAAGAGGGGGTCTTTCACACTATGTGGCATGTCACAAAAGGATCCGCGCTG 4680
8375              GGAGTTATGCAAGAGGGGGTCTTTCACACTATGTGGCACGTCACAAAAGGATCCGCACTG 4680
103344            GGAGTTATGCAAGAGGGGGTCTTTCACACTATGTGGCACGTCACAAAAGGATCCGCACTG 4680
BrasilZKV2015     GGAGTTATGCAAGAGGGGGTCTTTCACACTATGTGGCACGTCACAAAAGGATCCGCGCTG 4680
GD01              GGAGTTATGCAAGAGGGGGTCTTTCACACTATGTGGCACGTCACAAAAGGATCCGCGCTG 4680
Martinique        GGAGTTATGCAAGAGGGGGTCTTTCACACTATGTGGCACGTCACAAAAGGATCCGCGCTG 4680
NatalRGN          GGAGTTATGCAAGAGGGGGTCTTTCACACTATGTGGCACGTCACAAAAGGATCCGCGCTG 4680
HPF2013           GGAGTTATGCAAGAGGGGGTCTTTCACACTATGTGGCACGTCACAAAAGGATCCGCGCTG 4680
SPH2015           GGAGTTATGCAAGAGGGGGTCTTTCACACTATGTGGCACGTCACAAAAGGATCCGCGCTG 4680
Haiti2014         GGAGTTATGCAAGAGGGGGTCTTTCACACTATGTGGCACGTCACAAAAGGATCCGCGCTG 4680
PRVABC59          GGAGTTATGCAAGAGGGGGTCTTTCACACTATGTGGCACGTCACAAAAGGATCCGCGCTG 4680
BeH819015         GGAGTTATGCAAGAGGGGGTCTTTCACACTATGTGGCACGTCACAAAAGGATCCGCGCTG 4680
Z1106033          GGAGTTATGCAAGAGGGGGTCTTTCACACTATGTGGCACGTCACAAAAGGATCCGCGCTG 4680
BEH819966         GGAGTTATGCAAGAGGGGGTCTTTCACACTATGTGGCACGTCACAAAAGGATCCGCGCTG 4680
SSABr             GGAGTTATGCAAGAGGGGGTCTTTCACACTATGTGGCACGTCACAAAAGGATCCGCGCTG 4680
```

```
Beh815744          GGAGTTATGCAAGAGGGGGTCTTTCACACTATGTGGCACGTCACAAAAGGATCCGCGCTG 4680
BEH818995          GGAGTTATGCAAGAGGGGGTCTTTCACACTATGTGGCACGTCACAAAAGGATCCGCGCTG 4680
IbH30656           GGAGTCATGCAAGAGGGAGTCTTCCATACCATGTGGCACGTCACAAAAGGAGCCGCATTG 4662
ArD128000          GGGGTCATGCAAGAGGGAGTCTTCCACACCATGTGGCACGTCACAAAAGGAGCTGCATTG 4680
ArD7117            GGAGTCATGCAAGAGGGAGTCTTCCACACCATGTGGCACGTCACAAAAGGAGCCGCATTG 4680
ARD_41519          GGGGTCATGCAAGAGGGAGTCTTCCACACCATGTGGCACGTCACAAAAGGAGCCGCATTG 4680
ARD157995          GGAGTCATGCAAGAGGGAGTCTTCCACACCATGTGGCACGTTACAAAAGGAGCCGCACTG 4680
MR_766             GGAGTCATGCAAGAGGGAGTCTTCCACACCATGTGGCACGTTACAAAAGGAGCCGCACTG 4668
ARD158084          GGAGTCATGCAAGAGGGAGTCTTCCACACCATGTGGCACGTTACAAAAGGAGCCGCACTG 4680
ArB1362            GGAGTCATGCAGGAGGGAGTCTTCCACACCATGTGGCACGTCACAAAAGGAGCCGCACTG 4680
ARB13565           GGAGTCATGCAAGAGGGAGTCTTCCACACCATGTGGCACGTCACAAAAGGAGCTGCACTG 4680
ARB7701            GGAGTCATGCAAGAGGGAGTCTTCCACACCATGTGGCACGTCACAAAAGGAGCTGCACTG 4680
ARB15076           GGAGTCATGCAAGAGGGAGTCTTCCACACCATGTGGCACGTCACAAAAGGAGCTGCACTG 4662
                   ** ** ***** ***** ***** ** ** ******** ** ********* * **  **

P6-740             AGGAGCGGTGAAGGGAGACTTGATCCATACTGGGGAGATGTTAAGCAGGATCTGGTGTCA 4740
CPC0740            AGAAGCGGTGAAGGGAGACTTGATCCATACTGGGGAGATGTCAAGCAGGATCTGGTGTCA 4740
YAP2007            AGAAGCGGTGAAGGGAGACTTGATCCATACTGGGGAGATGTCAAGCAGGATCTGGTGTCA 4740
FSS13025           AGAAGCGGTGAAGGGAGACTTGATCCATACTGGGGAGATGTCAAGCAGGATCTGGTGTCA 4740
PLCal_ZV           AGAAGCGGTGAAGGGAGACTTGATCCATACTGGGGAGATGTCAAGCAGGATCTGGTGTCA 4629
SV0127/14          AGAAGCGGTGAAGGGAGACTTGATCCATACTGGGGAGATGTCAAGCAGGATCTGGTGTCA 4740
8375               AGAAGCGGTGAAGGGAGACTTGATCCATACTGGGGAGATGTCAAGCAGGATCTGGTGTCA 4740
103344             AGAAGCGGTGAAGGGAGACTTGATCCATACTGGGGAGATGTCAAGCAGGATCTGGTGTCA 4740
BrasilZKV2015      AGAAGCGGTGAAGGGAGACTTGATCCATACTGGGGAGATGTCAAGCAGGATCTGGTGTCA 4740
GD01               AGAAGCGGTGAAGGGAGACTTGATCCATACTGGGGAGATGTCAAGCAGGATCTGGTGTCA 4740
Martinique         AGAAGCGGTGAAGGGAGACTTGATCCATACTGGGGAGATGTCAAGCAGGATCTGGTGTCA 4740
NatalRGN           AGAAGCGGTGAAGGGAGACTTGATCCATACTGGGGAGATGTCAAGCAGGATCTGGTGTCA 4740
HPF2013            AGAAGCGGTGAAGGGAGACTTGATCCATACTGGGGAGATGTCAAGCAGGATCTGGTGTCA 4740
SPH2015            AGAAGCGGTGAAGGGAGACTTGATCCATACTGGGGAGATGTCAAGCAGGATCTGGTGTCA 4740
Haiti2014          AGAAGCGGTGAAGGGAGACTTGATCCATACTGGGGAGATGTCAAGCAGGATCTGGTGTCA 4740
PRVABC59           AGAAGCGGTGAAGGGAGACTTGATCCATACTGGGGAGATGTCAAGCAGGATCTGGTGTCA 4740
BeH819015          AGAAGCGGTGAAGGGAGACTTGATCCATACTGGGGAGATGTCAAGCAGGATCTGGTGTCA 4740
Z1106033           AGAAGCGGTGAAGGGAGACTTGATCCATACTGGGGAGATGTCAAGCAGGATCTGGTGTCA 4740
BEH819966          AGAAGCGGTGAAGGGAGACTTGATCCATACTGGGGAGATGTCAAGCAGGATCTGGTGTCA 4740
SSABr              AGAAGCGGTGAAGGGAGACTTGATCCATACTGGGGAGATGTCAAGCAGGATCTGGTGTCA 4740
Beh815744          AGAAGCGGTGAAGGGAGACTTGATCCATACTGGGGAGATGTCAAGCAGGATCTGGTGTCA 4740
BEH818995          AGAAGCGGTGAAGGGAGACTTGATCCATACTGGGGAGATGTCAAGCAGGATCTGGTGTCA 4740
IbH30656           AGGAGCGGTGAAGGAAGACTTGATCCATACTGGGGGGACGTCAAGCAGGACCTGGTGTCA 4722
ArD128000          AGGAGCGGTGAAGGAAGACTTGATCCATATTGGGGGGACGTCAAGCAGGACTTGGTGTCA 4740
ArD7117            AGGAGCGGTGAAGGAAGACTTGATCCATATTGGGGGGACGTCAAGCAGGACTTGGTGTCA 4740
ARD_41519          AGGAGCGGTGAAGGAAGACTTGATCCATATTGGGGGGACGTCAAGCAGGACTTGGTGTCA 4740
ARD157995          AGGAGCGGTGAGGGAAGACTTGATCCATACTGGGGGGATGTCAAGCAGGACTTGGTGTCA 4740
MR_766             AGGAGCGGTGAGGGAAGACTTGATCCATACTGGGGGGATGTCAAGCAGGACTTGGTGTCA 4728
ARD158084          AGGAGCGGTGAGGGAAGACTTGATCCATACTGGGGGGATGTCAAGCAGGACTTGGTGTCA 4740
ArB1362            AGGAGCGGTGAAGGGAGACTTGATCCATACTGGGGTGATGTCAAGCAGGACTTGGTGTCA 4740
ARB13565           AGGAGCGGTGAAGGGAGACTTGATCCATACTGGGGGGATGTCAAGCAGGACTTAGTGTCA 4740
ARB7701            AGGAGCGGTGAAGGGAGACTTGATCCATACTGGGGGGATGTCAAGCAGGACTTAGTGTCA 4740
ARB15076           AGGAGCGGCGAAGGGAGACTTGATCCATACTGGGGGGATGTCAAGCAGGACTTAGTGTCA 4722
                   ** ***** ** ** ************** ***** ** ** ********  * ******

P6-740             TACTGTGGCCCGTGGAAGCTAGATGCCGCTTGGGACGGACACAGCGAGGTGCAGCTTTTG 4800
CPC0740            TACTGTGGTCCGTGGAAGCTAGACGCCGCCTGGGACGGGCACAGCGAGGTGCAGCTCTTG 4800
YAP2007            TATTGTGGTCCGTGGAAGCTAGACGCCGCCTGGGACGGGCACAGCGAGGTGCAGCTCTTG 4800
FSS13025           TACTGTGGTCCATGGAAGCTAGATGCCGCCTGGGACGGGCACAGCGAGGTGCAGCTCTTG 4800
PLCal_ZV           TACTGTGGTCCATGGAAGCTAGATGCCGCCTGGGACGGACACAGCGAGGTGCAGCTCTTG 4689
SV0127/14          TACTGTGGTCCATGGAAGCTAGATGCCGCCTGGGACGGGCACAGCGAGGTGCAGCTCTTG 4800
8375               TACTGTGGTCCATGGAAGCTAGATGCCGCCTGGGACGGGCACAGCGAGGTGCAGCTCTTG 4800
103344             TACTGTGGTCCATGGAAGCTAGATGCCGCCTGGGACGGGCACAGCGAGGTGCAGCTCTTG 4800
BrasilZKV2015      TACTGTGGTCCATGGAAGCTAGATGCCGCCTGGGACGGGCACAGCGAGGTGCAGCTCTTG 4800
GD01               TACTGTGGTCCATGGAAGCTAGATGCCGCCTGGGACGGGCACAGCGAGGTGCAGCTCTTG 4800
Martinique         TACTGTGGTCCATGGAAGCTAGATGCCGCCTGGGACGGGCACAGCGAGGTGCAGCTCTTG 4800
NatalRGN           TACTGTGGTCCATGGAAGCTAGATGCCGCCTGGGACGGGCACAGCGAGGTGCAGCTCTTG 4800
HPF2013            TACTGTGGTCCATGGAAGCTAGATGCCGCCTGGGACGGGCACAGCGAGGTGCAGCTCTTG 4800
SPH2015            TACTGTGGTCCATGGAAGCTAGATGCCGCCTGGGACGGGCACAGCGAGGTGCAGCTCTTG 4800
Haiti2014          TACTGTGGTCCATGGAAGCTAGATGCCGCCTGGGACGGGCACAGCGAGGTGCAGCTCTTG 4800
PRVABC59           TACTGTGGTCCATGGAAGCTAGATGCCGCCTGGGATGGGCACAGCGAGGTGCAGCTCTTG 4800
BeH819015          TACTGTGGTCCATGGAAGCTAGATGCCGCCTGGGACGGGCACAGCGAGGTGCAGCTCTTG 4800
Z1106033           TACTGTGGTCCATGGAAGCTAGATGCCGCCTGGGACGGGCACAGCGAGGTGCAGCTCTTG 4800
```

```
BEH819966          TACTGTGGTCCATGGAAGCTAGATGCCGCCTGGGACGGGCACAGCGAGGTGCAGCTCTTG 4800
SSABr              TACTGTGGTCCATGGAAGCTAGATGCCGCCTGGGACGGGCACAGCGAGGTGCAGCTCTTG 4800
Beh815744          TACTGTGGTCCATGGAAGCTAGATGCCGCCTGGGACGGGCACAGCGAGGTGCAGCTCTTG 4800
BEH818995          TACTGTGGTCCATGGAAGCTAGATGCCGCCTGGGACGGGCACAGCGAGGTGCAGCTCTTG 4800
IbH30656           TATTGTGGGCCGTGGAAGTTGGATGCAGCCTGGGATGGACTAAGTGAGGTGCAGCTTTTG 4782
ArD128000          TATTGTGGGCCGTGGAAGTTGGACGCAACCTGGGATGGACTAAGTGAAGTGCAGCTTTTG 4800
ArD7117            TATTGTGGGCCGTGGAAGTTGGATGCAGCCTGGGATGGACTAAGTGAAGTGCAGCTTTTG 4800
ARD_41519          TATTGTGGGCCGTGGAAGTTGGATGCAGCCTGGGATGGACTAAGTGAAGTGCAGCTTTTG 4800
ARD157995          TACTGTGGGCCTTGGAAGTTGGATGCAGCTTGGGATGGACTTAGCGAGGTACAGCTTTTG 4800
MR_766             TACTGTGGGCCTTGGAAGTTGGATGCAGCTTGGGATGGACTCAGCGAGGTACAGCTTTTG 4788
ARD158084          TACTGTGGGCCTTGGAAGTTGGATGCAGCTTGGGATGGACTTAGCGAGGTACAGCTTTTG 4800
ArB1362            TACTGTGGGCCTTGGAAGCTGGATGCAGCTTGGGATGGACTTAGTGAGGTGCAGCTCTTG 4800
ARB13565           TACTGTGGGCCTTGGAAGTTGGATGCAGCTTGGGATGGACTTAGTGAGGTGCAGCTCTTG 4800
ARB7701            TACTGTGGGCCTTGGAAGTTGGATGCAGCTTGGGATGGACTTAGTGAGGTGCAGCTCTTG 4800
ARB15076           TACTGTGGGCCTTGGAAGTTGGATGCAGCTTGGGATGGACTTAGTGAGGTGCAGCTCTTG 4782
                   ** ***** ** ****** * ** **  * ***** ** *  ** ** ** ***** ***

P6-740             GCCGTGCCCCCCGGAGAGAGAGCGAGGAACATCCAGACTCTGCCCGGAATATTCAAGACA 4860
CPC0740            GCCGTGCCCCCCGGAGAGAGAGCGAGGAACATCCAGACTCTGCCCGGAACATTTAAGACA 4860
YAP2007            GCCGTGCCCCCCGGAGAGAGAGCGAGGAACATCCAGACTCCGCCCGGAATATTTAAGACA 4860
FSS13025           GCCGTGCCCCCCGGAGAGAGAGCGAGGAACATCCAGACTCTGCCCGGAATATTTAAGACA 4860
PLCal_ZV           GCCGTGCCCCCCGGAGAGAGAGCGAGGAACATCCAGACTCTGCCCGGAATATTTAAGACA 4749
SV0127/14          GCCGTGCCCCCCGGAGAGAGAGCGAGGAACATCCAGACTCTGCCCGGAATATTTAAGACA 4860
8375               GCCGTGCCCCCCGGAGAGAGAGCGAGGAACATCCAGACTCTGCCCGGAATATTTAAGACA 4860
103344             GCCGTGCCCCCCGGAGAGAGAGCGAGGAACATCCAGACTCTGCCCGGAATATTTAAGACA 4860
BrasilZKV2015      GCCGTGCCCCCCGGAGAGAGAGCGAGGAACATCCAGACTCTGCCCGGAATATTTAAGACA 4860
GD01               GCCGTGCCCCCCGGAGAGAGAGCGAGGAACATCCAGACTCTGCCCGGAATATTTAAGACA 4860
Martinique         GCCGTGCCCCCCGGAGAGAGAGCGAGGAACATCCAGACTCTGCCCGGAATATTTAAGACA 4860
NatalRGN           GCCGTGCCCCCCGGAGAGAGAGCGAGGAACATCCAGACTCTGCCCGGAATATTTAAGACA 4860
HPF2013            GCCGTGCCCCCCGGAGAGAGAGCGAGGAACATCCAGACTCTGCCCGGAATATTTAAGACA 4860
SPH2015            GCCGTGCCCCCCGGAGAGAGAGCGAGGAACATCCAGACTCTGCCCGGAATATTTAAGACA 4860
Haiti2014          GCCGTGCCCCCCGGAGAGAGAGCGAGGAACATCCAGACTCTGCCCGGAATATTTAAGACA 4860
PRVABC59           GCCGTGCCCCCCGGAGAGAGAGCGAGGAACATCCAGACTCTGCCCGGAATATTTAAGACA 4860
BeH819015          GCCGTGCCCCCCGGAGAGAGAGCGAGGAACATCCAGACTCTGCCCGGAATATTTAAGACA 4860
Z1106033           GCCGTGCCCCCCGGAGAGAGAGCGAGGAACATCCAGACTCTGCCCGGAATATTTAAGACA 4860
BEH819966          GCCGTGCCCCCCGGAGAGAGAGCGAGGAACATCCAGACTCTGCCCGGAATATTTAAGACA 4860
SSABr              GCCGTGCCCCCCGGAGAGAGAGCGAGGAACATCCAGACTCTGCCCGGAATATTTAAGACA 4860
Beh815744          GCCGTGCCCCCCGGAGAGAGAGCGAGGAACATCCAGACTCTGCCCGGAATATTTAAGACA 4860
BEH818995          GCCGTGCCCCCCGGAGAGAGAGCGAGGAACATCCAGACTCTGCCCGGAATATTTAAGACA 4860
IbH30656           GCCGTACCCCCCGGAGAGAGGGCTAAAAACATTCAGACTCTGCCTGGAATATTTAAGACA 4842
ArD128000          GCCGTACCCCCCGGAGAGAGGGCTAGAAACATTCAGACTCTGCCTGGAATATTCAAGACA 4860
ArD7117            GCCGTACCCCCCGGAGAGAGGGCTAGAAACATTCAGACTCTGCCTGGAATATTCAAGACA 4860
ARD_41519          GCCGTACCCCCCGGAGAGAGGGCTAGAAACATTCAGACTCTGCCTGGAATATTCAAGACA 4860
ARD157995          GCCGTACCTCCCGGAGAGAGGGCCAGAAACATTCAGACCCTGCCTGGAATATTCAAGACA 4860
MR_766             GCCGTACCTCCCGGAGAGAGGGCCAGAAACATTCAGACCCTGCCTGGAATATTCAAGACA 4848
ARD158084          GCCGTACCTCCCGGAGAGAGGGCCAGAAACATTCAGACCCTGCCTGGAATATTCAAGACA 4860
ArB1362            GCAGTACCTCCCGGAGAGAGGGCCAGAAACATCCAGACTCTGCCTGGAATATTCAAGACA 4860
ARB13565           GCAGTACCCCCCGGAGAGAGGGCCAGAAACATTCAGACTCTGCCTGGAATATTCAAGACA 4860
ARB7701            GCAGTACCCCCCGGAGAGAGGGCCAGAAACATTCAGACTCTGCCTGGAATATTCAAGACA 4860
ARB15076           GCAGTACCCCCCGGAGAGAGGGCCAGAAACATTCAGACTCTGCCTGGAATATTCAAGACA 4842
                   ** ** ** *********** ** *  ***** ***** * *** **** *** ******

P6-740             AAGGATGGGGACATCGGAGCAGTTGCTCTGGACTACCCAGCAGGAACTTCAGGATCTCCG 4920
CPC0740            AAGGATGGGGACATTGGAGCAGTTGCGCTGGACTACCCAGCAGGAACTTCAGGATCTCCA 4920
YAP2007            AAGGATGGGGACATTGGAGCAGTTGCGTTGGACTACCCAGCAGGAACTTCAGGATCTCCA 4920
FSS13025           AAGGATGGGGACATTGGAGCAGTTGCGCTGGACTACCCAGCAGGAACTTCAGGATCTCCA 4920
PLCal_ZV           AAGGATGGGGACATTGGAGCGGTTGCACTGGACTACCCAGCAGGAACTTCAGGATCTCCA 4809
SV0127/14          AAGGATGGGGACATTGGAGCGGTTGCGCTGGACTATCCAGCAGGAACTTCAGGATCTCCA 4920
8375               AAGGATGGGGACATTGGAGCGGTTGCGCTGGATTACCCAGCAGGAACTTCAGGATCTCCA 4920
103344             AAGGATGGGGACATTGGAGCGGTTGCGCTGGATTACCCAGCAGGAACTTCAGGATCTCCA 4920
BrasilZKV2015      AAGGATGGGGACATTGGAGCGGTTGCGCTGGATTACCCAGCAGGAACTTCAGGATCTCCA 4920
GD01               AAGGATGGGGACATTGGAGCGGTTGCGCTGGATTACCCAGCAGGAACTTCAGGATCTCCA 4920
Martinique         AAGGATGGGGACATTGGAGCGGTTGCGCTGGATTACCCAGCAGGAACTTCAGGATCTCCA 4920
NatalRGN           AAGGATGGGGACATTGGAGCGGTTGCGCTGGATTACCCAGCAGGAACTTCAGGATCTCCA 4920
HPF2013            AAGGATGGGGACATTGGAGCGGTTGCGCTGGATTACCCAGCAGGAACTTCAGGATCTCCA 4920
SPH2015            AAGGATGGGGACATTGGAGCGGTTGCGCTGGATTACCCAGCAGGAACTTCAGGATCTCCA 4920
Haiti2014          AAGGATGGGGACATTGGAGCGGTTGCGCTGGATTACCCAGCAGGAACTTCAGGATCTCCA 4920
PRVABC59           AAGGATGGGGACATTGGAGCGGTTGCGCTGGATTACCCAGCAGGAACTTCAGGATCTCCA 4920
```

```
BeH819015          AAGGATGGGGACATTGGAGCGGTTGCGCTGGATTACCCAGCAGGAACTTCAGGATCTCCA 4920
Z1106033           AAGGATGGGGACATTGGAGCGGTTGCGCTGGATTACCCAGCAGGAACTTCAGGATCTCCT 4920
BEH819966          AAGGATGGGGACATTGGAGCGGTTGCGCTGGATTACCCAGCAGGAACTTCAGGATCTCCA 4920
SSABr              AAGGATGGGGACATTGGAGCGGTTGCGCTGGATTACCCAGCAGGAACTTCAGGATCTCCA 4920
Beh815744          AAGGATGGGGACATTGGAGCGGTTGCGCTGGATTACCCAGCAGGAACTTCAGGATCTCCA 4920
BEH818995          AAGGATGGGGACATTGGAGCGGTTGCGCTGGATTACCCAGCAGGAACTTCAGGATCTCCA 4920
IbH30656           AAGGATGGGGACATCGGAGCAGTTGCTCTAGACTACCCTGCAGGAACCTCAGGATCTCCG 4902
ArD128000          AAGGATGGGGACATCGGAGCGGTTGCTCTAGACTACCCCGCAGGAACCTCAGGATCTCCA 4920
ArD7117            AAGGATGGGGACATCGGAGCAGTCGCTCTAGACTACCCCGCAGGAACCTCAGGATCTCCA 4920
ARD_41519          AAGGATGGGGACATCGGAGCAGTCGCTCTAGACTACCCCGCAGGAACTTCAGGATCTCCA 4920
ARD157995          AAGGACGGGGACATCGGAGCAGTTGCTCTGGACTACCCTGCAGGGACCTCAGGATCTCCG 4920
MR_766             AAGGACGGGGACATCGGAGCAGTTGCTCTGGACTACCCTGCAGGGACCTCAGGATCTCCG 4908
ARD158084          AAGGACGGGGACATCGGAGCAGTTGCTCTGGACTACCCTGCAGGGACCTCAGGATCTCCG 4920
ArB1362            AAGGATGGGGACATCGGAGCAGTTGCTCTGGACTACCCCGCAGGAACCTCAGGATCTCCG 4920
ARB13565           AAGGATGGGGACATCGGAGCAGTCGCTCTGGACTACCCCGCAGGAACTTCAGGATCCCCG 4920
ARB7701            AAGGATGGGGACATCGGAGCAGTCGCTCTGGACTACCCCGCAGGAACTTCAGGATCCCCG 4920
ARB15076           AAGGATGGGGACATCGGAGCAGTTGCTCTGGACTACCCCGCAGGAACTTCAGGATCCCCG 4902
                   ***** ******** ***** ** **  * ** ** ** ***** ** ******** **

P6-740             ATCCTAGACAAGTGTGGGAGAGTGATAGGACTCTATGGCAATGGGGTCGTGATCAAAAAT 4980
CPC0740            ATCCTAGACAAGTGTGGGAGAGTGATAGGACTCTATGGTAATGGGGTCGTGATAAAAAAT 4980
YAP2007            ATCCTAGACAAGTGTGGGAGAGTGATAGGACTCTATGGTAATGGGGTCGTGATAAAAAAT 4980
FSS13025           ATCCTAGATAAGTGTGGGAGAGTGATAGGACTCTATGGTAATGGGGTCGTGATCAAAAAT 4980
PLCal_ZV           ATCCTAGACAAGTGTGGGAGAGTGATAGGACTCTATGGCAATGGGGTCGTGATCAAAAAT 4869
SV0127/14          ATCCTAGACAAGTGTGGGAGAGTGATAGGACTCTATGGCAATGGGGTCGTGATCAAGAAT 4980
8375               ATCCTAGACAAGTGTGGGAGAGTGATAGGACTTTATGGCAATGGGGTCGTGATCAAAAAT 4980
103344             ATCCTAGACAAGTGTGGGAGAGTGATAGGACTTTATGGCAATGGGGTCGTGATCAAAAAT 4980
BrasilZKV2015      ATCCTAGACAAGTGTGGGAGAGTGATAGGACTTTATGGCAATGGGGTCGTGATAAAAAAT 4980
GD01               ATCCTAGACAAGTGTGGGAGAGTGATAGGACTTTATGGCAATGGGGTCGTGATCAAAAAT 4980
Martinique         ATCCTAGACAAGTGTGGGAGAGTGATAGGACTTTATGGCAATGGGGTCGTGATCAAAAAT 4980
NatalRGN           ATCCTAGACAAGTGTGGGAGAGTGATAGGACTTTATGGCAATGGGGTCGTGATCAAAAAT 4980
HPF2013            ATCCTAGACAAGTGTGGGAGAGTGATAGGACTTTATGGCAATGGGGTCGTGATCAAAAAT 4980
SPH2015            ATCCTAGACAAGTGTGGGAGAGTGATAGGACTTTATGGCAATGGGGTCGTGATCAAAAAT 4980
Haiti2014          ATCCTAGACAAGTGTGGGAGAGTGATAGGACTTTATGGCAATGGGGTCGTGATCAAAAAT 4980
PRVABC59           ATCCTAGACAAGTGTGGGAGAGTGATAGGACTTTATGGCAATGGGGTCGTGATCAAAAAC 4980
BeH819015          ATCCTAGACAAGTGTGGGAGAGTGATAGGACTTTATGGCAATGGGGTCGTGATCAAAAAT 4980
Z1106033           ATCCTAGACAAGTGTGGGAGAGTGATAGGACTTTATGGCAATGGGGTCGTGATCAAAAAT 4980
BEH819966          ATCCTAGACAAGTGTGGGAGAGTGATAGGACTTTATGGCAATGGGGTCGTGATCAAAAAT 4980
SSABr              ATCCTAGACAAGTGTGGGAGAGTGATAGGACTTTATGGCAATGGGGTCGTGATCAAAAAT 4980
Beh815744          ATCCTAGACAAGTGTGGGAGAGTGATAGGACTTTATGGCAATGGGGTCGTGATCAAAAAT 4980
BEH818995          ATCCTAGACAAGTGTGGGAGAGTGATAGGACTTTATGGCAATGGGGTCGTGATCAAAAAT 4980
IbH30656           ATCCTAGACAAATGCGGAAGAGTGATAGGACTTTATGGCAATGGGGTTGTGATCAAGAAT 4962
ArD128000          ATCCTAGACAAATGCGGAAGAGTGATAGGACTTTATGGCAATGGGGTTGTGATCAAGAAT 4980
ArD7117            ATCCTAGACAAATGCGGAAGAGTGATAGGACTTTATGGCAATGGGGTTGTGATCAAGAAT 4980
ARD_41519          ATCCTAGACAAATGCGGAAGAGTGATAGGACTTTATGGCAATGGGGTTGTGATCAAGAAT 4980
ARD157995          ATCCTAGACAAATGTGGAAGAGTGATAGGACTCTATGGCAATGGGGTTGTGATCAAGAAT 4980
MR_766             ATCCTAGACAAATGTGGAAGAGTGATAGGACTCTATGGCAATGGGGTTGTGATCAAGAAT 4968
ARD158084          ATCCTAGACAAATGTGGAAGAGTGATAGGACTCTATGGCAATGGGGTTGTGATCAAGAAT 4980
ArB1362            ATCCTAGACAAATGCGGAAGAGTGATAGGACTTTATGGCAATGGGGTTGTGATCAAGAAT 4980
ARB13565           ATCCTAGACAAATGCGGAAGAGTGATAGGACTTTATGGCAATGGGGTTGTGATCAAGAAT 4980
ARB7701            ATCCTAGACAAATGCGGAAGAGTGATAGGACTTTATGGCAATGGGGTTGTGATCAAGAAT 4980
ARB15076           ATCCTAGACAAATGCGGAAGAGTGATAGGACTTTATGGCAATGGGGTTGTGATCAAGAAT 4962
                   ******** ** ** ** ************** ***** ******** ***** ** **

P6-740             GGAAGTTATGTTAGTGCCATCACCCAAGGGAGGAGGGAGGAAGAGACTCCTGTTGAATGC 5040
CPC0740            GGGAGTTATGTTAGTGCCATCACCCAAGGGAGGAGGGAGGAAGAGACTCCTGTTGAGTGC 5040
YAP2007            GGGAGTTATGTTAGTGCCATCACCCAAGGGAGGAGGGAGGAAGAGACTCCTGTTGAGTGC 5040
FSS13025           GGGAGTTACGTTAGTGCCATCACCCAAGGGAGGAGGGAGGAAGAGACTCCTGTTGAGTGC 5040
PLCal_ZV           GGGAGTTATGTTAGTGCCATCACCCAAGGGAGGAGGGAGGAAGAGACTCCTGTTGAGTGC 4929
SV0127/14          GGGAGTTATGTCAGTGCCATCACCCAAGGGAGGAGGGAGGAAGAGACTCCTGTTGAGTGC 5040
8375               GGGAGTTATGTTAGTGCCATCACCCAAGGGAGGAGGGAGGAAGAGACTCCTGTTGAGTGC 5040
103344             GGGAGTTATGTTAGTGCCATCACCCAAGGGAGGAGGGAGGAAGAGACTCCTGTTGAGTGC 5040
BrasilZKV2015      GGGAGTTATGTTAGTGCCATCACCCAAGGGAGGAGGGAGGAAGAGACTCCTGTTGAGTGC 5040
GD01               GGGAGTTATGTTAGTGCCATCACCCAAGGGAGGAGGGAGGAAGAGACTCCTGTTGAGTGC 5040
Martinique         GGGAGTTATGTTAGTGCCATCACCCAAGGGAGGAGGGAGGAAGAGACTCCTGTTGAGTGC 5040
NatalRGN           GGGAGTTATGTTAGTGCCATCACCCAAGGGAGGAGGGAGGAAGAGACTCCTGTTGAGTGC 5040
HPF2013            GGGAGTTATGTTAGTGCCATCACCCAAGGGAGGAGGGAGGAAGAGACTCCTGTTGAGTGC 5040
SPH2015            GGGAGTTATGTTAGTGCCATCACCCAAGGGAGGAGGGAGGAAGAGACTCCTGTTGAGTGC 5040
```

```
Haiti2014        GGGAGTTATGTTAGTGCCATCACCCAAGGGAGGAGGGAGGAAGAGACTCCTGTTGAGTGC 5040
PRVABC59         GGGAGTTATGTTAGTGCCATCACCCAAGGGAGGAGGGAGGAAGAGACTCCTGTTGAGTGC 5040
BeH819015        GGGAGTTATGTTAGTGCCATCACCCAAGGGAGGAGGGAAGAAGAGACTCCTGTTGAGTGC 5040
Z1106033         GGGAGTTATGTTAGTGCCATCACCCAAGGGAGGAGGGAGGAAGAGACTCCTGTTGAGTGC 5040
BEH819966        GGGAGTTATGTTAGTGCCATCACCCAAGGGAGGAGGGAGGAAGAGACTCCTGTTGAGTGC 5040
SSABr            GGGAGTTATGTTAGTGCCATCACCCAAGGGAGGAGGGAGGAAGAGACTCCTGTTGAGTGC 5040
Beh815744        GGGAGTTATGTTAGTGCCATCACCCAAGGGAGGAGGGAGGAAGAGACTCCTGTTGAGTGC 5040
BEH818995        GGGAGTTATGTTAGTGCCATCACCCAAGGGAGGAGGGAGGAAGAGACTCCTGTTGAGTGC 5040
IbH30656         GGAAGCTATGTTAGTGCCATAACCCAGGGAAAAAGGGAGGAGGAGACTCCGGTTGAGTGC 5022
ArD128000        GGAAGCTATGTCAGTGCAATAACCCAGGGAAAAAGGGAGGAGGAGGCCCCAGTTGAGTGC 5040
ArD7117          GGAAGCTATGTTAGTGCCATAACCCAGGGAAAAAGGGAGGAGGAGGCTCCAGTTGAGTGC 5040
ARD_41519        GGAAGCTATGTTAGTGCAATAACCCAGGGAAAAAGGGAGGAGGAGGCTCCAGTTGAGTGC 5040
ARD157995        GGAAGCTATGTTAGTGCTATAACCCAGGGAAAGAGGGAGGAGGAGACTCCGGTTGAATGT 5040
MR_766           GGAAGCTATGTTAGTGCTATAACCCAGGGAAAGAGGGAGGAGGAGACTCCGGTTGAATGT 5028
ARD158084        GGAAGCTATGTTAGTGCTATAACCCAGGGAAAGAGGGAGGAGGAGACTCCGGTTGAATGT 5040
ArB1362          GGAAGCTATGTTAGTGCCATAACCCAGGGAAAGAGGGAGGAGGAGACTCCGGTTGAATGT 5040
ARB13565         GGAAGCTATGTTAGTGCCATAACCCAGGGAAAGAGGGAGGAGGAGACTCCGGTTGAATGT 5040
ARB7701          GGAAGCTATGTTAGTGCCATAACCCAGGGAAAGAGGGAGGAGGAGACTCCGGTTGAATGT 5040
ARB15076         GGAAGCTATGTTAGTGCCATAACCCAGGGAAAGAGGGAGGAGGAGACTCCGGTTGAATGT 5022
                 ** ** ** ** ***** ** ***** ** *  ***** ** *** * ** ***** **

P6-740           TTCGAACCTTCGATGCTGAAGAAGAAGCAGCTAACTGTCTTGGATCTGCATCCTGGAGCT 5100
CPC0740          TTCGAGCCTTCGATGCTGAAGAAGAAGCAGCTAACTGTCTTAGACCTGCATCCTGGAGCC 5100
YAP2007          TTCGAGCCTTCGATGTTGAAGAAGAAGCAGCTAACTGTCTTAGACCTGCATCCTGGAGCT 5100
FSS13025         TTCGAGCCTTCGATGCTGAAGAAGAAGCAGCTAACTGTCTTAGACTTGCATCCTGGAGCT 5100
PLCal_ZV         TTCGAGCCTTCGATGCTGAAGAAGAAGCAGCTAACTGTCTTAGACTTGCATCCTGGAGCT 4989
SV0127/14        TTCGAGCCTTCGATGCTGAAGAAGAAGCAGCTAACTGTCTTAGACTTGCATCCTGGAGCT 5100
8375             TTCGAGCCTTCGATGCTGAAGAAGAAGCAGCTAACTGTCTTAGACTTACATCCTGGAGCT 5100
103344           TTCGAGCCTTCGATGCTGAAGAAGAAGCAGCTAACTGTCTTAGACTTACATCCTGGAGCT 5100
BrasilZKV2015    TTCGAGCCTTCGATGCTGAAGAAGAAGCAGCTAACTGTCTTAGACTTGCATCCTGGAGCT 5100
GD01             TTCGAGCCTTCGATGCTGAAGAAGAAGCAGCTAACTGTCTTAGACTTGCATCCTGGAGCT 5100
Martinique       TTCGAGCCTTCGATGCTGAAGAAGAAGCAGCTAACTGTCTTAGACTTGCATCCTGGAGCT 5100
NatalRGN         TTCGAGCCTTCGATGCTGAAGAAGAAGCAGCTAACTGTCTTAGACTTGCATCCTGGAGCT 5100
HPF2013          TTCGAGCCTTCGATGCTGAAGAAGAAGCAGCTAACTGTCTTAGACTTGCATCCTGGAGCT 5100
SPH2015          TTCGAGCCTTCGATGCTGAAGAAGAAGCAGCTAACTGTCTTAGACTTGCATCCTGGAGCT 5100
Haiti2014        TTCGAGCCTTCGATGCTGAAGAAGAAGCAGCTAACTGTCTTAGACTTGCATCCTGGAGCT 5100
PRVABC59         TTCGAGCCCTCGATGCTGAAGAAGAAGCAGCTAACTGTCTTAGACTTGCATCCTGGAGCT 5100
BeH819015        TTCGAGCCCTCGATGCTGAAGAAGAAGCAGCTAACTGTCTTAGACTTGCATCCTGGAGCT 5100
Z1106033         TTCGAGCCTTCGATGCTGAAGAAGAAGCAGCTAACTGTCTTAGACTTGCATCCTGGAGCT 5100
BEH819966        TTCGAGCCTTCGATGCTGAAGAAGAAGCAGCTAACTGTCTTAGACTTGCATCCTGGAGCT 5100
SSABr            TTCGAGCCTTCGATGCTGAAGAAGAAGCAGCTAACTGTCTTAGACTTGCATCCTGGAGCT 5100
Beh815744        TTCGAGCCTTCGATGCTGAAGAAGAAGCAGCTAACTGTCTTAGACTTGCATCCTGGAGCT 5100
BEH818995        TTCGAGCCTTCGATGCTGAAGAAGAAGCAGCTAACTGTCTTAGACTTGCATCCTGGAGCT 5100
IbH30656         TTTGAACCCTCGATGCTGAGGAAGAAGCAGCTAACAGTCTTGGATCTGCATCCAGGAGCC 5082
ArD128000        TTTGAACCCTCGATGCTGAGGAAGAAGCAGCTAACAGTCTTGGATCTGCATCCAGGAGCC 5100
ArD7117          TTTGAACCCTCGATGCTGAGGAAGAAGCAGCTAACAGTCTTGGATCTGCATCCAGGAGCC 5100
ARD_41519        TTTGAACCTTCGATGCTGAGGAAGAAGCAGCTAACAGTCTTGGATCTGCATCCAGGAGCC 5100
ARD157995        TTCGAACCCTCGATGCTGAAGAAGAAGCAGCTAACTGTCCTGGATCTGCATCCAGGAGCC 5100
MR_766           TTCGAACCCTCGATGCTGAAGAAGAAGCAGCTAACTGTCTTGGATCTGCATCCAGGAGCC 5088
ARD158084        TTCGAACCCTCGATGCTGAAGAAGAAGCAGCTAACTGTCCTGGATCTGCATCCAGGAGCC 5100
ArB1362          TTCGAACCCTCGATGCTGAAGAAGAAGCAGCTAACTGTCCTGGACCTGCATCCAGGAGCC 5100
ARB13565         TTCGAACCCTCGATGCTGAAGAAGAAGCAGCTAACTGTCCTGGATCTGCATCCAGGAGCC 5100
ARB7701          TTCGAACCCTCGATGCTGAAGAAGAAGCAGCTAACTGTCCTGGATCTGCATCCAGGAGCC 5100
ARB15076         TTCGAACCCTCGATGCTGAAGAAGAAGCAGCTAACTGTCCTGGATCTGCATCCAGGAGCC 5082
                 ** ** ** ****** *** *************** *** * **  * ***** *****

P6-740           GGGAAAACCAGGAGAGTTCTTCCTGAAATAGTCCGTGAAGCCATAAAAACAAGACTCCGC 5160
CPC0740          GGGAAAACCAGGAGAGTTCTTCCTGAAATAGTCCGTGAAGCCATAAAAACAAGACTCCGT 5160
YAP2007          GGGAAAACCAGGAGAGTTCTTCCTGAAATAGTCCGTGAAGCCATAAAAACAAGACTCCGT 5160
FSS13025         GGGAAAACCAGGAGAGTTCTTCCTGAAATAGTCCGTGAAGCCATAAAAACAAGACTCCGC 5160
PLCal_ZV         GGGAAAACCAGGAGAGTTCTTCCTGAAATAGTCCGTGAAGCCATAAAAACAAGACTCCGT 5049
SV0127/14        GGGAAAACCAGGAGAGTTCTTCCTGAAATAGTCCGTGAAGCCATAAAAACGAGACTCCGT 5160
8375             GGGAAAACCAGGAGAGTTCTTCCTGAAATAGTCCGTGAAGCCATAAAAACAAGACTCCGT 5160
103344           GGGAAAACCAGGAGAGTTCTTCCTGAAATAGTCCGTGAAGCCATAAAAACAAGACTCCGT 5160
BrasilZKV2015    GGGAAAACCAGGAGAGTTCTTCCTGAAATAGTCCGTGAAGCCATAAAAACAAGACTCCGT 5160
GD01             GGGAAAACCAGGAGAGTTCTTCCTGAAATAGTCCGTGAAGCCATAAAAACAAGACTCCGT 5160
Martinique       GGGAAAACCAGGAGAGTTCTTCCTGAAATAGTCCGTGAAGCCATAAAAACAAGACTCCGT 5160
NatalRGN         GGGAAAACCAGGAGAGTTCTTCCTGAAATAGTCCGTGAAGCCATAAAAACAAGACTCCGT 5160
```

```
HPF2013             GGGAAAACCAGGAGAGTTCTTCCTGAAATAGTCCGTGAAGCCATAAAAACAAGACTCCGT 5160
SPH2015             GGGAAAACCAGGAGAGTTCTTCCTGAAATAGTCCGTGAAGCCATAAAAACAAGACTCCGT 5160
Haiti2014           GGGAAAACCAGGAGAGTTCTTCCTGAAATAGTCCGTGAAGCCATAAAAACAAGACTCCGT 5160
PRVABC59            GGGAAAACCAGGAGAGTTCTTCCTGAAATAGTCCGTGAAGCCATAAAAACAAGACTCCGT 5160
BeH819015           GGGAAAACCAGGAGAGTTCTTCCTGAAATAGTCCGTGAAGCCATAAAAACAAGACTCCGT 5160
Z1106033            GGGAAAACCAGGAGAGTTCTTCCTGAAATAGTCCGTGAAGCCATAAAAACAAGACTCCGT 5160
BEH819966           GGGAAAACCAGGAGAGTTCTTCCTGAAATAGTCCGTGAAGCCATAAAAACAAGACTCCGT 5160
SSABr               GGGAAAACCAGGAGAGTTCTTCCTGAAATAGTCCGTGAAGCCATAAAAACAAGACTCCGT 5160
Beh815744           GGGAAAACCAGGAGAGTTCTTCCTGAAATAGTCCGTGAAGCCATAAAAACAAGACTCCGC 5160
BEH818995           GGGAAAACCAGGAGAGTTCTTCCTGAAATAGTCCGTGAAGCCATAAAAACAAGACTCCGC 5160
IbH30656            GGGAAAACCAGGAGGGTTCTTCCTGAAATAGTCCGTGAAGCCATAAAGAAGAGACTTCGC 5142
ArD128000           GGAAAAACCAGGAGGGTTCTTCCTGAAATAGTCCGTGAAGCCATAAAAAAGAGACTCCGG 5160
ArD7117             GGGAAAACCAGGAGGGTTCTTCCTGAAATAGTCCGTGAAGCCATAAAGAAGAGACTTCGC 5160
ARD_41519           GGAAAAACCAGGAGGGTCCTTCCTGAAATAGTCCGTGAAGCCATAAAGAAGAGACTTCGC 5160
ARD157995           GGAAAAACCAGGAGAGTTCTTCCTGAAATAGTCCGTGAAGCCATAAAAAAGAGACTCCGG 5160
MR_766              GGAAAAACCAGGAGAGTTCTTCCTGAAATAGTCCGTGAAGCCATAAAAAAGAGACTCCGG 5148
ARD158084           GGAAAAACCAGGAGAGTTCTTCCTGAAATAGTCCGTGAAGCCATAAAAAAGAGACTCCGG 5160
ArB1362             GGAAAGACTAGGAGAGTTCTTCCTGAAATAGTCCGTGAAGCCATAAAAAAGAGACTCCGC 5160
ARB13565            GGAAAGACTAGGAGAGTTCTTCCTGAAATAGTCCGTGAAGCCATAAAAAAGAGACTCCGC 5160
ARB7701             GGAAAGACTAGGAGAGTTCTTCCTGAAATAGTCCGTGAAGCCATAAAAAAGAGACTCCGC 5160
ARB15076            GGAAAGACTAGGAGAGTTCTTCCTGAAATAGTCCGTGAAGCCATAAAAAAGAGACTCCGC 5142
                    ** ** ** ***** ** ***************************** *  ***** **

P6-740              ACGGTGATCCTGGCTCCAACCAGGGTTGTCGCTGCTGAAATGGAGGAAGCCCTTAGAGGG 5220
CPC0740             ACTGTGATCTTAGCTCCAACCAGGGTCGTCGCTGCTGAAATGGAGGAAGCCCTTAGAGGG 5220
YAP2007             ACTGTGATCTTAGCTCCAACCAGGGTTGTCGCTGCTGAAATGGAGGAAGCCCTTAGAGGG 5220
FSS13025            ACTGTGATCTTAGCTCCAACCAGGGTTGTCGCTGCTGAAATGGAGGAAGCCCTTAGAGGG 5220
PLCal_ZV            ACTGTGATCTTAGCTCCAACCAGGGTTGTCGCTGCTGAAATGGAGGAAGCCCTTAGAGGG 5109
SV0127/14           ACTGTGATCTTAGCTCCAACCAGGGTTGTCGCTGCTGAAATGGAGGAAGCCCTTAGAGGG 5220
8375                ACTGTGATCTTAGCTCCAACCAGGGTTGTCGCTGCTGAAATGGAGGAGGCCCTTAGAGGG 5220
103344              ACTGTGATCTTAGCTCCAACCAGGGTTGTCGCTGCTGAAATGGAGGAGGCCCTTAGAGGG 5220
BrasilZKV2015       ACTGTGATCTTAGCTCCAACCAGGGTTGTCGCTGCTGAAATGGAGGAAGCCCTTAGAGGG 5220
GD01                ACTGTGATCTTAGCTCCAACCAGGGTTGTCGCTGCTGAAATGGAGGAGGCCCTTAGAGGG 5220
Martinique          ACTGTGATCTTAGCTCCAACCAGGGTTGTCGCTGCTGAAATGGAGGAAGCCCTTAGAGGG 5220
NatalRGN            ACTGTGATCTTAGCTCCAACCAGGGTTGTCGCTGCTGAAATGGAGGAAGCCCTTAGAGGG 5220
HPF2013             ACTGTGATCTTAGCTCCAACCAGGGTTGTCGCTGCTGAAATGGAGGAAGCCCTTAGAGGG 5220
SPH2015             ACTGTGATCTTAGCTCCAACCAGGGTTGTCGCTGCTGAAATGGAGGAAGCCCTTAGAGGG 5220
Haiti2014           ACTGTGATCTTAGCTCCAACCAGGGTTGTCGCTGCTGAAATGGAGGAAGCCCTTAGAGGG 5220
PRVABC59            ACTGTGATCTTAGCTCCAACCAGGGTTGTCGCTGCTGAAATGGAGGAGGCCCTTAGAGGG 5220
BeH819015           ACTGTGATCTTAGCTCCAACCAGGGTTGTCGCTGCTGAAATGGAGGAGGCCCTTAGAGGG 5220
Z1106033            ACTGTGATCTTAGCTCCAACCAGGGTTGTCGCTGCTGAAATGGAGGAGGCCCTTAGAGGG 5220
BEH819966           ACTGTGATCTTAGCTCCAACCAGGGTTGTCGCTGCTGAAATGGAGGAGGCCCTTAGAGGG 5220
SSABr               ACTGTGATCTTAGCTCCAACCAGGGTTGTCGCTGCTGAAATGGAGGAGGCCCTTAGAGGG 5220
Beh815744           ACCGTGATCTTAGCTCCAACCAGGGTTGTCGCTGCTGAAATGGAGGAGGCCCTTAGAGGG 5220
BEH818995           ACCGTGATCTTAGCTCCAACCAGGGTTGTCGCTGCTGAAATGGAGGAGGCCCTTAGAGGG 5220
IbH30656            ACAGTGATCTTAGCACCAACCAGGGTTGTTGCTGCTGAGATGGAGGAAGCCCTAAGAGGA 5202
ArD128000           ACAGTGATCTTGGCACCAACTAGGGTTGTCGCTGCTGAGATGGAGGAGGCCTTGAGAGGA 5220
ArD7117             ACATTGATCCTAGCACCAACCAGGGTCGTTGCTGCTGAGATGGAGGAAGCCCTAAGAGGA 5220
ARD_41519           ACAGTGATCCTAGCACCAACCAGGGTCGTTGCTGCCGAGATGGAGGAAGCCCTAAGAGGA 5220
ARD157995           ACAGTGATCTTGGCACCAACTCCAGTTGAGAGATCATACTCAAGGTGGTCCTTGTGGCCA 5220
MR_766              ACAGTGATCTTGGCACCAACTAGGGTTGTCGCTGCTGAGATGGAGGAGGCCTTGAGAGGA 5208
ARD158084           ACAGTGATCTTGGCACCAACTAGGGTTGTCGCTGCTGAGATGGAGGAAGCCTTGAGAGGA 5220
ArB1362             ACAGTGATCTTGGCTCCAACCAGGGTTGTCGCTGCTGAGATGGAGGAAGCCTTGAGAGGA 5220
ARB13565            ACAGTGATTTTGGCTCCAACCAGGGTTGTCGCTGCTGAGATGGAGGAAGCCTTGAGAGGA 5220
ARB7701             ACAGTGATTTTGGCTCCAACCAGGGTTGTCGCTGCTGAGATGGAGGAAGCCTTGAGAGGA 5220
ARB15076            ACAGTGATTTTGGCTCCAACCAGGGTTGTCGCTGCTGAGATGGAGGAAGCCTTGAGAGGA 5202
                    **  ****  * ** *****    ** *      *  *      *    ** *  *

P6-740              CTTCCAGTGCGTTACATGACAACAGCAGTTAATGTCACCCACTCTGGGACAGAAATCGTT 5280
CPC0740             CTTCCAGTTCGTTATATGACAACAGCAGTCAATGTCACCCATTCTGGGACAGAAATCGTT 5280
YAP2007             CTTCCAGTGCGTTATATGACAACAGCAGTCAATGTCACCCATTCTGGGACAGAAATTGTT 5280
FSS13025            CTTCCAGTGCGTTATATGACAACAGCAGTCAATGTCACCCATTCTGGGACAGAAATCGTT 5280
PLCal_ZV            CTTCCAGTGCGTTATATGACAACAGCAGTCAATGTCACCCATTCTGGGACAGAAATCGTT 5169
SV0127/14           CTTCCAGTGCGTTATATGACAACAGCAGTCAATGTCACCCATTCTGGGACAGAAATCGTT 5280
8375                CTTCCAGTGCGTTATATGACAACAGCAGTCAATGTCACCCACTCTGGAACAGAAATCGTC 5280
103344              CTTCCAGTGCGTTATATGACAACAGCAGTCAATGTCACCCACTCTGGAACAGAAATCGTC 5280
BrasilZKV2015       CTTCCAGTGCGTTATATGACAACAGCAGTCAATGTCACCCACTCTGGAACAGAAATCGTC 5280
GD01                CTTCCAGTGCGTTATATGACAACAGCAGTCAATGTCACCCACTCTGGAACAGAAATCGTC 5280
```

```
Martinique         CTTCCAGTGCGTTATATGACAACAGCAGTCAATGTCACCCACTCTGGAACAGAAATCGTC 5280
NatalRGN           CTTCCAGTGCGTTATATGACAACAGCAGTCAATGTCACCCACTCTGGAACAGAAATCGTC 5280
HPF2013            CTTCCAGTGCGTTATATGACAACAGCAGTCAATGTCACCCACTCTGGAACAGAAATCGTC 5280
SPH2015            CTTCCAGTGCGTTATATGACAACAGCAGTCAATGTCACCCACTCTGGAACAGAAATCGTC 5280
Haiti2014          CTTCCAGTGCGTTATATGACAACAGCAGTCAATGTCACCCACTCTGGAACAGAAATCGTC 5280
PRVABC59           CTTCCAGTGCGTTATATGACAACAGCAGTCAATGTCACCCACTCTGGAACAGAAATCGTC 5280
BeH819015          CTTCCAGTGCGTTATATGACAACAGCAGTCAATGTCACCCACTCTGGAACAGAAATCGTC 5280
Z1106033           CTTCCAGTGCGTTATATGACAACAGCAGTCAATGTCACCCACTCTGGAACAGAAATCGTC 5280
BEH819966          CTTCCAGTGCGTTATATGACAACAGCAGTCAATGTCACCCACTCTGGAACAGAAATCGTC 5280
SSABr              CTTCCAGTGCGTTATATGACAACAGCAGTCAATGTCACCCACTCTGGAACAGAAATCGTC 5280
Beh815744          CTTCCAGTGCGTTATATGACAACAGCAGTCAATGTCACCCACTCTGGAACAGAAATCGTC 5280
BEH818995          CTTCCAGTGCGTTATATGACAACAGCAGTCAATGTCACCCACTCTGGAACAGAAATCGTC 5280
IbH30656           CTTCCGGTGCGTTACATGACAACAGCAGTCAACGTCACCCATTCTGGGACAGAAATCGTT 5262
ArD128000          CTTCCGGTGCGTTACATGACAACAGCAGTCAACGTCACCCATTCTGGGACAGAAATCGTT 5280
ArD7117            CTTCAGGTGCGTTACATGACAACAGCAGTCAACGTCACCCATTCTGGGACAGAAATCGTT 5280
ARD_41519          CTTCCGGTGCGTTACATGACAACAGCAGTCAACGTCACCCACTCTGGGACAGAAATCGTC 5280
ARD157995          TCTGTGGCACGTACCCAGACAACAGCAGTCAACGTCACCCATTCTGGGACAGAAATCGTT 5280
MR_766             CTTCCGGTGCGTTACATGACAACAGCAGTCAACGTCACCCATTCTGGGACAGAAATCGTT 5268
ARD158084          CTTCCGGTGCGTTACATGACAACAGCAGTCAACGTCACCCATTCTGGGACAGAAATCGTT 5280
ArB1362            CTTCcgGTgCGTTACATGACAACAGCAGTCAACGTCACCCATTCTGGGACAGAAATCGTT 5280
ARB13565           CTTCCGGTGCGTTACATGACAACAGCAGTTAACGTCACCCACTCTGGGACAGAAATCGTT 5280
ARB7701            CTTCCGGTGCGTTACATGACAACAGCAGTTAACGTCACCCACTCTGGGACAGAAATCGTT 5280
ARB15076           CTTCCGGTGCGTTACATGACAACAGCAGTTAACGTCACCCACTCTGGGACAGAAATCGTT 5262
                     *   *  ***     ************ ** ******** ***** ******** **

P6-740             GATTTAATGTGCCATGCCACCTTCACTTCACGCCTACTACAACCCATTAGAGTCCCCAAC 5340
CPC0740            GACTTAATGTGCCATGCTACCTTCACTTCACGCCTACTACAACCAATCAGAGTCCCCAAC 5340
YAP2007            GACTTAATGTGCCATGCCACCTTCACTTCACGTCTACTACAACCAATCAGAGTCCCCAAC 5340
FSS13025           GACTTAATGTGCCATGCCACCTTCACTTCACGTCTACTACAGCCAATCAGAGTCCCCAAC 5340
PLCal_ZV           GACTTAATGTGCCATGCCACCTTCACTTCACGTCTACTACAGCCAATCAGAGTCCCCAAC 5229
SV0127/14          GACTTAATGTGCCATGCCACCTTCACTTCACGTCTACTACAGCCAATCAGAGTCCCCAAC 5340
8375               GACTTAATGTGCCATGCCACCTTCACTTCACGTCTACTACAGCCAATCAGAGTCCCCAAC 5340
103344             GACTTAATGTGCCATGCCACCTTCACTTCACGTCTACTACAGCCAATCAGAGTCCCCAAC 5340
BrasilZKV2015      GACTTAATGTGCCATGCCACCTTCACTTCACGTCTACTACAGCCAATCAGAGTCCCCAAC 5340
GD01               GACTTAATGTGCCATGCCACCTTCACTTCACGTCTACTACAGCCAATTAGAGTCCCCAAC 5340
Martinique         GACTTAATGTGCCATGCCACCTTCACTTCACGTCTACTACAGCCAATCAGAGTCCCCAAC 5340
NatalRGN           GACTTAATGTGCCATGCCACCTTCACTTCACGTCTACTACAGCCAATCAGAGTCCCCAAC 5340
HPF2013            GACTTAATGTGCCATGCCACCTTCACTTCACGTCTACTACAGCCAATCAGAGTCCCCAAC 5340
SPH2015            GACTTAATGTGCCATGCCACCTTCACTTCACGTCTACTACAGCCAATCAGAGTCCCCAAC 5340
Haiti2014          GACTTAATGTGCCATGCCACCTTCACTTCACGTCTACTACAGCCAATCAGAGTCCCCAAC 5340
PRVABC59           GACTTAATGTGCCATGCCACCTTCACTTCACGTCTACTACAGCCAATCAGAGTCCCCAAC 5340
BeH819015          GACTTAATGTGCCATGCCACCTTCACTTCACGTCTACTACAGCCAATCAGAGTCCCCAAC 5340
Z1106033           GACTTAATGTGCCATGCCACCTTCACTTCGCGTCTACTACAGCCAATCAGAGTCCCCAAC 5340
BEH819966          GACTTAATGTGCCATGCCACCTTCACTTCACGTCTACTACAGCCAATCAGAGTCCCCAAC 5340
SSABr              GACTTAATGTGCCATGCCACCTTCACTTCACGTCTACTACAGCCAATCAGAGTCCCCAAC 5340
Beh815744          GACTTAATGTGCCATGCCACCTTCACTTCACGTCTACTACAGCCAATCAGAGTCCCCAAC 5340
BEH818995          GACTTAATGTGCCATGCCACCTTCACTTCACGTCTACTACAGCCAATCAGAGTCCCCAAC 5340
IbH30656           GATTTGATGTGCCATGCCACCTTCACTTCACGCCTACTACAACCAATCAGAGTCCCCAAC 5322
ArD128000          GATTTGATGTGCCATGCCACTTTCACTTCACGCTTACTACAACCCATCAGAGTCCCTAAT 5340
ArD7117            GATTTGATGTGCCATGCCACTTTCACTTCACGCTTACTACAACCCATCAGAGTCCCTAAT 5340
ARD_41519          GATTTGATGTGCCATGCCACCTTCACTTCACGCCTACTACAACCCATCAGAGTCCCCAAC 5340
ARD157995          GATTTGATGTGCCATGCCACTTTCACTTCACGCTTACTACAACCCATCAGAGTCCCTAAT 5340
MR_766             GATTTGATGTGCCATGCCACTTTCACTTCACGCTTACTACAACCCATCAGAGTCCCTAAT 5328
ARD158084          GATTTGATGTGCCATGCCACTTTCACTTCACGCTTACTACAACCCATCAGAGTCCCTAAT 5340
ArB1362            GATTTGATGTGCCATGCCACTTTCACTTCACGCTTACTACAACCCATCAGAGTCCCTAAT 5340
ARB13565           GATTTGATGTGCCATGCCACCTTCACCTCACGCCTATTACAACCCATCAGAGTCCCTAAT 5340
ARB7701            GATTTGATGTGCCATGCCACCTTCACCTCACGCCTATTACAACCCATCAGAGTCCCTAAT 5340
ARB15076           GATTTGATGTGCCATGCCACCTTCACTTCACGCTTATTACAACCCATCAGAGTCCCTAAT 5322
                   ** ** *********** ** ***** ** **  ** **** ** ** ******** **

P6-740             TACAATCTTTACATTATGGATGAGGCCCACTTCACAGATCCCTCAAGTATAGCAGCAAGA 5400
CPC0740            TATAATTTGTATATTATGGATGAGGCCCACTTCACAGATCCCTCAAGTATAGCAGCAAGA 5400
YAP2007            TATAATCTGTATATTATGGACGAGGCCCACTTCACAGATCCCTCAAGTATAGCAGCAAGA 5400
FSS13025           TATAATCTGTATATTATGGATGAGGCCCACTTCACAGATCCCTCAAGTATAGCAGCAAGA 5400
PLCal_ZV           TATAATCTGTATATTATGGATGAGGCCCACTTCACAGATCCCTCAAGTATAGCAGCAAGA 5289
SV0127/14          TATAATCTGTATATTATGGATGAGGCCCACTTCACAGATCCCTCAAGTATAGCAGCAAGA 5400
8375               TATAATCTGTATATTATGGATGAGGCCCACTTCACAGATCCCTCAAGTATAGCAGCAAGA 5400
103344             TATAATCTGTATATTATGGATGAGGCCCACTTCACAGATCCCTCAAGTATAGCAGCAAGA 5400
```

```
BrasilZKV2015       TATAATCTGTATATTATGGATGAGGCCCACTTCACAGATCCCTCAAGCATAGCAGCAAGA 5400
GD01                TATAATCTGTATATTATGGATGAGGCCCACTTCACAGATCCCTCAAGTATAGCAGCAAGA 5400
Martinique          TATAATCTGTATATTATGGATGAGGCCCACTTCACAGATCCCTCAAGTATAGCAGCAAGA 5400
NatalRGN            TATAATCTGTATATTATGGATGAGGCCCACTTCACAGATCCCTCAAGTATAGCAGCAAGA 5400
HPF2013             TATAATCTGTATATTATGGATGAGGCCCACTTCACAGATCCCTCAAGTATAGCAGCAAGA 5400
SPH2015             TATAATCTGTATATTATGGATGAGGCCCACTTCACAGATCCCTCAAGTATAGCAGCAAGA 5400
Haiti2014           TATAATCTGTATATTATGGATGAGGCCCACTTCACAGATCCCTCAAGTATAGCAGCAAGA 5400
PRVABC59            TATAATCTGTATATTATGGATGAGGCCCACTTCACAGATCCCTCAAGTATAGCAGCAAGA 5400
BeH819015           TATAATCTGTATATTATGGATGAGGCCCACTTCACAGATCCCTCAAGTATAGCAGCAAGA 5400
Z1106033            TATAATCTGTATATTATGGATGAGGCCCACTTCACAGATCCCTCAAGTATAGCAGCAAGA 5400
BEH819966           TATAATCTGTATATTATGGATGAGGCCCACTTCACAGATCCCTCAAGTATAGCAGCAAGA 5400
SSABr               TATAATCTGTATATTATGGATGAGGCCCACTTCACAGATCCCTCAAGTATAGCAGCAAGA 5400
Beh815744           TATAATCTGTATATTATGGATGAGGCCCACTTCACAGATCCCTCAAGTATAGCAGCAAGA 5400
BEH818995           TATAATCTGTATATTATGGATGAGGCCCACTTCACAGATCCCTCAAGTATAGCAGCAAGA 5400
IbH30656            TACAACCTTTATATCATGGATGAGGCTCATTTCACAGATCCTTCAAGCATAGCTGCAAGA 5382
ArD128000           TACAATCTCAACATCATGGATGAAGCCCACTTCACAGACCCCTCAAGTATAGCTGCAAGA 5400
ArD7117             TACAATCTCAACATCATGGATGAAGCCCACTTCACAGACCCCTCAAGTATAGCTGCAAGA 5400
ARD_41519           TACAACCTTTACATCATGGATGAAGCTCATTTCACAGATCCCTCAAGCATAGCTGCACGA 5400
ARD157995           TACAATCTCAACATCATGGATGAAGCCCACTTCACAGACCCCTCAAGTATAGCTGCAAGA 5400
MR_766              TACAATCTCAACATCATGGATGAAGCCCACTTCACAGACCCCTCAAGTATAGCTGCAAGA 5388
ARD158084           TACAATCTCAACATCATGGATGAAGCCCACTTCACAGACCCCTCAAGTATAGCTGCAAGA 5400
ArB1362             TACAATCTCAACATCATGGATGAAGCCCACTTCACAGACCCCTCAAGTATAGCTGCAAGA 5400
ARB13565            TACAATCTCTACATCATGGATGAAGCCCATTTCACAGACCCCTCAAGTATAGCTGCAAGA 5400
ARB7701             TACAATCTCTACATCATGGATGAAGCCCATTTCACAGACCCCTCAAGTATAGCTGCAAGA 5400
ARB15076            TACAATCTCTACATCATGGATGAAGCCCATTTCACAGACCCCTCAAGTATAGCTGCAAGA 5382
                    ** **  *  * ** ***** ** ** ** ******** ** ***** ***** *** **

P6-740              GGATACATATCAACAAGGGTTGAGATGGGCGAGGCGGCTGCCATCTTCATGACCGCCACA 5460
CPC0740             GGATACATTTCAACAAGGGTTGAGATGGGCGAGGCGGCTGCCATCTTCATGACCGCCACG 5460
YAP2007             GGATACATTTCAACAAGGGTTGAGATGGGCGAGGCGGCCGCCATCTTCATGACCGCCACC 5460
FSS13025            GGATACATTTCAACAAGGGTTGAGATGGGCGAGGCGGCTGCCATCTTCATGACTGCCACG 5460
PLCal_ZV            GGATACATTTCAACAAGGGTTGAGATGGGCGAGGCGGCCGCCATCTTCATGACCGCCACG 5349
SV0127/14           GGATACATTTCAACAAGGGTTGAGATGGGCGAGGCAGCTGCCATCTTCATGACCGCCACG 5460
8375                GGATACATTTCAACAAGGGTTGAGATGGGCGAGGCGGCTGCCATCTTCATGACCGCCACG 5460
103344              GGATACATTTCAACAAGGGTTGAGATGGGCGAGGCGGCTGCCATCTTCATGACCGCCACG 5460
BrasilZKV2015       GGATACATTTCAACAAGGGTTGAGATGGGCGAGGCGGCTGCCATCTTCATGACCGCCACG 5460
GD01                GGATACATTTCAACAAGGGTTGAGATGGGCGAGGCGGCTGCCATCTTCATGACCGCCACG 5460
Martinique          GGATACATTTCAACAAGGGTTGAGATGGGCGAGGCGGCTGCCATCTTCATGACCGCCACG 5460
NatalRGN            GGATACATTTCAACAAGGGTTGAGATGGGCGAGGCGGCTGCCATCTTCATGACCGCCACG 5460
HPF2013             GGATACATTTCAACAAGGGTTGAGATGGGCGAGGCGGCTGCCATCTTCATGACCGCCACG 5460
SPH2015             GGATACATTTCAACAAGGGTTGAGATGGGCGAGGCGGCTGCCATCTTCATGACCGCCACG 5460
Haiti2014           GGATACATTTCAACAAGGGTTGAGATGGGCGAGGCGGCTGCCATCTTCATGACCGCCACG 5460
PRVABC59            GGATACATTTCAACAAGGGTTGAGATGGGCGAGGCGGCTGCCATCTTCATGACCGCCACG 5460
BeH819015           GGATACATTTCAACAAGGGTTGAGATGGGCGAGGCGGCTGCCATCTTCATGACCGCCACG 5460
Z1106033            GGATACATTTCAACAAGGGTTGAGATGGGCGAGGCGGCCGCCATCTTCATGACCGCCACG 5460
BEH819966           GGATACATTTCAACAAGGGTTGAGATGGGCGAGGCGGCTGCCATCTTCATGACCGCCACG 5460
SSABr               GGATACATTTCAACAAGGGTTGAGATGGGCGAGGCGGCTGCCATCTTCATGACCGCCACG 5460
Beh815744           GGATACATTTCAACAAGGGTTGAGATGGGCGAGGCGGCTGCCATCTTCATGACCGCCACG 5460
BEH818995           GGATACATTTCAACAAGGGTTGAGATGGGCGAGGCGGCTGCCATCTTCATGACCGCCACG 5460
IbH30656            GGATACATATCAACAAGGGTTGAAATGGGCGAGGCGGCTGCTATCTTCATGACTGCTACA 5442
ArD128000           GGATACATATCAACAAGGGTTGAAATGGGCGAGGCGGCTGCCATTTTTATGACTGCCACA 5460
ArD7117             GGATACATATCAACAAGGGTTGAAATGGGCGAGGCGGCTGCCATTTTTATGACTGCCACA 5460
ARD_41519           GGATATATATCAACAAGGGTTGAAATGGGCGAGGCGGCTGCTATCTTCATGACTGCTACA 5460
ARD157995           GGATACATATCAACAAGGGTTGAAATGGGCGAGGCGGCTGCCATTTTTATGACTGCCACA 5460
MR_766              GGATACATATCAACAAGGGTTGAAATGGGCGAGGCGGCTGCCATTTTTATGACTGCCACA 5448
ARD158084           GGATACATATCAACAAGGGTTGAAATGGGCGAGGCGGCTGCCATTTTTATGACTGCCACA 5460
ArB1362             GGATACATATCAACAAGGGTTGAAATGGGCGAGGCGGCTGCCATTTTTATGACTGCCACA 5460
ARB13565            GGATACATATCAACAAGGGTTGAAATGGGCGAGGCGGCTGCCATCTTCATGACAGCCACG 5460
ARB7701             GGATACATATCAACAAGGGTTGAAATGGGCGAGGCGGCTGCCATCTTCATGACAGCCACG 5460
ARB15076            GGATACATATCAACAAGGGTTGAAATGGGCGAGGCGGCTGCCATCTTTATGACAGCCACG 5442
                    ***** ** ************** *********** ** ** ** ** ***** ** **

P6-740              CCACCAGGAACCCGCGACGCATTTCCGGACTCTAACTCACCAATCATGGACACAGAAGTG 5520
CPC0740             CCACCAGGAACCCGTGACGCATTCCCGGACTCCAACTCACCAATTATGGACACCGAGGTG 5520
YAP2007             CCACCAGGAACCCGTGACGCATTCCCGGACTCCAACTCACCAATTATGGACACCGAAGTG 5520
FSS13025            CCACCAGGAACCCGTGACGCATTCCCGGACTCCAACTCACCAATTATGGACACCGAAGTG 5520
PLCal_ZV            CCACCAGGAACCCGTGACGCATTCCCGGACTCCAACTCACCAATTATGGACACCGAAGTG 5409
SV0127/14           CCACCAGGAACCCGTGACGCATTCCCGGACTCCAACTCACCAATTATGGACACCGAAGTG 5520
```

```
8375              CCACCAGGAACCCGTGACGCATTTCCGGACTCCAACTCACCAATTATGGACACCGAAGTG 5520
103344            CCACCAGGAACCCGTGACGCATTTCCGGACTCCAACTCACCAATTATGGACACCGAAGTG 5520
BrasilZKV2015     CCACCAGGAACCCGTGACGCATTTCCGGACTCCAACTCACCAATTATGGACACCGAAGTG 5520
GD01              CCACCAGGAACCCGTGACGCATTTCCGGACTCCAACTCACCAATTATGGACACCGAAGTG 5520
Martinique        CCACCAGGAACCCGTGACGCATTTCCGGACTCCAACTCACCAATTATGGACACCGAAGTG 5520
NatalRGN          CCACCAGGAACCCGTGACGCATTTCCGGACTCCAACTCACCAATTATGGACACCGAAGTG 5520
HPF2013           CCACCAGGAACCCGTGACGCATTTCCGGACTCCAACTCACCAATTATGGACACCGAAGTG 5520
SPH2015           CCACCAGGAACCCGTGACGCATTTCCGGACTCCAACTCACCAATTATGGACACCGAAGTG 5520
Haiti2014         CCACCAGGAACCCGTGACGCATTTCCGGACTCCAACTCACCAATTATGGACACCGAAGTG 5520
PRVABC59          CCACCAGGAACCCGTGACGCATTTCCGGACTCCAACTCACCAATTATGGACACCGAAGTG 5520
BeH819015         CCACCAGGAACCCGTGACGCATTTCCGGACTCCAACTCACCAATTATGGACACCGAAGTG 5520
Z1106033          CCACCAGGAACCCGTGACGCATTTCCGGACTCCAACTCACCAATTATGGACACCGAAGTG 5520
BEH819966         CCACCAGGAACCCGTGACGCATTTCCGGACTCCAACTCACCAATTATGGACACCGAAGTG 5520
SSABr             CCACCAGGAACCCGTGACGCATTTCCGGACTCCAACTCACCAATTATGGACACCGAAGTG 5520
Beh815744         CCACCAGGAACCCGTGACGCATTTCCGGACTCCAACTCACCAATTATGGACACCGAAGTG 5520
BEH818995         CCACCAGGAACCCGTGACGCATTTCCGGACTCCAACTCACCAATTATGGACACCGAAGTG 5520
IbH30656          CCACCAGGAACCCGCGATGCGTTTCCAGATTCCAACTCACCAATCATGGACACAGAAGTG 5502
ArD128000         CCACCAGGAACCCGTGATGCGTTTCCTGACTCTAACTCACCAATCATGGACACAGAAGTG 5520
ArD7117           CCACCAGGAACCCGTGATGCGTTTCCTGACTCTAACTCACCAATCATGGACACAGAAGTG 5520
ARD_41519         CCACCAGGAACCCGCGATGCGTTTCCGGATTCCAACTCACCAATCATGGACACAGAAGTG 5520
ARD157995         CCACCAGGAACCCGTGATGCGTTTCCTGACTCTAACTCACCAATCATGGACACAGAAGTG 5520
MR_766            CCACCAGGAACCCGTGATGCGTTTCCTGACTCTAACTCACCAATCATGGACACAGAAGTG 5508
ARD158084         CCACCAGGAACCCGTGATGCGTTTCCTGACTCTAACTCACCAATCATGGACACAGAAGTG 5520
ArB1362           CCACCAGGAACCCGTGATGCGTTTCCTGACTCTAACTCACCAATCATGGACACAGAAGTG 5520
ARB13565          CCACCAGGAACCCGAGATGCATTTCCAGACTCCAACTCACCAATCATGGACACAGAAGTG 5520
ARB7701           CCACCAGGAACCCGAGATGCATTTCCAGACTCCAACTCACCAATCATGGACACAGAAGTG 5520
ARB15076          CCACCAGGAACCCGAGATGCATTTCCAGACTCCAACTCACCAATCATGGACACAGAAGTG 5502
                  ************** ** ** ** ** ** ** *********** ******** ** ***

P6-740            GAAGTCCCAGAGAGAGCCTGGAGCTCAGGCTTTGATTGGGTGACGGATCATTCTGGAAAA 5580
CPC0740           GAAGTCCCAGAGAGAGCCTGGAGCACAGGCTTTGATTGGGTGACGGATCATTCTGGGAAA 5580
YAP2007           GAAGTCCCAGAGAGAGCCTGGAGCTCAGGCTTTGATTGGGTGACGGATCATTCTGGAAAA 5580
FSS13025          GAAGTCCCAGAGAGAGCCTGGAGCTCAGGCTTTGATTGGGTGACGGATCATTCTGGAAAA 5580
PLCal_ZV          GAAGTCCCAGAGAGAGCCTGGAGCTCAGGCTTTGATTGGGTGACGGATCATTCTGGAAAA 5469
SV0127/14         GAAGTCCCAGAGAGAGCCTGGAGCTCAGGCTTTGATTGGGTGACGGATCATTCTGGAAAA 5580
8375              GAAGTCCCAGAGAGAGCCTGGAGCTCAGGCTTTGATTGGGTGACGGATCATTCTGGAAAA 5580
103344            GAAGTCCCAGAGAGAGCCTGGAGCTCAGGCTTTGATTGGGTGACGGATCATTCTGGAAAA 5580
BrasilZKV2015     GAAGTCCCAGAGAGAGCCTGGAGCTCAGGCTTTGATTGGGTGACGGATCATTCTGGAAAA 5580
GD01              GAAGTCCCAGAGAGAGCCTGGAGCTCAGGCTTTGATTGGGTGACGGATCATTCTGGAAAA 5580
Martinique        GAAGTCCCAGAGAGAGCCTGGAGCTCAGGCTTTGATTGGGTGACGGATCATTCTGGAAAA 5580
NatalRGN          GAAGTCCCAGAGAGAGCCTGGAGCTCAGGCTTTGATTGGGTGACGGATCATTCTGGAAAA 5580
HPF2013           GAAGTCCCAGAGAGAGCCTGGAGCTCAGGCTTTGATTGGGTGACGGATCATTCTGGAAAA 5580
SPH2015           GAAGTCCCAGAGAGAGCCTGGAGCTCAGGCTTTGATTGGGTGACGGATTATTCTGGAAAA 5580
Haiti2014         GAAGTCCCAGAGAGAGCCTGGAGCTCAGGCTTTGATTGGGTGACGGATTATTCTGGAAAA 5580
PRVABC59          GAAGTCCCAGAGAGAGCCTGGAGCTCAGGCTTTGATTGGGTGACGGATCATTCTGGAAAA 5580
BeH819015         GAAGTCCCAGAGAGAGCCTGGAGCTCAGGCTTTGATTGGGTGACGGATCATTCTGGAAAA 5580
Z1106033          GAAGTCCCAGAGAGAGCCTGGAGCTCAGGCTTTGATTGGGTGACGGATCATTCTGGAAAA 5580
BEH819966         GAAGTCCCAGAGAGAGCCTGGAGCTCAGGCTTTGATTGGGTGACGGATCATTCTGGAAAA 5580
SSABr             GAAGTCCCAGAGAGAGCCTGGAGCTCAGGCTTTGATTGGGTGACGGATCATTCTGGAAAA 5580
Beh815744         GAAGTCCCAGAGAGAGCCTGGAGCTCAGGCTTTGATTGGGTGACGGATCATTCTGGAAAA 5580
BEH818995         GAAGTCCCAGAGAGAGCCTGGAGCTCAGGCTTTGATTGGGTGACGGATCATTCTGGAAAA 5580
IbH30656          GAAGTCCCAGAGAGAGCCTGGAGCTCAGGCTTTGACTGGGTGACGGACCATTCTGGAAAA 5562
ArD128000         GAAGTCCCAGAGAGAGCCTGGAGCTCAGGCTTTGATTGGGTGACAGACCATTCTGGGAAA 5580
ArD7117           GAAGTCCCAGAGAGAGCCTGGAGCTCAGGCTTTGACTGGGTGACGGACCATTCTGGGAAA 5580
ARD_41519         GAAGTTCCAGAGAGAGCCTGGAGCTCAGGCTTTGACTGGGTGACGGATCATTCTGGGAAA 5580
ARD157995         GAAGTCCCAGAGAGAGCCTGGAGCTCAGGCTTTGATTGGGTGACAGACCATTCTGGGAAA 5580
MR_766            GAAGTCCCAGAGAGAGCCTGGAGCTCAGGCTTTGATTGGGTGACAGACCATTCTGGGAAA 5568
ARD158084         GAAGTCCCAGAGAGAGCCTGGAGCTCAGGCTTTGATTGGGTGACAGACCATTCTGGGAAA 5580
ArB1362           GAAGTCCCAGAGAGAGCCTGGAGCTCAGGCTTTGATTGGGTGACAGACCATTCTGGGAAA 5580
ARB13565          GAAGTCCCAGAGAGAGCCTGGAGCTCAGGCTTTGATTGGGTGACAGACCATTCTGGGAAA 5580
ARB7701           GAAGTCCCAGAGAGAGCCTGGAGCTCAGGCTTTGATTGGGTGACAGACCATTCTGGGAAA 5580
ARB15076          GAAGTCCCAGAGAGAGCCTGGAGCTCAGGCTTTGATTGGGTGACAGACCATTCTGGAAAA 5562
                  ***** ****************** ********** ******** **  ******* ***

P6-740            ACAGTTTGGTTTGTTCCAAGCGTGAGGAACGGCAACGAGATCGCGGCTTGTCTGACAAAA 5640
CPC0740           ACAGTCTGGTTTGTTCCAAGCGTGAGGAACGGCAATGAGATCGCAGCTTGTCTGACAAAG 5640
YAP2007           ACAGTTTGGTTTGTTCCAAGCGTGAGGAACGGCAATGAGATCGCAGCTTGTCTGACAAAG 5640
FSS13025          ACAGTTTGGTTTGTTCCAAGCGTGAGGAATGGCAATGAGATCGCAGCTTGTCTGACAAAG 5640
```

```
PLCal_ZV         ACAGTTTGGTTTGTTCCAAGCGTGAGGAACGGCAATGAGATCGCAGCTTGTCTGACGAAG 5529
SV0127/14        ACAGTTTGGTTTGTCCCAAGCGTGAGGAACGGCAATGAGATCGCAGCTTGTCTGACAAAG 5640
8375             ACAGTTTGGTTTGTTCCAAGCGTGAGGAACGGCAATGAGATCGCAGCTTGTCTGACAAAG 5640
103344           ACAGTTTGGTTTGTTCCAAGCGTGAGGAACGGCAATGAGATCGCAGCTTGTCTGACAAAG 5640
BrasilZKV2015    ACAGTTTGGTTTGTTCCAAGCGTGAGGAACGGCAATGAGATCGCAGCTTGTCTGACAAAG 5640
GD01             ACAGTTTGGTTTGTTCCAAGCGTGAGGAACGGCAATGAGATCGCAGCTTGTCTGACAAAG 5640
Martinique       ACAGTTTGGTTTGTTCCAAGCGTGAGGAACGGCAATGAGATCGCAGCTTGTCTGACAAAG 5640
NatalRGN         ACAGTTTGGTTTGTTCCAAGCGTGAGGAACGGCAATGAGATCGCAGCTTGTCTGACAAAG 5640
HPF2013          ACAGTTTGGTTTGTTCCAAGCGTGAGGAACGGCAATGAGATCGCAGCTTGTCTGACAAAG 5640
SPH2015          ACAGTTTGGTTTGTTCCAAGCGTGAGGAACGGCAATGAGATCGCAGCTTGTCTGACAAAG 5640
Haiti2014        ACAGTTTGGTTTGTTCCAAGCGTGAGGAACGGCAATGAGATCGCAGCTTGTCTGACAAAG 5640
PRVABC59         ACAGTTTGGTTTGTTCCAAGCGTGAGGAACGGCAATGAGATCGCAGCTTGTCTGACAAAG 5640
BeH819015        ACAGTTTGGTTTGTTCCAAGCGTGAGGAACGGCAATGAGATCGCAGCTTGTCTGACAAAG 5640
Z1106033         ACAGTTTGGTTTGTTCCAAGCGTGAGGAACGGCAATGAGATCGCAGCTTGTCTGACAAAG 5640
BEH819966        ACAGTTTGGTTTGTTCCAAGCGTGAGGAACGGCAATGAGATCGCAGCTTGTCTGACAAAG 5640
SSABr            ACAGTTTGGTTTGTTCCAAGCGTGAGGAACGGCAATGAGATCGCAGCTTGTCTGACAAAG 5640
Beh815744        ACAGTTTGGTTTGTTCCAAGCGTGAGGAACGGCAATGAGATCGCAGCTTGTCTGACAAAG 5640
BEH818995        ACAGTTTGGTTTGTTCCAAGCGTGAGGAACGGCAATGAGATCGCAGCTTGTCTGACAAAG 5640
IbH30656         ACAATTTGGTTTGTTCCAAGTGTGAGAAACGGAAATGAAATCGCAGCCTGTCTGACAAAG 5622
ArD128000        ACAATTTGGTTTGTTCCAAGTGTGAGAAACGGAAATGAAATCGCAGCCTGTCTGACAAAG 5640
ArD7117          ACAATTTGGTTTGTTCCAAGTGTGAGATCTGGAGAAGAAAGCGCAGCCTGTCTGACAAAG 5640
ARD_41519        ACAATTTGGTTTGTTCCAAGTGTGAGAAACGGAAATGAAATCGCAGCCTGTCTGACAAAG 5640
ARD157995        ACAGTTTGGTTCGTTCCAAGCGTGAGATCTGGAGAAGAAAGCGCAGCCTGTCTGACAAAG 5640
MR_766           ACAGTTTGGTTCGTTCCAAGCGTGAGAAACGGAAATGAAATCGCAGCCTGTCTGACAAAG 5628
ARD158084        ACAGTTTGGTTCGTTCCAAGCGTGAGAAACGGAAATGAAATCGCAGCCTGTCTGACAAAG 5640
ArB1362          ACAGTTTGGTTCGTTCCAAGTGTGAGAAATGGAAATGAAATCACAGCCTGCCTGACAAAG 5640
ARB13565         ACAGTTTGGTTCGTTCCAAGTGTGAGAAATGGAAATGAAATCGCAGCCTGTCTGACAAAG 5640
ARB7701          ACAGTTTGGTTCGTTCCAAGTGTGAGAAATGGAAATGAAATCGCAGCCTGTCTGACAAAG 5640
ARB15076         ACAGTTTGGTTTGTTCCAAGTGTGAGAAATGGAAATGAAATCGCAGCCTGTCTGACAAAG 5622
                 *** * ***** ** ***** *****     **  * ** * * * ** ** ***** **

P6-740           GCTGGAAAACGGGTCATACAGCTCAGCAGAAAGACTTTTGAGACAGAGTTCCAGAAAACA 5700
CPC0740          GCTGGAAAACGGGTCATACAGCTCAGCAGAAAGACTTTTGAGACAGAGTTCCAGAAAACG 5700
YAP2007          GCTGGAAAACGGGTCATACAGCTCAGCAGAAAGACTTTTGAGACAGAGTTCCTGAAAACA 5700
FSS13025         GCTGGAAAACGGGTCATACAGCTCAGCAGAAAGACTTTTGAGACAGAGTTCCAGAAAACA 5700
PLCal_ZV         GCTGGAAAACGGGTCATACAGCTCAGCAGAAAGACTTTTGAGACAGAGTTCCAGAAAACA 5589
SV0127/14        GCTGGAAAACGGGTCATACAGCTCAGCAGAAAGACTTTTGAGACAGAGTTCCAGAAAACA 5700
8375             GCTGGAAAACGGGTCATACAGCTCAGCAGAAAGACTTTTGAGACAGAGTTCCAGAAAACA 5700
103344           GCTGGAAAACGGGTCATACAGCTCAGCAGAAAGACTTTTGAGACAGAGTTCCAGAAAACA 5700
BrasilZKV2015    GCTGGAAAACGGGTCATACAGCTCAGCAGAAAGACTTTTGAGACAGAGTTCCAGAAAACA 5700
GD01             GCTGGAAAACGGGTCATACAGCTCAGCAGAAAGACTTTTGAGACAGAGTTCCAGAAAACA 5700
Martinique       GCTGGAAAACGGGTCATACAGCTCAGCAGAAAGACTTTTGAGACAGAGTTCCAGAAAACA 5700
NatalRGN         GCTGGAAAACGGGTCATACAGCTCAGCAGAAAGACTTTTGAGACAGAGTTCCAGAAAACA 5700
HPF2013          GCTGGAAAACGGGTCATACAGCTCAGCAGAAAGACTTTTGAGACAGAGTTCCAGAAAACA 5700
SPH2015          GCTGGAAAACGGGTCATACAGCTCAGCAGAAAGACTTTTGAGACAGAGTTCCAGAAAACA 5700
Haiti2014        GCTGGAAAACGGGTCATACAGCTCAGCAGAAAGACTTTTGAGACAGAGTTCCAGAAAACA 5700
PRVABC59         GCTGGAAAACGGGTCATACAGCTCAGCAGAAAGACTTTTGAGACAGAGTTCCAGAAAACA 5700
BeH819015        GCTGGAAAACGGGTCATACAGCTCAGCAGAAAGACTTTTGAGACAGAGTTCCAGAAAACA 5700
Z1106033         GCTGGAAAACGGGTCATACAGCTCAGCAGAAAGACTTTTGAGACAGAGTTCCAGAAAACA 5700
BEH819966        GCTGGAAAACGGGTCATACAGCTCAGCAGAAAGACTTTTGAGACAGAGTTCCAGAAAACA 5700
SSABr            GCTGGAAAACGGGTCATACAGCTCAGCAGAAAGACTTTTGAGACAGAGTTCCAGAAAACA 5700
Beh815744        GCTGGAAAACGGGTCATACAGCTCAGCAGAAAGACTTTTGAGACAGAGTTCCAGAAAACA 5700
BEH818995        GCTGGAAAACGGGTCATACAGCTCAGCAGAAAGACTTTTGAGACAGAGTTCCAGAAAACA 5700
IbH30656         GCTGGAAAGCGGGTTATACAGCTCAGCAGGAAGACTTTTGAGACAGAGTTTCAGAAGACA 5682
ArD128000        GCTGGAAAGCGGGTTATACAGCTCAGCAGGAAGACTTTTGAGACAGAGTTTCAGAAGACA 5700
ArD7117          GCTGGAAAGCGGGTTATACAGCTCAGCAGGAAGACTTTTGAGACAGAGTTTCAGAAGACA 5700
ARD_41519        GCTGGAAAGCGGGTCATACAGCTCAGCAGGAAGACTTTTGAGACAGAGTTTCAGAAGACA 5700
ARD157995        GCTGGAAAGCGGGTCATACAGCTCAGCAGGAAGACTTTTGAGACAGAATTTCAGAAAACA 5700
MR_766           GCTGGAAAGCGGGTCATACAGCTCAGCAGGAAGACTTTTGAGACAGAATTTCAGAAAACA 5688
ARD158084        GCTGGAAAGCGGGTCATACAGCTCAGCAGGAAGACTTTTGAGACAGAATTTCAGAAAACA 5700
ArB1362          GCTGGAAAGCGGGTCATACAGCTCAGCAGGAAGACTTTTGAGACAGAGTTCCAGAAAACA 5700
ARB13565         GCTGGAAAGCGGGTCATACAACTCAGCAGGAAGACTTTTGAGACAGAGTTTCAGAAAACA 5700
ARB7701          GCTGGAAAGCGGGTCATACAACTCAGCAGGAAGACTTTTGAGACAGAGTTTCAGAAAACA 5700
ARB15076         GCTGGAAAGCGGGTCATACAACTCAGCAGGAAGACTTTTGAGACAGAGTTTCAGAAAACA 5682
                 ******** ***** ***** ******** ***************** ** * *** **

P6-740           AAAAATCAAGAGTGGGACTTCGTCGTAACAACTGACATCTCAGAGATGGGCGCCAACTTC 5760
CPC0740          AAAAATCAAGAGTGGGACTTCGTCGTGACAACCGACATTTCAGAGATGGGCGCCAACTTT 5760
```

```
YAP2007          AAAAATCAAGAGTGGGACTTCGTCGTGACAACTGACATTTCAGAGATGGGCGCCAACTTT 5760
FSS13025         AAACATCAAGAGTGGGACTTCGTCGTGACAACTGACATTTCAGAGATGGGCGCCAACTTT 5760
PLCal_ZV         AAACATCAAGAGTGGGACTTCGTCGTGACAACTGACATTTCAGAGATGGGCGCCAACTTT 5649
SV0127/14        AAACATCAAGAGTGGGACTTCGTCGTGACAACTGACATTTCAGAGATGGGCGCCAACTTT 5760
8375             AAACATCAAGAGTGGGACTTTGTCGTGACAACTGACATTTCAGAGATGGGCGCCAACTTT 5760
103344           AAACATCAAGAGTGGGACTTTGTCGTGACAACTGACATTTCAGAGATGGGCGCCAACTTT 5760
BrasilZKV2015    AAACATCAAGAGTGGGACTTTGTCGTGACAACTGACATTTCAGAGATGGGCGCCAACTTT 5760
GD01             AAACATCAAGAGTGGGACTTTGTCGTGACAACTGACATTTCAGAGATGGGCGCCAACTTT 5760
Martinique       AAACATCAAGAGTGGGACTTTGTCGTGACAACTGACATTTCAGAGATGGGCGCCAACTTT 5760
NatalRGN         AAACATCAAGAGTGGGACTTTGTCGTGACAACTGACATTTCAGAGATGGGCGCCAACTTT 5760
HPF2013          AAACATCAAGAGTGGGACTTTGTCGTGACAACTGACATTTCAGAGATGGGCGCCAACTTT 5760
SPH2015          AAACATCAAGAGTGGGACTTTGTCGTGACAACTGACATTTCAGAGATGGGCGCCAACTTT 5760
Haiti2014        AAACATCAAGAGTGGGACTTTGTCGTGACAACTGACATTTCAGAGATGGGCGCCAACTTT 5760
PRVABC59         AAACATCAAGAGTGGGACTTTGTCGTGACAACTGACATTTCAGAGATGGGCGCCAACTTT 5760
BeH819015        AAACATCAAGAGTGGGACTTTGTCGTGACAACTGACATTTCAGAGATGGGCGCCAACTTT 5760
Z1106033         AAACATCAAGAGTGGGACTTTGTCGTGACAACTGACATTTCAGAGATGGGCGCCAACTTT 5760
BEH819966        AAACATCAAGAGTGGGACTTTGTCGTGACAACTGACATTTCAGAGATGGGCGCCAACTTT 5760
SSABr            AAACATCAAGAGTGGGACTTTGTCGTGACAACTGACATTTCAGAGATGGGCGCCAACTTT 5760
Beh815744        AAACATCAAGAGTGGGACTTTGTCGTGACAACTGACATTTCAGAGATGGGCGCCAACTTT 5760
BEH818995        AAACATCAAGAGTGGGACTTTGTCGTGACAACTGACATTTCAGAGATGGGCGCCAACTTT 5760
IbH30656         AAAAATCAAGAGTGGGACTTTGTCATAACAACTGACATTTCAGAGATGGGTGCCAACTTC 5742
ArD128000        AAAAATCAAGAGTGGGACTTTGTCATAACAACTGACATTTCAGAGATGGGTGCCAATTTC 5760
ArD7117          AAAAATCAAGAGTGGGACTTTGTCATAACAACTGACATTTCAGAGATGGGTGCCAATTTC 5760
ARD_41519        AAAAATCAAGAGTGGGACTTTGTCATAACAACTGACATTTCAGAGATGGGTGCCAATTTC 5760
ARD157995        AAAAATCAAGAGTGGGACTTTGTCATAACAACTGACATCTCAGAGATGGGCGCCAACTTC 5760
MR_766           AAAAATCAAGAGTGGGACTTTGTCATAACAACTGACATCTCAGAGATGGGCGCCAACTTC 5748
ARD158084        AAAAATCAAGAGTGGGACTTTGTCATAACAACTGACATCTCAGAGATGGGCGCCAACTTC 5760
ArB1362          AAAAACCAAGAGTGGGACTTTGTCATAACAACTGACATCTCAGAGATGGGCGCCAATTTC 5760
ARB13565         AAAAATCAAGAGTGGGACTTTGTCATAACAACTGACATCTCAGAGATGGGCGCGAATTTC 5760
ARB7701          AAAAATCAAGAGTGGGACTTTGTCATAACAACTGACATCTCAGAGATGGGCGCGAATTTC 5760
ARB15076         AAAAATCAAGAGTGGGACTTTGTCATAACAACTGACATCTCAGAGATGGGTGCCAATTTC 5742
                 *** * ************** *** * ***** ***** *********** ** ** **

P6-740           AAAGCTGACCGGGTCATAGATTCCAGGAGATGCCTGAAGCCGGTCATACTTGATGGCGAG 5820
CPC0740          AAAGCTGACCGTGTCATAGATTCCAGGAGATGCTTAAAGCCGGTCATACTTGATGGCGAG 5820
YAP2007          AAAGCTGACCGTGTCATAGATTCCAGGAGATGCCTAAAGCCGGTCATACTTGATGGCGAG 5820
FSS13025         AAAGCTGACCGTGTCATAGATTCCAGGAGATGCCTAAAGCCGGTCATACTTGATGGCGAG 5820
PLCal_ZV         AAAGCTGACCGTGTCATAGATTCCAGGAGATGCCTAAAGCCGGTCATACTTGATGGCGAG 5709
SV0127/14        AAAGCTGACCGTGTCATAGATTCCAGGAGATGCCTAAAGCCGGTCATACTTGATGGCGAG 5820
8375             AAAGCTGACCGTGTCATAGATTCCAGGAGATGCCTAAAGCCGGTCATACTTGATGGCGAG 5820
103344           AAAGCTGACCGTGTCATAGATTCCAGGAGATGCCTAAAGCCGGTCATACTTGATGGCGAG 5820
BrasilZKV2015    AAAGCTGACCGTGTCATAGATTCCAGGAGATGCCTAAAGCCGGTCATACTTGATGGCGAG 5820
GD01             AAAGCTGACCGTGTCATAGATTCCAGGAGATGCCTAAAGCCGGTCATACTTGATGGCGAG 5820
Martinique       AAAGCTGACCGTGTCATAGATTCCAGGAGATGCCTAAAGCCGGTCATACTTGATGGCGAG 5820
NatalRGN         AAAGCTGACCGTGTCATAGATTCCAGGAGATGCCTAAAGCCGGTCATACTTGATGGCGAG 5820
HPF2013          AAAGCTGACCGTGTCATAGATTCCAGGAGATGCCTAAAGCCGGTCATACTTGATGGCGAG 5820
SPH2015          AAAGCTGACCGTGTCATAGATTCCAGGAGATGCCTAAAGCCGGTCATACTTGATGGCGAG 5820
Haiti2014        AAAGCTGACCGTGTCATAGATTCCAGGAGATGCCTAAAGCCGGTCATACTTGATGGCGAG 5820
PRVABC59         AAAGCTGACCGTGTCATAGATTCCAGGAGATGCCTAAAGCCGGTCATACTTGATGGCGAG 5820
BeH819015        AAAGCTGACCGTGTCATAGATTCCAGGAGATGCCTAAAGCCGGTCATACTTGATGGCGAG 5820
Z1106033         AAAGCTGACCGTGTCATAGATTCCAGGAGATGCCTAAAGCCGGTCATACTTGATGGCGAG 5820
BEH819966        AAAGCTGACCGTGTCATAGATTCCAGGAGATGCCTAAAGCCGGTCATACTTGATGGCGAG 5820
SSABr            AAAGCTGACCGTGTCATAGATTCCAGGAGATGCCTAAAGCCGGTCATACTTGATGGCGAG 5820
Beh815744        AAAGCTGACCGTGTCATAGATTCCAGGAGATGCCTAAAGCCGGTCATACTTGATGGCGAG 5820
BEH818995        AAAGCTGACCGTGTCATAGATTCCAGGAGATGCCTAAAGCCGGTCATACTTGATGGCGAG 5820
IbH30656         AAGGCTGACCGGGTCATAGATTCCAGGAGATGCCTAAAGCCAGTCATACTTGATGGTGAG 5802
ArD128000        AAGGCTGACCGGGTCATCGATTCCAGGAGATGCCTAAAGCCAGTCATACTTGATGGTGAG 5820
ArD7117          AAGGCTGACCGGGTCATAGATTCCAGGAGATGCCTAAAGCCAGTCATACTCGATGGTGAG 5820
ARD_41519        AAGGCTGACCGGGTCATAGATTCCAGGAGATGCCTAAAGCCAGTCATACTTGATGGGGAG 5820
ARD157995        AAGGCTGACCGGGTCATAGACTCTAGGAGATGCCTAAAGCCAGTCATACTTGATGGTGAG 5820
MR_766           AAGGCTGACCGGGTCATAGACTCTAGGAGATGCCTAAAACCAGTCATACTTGATGGTGAG 5808
ARD158084        AAGGCTGACCGGGTCATAGACTCTAGGAGATGCCTAAAACCAGTCATACTTGATGGTGAG 5820
ArB1362          AAGGCTGACCGGGTCATAGACTCTAGGAGATGCCTAAAGCCAGTCATACTTGATGGTGAG 5820
ARB13565         AAAGCTGACCGGGTCATAGACTCTAGGAGATGCCTAAAGCCAGTCATACTTGATGGTGAG 5820
ARB7701          AAAGCTGACCGGGTCATAGACTCTAGGAGATGCCTAAAGCCAGTCATACTTGATGGTGAG 5820
ARB15076         AAGGCTGACCGGGTCATAGACTCTAGGAGATGCCTAAAGCCAGTTATACTTGATGGTGAG 5802
                 ** ******** ***** ** ** ********* * ** ** ** ***** ***** ***
```

```
P6-740            AGAGTCATTCTGGCTGGACCCATGCCTGTCACACATGCCAGCGCTGCCCAGAGGAGGGGG 5880
CPC0740           AGAGTCATTTTGGCTGGACCCATGCCTGTCACACATGCCAGCGCTGCTCAGAGGAGGGGG 5880
YAP2007           AGAGTCATTCTGGCTGGACCCATGCCTGTCACACATGCCAGCGCTGCCCAGAGGAGGGGG 5880
FSS13025          AGAGTCATTCTGGCTGGACCCATGCCTGTCACACATGCCAGCGCTGCCCAGAGGAGGGGG 5880
PLCal_ZV          AGAGTCATTCTGGCTGGACCCATGCCTGTCACACATGCCAGCGCTGCCCAGAGGAGGGGG 5769
SV0127/14         AGAGTCATTCTGGCTGGACCCATGCCTGTCACACATGCCAGCGCTGCCCAGAGGAGGGGG 5880
8375              AGAGTCATTCTGGCTGGACCCATGCCTGTCACACATGCCAGCGCTGCCCAGAGGAGGGGG 5880
103344            AGAGTCATTCTGGCTGGACCCATGCCTGTCACACATGCCAGCGCTGCCCAGAGGAGGGGG 5880
BrasilZKV2015     AGAGTCATTCTGGCTGGACCCATGCCTGTCACACATGCCAGCGCTGCCCAGAGGAGGGGG 5880
GD01              AGAGTCATTCTGGCTGGACCCATGCCTGTCACACATGCCAGCGCTGCCCAGAGGAGGGGG 5880
Martinique        AGAGTCATTCTGGCTGGACCCATGCCTGTCACACATGCCAGCGCTGCCCAGAGGAGGGGG 5880
NatalRGN          AGAGTCATTTTGGCTGGACCCATGCCTGTCACACATGCCAGCGCTGCCCAGAGGAGGGGG 5880
HPF2013           AGAGTCATTCTGGCTGGACCCATGCCTGTCACACATGCCAGCGCTGCCCAGAGGAGGGGG 5880
SPH2015           AGAGTCATTCTGGCTGGACCCATGCCTGTCACACATGCCAGCGCTGCCCAGAGGAGGGGG 5880
Haiti2014         AGAGTCATTCTGGCTGGACCCATGCCTGTCACACATGCCAGCGCTGCCCAGAGGAGGGGG 5880
PRVABC59          AGAGTCATTCTGGCTGGACCCATGCCTGTCACACATGCCAGCGCTGCCCAGAGGAGGGGG 5880
BeH819015         AGAGTCATTCTGGCTGGACCCATGCCTGTCACACATGCCAGCGCTGCCCAGAGGAGGGGG 5880
Z1106033          AGAGTCATTCTGGCTGGACCCATGCCTGTCACACATGCCAGCGCTGCCCAGAGGAGGGGG 5880
BEH819966         AGAGTCATTCTGGCTGGACCCATGCCTGTCACACATGCCAGCGCTGCCCAGAGGAGGGGG 5880
SSABr             AGAGTCATTCTGGCTGGACCCATGCCTGTCACACATGCCAGCGCTGCCCAGAGGAGGGGG 5880
Beh815744         AGAGTCATTCTGGCTGGACCCATGCCTGTCACACATGCCAGCGCTGCCCAGAGGAGGGGG 5880
BEH818995         AGAGTCATTCTGGCTGGACCCATGCCTGTCACACATGCCAGCGCTGCCCAGAGGAGGGGG 5880
IbH30656          AGAGTCATCCTGGCTGGGCCTATGCCCGTCACGCACGCCAGTGCTGCTCAGAGGAGAGGA 5862
ArD128000         AGAGTCATCCTGGCTGGGCCCATGCCTGTCACGCATGCCAGTGCTGCTCAGAGGAGAGGA 5880
ArD7117           AGAGTCATCCTGGCTGGGCCCATGCCTGTCACGCATGCCAGTGCTGCTCAGAGGAGAGGA 5880
ARD_41519         AGAGTCATCCTGGCTGGGCCCATGCCTGTCACGCATGCCAGTGCTGCTCAGAGGAGAGGA 5880
ARD157995         AGAGTCATCTTGGCTGGGCCCATGCCTGTCACGCATGCTAGTGCTGCTCAGAGGAGAGGA 5880
MR_766            AGAGTCATCTTGGCTGGGCCCATGCCTGTCACGCATGCTAGTGCTGCTCAGAGGAGAGGA 5868
ARD158084         AGAGTCATCTTGGCTGGGCCCATGCCTGTCACGCATGCTAGTGCTGCTCAGAGGAGAGGA 5880
ArB1362           AGAGTCATCTTGGCTGGGCCCATGCCTGTCACGCATGCTAGTGCTGCTCAGAGGAGAGGA 5880
ARB13565          AGAGTCATCTTGGCTGGGCCCATGCCTGTCACGCATGCTAGTGCTGCTCAAAGGAGAGGG 5880
ARB7701           AGAGTCATCTTGGCTGGGCCCATGCCTGTCACGCATGCTAGTGCTGCTCAAAGGAGAGGG 5880
ARB15076          AGAGTCATCTTGGCTGGGCCCATGCCTGTCACGCATGCTAGTGCTGCTCAAAGGAGAGGG 5862
                  ********  ******* ** ***** ***** ** ** ** ***** ** ***** **

P6-740            CGCATAGGCAGGAATCCCAACAAACCTGGAGATGAGTATATGTATGGAGGTGGGTGCGCA 5940
CPC0740           CGCATAGGCAGGAATCCCAACAAACCTGGAGATGAGTATCTGTATGGAGGTGGGTGCGCA 5940
YAP2007           CGCATAGGCAGGAATCCCAACAAACCTGGAGATGAGTATCTGTATGGAGGTGGGTGCGCA 5940
FSS13025          CGCATAGGCAGGAACCCCAACAAACCTGGAGATGAGTATCTGTATGGAGGTGGGTGCGCA 5940
PLCal_ZV          CGCATAGGCAGGAATCCCAATAAACCTGGAGATGAGTATCTGTATGGAGGTGGGTGCGCA 5829
SV0127/14         CGCATAGGCAGGAATCCCAACAAACCTGGAGATGAGTATCTGTATGGAGGTGGGTGCGCA 5940
8375              CGCATAGGCAGGAATCCCAACAAACCTGGAGATGAGTATCTGTATGGAGGTGGGTGCGCA 5940
103344            CGCATAGGCAGGAATCCCAACAAACCTGGAGATGAGTATCTGTATGGAGGTGGGTGCGCA 5940
BrasilZKV2015     CGCATAGGCAGGAATCCCAACAAACCTGGAGATGAGTACCTGTATGGAGGTGGGTGCGCA 5940
GD01              CGCATAGGCAGGAATCCCAACAAACCTGGAGATGAGTATCTGTATGGAGGTGGGTGCGCA 5940
Martinique        CGCATAGGCAGGAATCCCAATAAACCTGGAGATGAGTATCTGTATGGAGGTGGGTGCGCA 5940
NatalRGN          CGCATAGGCAGGAATCCCAACAAACCTGGAGATGAGTATCTGTATGGAGGTGGGTGCGCA 5940
HPF2013           CGCATAGGCAGGAATCCCAACAAACCTGGAGATGAGTATCTGTATGGAGGTGGGTGCGCA 5940
SPH2015           CGCATAGGCAGGAATCCCAACAAACCTGGAGATGAGTATCTGTATGGAGGTGGGTGCGCA 5940
Haiti2014         CGCATAGGCAGGAATCCCAACAAACCTGGAGATGAGTATCTGTATGGAGGTGGGTGCGCA 5940
PRVABC59          CGCATAGGCAGGAATCCCAACAAACCTGGAGATGAGTATCTGTATGGAGGTGGGTGCGCA 5940
BeH819015         CGCATAGGCAGGAATCCCAACAAACCTGGAGATGAGTATCTGTATGGAGGTGGGTGCGCA 5940
Z1106033          CGCATAGGCAGGAATCCCAACAAACCTGGAGATGAGTATCTGTATGGAGGTGGGTGCGCA 5940
BEH819966         CGCATAGGCAGGAATCCCAACAAACCTGGAGATGAGTATCTGTATGGAGGTGGGTGCGCA 5940
SSABr             CGCATAGGCAGGAATCCCAACAAACCTGGAGATGAGTATCTGTATGGAGGTGGGTGCGCA 5940
Beh815744         CGCATAGGCAGGAATCCCAACAAACCTGGAGATGAGTATCTGTATGGAGGTGGGTGCGCA 5940
BEH818995         CGCATAGGCAGGAATCCCAACAAACCTGGAGATGAGTATCTGTATGGAGGTGGGTGCGCA 5940
IbH30656          CGTATAGGCAGGAACCCCAACAAACCTGGAGATGAGTATATGTATGGAGGTGGGTGTGCA 5922
ArD128000         CGTATAGGCAGGAACCCCAACAAACCCGGAGATGAGTACATGTATGGAGGTGGGTGTGCA 5940
ArD7117           CGTATAGGCAGGAACCCCAACAAACCTGGAGATGAGTACATGTATGGAGGTGGGTGTGCA 5940
ARD_41519         CGTGTAGGCAGGAACCCCAACAAACCTGGAGATGAGTACATGTATGGAGGTGGGTGTGCA 5940
ARD157995         CGTATAGGCAGGAACCCTAACAAACCTGGAGATGAGTACATGTATGGAGGTGGGTGTGCA 5940
MR_766            CGTATAGGCAGGAACCCTAACAAACCTGGAGATGAGTACATGTATGGAGGTGGGTGTGCA 5928
ARD158084         CGTATAGGCAGGAACCCTAACAAACCTGGAGATGAGTACATGTATGGAGGTGGGTGTGCA 5940
ArB1362           CGTATAGGCAGGAACCCTAACAAACCTGGAGATGAGTACATGTATGGAGGTGGGTGTGCA 5940
ARB13565          CGTATAGGCAGGAACCCTAACAAACCTGGAGATGAGTACATGTATGGAGGTGGATGTGCA 5940
ARB7701           CGTATAGGCAGGAACCCTAACAAACCTGGAGATGAGTACATGTATGGAGGTGGATGTGCA 5940
ARB15076          CGTATAGGCAGGAACCCTAACAAACCTGGAGATGAGTACATGTATGGAGGTGGATGTGCA 5922
```

```
                   **  ********** ** ** ***** ***********  ************* ** ***

P6-740             GAGACTGATGAAGACCATGCACACTGGCTTGAAGCAAGAATGCTTCTTGATAACATTTAC 6000
CPC0740            GAGACTGATGAAGATCACGCACACTGGCTTGAAGCAAGAATGCTTCTTGACAACATTTAC 6000
YAP2007            GAGACTGATGAAGACCATGCACACTGGCTTGAAGCAAGAATGCTTCTTGACAACATTTAC 6000
FSS13025           GAGACTGATGAAGACCATGCACACTGGCTTGAAGCAAGAATGCTTCTTGACAACATTTAC 6000
PLCal_ZV           GAGACTGATGAAGACCATGCTCACTGGCTTGAAGCAAGAATGCTCCTTGACAACATTTAC 5889
SV0127/14          GAGACTGATGAAGACCATGCACACTGGCTTGAAGCAAGAATGCTCCTTGACAATATTTAC 6000
8375               GAGACTGACGAAGACCATGCACACTGGCTTGAAGCAAGAATGCTCCTTGACAATATTTAC 6000
103344             GAGACTGACGAAGACCATGCACACTGGCTTGAAGCAAGAATGCTCCTTGACAATATTTAC 6000
BrasilZKV2015      GAGACTGACGAAGACCATGCACACTGGCTTGAAGCAAGAATGCTCCTTGACAATATTTAC 6000
GD01               GAGACTGACGAAGACCATGCACACTGGCTTGAAGCAAGAATGCTCCTTGACAATATTTAC 6000
Martinique         GAGACTGACGAAGACCATGCACACTGGCTTGAAGCAAGAATGCTCCTTGACAATATTTAC 6000
NatalRGN           GAGACTGACGAAGACCATGCACACTGGCTTGAAGCAAGAATGCTCCTTGACAATATTTAC 6000
HPF2013            GAGACTGACGAAGACCATGCACACTGGCTTGAAGCAAGAATGCTCCTTGACAATATTTAC 6000
SPH2015            GAGACTGACGAAGACCATGCACACTGGCTTGAAGCAAGAATGCTCCTTGACAATATTTAC 6000
Haiti2014          GAGACTGACGAAGACCATGCACACTGGCTTGAAGCAAGAATGCTCCTTGACAATATTTAC 6000
PRVABC59           GAGACTGACGAAGACCATGCACACTGGCTTGAAGCAAGAATGCTCCTTGACAATATTTAC 6000
BeH819015          GAGACTGACGAAGACCATGCACACTGGCTTGAAGCAAGAATGCTCCTTGACAATATTTAC 6000
Z1106033           GAGACTGACGAAGACCATGCACACTGGCTTGAAGCAAGAATGCTCCTTGACAATATTTAC 6000
BEH819966          GAGACTGACGAAGACCATGCACACTGGCTTGAAGCAAGAATGCTCCTTGACAATATTTAC 6000
SSABr              GAGACTGACGAAGACCATGCACACTGGCTTGAAGCAAGAATGCTCCTTGACAATATTTAC 6000
Beh815744          GAGACTGACGAAGACCATGCACACTGGCTTGAAGCAAGAATGCTCCTTGACAATATTTAC 6000
BEH818995          GAGACTGACGAAGACCATGCACACTGGCTTGAAGCAAGAATGCTCCTTGACAATATTTAC 6000
IbH30656           GAGACTGATGAAGACCATGCACACTGGCTTGAAGCAAGAATGCTTCTCGACAACATTTAC 5982
ArD128000          GAGACTGATGAAGACCATGCACACTGGCTTGAAGCAAGAATGCTTCTTGACAACATTTAC 6000
ArD7117            GAGACTGATGAAGACCATGCACACTGGCTTGAAGCAAGAATGCTTCTTGACAACATTTAC 6000
ARD_41519          GAGACTGATGAAGACCATGCACACTGGCTTGAAGCAAGAATGCTTCTTGACAACATTTAC 6000
ARD157995          GAGACTGATGAAGACCATGCACACTGGCTTGAAGCAAGAATGCTTCTTGACAACATCTAC 6000
MR_766             GAGACTGATGAAGGCCATGCACACTGGCTTGAAGCAAGAATGCTTCTTGACAACATCTAC 5988
ARD158084          GAGACTGATGAAGGCCATGCACACTGGCTTGAAGCAAGAATGCTTCTTGACAACATCTAC 6000
ArB1362            GAGACTGATGAAGACCACGCACACTGGCTTGAAGCAAGAATGCTTCTTGACAACATCTAC 6000
ARB13565           GAGACCGATGAAGACCATGCACACTGGCTTGAAGCAAGAATGCTTCTTGACAACATCTAC 6000
ARB7701            GAGACCGATGAAGACCATGCACACTGGCTTGAAGCAAGAATGCTTCTTGACAACATCTAC 6000
ARB15076           GAGACCGATGAAGACCATGCACACTGGCTTGAAGCAAGAATGCTTCTTGACAACATCTAC 5982
                   ***** ** ****  ** ** ********************** ** ** ** ** ***

P6-740             CTCCAAGATGGCCTCATAGCCTCGCTCTATCGACCTGAGGCCGATAAGGTAGCAGCCATT 6060
CPC0740            CTCCAAGATGGCCTCATAGCTTCGCTCTATCGACCTGAGGCCGACAAAGTAGCAGCTATT 6060
YAP2007            CTCCAAGATGGCCTCATAGCCTCGCTCTATCGACCTGAGGCCGACAAAGTAGCAGCTATT 6060
FSS13025           CTCCAAGATGGCCTCATAGCCTCGCTCTATCGACCTGAGGCCGACAAAGTAGCAGCTATT 6060
PLCal_ZV           CTCCAAGATGGCCTCATAGCCTCGCTCTATCGACCTGAGGCCGACAAGGTAGCAGCTATT 5949
SV0127/14          CTCCAAGATGGCCTCATAGCCTCGCTCTATCGACCTGAGGCCGACAAAGTAGCAGCCATT 6060
8375               CTCCAAGATGGCCTCATAGCCTCGCTCTATCGACCTGAGGCCGACAAAGTAGCAGCCATT 6060
103344             CTCCAAGATGGCCTCATAGCCTCGCTCTATCGACCTGAGGCCGACAAAGTAGCAGCCATT 6060
BrasilZKV2015      CTCCAAGATGGCCTCATAGCCTCGCTCTATCGACCTGAGGCCGACAAAGTAGCAGCCATT 6060
GD01               CTCCAAGATGGCCTCATAGCCTCGCTCTATCGACCTGAGGCCGACAAAGTAGCAGCCATT 6060
Martinique         CTCCAAGATGGCCTCATAGCCTCGCTCTATCGACCTGAGGCCGACAAAGTAGCAGCCATT 6060
NatalRGN           CTCCAAGATGGCCTCATAGCCTCGCTCTATCGACCTGAGGCCGACAAAGTAGCAGCCATT 6060
HPF2013            CTCCAAGATGGCCTCATAGCCTCGCTCTATCGACCTGAGGCCGACAAAGTAGCAGCCATT 6060
SPH2015            CTCCAAGATGGCCTCATAGCCTCGCTCTATCGACCTGAGGCCGACAAAGTAGCAGCCATT 6060
Haiti2014          CTCCAAGATGGCCTCATAGCCTCGCTCTATCGACCTGAGGCCGACAAAGTAGCAGCCATT 6060
PRVABC59           CTCCAAGATGGCCTCATAGCCTCGCTCTATCGACCTGAGGCCGACAAAGTAGCAGCCATT 6060
BeH819015          CTCCAAGATGGCCTCATAGCCTCGCTCTATCGACCTGAGGCCGACAAAGTAGCAGCCATT 6060
Z1106033           CTCCAAGATGGCCTCATAGCCTCGCTCTATCGACCTGAGGCCGACAAAGTAGCAGCCATT 6060
BEH819966          CTCCAAGATGGCCTCATAGCCTCGCTCTATCGACCTGAGGCCGACAAAGTAGCAGCCATT 6060
SSABr              CTCCAAGATGGCCTCATAGCCTCGCTCTATCGACCTGAGGCCGACAAAGTAGCAGCCATT 6060
Beh815744          CTCCAAGATGGCCTCATAGCCTCGCTCTATCGACCTGAGGCCGACAAAGTAGCAGCCATT 6060
BEH818995          CTCCAAGATGGCCTCATAGCCTCGCTCTATCGACCTGAGGCCGACAAAGTAGCAGCCATT 6060
IbH30656           CTCCAGGATGGCCTCATAGCCTCGCTCTATCGGCCTGAGGCTGACAAGGTTGCCGCCATT 6042
ArD128000          CTCCAAGATGGCCTCATAGCCTCGCTCTATCGGCCTGAGGCCGACAAGGTAGCCGCCATT 6060
ArD7117            CTCCAGGATGGCCTCATAGCCTCGCTCTATCGGCCTGAGGCCGACAAGGTAGCCGCCATT 6060
ARD_41519          CTCCAGGATGGCCTCATAGCCTCGCTCTATCGGCCTGAGGCCGACAAGGTAGCCGCCATT 6060
ARD157995          CTCCAGGATGGCCTCATAGCCTCGCTCTATCGGCCTGAGGCCGATAAGGTAGCCGCCATT 6060
MR_766             CTCCAGGATGGCCTCATAGCCTCGCTCTATCGGCCTGAGGCCGATAAGGTAGCCGCCATT 6048
ARD158084          CTCCAGGATGGCCTCATAGCCTCGCTCTATCGGCCTGAGGCCGATAAGGTAGCCGCCATT 6060
ArB1362            CTCCAGGATGGCCTCATAGCCTCGCTCTATCGGCCTGAGGCCGATAAGGTGGCCGCCATT 6060
ARB13565           CTCCAGGATGGCCTCATAGCCTCGCTCTACCGGCCTGAGGCCGATAAGGTAGCTGCCATT 6060
```

```
ARB7701          CTCCAGGATGGCCTCATAGCCTCGCTCTACCGGCCTGAGGCCGATAAGGTAGCTGCCATT 6060
ARB15076         CTCCAGGATGGCCTCATAGCTTCGCTCTACCGGCCTGAGGCCGACAAGGTAGCCGCCATT 6042
                 ***** ************** ******** ** ******** ** ** ** ** ** ***

P6-740           GAGGGAGAGTTCAAGCTTAGGACGGAGCAAAGGAAGACCTTTGTGGAACTCATGAAAAGA 6120
CPC0740          GAGGGAGAGTTCAAGCTTAGGACGGAGCAAAGGAAGACCTTTGTGGAACTCATGAAAAGA 6120
YAP2007          GAGGGAGAGTTCAAGCTTAGGACGGAGCAAAGGAAGACCTTTGTGGAACTCATGAAAAGA 6120
FSS13025         GAGGGAGAGTTCAAGCTTAGGACGGAGCAAAGGAAGACCTTTGTGGAACTCATGAAAAGA 6120
PLCal_ZV         GAGGGAGAGTTCAAGCTTAGGACGGAGCAAAGGAAGACCTTTGTGGAACTCATGAAAAGA 6009
SV0127/14        GAGGGAGAGTTCAAGCTTAGGACGGAGCAAAGGAAGACCTTTGTGGAACTCATGAAAAGA 6120
8375             GAGGGAGAGTTCAAGCTTAGGACGGAGCAAAGGAAGACCTTTGTGGAACTCATGAAAAGA 6120
103344           GAGGGAGAGTTCAAGCTTAGGACGGAGCAAAGGAAGACCTTTGTGGAACTCATGAAAAGA 6120
BrasilZKV2015    GAGGGAGAGTTCAAGCTTAGGACGGAGCAAAGGAAGACCTTTGTGGAACTCATGAAAAGA 6120
GD01             GAGGGAGAGTTCAAGCTTAGGACGGAGCAAAGGAAGACCTTTGTGGAACTCATGAAAAGA 6120
Martinique       GAGGGAGAGTTCAAGCTTAGGACGGAGCAAAGGAAGACCTTTGTGGAACTCATGAAAAGA 6120
NatalRGN         GAGGGAGAGTTCAAGCTTAGGACGGAGCAAAGGAAGACCTTTGTGGAACTCATGAAAAGA 6120
HPF2013          GAGGGAGAGTTCAAGCTTAGGACGGAGCAAAGGAAGACCTTTGTGGAACTCATGAAAAGA 6120
SPH2015          GAGGGAGAGTTCAAGCTTAGGACGGAGCAAAGGAAGACCTTTGTGGAACTCATGAAAAGA 6120
Haiti2014        GAGGGAGAGTTCAAGCTTAGGACGGAGCAAAGGAAGACCTTTGTGGAACTCATGAAAAGA 6120
PRVABC59         GAGGGAGAGTTCAAGCTTAGGACGGAGCAAAGGAAGACCTTTGTGGAACTCATGAAAAGA 6120
BeH819015        GAGGGAGAGTTCAAGCTTAGGACGGAGCAAAGGAAGACCTTTGTGGAACTCATGAAAAGA 6120
Z1106033         GAGGGAGAGTTCAAGCTTAGGACGGAGCAAAGGAAGACCTTTGTGGAACTCATGAAAAGA 6120
BEH819966        GAGGGAGAGTTCAAGCTTAGGACGGAGCAAAGGAAGACCTTTGTGGAACTCATGAAAAGA 6120
SSABr            GAGGGAGAGTTCAAGCTTAGGACGGAGCAAAGGAAGACCTTTGTGGAACTCATGAAAAGA 6120
Beh815744        GAGGGAGAGTTCAAGCTTAGGACGGAGCAAAGGAAGACCTTTGTGGAACTCATGAAAAGA 6120
BEH818995        GAGGGAGAGTTCAAGCTTAGGACGGAGCAAAGGAAGACCTTTGTGGAACTCATGAAAAGA 6120
IbH30656         GAGGGAGAGTTCAAGCTGAGGACAGAGCAAAGGAAGACCTTTGTGGAACTCATGAAGAGA 6102
ArD128000        GAGGGAGAGTTCAAGCTGAGGACAGAGCAAAGGAAGACCTTTGTGGAACTCATGAAGAGA 6120
ArD7117          GAGGGAGAGTTCAAGCTGAGGACAGAGCAAAGGAAGACCTTTGTGGAACTCATGAAGAGA 6120
ARD_41519        GAGGGAGAGTTCAAGCTGAGGACAGAGCAAAGGAAGACCTTTGTGGAACTCATGAAGAGA 6120
ARD157995        GAGGGAGAGTTTAAGCTGAGGACAGAGCAAAGGAAGACCTTCGTGGAACTCATGAAGAGA 6120
MR_766           GAGGGAGAGTTTAAGCTGAGGACAGAGCAAAGGAAGACCTTCGTGGAACTCATGAAGAGA 6108
ARD158084        GAGGGAGAGTTTAAGCTGAGGACAGAGCAAAGGAAGACCTTCGTGGAACTCATGAAGAGA 6120
ArB1362          GAGGGAGAGTTTAAGCTGAGGACAGAGCAAAGGAAGACCTTTGTGGAACTTATGAAGAGA 6120
ARB13565         GAGGGAGAGTTCAAGCTGAGGACAGAGCAAAGGAAAACCTTTGTGGAACTTATGAAGAGA 6120
ARB7701          GAGGGAGAGTTCAAGCTGAGGACAGAGCAAAGGAAAACCTTTGTGGAACTTATGAAGAGA 6120
ARB15076         GAGGGAGAGTTCAAGCTGAGGACGGAGCAAAGGAAAACCTTTGTGGAACTTATGAAGAGA 6102
                 *********** ***** ***** *********** ***** ******** ***** ***

P6-740           GGAGATCTTCCTGTTTGGCTGGCCTATCAGGTTGCATCTGCCGGAATAACCTACACAGAT 6180
CPC0740          GGAGATCTTCCGGTTTGGTTGGCCTATCAGGTTGCATCTGCCGGAATAACCTACACAGAT 6180
YAP2007          GGAGATCTTCCTGTTTGGCTGGCCTATCAGGTTGCATCTGCCGGAATAACCTACACAGAT 6180
FSS13025         GGAGATCTTCCTGTTTGGCTGGCCTATCAGGTTGCATCTGCCGGAATAACCTACACAGAT 6180
PLCal_ZV         GGAGATCTTCCTGTTTGGCTGGCCTATCAGGTTGCATCTGCCGGAATTACCTACACAGAT 6069
SV0127/14        GGAGATCTTCCTGTTTGGCTGGCCTATCAGGTTGCATCTGCCGGAATAACCTACACAGAT 6180
8375             GGAGATCTTCCTGTTTGGCTGGCCTATCAGGTTGCATCTGCCGGAATAACCTACACAGAT 6180
103344           GGAGATCTTCCTGTTTGGCTGGCCTATCAGGTTGCATCTGCCGGAATAACCTACACAGAT 6180
BrasilZKV2015    GGAGATCTTCCTGTTTGGCTGGCCTATCAGGTTGCATCTGCCGGAATAACCTACACAGAT 6180
GD01             GGAGATCTTCCTGTTTGGCTGGCCTATCAGGTTGCATCTGCCGGAATAACCTACACAGAT 6180
Martinique       GGAGATCTTCCTGTTTGGCTGGCCTATCAGGTTGCATCTGCCGGAATAACCTACACAGAT 6180
NatalRGN         GGAGATCTTCCTGTTTGGCTGGCCTATCAGGTTGCATCTGCCGGAATAACCTACACAGAT 6180
HPF2013          GGAGATCTTCCTGTTTGGCTGGCCTATCAGGTTGCATCTGCCGGAATAACCTACACAGAT 6180
SPH2015          GGAGATCTTCCTGTTTGGCTGGCCTATCAGGTTGCATCTGCCGGAATAACCTACACAGAT 6180
Haiti2014        GGAGATCTTCCTGTTTGGCTGGCCTATCAGGTTGCATCTGCCGGAATAACCTACACAGAT 6180
PRVABC59         GGAGATCTTCCTGTTTGGCTGGCCTATCAGGTTGCATCTGCCGGAATAACCTACACAGAT 6180
BeH819015        GGAGATCTTCCTGTTTGGCTGGCCTATCAGGTTGCATCTGCCGGAATAACCTACACAGAT 6180
Z1106033         GGAGATCTTCCTGTTTGGCTGGCCTATCAGGTTGCATCTGCCGGAATAACCTACACAGAT 6180
BEH819966        GGAGATCTTCCTGTTTGGCTGGCCTATCAGGTTGCATCTGCCGGAATAACCTACACAGAT 6180
SSABr            GGAGATCTTCCTGTTTGGCTGGCCTATCAGGTTGCATCTGCCGGAATAACCTACACAGAT 6180
Beh815744        GGAGATCTTCCTGTTTGGCTGGCCTATCAGGTTGCATCTGCCGGAATAACCTACACAGAT 6180
BEH818995        GGAGATCTTCCTGTTTGGCTGGCCTATCAGGTTGCATCTGCCGGAATAACCTACACAGAT 6180
IbH30656         GGAGACCTTCCCGTTTGGCTGGCCTATCAAGTAGCATCTGCCGGAATAACTTACACAGAC 6162
ArD128000        GGAGACCTTCCCGTTTGGCTGGCCTATCAAGTAGCATCTGCCGGAATAACTTACACAGAC 6180
ArD7117          GGAGACCTTCCCGTTTGGCTGGCCTATCAAGTAGCATCTGCCGGAATAACTTACACAGAC 6180
ARD_41519        GGAGACCTTCCCGTTTGGCTGGCCTATCAAGTAGCATCTGCCGGAATAACTTACACAGAT 6180
ARD157995        GGAGACCTTCCCGTCTGGCTAGCCTATCAGGTTGCATCTGCCGGAATAACTTACACAGAC 6180
MR_766           GGAGACCTTCCCGTCTGGCTAGCCTATCAGGTTGCATCTGCCGGAATAACTTACACAGAC 6168
ARD158084        GGAGACCTTCCCGTCTGGCTAGCCTATCAGGTTGCATCTGCCGGAATAACTTACACAGAC 6180
```

```
ArB1362         GGAGACCTTCCCGTTTGGCTAGCCTATCAGGTTGCATCTGCCGGAATAACTTACACAGAC 6180
ARB13565        GGAGACCTTCCCGTTTGGCTAGCCTATCAGGTTGCATCTGCCGGAATAACTTACACAGAC 6180
ARB7701         GGAGACCTTCCCGTTTGGCTAGCCTATCAGGTTGCATCTGCCGGAATAACTTACACAGAC 6180
ARB15076        GGAGACCTTCCCGTTTGGCTGGCCTATCAGGTTGCATCTGCCGGAATAACTTACACAGAC 6162
                ***** ***** ** *** * ******** ** ************** ** ********

P6-740          AGAAGATGGTGTTTTGATGGCACGACCAACAACACCATAATGGAAGACAGTGTGCCGGCA 6240
CPC0740         AGAAGATGGTGCTTTGATGGCATGACCAACAACACCATAATGGAAGACAGTGTGCCGGCA 6240
YAP2007         AGAAAATGGTGCTTTGATGGCACGACCAACAACACCATAATGGAAGACAGTGTGCCGGCA 6240
FSS13025        AGAAGATGGTGCTTTGATGGCACGACCAACAACACCATAATGGAAGACAGTGTGCCGGCA 6240
PLCal_ZV        AGAAGATGGTGCTTTGATGGCACGACCAACAACACCATAATGGAAGACAGTGTGCCGGCA 6129
SV0127/14       AGAAGATGGTGCTTTGATGGCATGACCAACAACACCATAATGGAAGACAGTGTGCCGGCA 6240
8375            AGAAGATGGTGCTTTGATGGCACGACCAACAACACCATACTGGAAGACAGTGTGCCGGCA 6240
103344          AGAAGATGGTGCTTTGATGGCACGACCAACAACACCATACTGGAAGACAGTGTGCCGGCA 6240
BrasilZKV2015   AGAAGATGGTGCTTTGATGGCACGACCAACAACACCATAATGGAAGACAGTGTGCCGGCA 6240
GD01            AGAAGATGGTGCTTTGATGGCACGACCAACAACACCATAATGGAAGACAGTGTGCCGGCA 6240
Martinique      AGAAGATGGTGCTTTGATGGCACGACCAACAACACCATAATGGAAGACAGTGTGCCGGCA 6240
NatalRGN        AGAAGATGGTGCTTTGATGGCACGACCAACAACACCATAATGGAAGACAGTGTGCCGGCA 6240
HPF2013         AGAAGATGGTGCTTTGATGGCACGACCAACAACACCATAATGGAAGACAGTGTGCCGGCA 6240
SPH2015         AGAAGATGGTGCTTTGATGGCACGACCAACAACACCATAATGGAAGACAGTGTGCCGGCA 6240
Haiti2014       AGAAGATGGTGCTTTGATGGCACGACCAACAACACCATAATGGAAGACAGTGTGCCGGCA 6240
PRVABC59        AGAAGATGGTGCTTTGATGGCACGACCAACAACACCATAATGGAAGACAGTGTGCCGGCA 6240
BeH819015       AGAAGATGGTGCTTTGATGGCACGACCAACAACACCATAATGGAAGATAGTGTGCCGGCA 6240
Z1106033        AGAAGATGGTGCTTTGATGGCACGACCAACAACACCATAATGGAAGACAGTGTGCCGGCA 6240
BEH819966       AGAAGATGGTGCTTTGATGGCACGACCAACAACACCATAATGGAAGACAGTGTGCCGGCA 6240
SSABr           AGAAGATGGTGCTTTGATGGCACGACCAACAACACCATAATGGAAGACAGTGTGCCGGCA 6240
Beh815744       AGAAGATGGTGCTTTGATGGCACGACCAACAACACCATAATGGAAGACAGTGTGCCGGCA 6240
BEH818995       AGAAGATGGTGCTTTGATGGCACGACCAACAACACCATAATGGAAGACAGTGTGCCGGCA 6240
IbH30656        AGAAGATGGTGCTTTGATGGCACTACCAACAACACCATAATGGAAGACAGTGTACCAGCA 6222
ArD128000       AGAAGATGGTGCTTTGATGGCACTACCAACAACACCATAATGGAAGACAGTGTACCAGCA 6240
ArD7117         AGAAGATGGTGCTTTGATGGCACTACCAACAACACCATAATGGAAGACAGTGTACCAGCA 6240
ARD_41519       AGAAGATGGTGCTTTGATGGCACTACCAACAACACCATAATGGAAGACAGTGTACCAGCA 6240
ARD157995       AGAAGATGGTGCTTTGATGGCACAACCAACAACACCATAATGGAAGACAGCGTACCAGCA 6240
MR_766          AGAAGATGGTGCTTTGATGGCACAACCAACAACACCATAATGGAAGACAGTGTACCAGCA 6228
ARD158084       AGAAGATGGTGCTTTGATGGCACAACCAACAACACCATAATGGAAGACAGTGTACCAGCA 6240
ArB1362         AGAAGATGGTGCTTTGATGGCACAACTAACAACACCATAATGGAAGACAGTGTACCAGCA 6240
ARB13565        AGAAGATGGTGCTTTGATGGCACAACCAACAACACCATAATGGAAGATAGTGTACCAGCA 6240
ARB7701         AGAAGATGGTGCTTTGATGGCACAACCAACAACACCATAATGGAAGATAGTGTACCAGCA 6240
ARB15076        AGAAGATGGTGCTTTGATGGCACAACCAACAACACCATAATGGAAGATAGTGTACCAGCA 6222
                **** ****** **********  ** ************ ******* ** ** ** ***

P6-740          GAGGTGTGGACCAGATACGGAGAGAAAAGAGTGCTCAAACCGAGGTGGATGGACGCCAGA 6300
CPC0740         GAGGTGTGGACCAGATACGGAGAGAAAAGAGTGCTCAAACCGAGGTGGATGGACGCCAGA 6300
YAP2007         GAGGTGTGGACCAGATACGGAGAGAAAAGAGTGCTCAAACCAAGGTGGATGGACGCCAGA 6300
FSS13025        GAGGTGTGGACCAGATACGGAGAGAAAAGAGTGCTCAAACCGAGGTGGATGGACGCCAGA 6300
PLCal_ZV        GAGGTGTGGACCAGATACGGAGAGAAAAGAGTGCTCAAACCGAGGTGGATGGACGCCAGA 6189
SV0127/14       GAGGTGTGGACCAGACACGGAGAGAAAAGAGTGCTCAAACCGAGGTGGATGGACGCCAGA 6300
8375            GAGGTGTGGACCAGACACGGAGAGAAAAGAGTGCTCAAACCGAGGTGGATGGACGCCAGA 6300
103344          GAGGTGTGGACCAGACACGGAGAGAAAAGAGTGCTCAAACCGAGGTGGATGGACGCCAGA 6300
BrasilZKV2015   GAGGTGTGGACCAGACACGGAGAGAAAAGAGTGCTCAAACCGAGGTGGATGGACGCCAGA 6300
GD01            GAGGTGTGGACCAGACACGGAGAGAAAAGAGTGCTCAAACCGAGGTGGATGGACGCCAGA 6300
Martinique      GAGGTGTGGACCAGACACGGAGAGAAAAGAGTGCTCAAACCGAGGTGGATGGACGCCAGA 6300
NatalRGN        GAGGTGTGGACCAGACACGGAGAGAAAAGAGTGCTCAAACCGAGGTGGATGGACGCCAGA 6300
HPF2013         GAGGTGTGGACCAGACACGGAGAGAAAAGAGTGCTCAAACCGAGGTGGATGGACGCCAGA 6300
SPH2015         GAGGTGTGGACCAGACACGGAGAGAAAAGAGTGCTCAAACCGAGGTGGATGGACGCCAGA 6300
Haiti2014       GAGGTGTGGACCAGACACGGAGAGAAAAGAGTGCTCAAACCGAGGTGGATGGACGCCAGA 6300
PRVABC59        GAGGTGTGGACCAGACACGGAGAGAAAAGAGTGCTCAAACCGAGGTGGATGGACGCCAGA 6300
BeH819015       GAGGTGTGGACCAGACACGGAGAGAAAAGAGTGCTCAAACCGAGGTGGATGGACGCCAGA 6300
Z1106033        GAAGTGTGGACCAGACACGGAGAGAAAAGAGTGCTCAAACCGAGGTGGATGGACGCCAGA 6300
BEH819966       GAGGTGTGGACCAGACACGGAGAGAAAAGAGTGCTCAAACCGAGGTGGATGGACGCCAGA 6300
SSABr           GAGGTGTGGACCAGACACGGAGAGAAAAGAGTGCTCAAACCGAGGTGGATGGACGCCAGA 6300
Beh815744       GAGGTGTGGACCAGACACGGAGAGAAAAGAGTGCTCAAACCGAGGTGGATGGACGCCAGA 6300
BEH818995       GAGGTGTGGACCAGACACGGAGAGAAAAGAGTGCTCAAACCGAGGTGGATGGACGCCAGA 6300
IbH30656        GAGGTGTGGACCAAGTATGGAGAGAAGAGAGTGCTCAAACCGAGGTGGATGGATGCCAGG 6282
ArD128000       GAGGTGTGGACCAAGTATGGAGAGAAGAGAGTGCTTAAACCGAGGTGGATGGATGCTAGG 6300
ArD7117         GAGGTGTGGACCAAGTATGGAGAGAAGAGAGTGCTTAAACCGAGGTGGATGGATGCTAGG 6300
ARD_41519       GAGGTGTGGACCAAGTATGGAGAGAAGAGAGTGCTTAAACCGAGGTGGATGGATGCTAGG 6300
ARD157995       GAGGTGTGGACAAAGTATGGAGAGAAGAGAGTGCTCAAACCGAGATGGATGGATGCTAGG 6300
```

```
MR_766          GAGGTTTGGACAAAGTATGGAGAGAAGAGAGTGCTCAAACCGAGATGGATGGATGCTAGG 6288
ARD158084       GAGGTTTGGACAAAGTATGGAGAGAAGAGAGTGCTCAAACCGAGATGGATGGATGCTAGG 6300
ArB1362         GAGGTGTGGACAAAATATGGAGAGAAGAGAGTGCTCAAACCAAGATGGATGGATGCTAGA 6300
ARB13565        GAAGTGTGGACAAAATATGGAGAGAAGAGAGTGCTCAAACCAAGATGGATGGATGCGAGG 6300
ARB7701         GAAGTGTGGACAAAATATGGAGAGAAGAGAGTGCTCAAACCAAGATGGATGGATGCGAGG 6300
ARB15076        GAGGTGTGGACAAAATATGGAGAGAAGAGAGTGCTTAAACCAAGATGGATGGATGCGAGG 6282
                ** ** ***** *   * ******** ******** ***** ** ******** ** **

P6-740          GTTTGTTCAGATCATGCGGCCCTGAAGTCATTCAAAGAATTTGCCGCTGGGAAAAGAGGA 6360
CPC0740         GTTTGTTCAGATCATGCGGCCCTGAAGTCATTCAAAGAGTTTGCCGCTGGGAAAAGAGGA 6360
YAP2007         GTTTGTTCAGATCATGCGGCCCTGAAGTCATTCAAAGAGTTTGCCGCTGGGAAAAGAGGA 6360
FSS13025        GTTTGTTCAGATCATGCGGCCCTGAAGTCATTCAAAGAGTTTGCCGCTGGGAAAAGAGGA 6360
PLCal_ZV        GTTTGTTCAGATCATGCGGCCCTGAAGTCATTCAAGGAGTTTGCCGCTGGGAAAAGAGGA 6249
SV0127/14       GTTTGTTCAGATCATGCGGCCCTGAAGTCATTCAAGGAGTTTGCCGCTGGGAAAAGAGGA 6360
8375            GTTTGTTCAGATCATGCGGCCCTGAAGTCATTCAAGGAGTTTGCCGCTGGGAAAAGAGGA 6360
103344          GTTTGTTCAGATCATGCGGCCCTGAAGTCATTCAAGGAGTTTGCCGCTGGGAAAAGAGGA 6360
BrasilZKV2015   GTTTGTTCAGATCATGCGGCCCTGAAGTCATTCAAGGAGTTTGCCGCTGGGAAAAGAGGA 6360
GD01            GTTTGTTCAGATCATGCGGCCCTGAAGTCATTCAAGGAGTTTGCCGCTGGGAAAAGAGGA 6360
Martinique      GTTTGTTCAGATCATGCGGCCCTGAAGTCATTCAAGGAGTTTGCCGCTGGGAAAAGAGGA 6360
NatalRGN        GTTTGTTCAGATCATGCGGCCCTGAAGTCATTCAAGGAGTTTGCCGCTGGGAAAAGAGGA 6360
HPF2013         GTTTGTTCAGATCATGCGGCCCTGAAGTCATTCAAGGAGTTTGCCGCTGGGAAAAGAGGA 6360
SPH2015         GTTTGTTCAGATCATGCGGCCCTGAAGTCATTCAAGGAGTTTGCCGCTGGGAAAAGAGGA 6360
Haiti2014       GTTTGTTCAGATCATGCGGCCCTGAAGTCATTCAAGGAGTTTGCCGCTGGGAAAAGAGGA 6360
PRVABC59        GTTTGTTCAGATCATGCGGCCCTGAAGTCATTCAAGGAGTTTGCCGCTGGGAAAAGAGGA 6360
BeH819015       GTTTGTTCAGATCATGCGGCCCTGAAGTCATTCAAGGAGTTTGCCGCTGGGAAAAGAGGA 6360
Z1106033        GTTTGTTCAGATCATGCGGCCCTGAAGTCATTCAAGGAGTTTGCCGCTGGGAAAAGAGGA 6360
BEH819966       GTTTGTTCAGATCATGCGGCCCTGAAGTCATTCAAGGAGTTTGCCGCTGGGAAAAGAGGA 6360
SSABr           GTTTGTTCAGATCATGCGGCCCTGAAGTCATTCAAGGAGTTTGCCGCTGGGAAAAGAGGA 6360
Beh815744       GTTTGTTCAGATCATGCGGCCCTGAAGTCATTCAAGGAGTTTGCCGCTGGGAAAAGAGGA 6360
BEH818995       GTTTGTTCAGATCATGCGGCCCTGAAGTCATTCAAGGAGTTTGCCGCTGGGAAAAGAGGA 6360
IbH30656        GTCTGTTCAGATCATGCGGCTTTGAAGTCGTTCAAAGAATTTGCCGCTGGGAAGAGAGGA 6342
ArD128000       GTCTGTTCAGATCATGCGGCTCTGAAGTCGTTCAAAGAATTTGCCGCTGGGAAGAGAGGA 6360
ArD7117         GTCTGTTCAGATCATGCGGCTTTGAAGTCGTTCAAAGAATTTGCCGCTGGGAAGAGAGGA 6360
ARD_41519       GTCTGTTCAGATCATGCGGCTCTGAAGTCGTTCAAAGAATTTGCCGCTGGGAAGAGAGGA 6360
ARD157995       GTCTGTTCAGACCATGCGGCCCTGAAGTCGTTCAAAGAATTCGCCGCTGGAAAAAGAGGA 6360
MR_766          GTCTGTTCAGACCATGCGGCCCTGAAGTCGTTCAAAGAATTCGCCGCTGGAAAAAGAGGA 6348
ARD158084       GTCTGTTCAGACCATGCGGCCCTGAAGTCGTTCAAAGAATTCGCCGCTGGAAAAAGAGGA 6360
ArB1362         GTCTGTTCAGATCATGCCGCCCTGAAGTCGTTCAAAGAGTTTGCTGCCGGGAAAAGAGGA 6360
ARB13565        GTCTGTTCCGATCATGCTGCCCTGAAGTCGTTCAAAGAATTTGCTGCCGGGAAAAGAGGA 6360
ARB7701         GTCTGTTCCGATCATGCTGCCCTGAAGTCGTTCAAAGAATTTGCTGCCGGGAAAAGAGGA 6360
ARB15076        GTCTGTTCTGATCATGCTGCCCTGAAGTCGTTCAAAGAATTTGCTGCCGGGAAAAGAGGA 6342
                ** ***** ** ***** **  ******* ***** ** ** ** ** ** ** ******

P6-740          GCGGCCTTTGGAGTGATGGAAGCCCTGGGAACACTGCCAGGACACATGACAGAGAGGTTT 6420
CPC0740         GCGGCCTTTGGAGTGATAGAAGCCCTGGGAACACTGCCAGGACACATGACAGAGAGATTC 6420
YAP2007         GcGGCCTTTGGAGTGATGGAAGCCCTGGGAACATTGCCGGGACACATGACAGAGAGATTC 6420
FSS13025        GCGGCCTTTGGAGTGATGGAAGCCCTGGGAACACTGCCAGGACATATGACAGAGAGATTC 6420
PLCal_ZV        GCGGCATTTGGAGTGATGGAAGCCCTGGGAACACTGCCAGGACACATGACAGAGAGATTC 6309
SV0127/14       GCGGCTTTTGGAGTGATGGAAGCCCTGGGAACACTGCCAGGACACATGACGGAGAGATTC 6420
8375            GCGGCTTTTGGAGTGATGGAAGCCTTGGGAACACTGCCAGGACACATGACAGAGAGATTC 6420
103344          GCGGCTTTTGGAGTGATGGAAGCCTTGGGAACACTGCCAGGACACATGACAGAGAGATTC 6420
BrasilZKV2015   GCGGCTTTTGGAGTGATGGAAGCCCTGGGAACACTGCCAGGACACATGACAGAGAGATTC 6420
GD01            GCGGCTTTTGGAGTGATGGAAGCCCTGGGAACACTGCCAGGACACATGACAGAGAGATTC 6420
Martinique      GCGGCTTTTGGAGTGATGGAAGCCCTGGGAACACTGCCAGGACACATGACAGAGAGATTC 6420
NatalRGN        GCGGCTTTTGGAGTGATGGAAGCCCTGGGAACACTGCCAGGACACATGACAGAGAGATTC 6420
HPF2013         GCGGCTTTTGGAGTGATGGAAGCCCTGGGAACACTGCCAGGACACATGACAGAGAGATTC 6420
SPH2015         GCGGCTTTTGGAGTGATGGAAGCCCTGGGAACACTGCCAGGACACATGACAGAGAGATTC 6420
Haiti2014       GCGGCTTTTGGAGTGATGGAAGCCCTGGGAACACTGCCAGGACACATGACAGAGAGATTC 6420
PRVABC59        GCGGCTTTTGGAGTGATGGAAGCCCTGGGAACACTGCCAGGACACATGACAGAGAGATTC 6420
BeH819015       GCGGCTTTTGGAGTGATGGAAGCCCTGGGAACACTGCCAGGACACATGACAGAGAGATTC 6420
Z1106033        GCGGCTTTTGGAGTGATGGAAGCCCTGGGAACACTGCCAGGACACATGACAGAGAGATTC 6420
BEH819966       GCGGCTTTTGGAGTGATGGAAGCCCTGGGAACACTGCCAGGACACATGACAGAGAGATTC 6420
SSABr           GCGGCTTTTGGAGTGATGGAAGCCCTGGGAACACTGCCAGGACACATGACAGAGAGATTC 6420
Beh815744       GCGGCTTTTGGAGTGATGGAAGCCCTGGGAACACTGCCAGGACACATGACAGAGAGATTC 6420
BEH818995       GCGGCTTTTGGAGTGATGGAAGCCCTGGGAACACTGCCAGGACACATGACAGAGAGATTC 6420
IbH30656        GCGGCTTTGGGAGTAATGGATGCCCTAGGAACATTGCCAGGACACATGACAGAGAGGTTT 6402
ArD128000       GCGGCTTTGGGAGTAATGGATGCCCTGGGAACATTGCCAGGACACATGACAGAGAGGTTT 6420
ArD7117         GCGGCTTTGGGAGTGATGGATGCCCTGGGAACATTGCCAGGACACATGACAGAGAGGTTT 6420
```

```
ARD_41519          GCGGCTTTGGGAGTAATGGATGCCCTGGGAACATTGCCAGGACACATGACAGAGAGGTTT 6420
ARD157995          GCGGCTTTGGGAGTAATGGAGGCCCTGGGAACACTGCCAGGACACATGACAGAGAGGTTT 6420
MR_766             GCGGCTTTGGGAGTAATGGAGGCCCTGGGAACACTGCCAGGACACATGACAGAGAGGTTT 6408
ARD158084          GCGGCTTTGGGAGTAATGGAGGCCCTGGGAACACTGCCAGGACACATGACAGAGAGGTTT 6420
ArB1362            GCGGCTTTGGGAGTAATGGAGGCCCTGGGAACATTGCCAGGACACATGACAGAGAGGTTT 6420
ARB13565           GTGGCTTTAGGAGTAATGGAGGCCCTGGGAACATTGCCAGGACACATGACAGAGAGGTTT 6420
ARB7701            GTGGCTTTAGGAGTAATGGAGGCCCTGGGAACATTGCCAGGACACATGACAGAGAGGTTT 6420
ARB15076           GCGGCTTTAGGAGTAATGGAGGCCCTGGGAACATTACCAGGACACATGACAGAGAGGTTT 6402
                   * *** ** ***** ** ** *** * ****** * ** ***** ***** ***** **

P6-740             CAGGAAGCCATTGACAACCTCGCTGTGCTCATGCGGGCAGAGACTGGAAGCAGGCCCTAC 6480
CPC0740            CAGGAAGCCATTGACAACCTCGCTGTGCTCATGCGGGCAGAGACTGGAAGCAGGCCTTAC 6480
YAP2007            CAGGAAGCCATTGACAACCTCGCTGTGCTCATGCGGGCAGAGACTGGAAGCAGGCCTTAC 6480
FSS13025           CAGGAGGCCATTGACAACCTCGCTGTGCTCATGCGGGCAGAGACTGGAAGCAGGCCCTAC 6480
PLCal_ZV           CAGGAAGCCATTGACAACCTCGCTGTGCTCATGCGGGCAGAGACTGGAAGCAGGCCTTAC 6369
SV0127/14          CAGGAAGCCATTGACAACCTCGCTGTGCTCATGCGGGCAGAGACTGGAAGCAGGCCTTAC 6480
8375               CAGGAAGCCATTGACAACCTCGCTGTGCTCATGCGGGCAGAGACTGGAAGCAGGCCTTAC 6480
103344             CAGGAAGCCATTGACAACCTCGCTGTGCTCATGCGGGCAGAGACTGGAAGCAGGCCTTAC 6480
BrasilZKV2015      CAGGAAGCCATTGACAACCTCGCTGTGCTCATGCGGGCAGAGACTGGAAGCAGGCCTTAC 6480
GD01               CAGGAAGCCATTGACAACCTCGCTGTGCTCATGCGGGCAGAGACTGGAAGCAGGCCTTAC 6480
Martinique         CAGGAAGCCATTGACAACCTCGCTGTGCTCATGCGGGCAGAGACTGGAAGCAGGCCTTAC 6480
NatalRGN           CAGGAAGCCATTGACAACCTCGCTGTGCTCATGCGGGCAGAGACTGGAAGCAGGCCTTAC 6480
HPF2013            CAGGAAGCCATTGACAACCTCGCTGTGCTCATGCGGGCAGAGACTGGAAGCAGGCCTTAC 6480
SPH2015            CAGGAAGCCATTGACAACCTCGCTGTGCTCATGCGGGCAGAGACTGGAAGCAGGCCTTAC 6480
Haiti2014          CAGGAAGCCATTGACAACCTCGCTGTGCTCATGCGGGCAGAGACTGGAAGCAGGCCTTAC 6480
PRVABC59           CAGGAAGCCATTGACAACCTCGCTGTGCTCATGCGGGCAGAGACTGGAAGCAGGCCTTAC 6480
BeH819015          CAGGAAGCCATTGACAACCTCGCTGTGCTCATGCGGGCAGAGACTGGAAGCAGGCCTTAC 6480
Z1106033           CAGGAAGCCATTGACAACCTCGCTGTGCTCATGCGGGCAGAGACTGGAAGCAGGCCTTAC 6480
BEH819966          CAGGAAGCCATTGACAACCTCGCTGTGCTCATGCGGGCAGAGACTGGAAGCAGGCCTTAC 6480
SSABr              CAGGAAGCCATTGACAACCTCGCTGTGCTCATGCGGGCAGAGACTGGAAGCAGGCCTTAC 6480
Beh815744          CAGGAAGCCATTGACAACCTCGCTGTGCTCATGCGGGCAGAGACTGGAAGCAGGCCTTAC 6480
BEH818995          CAGGAAGCCATTGACAACCTCGCTGTGCTCATGCGGGCAGAGACTGGAAGCAGGCCTTAC 6480
IbH30656           CAGGAAGCCATTGACAATCTCGCTGTGCTCATGCGAGCAGAGACTGGAAGTAGGCCCTAC 6462
ArD128000          CAGGAAGCCATTGACAATCTTGCTGTGCTCATGCGAGCAGAGACTGGAAGTAGGCCCTAC 6480
ArD7117            CAGGAAGCCATTGACAATCTCGCTGTGCTCATGCGAGCAGAGACTGGAAGTAGGCCCTAC 6480
ARD_41519          CAGGAAGCCATTGACAATCTCGCTGTGCTCATGCGAGCAGAGACTGGAAGTAGGCCCTAC 6480
ARD157995          CAGGAAGCCATTGACAACCTCGCCGTGCTCATGCGAGCAGAGACTGGAAGCAGGCCTTAT 6480
MR_766             CAGGAAGCCATTGACAACCTCGCCGTGCTCATGCGAGCAGAGACTGGAAGCAGGCCTTAT 6468
ARD158084          CAGGAAGCCATTGACAACCTCGCCGTGCTCATGCGAGCAGAGACTGGAAGCAGGCCTTAT 6480
ArB1362            CAGGAAGCCATTGACAATCTCGCTGTGCTTATGCGAGCAGAAACTGGAAGCAGGCCTTAC 6480
ARB13565           CAGGAGGCCATTGACAATCTCGCTGTGCTCATGCGAGCAGAGACTGGAAGCAGGCCTTAC 6480
ARB7701            CAGGAGGCCATTGACAATCTCGCTGTGCTCATGCGAGCAGAGACTGGAAGCAGGCCTTAC 6480
ARB15076           CAGGAGGCCATTGACAATCTCGCTGTGCTTATGCGAGCAGAGACTGGAAGTAGGCCTTAC 6462
                   ***** *********** ** ** ***** ***** ***** ******** ***** **

P6-740             AAAGCCGCGGCGGCCCAATTACCGGAGACCTTAGAGACCATCATGCTTTTGGGTTTGCTG 6540
CPC0740            AAAGCCGCGGCGGCCCAATTGCCGGAGACCCTAGAGACCATTATGCTTTTGGGGTTGCTG 6540
YAP2007            GAAGCCGCGGCGGCCCAATTGCCGGAGACCTTAGAGACCATCATGCTTTTGGGGTTGCTG 6540
FSS13025           AAAGCCGCGGCGGCCCAATTACCGGAGACCCTAGAGACTATCATGCTTTTGGGGTTGCTG 6540
PLCal_ZV           AAAGCCGCGGCGGCCCAATTGCCGGAGACCCTAGAGACCATTATGCTTTTGGGGTTGCTG 6429
SV0127/14          AAAGCCGCGGCGGCCCAATTGCCGGAGACCCTAGAGACCATTATGCTTTTGGGGTTGCTG 6540
8375               AAAGCCGCGGCGGCCCAATTGCCGGAGACCCTAGAGACCATTATGCTTTTGGGGTTGCTG 6540
103344             AAAGCCGCGGCGGCCCAATTGCCGGAGACCCTAGAGACCATTATGCTTTTGGGGTTGCTG 6540
BrasilZKV2015      AAAGCCGCGGCGGCCCAATTGCCGGAGACCCTAGAGACCATTATGCTTTTGGGGTTGCTG 6540
GD01               AAAGCCGCGGCAGCCCAATTGCCGGAGACCCTAGAGACCATTATGCTTTTGGGGTTGCTG 6540
Martinique         AAAGCCGCGGCGGCCCAATTGCCGGAGACCCTAGAGACCATTATGCTTTTGGGGTTGCTG 6540
NatalRGN           AAAGCCGCGGCGGCCCAATTGCCGGAGACCCTAGAGACCATTATGCTTTTGGGGTTGCTG 6540
HPF2013            AAAGCCGCGGCGGCCCAATTGCCGGAGACCCTAGAGACCATTATGCTTTTGGGGTTGCTG 6540
SPH2015            AAAGCCGCGGCGGCCCAATTGCCGGAGACCCTAGAGACCATTATGCTTTTGGGGTTGCTG 6540
Haiti2014          AAAGCCGCGGCGGCCCAATTGCCGGAGACCCTAGAGACCATTATGCTTTTGGGGTTGCTG 6540
PRVABC59           AAAGCCGCGGCGGCCCAATTGCCGGAGACCCTAGAGACCATAATGCTTTTGGGGTTGCTG 6540
BeH819015          AAAGCCGCGGCGGCCCAATTGCCGGAGACCCTAGAGACCATAATGCTTTTGGGGTTGCTG 6540
Z1106033           AAAGCCGCGGCGGCCCAATTGCCGGAGACCCTAGAGACCATTATGCTTTTGGGGTTGCTG 6540
BEH819966          AAAGCCGCGGCGGCCCAATTGCCGGAGACCCTAGAGACCATTATGCTTTTGGGGTTGCTG 6540
SSABr              AAAGCCGCGGCGGCCCAATTGCCGGAGACCCTAGAGACCATTATGCTTTTGGGGTTGCTG 6540
Beh815744          AAAGCCGCGGCGGCCCAATTGCCGGAGACCCTAGAGACCATTATGCTTTTGGGGTTGCTG 6540
BEH818995          AAAGCCGCGGCGGCCCAATTGCCGGAGACCCTAGAGACCATTATGCTTTTGGGGTTGCTG 6540
IbH30656           AAAGCAGCGGCAGCTCAACTGCCGGAGACCCTAGAGACCATTATGCTCTTGGGTTTATTG 6522
```

```
ArD128000          AAAGCAGCGGCAGCTCAACTGCCGGAGACCCTAGAGACCATCATGCTTTTGGGCTTATTG 6540
ArD7117            AAAGCAGCGGCAGCTCAACTGCCGGAGACCCTAGAGACCATCATGCTTTTGGGCTTATTG 6540
ARD_41519          AAAGCAGCGGCAGCTCAACTGCCGGAGACCTTAGAGACTATCATGCTTCTGGGCTTATTG 6540
ARD157995          AAGGCAGCGGCAGCCCAACTGCCGGAGACCCTAGAGACCATTATGCTCTTAGGTTTGCTG 6540
MR_766             AAGGCAGCGGCAGCCCAACTGCCGGAGACCCTAGAGACCATTATGCTCTTAGGTTTGCTG 6528
ARD158084          AAGGCAGCGGCAGCCCAACTGCCGGAGACCCTAGAGACCATTATGCTCTTAGGTTTGCTG 6540
ArB1362            AAGGCAGCGGCAGCCCCACTGCCGGAGACCCTAGAGACCATCATGCTCTTAGGTTTGCTG 6540
ARB13565           AAGGCAGCGGCAGCCCAACTGCCGGAGACCCTAGAGACCATCATGCTCTTAGGCTTGTTG 6540
ARB7701            AAGGCAGCGGCAGCCCAACTGCCGGAGACCCTAGAGACCATCATGCTCTTAGGCTTGTTG 6540
ARB15076           AAGGCAGCGGCAGCCCAACTGCCGGAGACCCTAGAGACCATCATGCTCCTAGGTTTGCTG 6522
                    * ** ***** ** * * * ********* ******* ** *****  * ** **  **

P6-740             GGAACAGTCTCGCTGGGAATCTTCTTTGTCTTGATGCGGAACAAGGGCATAGGGAAGATG 6600
CPC0740            GGAACAGTCTCGCTGGGAATCTTTTTCGTCTTGATGCGGAACAAGGGCATGGGGAAGATG 6600
YAP2007            GGAACAGTCTCGCTGGGAATCTTTTTCGTCTTGATGCGGAATAAGGGCATCGGGAAGATG 6600
FSS13025           GGAACAGTCTCGCTGGGAATCTTTTTCGTCTTGATGCGGAACAAGGGCATAGGGAAGATG 6600
PLCal_ZV           GGAACAGTCTCGCTGGGAATCTTTTTCGTCTTGATGCGGAACAAGGGCATAGGGAAGATG 6489
SV0127/14          GGAACAGTCTCGCTGGGAATCTTTTTCGTCTTGATGCGGAACAAGGGCATAGGGAAGATG 6600
8375               GGAACAGTCTCGCTGGGAATCTTTTTCGTCTTGATGAGGAACAAGGGCATAGGGAAGATG 6600
103344             GGAACAGTCTCGCTGGGAATCTTTTTCGTCTTGATGAGGAACAAGGGCATAGGGAAGATG 6600
BrasilZKV2015      GGAACAGTCTCGCTGGGAATCTTTTTCGTCTTGATGAGGAACAAGGGCATAGGGAAGATG 6600
GD01               GGAACAGTCTCGCTGGGAATCTTCTTCGTCTTGATGAGGAACAAGGGCATAGGGAAGATG 6600
Martinique         GGAACAGTCTCGCTGGGAATCTTTTTCGTCTTGATGAGGAACAAGGGCATAGGGAAGATG 6600
NatalRGN           GGAACAGTCTCGCTGGGAATCTTTTTCGTCTTGATGAGGAACAAGGGCATAGGGAAGATG 6600
HPF2013            GGAACAGTCTCGCTGGGAATCTTTTTCGTCTTGATGAGGAACAAGGGCATAGGGAAGATG 6600
SPH2015            GGAACAGTCTCGCTGGGAATCTTTTTCGTCTTGATGAGGAACAAGGGCATAGGGAAGATG 6600
Haiti2014          GGAACAGTCTCGCTGGGAATCTTTTTCGTCTTGATGAGGAACAAGGGCATAGGGAAGATG 6600
PRVABC59           GGAACAGTCTCGCTGGGAATCTTCTTCGTCTTGATGAGGAACAAGGGCATAGGGAAGATG 6600
BeH819015          GGAACAGTCTCGCTGGGAATCTTCTTCGTCTTGATGAGGAACAAGGGCATAGGGAAGATG 6600
Z1106033           GGAACAGTCTCGCTGGGAATCTTCTTCGTCTTGATGAGGAACAAGGGCATAGGGAAGATG 6600
BEH819966          GGAACAGTCTCGCTGGGAATCTTCTTCGTCTTGATGAGGAACAAGGGCATAGGGAAGATG 6600
SSABr              GGAACAGTCTCGCTGGGAATCTTCTTCGTCTTGATGAGGAACAAGGGCATAGGGAAGATG 6600
Beh815744          GGAACAGTCTCGCTGGGAATCTTCTTCGTCTTGATGAGGAACAAGGGCATAGGGAAGATG 6600
BEH818995          GGAACAGTCTCGCTGGGAATCTTCTTCGTCTTGATGAGGAACAAGGGCATAGGGAAGATG 6600
IbH30656           GGAACAGTTTCGCTAGGGATCTTCTTTGTCTTGATGCGGAACAAGGGCATCGGGAAGATG 6582
ArD128000          GGAACAGTTTCGCTAGGAATCTTCTTTGTCTTGATGCGGAACAAGGGCATCGGGAAGATG 6600
ArD7117            GGAACAGTTTCGCTAGGAATCTTCTTTGTCTTGATGCGGAACAAGGGCATCGGGAAGATG 6600
ARD_41519          GGAACAGTTTCGCTAGGAATCTTCTTTGTCTTGATGCGGAACAAGGGCATCGGGAAGATG 6600
ARD157995          GGAACAGTTTCACTGGGGATCTTCTTCGTCTTGATGCGGAATAAGGGCATCGGGAAGATG 6600
MR_766             GGAACAGTTTCACTGGGGATCTTCTTCGTCTTGATGCGGAATAAGGGCATCGGGAAGATG 6588
ARD158084          GGAACAGTTTCACTGGGGATCTTCTTTGTCTTGATGCGGAATAAGGGCATCGGGAAGATG 6600
ArB1362            GGAACAGTTTCGTTGGGGATATTCTTTGTCTTGATGCGGAACAAGGGCATCGGGAAGATG 6600
ARB13565           GGAACAGTTTCGTTGGGGATCTTCTTTGTCTTGATGCGGAACAAGGGCATCGGGAAGATG 6600
ARB7701            GGAACAGTTTCGTTGGGGATCTTCTTTGTCTTGATGCGGAACAAGGGCATCGGGAAGATG 6600
ARB15076           GGAACAGTTTCGTTGGGGATCTTCTTTGTCTTGATGCGGAACAAGGGCATCGGGAAGATG 6582
                   ******** **  * ** ** ** ** ********* **** ******** *********

P6-740             GGCTTTGGAATGGTGACCCTTGGGGCCAGTGCATGGCTTATGTGGCTCTCGGAAATTGAG 6660
CPC0740            GGCTTTGGAATGGTGACTCTTGGGGCCAGCGCATGGCTTATGTGGCTCTCGGAAATTGAG 6660
YAP2007            GGCTTTGGAATGGTGACTCTTGGGGCCAGCGCATGGCTTATGTGGCTCTCGGAAATTGAG 6660
FSS13025           GGCTTTGGAATGGTGACTCTTGGGGCCAGCGCATGGCTTATGTGGCTCTCGGAAATTGAG 6660
PLCal_ZV           GGCTTTGGAATGGTGACTCTTGGGGCCAGCGCATGGCTCATGTGGCTCTCGGAAATTGAG 6549
SV0127/14          GGCTTTGGAATGGTGACTCTTGGGGCCAGCGCATGGCTCATGTGGCTCTCGGAAATTGAG 6660
8375               GGCTTTGGAATGGTGACCCTTGGGGCCAGTGCATGGCTCATGTGGCTCTCGGAAATTGAG 6660
103344             GGCTTTGGAATGGTGACCCTTGGGGCCAGTGCATGGCTCATGTGGCTCTCGGAAATTGAG 6660
BrasilZKV2015      GGCTTTGGAATGGTGACTCTTGGGGCCAGCGCATGGCTCATGTGGCTCTCGGAAATTGAG 6660
GD01               GGCTTTGGAATGGTGACTCTTGGGGCCAGCGCATGGCTCATGTGGCTCTCGGAAATTGAG 6660
Martinique         GGCTTTGGAATGGTGACTCTTGGGGCCAGCGCATGGCTCATGTGGCTCTCGGAAATTGAG 6660
NatalRGN           GGCTTTGGAATGGTGACTCTTGGGGCCAGCGCATGGCTCATGTGGCTCTCGGAAATTGAG 6660
HPF2013            GGCTTTGGAATGGTGACTCTTGGGGCCAGCGCATGGCTCATGTGGCTCTCGGAAATTGAG 6660
SPH2015            GGCTTTGGAATGGTGACTCTTGGGGCCAGCGCATGGCTCATGTGGCTCTCGGAAATTGAG 6660
Haiti2014          GGCTTTGGAATGGTGACTCTTGGGGCCAGCGCATGGCTCATGTGGCTCTCGGAAATTGAG 6660
PRVABC59           GGCTTTGGAATGGTGACTCTTGGGGCCAGCGCATGGCTCATGTGGCTCTCGGAAATTGAG 6660
BeH819015          GGCTTTGGAATGGTGACTCTTGGGGCCAGCGCATGGCTCATGTGGCTCTCGGAAATTGAG 6660
Z1106033           GGCTTTGGAATGGTGACTCTTGGGGCCAGCGCATGGCTCATGTGGCTCTCGGAAATTGAG 6660
BEH819966          GGCTTTGGAATGGTGACTCTTGGGGCCAGCGCATGGCTCATGTGGCTCTCGGAAATTGAG 6660
SSABr              GGCTTTGGAATGGTGACTCTTGGGGCCAGCGCATGGCTCATGTGGCTCTCGGAAATTGAG 6660
Beh815744          GGCTTTGGAATGGTGACTCTTGGGGCCAGCGCATGGCTCATGTGGCTCTCGGAAATTGAG 6660
```

```
BEH818995          GGCTTTGGAATGGTGACTCTTGGGGCCAGCGCATGGCTCATGTGGCTCTCGGAAATTGAG 6660
IbH30656           GGCTTCGGAATGGTAACCCTTGGGGCCAGCGCATGGCTCATGTGGCTTTCGGAAATTGAA 6642
ArD128000          GGCTTCGGAATGGTAACCCTTGGGGCCAGCGCATGGCTCATGTGGCTCTCGGAAATTGAA 6660
ArD7117            GGCTTCGGAATGGTAACCCTTGGGGCCAGCGCATGGCTCATGTGGCTTTCGGAAATTGAA 6660
ARD_41519          GGCTTCGGAATGGTAACCCTTGGGGCCAGCGCATGGCTCATGTGGCTTTCGGAAATTGAA 6660
ARD157995          GGCTTTGGAATGGTAACCCTTGGGGCCAGTGCATGGCTCATGTGGCTTTCGGAAATTGAA 6660
MR_766             GGCTTTGGAATGGTAACCCTTGGGGCCAGTGCATGGCTCATGTGGCTTTCGGAAATTGAA 6648
ARD158084          GGCTTTGGAATGGTAACCCTTGGGGCCAGTGCATGGCTCATGTGGCTTTCGGAAATTGAA 6660
ArB1362            GGGTTTGGAATGGTAACTCTTGGGGCCAGTGCATGGCTCATGTGGCTTTCGGAAATTGAA 6660
ARB13565           GGTTTTGGAATGGTAACTCTTGGGGCCAGTGCATGGCTCATGTGGCTTTCGGAAATTGAA 6660
ARB7701            GGTTTTGGAATGGTAACTCTTGGGGCCAGTGCATGGCTCATGTGGCTTTCGGAAATTGAA 6660
ARB15076           GGTTTTGGAATGGTAACTCTTGGGGCCAGTGCATGGCTCATGTGGCTTTCGGAAATTGAG 6642
                   ** ** ******** ** *********** ******** ******** ***********

P6-740             CCAGCCAGAATTGCATGTGTCCTCATTGTCGTGTTTCTATTGCTGGTGGTGCTCATACCT 6720
CPC0740            CCAGCCAGAATTGCATGTGTCCTCATTGTCGTGTTCCTATTGCTGGTGGTGCTCATACCT 6720
YAP2007            CCAGCCAGAATTGCATGTGTCCTCATTGTTGTGTTCCTATTGCTGGTGGTGCTCATACCT 6720
FSS13025           CCAGCCAGAATTGCATGTGTCCTCATTGTTGTGTTCCTATTGCTGGTGGTGCTCATACCT 6720
PLCal_ZV           CCAGCCAGAATTGCATGTGTCCTCATTGTTGTGTTCCTACTGCTGGTGGTGCTCATACCT 6609
SV0127/14          CCAGCCAGAATTGCATGCGTCCTCATTGTTGTGTTCCTATTGCTGGTGGTGCTCATACCT 6720
8375               CCAGCCAGAATTGCATGTGTCCTCATTGTTGTGTTCCTATTGCTGGTGGTGCTCATACCT 6720
103344             CCAGCCAGAATTGCATGTGTCCTCATTGTTGTGTTCCTATTGCTGGTGGTGCTCATACCT 6720
BrasilZKV2015      CCAGCCAGAATTGCATGTGTCCTCATTGTTGTGTTCCTATTGCTGGTGGTGCTCATACCT 6720
GD01               CCAGCCAGAATTGCATGTGTCCTCATTGTTGTGTTCCTATTGCTGGTGGTGCTCATACCT 6720
Martinique         CCAGCCAGAATTGCATGTGTCCTCATTGTTGTGTTCCTATTGCTGGTGGTGCTCATACCT 6720
NatalRGN           CCAGCCAGAATTGCATGTGTCCTCATTGTTGTGTTCCTATTGCTGGTGGTGCTCATACCT 6720
HPF2013            CCAGCCAGAATTGCATGTGTCCTCATTGTTGTGTTCCTATTGCTGGTGGTGCTCATACCT 6720
SPH2015            CCAGCCAGAATTGCATGTGTCCTCATTGTTGTGTTCCTATTGCTGGTGGTGCTCATACCT 6720
Haiti2014          CCAGCCAGAATTGCATGTGTCCTCATTGTTGTGTTCCTATTGCTGGTGGTGCTCATACCT 6720
PRVABC59           CCAGCCAGAATTGCATGTGTCCTCATTGTTGTGTTCCTATTGCTGGTGGTGCTCATACCT 6720
BeH819015          CCAGCCAGAATTGCATGTGTCCTCATTGTTGTGTTCCTATTGCTGGTGGTGCTCATACCT 6720
Z1106033           CCAGCCAGAATTGCATGTGTCCTCATTGTTGTGTTCCTATTGCTGGTGGTGCTCATACCT 6720
BEH819966          CCAGCCAGAATTGCATGTGTCCTCATTGTTGTGTTTCTATTGCTGGTGGTGCTCATACCT 6720
SSABr              CCAGCCAGAATTGCATGTGTCCTCATTGTTGTGTTTCTATTGCTGGTGGTGCTCATACCT 6720
Beh815744          CCAGCCAGAATTGCATGTGTCCTCATTGTTGTGTTTCTATTGCTGGTGGTGCTCATACCT 6720
BEH818995          CCAGCCAGAATTGCATGTGTCCTCATTGTTGTGTTTCTATTGCTGGTGGTGCTCATACCT 6720
IbH30656           CCAGCCAGAATCGCATGTGTCCTCATTGTCGTGTTTCTGTTACTGGTGGTGCTCATACCT 6702
ArD128000          CCAGCCAGAATCGCATGTGTCCTCATTGTCGTGTTTTTGTTACTGGTGGTGCTCATACCC 6720
ArD7117            CCAGCCAGAATCGCATGTGTCCTCATTGTCGTGTTTTTGTTACTGGTGGTGCTCATACCC 6720
ARD_41519          CCAGCCAGAATCGCATGTGTCCTCATTGTCGTGTTTTTGTTGCTGGTGGTGCTCATACCC 6720
ARD157995          CCAGCCAGAATTGCATGTGTCCTCATTGTTGTGTTTTTATTACTGGTGGTGCTCATACCC 6720
MR_766             CCAGCCAGAATTGCATGTGTCCTCATTGTTGTGTTTTTATTACTGGTGGTGCTCATACCC 6708
ARD158084          CCAGCCAGAATTGCATGTGTCCTCATTGTTGTGTTTTTATTACTGGTGGTGCTCATACCC 6720
ArB1362            CCAGCCAGAATTGCATGTGTCCTCATTGTTGTGTTTTTGTTACTGGTGGTACTCATACCT 6720
ARB13565           CCAGCCAGAATTGCATGTGTCCTCATTGTTGTGTTCTTGTTACTGGTGGTACTCATACCC 6720
ARB7701            CCAGCCAGAATTGCATGTGTCCTCATTGTTGTGTTCTTGTTACTGGTGGTACTCATACCC 6720
ARB15076           CCAGCCAGAATTGCATGTGTCCTCATTGTTGTGTTCTTGTTACTGGTGGTACTCATACCC 6702
                   *********** ***** *********** *****  *  * ******** ********

P6-740             GAGCCAGAAAAGCAGAGATCTCCCCAGGACAACCAAATGGCAATTATCATCATGGTAGCA 6780
CPC0740            GAGCCAGAAAAGCAAAGATCTCCTCAGGACAACCAAATGGCAATCATCATCATGGTAGCA 6780
YAP2007            GAGCCAGAAAAGCAAAGATCTCCCCAGGACAACCAAATGGCAATCATCATCATGATAGCA 6780
FSS13025           GAGCCAGAAAAGCAAAGATCTCCCCAGGACAACCAAATGGCAATCATCATCATGGTAGCA 6780
PLCal_ZV           GAGCCAGAGAAGCAAAGATCTCCTCAGGACAACCAAATGGCAATCATCATCATGGTAGCA 6669
SV0127/14          GAGCCAGAAAAGCAAAGATCCCCCCAGGACAACCAAATGGCAATCATCATCATGGTAGCA 6780
8375               GAGCCAGAAAAGCAAAGATCTCCCCAGGACAACCAAATGGCAATCATCATCATGGTAGCA 6780
103344             GAGCCAGAAAAGCAAAGATCTCCCCAGGACAACCAAATGGCAATCATCATCATGGTAGCA 6780
BrasilZKV2015      GAGCCAGAAAAGCAAAGATCTCCCCAGGACAACCAAATGGCAATCATCATCATGGTAGCA 6780
GD01               GAGCCAGAAAAGCAAAGATCTCCCCAGGACAACCAAATGGCAATCATCATCATGGTAGCA 6780
Martinique         GAGCCAGAAAAGCAAAGATCTCCCCAGGACAACCAAATGGCAATCATCATCATGGTAGCA 6780
NatalRGN           GAGCCAGAAAAGCAAAGATCTCCCCAGGACAACCAAATGGCAATCATCATCATGGTAGCA 6780
HPF2013            GAGCCAGAAAAGCAAAGATCTCCCCAGGACAACCAAATGGCAATCATCATCATGGTAGCA 6780
SPH2015            GAGCCAGAAAAGCAAAGATCTCCCCAGGACAACCAAATGGCAATCATCATCATGGTAGCA 6780
Haiti2014          GAGCCAGAAAAGCAAAGATCTCCCCAGGACAACCAAATGGCAATCATCATCATGGTAGCA 6780
PRVABC59           GAGCCAGAAAAGCAAAGATCTCCCCAGGACAACCAAATGGCAATCATCATCATGGTAGCA 6780
BeH819015          GAGCCAGAAAAGCAAAGATCTCCCCAGGACAACCAAATGGCAATCATCATCATGGTAGCA 6780
Z1106033           GAGCCAGAAAAGCAAAGATCTCCCCAGGACAACCAAATGGCAATCATCATCATGGTAGCA 6780
BEH819966          GAGCCAGAAAAGCAAAGATCTCCCCAGGACAACCAAATGGCAATCATCATCATGGTAGCA 6780
```

```
SSABr           GAGCCAGAAAAGCAAAGATCTCCCCAGGACAACCAAATGGCAATCATCATCATGGTAGCA 6780
Beh815744       GAGCCAGAAAAGCAAAGATCTCCCCAGGACAACCAAATGGCAATCATCATCATGGTAGCA 6780
BEH818995       GAGCCAGAAAAGCAAAGATCTCCCCAGGACAACCAAATGGCAATCATCATCATGGTAGCA 6780
IbH30656        GAGCCAGAGAAGCAAAGATCTCCCCAGGACAATCAAATGGCAATCATCATCATGGTGGCA 6762
ArD128000       GAGCCAGAGAAGCAAAGATCTCCCCAGGATAATCAAATGGCAATCATCATCATGGTGGCA 6780
ArD7117         GAGCCAGAGAAGCAAAGATCTCCCCAGGATAATCAAATGGCAATCATCATCATGGTGGCA 6780
ARD_41519       GAGCCAGAGAAGCAAAGATCTCCCCAGGATAATCAAATGGCAATCATCATCATGGTGGCA 6780
ARD157995       GAGCCAGAGAAGCAAAGATCTCCCCAAGATAACCAGATGGCAATTATCATCATGGTGGCA 6780
MR_766          GAGCCAGAGAAGCAAAGATCTCCCCAAGATAACCAGATGGCAATTATCATCATGGTGGCA 6768
ARD158084       GAGCCAGAGAAGCAAAGATCTCCCCAAGATAACCAGATGGCAATTATCATCATGGTGGCA 6780
ArB1362         GAGCCAGAGAAGCAAAGATCTCCCCAGGATAACCAGATGGCAATCATCATCATGGTGGCA 6780
ARB13565        GAGCCAGAGAAGCAAAGATCTCCCCAGGACAACCAGATGGCAATCATCATCATGGTGGCA 6780
ARB7701         GAGCCAGAGAAGCAAAGATCTCCCCAGGACAACCAGATGGCAATCATCATCATGGTGGCA 6780
ARB15076        GAGCCAGAGAAGCAAAGATCTCCCCAGGACAACCAGATGGCAATCATCATCATGGTGGCA 6762
                ******** ***** ***** ** ** ** ** ** ******** ********* * ***

P6-740          GTGGGTCTTCTGGGCTTGATAACCGCCAATGAACTCGGATGGTTGGAGAGAACAAAAAGT 6840
CPC0740         GTGGGTCTTCTGGGCTTGATTACCGCCAATGAACTCGGATGGTTGGAGAGAACAAAAAGT 6840
YAP2007         GTGGGTCTTCTGGGTTTGATTACCGCCAATGAACTTGGATGGTTGGAAAGAACAAAGAGT 6840
FSS13025        GTGGGTCTTCTGGGCTTGATTACCGCCAATGAACTCGGATGGTTGGAGAGAACAAAGAGT 6840
PLCal_ZV        GTGGGTCTTCTGGGCTTGATTACTGCCAATGAACTCGGATGGTTGGAAAGAACAAAGAGT 6729
SV0127/14       GTAGGTCTTCTGGGCTTGATTACCGCCAATGAACTCGGATGGTTGGAGAGAACAAAGAGT 6840
8375            GTAGGTCTTCTGGGCTTGATTACCGCCAATGAACTCGGATGGTTGGAGAGAACAAAGAGT 6840
103344          GTAGGTCTTCTGGGCTTGATTACCGCCAATGAACTCGGATGGTTGGAGAGAACAAAGAGT 6840
BrasilZKV2015   GTAGGTCTTCTGGGCTTGATTACCGCCAATGAACTCGGATGGTTGGAGAGAACAAAGAGT 6840
GD01            GTAGGTCTTCTGGGCTTGATTACCGCCAATGAACTCGGATGGTTGGAGAGAACAAAGAGT 6840
Martinique      GTAGGTCTTCTGGGCTTGATTACCGCCAATGAACTCGGATGGTTGGAGAGAACAAAGAGT 6840
NatalRGN        GTAGGTCTTCTGGGCTTGATTACCGCCAATGAACTCGGATGGTTGGAGAGAACAAAGAGT 6840
HPF2013         GTAGGTCTTCTGGGCTTGATTACCGCCAATGAACTCGGATGGTTGGAGAGAACAAAGAGT 6840
SPH2015         GTAGGTCTTCTGGGCTTGATTACCGCCAATGAACTCGGATGGTTGGAGAGAACAAAGAGT 6840
Haiti2014       GTAGGTCTTCTGGGCTTGATTACCGCCAATGAACTCGGATGGTTGGAGAGAACAAAGAGT 6840
PRVABC59        GTAGGTCTTCTGGGCTTGATTACCGCCAATGAACTCGGATGGTTGGAGAGAACAAAGAGT 6840
BeH819015       GTAGGTCTTCTGGGCTTGATTACCGCCAATGAACTCGGATGGTTGGAGAGAACAAAGAGT 6840
Z1106033        GTAGGTCTTCTGGGCTTGATTACCGCCAATGAACTCGGATGGTTGGAGAGAACAAAGAGT 6840
BEH819966       GTAGGTCTTCTGGGCTTGATTACCGCCAATGAACTCGGATGGTTGGAGAGAACAAAGAGT 6840
SSABr           GTAGGTCTTCTGGGCTTGATTACCGCCAATGAACTCGGATGGTTGGAGAGAACAAAGAGT 6840
Beh815744       GTAGGTCTTCTGGGCTTGATTACCGCCAATGAACTCGGATGGTTGGAGAGAACAAAGAGT 6840
BEH818995       GTAGGTCTTCTGGGCTTGATTACCGCCAATGAACTCGGATGGTTGGAGAGAACAAAGAGT 6840
IbH30656        GTGGGCCTTCTGGGTTTGATAACTGCAAACGAACTCGGATGGCTGGAAAGAACAAAAAGT 6822
ArD128000       GTTGGCCTTTTGGGTTTGATAACTGCAAATGAACTCGGATGGCTGGAAAGGACAAAAAGT 6840
ArD7117         GTTGGCCTTCTGGGTTTGATAACAGCAAATGAACTCGGATGGCTGGAAAGGACAAAAAGT 6840
ARD_41519       GTTGGCCTTCTGGGTTTGATAACTGCAAATGAACTCGGATGGCTGGAAAGGACAAAAAGT 6840
ARD157995       GTGGGCCTTCTAGGTTTGATAACTGCAAACGAACTTGGATGGCTGGAAAGAACAAAAAAT 6840
MR_766          GTGGGCCTTCTAGGTTTGATAACTGCAAACGAACTTGGATGGCTGGAAAGAACAAAAAAT 6828
ARD158084       GTGGGCCTTCTAGGTTTGATAACTGCAAACGAACTTGGATGGCTGGAAAGAACAAAAAAT 6840
ArB1362         GTGGGCCTTCTGGGGTTGATAACTGCAAATGAACTTGGATGGCTGGAAAGAACAAAAAAT 6840
ARB13565        GTGGGCCTTCTGGGTTTGATAACTGCAAACGAACTTGGATGGCTGGAAAGAACAAAAAAT 6840
ARB7701         GTGGGCCTTCTGGGTTTGATAACTGCAAACGAACTTGGATGGCTGGAAAGAACAAAAAAT 6840
ARB15076        GTGGGCCTTCTGGGTTTGATAACTGCAAACGAACTTGGATGGCTGGAAAGAACAAAAAAT 6822
                ** ** *** * ** ***** ** ** ** ***** ****** **** ** ***** * *

P6-740          GACCTAGGCCATCTAATGGGAAGGAGAGAGGAGGGGGCAACCATGGGATTCTCAATGGAC 6900
CPC0740         GACCTAAGCCATCTAATGGGAAGGAGAGAGGAGGGGGCAACCACAGGATTCTCAATGGAC 6900
YAP2007         GACCTAAGCCATCTAATGGGAAGGAGAGAGGAGGGGGCAACCATAGGATTCTCAATGGAC 6900
FSS13025        GACCTAAGCCATCTAATGGGAAGGAGAGAGGAGGGGGCAACNATAGGATTCTCAATGGAC 6900
PLCal_ZV        GACCTAAGCCATCTAATGGGAAGGAGAGAGGAGGGGGCAACCATAGGATTCTCAATGGAC 6789
SV0127/14       GACCTAAGCCATCTAATGGGAAGGAGAGAGGAGGGGGCAACCATAGGATTCTCAATGGAC 6900
8375            GACCTAAGCCATCTAATGGGAAGGAGAGAGGAGGGAGCAACCATAGGATTCTCAATGGAC 6900
103344          GACCTAAGCCATCTAATGGGAAGGAGAGAGGAGGGAGCAACCATAGGATTCTCAATGGAC 6900
BrasilZKV2015   GACCTAAGCCATCTAATGGGAAGGAGAGAGGAGGGGGCAACCATAGGATTCTCAATGGAC 6900
GD01            GACCTAAGCCATCTAATGGGAAGGAGAGAGGAGGGGGCAACCATAGGATTCTCAATGGAC 6900
Martinique      GACCTAAGCCATCTAATGGGAAGGAGAGAGGAGGGGGCAACCATAGGATTCTCAATGGAC 6900
NatalRGN        GACCTAAGCCATCTAATGGGAAGGAGAGAGGAGGGAGCAACCATAGGATTCTCAATGGAC 6900
HPF2013         GACCTAAGCCATCTAATGGGAAGGAGAGAGGAGGGGGCAACCATAGGATTCTCAATGGAC 6900
SPH2015         GACCTAAGCCATCTAATGGGAAGGAGAGAGGAGGGGGCAACCATGGGATTCTCAATGGAC 6900
Haiti2014       GACCTAAGCCATCTAATGGGAAGGAGAGAGGAGGGGGCAACCATGGGATTCTCAATGGAC 6900
PRVABC59        GACCTAAGCCATCTAATGGGAAGGAGAGAGGAGGGGGCAACCATAGGATTCTCAATGGAC 6900
BeH819015       GACCTAAGCCATCTAATGGGAAGGAGAGAGGAGGGGGCAACCATAGGATTCTCAATGGAC 6900
```

```
Z1106033           GACCTAAGCCATCTAATGGGAAGGAGAGAGGAGGGGGCAACCATAGGATTCTCAATGGAC 6900
BEH819966          GACCTAAGCCATCTAATGGGAAGGAGAGAGGAGGGGGCAACCATAGGATTCTCAATGGAC 6900
SSABr              GACCTAAGCCATCTAATGGGAAGGAGAGAGGAGGGGGCAACCATAGGATTCTCAATGGAC 6900
Beh815744          GACCTAAGCCATCTAATGGGAAGGAGAGAGGAGGGGGCAACCATAGGATTCTCAATGGAC 6900
BEH818995          GACCTAAGCCATCTAATGGGAAGGAGAGAGGAGGGGGCAACCATAGGATTCTCAATGGAC 6900
IbH30656           GATATAGCTCATCTAATGGGAAGGAAAGAAGAGGGGACAACCGTAGGATTCTCAATGGAT 6882
ArD128000          GATATAGCTTATCTGATGGGAAGGAAAGAAGAGGGGACAACCATAGGATTCTCAATGGAT 6900
ArD7117            GATATAGCTCATCTAATGGGAAGGAAAGAAGAGGGGACAACCATAGGATTCTCAATGGAT 6900
ARD_41519          GATATAGCTCATCTAATGGGAAGGAAAGAAGAGGGGACAACCATGGGATTCTCAATGGAT 6900
ARD157995          GACATAGCTCATCTAATGGGAAGGAGAGAAGAAGGAGCAACCATGGGATTCTCAATGGAC 6900
MR_766             GACATAGCTCATCTAATGGGAAGGAGAGAAGAAGGAGCAACCATGGGATTCTCAATGGAC 6888
ARD158084          GACATAGCTCATCTAATGGGAAGGAGAGAAGAAGGAGCAACCATGGGATTCTCAATGGAC 6900
ArB1362            GACATAGCTCATCTAATGGGAAGGAGAGAAGAAGGAGTAACTATGGGATTCTCAATGGAC 6900
ARB13565           GACATAGCTCATCTAATGGGAAGGAGAGAAGAAGGAGTAACTATGGGATTCTCAATGGAC 6900
ARB7701            GACATAGCTCATCTAATGGGAAGGAGAGAAGAAGGAGTAACTATGGGATTCTCAATGGAC 6900
ARB15076           GACATAGCTCATCTAATGGGAAGGAGAGAAGAAGGAGTAACTATGGGATTCTCAATGGAC 6882
                   **  **    **** ********** *** ** **   ***    **************

P6-740             ATTGACTTGCGGCCAGCCTCAGCTTGGGCTATCTATGCCGCTCTGACAACTCTCATCACC 6960
CPC0740            ATTGACCTGCGGCCAGCCTCAGCTTGGGCTATCTATGCTGCTCTGACAACTTTCATCACC 6960
YAP2007            ATTGACCTGCGGCCAGCCTCAGCTTGGGCTATCTATGCTGCTCTGACAACTTTCATCACC 6960
FSS13025           ATTGACCTGCGGCCAGCCTCAGCTTGGGCTATCTATGCTGCTCTGACAACTTTCATTACC 6960
PLCal_ZV           ATTGACCTGCGGCCAGCCTCAGCTTGGGCTATCTACGCTGCTTTGACAACTTTCATTACC 6849
SV0127/14          ATTGACCTGCGGCCAGCCTCGGCCTGGGCCATCTATGCTGCCCTGACAACTTTCATTACC 6960
8375               ATTGACCTGCGGCCAGCCTCAGCTTGGGCCATCTATGCTGCCTTGACAACTTTCATTACC 6960
103344             ATTGACCTGCGGCCAGCCTCAGCTTGGGCCATCTATGCTGCCTTGACAACTTTCATTACC 6960
BrasilZKV2015      ATTGACCTGCGGCCAGCCTCAGCTTGGGCCATCTATGCTGCCTTGACAACTTTCATTACC 6960
GD01               ATTGACCTGCGGCCAGCCTCAGCTTGGGCCATCTATGCTGCCTTGACAACTTTCATTACC 6960
Martinique         ATTGACCTGCGGCCAGCCTCAGCTTGGGCCATCTATGCTGCCTTGACAACTTTCATTACC 6960
NatalRGN           ATTGACCTGCGGCCAGCCTCAGCTTGGGCCATCTATGCTGCCTTGACAACTTTCATTACC 6960
HPF2013            ATTGACCTGCGGCCAGCCTCAGCTTGGGCCATCTATGCTGCCTTGACAACTTTCATTACC 6960
SPH2015            ATTGACCTGCGGCCAGCCTCAGCTTGGGCCATCTATGCTGCCTTGACAACTTTCATTACC 6960
Haiti2014          ATTGACCTGCGGCCAGCCTCAGCTTGGGCCATCTATGCTGCCTTGACAACTTTCATTACC 6960
PRVABC59           ATTGACCTGCGGCCAGCCTCAGCTTGGGCCATCTATGCTGCCTTGACAACTTTCATTACC 6960
BeH819015          ATTGACCTGCGGCCAGCCTCAGCTTGGGCCATCTATGCTGCCTTGACAACTTTCATTACC 6960
Z1106033           ATTGACCTGCGGCCAGCCTCAGCTTGGGCCATCTATGCTGCCTTGACAACTTTCATTACC 6960
BEH819966          ATTGACCTGCGGCCAGCCTCAGCTTGGGCCATCTATGCTGCCTTGACAACTTTCATTACC 6960
SSABr              ATTGACCTGCGGCCAGCCTCAGCTTGGGCCATCTATGCTGCCTTGACAACTTTCATTACC 6960
Beh815744          ATTGACCTGCGGCCAGCCTCAGCTTGGGCCATCTATGCTGCCTTGACAACTTTCATTACC 6960
BEH818995          ATTGACCTGCGGCCAGCCTCAGCTTGGGCCATCTATGCTGCCTTGACAACTTTCATTACC 6960
IbH30656           ATTGATCTGCGGCCAGCCTCCGCCTGGGCTATTTATGCCGCATTGACAACTCTCATCACC 6942
ArD128000          ATTGATCTGCGGCCAGCCTCTGCCTGGGCTATTTATGCCGCATTGACAACTCTCATCACC 6960
ArD7117            ATTGATCTGCGGCCAGCCTCCGCCTGGGCTATTTATGCCGCATTGACAACTCTCATCACC 6960
ARD_41519          ATTGATCTGCGGCCAGCCTCCGCCTGGGCTATTTATGCCGCATTGACAACTCTCATCACC 6960
ARD157995          ATTGATCTGCGGCCAGCCTCCGCCTGGGCTATCTATGCCGCATTGACAACTCTCATCACC 6960
MR_766             ATTGATCTGCGGCCAGCCTCCGCCTGGGCTATCTATGCCGCATTGACAACTCTCATCACC 6948
ARD158084          ATTGATCTGCGGCCAGCCTCCGCCTGGGCTATCTATGCCGCATTGACAACTCTCATCACC 6960
ArB1362            ATCGATTTGAGGCCGGCCTCCGCCTGGGCTATCTATGCTGCATTGACAACTCTCATCACC 6960
ARB13565           ATCGATTTGCGGCCAGCCTCCGCCTGGGCTATCTATGCCGCACTGACAACTCTCATCACC 6960
ARB7701            ATCGATTTGCGGCCAGCCTCCGCCTGGGCTATCTATGCCGCACTGACAACTCTCATCACC 6960
ARB15076           ATCGATTTGCGGCCAGCCTCCGCCTGGGCTATTTATGCCGCACTGACAACTCTCATCACC 6942
                   ** **  ** **** ***** ** ***** ** ** ** **  ******** **** ***

P6-740             CCAGCCGTCCAACATGCGGTAACCACTTCATACAACAACTACTCCTTAATGGCGATGGCC 7020
CPC0740            CCAGCCGTCCAACATGCGGTGACCACTTCATACAACAACTACTCCTTAATGGCGATGGCC 7020
YAP2007            CCAGCCGTCCAACATGCGGTGACCACTTCATACAACAACTACTCCTTAATGGCGATGGCC 7020
FSS13025           CCAGCCGTCCAACATGCAGTGACCACTTCATACAACAACTACTCCTTAATGGCGATGGCC 7020
PLCal_ZV           CCAGCCGTCCAGCATGCAGTGACCACTTCATACAACAACTACTCCTTAATGGCGATGGCC 6909
SV0127/14          CCAGCCGTCCAACATGCAGTGACCACTTCATACAACAACTACTCCTTAATGGCGATGGCC 7020
8375               CCAGCCGTCCAACATGCAGTGACCACTTCATACAACAACTACTCCTTAATGGCGATGGCC 7020
103344             CCAGCCGTCCAACATGCAGTGACCACTTCATACAACAACTACTCCTTAATGGCGATGGCC 7020
BrasilZKV2015      CCAGCCGTCCAACATGCAGTGACCACTTCATACAACAACTACTCCTTAATGGCGATGGCC 7020
GD01               CCAGCCGTCCAACATGCAGTGACCACCTCATACAACAACTACTCCTTAATGGCGATGGCC 7020
Martinique         CCAGCCGTCCAACATGCAGTGACCACTTCATACAACAACTACTCCTTAATGGCGATGGCC 7020
NatalRGN           CCAGCCGTCCAACATGCAGTGACCACTTCATACAACAACTACTCCTTAATGGCGATGGCC 7020
HPF2013            CCAGCCGTCCAACATGCAGTGACCACTTCATACAACAACTACTCCTTAATGGCGATGGCC 7020
SPH2015            CCAGCCGTCCAACATGCAGTGACCACTTCATACAACAACTACTCCTTAATGGCGATGGCC 7020
Haiti2014          CCAGCCGTCCAACATGCAGTGACCACTTCATACAACAACTACTCCTTAATGGCGATGGCC 7020
```

```
PRVABC59          CCAGCCGTCCAACATGCAGTGACCACCTCATACAACAACTACTCCTTAATGGCGATGGCC 7020
BeH819015         CCAGCCGTCCAACATGCAGTGACCACCTCATACAACAACTACTCCTTAATGGCGATGGCC 7020
Z1106033          CCAGCCGTCCAACATGCAGTGACCACCTCATACAACAACTACTCCTTAATGGCGATGGCC 7020
BEH819966         CCAGCCGTCCAACATGCAGTGACCACTTCATACAACAACTACTCCTTAATGGCGATGGCC 7020
SSABr             CCAGCCGTCCAACATGCAGTGACCACTTCATACAACAACTACTCCTTAATGGCGATGGCC 7020
Beh815744         CCAGCCGTCCAACATGCAGTGACCACTTCATACAACAACTACTCCTTAATGGCGATGGCC 7020
BEH818995         CCAGCCGTCCAACATGCAGTGACCACTTCATACAACAACTACTCCTTAATGGCGATGGCC 7020
IbH30656          CCAGCCGTCCAACATGCGGTGACCACCTCATACAACAACTACTCCCTGATGGCGATGGCC 7002
ArD128000         CCAGCCGTCCAACATGCGGTAACCACCTCATACAACAACTACTCCCTGATGGCGATGGCC 7020
ArD7117           CCAGCCGTCCAACATGCGGTAACCACCTCATACAACAACTACTCCCTGATGGCGATGGCC 7020
ARD_41519         CCAGCCGTCCAACATGCGGTAACCACCTCATACAACAACTACTCCCTGATGGCGATGGCC 7020
ARD157995         CCAGCTGTCCAACATGCGGTAACCACTTCATACAACAACTACTCCTTAATGGCGATGGCC 7020
MR_766            CCAGCTGTCCAACATGCGGTAACCACTTCATACAACAACTACTCCTTAATGGCGATGGCC 7008
ARD158084         CCAGCTGTCCAACATGCGGTAACCACTTCATACAACAACTACTCCTTAATGGCGATGGCC 7020
ArB1362           CCAGCCGTCCAACATGCGGTAACCACTTCATATAACAACTACTCCTTAATGGCGATGGCC 7020
ARB13565          CCAGCCGTCCAACACGCGGTAACCACTTCATACAACAACTACTCCCTAATGGCGATGGCC 7020
ARB7701           CCAGCCGTCCAACACGCGGTAACCACTTCATACAACAACTACTCCCTAATGGCGATGGCC 7020
ARB15076          CCAGCTGTCCAACACGCGGTAACCACTTCATACAACAACTACTCCCTAATGGCGATGGCC 7002
                  ***** ***** ** ** ** ***** ***** ************ * ************

P6-740            ACGCAAGCCGGAGTGTTGTTTGGCATGGGCAAAGGGATGCCATTCTATGCGTGGGACTTC 7080
CPC0740           ACGCAAGCTGGGGTGTTGTTTGGTATGGGCAAAGGGATGCCATTCTACGCATGGGACTTT 7080
YAP2007           ACGCAAGCTGGAGTGTTGTTTGGTATGGGTAAAGGGATGCCATTCTACGCATGGGACTTT 7080
FSS13025          ACGCAAGCTGGAGTGTTGTTCGGTATGGGTAAAGGGATGCCATTCTATGCATGGGACTTT 7080
PLCal_ZV          ACGCAAGCCGGAGTGTTGTTTGGTATGGGCAAAGGGATGCCATTCTACGCATGGGACTTT 6969
SV0127/14         ACGCAAGCTGGAGTGTTGTTTGGTATGGGCAAAGGGATGCCATTCTACGCATGGGACTTT 7080
8375              ACGCAAGCTGGAGTGTTGTTTGGTATGGGCAAAGGGATGCCATTCTACGCATGGGACTTT 7080
103344            ACGCAAGCTGGAGTGTTGTTTGGTATGGGCAAAGGGATGCCATTCTACGCATGGGACTTT 7080
BrasilZKV2015     ACGCAAGCTGGAGTGTTGTTTGGTATGGGCAAAGGGATGCCATTCTACGCATGGGACTTT 7080
GD01              ACGCAAGCTGGAGTGTTGTTTGGTATGGGCAAAGGGATGCCATTCTACGCATGGGACTTT 7080
Martinique        ACGCAAGCTGGAGTGTTGTTTGGTATGGGCAAAGGGATGCCATTCTACGCATGGGACTTT 7080
NatalRGN          ACGCAAGCTGGAGTGTTGTTTGGTATGGGCAAAGGGATGCCATTCTACGCATGGGACTTT 7080
HPF2013           ACGCAAGCTGGAGTGTTGTTTGGTATGGGCAAAGGGATGCCATTCTACGCATGGGACTTT 7080
SPH2015           ACGCAAGCTGGAGTGTTGTTTGGTATGGGCAAAGGGATGCCATTCTACGCATGGGACTTT 7080
Haiti2014         ACGCAAGCTGGAGTGTTGTTTGGTATGGGCAAAGGGATGCCATTCTACGCATGGGACTTT 7080
PRVABC59          ACGCAAGCTGGAGTGTTGTTTGGCATGGGCAAAGGGATGCCATTCTACGCATGGGACTTT 7080
BeH819015         ACGCAAGCTGGAGTGTTGTTTGGTATGGGCAAAGGGATGCCATTCTACGCATGGGACTTT 7080
Z1106033          ACGCAAGCTGGAGTGTTGTTTGGTATGGGCAAAGGGATGCCATTCTACGCATGGGACTTT 7080
BEH819966         ACGCAAGCTGGAGTGTTGTTTGGTATGGGCAAAGGGATGCCATTCTACGCATGGGACTTT 7080
SSABr             ACGCAAGCTGGAGTGTTGTTTGGTATGGGCAAAGGGATGCCATTCTACGCATGGGACTTT 7080
Beh815744         ACGCAAGCTGGAGTGTTGTTTGGTATGGGCAAAGGGATGCCATTCTACGCATGGGACTTT 7080
BEH818995         ACGCAAGCTGGAGTGTTGTTTGGTATGGGCAAAGGGATGCCATTCTACGCATGGGACTTT 7080
IbH30656          ACACAAGCTGGAGTGCTGTTTGGCATGGGCAAAGGGATGCCATTTTATGCATGGGACTTT 7062
ArD128000         ACACAAGCTGGAGTGCTGTTTGGCATGGGCAAAGGGATGCCATTTTATGCATGGGACTTT 7080
ArD7117           ACACAAGCTGGAGTGCTGTTTGGCATGGGCAAAGGGATGCCATTTTATGCATGGGACTTT 7080
ARD_41519         ACACAAGCTGGAGTGCTGTTTGGCATGGGCAAAGGGATGCCATTTTATGCATGGGACTTT 7080
ARD157995         ACACAAGCTGGAGTGCTGTTTGGCATGGGCAAAGGGATGCCATTTATGCATGGGGACCTT 7080
MR_766            ACACAAGCTGGAGTGCTGTTTGGCATGGGCAAAGGGATGCCATTTATGCATGGGGACCTT 7068
ARD158084         ACACAAGCTGGAGTGCTGTTTGGCATGGGCAAAGGGATGCCATTTTATGCATGGGACTTT 7080
ArB1362           ACACAAGCTGGAGTGCTGTTTGGCATGGGCAAAGGGATGCCATTTTATGCATGGGACTTT 7080
ARB13565          ACACAAGCTGGAGTGCTATTTGGCATGGGCAAAGGGATGCCATTTTATGCATGGGACTTT 7080
ARB7701           ACACAAGCTGGAGTGCTATTTGGCATGGGCAAAGGGATGCCATTTTATGCATGGGACTTT 7080
ARB15076          ACACAAGCTGGAGTGCTATTTGGCATGGGCAAAGGGATGCCATTTTATGCATGGGACTTT 7062
                  ** ***** ** *** * ** ** ***** **************        ***** *

P6-740            GGAGTCCCGCTGCTAATGATGGGTTGCTACTCACAATTAACACCCTTGACCTTAATAGTG 7140
CPC0740           GGAGTCCCGCTGCTAATGATGGGTTGCTACTCACAATTAACACCTCTGACCCTAATAGTG 7140
YAP2007           GGAGTCCCGCTGCTAATGATGGGTTGCTACTCACAATTAACACCCCTGACCCTAATAGTG 7140
FSS13025          GGAGTCCCGCTGCTAATGATAGGTTGCTACTCACAATTAACACCCCTGACCCTAATAGTG 7140
PLCal_ZV          GGAGTCCCGCTGCTAATGATAGGTTGCTACTCACAATTAACACCCCTGACCCTAATAGTG 7029
SV0127/14         GGAGTCCCGCTGCTAATGATAGGTTGCTACTCACAATTAACACCCCTGACCCTAATAGTG 7140
8375              GGAGTCCCGCTGCTAATGATAGGTTGCTACTCACAATTAACACCCCTGACCCTAATAGTG 7140
103344            GGAGTCCCGCTGCTAATGATAGGTTGCTACTCACAATTAACACCCCTGACCCTAATAGTG 7140
BrasilZKV2015     GGAGTCCCGCTGCTAATGATAGGTTGCTACTCACAATTAACACCCCTGACCCTAATAGTG 7140
GD01              GGAGTCCCGCTGCTAATGATAGGCTGCTACTCACAATTAACACCCCTGACCCTAATAGTG 7140
Martinique        GGAGTCCCACTGCTAATGATAGGTTGCTACTCACAATTAACACCCCTGACCCTAATAGTG 7140
NatalRGN          GGAGTCCCGCTGCTAATGATAGGTTGCTACTCACAATTAACACCCCTGACCCTAATAGTG 7140
HPF2013           GGAGTCCCGCTGCTAATGATAGGTTGCTACTCACAATTAACACCCCTGACCCTAATAGTG 7140
```

```
SPH2015            GGAGTCCCGCTGCTAATGATAGGTTGCTACTCACAATTAACGCCCCTGACCCTAATAGTG 7140
Haiti2014          GGAGTCCCGCTGCTAATGATAGGTTGCTACTCACAATTAACGCCCCTGACCCTAATAGTG 7140
PRVABC59           GGAGTCCCGCTGCTAATGATAGGTTGCTACTCACAATTAACACCCCTGACCCTAATAGTG 7140
BeH819015          GGAGTCCCGCTGCTAATGATAGGTTGCTACTCACAATTAACACCCCTGACCCTAATAGTG 7140
Z1106033           GGAGTCCCGCTGCTAATGATAGGTTGCTACTCACAATTAACACCCCTGACCCTAATAGTG 7140
BEH819966          GGAGTCCCGCTGCTAATGATAGGTTGCTACTCACAATTAACACCCCTGACCCTAATAGTG 7140
SSABr              GGAGTCCCGCTGCTAATGATAGGTTGCTACTCACAATTAACACCCCTGACCCTAATAGTG 7140
Beh815744          GGAGTCCCGCTGCTAATGATAGGTTGCTACTCACAATTAACACCCCTGACCCTAATAGTG 7140
BEH818995          GGAGTCCCGCTGCTAATGATAGGTTGCTACTCACAATTAACACCCCTGACCCTAATAGTG 7140
IbH30656           GGAGTCCCGCTGCTAATGATGGGTTGTTACTCACAATTAACACCCCTGACCCTGATAGTG 7122
ArD128000          GGAGTCCCGCTGCTAATGATAGGTTGCTACTCACAGTTAACACCCCTGACCCTGATAGTA 7140
ArD7117            GGAGTCCCGCTGCTAATGATGGGTTGCTACTCACAATTAACACCCCTGACCCTGATAGTG 7140
ARD_41519          GGAGTCCCGCTGCTAATGATGGGTTGCTACTCACAATTAACACCCCTGACCCTGATAGTG 7140
ARD157995          GGAGTCCCGCTGCTAATGATGGGTTGCTATTCACAATTAACACCCCTGACTCTGATAGTA 7140
MR_766             GGAGTCCCGCTGCTAATGATGGGTTGCTATTCACAATTAACACCCCTGACTCTGATAGTA 7128
ARD158084          GGAGTCCCGCTGCTAATGATGGGTTGCTATTCACAATTAACACCCCTGACTCTGATAGTA 7140
ArB1362            GGAGTCCCGCTGCTAATGATGGGTTGCTACTCACAATTAACACCCCTGACCCTGATAGTG 7140
ARB13565           GGAGTCCCGCTGCTAATGATGGGTTGCTACTCACAATTAACACCCCTAACCTTGATAGTG 7140
ARB7701            GGAGTCCCGCTGCTAATGATGGGTTGCTACTCACAATTAACACCCCTAACCTTGATAGTG 7140
ARB15076           GGAGTCCCGCTGCTAATGGTGGGTTGCTACTCACAATTAACACCCCTAACCTTGATAGTG 7122
                   ******** ********* * ** ** ** ***** ***** **  * **  * *****

P6-740             GCCATCATTCTGCTCGTGGCGCACTACATGTACTTGATCCCAGGTCTACAGGCAGCAGCG 7200
CPC0740            GCCATCATTTTGCTCGTGGCGCACTACATGTACTTGATCCCAGGGCTGCAGGCAGCAGCT 7200
YAP2007            GCCATCATTTTGCTCGTGGCGCACTACATGTACTTGATCCCAGGGCTGCAGGCAGCAGCT 7200
FSS13025           GCCATCATTTTGCTCGTGGCGCACTACATGTACTTGATCCCAGGGCTGCAGGCAGCAGCT 7200
PLCal_ZV           GCCATCATTTTGCTCGTGGCGCACTACATGTACTTGATCCCAGGGCTGCAGGCAGCAGCT 7089
SV0127/14          GCTATCATTTTGCTCGTGGCGCACTACATGTACTTGATCCCAGGGCTGCAGGCAGCAGCT 7200
8375               GCCATCATTTTGCTCGTGGCGCACTACATGTACTTGATCCCAGGGCTGCAGGCAGCAGCT 7200
103344             GCCATCATTTTGCTCGTGGCGCACTACATGTACTTGATCCCAGGGCTGCAGGCAGCAGCT 7200
BrasilZKV2015      GCCATCATTTTGCTCGTGGCGCACTACATGTACTTGATCCCAGGGCTGCAGGCAGCAGCT 7200
GD01               GCCATCATTTTGCTCGTGGCGCACTACATGTACTTGATCCCAGGGCTGCAGGCAGCAGCT 7200
Martinique         GCCATCATTTTGCTCGTGGCGCACTACATGTACTTGATCCCAGGGCTGCAGGCAGCAGCT 7200
NatalRGN           GCCATCATTTTGCTCGTGGCGCACTACATGTACTTGATCCCAGGGCTGCAGGCAGCAGCT 7200
HPF2013            GCCATCATTTTGCTCGTGGCGCACTACATGTACTTGATCCCAGGGCTGCAGGCAGCAGCT 7200
SPH2015            GCCATCATTTTGCTCGTGGCGCACTACATGTACTTGATCCCAGGGCTGCAGGCAGCAGCT 7200
Haiti2014          GCCATCATTTTGCTCGTGGCGCACTACATGTACTTGATCCCAGGGCTGCAGGCAGCAGCT 7200
PRVABC59           GCCATCATTTTGCTCGTGGCGCACTACATGTACTTGATCCCAGGGCTGCAGGCAGCAGCT 7200
BeH819015          GCCATCATTTTGCTCGTGGCGCACTACATGTACTTGATCCCAGGGCTGCAGGCAGCAGCT 7200
Z1106033           GCCATCATTTTGCTCGTGGCGCACTACATGTACTTGATCCCAGGGCTGCAGGCAGCAGCT 7200
BEH819966          GCCATCATTTTGCTCGTGGCGCACTACATGTACTTGATCCCAGGGCTGCAGGCAGCAGCT 7200
SSABr              GCCATCATTTTGCTCGTGGCGCACTACATGTACTTGATCCCAGGGCTGCAGGCAGCAGCT 7200
Beh815744          GCCATCATTTTGCTCGTGGCGCACTACATGTACTTGATCCCAGGGCTGCAGGCAGCAGCT 7200
BEH818995          GCCATCATTTTGCTCGTGGCGCACTACATGTACTTGATCCCAGGGCTGCAGGCAGCAGCT 7200
IbH30656           GCCATCATTCTGCTTGTGGCACACTACATGTATTTGATCCCAGGTTTGCAGGCAGCAGCA 7182
ArD128000          GCCATCATTCTGCTTGTAGCACACTACATGTACTTGATCCCAGGTTTGCAGGCAGCAGCA 7200
ArD7117            GCCATCATTCTGCTTGTGGCACACTACATGTACTTGATCCCAGGTTTGCAGGCAGCAGCA 7200
ARD_41519          GCCATCATTCTGCTTGTGGCACACTACATGTACTTGATCCCAGGTTTGCAGGCAGCAGCA 7200
ARD157995          GCTATCATTCTGCTTGTGGCGCACTACATGTACTTGATCCCAGGCCTACAAGCGGCAGCA 7200
MR_766             GCTATCATTCTGCTTGTGGCGCACTACATGTACTTGATCCCAGGCCTACAAGCGGCAGCA 7188
ARD158084          GCTATCATTCTGCTTGTGGCGCACTACATGTACTTGATCCCAGGCCTACAAGCGGCAGCA 7200
ArB1362            GCTATCATTCTGCTTGTGGCACACTACATGTACTTGATCCCAGGCCTACAGGCAGCAGCA 7200
ARB13565           GCTATCATTCTGCTTGTGGCACACTATATGTACTTGATCCCAGGCCTACAGGCAGCAGCA 7200
ARB7701            GCTATCATTCTGCTTGTGGCACACTATATGTACTTGATCCCAGGCCTACAGGCAGCAGCA 7200
ARB15076           GCTATCATTCTGCTTGTGGCACACTACATGTACTTGATCCCAGGCCTACAGGCAGCAGCA 7182
                   ** ****** **** ** ** ***** ***** ***********  * ** ** *****

P6-740             GCGCGCGCTGCCCAGAAGAGAACGGCAGCTGGCATCATGAAGAACCCTGTTGTGGATGGA 7260
CPC0740            GCGCGGGCTGCCCAGAAGAGAACGGCAGCTGGCATCATGAAGAACCCTGTTGTGGATGGA 7260
YAP2007            GCGCGTGCTGCCCAGAAGAGAACGGCAGCTGGCATCATGAAGAACCCTGTTGTGGATGGA 7260
FSS13025           GCGCGTGCTGCCCAGAAGAGAACGGCAGCTGGCATCATGAAGAACCCTGTTGTGGATGGA 7260
PLCal_ZV           GCGCGTGCTGCCCAGAAGAGAACGGCAGCTGGCATCATGAAGAACCCTGTCGTGGATGGA 7149
SV0127/14          GCGCGTGCTGCCCAGAAGAGAACGGCAGCTGGCATCATGAAGAACCCTGTTGTGGATGGA 7260
8375               GCGCGTGCTGCCCAGAAGAGAACGGCAGCTGGCATCATGAAGAACCCTGTTGTGGATGGA 7260
103344             GCGCGTGCTGCCCAGAAGAGAACGGCAGCTGGCATCATGAAGAACCCTGTTGTGGATGGA 7260
BrasilZKV2015      GCGCGTGCTGCCCAGAAGAGAACGGCAGCTGGCATCATGAAGAACCCTGTTGTGGATGGA 7260
GD01               GCGCGTGCTGCCCAGAAGAGAACGGCAGCTGGCATCATGAAGAACCCTGTTGTGGATGGA 7260
Martinique         GCGCGTGCTGCCCAGAAGAGAACGGCAGCTGGCATCATGAAGAACCCTGTTGTGGATGGA 7260
```

```
NatalRGN         GCGCGTGCTGCCCAGAAGAGAACGGCAGCTGGCATCATGAAGAACCCTGTTGTGGATGGA 7260
HPF2013          GCGCGTGCTGCCCAGAAGAGAACGGCAGCTGGCATCATGAAGAACCCTGTTGTGGATGGA 7260
SPH2015          GCGCGTGCTGCCCAGAAGAGAACGGCAGCTGGCATCATGAAGAACCCTGTTGTGGATGGA 7260
Haiti2014        GCGCGTGCTGCCCAGAAGAGAACGGCAGCTGGCATCATGAAGAACCCTGTTGTGGATGGA 7260
PRVABC59         GCGCGTGCTGCCCAGAAGAGAACGGCAGCTGGCATCATGAAGAACCCTGTTGTGGATGGA 7260
BeH819015        GCGCGTGCTGCCCAGAAGAGAACGGCAGCTGGCATCATGAAGAACCCTGTTGTGGATGGA 7260
Z1106033         GCGCGTGCTGCCCAGAAGAGAACGGCAGCTGGCATCATGAAGAACCCTGTTGTGGATGGA 7260
BEH819966        GCGCGTGCTGCCCAGAAGAGAACGGCAGCTGGCATCATGAAGAACCCTGTTGTGGATGGA 7260
SSABr            GCGCGTGCTGCCCAGAAGAGAACGGCAGCTGGCATCATGAAGAACCCTGTTGTGGATGGA 7260
Beh815744        GCGCGTGCTGCCCAGAAGAGAACGGCAGCTGGCATCATGAAGAACCCTGTTGTGGATGGA 7260
BEH818995        GCGCGTGCTGCCCAGAAGAGAACGGCAGCTGGCATCATGAAGAACCCTGTTGTGGATGGA 7260
IbH30656         GCACGTGCCGCCCAGAAGAGGACAGCAGCTGGCATCATGAAGAATCCCGTTGTTGATGGA 7242
ArD128000        GCACGTGCTGCCCAGAAGAGGACAGCAGCTGGCATCATGAAAAATCCCGTTGTGGATGGA 7260
ArD7117          GCACGTGCTGCCCAGAAGAGGACAGCAGCTGGCATCATGAAGAATCCCGTTGTGGATGGA 7260
ARD_41519        GCACGTGCTGCCCAGAAGAGGACAGCAGCTGGCATCATGAAGAATCCCGTTGTGGATGGA 7260
ARD157995        GCGCGTGCTGCCCAGAAAAGGACAGCAGCTGGCATCATGAAGAATCCCGTTGTGGATGGA 7260
MR_766           GCGCGTGCTGCCCAGAAAAGGACAGCAGCTGGCATCATGAAGAATCCCGTTGTGGATGGA 7248
ARD158084        GCGCGTGCTGCCCAGAAAAGGACAGCAGCTGGCATCATGAAGAATCCCGTTGTGGATGGA 7260
ArB1362          GCGCGTGCTGCCCAGAAGAGAACAGCAGCTGGCATCATGAAGAATCCCGTTGTGGATGGA 7260
ARB13565         GCGCGTGCTGCCCAGAAGAGAACAGCAGCTGGCATCATGAAGAATCCCGTTGTGGATGGA 7260
ARB7701          GCGCGTGCTGCCCAGAAGAGAACAGCAGCTGGCATCATGAAGAATCCCGTTGTGGATGGA 7260
ARB15076         GCGCGTGCTGCCCAGAAGAGAACAGCAGCTGGCATCATGAAGAATCCCGTTGTGGATGGA 7242
                 ** ** ** ******** ** ** ***************** ** ** ** ** ******

P6-740           ATAGTGGTGACTGACATTGACACAATGACAATTGACCCCCAAGTGGAGAAAAAGATGGGA 7320
CPC0740          ATAGTGGTGACTGACATTGACACAATGACAATTGACCCCCAAGTGGAAAAAAAGATGGGG 7320
YAP2007          ATAGTGGTGACTGACATTGACACAATGACAATTGACCACCGAGTGGAGAAAAAGATGGGA 7320
FSS13025         ATAGTGGTGACTGACATTGACACAATGACAATTGACCCCCAAGTGGAGAAAAAGATGGGA 7320
PLCal_ZV         ATAGTGGTGACTGACATTGACACAATGACAATTGACCCCCAAGTGGAGAAAAAGATGGGA 7209
SV0127/14        ATAGTGGTGACTGACATTGACACAATGACTATTGACCCCCAAGTGGAGAAAAAGATGGGA 7320
8375             ATAGTGGTGACTGACATTGACACAATGACAATTGACCCCCAAGTGGAGAAAAAGATGGGA 7320
103344           ATAGTGGTGACTGACATTGACACAATGACAATTGACCCCCAAGTGGAGAAAAAGATGGGA 7320
BrasilZKV2015    ATAGTGGTGACTGACATTGACACAATGACAATTGACCCCCAAGTGGAGAAAAAGATGGGA 7320
GD01             ATAGTGGTGACTGACATTGACACAATGACAATTGACCCCCAAGTGGAGAAAAAGATGGGA 7320
Martinique       ATAGTGGTGACTGACATTGACACAATGACAATTGACCCCCAAGTGGAGAAAAAGATGGGA 7320
NatalRGN         ATAGTGGTGACTGACATTGACACAATGACAATTGACCCCCAAGTGGAGAAAAAGATGGGA 7320
HPF2013          ATAGTGGTGACTGACATTGACACAATGACAATTGACCCCCAAGTGGAGAAAAAGATGGGA 7320
SPH2015          ATAGTGGTGACTGACATTGACACAATGACAATTGACCCCCAAGTGGAGAAAAAGATGGGA 7320
Haiti2014        ATAGTGGTGACTGACATTGACACAATGACAATTGACCCCCAAGTGGAGAAAAAGATGGGA 7320
PRVABC59         ATAGTGGTGACTGACATTGACACAATGACAATTGACCCCCAAGTGGAGAAAAAGATGGGA 7320
BeH819015        ATAGTGGTGACTGACATTGACACAATGACAATTGACCCCCAAGTGGAGAAAAAGATGGGA 7320
Z1106033         ATAGTGGTGACTGACATTGACACAATGACAATTGACCCCCAAGTGGAGAAAAAGATGGGA 7320
BEH819966        ATAGTGGTGACTGACATTGACACAATGACAATTGACCCCCAAGTGGAGAAAAAGATGGGA 7320
SSABr            ATAGTGGTGACTGACATTGACACAATGACAATTGACCCCCAAGTGGAGAAAAAGATGGGA 7320
Beh815744        ATAGTGGTGACTGACATTGACACAATGACAATTGACCCCCAAGTGGAGAAAAAGATGGGA 7320
BEH818995        ATAGTGGTGACTGACATTGACACAATGACAATTGACCCCCAAGTGGAGAAAAAGATGGGA 7320
IbH30656         ATAGTGGTGACTGACATTGACACAATGACAATTGACCCCCAAGTGGAGAAGAAGATGGGA 7302
ArD128000        ATAGTGGTGACTGACATTGACACAATGACAATTGACCCCCAAGTGGAGAAGAAGATGGGA 7320
ArD7117          ATAGTGGTGACTGACATTGACACAATGACAATTGACCCCCAAGTGGAGAAGAAGATGGGA 7320
ARD_41519        ATAGTGGTGACTGACATTGACACAATGACAATTGACCCCCAAGTGGAGAAGAAGATGGGA 7320
ARD157995        ATAGTGGTAACTGACATTGACACAATGACAATAGACCCCCAGGTGGAGAAGAAGATGGGA 7320
MR_766           ATAGTGGTAACTGACATTGACACAATGACAATAGACCCCCAGGTGGAGAAGAAGATGGGA 7308
ARD158084        ATAGTGGTAACTGACATTGACACAATGACAATAGACCCCCAGGTGGAGAAGAAGATGGGA 7320
ArB1362          ATAGTGGTAACTGACATTGACACAATGACAATTGACCCCCAAGTGGAGAAGAAGATGGGA 7320
ARB13565         ATAGTGGTAACTGACATTGACACAATGACAATTGACCCCCAAGTGGAGAAGAAGATGGGA 7320
ARB7701          ATAGTGGTAACTGACATTGACACAATGACAATTGACCCCCAAGTGGAGAAGAAGATGGGA 7320
ARB15076         ATAGTGGTAACTGACATTGACACAATGACAATTGACCCCCAAGTGGAGAAGAAGATGGGA 7302
                 ******** ******************** ** **** **  ***** ** ********

P6-740           CAAGTGCTACTCATAGCAGTAGCCATCTCCAGTGCCGTTCTGCTGCGCACCGCCTGGGGG 7380
CPC0740          CAGGTGCTACTCATAGCAGTAGCCGTCTCCAGCGCCATACTGTCGCGGACCGCCTGGGGG 7380
YAP2007          CAGGTGCTACTCATAGCAGTAGCCGTCTCCAGCGCCATACTGTCGCGGACCGCCTGGGGG 7380
FSS13025         CAGGTGCTACTCATAGCAGTAGCTGTCTCCAGCGCCATACTGTCGCGGACCGCCTGGGGG 7380
PLCal_ZV         CAGGTGCTACTCATAGCAGTAGCCGTCTCCAGCGCCATACTGTCGCGGACCGCCTGGGGG 7269
SV0127/14        CAGGTGCTACTCATAGCAGTAGCCGTCTCCAGCGCCATACTGTCGCGGACCGCCTGGGGG 7380
8375             CAGGTGCTACTCATAGCAGTAGCCGTCTCCAGCGCCATACTGTCGCGGACCGCCTGGGGG 7380
103344           CAGGTGCTACTCATAGCAGTAGCCGTCTCCAGCGCCATACTGTCGCGGACCGCCTGGGGG 7380
BrasilZKV2015    CAGGTGCTACTCATAGCAGTAGCCGTCTCCAGCGCCATACTGTCGCGGACCGCCTGGGGG 7380
```

```
GD01               CAGGTGCTACTCATAGCAGTAGCCGTCTCCAGCGCCATACTGTCGCGGACCGCCTGGGGG 7380
Martinique         CAGGTGCTACTCATAGCAGTAGCCGTCTCCAGCGCCATACTGTCGCGGACCGCCTGGGGG 7380
NatalRGN           CAGGTGCTACTCATAGCAGTAGCAGTCTCCAGCGCCATACTGTCGCGGACCGCCTGGGGG 7380
HPF2013            CAGGTGCTACTCATAGCAGTAGCCGTCTCCAGCGCCATACTGTCGCGGACCGCCTGGGGG 7380
SPH2015            CAGGTGCTACTCATGGCAGTAGCCGTCTCCAGCGCCATACTGTCGCGGACCGCCTGGGGG 7380
Haiti2014          CAGGTGCTACTCATGGCAGTAGCCGTCTCCAGCGCCATACTGTCGCGGACCGCCTGGGGG 7380
PRVABC59           CAGGTGCTACTCATAGCAGTAGCCGTCTCCAGCGCCATACTGTCGCGGACCGCCTGGGGG 7380
BeH819015          CAGGTGCTACTCATAGCAGTAGCCGTCTCCAGCGCCATACTGTCGCGGACCGCCTGGGGG 7380
Z1106033           CAGGTGCTACTCATAGCAGTAGCCGTCTCCAGCGCCATACTGTCGCGGACCGCCTGGGGG 7380
BEH819966          CAGGTGCTACTCATAGCAGTAGCCGTCTCCAGCGCCATACTGTCGCGGACCGCCTGGGGG 7380
SSABr              CAGGTGCTACTCATAGCAGTAGCCGTCTCCAGCGCCATACTGTCGCGGACCGCCTGGGGG 7380
Beh815744          CAGGTGCTACTCATAGCAGTAGCCGTCTCCAGCGCCATACTGTCGCGGACCGCCTGGGGG 7380
BEH818995          CAGGTGCTACTCATAGCAGTAGCCGTCTCCAGCGCCATACTGTCGCGGACCGCCTGGGGG 7380
IbH30656           CAAGTGTTACTCATAGCAGTAGCTGCCTCCAGTGCCGTGCTGCTGCGGACCGCTTGGGGA 7362
ArD128000          CAAGTGTTACTCATAGCAGTAGCTGTCTCCAGTGCTGTGCTGCTGCGGACCGCTGGGGGA 7380
ArD7117            CAAGTGTTACTCATAGCAGTAGCTGTCTCCAGTGCTGTGCTGCTGCGGACCGCTTGGGGA 7380
ARD_41519          CAAGTGTTACTCATAGCAGTAGCTGTCTCCAGTGCTGTGCTACTGCGGACCGCTTGGGGA 7380
ARD157995          CAAGTGTTACTCATAGCAGTAGCCATCTCCAGTGCTGTGCTGCTGCGGACCGCCTGGGGA 7380
MR_766             CAAGTGTTACTCATAGCAGTAGCCATCTCCAGTGCTGTGCTGCTGCGGACCGCCTGGGGA 7368
ARD158084          CAAGTGTTACTCATAGCAGTAGCCATCTCCAGTGCTGTGCTGCTGCGGACCGCCTGGGGA 7380
ArB1362            CAAGTGTTACTCATAGCAGTAGCCATCTCCAGTGCTGTGCTGCTGCGGACTGCTTGGGGA 7380
ARB13565           CAAGTGTTACTTATAGCAGTAGCCATCTCCAGTGCTGTGCTGCTGCGGACTGCTTGGGGA 7380
ARB7701            CAAGTGTTACTTATAGCAGTAGCCATCTCCAGTGCTGTGCTGCTGCGGACTGCTTGGGGA 7380
ARB15076           CAAGTGTTACTCATAGCAGTAGCCATCTCCAGTGCTGTGCTGCTGCGAACTGCTTGGGGA 7362
                   ** *** **** ** ********   ****** **  * **   *** ** **  ****

P6-740             TGGGGGGAGGCTGGGGCCCTGATCACAGCCGCAACTTCCACTTTGTGGGAAGGCTCTCCG 7440
CPC0740            TGGGGGGAGGCTGGGGCCCTGATCACAGCTGCAACTTCCACCTTGTGGGAAGGCTCTCCG 7440
YAP2007            TGGGGGGAGGCTGGGGCCCTGATCACAGCTGCAACTTCCACTTTGTGGGAAGGCTCTCCG 7440
FSS13025           TGGGGTGAGGCTGGGGCCCTGATCACAGCTGCAACTTCCACTTTGTGGGAGGGCTCTCCG 7440
PLCal_ZV           TGGGGGGAGGCTGGGGCCCTGATCACAGCTGCAACTTCCACTTTGTGGGAAGGCTCTCCG 7329
SV0127/14          TGGGGGGAAGCTGGGGCCCTGATCACAGCTGCAACTTCCACTTTGTGGGAAGGCTCTCCG 7440
8375               TGGGGGGAGGCTGGGGCCCTGATCACAGCCGCAACTTCCACTTTGTGGGAAGGCTCTCCG 7440
103344             TGGGGGGAGGCTGGGGCCCTGATCACAGCCGCAACTTCCACTTTGTGGGAAGGCTCTCCG 7440
BrasilZKV2015      TGGGGGGAGGCTGGGGCCCTGATCACAGCCGCAACTTCCACTTTGTGGGAAGGCTCTCCG 7440
GD01               TGGGGGGAGGCTGGGGCCCTGATCACAGCCGCAACTTCCACTTTGTGGGAAGGCTCTCCG 7440
Martinique         TGGGGGGAGGCTGGGGCCCTGATCACAGCCGCAACTTCCACTTTGTGGGAAGGCTCTCCG 7440
NatalRGN           TGGGGGGAGGCTGGGGCCCTGATCACAGCCGCAACTTCCACTTTGTGGGAAGGCTCTCCG 7440
HPF2013            TGGGGGGAGGCTGGGGCCCTGATCACAGCGGCAACTTCCACTTTGTGGGAAGGCTCTCCG 7440
SPH2015            TGGGGGGAGGCTGGGGCCCTGATCACAGCCGCAACTTCCACTTTGTGGGAAGGCTCTCCG 7440
Haiti2014          TGGGGGGAGGCTGGGGCCCTGATCACAGCCGCAACTTCCACTTTGTGGGAAGGCTCTCCG 7440
PRVABC59           TGGGGGGAGGCTGGGGCTCTGATCACAGCCGCAACTTCCACTTTGTGGGAAGGCTCTCCG 7440
BeH819015          TGGGGGGAGGCTGGGGCCCTGATCACAGCCGCAACTTCCACTTTGTGGGAAGGCTCTCCG 7440
Z1106033           TGGGGGGAGGCTGGGGCCCTGATCACAGCCGCAACTTCCACTTTGTGGGAAGGCTCTCCG 7440
BEH819966          TGGGGGGAGGCTGGGGCCCTGATCACAGCCGCAACTTCCACTTTGTGGGAAGGCTCTCCG 7440
SSABr              TGGGGGGAGGCTGGGGCCCTGATCACAGCCGCAACTTCCACTTTGTGGGAAGGCTCTCCG 7440
Beh815744          TGGGGGGAGGCTGGGGCCCTGATCACAGCCGCAACTTCCACTTTGTGGGAAGGCTCTCCG 7440
BEH818995          TGGGGGGAGGCTGGGGCCCTGATCACAGCCGCAACTTCCACTTTGTGGGAAGGCTCTCCG 7440
IbH30656           TGGGGGGAGGCTGGGGCTCTGATCACAGCAGCAACCTCCACCTTATGGGAAGGCTCTCCA 7422
ArD128000          TGGGGGGAGGCTGGGGCTCTGATCACAGCAGCTACCTCCACCTTATGGGAAGGCTCTCCA 7440
ArD7117            TGGGGGGAGGCTGGGGCTCTGATCACAGCAGCAACCTCCACCTTATGGGAAGGCTCTCCA 7440
ARD_41519          TGGGGGGAGGCTGGGGCTCTGATCACAGCAGCAACCTCCACCTTATGGGAAGGCTCTCCA 7440
ARD157995          TGGGGGGAGGCTGGAGCTCTGATCACAGCAGCGACCTCCACCTTGTGGGAAGGCTCTCCA 7440
MR_766             TGGGGGGAGGCTGGAGCTCTGATCACAGCAGCGACCTCCACCTTGTGGGAAGGCTCTCCA 7428
ARD158084          TGGGGGGAGGCTGGAGCTCTGATCACAGCAGCGACCTCCACCTTGTGGGAAGGCTCTCCA 7440
ArB1362            TGGGGGGAAGCTGGAGCTCTGATCACAGCAGCAACCTCCACTTTGTGGGAAGGTTCTCCA 7440
ARB13565           TGGGGGGAGGCTGGAGCTCTGATTACTGCAGCAACCTCCACCTTGTGGGAAGGTTCTCCA 7440
ARB7701            TGGGGGGAGGCTGGAGCTCTGATTACTGCAGCAACCTCCACCTTGTGGGAAGGTTCTCCA 7440
ARB15076           TGGGGGGAGGCTGGAGCTCTGATCACAGCAGCAACCTCCACCTTGTGGGAAGGTTCTCCA 7422
                   ***** ** ***** ** ***** ** ** ** ** ***** ** ***** ** *****

P6-740             AATAAATACTGGAACTCCTCCACAGCCACTTCACTGTGTAACATTTTTAGGGGAAGTTAC 7500
CPC0740            AACAAGTACTGGAACTCCTCCACAGCCACTTCACTGTGTAACATTTTTAGGGGAAGTTAC 7500
YAP2007            AACAAGTACTGGAACTCCTCCACAGCCACTTCACTGTGTAACATTTTTAGGGGAAGTTAC 7500
FSS13025           AACAAGTACTGGAACTCCTCCACAGCCACCTCACTGTGTAACATTTTTAGGGGAAGCTAC 7500
PLCal_ZV           AACAAGTACTGGAACTCCTCTACAGCCACTTCACTGTGTAACATTTTTAGGGGAAGTTAC 7389
SV0127/14          AACAAGTACTGGAACTCCTCTACAGCCACTTCACTGTGCAACATTTTTAGGGGAAGTTAC 7500
8375               AACAAGTACTGGAACTCCTCTACAGCCACTTCACTGTGTAACATTTTTAGGGGAAGTTAC 7500
```

```
103344             AACAAGTACTGGAACTCCTCTACAGCCACTTCACTGTGTAACATTTTTAGGGGAAGTTAT 7500
BrasilZKV2015      AACAAGTACTGGAACTCCTCTACAGCCACTTCACTGTGTAACATTTTTAGGGGAAGTTAC 7500
GD01               AACAAGTACTGGAACTCCTCTACAGCCACTTCACTGTGTAACATTTTTAGGGGAAGTTAC 7500
Martinique         AACAAGTACTGGAACTCCTCTACAGCCACTTCACTGTGTAACATTTTTAGGGGAAGTTAC 7500
NatalRGN           AACAAGTACTGGAACTCCTCTACAGCCACTTCACTGTGTAACATTTTTAGGGGAAGTTAC 7500
HPF2013            AACAAGTACTGGAACTCCTCTACAGCCACTTCACTGTGTAACATTTTTAGGGGAAGTTAC 7500
SPH2015            AACAAGTACTGGAACTCCTCTACAGCCACTTCACTGTGTAACATTTTTAGGGGAAGTTAC 7500
Haiti2014          AACAAGTACTGGAACTCCTCTACAGCCACTTCACTGTGTAACATTTTTAGGGGAAGTTAC 7500
PRVABC59           AACAAGTACTGGAACTCCTCTACAGCCACTTCACTGTGTAACATTTTTAGGGGAAGTTAC 7500
BeH819015          AACAAGTACTGGAACTCCTCTACAGCCACTTCACTGTGTAACATTTTTAGGGGAAGTTAC 7500
Z1106033           AACAAGTACTGGAACTCCTCTACAGCCACTTCACTGTGTAACATTTTTAGGGGAAGTTAC 7500
BEH819966          AACAAGTACTGGAACTCCTCTACAGCCACTTCACTGTGTAACATTTTTAGGGGAAGTTAC 7500
SSABr              AACAAGTACTGGAACTCCTCTACAGCCACTTCACTGTGTAACATTTTTAGGGGAAGTTAC 7500
Beh815744          AACAAGTACTGGAACTCCTCTACAGCCACTTCACTGTGTAACATTTTTAGGGGAAGTTAC 7500
BEH818995          AACAAGTACTGGAACTCCTCTACAGCCACTTCACTGTGTAACATTTTTAGGGGAAGTTAC 7500
IbH30656           AACAAATACTGGAACTCCTCTACAGCCACTTCACTGTGCAATATCTTCAGAGGAAGTTAT 7482
ArD128000          AACAAATACTGGAACTCCTCTACAGCCACCTCACTATGCAACATCTTTAGAGGAAGTTAT 7500
ArD7117            AACAAATACTGGAACTCCTCCACAGCCACCTCACTGTGCAACATCTTTAGAGGAAGTTAT 7500
ARD_41519          AACAAATACTGGAACTCCTCCACAGCCACCTCACTGTGCAACATCTTTAGAGGAAGTTAT 7500
ARD157995          AACAAATACTGGAACTCCTCTACAGCCACCTCACTGTGCAACATCTTCAGAGGAAGCTAT 7500
MR_766             AACAAATACTGGAACTCCTCTACAGCCACCTCACTGTGCAACATCTTCAGAGGAAGCTAT 7488
ARD158084          AACAAATACTGGAACTCCTCTACAGCCACCTCACTGTGCAACATCTTCAGAGGAAGCTAT 7500
ArB1362            AACAAATACTGGAACTCCTCTACAGCCACCTCACTGTGCAACATCTTTAGAGGAAGTTAC 7500
ARB13565           AACAAATACTGGAACTCCTCTACAGCCACCTCACTGTGCAACATCTTCAGAGGAAGTTAC 7500
ARB7701            AACAAATACTGGAACTCCTCTACAGCCACCTCACTGTGCAACATCTTCAGAGGAAGTTAC 7500
ARB15076           AACAAATACTGGAACTCCTCTACAGCCACCTCACTGTGCAACATCTTTAGAGGAAGTTAC 7482
                   ** ** ************** ******** ***** ** ** ** ** ** ***** **

P6-740             TTGGCTGGAGCTTCTCTTATTTACACAGTAACAAGAAACGCTGGCCTGGTCAAGAGACGT 7560
CPC0740            TTGGCTGGAGCTTCTCTAATCTACACAGTAACAAGAAACGCTGGCTTGGTCAAGAGACGT 7560
YAP2007            TTGGCTGGAGCTTCTCTAATCTACACAGTAACAAGAAACGCTGGCTTGGTCAAGAGACGT 7560
FSS13025           TTGGCTGGAGCTTCTCTAATCTACACAGTAACAAGAAACGCTGGCTTGGTCAAGAGACGT 7560
PLCal_ZV           TTGGCTGGAGCTTCTCTAATCTACACAGTAACAAGAAACGCTGGCTTGGTCAAGAGACGT 7449
SV0127/14          TTGGCTGGAGCTTCTCTAATCTACACAGTAACAAGAAACGCTGGCTTGGTCAAGAGACGT 7560
8375               TTGGCTGGAGCTTCTCTAATCTACACAGTAACAAGAAACGCTGGCTTGGTCAAGAGACGT 7560
103344             TTGGCTGGAGCTTCTCTAATCTACACAGTAACAAGAAACGCTGGCTTGGTCAAGAGACGT 7560
BrasilZKV2015      TTGGCTGGAGCTTCTCTAATCTACACAGTAACAAGAAACGCTGGCTTGGTCAAGAGACGT 7560
GD01               TTGGCTGGAGCTTCTCTAATCTACACAGTAACAAGAAACGCTGGCTTGGTCAAGAGACGT 7560
Martinique         TTGGCTGGAGCTTCTCTAATCTACACAGTAACAAGAAACGCTGGCTTGGTCAAGAGACGT 7560
NatalRGN           TTGGCTGGAGCTTCTCTAATCTACATAGTAACAAGAAACGCTGGCTTGGTCAAGAGACGT 7560
HPF2013            TTGGCTGGAGCTTCTCTAATCTACACAGTAACAAGAAACGCTGGCTTGGTCAAGAGACGT 7560
SPH2015            TTGGCTGGAGCTTCTCTAATCTACACAGTAACAAGAAACGCTGGCTTGGTCAAGAGACGT 7560
Haiti2014          TTGGCTGGAGCTTCTCTAATCTACACAGTAACAAGAAACGCTGGCTTGGTCAAGAGACGT 7560
PRVABC59           TTGGCTGGAGCTTCTCTAATCTACACAGTAACAAGAAACGCTGGCTTGGTCAAGAGACGT 7560
BeH819015          TTGGCTGGAGCTTCTCTAATCTACACAGTAACAAGAAACGCTGGCTTGGTCAAGAGACGT 7560
Z1106033           TTGGCTGGAGCTTCTCTAATCTACACAGTAACAAGAAACGCTGGCTTGGTCAAGAGACGT 7560
BEH819966          TTGGCTGGAGCTTCTCTAATCTACACAGTAACAAGAAACGCTGGCTTGGTCAAGAGACGT 7560
SSABr              TTGGCTGGAGCTTCTCTAATCTACACAGTAACAAGAAACGCTGGCTTGGTCAAGAGACGT 7560
Beh815744          TTGGCTGGAGCTTCTCTAATCTACACAGTAACAAGAAACGCTGGCTTGGTCAAGAGACGT 7560
BEH818995          TTGGCTGGAGCTTCTCTAATCTACACAGTAACAAGAAACGCTGGCTTGGTCAAGAGACGT 7560
IbH30656           TTGGCAGGGGCTTCCCTTATTTACACAGTGACAAGAAATGCCGGTCTGGTTAAGAGACGT 7542
ArD128000          TTGGCAGGGGCTTCCCTTATTTACACAGTGACAAGAAATGCCGGTCTGGTTAAGAGACGT 7560
ArD7117            TTGGCAGGGGCTTCCCTTATTTACACAGTGACAAGAAATGCCGGTCTGGTTAAGAGACGT 7560
ARD_41519          TTGGCAGGGGCTTCCCTTATTTACACAGTGACAAGAAATGCCGGTCTGGTTAAGAGACGT 7560
ARD157995          CTGGCAGGAGCTTCCCTTATCTATACAGTGACGAGAAACGCTGGCCTGGTTAAGAGACGT 7560
MR_766             CTGGCAGGAGCTTCCCTTATCTATACAGTGACGAGAAACGCTGGCCTGGTTAAGAGACGT 7548
ARD158084          CTGGCAGGAGCTTCCCTTATCTATACAGTGACGAGAAACGCTGGCCTGGTTAAGAGACGT 7560
ArB1362            CTGGCAGGCGCTTCTCTTATTTACACAGTGACAAGAAATGCTGGCCTGGTCAAGAGACGT 7560
ARB13565           CTGGCAGGCGCTTCTCTTATTTATACAGTGACAAGAAATGCTGGCCTAGTCAAGAGACGT 7560
ARB7701            CTGGCAGGCGCTTCTCTTATTTATACAGTGACAAGAAATGCTGGCCTAGTCAAGAGACGT 7560
ARB15076           CTGGCAGGCGCTTCTCTCATTTATACAGTGACAAGAAATGCTGGCCTAGTCAAGAGACGT 7542
                    **** ** ***** ** ** ** * *** ** ***** ** **  * ** *********

P6-740             GGAGGTGGAACGGGAGAGACCCTGGGGGAGAAATGGAAGGCCCGCCTGAACCAGATGTCG 7620
CPC0740            GGGGGTGGAACGGGAGAGACCCTGGGAGAGAAATGGAAGGCCCGCCTGAACCAGATGTCG 7620
YAP2007            GGGGGTGGAACGGGAGAGACCCTGGGAGAGAAATGGAAGGCCCGCCTGAACCAGATGTCG 7620
FSS13025           GGGGGTGGAACGGGAGAGACCCTGGGAGAGAAATGGAAGGCCCGCCTGAACCAGATGTCG 7620
PLCal_ZV           GGGGGTGGAACAGGAGAGACCCTGGGAGAGAAATGGAAGGCCCGCTTGAACCAGATGTCG 7509
```

```
SV0127/14          GGGGGTGGAACAGGAGAGACCCTGGGAGAGAAATGGAAGGCCCGCTTGAACCAGATGTCG 7620
8375               GGGGGTGGAACAGGAGAGACCCTGGGAGAGAAATGGAAGGCCCGCTTGAACCAGATGTCG 7620
103344             GGGGGTGGAACAGGAGAGACCCTGGGAGAGAAATGGAAGGCCCGCTTGAACCAGATGTCG 7620
BrasilZKV2015      GGGGGTGGAACAGGAGAGACCCTGGGAGAGAAATGGAAGGCCCGCTTGAACCAGATGTCG 7620
GD01               GGGGGTGGAACAGGAGAGACCCTGGGAGAGAAATGGAAGGCCCGCTTGAACCAGATGTCG 7620
Martinique         GGGGGTGGAACAGGAGAGACCCTGGGAGAGAAATGGAAGGCCCGCTTGAACCAGATGTCG 7620
NatalRGN           GGGGGTGGAACAGGAGAGACCCTGGGAGAGAAATGGAAGGCCCGCTTGAACCAGATGTCG 7620
HPF2013            GGGGGTGGAACAGGAGAGACCCTGGGAGAGAAATGGAAGGCCCGCTTGAACCAGATGTCG 7620
SPH2015            GGGGGTGGAACAGGAGAGACCCTGGGAGAGAAATGGAAGGCCCGCTTGAACCAGATGTCG 7620
Haiti2014          GGGGGTGGAACAGGAGAGACCCTGGGAGAGAAATGGAAGGCCCGCTTGAACCAGATGTCG 7620
PRVABC59           GGGGGTGGAACAGGAGAGACCCTGGGAGAGAAATGGAAGGCCCGCTTGAACCAGATGTCG 7620
BeH819015          GGGGGTGGAACAGGAGAGACCCTGGGAGAGAAATGGAAGGCCCGCTTGAACCAGATGTCG 7620
Z1106033           GGGGGTGGAACAGGAGAGACCCTGGGAGAGAAATGGAAGGCCCGCTTGAACCAGATGTCG 7620
BEH819966          GGGGGTGGAACAGGAGAGACCCTGGGAGAGAAATGGAAGGCCCGCTTGAACCAGATGTCG 7620
SSABr              GGGGGTGGAACAGGAGAGACCCTGGGAGAGAAATGGAAGGCCCGCTTGAACCAGATGTCG 7620
Beh815744          GGGGGTGGAACAGGAGAGACCCTGGGAGAGAAATGGAAGGCCCGCTTGAACCAGATGTCG 7620
BEH818995          GGGGGTGGAACAGGAGAGACCCTGGGAGAGAAATGGAAGGCCCGCTTGAACCAGATGTCG 7620
IbH30656           GGAGGTGGAACGGGAGAGACTCTGGGAGAGAAGTGGAAAGCCCGCCTGAACCAGATGTCG 7602
ArD128000          GGAGGTGGAACGGGAGAGACCCTGGGAGAGAAGTGGAAAGCCCGCCTGAACCAGATGTCG 7620
ArD7117            GGAGGTGGAACGGGAGAGACCCTGGGAGAGAAGTGGAAAGCCCGCCTGAACCAGATGTCG 7620
ARD_41519          GGAGGTGGAACGGGAGAGACCCTGGGAGAGAAGTGGAAAGCCCGCCTGAACCAGATGTCG 7620
ARD157995          GGAGGTGGGACGGGAGAGACTCTGGGAGAGAAGTGGAAAGCTCGTCTGAATCAGATGTCG 7620
MR_766             GGAGGTGGGACGGGAGAGACTCTGGGAGAGAAGTGGAAAGCTCGTCTGAATCAGATGTCG 7608
ARD158084          GGAGGTGGGACGGGAGAGACTCTGGGAGAGAAGTGGAAAGCTCGTCTGAATCAGATGTCG 7620
ArB1362            GGAGGTGGAACGGGAGAGACTCTAGGAGAGAAGTGGAAAGCTCGCCTGAATCAGATGTCG 7620
ARB13565           GGAGGTGGAACGGGAGAGACTCTAGGAGAGAAGTGGAAAGCTCGCCTGAATCAGATGTCG 7620
ARB7701            GGAGGTGGAACGGGAGAGACTCTAGGAGAGAAGTGGAAAGCTCGCCTGAATCAGATGTCG 7620
ARB15076           GGAGGTGGAACGGGAGAGACTCTAGGAGAGAAGTGGAAAGCTCGCCTGAATCAGATGTCG 7602
                   ** ***** ** ******** ** ** ***** ***** ** **  **** *********

P6-740             GCCCTGGAGTTTTACTCCTACAAAAAGTCAGGCATCACCGAAGTGTGCAGAGAAGAAGCC 7680
CPC0740            GCCCTGGAGTTCTACTCCTACAAAAAGTCAGGCATCACCGAGGTGTGCAGAGAAGAGGCC 7680
YAP2007            GCCCTAGAGTTCTACTCCTACAAAAAGTCAGGCATCACCGAGGTGTGCAGAGAAGAGGCC 7680
FSS13025           GCCCTGGAGTTCTACTCCTACAAAAAGTCAGGCATCACCGAGGTGTGCAGAGAAGAGGCC 7680
PLCal_ZV           GCCCTGGAGTTCTACTCCTACAAAAAGTCAGGCATCACCGAGGTGTGCAGAGAAGAGGCC 7569
SV0127/14          GCCCTGGAGTTCTACTCCTACAAAAAGTCAGGCATCACCGAGGTGTGCAGAGAAGAGGCC 7680
8375               GCCCTGGAGTTCTACTCCTACAAAAAGTCAGGCATCACTGAGGTGTGCAGAGAAGAGGCC 7680
103344             GCCCTGGAGTTCTACTCCTACAAAAAGTCAGGCATCACTGAGGTGTGCAGAGAAGAGGCC 7680
BrasilZKV2015      GCCCTGGAGTTCTACTCCTACAAAAAGTCAGGCATCACCGAGGTGTGCAGAGAAGAGGCC 7680
GD01               GCCCTGGAGTTCTACTCCTACAAAAAGTCAGGCATCACCGAGGTGTGCAGAGAAGAGGCC 7680
Martinique         GCCCTGGAGTTCTACTCCTACAAAAAGTCAGGCATCACCGAGGTGTGCAGAGAAGAGGCC 7680
NatalRGN           GCCCTGGAGTTCTACTCCTACAAAAAGTCAGGCATCACCGAGGTGTGCAGAGAAGAGGCC 7680
HPF2013            GCCCTGGAGTTCTACTCCTACAAAAAGTCAGGCATCACCGAGGTGTGCAGAGAAGAGGCC 7680
SPH2015            GCCCTGGAGTTCTACTCCTACAAAAAGTCAGGCATCACCGAGGTGTGCAGAGAAGAGGCC 7680
Haiti2014          GCCCTGGAGTTCTACTCCTACAAAAAGTCAGGCATCACCGAGGTGTGCAGAGAAGAGGCC 7680
PRVABC59           GCCCTGGAGTTCTACTCCTACAAAAAGTCAGGCATCACCGAGGTGTGCAGAGAAGAGGCC 7680
BeH819015          GCCCTGGAGTTCTACTCCTACAAAAAGTCAGGCATCACCGAGGTGTGCAGAGAAGAGGCC 7680
Z1106033           GCCCTGGAGTTCTACTCCTACAAAAAGTCAGGCATCACCGAGGTGTGCAGAGAAGAGGCC 7680
BEH819966          GCCCTGGAGTTCTACTCCTACAAAAAGTCAGGCATCACCGAGGTGTGCAGAGAAGAGGCC 7680
SSABr              GCCCTGGAGTTCTACTCCTACAAAAAGTCAGGCATCACCGAGGTGTGCAGAGAAGAGGCC 7680
Beh815744          GCCCTGGAGTTCTACTCCTACAAAAAGTCAGGCATCACCGAGGTGTGCAGAGAAGAGGCC 7680
BEH818995          GCCCTGGAGTTCTACTCCTACAAAAAGTCAGGCATCACCGAGGTGTGCAGAGAAGAGGCC 7680
IbH30656           GCTTTGGAGTTCTATTCTTACAAAAAGTCAGGCATCACCGAAGTGTGTAGGGAGGAGGCA 7662
ArD128000          GCCCTGGAGTTTTACTCTTACAAAAAGTCAGGCATCACCGAAGTGTGTAGGGAGGAGGCG 7680
ArD7117            GCCTTGGAGTTTTACTCTTACAAAAAGTCAGGCATCACCGAAGTGTGTAGGGAGGAGGCG 7680
ARD_41519          GCCTTGGAGTTCTACTCTTACAAAAAGTCAGGCATCACCGAAGTGTGTAGGGAGGAGGCG 7680
ARD157995          GCCCTGGAGTTCTACTCTTATAAAAAGTCAGGTATCACTGAAGTGTGTAGAGAGGAGGCT 7680
MR_766             GCCCTGGAGTTCTACTCTTATAAAAAGTCAGGTATCACTGAAGTGTGTAGAGAGGAGGCT 7668
ARD158084          GCCCTGGAGTTCTACTCTTATAAAAAGTCAGGTATCACTGAAGTGTGTAGAGAGGAGGCT 7680
ArB1362            GCCCTGGAGTTCTACTCTTACAAAAAGTCGGGCATCACTGAAGTGTGTAGAGAGGAGGCT 7680
ARB13565           GCCCTGGAGTTCTACTCTTACAAAAAGTCAGGCATCACTGAAGTGTGTAGAGAGGAGGCT 7680
ARB7701            GCCCTGGAGTTCTACTCTTACAAAAAGTCAGGCATCACTGAAGTGTGTAGAGAGGAGGCT 7680
ARB15076           GCCCTGGAGTTCTACTCTTACAAAAAGTCAGGCATCACTGAAGTGTGTAGAGAGGAGGCT 7662
                   **  * ***** ** ** ** ******** ** ***** ** ***** ** ** ** **

P6-740             CGCCGCGCCCTCAAGGACGGAGTGGCAACAGGAGGCCATGCTGTGTCCCGAGGAAGCGCA 7740
CPC0740            CGCCGTGCCCTCAAGGACGGTGTGGCAACAGGAGGCCATGCTGTGTCCCGAGGAAGTGCA 7740
YAP2007            CGCCGCGCCCTCAAGGACGGTGTGGCAACAGGAGGCCATGCTGTGTCCCGAGGAAGTGCA 7740
```

```
FSS13025        CGCCGCGCCCTCAAGGACGGTGTGGCAACGGGAGGCCACGCTGTGTCCCGAGGAAGTGCA 7740
PLCal_ZV        CGCCGCGCCCTCAAGGACGGTGTGGCAACGGGAGGCCATGCTGTGTCCCGAGGAAGTGCG 7629
SV0127/14       CGCCGCGCCCTCAAGGACGGTGTGGCAACGGGAGGCCATGCTGTGTCCCGAGGAAGTGCA 7740
8375            CGCCGCGCCCTCAAGGACGGTGTGGCAACGGGAGGCCATGCTGTGTCCCGAGGAAGTGCA 7740
103344          CGCCGCGCCCTCAAGGACGGTGTGGCAACGGGAGGCCATGCTGTGTCCCGAGGAAGTGCA 7740
BrasilZKV2015   CGCCGCGCCCTCAAGGACGGTGTGGCAACGGGAGGCCATGCTGTGTCCCGAGGAAGTGCA 7740
GD01            CGCCGCGCCCTCAAGGACGGTGTGGCAACGGGAGGCCATGCTGTGTCCCGAGGAAGTGCA 7740
Martinique      CGCCGCGCCCTCAAGGACGGTGTGGCAACGGGAGGCCATGCTGTGTCCCGAGGAAGTGCA 7740
NatalRGN        CGCCGCGCCCTCAAGGATGGTGTGGCAACGGGAGGCCATGCTGTGTCCCGAGGAAGTGCA 7740
HPF2013         CGCCGCGCCCTCAAGGACGGTGTGGCAACGGGAGGCCATGCTGTGTCCCGAGGAAGTGCA 7740
SPH2015         CGCCGCGCCCTCAAGGACGGTGTGGCAACGGGAGGCCATGCTGTGTCCCGAGGAAGTGCA 7740
Haiti2014       CGCCGCGCCCTCAAGGACGGTGTGGCAACGGGAGGCCATGCTGTGTCCCGAGGAAGTGCA 7740
PRVABC59        CGCCGCGCCCTCAAGGACGGTGTGGCAACGGGAGGCCATGCTGTGTCCCGAGGAAGTGCA 7740
BeH819015       CGCCGCGCCCTCAAGGACGGTGTGGCAACGGGAGGCCATGCTGTGTCCCGAGGAAGTGCA 7740
Z1106033        CGCCGCGCCCTCAAGGACGGTGTGGCAACGGGAGGCCATGCTGTGTCCCGAGGAAGTGCA 7740
BEH819966       CGCCGCGCCCTCAAGGACGGTGTGGCAACGGGAGGCCATGCTGTGTCCCGAGGAAGTGCA 7740
SSABr           CGCCGCGCCCTCAAGGACGGTGTGGCAACGGGAGGCCATGCTGTGTCCCGAGGAAGTGCA 7740
Beh815744       CGCCGCGCCCTCAAGGACGGTGTGGCAACGGGAGGCCATGCTGTGTCCCGAGGAAGTGCA 7740
BEH818995       CGCCGCGCCCTCAAGGACGGTGTGGCAACGGGAGGCCATGCTGTGTCCCGAGGAAGTGCA 7740
IbH30656        CGCCGCGCCCTCAAGGATGGAGTGGCCACAGGAGGACATGCTGTATCCCGGGGAAGCGCA 7722
ArD128000       CGCCGCGCTCTCAAGGATGGAGTGGCCACAGGAGGGCATGCTGTATCCCGGGGAAGCGCA 7740
ArD7117         CGCCGCGCCCTCAAGGATGGAGTGGCCACAGGAGGACATGCTGTATCCCGGGGAAGCGCA 7740
ARD_41519       CGCCGCGCCCTCAAGGATGGAGTGGCCACAGGAGGACATGCTGTATCCCGGGGAAGCGCA 7740
ARD157995       CGCCGTGCCCTCAAGGATGGAGTGGCCACAGGAGGACATGCCGTATCCCGGGGAAGTGCA 7740
MR_766          CGCCGTGCCCTCAAGGATGGAGTGGCCACAGGAGGACATGCCGTATCCCGGGGAAGTGCA 7728
ARD158084       CGCCGTGCCCTCAAGGATGGAGTGGCCACAGGAGGACATGCCGTATCCCGGGGAAGTGCA 7740
ArB1362         CGCCGCGCCCTCAAGGATGGAGTGGCCACAGGAGGACATGCTGTATCCCGGGGAAGCGCA 7740
ARB13565        CGCCGCGCCCTCAAGGATGGAGTGGCCACAGGAGGACATGCTGTATCCCGGGGAAGCGCA 7740
ARB7701         CGCCGCGCCCTCAAGGATGGAGTGGCCACAGGAGGACATGCTGTATCCCGGGGAAGCGCA 7740
ARB15076        CGCCGCGCCCTCAAGAATGGAGTGGCCACAGGAGGACATGCTGTATCCCGGGGAAGCGCA 7722
                ***** ** ****** * ** ***** ** ***** ** ** ** ***** ***** **

P6-740          AAGCTTAGATGGTTGGTGGAGAGAGGATACCTGCAGCCCTATGGAAAGGTCATTGATCTT 7800
CPC0740         AAGCTTAGATGGCTGGTGGAGAGAGGATACCTGCAGCCCTATGGAAAGGTCATTGATCTT 7800
YAP2007         AAGCTTAGATGGTTGGTGGAGAGAGGATACCTGCAGCCCTATGGAAAGGTCATTGATCTT 7800
FSS13025        AAGCTGAGATGGTTGGTGGAGAGGGGATACCTGCAGCCCTATGGAAAGGTCATTGATCTT 7800
PLCal_ZV        AAGCTGAGATGGTTGGTGGAGCGGGGATACCTGCAGCCCTATGGAAAGGTCATTGATCTT 7689
SV0127/14       AAGCTGAGATGGTTGGTGGAGCGGGGATACCTGCAGCCCTATGGAAAGGTCATTGATCTT 7800
8375            AAGCTGAGATGGTTGGTGGAGCGGGGATACCTGCAGCCCTATGGAAAGGTCATTGATCTT 7800
103344          AAGCTGAGATGGTTGGTGGAGCGGGGATACCTGCAGCCCTATGGAAAGGTCATTGATCTT 7800
BrasilZKV2015   AAGCTGAGATGGTTGGTGGAGCGGGGATACCTGCAGCCCTATGGAAAGGTCATTGATCTT 7800
GD01            AAGCTGAGATGGTTGGTGGAGCGGGGATACCTGCAGCCCTATGGAAAGGTCATTGATCTT 7800
Martinique      AAGCTGAGATGGTTGGTGGAGCGGGGATACCTGCAGCCCTATGGAAAGGTCATTGATCTT 7800
NatalRGN        AAGCTGAGATGGTTGGTGGAGCGGGGATACCTGCAGCCCTATGGAAAGGTCATTGATCTT 7800
HPF2013         AAGCTGAGATGGTTGGTGGAGCGGGGATACCTGCAGCCCTATGGAAAGGTCATTGATCTT 7800
SPH2015         AAGCTGAGATGGTTGGTGGAGCGGGGATACCTGCAGCCCTATGGAAAGGTCATTGATCTT 7800
Haiti2014       AAGCTGAGATGGTTGGTGGAGCGGGGATACCTGCAGCCCTATGGAAAGGTCATTGATCTT 7800
PRVABC59        AAGCTGAGATGGTTGGTGGAGCGGGGATACCTGCAGCCCTATGGAAAGGTCATTGATCTT 7800
BeH819015       AAGCTGAGATGGTTGGTGGAGCGGGGATACCTGCAGCCCTATGGAAAGGTCATTGATCTT 7800
Z1106033        AAGCTGAGATGGTTGGTGGAGCGGGGATACCTGCAGCCCTATGGAAAGGTCATTGATCTT 7800
BEH819966       AAGCTGAGATGGTTGGTGGAGCGGGGATACCTGCAGCCCTATGGAAAGGTCATTGATCTT 7800
SSABr           AAGCTGAGATGGTTGGTGGAGCGGGGATACCTGCAGCCCTATGGAAAGGTCATTGATCTT 7800
Beh815744       AAGCTGAGATGGTTGGTGGAGCGGGGATACCTGCAGCCCTATGGAAAGGTCATTGATCTT 7800
BEH818995       AAGCTGAGATGGTTGGTGGAGCGGGGATACCTGCAGCCCTATGGAAAGGTCATTGATCTT 7800
IbH30656        AAGCTTAGATGGTTGGTAGAGAGAGGATACCTGCAGCCCCATGGAAAGGTTGTTGACCTC 7782
ArD128000       AAGCTTAGATGGTTGGTAGAGAGAGGATACCTGCAGCCCCATGGAAAGGTTGTTGACCTT 7800
ArD7117         AAGCTTAGATGGTTGGTAGAGAGAGGATACCTGCAGCCCCATGGAAAGGTTGTTGACCTT 7800
ARD_41519       AAGCTTAGATGGTTGGTAGAGAGAGGATACCTGCAGCCCCATGGAAAGGTTGTTGACCTT 7800
ARD157995       AAGCTCAGATGGTTGGTGGAGAGAGGATATCTGCAGCCCCATGGAAGGTTGTTGACCTC 7800
MR_766          AAGATCAGATGGTTGGAGGAGAGAGGATATCTGCAGCCCTATGGGAAGGTTGTTGACCTC 7788
ARD158084       AAGCTCAGATGGTTGGTGGAGAGAGGATATCTGCAGCCCCATGGGAAGGTTGTTGACCTC 7800
ArB1362         AAGCTCAGATGGCTGGTGGAGAGAGGATACCTGCAGCCCCATGGAAAGGTTGTTGACCTC 7800
ARB13565        AAGCTCAGATGGCTGGTGGAGAGAGGATACCTGCAACCCCATGGAAAGGTTGTTGACCTC 7800
ARB7701         AAGCTCAGATGGCTGGTGGAGAGAGGATACCTGCAACCCCATGGAAAGGTTGTTGACCTC 7800
ARB15076        AAGCTCAGATGGCTGGTGGAGAGAGGATACCTGCAACCCCATGGAAAGGTTGTTGACCTC 7782
                *** * ****** ***  *** * ***** ***** *** **** *****  **** **

P6-740          GGATGTGGCAGAGGGGGCTGGAGTTACTACGCCGCCACCATCCGCAAAGTTCAAGAGGTG 7860
```

```
CPC0740          GGATGTGGCAGAGGGGGCTGGAGTTACTATGCCGCCACCATCCGCAAAGTTCAGGAAGTG 7860
YAP2007          GGATGTGGCAGAGGGGGCTGGAGTTACTACGCCGCCACCATCCGCAAAGTTCAAGAAGTG 7860
FSS13025         GGATGTGGCAGAGGGGGCTGGAGTTACTATGCCGCCACCATCCGCAAAGTTCAAGAAGTG 7860
PLCal_ZV         GGATGTGGCAGAGGGGGCTGGAGTTACTACGCCGCCACCATCCGCAAAGTTCAAGAGGTG 7749
SV0127/14        GGATGTGGCAGAGGGGGCTGGAGTTACTACGCCGCCACCATCCGCAAAGTTCAAGAAGTG 7860
8375             GGATGTGGCAGAGGGGGCTGGAGTTACTACGCCGCCACCATCCGCAAAGTTCAAGAAGTG 7860
103344           GGATGTGGCAGAGGGGGCTGGAGTTACTACGCCGCCACCATCCGCAAAGTTCAAGAAGTG 7860
BrasilZKV2015    GGATGTGGCAGAGGGGGCTGGAGTTACTACGCCGCCACCATCCGCAAAGTTCAAGAAGTG 7860
GD01             GGATGTGGCAGAGGGGGCTGGAGTTACTACGCCGCCACCATCCGCAAAGTTCAAGAAGTG 7860
Martinique       GGATGTGGCAGAGGGGGCTGGAGTTACTACGCCGCCACCATCCGCAAAGTTCAAGAAGTG 7860
NatalRGN         GGATGTGGCAGAGGGGGCTGGAGTTACTACGCCGCCACCATCCGCAAAGTTCAAGAAGTG 7860
HPF2013          GGATGTGGCAGAGGGGGCTGGAGTTACTACGCCGCCACCATCCGCAAAGTTCAAGAAGTG 7860
SPH2015          GGATGTGGCAGAGGGGGCTGGAGTTACTACGCCGCCACCATCCGCAAAGTTCAAGAAGTG 7860
Haiti2014        GGATGTGGCAGAGGGGGCTGGAGTTACTACGCCGCCACCATCCGCAAAGTTCAAGAAGTG 7860
PRVABC59         GGATGTGGCAGAGGGGGCTGGAGTTACTACGTCGCCACCATCCGCAAAGTTCAAGAAGTG 7860
BeH819015        GGATGTGGCAGAGGGGGCTGGAGTTACTACGCCGCCACCATCCGCAAAGTTCAAGAAGTG 7860
Z1106033         GGATGTGGCAGAGGGGGCTGGAGTTACTACGCCGCCACCATCCGCAAAGTTCAAGAAGTG 7860
BEH819966        GGATGTGGCAGAGGGGGCTGGAGTTACTACGCCGCCACCATCCGCAAAGTTCAAGAAGTG 7860
SSABr            GGATGTGGCAGAGGGGGCTGGAGTTACTACGCCGCCACCATCCGCAAAGTTCAAGAAGTG 7860
Beh815744        GGATGTGGCAGAGGGGGCTGGAGTTACTACGCCGCCACCATCCGCAAAGTTCAAGAAGTG 7860
BEH818995        GGATGTGGCAGAGGGGGCTGGAGTTACTACGCCGCCACCATCCGCAAAGTTCAAGAAGTG 7860
IbH30656         GGATGTGGCAGAGGGGGCTGGAGTTATTACGCTGCCACCATCCGTAAAGTGCAGGAGGTC 7842
ArD128000        GGATGTGGCAGAGGAGGCTGGAGTTATTACGCTGCCACTATCCGTAAAGTGCAGGAGGTC 7860
ArD7117          GGATGTGGCAGAGGAGGCTGGAGTTATTACGCTGCCACCATCCGTAAAGTGCAGGAGGTC 7860
ARD_41519        GGATGTGGCAGAGGAGGCTGGAGTTATTACGCTGCCACCATCCGTAAAGTGCAGGAGGTC 7860
ARD157995        GGATGTGGCAGAGGGGGCTGGAGCTATTATGCCGCCACCATCCGCAAAGTGCAGGAGGTG 7860
MR_766           GGATGTGGCAGAGGGGGCTGGAGCTATTATGCCGCCACCATCCGCAAAGTGCAGGAGGTG 7848
ARD158084        GGATGTGGCAGAGGGGGCTGGAGCTATTATGCCGCCACCATCCGCAAAGTGCAGGAGGTG 7860
ArB1362          GGATGTGGCAGAGGGGGTTGGAGCTATTATGCCGCTACCATCCGGAAAGTGCAGGAGGTG 7860
ARB13565         GGATGTGGCAGAGGGGGTTGGAGCTATTATGCCGCCACCATCCGGAAAGTGCAGGAGGTG 7860
ARB7701          GGATGTGGCAGAGGGGGTTGGAGCTATTATGCCGCCACCATCCGGAAAGTGCAGGAGGTG 7860
ARB15076         GGATGTGGCAGAGGGGGTTGGAGCTATTATGCCGCCACCATCCGGAAAGTGCAGGAGGTG 7842
                 ************** ** ***** ** ** *  ** ** ***** ***** ** ** **

P6-740           AAAGGATACACAAAGGGAGGCCCTGGTCATGAAGAACCCACGTTGGTGCAAAGCTATGGA 7920
CPC0740          AAAGGATACACAAAAGGAGGCCCTGGTCATGAAGAACCCATGTTGGTGCAAAGCTATGGG 7920
YAP2007          AAAGGATACACAAAAGGAGGCCCTGGTCATGAAGAACCCATGTTGGTGCAAAGCTATGGG 7920
FSS13025         AAAGGATACACAAAAGGAGGCCCTGGTCATGAAGAACCCATGTTGGTGCAAAGCTATGGG 7920
PLCal_ZV         AAAGGATACACAAAAGGAGGCCCTGGTCATGAAGAACCCATGTTGGTGCAAAGCTATGGG 7809
SV0127/14        AAAGGATACACAAAAGGAGGCCCTGGTCATGAAGAACCCATGTTGGTGCAAAGCTATGGG 7920
8375             AAAGGATACACAAAAGGAGGCCCTGGTCATGAAGAACCCGTGTTGGTGCAAAGCTATGGG 7920
103344           AAAGGATACACAAAAGGAGGCCCTGGTCATGAAGAACCCGTGTTGGTGCAAAGCTATGGG 7920
BrasilZKV2015    AAAGGATACACAAAAGGAGGCCCTGGTCATGAAGAACCCGTGTTGGTGCAAAGCTATGGG 7920
GD01             AAAGGATACACAAAAGGAGGCCCTGGTCATGAAGAACCCGTGTTGGTGCAAAGCTATGGG 7920
Martinique       AAAGGATACACAAAAGGAGGCCCTGGTCATGAAGAACCCGTGTTGGTGCAAAGCTATGGG 7920
NatalRGN         AAAGGATACACAAAAGGAGGCCCTGGTCATGAAGAACCCGTGTTGGTGCAAAGCTATGGG 7920
HPF2013          AAAGGATACACAAAAGGAGGCCCTGGTCATGAAGAACCCATGTTGGTGCAAAGCTATGGG 7920
SPH2015          AAAGGATACACAAAAGGAGGCCCTGGTCATGAAGAACCCGTGTTGGTGCAAAGCTATGGG 7920
Haiti2014        AAAGGATACACAAAAGGAGGCCCTGGTCATGAAGAACCCGTGTTGGTGCAAAGCTATGGG 7920
PRVABC59         AAAGGATACACAAAAGGAGGCCCTGGTCATGAAGAACCCGTGTTGGTGCAAAGCTATGGG 7920
BeH819015        AAAGGATACACAAAAGGAGGCCCTGGTCATGAAGAACCCGTGTTGGTGCAAAGCTATGGG 7920
Z1106033         AAAGGATACACAAAAGGAGGCCCTGGTCATGAAGAACCCGTGTTGGTGCAAAGCTATGGG 7920
BEH819966        AAAGGATACACAAAAGGAGGCCCTGGTCATGAAGAACCCGTGTTGGTGCAAAGCTATGGG 7920
SSABr            AAAGGATACACAAAAGGAGGCCCTGGTCATGAAGAACCCGTGTTGGTGCAAAGCTATGGG 7920
Beh815744        AAAGGATACACAAAAGGAGGCCCTGGTCATGAAGAACCCGTGTTGGTGCAAAGCTATGGG 7920
BEH818995        AAAGGATACACAAAAGGAGGCCCTGGTCATGAAGAACCCGTGTTGGTGCAAAGCTATGGG 7920
IbH30656         AGAGGATACACAAAGGGAGGTCCTGGTCATGAAGAACCCATGCTGGTGCAAAGCTATGGG 7902
ArD128000        AGAGGATACACAAAGGGAGGTCCTGGTCATGAAGAACCCATGCTGGTGCAAAGCTATGGG 7920
ArD7117          AGAGGATACACAAAGGGAGGTCCTGGTCATGAAGAACCCATGCTGGTGCAAAGCTATGGG 7920
ARD_41519        AGAGGATACACAAAGGGAGGTCCTGGCCATGAAGAGCCCATGCTGGTGCAAAGCTATGGG 7920
ARD157995        AGAGGATACACAAAGGGAGGTCCCGGTCATGAAGAACCCATGCTGGTGCAAAGCTATGGG 7920
MR_766           AGAGGATACACAAAGGGAGGTCCCGGTCATGAAGAACCCATGCTGGTGCAAAGCTATGGG 7908
ARD158084        AGAGGATACACAAAGGGAGGTCCCGGTCATGAAGAACCCATGCTGGTGCAAAGCTATGGG 7920
ArB1362          AAAGGATACACAAAGGGAGGTCCCGGTCATGAAGAACCCATGCTGGTGCAAAGCTATGGA 7920
ARB13565         AAAGGATACACAAAGGGAGGTCCCGGTCATGAAGAACCCATGCTGGTGCAAAGCTATGGG 7920
ARB7701          AAAGGATACACAAAGGGAGGTCCCGGTCATGAAGAACCCATGCTGGTGCAAAGCTATGGG 7920
ARB15076         AAAGGATACACAAAGGGAGGTCCTGGTCATGAAGAACCCATGCTGGTGCAAAGCTATGGG 7902
                 * ************ ***** ** ** ******** ***  * ****************
```

```
P6-740          TGGAACATAGTCCGTCTTAAGAGTGGGGTGGACGTCTTTCACATGGCGGCGGAGTCGTGT 7980
CPC0740         TGGAACATAGTCCGTCTTAAGAGTGGGGTGGACGTCTTTCACATGGCGGCTGAGCCGTGT 7980
YAP2007         TGGAACATAGTCCGTCTTAAGAGTGGGGTGGACGTCTTTCATATGGCGGCTGAGCCGTGT 7980
FSS13025        TGGAACATAGTCCGTCTTAAGAGTGGGGTGGACGTCTTTCATATGGCGGCTGAGCCGTGT 7980
PLCal_ZV        TGGAACATAGTCCGTCTTAAGAGTGGGGTGGACGTCTTTCATATGGCGGCTGAGCCGTGT 7869
SV0127/14       TGGAACATAGTCCGTCTTAAGAGTGGGGTGGACGTCTTTCATATGGCGGCTGAGCCGTGT 7980
8375            TGGAACATAGTCCGTCTTAAGAGTGGGGTGGACGTCTTTCATATGGCGGCTGAGCCGTGT 7980
103344          TGGAACATAGTCCGTCTTAAGAGTGGGGTGGACGTCTTTCATATGGCGGCTGAGCCGTGT 7980
BrasilZKV2015   TGGAACATAGTCCGTCTTAAGAGTGGGGTGGACGTCTTTCATATGGCGGCTGAGCCGTGT 7980
GD01            TGGAACATAGTCCGTCTTAAGAGTGGGGTGGACGTCTTTCATATGGCGGCTGAGCCGTGT 7980
Martinique      TGGAACATAGTCCGTCTTAAGAGTGGGGTGGACGTCTTTCATATGGCGGCTGAGCCGTGT 7980
NatalRGN        TGGAACATAGTCCGTCTTAAGAGTGGGGTGGACGTCTTTCATATGGCGGCTGAGCCGTGT 7980
HPF2013         TGGAACATAGTCCGTCTTAAGAGTGGGGTGGACGTCTTTCATATGGCGGCTGAGCCGTGT 7980
SPH2015         TGGAACATAGTCCGTCTTAAGAGTGGGGTGGACGTCTTTCATATGGCGGCTGAGCCGTGT 7980
Haiti2014       TGGAACATAGTCCGTCTTAAGAGTGGGGTGGACGTCTTTCATATGGCGGCTGAGCCGTGT 7980
PRVABC59        TGGAACATAGTCCGTCTTAAGAGTGGGGTGGACGTCTTTCATATGGCGGCTGAGCCGTGT 7980
BeH819015       TGGAACATAGTCCGTCTTAAGAGTGGGGTGGACGTCTTTCATATGGCGGCTGAGCCGTGT 7980
Z1106033        TGGAACATAGTCCGTCTTAAGAGTGGGGTGGACGTCTTTCATATGGCGGCTGAGCCGTGT 7980
BEH819966       TGGAACATAGTCCGTCTTAAGAGTGGGGTGGACGTCTTTCATATGGCGGCTGAGCCGTGT 7980
SSABr           TGGAACATAGTCCGTCTTAAGAGTGGGGTGGACGTCTTTCATATGGCGGCTGAGCCGTGT 7980
Beh815744       TGGAACATAGTCCGTCTTAAGAGTGGGGTGGACGTCTTTCATATGGCGGCTGAGCCGTGT 7980
BEH818995       TGGAACATAGTCCGTCTTAAGAGTGGGGTGGACGTCTTTCATATGGCGGCTGAGCCGTGT 7980
IbH30656        TGGAACATAGTTCGCCTCAAGAGTGGAGTGGACGTCTTTCACATGGCGGCTGAGCCGTGT 7962
ArD128000       TGGAACATAGTTCGCCTCAAGAGTGGAGTGGACGTCTTCCACATGGCGGCTGAGCCGTGT 7980
ArD7117         TGGAACATAGTTCGCCTCAAGAGTGGAGTGGACGTCTTCCACATGGCGGCTGAGCCGTGT 7980
ARD_41519       TGGAACATAGTTCGCCTCAAGAGTGGAGTGGACGTCTTCCACATGGCGGCTGAACCGTGT 7980
ARD157995       TGGAACATAGTTCGTCTCAAGAGTGGAGTGGACGTCTTCCACATGGCGGCTGAGCCGTGT 7980
MR_766          TGGAACATAGTTCGTCTCAAGAGTGGAGTGGACGTCTTCCACATGGCGGCTGAGCCGTGT 7968
ARD158084       TGGAACATAGTTCGTCTCAAGAGTGGAGTGGACGTCTTCCACATGGCGGCTGAGCCGTGT 7980
ArB1362         TGGAACATAGTTCGTCTCAAGAGTGGAGTGGACGTCTTCCACATGGCGGCTGAGCCGTGT 7980
ARB13565        TGGAACATCGTTCGTCTCAAGAGTGGAGTGGACGTCTTCCACATGGCGGCCGAGCCGTGT 7980
ARB7701         TGGAACATCGTTCGTCTCAAGAGTGGAGTGGACGTCTTCCACATGGCGGCCGAGCCGTGT 7980
ARB15076        TGGAACATAGTTCGTCTCAAGAGTGGAGTGGATGTCTTCCACATGGCGGCCGAGCCGTGT 7962
                ******** ** ** ** ******** ***** ***** ** ******** **  *****

P6-740          GACACTTTGCTGTGTGACATAGGTGAGTCATCATCTAGTCCTGAAGTGGAAGAAGCACGG 8040
CPC0740         GACACTTTGCTGTGTGATATAGGTGAGTCATCATCTAGTCCTGAAGTGGAAGAAGCACGG 8040
YAP2007         GACACTTTGCTGTGTGATATAGGTGAGTCATCATCTAGTCCTGAAGTGGAAGAAGCACGG 8040
FSS13025        GACACGTTGCTGTGTGATATAGGTGAGTCATCATCTAGTCCTGAAGTGGAAGAAGCACGG 8040
PLCal_ZV        GACACGTTGCTGTGTGATATAGGTGAGTCATCATCTAGTCCTGAAGTGGAAGAAGCACGG 7929
SV0127/14       GACACGTTGCTGTGTGACATAGGTGAGTCATCATCTAGTCCTGAAGTGGAAGAAGCACGG 8040
8375            GACACGTTGCTGTGTGACATAGGCGAGTCATCATCTAGTCCTGAAGTGGAAGAAGCACGG 8040
103344          GACACGTTGCTGTGTGACATAGGCGAGTCATCATCTAGTCCTGAAGTGGAAGAAGCACGG 8040
BrasilZKV2015   GACACGTTGCTGTGTGACATAGGTGAGTCATCATCTAGTCCTGAAGTGGAAGAAGCACGG 8040
GD01            GACACGTTGCTGTGTGACATAGGTGAGTCATCATCTAGTCCTGAAGTGGAAGAAGCACGG 8040
Martinique      GACACGTTGCTGTGTGACATAGGTGAGTCATCATCTAGTCCTGAAGTGGAAGAAGCACGG 8040
NatalRGN        GACACGTTGCTGTGTGACATAGGTGAGTCATCATCTAGTCCTGAAGTGGAAGAAGCACGG 8040
HPF2013         GACACGTTGCTGTGTGACATAGGTGAGTCATCATCTAGTCCTGAAGTGGAAGAAGCACGG 8040
SPH2015         GACACGTTGCTGTGTGACATAGGTGAGTCATCATCTAGTCCTGAAGTGGAAGAAGCACGG 8040
Haiti2014       GACACGTTGCTGTGTGACATAGGTGAGTCATCATCTAGTCCTGAAGTGGAAGAAGCACGG 8040
PRVABC59        GACACGTTGCTGTGTGACATAGGTGAGTCATCATCTAGTCCTGAAGTGGAAGAAGCACGG 8040
BeH819015       GACACGCTGCTGTGTGACATAGGTGAGTCATCATCTAGTCCTGAAGTGGAAGAAGCACGG 8040
Z1106033        GACACGTTGCTGTGTGACATAGGTGAGTCATCATCTAGTCCTGAAGTGGAAGAAGCACGG 8040
BEH819966       GACACGTTGCTGTGTGACATAGGTGAGTCATCATCTAGTCCTGAAGTGGAAGAAGCACGG 8040
SSABr           GACACGTTGCTGTGTGACATAGGTGAGTCATCATCTAGTCCTGAAGTGGAAGAAGCACGG 8040
Beh815744       GACACGTTGCTGTGTGACATAGGTGAGTCATCATCTAGTCCTGAAGTGGAAGAAGCACGG 8040
BEH818995       GACACGTTGCTGTGTGACATAGGTGAGTCATCATCTAGTCCTGAAGTGGAAGAAGCACGG 8040
IbH30656        GACACTTTGCTGTGTGACATTGGCGAGTCATCGTCCAGTCCTGAAGTGGAAGAGACGCGA 8022
ArD128000       GACACTCTGCTGTGTGACATAGGCGAGTCATCATCTAGTCCTGAAGTGGAAGAGACGCGA 8040
ArD7117         GACACTCTGCTGTGTGACATCGGCGAGTCATCATCTAGTCCTGAAGTGGAAGAGACGCGA 8040
ARD_41519       GACACTCTGCTGTGTGACATAGGCGAGTCATCATCCAGTCCTGAAGTGGAAGAGACGCGA 8040
ARD157995       GACACTCTGCTGTGTGACATAGGTGAGTCATCATCTAGTCCTGAAGTGGAAGAGACACGA 8040
MR_766          GACACTCTGCTGTGTGACATAGGTGAGTCATCATCTAGTCCTGAAGTGGAAGAGACACGA 8028
ARD158084       GACACTCTGCTGTGTGACATAGGTGAGTCATCATCTAGTCCTGAAGTGGAAGAGACACGA 8040
ArB1362         GATACTCTGCTGTGTGACATAGGTGAGTCATCATCCAGTCCTGAAGTAGAAGAGACGCGA 8040
ARB13565        GATACCTTGCTGTGTGACATAGGTGAGTCATCATCTAGTCCTGAAGTGGAAGAGACGCGA 8040
ARB7701         GATACCTTGCTGTGTGACATAGGTGAGTCATCATCTAGTCCTGAAGTGGAAGAGACGCGA 8040
```

```
ARB15076            GACACCTTGCTGTGCGACATAGGTGAGTCATCATCTAGTCCTGAAGTGGAAGAGACGCGA 8022
                    ** **  ******* ** ** ** ******** ** *********** *****  * **

P6-740              ACGCTCAGAGTACTCTCCATGGTGGGGGATTGGCTTGAAAAAAGACCAGGGGCCTTTTGT 8100
CPC0740             ACGCTCAGAGTCCTCTCCATGGTGGGGGATTGGCTTGAAAAAAGACCAGGAGCCTTTTGT 8100
YAP2007             ACGCTCAGAGTCCTTTCCATGGTGGGGGATTGGCTTGAAAAAAGACCAGGAGCCTTTTGT 8100
FSS13025            ACGCTCAGAGTCCTCTCCATGGTGGGGGATTGGCTTGAAAAAAGACCAGGAGCCTTTTGT 8100
PLCal_ZV            ACGCTCAGAGTCCTCTCCATGGTGGGGGATTGGCTTGAAAAAAGACCAGGAGCCTTTTGT 7989
SV0127/14           ACGCTCAGAGTCCTCTCCATGGTGGGGGATTGGCTTGAAAAAAGACCAGGAGCCTTTTGT 8100
8375                ACGCTCAGAGTCCTCTCCATGGTGGGGGATTGGCTTGAAAGAAGACCAGGAGCCTTTTGT 8100
103344              ACGCTCAGAGTCCTCTCCATGGTGGGGGATTGGCTTGAAAGAAGACCAGGAGCCTTTTGT 8100
BrasilZKV2015       ACGCTCAGAGTCCTCTCCATGGTGGGGGATTGGCTTGAAAAAAGACCAGGAGCCTTTTGC 8100
GD01                ACGCTCAGAGTCCTCTCCATGGTGGGGGATTGGCTTGAAAAAAGACCAGGAGCCTTTTGT 8100
Martinique          ACGCTCAGAGTCCTCTCCATGGTGGGGGATTGGCTTGAAAAAAGACCAGGAGCCTTTTGT 8100
NatalRGN            ACGCTCAGAGTCCTCTCCATGGTGGGGGATTGGCTTGAAAAAAGACCAGGAGCCTTTTGT 8100
HPF2013             ACGCTCAGAGTCCTCTCCATGGTGGGGGATTGGCTTGAAAAAAGACCAGGAGCCTTTTGT 8100
SPH2015             ACGCTCAGAGTCCTCTCCATGGTGGGGGATTGGCTTGAAAAAAGACCAGGAGCCTTTTGT 8100
Haiti2014           ACGCTCAGAGTCCTCTCCATGGTGGGGGATTGGCTTGAAAAAAGACCAGGAGCCTTTTGT 8100
PRVABC59            ACGCTCAGAGTCCTCTCCATGGTGGGGGATTGGCTTGAAAAAAGACCAGGAGCCTTTTGT 8100
BeH819015           ACGCTCAGAGTCCTCTCCATGGTGGGGGATTGGCTTGAAAAAAGACCAGGAGCCTTTTGT 8100
Z1106033            ACGCTCAGAGTCCTCTCCATGGTGGGGGATTGGCTTGAAAAAAGACCAGGAGCCTTTTGT 8100
BEH819966           ACGCTCAGAGTCCTCTCCATGGTGGGGGATTGGCTTGAAAAAAGACCAGGAGCCTTTTGT 8100
SSABr               ACGCTCAGAGTCCTCTCCATGGTGGGGGATTGGCTTGAAAAAAGACCAGGAGCCTTTTGT 8100
Beh815744           ACGCTCAGAGTCCTCTCCATGGTGGGGGATTGGCTTGAAAAAAGACCAGGAGCCTTTTGT 8100
BEH818995           ACGCTCAGAGTCCTCTCCATGGTGGGGGATTGGCTTGAAAAAAGACCAGGAGCCTTTTGT 8100
IbH30656            ACACTCAGAGTGCTCTCCATGGTGGGAGACTGGCTCGAGAAAAGACCAGGGGCCTTCTGC 8082
ArD128000           ACACTCAGAGTGCTCTCCATGGTAGAAGACTGGCTTGAGAAAAGACCAGGGGCCTTCTGC 8100
ArD7117             ACACTCAGAGTGCTCTCCATGGTGGGAGACTGGCTTGAGAAAAGACCAGGGGCCTTCTGC 8100
ARD_41519           ACACTCAGAGTGCTCTCCATGGTGGGAGACTGGCTTGAAAAAAGACCAGGGGCCTTCTGC 8100
ARD157995           ACACTCAGAGTGCTCTCTATGGTGGGGGACTGGCTTGAAAAAAGACCAGGGGCCTTCTGT 8100
MR_766              ACACTCAGAGTGCTCTCTATGGTGGGGGACTGGCTTGAAAAAAGACCAGGGGCCTTCTGT 8088
ARD158084           ACACTCAGAGTGCTCTCTATGGTGGGGGACTGGCTTGAAAAAAGACCAGGGGCCTTCTGT 8100
ArB1362             ACACTCAGAGTGCTCTCTATGGTGGGGGACTGGCTTGAAAAAAGACCAGGGGCCTTCTGC 8100
ARB13565            ACACTCCGAGTGCTCTCTATGGTGGGGGACTGGCTTGAGAAAAGACCGGGGGCCTTCTGT 8100
ARB7701             ACACTCCGAGTGCTCTCTATGGTGGGGGACTGGCTTGAGAAAAGACCGGGGGCCTTCTGT 8100
ARB15076            ACACTCAGAGTGCTCTCTATGGTGGGGGACTGGCTTGAGAAAAGACCGGGAGCCTTCTGC 8082
                    ** *** **** ** ** ***** *  ** ***** ** * ****** ** ***** **

P6-740              ATAAAGGTGTTGTGCCCATACACCAGCACCATGATGGAAACCCTAGAGCGACTGCAGCGT 8160
CPC0740             ATAAAAGTGTTGTGCCCATACACCAGCACTATGATGGAAACCCTGGAGCGACTGCAGCGT 8160
YAP2007             ATAAAAGTGTTGTGCCCATACACCAGCACTATGATGGAAACCCTGGAGCGACTGCAGCGT 8160
FSS13025            ATAAAAGTGTTGTGCCCATACACCAGCACTATGATGGAAACCCTGGAGCGACTGCAGCGT 8160
PLCal_ZV            ATAAAAGTGTTGTGCCCATACACCAGCACTATGATGGAAACCCTGGAGCGACTGCAGCGT 8049
SV0127/14           GTAAAAGTGTTGTGCCCATACACCAGCACTATGATGGAAACCCTGGAGCGACTGCAGCGT 8160
8375                ATAAAAGTGTTGTGCCCATACACCAGCACTATGATGGAAACCCTGGAGCGACTGCAGCGT 8160
103344              ATAAAAGTGTTGTGCCCATACACCAGCACTATGATGGAAACCCTGGAGCGACTGCAGCGT 8160
BrasilZKV2015       ATAAAAGTGTTGTGCCCATACACCAGCACTATGATGGAAACCCTGGAGCGACTGCAGCGT 8160
GD01                ATAAAGGTGTTGTGCCCATACACCAGCACTATGATGGAAACCCTGGAGCGACTGCAGCGT 8160
Martinique          ATAAAAGTGTTGTGCCCATACACCAGCACTATGATGGAAACCCTGGAGCGACTGCAGCGT 8160
NatalRGN            ATAAAAGTGTTGTGCCCATACACCAGCACTATGATGGAAACCCTGGAGCGACTGCAGCGT 8160
HPF2013             ATAAAAGTGTTGTGCCCATACACCAGCACTATGATGGAAACCCTGGAGCGACTGCAGCGT 8160
SPH2015             ATAAAAGTGTTGTGCCCATACACCAGCACTATGATGGAAACCCTGGAGCGACTGCAGCGT 8160
Haiti2014           ATAAAAGTGTTGTGCCCATACACCAGCACTATGATGGAAACCCTGGAGCGACTGCAGCGT 8160
PRVABC59            ATAAAAGTGTTGTGCCCATACACCAGCACTATGATGGAAACCCTGGAGCGACTGCAGCGT 8160
BeH819015           ATAAAAGTGTTGTGCCCATACACCAGCACTATGATGGAAACCCTGGAGCGACTGCAGCGT 8160
Z1106033            ATAAAAGTGTTGTGCCCATACACCAGCACTATGATGGAAACCCTGGAGCGACTGCAGCGT 8160
BEH819966           ATAAAGGTGTTGTGCCCATACACCAGCACTATGATGGAAACCCTGGAGCGACTGCAGCGT 8160
SSABr               ATAAAGGTGTTGTGCCCATACACCAGCACTATGATGGAAACCCTGGAGCGACTGCAGCGT 8160
Beh815744           ATAAAGGTGTTGTGCCCATACACCAGCACTATGATGGAAACCCTGGAGCGACTGCAGCGT 8160
BEH818995           ATAAAGGTGTTGTGCCCATACACCAGCACTATGATGGAAACCCTGGAGCGACTGCAGCGT 8160
IbH30656            ATAAAGGTGCTGTGCCCATACACCAGTACTATGATGGAGACCATGGAGCGACTGCAACGT 8142
ArD128000           ATAAAGGTGCTGTGCCCATACACCAGCACAATGATGGAGACCATGGAGCGACTGCAACGT 8160
ArD7117             ATAAAGGTGCTGTGCCCATACACCAGCACAATGATGGAGACCATGGAGCGACTGCAACGT 8160
ARD_41519           ATAAAGGTGCTGTGCCCATACACCAGCACAATGATGGAGACCATGGAGCGACTGCAACGT 8160
ARD157995           ATAAAGGTGCTGTGCCCATACACCAGCACTATGATGGAAACCATGGAGCGACTGCAACGT 8160
MR_766              ATAAAGGTGCTGTGCCCATACACCAGCACTATGATGGAAACCATGGAGCGACTGCAACGT 8148
ARD158084           ATAAAGGTGCTGTGCCCATACACCAGCACTATGATGGAAACCATGGAGCGACTGCAACGT 8160
ArB1362             ATAAAGGTGCTGTGCCCATACACCAGCACTATGATGGAGACCATGGAGCGACTGCAACGT 8160
```

```
ARB13565         ATAAAGGTGCTGTGTCCATACACCAGCACTATGATGGAGACCATGGAGCGCCTGCAACGT 8160
ARB7701          ATAAAGGTGCTGTGTCCATACACCAGCACTATGATGGAGACCATGGAGCGCCTGCAACGT 8160
ARB15076         ATAAAGGTGCTGTGCCCATACACCAGCACTATGATGGAGACCATGGAGCGCCTGCAACGT 8142
                  **** *** **** *********** ** ******** *** * ***** ***** ***

P6-740           AGGTATGGGGGAGGACTGGTCAGAGTGCCACTCTCCCGCAACTCTACACATGAGATGTAC 8220
CPC0740          AGGTATGGGGGAGGACTGGTCAGGGTGCCACTCTCCCGCAACTCTACACATGAGATGTAC 8220
YAP2007          AGGTATGGGGGAGGACTGGTCAGAGTGCCACTCTCCCGCAACTCTACACATGAGATGTAC 8220
FSS13025         AGGTATGGGGGAGGACTGGTCAGAGTGCCACTCTCCCGCAACTCTACACATGAGATGTAC 8220
PLCal_ZV         AGGTATGGGGGAGGACTGGTCAGAGTGCCACTCTCCCGCAACTCTACACATGAGATGTAC 8109
SV0127/14        AGGTATGGGGGAGGACTGGTCAGAGTGCCACTCTCCCGCAACTCTACACATGAGATGTAC 8220
8375             AGGTATGGGGGAGGACTGGTCAGAGTGCCACTCTCCCGCAACTCTACACATGAGATGTAC 8220
103344           AGGTATGGGGGAGGACTGGTCAGAGTGCCACTCTCCCGCAACTCTACACATGAGATGTAC 8220
BrasilZKV2015    AGGTATGGGGGAGGACTGGTCAGAGTGCCACTCTCCCGCAACTCTACACATGAGATGTAC 8220
GD01             AGGTATGGGGGAGGACTGGTCAGAGTGCCACTCTCCCGCAACTCTACACATGAGATGTAC 8220
Martinique       AGGTATGGGGGAGGACTGGTCAGAGTGCCACTCTCCCGCAACTCTACACATGAGATGTAC 8220
NatalRGN         AGGTATGGGGGAGGACTGGTCAGAGTGCCACTCTCCCGCAACTCTACACATGAGATGTAC 8220
HPF2013          AGGTATGGGGGAGGACTGGTCAGAGTGCCACTCTCCCGCAACTCTACACATGAGATGTAC 8220
SPH2015          AGGTATGGGGGAGGACTGGTCAGAGTGCCACTCTCCCGCAACTCTACACATGAGATGTAC 8220
Haiti2014        AGGTATGGGGGAGGACTGGTCAGAGTGCCACTCTCCCGCAACTCTACACATGAGATGTAC 8220
PRVABC59         AGGTATGGGGGAGGACTGGTCAGAGTGCCACTCTCCCGCAACTCTACACATGAGATGTAC 8220
BeH819015        AGGTATGGGGGAGGACTGGTCAGAGTGCCACTCTCCCGCAACTCTACACATGAGATGTAC 8220
Z1106033         AGGTATGGGGGAGGACTGGTCAGAGTGCCACTCTCCCGCAACTCTACACATGAGATGTAC 8220
BEH819966        AGGTATGGGGGAGGACTGGTCAGAGTGCCACTCTCCCGCAACTCTACACATGAGATGTAT 8220
SSABr            AGGTATGGGGGAGGACTGGTCAGAGTGCCACTCTCCCGCAACTCTACACATGAGATGTAT 8220
Beh815744        AGGTATGGGGGAGGACTGGTCAGAGTGCCACTCTCCCGCAACTCTACACATGAGATGTAT 8220
BEH818995        AGGTATGGGGGAGGACTGGTCAGAGTGCCACTCTCCCGCAACTCTACACATGAGATGTAT 8220
IbH30656         AGGTATGGGGGAGGATTGGTCAGAGTGCCATTGTCCCGCAACTCCACACATGAGATGTAT 8202
ArD128000        AGGCATGGGGGAGGATTGGTCAGAGTGCCATTGTCCCGCAACTCCACACATGAGATGTAT 8220
ArD7117          AGGCATGGGGGAGGATTGGTCAGAGTGCCATTGTCCCGCAACTCCACACATGAGATGTAT 8220
ARD_41519        AGGCATGGGGGAGGATTAGTCAGAGTGCCATTGTCCCGCAACTCTACACATGAGATGTAT 8220
ARD157995        AGGCATGGGGGAGGATTAGTCAGAGTGCCATTGTCTCGCAACTCCACACATGAGATGTAC 8220
MR_766           AGGCATGGGGGAGGATTAGTCAGAGTGCCATTGTGTCGCAACTCCACACATGAGATGTAC 8208
ARD158084        AGGCATGGGGGAGGATTAGTCAGAGTGCCATTGTCTCGCAACTCCACACATGAGATGTAC 8220
ArB1362          AGGTATGGGGGAGGATTAGTCAGAGTGCCATTGTCTCGCAACTCCACACATGAGATGTAT 8220
ARB13565         AGGTATGGGGGAGGATTAGTCAGAGTGCCATTGTCTCGCAACTCCACACATGAGATGTAT 8220
ARB7701          AGGTATGGGGGAGGATTAGTCAGAGTGCCATTGTCTCGCAACTCCACACATGAGATGTAT 8220
ARB15076         AGGTATGGGGGAGGATTAGTCAGAGTGCCATTGTCTCGCAACTCCACACATGAAATGTAT 8202
                 *** *********** * ***** ****** * *  ******** ******** *****

P6-740           TGGGTCTCTGGAGCGAAAAGCAACATCATAAAAAGTGTGTCCACCACGAGCCAGCTCCTC 8280
CPC0740          TGGGTCTCTGGAGCGAAAAGCAACACCATAAAAAGTGTGTCCACCACGAGCCAGCTCCTC 8280
YAP2007          TGGGTCTCTGGAGCGAAAAGCAACACCATAAAAAGTGTGTCCACCACGAGCCAGCTCCTC 8280
FSS13025         TGGGTCTCTGGAGCGAAAAGCAACACCATAAAAAGTGTGTCCACCACGAGCCAGCTCCTT 8280
PLCal_ZV         TGGGTCTCTGGAGCGAAAAGCAACACCATAAAAAGTGTGTCCACCACGAGCCAGCTCCTC 8169
SV0127/14        TGGGTCTCTGGAGCGAAAAGCAACACCATAAAAAGTGTGTCCACCACGAGCCAGCTCCTC 8280
8375             TGGGTCTCTGGAGCGAAAAGCAACACCATAAAAAGTGTGTCCACCACGAGCCAGCTCCTC 8280
103344           TGGGTCTCTGGAGCGAAAAGCAACACCATAAAAAGTGTGTCCACCACGAGCCAGCTCCTC 8280
BrasilZKV2015    TGGGTCTCTGGAGCGAAAAGCAACACCATAAAAAGTGTGTCCACCACGAGCCAGCTCCTC 8280
GD01             TGGGTCTCTGGAGCGAAAAGCAACATCATAAAAAGTGTGTCCACCACGAGCCAGCTCCTC 8280
Martinique       TGGGTCTCTGGAGCGAAAAGCAACACCATAAAAAGTGTGTCCACCACGAGCCAGCTCCTC 8280
NatalRGN         TGGGTCTCTGGAGCGAAAAGCAACACCATAAAAAGTGTGTCCACCACGAGCCAGCTCCTC 8280
HPF2013          TGGGTCTCTGGAGCGAAAAGCAACACCATAAAAAGTGTGTCCACCACGAGCCAGCTCCTC 8280
SPH2015          TGGGTCTCTGGAGCGAAAAGCAACACCATAAAAAGTGTGTCCACCACGAGCCAGCTCCTC 8280
Haiti2014        TGGGTCTCTGGAGCGAAAAGCAACACCATAAAAAGTGTGTCCACCACGAGCCAGCTCCTC 8280
PRVABC59         TGGGTCTCTGGAGCGAAAAGCAACACCATAAAAAGTGTGTCCACCACGAGCCAGCTCCTC 8280
BeH819015        TGGGTCTCTGGAGCGAAAAGCAACACCATAAAAAGTGTGTCCACCACGAGCCAGCTCCTC 8280
Z1106033         TGGGTCTCTGGAGCGAAAAGCAACACCATAAAAAGTGTGTCCACCACGAGCCAGCTCCTC 8280
BEH819966        TGGGTCTCTGGAGCGAAAAGCAACACCATAAAAAGTGTGTCCACCACGAGCCAGCTCCTC 8280
SSABr            TGGGTCTCTGGAGCGAAAAGCAACACCATAAAAAGTGTGTCCACCACGAGCCAGCTCCTC 8280
Beh815744        TGGGTCTCTGGAGCGAAAAGCAACACCATAAAAAGTGTGTCCACCACGAGCCAGCTCCTC 8280
BEH818995        TGGGTCTCTGGAGCGAAAAGCAACACCATAAAAAGTGTGTCCACCACGAGCCAGCTCCTC 8280
IbH30656         TGGGTCTCTGGAGCCAAAAGTAACATCATAAAGAGTGTGTCCACCACAAGTCAGCTCCTC 8262
ArD128000        TGGGTCTCTGGAGCCAAAAGTAACATCATAAAGAGTGTGTCCACCACAAGTCAGCTCCTC 8280
ArD7117          TGGGTCTCTGGAGCCAAAAGTAACATCATAAAGAGTGTGTCCACCACAAGTCAGCTCCTC 8280
ARD_41519        TGGGTCTCTGGAGCCAAAAGTAACATCATAAAGAGTGTGTCCACCACAAGTCAGCTCCTC 8280
ARD157995        TGGGTCTCTGGGGCAAAAAGCAACATCATAAAAAGTGTGTCCACCACAAGTCAGCTCCTC 8280
MR_766           TGGGTCTCTGGGGCAAAGAGCAACATCATAAAAAGTGTGTCCACCACAAGTCAGCTCCTC 8268
```

```
ARD158084           TGGGTCTCTGGGGCAAAAAGCAACATCATAAAAAGTGTGTCCACCACAAGTCAGCTCCTC 8280
ArB1362             TGGGTCTCTGGAGCAAAAAGCAACATCATAAAAAGTGTGTCCACCACAAGCCAGCTCCTC 8280
ARB13565            TGGGTCTCTGGAGCAAAAAGTAACGTCATAAAAAGTGTGTCCACCACAAGTCAGCTCCTC 8280
ARB7701             TGGGTCTCTGGAGCAAAAAGTAACGTCATAAAAAGTGTGTCCACCACAAGTCAGCTCCTC 8280
ARB15076            TGGGTCTCTGGAGCAAAAAGTAACATCATAAAAAGTGTGTCCACCACAAGTCAGCTCCTC 8262
                    *********** ** ** ** ***  ****** ************** ** ********

P6-740              TTGGGACGCATGGACGGGCCCAGGAGGCCAGTGAAATATGAGGAGGATGTGAATCTCGGC 8340
CPC0740             TTGGGGCGCATGGACGGGCCCAGGAGGCCAGTGAAATATGAGGAGGATGTGAATCTCGGC 8340
YAP2007             TTGGGGCGCATGGACGGGCCCAGGAGGCCAGTGAAATATGAGGAGGATGTGAATCTCGGC 8340
FSS13025            TTGGGGCGCATGGACGGGCCCAGGAGGCCAGTGAAATATGAAGAGGATGTGAATCTCGGC 8340
PLCal_ZV            TTGGGGCGCATGGACGGGCCCAGGAGGCCAGTGAAATATGAGGAGGATGTGAATCTCGGC 8229
SV0127/14           TTGGGGCGCATGGACGGGCCCAGGAGGCCAGTGAAATATGAGGAGGATGTGAATCTCGGC 8340
8375                TTGGGGCGCATGGACGGGCCTAGGAGGCCAGTGAAATATGAGGAGGATGTGAATCTCGGC 8340
103344              TTGGGGCGCATGGACGGGCCTAGGAGGCCAGTGAAATATGAGGAGGATGTGAATCTCGGC 8340
BrasilZKV2015       TTGGGGCGCATGGACGGGCCTAGGAGGCCAGTGAAATATGAGGAGGATGTGAATCTCGGC 8340
GD01                TTGGGGCGCATGGACGGGCCTAGGAGGCCAGTGAAATATGAGGAGGATGTGAATCTCGGC 8340
Martinique          TTGGGGCGCATGGACGGGCCTAGGAGGCCAGTGAAATATGAGGAGGATGTGAATCTCGGC 8340
NatalRGN            TTGGGGCGCATGGACGGGCCTAGGAGGCCAGTGAAATATGAGGAGGATGTGAATCTCGGC 8340
HPF2013             TTGGGGCGCATGGACGGGCCCAGGAGGCCAGTGAAATATGAGGAGGATGTGAATCTCGGC 8340
SPH2015             TTGGGGCGCATGGACGGGCCTAGGAGGCCAGTGAAATATGAGGAGGATGTGAATCTCGGC 8340
Haiti2014           TTGGGGCGCATGGACGGGCCTAGGAGGCCAGTGAAATATGAGGAGGATGTGAATCTCGGC 8340
PRVABC59            TTGGGGCGCATGGACGGGCCTAGGAGGCCAGTGAAATATGAGGAGGATGTGAATCTCGGC 8340
BeH819015           TTGGGGCGCATGGACGGGCCTAGGAGGCCAGTGAAATATGAGGAGGATGTGAATCTCGGC 8340
Z1106033            TTGGGGCGCATGGACGGGCCTAGGAGGCCAGTGAAATATGAGGAGGATGTGAATCTCGGC 8340
BEH819966           TTGGGGCGCATGGACGGGCCTAGGAGGCCAGTGAAATATGAGGAGGATGTGAATCTCGGC 8340
SSABr               TTGGGGCGCATGGACGGGCCTAGGAGGCCAGTGAAATATGAGGAGGATGTGAATCTCGGC 8340
Beh815744           TTGGGGCGCATGGACGGGCCTAGGAGGCCAGTGAAATATGAGGAGGATGTGGATCTCGGC 8340
BEH818995           TTGGGGCGCATGGACGGGCCTAGGAGGCCAGTGAAATATGAGGAGGATGTGGATCTCGGC 8340
IbH30656            TTGGGACGCATGGATGGGCCTAGGAGGCCAGTGAAATATGAAGAGGATGTGAACCTCGGC 8322
ArD128000           TTGGGACGCATGGATGGGCCTAGGAGGCCAGTGAAATATGAGGAGGATGTGAACCTCGGC 8340
ArD7117             TTGGGACGCATGGATGGGCCTAGGAGGCCAGTGAAATATGAGGAGGATGTGAACCTCGGC 8340
ARD_41519           TTGGGACGCATGGAAGGGCCTAGGAGGCCAGTGAAATATGAGGAGGATGTGAACCTCGGC 8340
ARD157995           CTGGGACGCATGGATGGCCCCAGGAGGCCAGTGAAATATGAGGAGGATGTGAACCTCGGC 8340
MR_766              CTGGGACGCATGGATGGCCCCAGGAGGCCAGTGAAATATGAGGAGGATGTGAACCTCGGC 8328
ARD158084           CTGGGACGCATGGATGGCCCCAGGAGGCCAGTGAAATATGAGGAGGATGTGAACCTCGGC 8340
ArB1362             CTGGGACGCATGGATGGGCCCAGGAGGCCAGTGAAATATGAGGAGGATGTGAACCTCGGC 8340
ARB13565            CTGGGACGCATGGATGGTCCCAGGAGGCCAGTGAAATATGAGGAAGATGTGAACCTCGGC 8340
ARB7701             CTGGGACGCATGGATGGTCCCAGGAGGCCAGTGAAATATGAGGAAGATGTGAACCTCGGC 8340
ARB15076            CTGGGACGCATGGATGGCCCCAGGAGGCCAGTGAAATATGAGGAAGATGTGAACCTCGGC 8322
                     **** ******** ** ** ******************* ** ****** * ******

P6-740              TCCGGCACGCGAGCTGTGGCAAGCTGCGCCGAAGCTCCCAACCTGAAGATCATTGGTAAC 8400
CPC0740             TCTGGCACGCGGGCTGTGGTAAGCTGCGCTGAAGCTCCCAACATGAAGATCATTGGTAAC 8400
YAP2007             TCCGGCACGCGGGCTGTGGTAAGCTGCGCTGAAGCTCCCAACATGAAGATCATTGGTAAC 8400
FSS13025            TCTGGCACGCGGGCTGTGGTAAGCTGCGCTGAAGCTCCCAACATGAAGATCATTGGTAAC 8400
PLCal_ZV            TCTGGCACGCGGGCTGTGGTAAGCTGCGCTGAAGCTCCCAACATGAAGATCATTGGTAAC 8289
SV0127/14           TCTGGCACGCGGGCTGTGGTAAGCTGCGCTGAAGCTCCCAACATGAAGATCATTGGTAAC 8400
8375                TCTGGCACGCGGGCTGTGGTAAGCTGCGCTGAAGCTCCCAACATGAAGATCATTGGTAAC 8400
103344              TCTGGCACGCGGGCTGTGGTAAGCTGCGCTGAAGCTCCCAACATGAAGATCATTGGTAAC 8400
BrasilZKV2015       TCTGGCACGCGGGCTGTGGTAAGCTGCGCTGAAGCTCCCAACATGAAGATCATTGGTAAC 8400
GD01                TCTGGCACGCGGGCTGTGGCAAGCTGCGCTGAAGCTCCCAACATGAAGATCATTGGTAAC 8400
Martinique          TCTGGCACGCGGGCTGTGGTAAGCTGCGCTGAAGCTCCCAACATGAAGATCATTGGTAAC 8400
NatalRGN            TCTGGCACGCGGGCTGTGGTAAGCTGCGCTGAAGCTCCCAACATGAAGATCATTGGTAAC 8400
HPF2013             TCTGGCACGCGGGCTGTGGTAAGCTGCGCTGAAGCTCCCAACATGAAGATCATTGGTAAC 8400
SPH2015             TCTGGCACGCGGGCTGTGGTAAGCTGCGCTGAAGCTCCCAACATGAAGATCATTGGTAAC 8400
Haiti2014           TCTGGCACGCGGGCTGTGGTAAGCTGCGCTGAAGCTCCCAACATGAAGATCATTGGTAAC 8400
PRVABC59            TCTGGCACGCGGGCTGTGGTAAGCTGCGCTGAAGCTCCCAACATGAAGATCATTGGTAAC 8400
BeH819015           TCTGGCACGCGGGCTGTGGTAAGCTGCGCTGAAGCTCCCAACATGAAGATCATTGGTAAC 8400
Z1106033            TCTGGCACGCGGGCTGTGGTAAGCTGCGCTGAAGCTCCCAACATGAAGATCATTGGTAAC 8400
BEH819966           TCTGGCACGCGGGCTGTGGTAAGCTGCGCTGAAGCTCCCAACATGAAGATCATTGGTAAC 8400
SSABr               TCTGGCACGCGGGCTGTGGTAAGCTGCGCTGAAGCTCCCAACATGAAGATCATTGGTAAC 8400
Beh815744           TCTGGCACGCGGGCTGTGGTAAGCTGCGCTGAAGCTCCCAACATGAAGATCATTGGTAAC 8400
BEH818995           TCTGGCACGCGGGCTGTGGTAAGCTGCGCTGAAGCTCCCAACATGAAGATCATTGGTAAC 8400
IbH30656            TCAGGCACACGAGCTGTGGCAAGCTGTGCTGAGGCTCCCAACATGAAGATCATTGGTAGG 8382
ArD128000           TCAGGCACACGAGCTGTGGCAAGCTGCGCTGAGGCTCCCAACATGAAGATCATTGGTAGG 8400
ArD7117             TCAGGCACACGAGCTGTGGCAAGCTGCGCTGAGGCTCCCAACATGAAGATCATTGGTAGG 8400
ARD_41519           TCAGGCACACGAGCTGTGGCAAGCTGCGCTGAGGCTCCCAACATGAAGATCATTGGTAGG 8400
```

```
ARD157995           TCGGGTACACGAGCTGTGGCAAGCTGTGCTGAGGCTCCTAACATGAAAATCATCGGCAGG 8400
MR_766              TCGGGTACACGAGCTGTGGCAAGCTGTGCTGAGGCTCCTAACATGAAAATCATCGGCAGG 8388
ARD158084           TCGGGTACACGAGCTGTGGCAAGCTGTGCTGAGGCTCCTAACATGAAAATCATCGGCAGG 8400
ArB1362             TCGGGCACACGAGCTGTGGCAAGCTGTGCTGAGGCTCCCAACATGAAGATCATTGGTAAG 8400
ARB13565            TCGGGCACACGAGCTGTGGCAAGCTGTGCTGAAGCTCCCAACATGAAGGTCATTGGCAAG 8400
ARB7701             TCGGGCACACGAGCTGTGGCAAGCTGTGCTGAAGCTCCCAACATGAAGGTCATTGGCAAG 8400
ARB15076            TCGGGCACACGAGCTGTGGCAAGCTGTGCTGAAGCTCCCAACATGAAGATCATTGGCAAG 8382
                    ** ** ** ** ******* ****** ** ** ***** *** ****  **** ** *

P6-740              CGCGTTGAGAGGATCCGCAGTGAGCATGCGGAAACGTGGTTCTTTGATGAGAACCACCCA 8460
CPC0740             CGCATTGAGAGGATCCGCAGTGAGCACGCGGAAACGTGGTTCTTTGACGAGAACCACCCA 8460
YAP2007             CGCATTGAGAGGATCCGCAGTGAGCACGCGGAAACGTGGTTCTTTGACGAGAACCACCCA 8460
FSS13025            CGCATTGAGAGGATCCGCAGTGAGCACGCGGAAACGTGGTTCTTTGACGAGAACCACCCA 8460
PLCal_ZV            CGCATTGAGAGGATCCGCAGTGAGCACGCGGAAACGTGGTTCTTTGACGAGAACCACCCA 8349
SV0127/14           CGCATTGAAAGGATCCGCAGTGAGCACGCGGAAACGTGGTTCTTTGACGAGAACCACCCA 8460
8375                CGCATTGAAAGGATCCGCAGTGAGCACGCGGAAACGTGGTTCTTTGACGAGAACCACCCA 8460
103344              CGCATTGAAAGGATCCGCAGTGAGCACGCGGAAACGTGGTTCTTTGACGAGAACCACCCA 8460
BrasilZKV2015       CGCATTGAAAGGATCCGCAGTGAGCACGCGGAAACGTGGTTCTTTGACGAAAACCACCCA 8460
GD01                CGCATTGAAAGGATCCGCAGTGAGCACGCGGAAACGTGGTTCTTTGACGAGAACCACCCA 8460
Martinique          CGCATTGAAAGGATCCGCAGTGAGCACGCGGAAACGTGGTTCTTTGACGAGAACCACCCA 8460
NatalRGN            CGCATTGAAAGGATCCGCAGTGAGCACGCGGAAACGTGGTTCTTTGACGAGAACCACCCA 8460
HPF2013             CGCATTGAAAGGATCCGCAGTGAGCACGCGGAAACGTGGTTCTTTGACGAGAACCACCCA 8460
SPH2015             CGCATTGAAAGGATCCGCAGTGAGCACGCGGAAACGTGGTTCTTTGACGAGAACCACCCA 8460
Haiti2014           CGCATTGAAAGGATCCGCAGTGAGCACGCGGAAACGTGGTTCTTTGACGAGAACCACCCA 8460
PRVABC59            CGCATTGAAAGGATCCGCAGTGAGCACGCGGAAACGTGGTTCTTTGACGAGAACCACCCA 8460
BeH819015           CGCATTGAAAGGATCCGCAGTGAGCACGCGGAAACGTGGTTCTTTGACGAGAACCACCCA 8460
Z1106033            CGCATTGAAAGGATCCGCAGTGAGCACGCGGAAACGTGGTTCTTTGACGAGAACCACCCA 8460
BEH819966           CGCATTGAAAGGATCCGCAGTGAGCACGCGGAAACGTGGTTCTTTGACGAGAACCACCCA 8460
SSABr               CGCATTGAAAGGATCCGCAGTGAGCACGCGGAAACGTGGTTCTTTGACGAGAACCACCCA 8460
Beh815744           CGCATTGAAAGGATCCGCAGTGAGCACGCGGAAACGTGGTTCTTTGACGAGAACCACCCA 8460
BEH818995           CGCATTGAAAGGATCCGCAGTGAGCACGCGGAAACGTGGTTCTTTGACGAGAACCACCCA 8460
IbH30656            CGCATTGAGAGAATCCGCAATGAACATGCAGAGACATGGTTCTTTGATGAAAACCACCCA 8442
ArD128000           CGCATTGAGAGAATCCGCAATGAACATGCAGAGACATGGTTCTTTGATGAAAACCACCCA 8460
ArD7117             CGCATTGAGAGAATCCGCAATGAACATGCAGAGACATGGTTCTTTGATGAAAACCACCCA 8460
ARD_41519           CGCATTGAGAGAATCCGCAATGAACATGCAGAGACATGGTTCTTTGATGAAAACCACCCA 8460
ARD157995           CGCATTGAGAGAATCCGCAATGAACATGCAGAAACATGGTTTCTTGATGAAAACCACCCA 8460
MR_766              CGCATTGAGAGAATCCGCAATGAACATGCAGAAACATGGTTTCTTGATGAAAACCACCCA 8448
ARD158084           CGCATTGAGAGAATCCGCAATGAACATGCAGAAACATGGTTTCTTGATGAAAACCACCCA 8460
ArB1362             CGCATTGAGAGAATCCGCAGTGAACATGCAGAAACATGGTTCTTTGATGAAAACCACCCA 8460
ARB13565            CGCATTGAGAGAATCCGCAGTGAACATGCAGAAACATGGTTCTTTGATGAAAACCATCCA 8460
ARB7701             CGCATTGAGAGAATCCGCAGTGAACATGCAGAAACATGGTTCTTTGATGAAAACCATCCA 8460
ARB15076            CGCATTGAGAGAATCCGCAGTGAACATGCAGAAACATGGTTCTTTGATGAAAACCACCCA 8442
                    *** **** ** ******* *** ** ** ** ** *****  **** ** ***** ***

P6-740              TACAGGACATGGGCTTACCATGGGAGCTACGAGGCCCCTACACAAGGGTCAGCGTCTTCT 8520
CPC0740             TATAGGACATGGGCTTACCATGGAAGCTATGAGGCCCCTACACAAGGGTCAGCGTCCTCT 8520
YAP2007             TATAGGACATGGGCTTACCATGGAAGCTATGAGGCCCCTACACAAGGGTCAGCGTCCTCT 8520
FSS13025            TATAGGACATGGGCTTACCATGGAAGCTACGAGGCCCCCACACAAGGGTCAGCGTCCTCT 8520
PLCal_ZV            TATAGGACATGGGCTTACCATGGAAGCTATGAGGCCCCTACACAAGGGTCAGCGTCCTCT 8409
SV0127/14           TATAGGACATGGGCTTACCATGGAAGCTATGAGGCCCCTACACAAGGGTCAGCGTCCTCT 8520
8375                TATAGGACATGGGCTTACCATGGAAGCTATGAGGCCCCCACACAAGGGTCAGCGTCCTCT 8520
103344              TATAGGACATGGGCTTACCATGGAAGCTATGAGGCCCCCACACAAGGGTCAGCGTCCTCT 8520
BrasilZKV2015       TATAGGACATGGGCTTACCATGGAAGCTATGTGGCCCCCACACAAGGGTCAGCGTCCTCT 8520
GD01                TATAGGACATGGGCTTACCATGGAAGCTATGAGGCCCCCACACAAGGGTCAGCGTCCTCT 8520
Martinique          TATAGGACATGGGCTTACCATGGAAGCTATGAGGCCCCCACACAAGGGTCAGCGTCCTCT 8520
NatalRGN            TATAGGACATGGGCTTACCATGGAAGCTATGAGGCCCCCACACAAGGGTCAGCGTCCTCT 8520
HPF2013             TATAGGACATGGGCTTACCATGGAAGCTATGAGGCCCCCACACAAGGGTCAGCGTCCTCT 8520
SPH2015             TATAGGACATGGGCTTACCATGGAAGCTATGAGGCCCCCACACAAGGGTCAGCGTCCTCT 8520
Haiti2014           TATAGGACATGGGCTTACCATGGAAGCTATGAGGCCCCCACACAAGGGTCAGCGTCCTCT 8520
PRVABC59            TATAGGACATGGGCTTACCATGGAAGCTATGAGGCCCCCACACAAGGGTCAGCGTCCTCT 8520
BeH819015           TATAGGACATGGGCTTACCATGGAAGCTATGAGGCCCCCACACAAGGGTCAGCGTCCTCT 8520
Z1106033            TATAGGACATGGGCTTACCATGGAAGCTATGAGGCCCCCACACAAGGGTCAGCGTCCTCT 8520
BEH819966           TATAGGACATGGGCTTACCATGGAAGCTATGAGGCCCCCACACAAGGGTCAGCGTCCTCT 8520
SSABr               TATAGGACATGGGCTTACCATGGAAGCTATGAGGCCCCCACACAAGGGTCAGCGTCCTCT 8520
Beh815744           TATAGGACATGGGCTTACCATGGAAGCTATGAGGCCCCCACACAAGGGTCAGCGTCCTCT 8520
BEH818995           TATAGGACATGGGCTTACCATGGAAGCTATGAGGCCCCCACACAAGGGTCAGCGTCCTCT 8520
IbH30656            TACAGGACATGGGCCTACCATGGGAGCTACGAAGCCCCCACGCAGGGGTCAGCGTCATCC 8502
ArD128000           TACAGGACATGGGCCTACCATGGGAGCTACGAAGCCCCCACGCAGGGGTCAGCGTCATCC 8520
```

```
ArD7117         TACAGGACATGGGCCTACCATGGGAGCTACGAAGCCCCCACGCAGGGGTCAGCGTCATCC 8520
ARD_41519       TACAGGACATGGGCCTACCATGGGAGCTACGAAGCCCCCACGCAGGGGTCAGCGTCATCC 8520
ARD157995       TACAGGACATGGGCCTACCATGGGAGCTACGAAGCCCCCACGCAAGGATCAGCGTCTTCC 8520
MR_766          TACAGGACATGGGCCTACCATGGGAGCTACGAAGCCCCCACGCAAGGATCAGCGTCTTCC 8508
ARD158084       TACAGGACATGGGCCTACCATGGGAGCTACGAAGCCCCCACGCAAGGATCAGCGTCTTCC 8520
ArB1362         TACAGGACATGGGCTTACCATGGGAGCTACGAAGCCCCCACGCAAGGGTCAGCGTCTTCC 8520
ARB13565        TACAGGACATGGGCCTACCATGGGAGCTACGAAGCCCCCACACAAGGGTCAGCATCTTCC 8520
ARB7701         TACAGGACATGGGCCTACCATGGGAGCTACGAAGCCCCCACACAAGGGTCAGCATCTTCC 8520
ARB15076        TACAGGACATGGGCCTACCATGGGAGCTACGAAGCCCCCACCCAAGGATCAGCGTCTTCC 8502
                ** *********** ******** ***** *  ***** ** ** ** ***** ** **

P6-740          CTCATAAACGGGGTTGTCAGGCTCCTGTCAAAGCCCTGGGATGTGGTGACTGGAGTCACA 8580
CPC0740         CTAATAAACGGGGTTGTCAGGCTCCTGTCAAAACCCTGGGATGTGGTGACTGGAGTCACA 8580
YAP2007         CTAATAAACGGGGTTGTCAGGCTCCTGTCAAAACCCTGGGATGTGGTGACTGGAGTCACA 8580
FSS13025        CTAATAAACGGGGTTGTCAGGCTCCTGTCAAAACCCTGGGATGTGGTGACTGGAGTCACA 8580
PLCal_ZV        CTAATAAACGGGGTTGTCAGGCTCCTGTCAAAACCCTGGGATGTGGTGACTGGAGTCACA 8469
SV0127/14       CTAATAAACGGGGTTGTCAGGCTCCTGTCAAAACCCTGGGATGTGGTGACTGGAGTCACA 8580
8375            CTAATAAACGGGGTTGTCAGGCTCCTGTCAAAACCCTGGGATGTGGTGACTGGAGTCACA 8580
103344          CTAATAAACGGGGTTGTCAGGCTCCTGTCAAAACCCTGGGATGTGGTGACTGGAGTCACA 8580
BrasilZKV2015   CTAATAAACGGGGTTGTCAGGCTCCTGTCAAAACCCTGGGATGTGGTGACTGGAGTCACA 8580
GD01            CTAATAAACGGGGTTGTCAGGCTCCTGTCAAAACCCTGGGATGTGGTGACTGGAGTCACA 8580
Martinique      CTAATAAACGGGGTTGTCAGGCTCCTGTCAAAACCCTGGGATGTGGTGACTGGAGTCACA 8580
NatalRGN        CTAATAAACGGGGTTGTCAGGCTCCTGTCAAAACCCTGGGATGTGGTGACTGGAGTCACA 8580
HPF2013         CTAATAAACGGGGTTGTCAGGCTCCTGTCAAAACCCTGGGATGTGGTGACTGGAGTCACA 8580
SPH2015         CTAATAAACGGGGTTGTCAGGCTCCTGTCAAAACCCTGGGATGTGGTGACTGGAGTCACA 8580
Haiti2014       CTAATAAACGGGGTTGTCAGGCTCCTGTCAAAACCCTGGGATGTGGTGACTGGAGTCACA 8580
PRVABC59        CTAATAAACGGGGTTGTCAGGCTCCTGTCAAAACCCTGGGATGTGGTGACTGGAGTCACA 8580
BeH819015       CTAATAAACGGGGTTGTCAGGCTCCTGTCAAAACCCTGGGATGTGGTGACTGGAGTCACA 8580
Z1106033        CTAATAAACGGGGTTGTCAGGCTCCTGTCAAAACCCTGGGATGTGGTGACTGGAGTCACA 8580
BEH819966       CTAATAAACGGGGTTGTCAGGCTCCTGTCAAAACCCTGGGATGTGGTGACTGGAGTCACA 8580
SSABr           CTAATAAACGGGGTTGTCAGGCTCCTGTCAAAACCCTGGGATGTGGTGACTGGAGTCACA 8580
Beh815744       CTAATAAACGGGGTTGTCAGGCTCCTGTCAAAACCCTGGGATGTGGTGACTGGAGTCACA 8580
BEH818995       CTAATAAACGGGGTTGTCAGGCTCCTGTCAAAACCCTGGGATGTGGTGACTGGAGTCACA 8580
IbH30656        CTCGTGAACGGGGTTGTTAGACTCCTGTCAAAGCCCTGGGATGTGGTGACTGGAGTCACA 8562
ArD128000       CTCGTGAATGGGGTTGTTAGACTCTTGTCAAAGCCCTGGGATGTGGTGACTGGAGTTACA 8580
ArD7117         CTCGTGAACGGGGTTGTTAGACTCTTGTCAAAACCCTGGGATGTGGTGACTGGAGTTACA 8580
ARD_41519       CTCGTGAACGGGGTTGTTAGACTCTTGTCAAAGCCCTGGGATGTGGTGACTGGAGTTACA 8580
ARD157995       CTCGTGAACGGGGTTGTTAGACTCCTGTCAAAGCCTTGGGACGTGGTGACTGGAGTTACA 8580
MR_766          CTCGTGAACGGGGTTGTTAGACTCCTGTCAAAGCCTTGGGACGTGGTGACTGGAGTTACA 8568
ARD158084       CTCGTGAACGGGGTTGTTAGACTCCTGTCAAAGCCTTGGGACGTGGTGACTGGAGTTACA 8580
ArB1362         CTCGTGAATGGGGTTGTTAGGCTCCTGTCAAAGCCCTGGGATGTAGTGACTGGAGTTACA 8580
ARB13565        CTCGTGAATGGGGTTGTTAGACTCCTGTCAAAGCCCTGGGATGTAGTGACTGGAGTTACA 8580
ARB7701         CTCGTGAATGGGGTTGTTAGACTCCTGTCAAAGCCCTGGGATGTAGTGACTGGAGTTACA 8580
ARB15076        CTCGTGAATGGGGTTGTTAGACTCCTGTCAAAGCCCTGGGATGTTGTGACTGGAGTTACA 8562
                **  * ** ******** ** *** ******* ** ***** ** *********** ***

P6-740          GGAATAGCCATGACCGACACCACACCGTATGGCCAGCAAAGAGTTTTCAAGGAAAAAGTG 8640
CPC0740         GGAATAGCCATGACTGACACCACACCGTATGGTCAGCAAAGAGTTTTCAAGGAAAAAGTG 8640
YAP2007         GGAATAGCCATGACCGACACCACACCGTATGGTCAGCAAAGAGTTTTCAAGGAAAAAGTG 8640
FSS13025        GGAATAGCCATGACCGACACCACACCGTATGGTCAGCAAAGAGTTTTCAAGGAAAAAGTG 8640
PLCal_ZV        GGAATAGCCATGACCGACACCACACCGTATGGTCAGCAAAGAGTTTTCAAGGAAAAAGTG 8529
SV0127/14       GGAATAGCCATGACCGACACCACACCGTATGGTCAGCAAAGAGTTTTCAAGGAAAAAGTG 8640
8375            GGAATAGCCATGACCGACACCACACCGTATGGTCAGCAAAGAGTTTTCAAGGAAAAAGTG 8640
103344          GGAATAGCCATGACCGACACCACACCGTATGGTCAGCAAAGAGTTTTCAAGGAAAAAGTG 8640
BrasilZKV2015   GGAATAGCCATGACCGACACCACACCGTATGGTCAGCAAAGAGTTTTCAAGGAAAAAGTG 8640
GD01            GGAATAGCCATGACCGACACCACACCGTATGGTCAGCAAAGAGTTTTCAAGGAAAAAGTG 8640
Martinique      GGAATAGCCATGACCGACACCACACCGTATGGTCAGCAAAGAGTTTTCAAGGAAAAAGTG 8640
NatalRGN        GGAATAGCCATGACCGACACCACACCGTATGGTCAGCAAAGAGTTTTCAAGGAAAAAGTG 8640
HPF2013         GGAATAGCCATGACCGACACCACACCGTATGGTCAGCAAAGAGTTTTCAAGGAAAAAGTG 8640
SPH2015         GGAATAGCCATGACCGACACCACACCGTATGGTCAGCAAAGAGTTTTCAAGGAAAAAGTG 8640
Haiti2014       GGAATAGCCATGACCGACACCACACCGTATGGTCAGCAAAGAGTTTTCAAGGAAAAAGTG 8640
PRVABC59        GGAATAGCCATGACCGACACCACACCGTATGGTCAGCAAAGAGTTTTCAAGGAAAAAGTG 8640
BeH819015       GGAATAGCCATGACCGACACCACACCGTATGGTCAGCAAAGAGTTTTCAAGGAAAAAGTG 8640
Z1106033        GGAATAGCCATGACCGACACCACACCGTATGGTCAGCAAAGAGTTTTCAAGGAAAAAGTG 8640
BEH819966       GGAATAGCCATGACCGACACCACACCGTATGGTCAGCAAAGAGTTTTCAAGGAAAAAGTG 8640
SSABr           GGAATAGCCATGACCGACACCACACCGTATGGTCAGCAAAGAGTTTTCAAGGAAAAAGTG 8640
Beh815744       GGAATAGCCATGACCGACACCACACCGTATGGTCAGCAAAGAGTTTTCAAGGAAAAAGTG 8640
BEH818995       GGAATAGCCATGACCGACACCACACCGTATGGTCAGCAAAGAGTTTTCAAGGAAAAAGTG 8640
```

```
IbH30656        GGAATAGCTATGACTGACACCACGCCATACGGCCAACAAAGAGTCTTCAAAGAAAAGGTG 8622
ArD128000       GGAATAGCTATGACTGACACCACGCCATACGGCCAACAAAGAGTCTTCAAGGAAAAAGTG 8640
ArD7117         GGAATAGCTATGACTGACACCACGCCATACGGCCAACAAAGAGTCTTCAAGGAAAAAGTG 8640
ARD_41519       GGAATAGCTATGACTGACACCACGCCATACGGCCAACAAAGAGTCTTCAAGGAAAAAGTG 8640
ARD157995       GGAATAGCCATGACTGACACCACACCATACGGCCAACAAAGAGTCTTCAAAGAAAAAGTG 8640
MR_766          GGAATAGCCATGACTGACACCACACCATACGGCCAACAAAGAGTCTTCAAAGAAAAAGTG 8628
ARD158084       GGAATAGCCATGACTGACACCACACCATACGGCCAACAAAGAGTCTTCAAAGAAAAAGTG 8640
ArB1362         GGAATAGCTATGACTGACACCACACCATATGGCCAACAAAGAGTCTTCAAAGAAAAAGTG 8640
ARB13565        GGAATAGCTATGACTGATACAACACCATACGGCCAACAAAGAGTCTTCAAAGAAAAAGTG 8640
ARB7701         GGAATAGCTATGACTGATACAACACCATACGGCCAACAAAGAGTCTTCAAAGAAAAAGTG 8640
ARB15076        GGAATAGCTATGACTGACACTACACCATACGGCCAACAAAGAGTCTTCAAAGAAAAAGTG 8622
                ******** ***** ** ** ** ** ** ** ** ******** ***** ***** ***

P6-740          GACACTAGGGTGCCAGACCCCCAGGAAGGCACTCGTCAGGTGATGAACATGGTCTCTTCC 8700
CPC0740         GACACTAGGGTGCCAGACCCCCAAGAAGGCACTCGTCAGGTTATGAGCATGGTCTCTTCC 8700
YAP2007         GACACTAGGGTGCCAGACCCCCAAGAAGGCACTCGTCAGGTTATGAGCATGGTCTCTTCC 8700
FSS13025        GACACTAGGGTGCCAGACCCCCAAGAAGGCACTCGTCAGGTTATGAGCATGGTCTCTTCC 8700
PLCal_ZV        GACACTAGGGTGCCAGACCCCCAAGAAGGCACTCGTCAGATTATGAGCATGGTCTCTTCC 8589
SV0127/14       GACACCAGGGTGCCAGACCCCCAAGAAGGCACTCGTCAGGTTATGAGCATGGTCTCTTCC 8700
8375            GACACTAGGGTGCCAGACCCCCAAGAAGGCACTCGTCAGGTTATGAGCATGGTCTCTTCC 8700
103344          GACACTAGGGTGCCAGACCCCCAAGAAGGCACTCGTCAGGTTATGAGCATGGTCTCTTCC 8700
BrasilZKV2015   GACACTAGGGTGCCAGACCCCCAAGAAGGCACTCGTCAGGTTATGAGCATGGTCTCTTCC 8700
GD01            GACACTAGGGTGCCAGACCCCCAAGAAGGCACTCGTCAGGTTATGAGCATGGTCTCTTCC 8700
Martinique      GACACTAGGGTGCCAGACCCCCAAGAAGGCACTCGTCAGGTTATGAGCATGGTCTCTTCC 8700
NatalRGN        GACACTAGGGTGCCAGACCCCCAAGAAGGCACTCGTCAGGTTATGAGCATGGTCTCTTCC 8700
HPF2013         GACACTAGGGTGCCAGACCCCCAAGAAGGCACTCGTCAGGTTATGAGCATGGTCTCTTCC 8700
SPH2015         GACACTAGGGTGCCAGACCCCCAAGAAGGTACTCGTCAGGTTATGAGCATGGTCTCTTCC 8700
Haiti2014       GACACTAGGGTGCCAGACCCCCAAGAAGGCACTCGTCAGGTTATGAGCATGGTCTCTTCC 8700
PRVABC59        GACACTAGGGTGCCAGACCCCCAAGAAGGCACTCGTCAGGTTATGAGCATGGTCTCTTCC 8700
BeH819015       GACACTAGGGTGCCAGACCCCCAAGAAGGCACTCGTCAGGTTATGAGCATGGTCTCTTCC 8700
Z1106033        GACACTAGGGTGCCAGACCCCCAAGAAGGCACTCGTCAGGTTATGAGCATGGTCTCTTCC 8700
BEH819966       GACACTAGGGTGCCAGACCCCCAAGAAGGCACTCGTCAGGTTATGAGCATGGTCTCTTCC 8700
SSABr           GACACTAGGGTGCCAGACCCCCAAGAAGGCACTCGTCAGGTTATGAGCATGGTCTCTTCC 8700
Beh815744       GACACTAGGGTGCCAGACCCCCAAGAAGGCACTCGTCAGGTTATGAGCATGGTCTCTTCC 8700
BEH818995       GACACTAGGGTGCCAGACCCCCAAGAAGGCACTCGTCAGGTTATGAGCATGGTCTCTTCC 8700
IbH30656        GACACTAGGGTGCCAGACCCCCAAGAAGGCACCCGCCGAGTAATGAACATGGTCTCGTCT 8682
ArD128000       GACACAAGGGTGCCAGATCCCCAAGAAGGCACTCGCCAAGTAATGAACATAGTTTCGTCT 8700
ArD7117         GACACTAGGGTGCCAGATCCCCAAGAAGGCACTCGCCAAGTAATGAACATGGTCTCGTCT 8700
ARD_41519       GACACCAGGGTGCCAGATCCCCAAGAAGGCACTCGCCAAGTGATGAACATGGTATCGTCT 8700
ARD157995       GACACCAGGGTGCCAGATCCCCAAGAAGGCACTCGCCAGGTAATGAACATGGTCTCTTCC 8700
MR_766          GACACCAGGGTGCCAGATCCCCAAGAAGGCACTCGCCAGGTAATGAACATAGTCTCTTCC 8688
ARD158084       GACACCAGGGTGCCAGATCCCCAAGAAGGCACTCGCCAGGTAATGAACATGGTCTCTTCC 8700
ArB1362         GACACCAGGGTGCCAGATCCCCAAGAAGGCACTCGCCAGACAATGAACATGGTCTCGTCC 8700
ARB13565        GACACCAGGGTGCCAGATCCCCAAGAAGGCACTCGCCAGGCAATGAACATGGTCTCTTCC 8700
ARB7701         GACACCAGGGTGCCAGATCCCCAAGAAGGCACTCGCCAGGCAATGAACATGGTCTCTTCC 8700
ARB15076        GACACCAGGGTGCCAGATCCTCAAGAAGGCACTCGCCAGACAATGAACATGGTTTCTTCC 8682
                ***** *********** ** ** ***** ** ** *     **** *** ** ** ** 

P6-740          TGGCTATGGAAGGAGCTAGGTAAACACAAACGGCCACGAGTTTGCACCAAAGAAGAGTTC 8760
CPC0740         TGGTTATGGAAGGAGCTAGGCAAACACAAACGGCCACGAGTCTGTACCAAAGAAGAGTTC 8760
YAP2007         TGGTTATGGAAAGAGCTAGGCAAACACAAACGGCCACGAGTCTGTACCAAAGAAGAGTTC 8760
FSS13025        TGGTTGTGGAAAGAGTTAGGCAAACACAAACGGCCACGAGTCTGTACCAAAGAAGAGTTC 8760
PLCal_ZV        TGGTTGTGGAAAGAGCTAGGCAAACACAAACGGCCACGAGTCTGTACCAAAGAAGAGTTC 8649
SV0127/14       TGGTTGTGGAAAGAGCTAGGCAAACACAAACGGCCACGAGTCTGTACCAAAGAAGAGTTC 8760
8375            TGGTTGTGGAAAGAGCTAGGCAAACACAAACGGCCACGAGTCTGTACCAAAGAAGAGTTC 8760
103344          TGGTTGTGGAAAGAGCTAGGCAAACACAAACGGCCACGAGTCTGTACCAAAGAAGAGTTC 8760
BrasilZKV2015   TGGTTGTGGAAAGAGCTAGGCAAACACAAACGACCACGAGTCTGTACCAAAGAAGAGTTC 8760
GD01            TGGTTGTGGAAAGAGCTAGGCAAACACAAACGGCCACGAGTCTGTACCAAAGAAGAGTTC 8760
Martinique      TGGTTGTGGAAAGAGCTAGGCAAACACAAACGGCCACGAGTCTGTACCAAAGAAGAGTTC 8760
NatalRGN        TGGTTGTGGAAAGAGCTAGGCAAACACAAACGGCCACGAGTCTGTACCAAAGAAGAGTTC 8760
HPF2013         TGGTTGTGGAAAGAGCTAGGCAAACACAAACGGCCACGAGTCTGTACCAAAGAAGAGTTC 8760
SPH2015         TGGTTGTGGAAAGAGCTAGGCAAACACAAACGGCCACGAGTCTGTACCAAAGAAGAGTTC 8760
Haiti2014       TGGTTGTGGAAAGAGCTAGGCAAACACAAACGGCCACGAGTCTGTACCAAAGAAGAGTTC 8760
PRVABC59        TGGTTGTGGAAAGAGCTAGGCAAACACAAACGGCCACGAGTCTGCACCAAAGAAGAGTTC 8760
BeH819015       TGGTTGTGGAAAGAGCTAGGCAAACACAAACGGCCACGAGTCTGTACCAAAGAAGAGTTC 8760
Z1106033        TGGTTGTGGAAAGAGCTAGGCAAACACAAACGGCCACGAGTCTGTACCAAAGAAGAGTTC 8760
BEH819966       TGGTTGTGGAAAGAGCTAGGCAAACACAAACGGCCACGAGTCTGTACCAAAGAAGAGTTC 8760
SSABr           TGGTTGTGGAAAGAGCTAGGCAAACACAAACGGCCACGAGTCTGTACCAAAGAAGAGTTC 8760
```

```
Beh815744          TGGTTGTGGAAAGAGCTAGGCAAACACAAACGGCCACGAGTCTGTACCAAAGAAGAGTTC 8760
BEH818995          TGGTTGTGGAAAGAGCTAGGCAAACACAAACGGCCACGAGTCTGTACCAAAGAAGAGTTC 8760
IbH30656           TGGCTATGGAAGGAGCTGGGAAAACGCAAGCGGCCACGTGTCTGCACCAAAGAAGAGTTC 8742
ArD128000          TGGCTATGGAAGGAGCTAGGAAAACGCAAGCGACCACGTGTCTGCACCAAAGAAGAGTTC 8760
ArD7117            TGGCTATGGAAGGAGCTGGGAAAACGCAAGCGGCCACGTGTCTGCACCAAAGAAGAGTTC 8760
ARD_41519          TGGCTATGGAAGGAGCTGGGAAAACGCAAGCGACCACGTGTCTGCACCAAAGAAGAGTTC 8760
ARD157995          TGGCTGTGGAAGGAGCTGGGGAAACGCAAGCGGCCACGCGTCTGCACCAAAGAAGAGTTT 8760
MR_766             TGGCTGTGGAAGGAGCTGGGGAAACGCAAGCGGCCACGCGTCTGCACCAAAGAAGAGTTT 8748
ARD158084          TGGCTGTGGAAGGAGCTGGGGAAACGCAAGCGGCCACGCGTCTGCACCAAAGAAGAGTTT 8760
ArB1362            TGGCTGTGGAAGGAGCTGGGAAAACGCAAGCGGCCACGCGTCTGCACCAAAGAAGAGTTC 8760
ARB13565           TGGCTGTGGAAGGAGTTGGGGAAACGCAAGCGGCCACGTGTCTGCACCAAAGAAGAGTTC 8760
ARB7701            TGGCTGTGGAAGGAGTTGGGGAAACGCAAGCGGCCACGTGTCTGCACCAAAGAAGAGTTC 8760
ARB15076           TGGCTGTGGAAGGAGCTGGGGAAACGCAAGCGGCCACGTGTCTGCACCAAAGAAGAGTTC 8742
                   *** * ***** *** * ** **** *** ** ***** ** ** **************

P6-740             ATCAATAAGGTTCGCAGCAATGCAGCACTGGGGGCAATATTTGAAGAGGAGAAAGAATGG 8820
CPC0740            ATCAACAAGGTTCGTAGCAATGCAGCATTAGGGGCAATATTTGAAGAGGAAAAAGAGTGG 8820
YAP2007            ATCAACAAGGTTCGTAGCAATGCAGCATTAGGGGCAATATTTGAAGAGGAAAAAGAGTGG 8820
FSS13025           ATCAACAAGGTTCGTAGCAACGCAGCATTAGGGGCAATATTTGAAGAGGAAAAAGAGTGG 8820
PLCal_ZV           ATCAACAAGGTTCGTAGCAATGCAGCATTAGGGGCAATATTTGAAGAGGAAAAAGAGTGG 8709
SV0127/14          ATCAACAAGGTTCGTAGCAATGCAGCATTAGGGGCAATATTTGAAGAGGAAAAAGAGTGG 8820
8375               ATCAACAAGGTTCGTAGCAATGCAGCATTAGGGGCAATATTTGAAGAGGAAAAAGAGTGG 8820
103344             ATCAACAAGGTTCGTAGCAATGCAGCATTAGGGGCAATATTTGAAGAGGAAAAAGAGTGG 8820
BrasilZKV2015      ATCAACAAGGTTCGTAGCAATGCAGCATTAGGGGCAATATTTGAAGAGGAAAAAGAGTGG 8820
GD01               ATCAACAAGGTTCGTAGCAATGCAGCATTAGGGGCAATATTTGAAGAGGAAAAAGAGTGG 8820
Martinique         ATCAACAAGGTTCGTAGCAATGCAGCATTAGGGGCAATATTTGAAGAGGAAAAAGAGTGG 8820
NatalRGN           ATCAACAAGGTTCGTAGCAATGCAGCATTAGGGGCAATATTTGAAGAGGAAAAAGAGTGG 8820
HPF2013            ATCAACAAGGTTCGTAGCAATGCAGCATTAGGGGCAATATTTGAAGAGGAAAAAGAGTGG 8820
SPH2015            ATCAACAAGGTTCGTAGCAATGCAGCATTAGGGGCAATATTTGAAGAGGAAAAAGAGTGG 8820
Haiti2014          ATCAACAAGGTTCGTAGCAATGCAGCATTAGGGGCAATATTTGAAGAGGAAAAAGAGTGG 8820
PRVABC59           ATCAACAAGGTTCGTAGCAATGCAGCATTAGGGGCAATATTTGAAGAGGAAAAAGAGTGG 8820
BeH819015          ATCAACAAGGTTCGTAGCAATGCAGCATTAGGGGCAATATTTGAAGAGGAAAAAGAGTGG 8820
Z1106033           ATCAACAAGGTTCGTAGCAATGCAGCATTAGGGGCAATATTTGAAGAGGAAAAAGAGTGG 8820
BEH819966          ATCAACAAGGTTCGTAGCAATGCAGCATTAGGGGCAATATTTGAAGAGGAAAAAGAGTGG 8820
SSABr              ATCAACAAGGTTCGTAGCAATGCAGCATTAGGGGCAATATTTGAAGAGGAAAAAGAGTGG 8820
Beh815744          ATCAACAAGGTTCGTAGCAATGCAGCATTAGGGGCAATATTTGAAGAGGAAAAAGAGTGG 8820
BEH818995          ATCAACAAGGTTCGTAGCAATGCAGCATTAGGGGCAATATTTGAAGAGGAAAAAGAGTGG 8820
IbH30656           ATCAATAAGGTGCGCAGCAATGCAGCACTGGGAGCAATATTTGAAGAGGAAAAAGAATGG 8802
ArD128000          ATCAATAAGGTGCGCAGCAATGCAGCACTGGGAGCAATATTTGAAGAGGAAAAAGAATGG 8820
ArD7117            ATTAATAAGGTGCGCAGCAATGCAGCACTGGGAGCAATATTTGAAGAGGAAAAAGAATGG 8820
ARD_41519          ATTAATAAGGTGCGCAGCAATGCAGCACTGGGAGCAATATTTGAAGAGGAAAAAGAATGG 8820
ARD157995          ATCAACAAGGTGCGCAGCAATGCAGCACTGGGAGCAATATTTGAAGAGGAAAAAGAATGG 8820
MR_766             ATCAACAAGGTGCGCAGCAATGCAGCACTGGGAGCAATATTTGAAGAGGAAAAAGAATGG 8808
ARD158084          ATCAACAAGGTGCGCAGCAATGCAGCACTGGGAGCAATATTTGAAGAGGAAAAAGAATGG 8820
ArB1362            ATCAACAAGGTGCGCAGCAATGCAGCGCTGGGAGCAATATTTGAAGAGGAAAAAGAATGG 8820
ARB13565           ATCAACAAGGTGCGCAGCAATGCAGCACTGGGAGCAATATTTGAAGAGGAAAAAGAATGG 8820
ARB7701            ATCAACAAGGTGCGCAGCAATGCAGCACTGGGAGCAATATTTGAAGAGGAAAAAGAATGG 8820
ARB15076           ATCAACAAGGTGCGCAGCAATGCAGCACTGGGAGCAATATTTGAAGAGGAAAAAGAATGG 8802
                   ** ** ***** ** ***** *****  * ** ***************** ***** ***

P6-740             AAGACTGCAGTGGAAGCTGTGAACGATCCAAGGTTCTGGGCCCTAGTGGACAAGGAAAGA 8880
CPC0740            AAGACTGCAGTGGAAGCTGTGAATGATCCAAGGTTCTGGGCTCTAGTGGACAAGGAAAGA 8880
YAP2007            AAGACTGCAGTGGAAGCTGTGAACGATCCAAGGTTCTGGGCTCTAGTGGACAAGGAAAGA 8880
FSS13025           AAGACTGCAGTGGAAGCTGTGAACGATCCAAGGTTCTGGGCTCTAGTGGACAAGGAAAGA 8880
PLCal_ZV           AAGACTGCAGTGGAAGCTGTGAACGATCCAAGGTTCTGGGCTCTAGTGGACAAGGAAAGA 8769
SV0127/14          AAGACCGCAGTGGAAGCTGTGAACGATCCAAGGTTCTGGGCTCTAGTGGACAAGGAAAGA 8880
8375               AAGACTGCAGTGGAAGCTGTGAACGATCCAAGGTTCTGGGCTCTAGTGGACAAGGAAAGA 8880
103344             AAGACTGCAGTGGAAGCTGTGAACGATCCAAGGTTCTGGGCTCTAGTGGACAAGGAAAGA 8880
BrasilZKV2015      AAGACTGCAGTGGAAGCTGTGAACGATCCAAGGTTCTGGGCTCTAGTGGACAAGGAAAGA 8880
GD01               AAGACTGCAGTGGAAGCTGTGAACGATCCAAGGTTCTGGGCTCTAGTGGACAAGGAAAGA 8880
Martinique         AAGACTGCAGTGGAAGCTGTGAACGATCCAAGGTTCTGGGCTCTAGTGGACAAGGAAAGA 8880
NatalRGN           AAGACTGCAGTGGAAGCTGTGAACGATCCAAGGTTCTGGGCTCTAGTGGACAAGGAAAGA 8880
HPF2013            AAGACTGCAGTGGAAGCTGTGAACGATCCAAGGTTCTGGGCTCTAGTGGACAAGGAAAGA 8880
SPH2015            AAGACTGCAGTGGAAGCTGTGAACGATCCAAGGTTCTGGGCTCTAGTGGACAAGGAAAGA 8880
Haiti2014          AAGACTGCAGTGGAAGCTGTGAACGATCCAAGGTTCTGGGCTCTAGTGGACAAGGAAAGA 8880
PRVABC59           AAGACTGCAGTGGAAGCTGTGAACGATCCAAGGTTCTGGGCTCTAGTGGACAAGGAAAGA 8880
BeH819015          AAGACTGCAGTGGAAGCTGTGAACGATCCAAGGTTCTGGGCTCTAGTGGACAAGGAAAGA 8880
Z1106033           AAGACTGCAGTGGAAGCTGTGAACGATCCAAGGTTCTGGGCTCTAGTGGACAAGGAAAGA 8880
```

```
BEH819966       AAGACTGCAGTGGAAGCTGTGAACGATCCAAGGTTCTGGGCTCTAGTGGATAAGGAAAGA 8880
SSABr           AAGACTGCAGTGGAAGCTGTGAACGATCCAAGGTTCTGGGCTCTAGTGGATAAGGAAAGA 8880
Beh815744       AAGACTGCAGTGGAAGCTGTGAACGATCCAAGGTTCTGGGCTCTAGTGGATAAGGAAAGA 8880
BEH818995       AAGACTGCAGTGGAAGCTGTGAACGATCCAAGGTTCTGGGCTCTAGTGGATAAGGAAAGA 8880
IbH30656        AAGACAGCTGTAGAAGCTGTGAATGATCCGAGATTTTGGGCTCTAGTGGACAAGGAAAGA 8862
ArD128000       AAGACAGCTGTAGAAGCTGTGAATGATCCAAGATTTTGGGCTCTAGTGGACAAGGAAAGA 8880
ArD7117         AAGACAGCTGTAGAAGCTGTGAATGATCCAAGATTTTGGGCTCTAGTGGACAAGGAAAGA 8880
ARD_41519       AAGACAGCTGTAGAAGCTGTGAATGATCCAAGATTTTGGGCTCTAGTGGACAAGGAAAGA 8880
ARD157995       AAGACGGCTGTGGAAGCTGTGAATGATCCAAGGTTTTGGGCCCTAGTGGATAGGGAGAGA 8880
MR_766          AAGACGGCTGTGGAAGCTGTGAATGATCCAAGGTTTTGGGCCCTAGTGGATAGGGAGAGA 8868
ARD158084       AAGACGGCTGTGGAAGCTGTGAATGATCCAAGGTTTTGGGCCCTAGTGGATAGGGAGAGA 8880
ArB1362         AAGACGGCCGTGGAAGCTGTGAATGATCCAAGGTTTTGGGCCCTAGTGGATAAGGAGAGA 8880
ARB13565        AAGACGGCCGTGGAGGCTGTGAATGATCCAAGGTTTTGGGCTCTAGTGGACAAGGAAAGA 8880
ARB7701         AAGACGGCCGTGGAGGCTGTGAATGATCCAAGGTTTTGGGCTCTAGTGGACAAGGAAAGA 8880
ARB15076        AAGACGGCCGTGGAAGCTGTGAATGATCCAAGGTTTTGGGCCCTAGTGGACAAGGAGAGA 8862
                ***** ** ** ** ******** ***** ** ** ***** ******** * *** ***

P6-740          GAGCACCACTTGAGAGGAGAGTGTCAGAGCTGTGTGTACAACATGATGGGAAAAAGAGAA 8940
CPC0740         GAGCATCACCTGAGAGGAGAGTGTCAGAGCTGTGTGTACAACATGATGGGAAAAAGAGAA 8940
YAP2007         GAGCACCACCTGAGAGGAGAGTGTCAGAGCTGTGTGTACAACATGATGGGAAAAAGAGAA 8940
FSS13025        GAGCACCACCTGAGAGGAGAGTGCCAGAGCTGTGTGTACAACATGATGGGAAAAAGAGAA 8940
PLCal_ZV        GAGCACCACCTGAGAGGAGAGTGCCAGAGCTGTGTGTACAACATGATGGGAAAAAGAGAA 8829
SV0127/14       GAGCACCACCTGAGAGGAGAGTGCCAGAGCTGTGTGTACAACATGATGGGAAAAAGAGAA 8940
8375            GAGCACCACCTGAGAGGAGAGTGCCAGAGTTGTGTGTACAACATGATGGGAAAAAGAGAA 8940
103344          GAGCACCACCTGAGAGGAGAGTGCCAGAGTTGTGTGTACAACATGATGGGAAAAAGAGAA 8940
BrasilZKV2015   GAGCACCACCTGAGAGGAGAGTGCCAGAGTTGTGTGTACAACATGATGGGAAAAAGAGAA 8940
GD01            GAGCACCACCTGAGAGGAGAGTGCCAGAGTTGTGTGTACAACATGATGGGAAAAAGAGAA 8940
Martinique      GAGCACCACCTGAGAGGAGAGTGCCAGAGTTGTGTGTACAACATGATGGGAAAAAGAGAA 8940
NatalRGN        GAGCACCACCTGAGAGGAGAGTGCCAGAGTTGTGTGTACAACATGATGGGAAAAAGAGAA 8940
HPF2013         GAGCACCACCTGAGAGGAGAGTGCCAGAGTTGTGTGTACAACATGATGGGAAAAAGAGAA 8940
SPH2015         GAGCACCACCTGAGAGGAGAGTGCCAGAGTTGTGTGTACAACATGATGGGAAAAAGAGAA 8940
Haiti2014       GAGCACCACCTGAGAGGAGAGTGCCAGAGTTGTGTGTACAACATGATGGGAAAAAGAGAA 8940
PRVABC59        GAGCACCACCTGAGAGGAGAGTGCCAGAGCTGTGTGTACAACATGATGGGAAAAAGAGAA 8940
BeH819015       GAGCACCACCTGAGAGGAGAGTGCCAGAGTTGTGTGTATAACATGATGGGAAAAAGAGAA 8940
Z1106033        GAGCACCACCTGAGAGGAGAGTGCCAGAGTTGTGTGTACAACATGATGGGAAAAAGAGAA 8940
BEH819966       GAGCACCACCTGAGAGGAGAGTGCCAGAGTTGTGTGTACAACATGATGGGAAAAAGAGAA 8940
SSABr           GAGCACCACCTGAGAGGAGAGTGCCAGAGTTGTGTGTACAACATGATGGGAAAAAGAGAA 8940
Beh815744       GAGCACCACCTGAGAGGAGAGTGCCAGAGTTGTGTGTACAACATGATGGGAAAAAGAGAA 8940
BEH818995       GAGCACCACCTGAGAGGAGAGTGCCAGAGTTGTGTGTACAACATGATGGGAAAAAGAGAA 8940
IbH30656        GAACACCACCTGAGAGGAGAGTGTCACAGCTGTGTGTACAACATGATGGGAAAAAGAGAA 8922
ArD128000       GAACACCACCTGAGAGGAGAGTGTCACAGCTGTGTGTACAACATGATGGGTAAAAGAGAA 8940
ArD7117         GAACACCACCTGAGAGGAGAGTGTCACAGCTGTGTGTACAACATGATGGGTAAAAGAGAA 8940
ARD_41519       GAACACCACCTGAGAGGAGAGTGTCATAGCTGTGTGTACAACATGATGGGCAAAAGAGAA 8940
ARD157995       GAACACCACCTGAGAGGAGAGTGTCACAGCTGTGTGTACAACATGATGGGAAAAAGAGAA 8940
MR_766          GAACACCACCTGAGAGGAGAGTGTCACAGCTGTGTGTACAACATGATGGGAAAAAGAGAA 8928
ARD158084       GAACACCACCTGAGAGGAGAGTGTCACAGCTGTGTGTACAACATGATGGGAAAAAGAGAA 8940
ArB1362         GAGCATCACCTAAGAGGAGAGTGTCATAGCTGTGTGTACAACATGATGGGAAAAAGAGAG 8940
ARB13565        GAACATCACCTGAGAGGAGAGTGTCATAGCTGTGTGTACAACATGATGGGAAAAAGAGAG 8940
ARB7701         GAACATCACCTGAGAGGAGAGTGCCATAGCTGTGTGTACAATATGATGGGAAAAAGAGAG 8940
ARB15076        GAACATCACCTGAGAGGAGAGTGCCATAGCTGTGTGTACAATATGATGGGAAAAAGAGAG 8922
                ** ** *** * *********** ** ** ******** ** ******** ********

P6-740          AAGAAGCAAGGGGAATTTGGAAAGGCCAAGGGCAGCCGCGCCATTTGGTACATGTGGCTA 9000
CPC0740         AAGAAACAAGGGGAATTTGGAAAGGCCAAGGGCAGCCGCGCCATCTGGTATATGTGGCTA 9000
YAP2007         AAGAAACAAGGGGAATTTGGAAAGGCCAAGGGCAGCCGCGCCATCTGGTATATGTGGCTA 9000
FSS13025        AAGAAACAAGGGGAATTTGGAAAGGCCAAGGGCAGCCGCGCCATCTGGTACATGTGGCTA 9000
PLCal_ZV        AAGAAACAAGGGGAATTTGGAAAGGCCAAGGGCAGCCGCGCCATCTGGTATATGTGGCTA 8889
SV0127/14       AAGAAACAAGGGGAATTTGGAAAGGCCAAGGGCAGCCGCGCCATCTGGTATATGTGGCTA 9000
8375            AAGAAACAAGGGGAATTTGGAAAGGCCAAGGGCAGCCGCGCCATCTGGTATATGTGGCTA 9000
103344          AAGAAACAAGGGGAATTTGGAAAGGCCAAGGGCAGCCGCGCCATCTGGTATATGTGGCTA 9000
BrasilZKV2015   AAGAAACAAGGGGAATTTGGAAAGGCCAAGGGCAGCCGCGCCATCTGGTATATGTGGCTA 9000
GD01            AAGAAACAAGGGGAATTTGGAAAGGCCAAGGGCAGCCGCGCCATCTGGTATATGTGGCTA 9000
Martinique      AAGAAACAAGGGGAATTTGGAAAGGCCAAGGGCAGCCGCGCCATCTGGTATATGTGGCTA 9000
NatalRGN        AAGAAACAAGGGGAATTTGGAAAGGCCAAGGGCAGCCGCGCCATCTGGTATATGTGGCTA 9000
HPF2013         AAGAAACAAGGGGAATTTGGAAAGGCCAAGGGCAGCCGCGCCATCTGGTATATGTGGCTA 9000
SPH2015         AAGAAACAAGGGGAATTTGGAAAGGCCAAGGGCAGCCGCGCCATCTGGTATATGTGGCTA 9000
Haiti2014       AAGAAACAAGGGGAATTTGGAAAGGCCAAGGGCAGCCGCGCCATCTGGTATATGTGGCTA 9000
PRVABC59        AAGAAACAAGGGGAATTTGGAAAGGCCAAGGGCAGCCGCGCCATCTGGTATATGTGGCTA 9000
```

```
BeH819015           AAGAAACAAGGGGAATTTGGAAAGGCCAAGGGCAGCCGCGCCATCTGGTATATGTGGCTA 9000
Z1106033            AAGAAACAAGGGGAATTTGGAAAGGCCAAGGGCAGCCGCGCCATCTGGTATATGTGGCTA 9000
BEH819966           AAGAAACAAGGGGAATTTGGAAAGGCCAAGGGCAGCCGCGCCATCTGGTATATGTGGCTA 9000
SSABr               AAGAAACAAGGGGAATTTGGAAAGGCCAAGGGCAGCCGCGCCATCTGGTATATGTGGCTA 9000
Beh815744           AAGAAACAAGGGGAATTTGGAAAGGCCAAGGGCAGCCGCGCCATCTGGTATATGTGGCTA 9000
BEH818995           AAGAAACAAGGGGAATTTGGAAAGGCCAAGGGCAGCCGCGCCATCTGGTATATGTGGCTA 9000
IbH30656            AAGAAGCAAGGAGAATTCGGGAAAGCAAAAGGCAGCCGCGCAATCTGGTACATGTGGTTG 8982
ArD128000           AAGAAGCAAGGAGAATTCGGGAAAGCAAAAGGCAGCCGCGCAATCTGGTACATGTGGTTG 9000
ArD7117             AAGAAGCAAGGAGAATTCGGGAAAGCAAAAGGCAGCCGCGCAATCTGGTACATGTGGTTG 9000
ARD_41519           AAGAAGCAAGGAGAATTCGGGAAAGCAAAAGGCAGCCGCGCAATCTGGTACATGTGGTTG 9000
ARD157995           AAGAAGCAAGGAGAGTTCGGGAAAGCAAAAGGTAGCCGCGCCATCTGGTACATGTGGTTG 9000
MR_766              AAGAAGCAAGGAGAGTTCGGGAAAGCAAAAGGTAGCCGCGCCATCTGGTACATGTGGTTG 8988
ARD158084           AAGAAGCAAGGAGAGTTCGGGAAAGCAAAAGGTAGCCGCGCCATCTGGTACATGTGGTTG 9000
ArB1362             AAGAAGcAAGGAGAATTCGGGAAAGCAAAAGGCAGCCGCGCCATCTGGTACATGTGGTTG 9000
ARB13565            AAGAAGCAAGGAGAATTCGGAAAAGCAAAAGGCAGCCGCGCCATCTGGTACATGTGGTTG 9000
ARB7701             AAGAAGCAAGGAGAATTCGGAAAAGCAAAAGGCAGCCGCGCCATCTGGTACATGTGGTTG 9000
ARB15076            AAGAAGCAAGGAGAATTCGGAAAAGCAAAAGGCAGCCGCGCCATCTGGTACATGTGGTTG 8982
                    ***** ***** ** ** ** ** ** ** ** ******** ** ***** ****** *

P6-740              GGGGCTAGATTTCTAGAGTTTGAAGCCCTTGGATTCTTGAACGAGGATCACTGGATGGGG 9060
CPC0740             GGGGCTAGATTCCTAGAGTTCGAAGCCCTTGGATTCTTGAATGAGGATCATTGGATGGGG 9060
YAP2007             GGGGCTAGATTCCTGGAGTTCGAAGCCCTTGGATTCTTGAACGAGGATCACTGGATGGGG 9060
FSS13025            GGGGCTAGATTTCTAGAGTTCGAAGCCCTTGGATTCTTGAACGAGGATCACTGGATGGGG 9060
PLCal_ZV            GGGGCTAGATTTCTAGAGTTCGAAGCCCTTGGATTCTTGAACGAGGATCACTGGATGGGG 8949
SV0127/14           GGGGCTAGATTTCTAGAGTTCGAAGCCCTTGGATTCTTAAATGAGGATCACTGGATGGGG 9060
8375                GGGGCTAGATTTCTAGAGTTCGAAGCCCTTGGATTCTTGAACGAGGATCACTGGATGGGG 9060
103344              GGGGCTAGATTTCTAGAGTTCGAAGCCCTTGGATTCTTGAACGAGGATCACTGGATGGGG 9060
BrasilZKV2015       GGGGCTAGATTTCTAGAGTTCGAAGCCCTTGGATTCTTGAACGAGGATCACTGGATGGGG 9060
GD01                GGGGCTAGATTTCTAGAGTTCGAAGCCCTTGGATTCTTGAACGAGGATCACTGGATGGGG 9060
Martinique          GGGGCTAGATTTCTAGAGTTCGAAGCCCTTGGATTCTTGAACGAGGATCACTGGATGGGG 9060
NatalRGN            GGGGCTAGATTTCTAGAGTTCGAAGCCCTTGGATTCTTGAACGAGGATCACTGGATGGGG 9060
HPF2013             GGGGCTAGATTTCTAGAGTTCGAAGCCCTTGGATTCTTGAACGAGGATCACTGGATGGGG 9060
SPH2015             GGGGCTAGATTTCTAGAGTTCGAAGCCCTTGGATTCTTGAACGAGGATCACTGGATGGGG 9060
Haiti2014           GGGGCTAGATTTCTAGAGTTCGAAGCCCTTGGATTCTTGAACGAGGATCACTGGATGGGG 9060
PRVABC59            GGGGCTAGATTTCTAGAGTTCGAAGCCCTTGGATTCTTGAACGAGGATCACTGGATGGGG 9060
BeH819015           GGGGCTAGATTTCTAGAGTTCGAAGCCCTTGGATTCTTGAACGAGGATCACTGGATGGGG 9060
Z1106033            GGGGCTAGATTTCTAGAGTTCGAAGCCCTTGGATTCTTGAACGAGGATCACTGGATGGGG 9060
BEH819966           GGGGCTAGATTTCTAGAGTTCGAAGCCCTTGGATTCTTGAACGAGGATCACTGGATGGGG 9060
SSABr               GGGGCTAGATTTCTAGAGTTCGAAGCCCTTGGATTCTTGAACGAGGATCACTGGATGGGG 9060
Beh815744           GGGGCTAGATTTCTAGAGTTCGAAGCCCTTGGATTCTTGAACGAGGATCACTGGATGGGG 9060
BEH818995           GGGGCTAGATTTCTAGAGTTCGAAGCCCTTGGATTCTTGAACGAGGATCACTGGATGGGG 9060
IbH30656            GGAGCCAGATTTCTGGAGTTTGAGGCTCTTGGATTCTTGAATGAGGACCATTGGATGGGA 9042
ArD128000           GGAGCCAGATTTTTGGAGTTTGAAGCTCTTGGATTCTTGAACGAGGATCACTGGATGGGA 9060
ArD7117             GGAGCCAGATTTTTGGAGTTTGAAGCTCTTGGATTCTTGAACGAGGACCACTGGATGGGA 9060
ARD_41519           GGAGCCAGATTTTTGGAGTTTGAAGCTCTTGGGTTCTTGAACGAGGACCACTGGATGGGG 9060
ARD157995           GGAGCCAGGTTTCTGGAGTTTGAATCACTGGGGTTTCTGAATGAAGATCACTGGATGGGA 9060
MR_766              GGAGCCAGATTCTTGGAGTTTGAAGCCCTTGGATTCTTGAACGAGGACCATTGGATGGGA 9048
ARD158084           GGAGCCAGATTCTTGGAGTTTGAAGCCCTTGGATTCTTGAACGAGGACCATTGGATGGGA 9060
ArB1362             GGAGCCAGGTTTCTGGAGTTTGAATCACTGGGGTTTCTGAATGAAGATCACTGGATGGGA 9060
ARB13565            GGAGCCAGATTCTTGGAGTTTGAAGCCCTTGGATTCTTGAATGAGGACCATTGGATGGGA 9060
ARB7701             GGAGCCAGATTCTTGGAGTTTGAAGCCCTTGGATTCTTGAATGAGGACCATTGGATGGGA 9060
ARB15076            GGAGCCAGATTCTTGGAGTTTGAAGCCCTTGGATTCTTGAATGAGGACCATTGGATGGGA 9042
                    ** ** ** **  * ***** **  * ** ** **  * ** ** ** ** ********

P6-740              AGAGAGAATTCAGGAGGTGGTGTTGAAGGGCTGGGATTACAAAGACTTGGATATGTTCTA 9120
CPC0740             AGAGAGAATTCAGGAGGTGGTGTTGAAGGACTGGGATTACAAAGACTCGGATATGTCCTA 9120
YAP2007             AGAGAGAATTCAGGAGGTGGTGTTGAAGGGCTGGGATTACAAAGACTCGGATATGTCCTA 9120
FSS13025            AGAGAGAATTCAGGAGGTGGTGTTGAAGGGCTAGGATTACAAAGACTCGGATATGTCTTA 9120
PLCal_ZV            AGAGAGAACTCAGGAGGTGGTGTTGAAGGGCTGGGATTACAAAGACTCGGATATGTCCTA 9009
SV0127/14           AGAGAGAACTCAGGAGGTGGTGTTGAAGGGCTGGGATTACAAAGACTCGGATATGTCCTA 9120
8375                AGAGAGAACTCAGGAGGTGGTGTTGAAGGGCTGGGATTACAAAGACTCGGATATGTCCTA 9120
103344              AGAGAGAACTCAGGAGGTGGTGTTGAAGGGCTGGGATTACAAAGACTCGGATATGTCCTA 9120
BrasilZKV2015       AGAGAGAACTCAGGAGGTGGTGTTGAAGGGCTGGGATTACAAAGACTCGGATATGTCCTA 9120
GD01                AGAGAGAACTCAGGAGGTGGTGTTGAAGGGCTGGGATTACAAAGACTCGGATATGTCCTA 9120
Martinique          AGAGAGAACTCAGGAGGTGGTGTTGAAGGGCTGGGATTACAAAGACTCGGATATGTCCTA 9120
NatalRGN            AGAGAGAACTCAGGAGGTGGTGTTGAAGGGCTGGGATTACAAAGACTCGGATATGTCCTA 9120
HPF2013             AGAGAGAACTCAGGAGGTGGTGTTGAAGGGCTGGGATTACAAAGACTCGGATATGTCCTA 9120
SPH2015             AGAGAGAACTCAGGAGGTGGTGTTGAAGGGCTGGGATTACAAAGACTCGGATATGTCCTA 9120
```

```
Haiti2014        AGAGAGAACTCAGGAGGTGGTGTTGAAGGGCTGGGATTACAAAGACTCGGATATGTCCTA 9120
PRVABC59         AGAGAGAACTCAGGAGGTGGTGTTGAAGGGCTGGGATTACAAAGACTCGGATATGTCCTA 9120
BeH819015        AGAGAGAACTCAGGAGGTGGTGTTGAAGGGCTGGGATTACAAAGACTCGGATATGTCCTA 9120
Z1106033         AGAGAGAACTCAGGAGGTGGTGTTGAAGGGCTGGGATTACAAAGACTCGGATATGTCCTA 9120
BEH819966        AGAGAGAACTCAGGAGGTGGTGTTGAAGGGCTGGGATTACAAAGACTCGGATATGTCCTA 9120
SSABr            AGAGAGAACTCAGGAGGTGGTGTTGAAGGGCTGGGATTACAAAGACTCGGATATGTCCTA 9120
Beh815744        AGAGAGAACTCAGGAGGTGGTGTTGAAGGGCTGGGATTACAAAGACTCGGATATGTCCTA 9120
BEH818995        AGAGAGAACTCAGGAGGTGGTGTTGAAGGGCTGGGATTACAAAGACTCGGATATGTCCTA 9120
IbH30656         AGAGAAAACTCAGGAGGTGGCGTTGAAGGGCTAGGACTGCAAAGGCTTGGATACATTCTA 9102
ArD128000        AGAGAAAACTCAGGAGGTGGTGTTGAAGGGCTAGGACTGCAAAGGCTTGGATATATCCTA 9120
ArD7117          AGAGAAAACTCAGGAGGTGGCGTTGAAGGGCTAGGACTGCAAAGGCTTGGATATATCCTA 9120
ARD_41519        AGAGAAAACTCAGGAGGTGGCGTTGAAGGGCTAGGACTGCAAAGGCTTGGATATATCCTA 9120
ARD157995        AGAGAGAACTCTGGAGGCGGAGTTGAAGGACTGGGACTGCAGAGACTGGGCTATGTCCTT 9120
MR_766           AGAGAAAACTCAGGAGGTGGAGTCGAAGGGTTAGGATTGCAAAGACTTGGATACATTCTA 9108
ARD158084        AGAGAGAACTCTGGAGGCGGAGTTGAAGGACTGGGACTGCAGAGACTGGGCTATGTCCTT 9120
ArB1362          AGAGAGAACTCAGGAGGTGGTGTCGAAGGGCTAGGACTGCAAAGACTTGGATATATTCTA 9120
ARB13565         AGAGAAAACTCAGGAGGTGGTGTCGAAGGGCTAGGACTGCAAAGACTTGGATACATTCTA 9120
ARB7701          AGAGAAAACTCAGGAGGTGGTGTCGAAGGGCTAGGACTGCAAAGACTTGGATACATTCTA 9120
ARB15076         AGAGAAAACTCAGGAGGTGGTGTCGAAGGGCTAGGACTGCAAAGACTTGGATACATTCTA 9102
                 ***** ** ** ***** ** ** *****  * *** * ** ** ** ** **  *  *

P6-740           GAAGAAATGAGCCGCACACCAGGAGGAAAGATGTATGCAGATGATACCGCTGGCTGGGAC 9180
CPC0740          GAAGAGATGAGTCGCATACCAGGAGGAAGGATGTATGCAGATGATACTGCTGGCTGGGAC 9180
YAP2007          GAAGAGATGAGTCGCATACCAGGAGGAAGGATGTATGCTGATGACACAGCTGGCTGGGAC 9180
FSS13025         GAAGAGATGAGTCGCATACCAGGAGGAAGGATGTATGCAGATGATACTGCTGGCTGGGAC 9180
PLCal_ZV         GAAGAGATGAGTCGCATACCAGGAGGAAGGATGTATGCAGATGACACTGCTGGCTGGGAC 9069
SV0127/14        GAAGAGATGAGTCGCATACCAGGAGGAAGGATGTATGCAGATGACACTGCTGGCTGGGAC 9180
8375             GAAGAGATGAGTTGCATACCAGGAGGAAGGATGTATGCAGATGACACTGCTGGCTGGGAC 9180
103344           GAAGAGATGAGTTGCATACCAGGAGGAAGGATGTATGCAGATGACACTGCTGGCTGGGAC 9180
BrasilZKV2015    GAAGAGATGAGTCGCATACCAGGAGGAAGGATGTATGCAGATGACACTGCTGGCTGGGAC 9180
GD01             GAAGAGATGAGTCGTATACCAGGAGGAAGGATGTATGCAGATGACACTGCTGGCTGGGAC 9180
Martinique       GAAGAGATGAGTCGCATACCAGGAGGAAGGATGTATGCAGATGACACTGCTGGCTGGGAC 9180
NatalRGN         GAAGAGATGAGTCGCATACCAGGAGGAAGGATGTATGCAGATGACACTGCTGGCTGGGAC 9180
HPF2013          GAAGAGATGAGTCGCATACCAGGAGGAAGGATGTATGCAGATGACACTGCTGGCTGGGAC 9180
SPH2015          GAAGAGATGAGTCGCATACCAGGAGGAAGGATGTATGCAGATGACACTGCTGGCTGGGAC 9180
Haiti2014        GAAGAGATGAGTCGCATACCAGGAGGAAGGATGTATGCAGATGACACTGCTGGCTGGGAC 9180
PRVABC59         GAAGAGATGAGTCGTATACCAGGAGGAAGGATGTATGCAGATGACACTGCTGGCTGGGAC 9180
BeH819015        GAAGAGATGAGTCGTATACCAGGAGGAAGGATGTATGCAGATGACACTGCTGGCTGGGAC 9180
Z1106033         GAAGAGATGAGTCGTATACCAGGAGGAAGGATGTATGCAGATGACACTGCTGGCTGGGAC 9180
BEH819966        GAAGAGATGAGTCGTATACCAGGAGGAAGGATGTATGCAGATGACACTGCTGGCTGGGAC 9180
SSABr            GAAGAGATGAGTCGTATACCAGGAGGAAGGATGTATGCAGATGACACTGCTGGCTGGGAC 9180
Beh815744        GAAGAGATGAGTCGTATACCAGGAGGAAGGATGTATGCAGATGACACTGCTGGCTGGGAC 9180
BEH818995        GAAGAGATGAGTCGTATACCAGGAGGAAGGATGTATGCAGATGACACTGCTGGCTGGGAC 9180
IbH30656         GAAGAAATGAACCGGGCGCCAGGAGGAAAGATGTATGCAGATGACACCGCTGGCTGGGAT 9162
ArD128000        GAAGAAATGAACCGGGCACCAGGAGGAAAGATGTATGCAGATGACACCGCTGGCTGGGAC 9180
ArD7117          GAAGAAATGAACCGGGCACCAGGAGGAAAGATGTATGCAGATGACACCGCTGGCTGGGAC 9180
ARD_41519        GAAGAAATGAACCGGGCACCAGGAGGAAAGATGTATGCAGATGACACTGCTGGCTGGGAC 9180
ARD157995        GAGGAGATGAGCCAGGCACCAGGAGGGAAGATGTACGCAGATGACACTGCTGGCTGGGAC 9180
MR_766           GAAGAAATGAATCGGGCACCAGGAGGAAAGATGTACGCAGATGACACTGCTGGCTGGGAC 9168
ARD158084        GAGGAGATGAGCCAGGCACCAGGAGGGAAGATGTACGCAGATGACACTGCTGGCTGGGAC 9180
ArB1362          GAAGAAATGAATCGGGCACCAGGAGGAAAGATGTATGCAGATGACACCGCTGGTTGGGAT 9180
ARB13565         GAAGAAATGAATCGGGCACCAGGAGGAAAGATGTATGCAGATGACACCGCTGGCTGGGAC 9180
ARB7701          GAAGAAATGAATCGGGCACCAGGAGGAAAGATGTATGCAGATGACACCGCTGGCTGGGAC 9180
ARB15076         GAAGAAATGAATCGAGCACCAGGAGGAAAGATGTATGCAGATGACACCGCTGGCTGGGAT 9162
                 ** ** ****        ******** * ****** ** ***** ** ***** *****

P6-740           ACCCGCATCAGTAGGTTTGATCTGGAGAATGAAGCTCTGATCACCAACCAAATGGAGAAA 9240
CPC0740          ACCCGCATCAGCAGGTTTGATCTGGAGAATGAAGCTCTAATCACCAACCAAATGGAGAAA 9240
YAP2007          ACCCGCATCAGCAGGTTTGATCTGGAGAATGAAGCTCTAATCACCAACCAAATGGAGAAA 9240
FSS13025         ACCCGCATCAGCAGGTTTGATCTGGAGAATGAAGCTCTAATCACCAACCAAATGGAGAAA 9240
PLCal_ZV         ACCCGCATCAGCAGGTTTGATCTGGAGAATGAAGCTCTAATCACCAACCAAATGGAGAAA 9129
SV0127/14        ACCCGCATCAGCAGGTTTGATCTGGAGAATGAAGCTTTAATCACCAACCAAATGGAGAAA 9240
8375             ACCCGCATCAGCAGGTTTGATCTGGAGAATGAAGCTCTAATCACCAACCAAATGGAGAAA 9240
103344           ACCCGCATCAGCAGGTTTGATCTGGAGAATGAAGCTCTAATCACCAACCAAATGGAGAAA 9240
BrasilZKV2015    ACCCGCATCAGCAGGTTTGATCTGGAGAATGAAGCTCTAATCACCAACCAAATGGAGAAA 9240
GD01             ACCCGCATTAGCAGGTTTGATCTGGAGAATGAAGCTCTAATCACCAACCAAATGGAGAAA 9240
Martinique       ACCCGCATCAGCAGGTTTGATCTGGAGAATGAAGCTCTAATCACCAACCAAATGGAGAAA 9240
NatalRGN         ACCCGCATCAGCAGGTTCGATCTGGAGAATGAAGCTCTAATCACCAACCAAATGGAGAAA 9240
```

```
HPF2013            ACCCGCATCAGCAGGTTTGATCTGGAGAATGAAGCTCTAATCACCAACCAAATGGAGAAA 9240
SPH2015            ACCCGCATCAGCAGGTTTGATCTGGAGAATGAAGCTCTAATCACCAACCAAATGGAGAAA 9240
Haiti2014          ACCCGCATCAGCAGGTTTGATCTGGAGAATGAAGCTCTAATCACCAACCAAATGGAGAAA 9240
PRVABC59           ACCCGCATTAGCAGGTTTGATCTGGAGAATGAAGCTCTAATCACCAACCAAATGGAGAAA 9240
BeH819015          ACCCGCATTAGCAGGTTTGATCTGGAGAATGAAGCTCTAATCACCAACCAAATGGAGAAA 9240
Z1106033           ACCCGCATTAGCAGGTTTGATCTGGAGAATGAAGCTCTAATCACCAACCAAATGGAGAAA 9240
BEH819966          ACCCGCATCAGCAGGTTTGATCTGGAGAATGAAGCTCTAATCACCAACCAAATGGAAAAA 9240
SSABr              ACCCGCATCAGCAGGTTTGATCTGGAGAATGAAGCTCTAATCACCAACCAAATGGAAAAA 9240
Beh815744          ACCCGCATCAGCAGGTTTGATCTGGAGAATGAAGCTCTAATCACCAACCAAATGGAAAAA 9240
BEH818995          ACCCGCATCAGCAGGTTTGATCTGGAGAATGAAGCTCTAATCACCAACCAAATGGAAAAA 9240
IbH30656           ACCCGTATTAGCAGGTTTGATCTGGAGAATGAAGCCCTGATCACTAACCAGATGGAAGAA 9222
ArD128000          ACCCGTATTAGTAAGTTTGATCTAGAGAATGAAGCCCTGATCACTAACCAGATGGAAGAA 9240
ArD7117            ACCCGCATTAGCAAGTTTGATCTAGAGAATGAAGCCCTGATCACTAACCAGATGGAAGAA 9240
ARD_41519          ACCCGCATTAGCAAGTTTGATCTAGAGAATGAAGCCCTGATCACTAACCAGATGGAAGAA 9240
ARD157995          ACCCGCATTAGTAAGTTTGATCTGGAGAATGAAGCTTTGATTACCAACCAAATGGAGGAA 9240
MR_766             ACCCGCATTAGTAAGTTTGATCTGGAGAATGAAGCTCTGATTACCAACCAAATGGAGGAA 9228
ARD158084          ACCCGCATTAGTAAGTTTGATCTGGAGAATGAAGCTTTGATTACCAACCAAATGGAGGAA 9240
ArB1362            ACCCGCATTAGTAAGTTTGATCTGGAGAATGAAGCCTTGATTACCAACCAAATGGAGGAA 9240
ARB13565           ACCCGCATTAGTAAGTTTGATCTGGAGAATGAAGCCTTAATTACCAACCAAATGGAGGAA 9240
ARB7701            ACCCGCATTAGTAAGTTTGATCTGGAGAATGAAGCCTTAATTACCAACCAAATGGAGGAA 9240
ARB15076           ACCCGTATTAGTAAGTTTGATCTGGAGAATGAAGCCTTAATTACCAACCAAATGGAGGAA 9222
                   ***** ** ** * *** ***** ***********  * ** ** ***** *****  **

P6-740             GGGCACAGGGCCTTGGCGTTGGCCATAATCAAGTACACATACCAAAACAAAGTGGTAAAG 9300
CPC0740            GGGCACAGGGCCTTGGCATTGGCCATAATCAAGTACACATACCAAAACAAAGTGGTAAAG 9300
YAP2007            GGGCACAGGGCCTTGGCATTGGCCATAATCAAGTACACATACCAAAACAAAGTGGTAAAG 9300
FSS13025           GGGCACAGGGCCTTGGCATTGGCCATAATCAAGTACACATACCAAAACAAAGTGGTAAAG 9300
PLCal_ZV           GGGCACAGGGCCTTGGCATTGGCCATAATCAAGTACACATACCAAAACAAAGTGGTAAAG 9189
SV0127/14          GGGCACAGGGCCTTAGCATTGGCCATAATCAAGTACACATACCAAAACAAAGTGGTAAAG 9300
8375               GGGCACAGGGCCTTGGCATTGGCCATAATCAAGTACACATACCAAAACAAAGTGGTAAAG 9300
103344             GGGCACAGGGCCTTGGCATTGGCCATAATCAAGTACACATACCAAAACAAAGTGGTAAAG 9300
BrasilZKV2015      GGGCACAGGGCCTTGGCATTGGCCATAATCAAGTACACATACCAAAACAAAGTGGTAAAG 9300
GD01               GGGCACAGGGCCTTGGCATTGGCCATAATCAAGTACACATACCAAAACAAAGTGGTAAAG 9300
Martinique         GGGCACAGGGCCTTGGCATTGGCCATAATCAAGTACACATACCAAAACAAAGTGGTAAAG 9300
NatalRGN           GGGCATAGGGCCTTGGCATTGGCCATAATCAAGTACACATACCAAAACAAAGTGGTAAAG 9300
HPF2013            GGGCACAGGGCCTTGGCATTGGCCATAATCAAGTACACATACCAAAACAAAGTGGTAAAG 9300
SPH2015            GGGCACAGGGCCTTGGCATTGGCCATAATCAAGTACACATACCAAAACAAAGTGGTAAAG 9300
Haiti2014          GGGCACAGGGCCTTGGCATTGGCCATAATCAAGTACACATACCAAAACAAAGTGGTAAAG 9300
PRVABC59           GGGCACAGGGCCTTGGCATTGGCCATAATCAAGTACACATACCAAAACAAAGTGGTAAAG 9300
BeH819015          GGGCACAGGGCCTTGGCATTGGCCATAATCAAGTACACATACCAAAACAAAGTGGTAAAG 9300
Z1106033           GGGCACAGGGCCTTGGCATTGGCCATAATCAAGTACACATACCAAAACAAAGTGGTAAAG 9300
BEH819966          GGGCACAGGGCCTTGGCATTGGCCATAATCAAGTACACATACCAAAACAAAGTGGTAAAG 9300
SSABr              GGGCACAGGGCCTTGGCATTGGCCATAATCAAGTACACATACCAAAACAAAGTGGTAAAG 9300
Beh815744          GGGCACAGGGCCTTGGCATTGGCCATAATCAAGTACACATACCAAAACAAAGTGGTAAAG 9300
BEH818995          GGGCACAGGGCCTTGGCATTGGCCATAATCAAGTACACATACCAAAACAAAGTGGTAAAG 9300
IbH30656           GGGCACAGAGCTCTGGCGTTGGCCGTGATTAAATACACATACCAAAACAAAGTGGTGAAG 9282
ArD128000          GGGCACAGAGCTCTGGCGTTGGCCGTGATTAAATACACATACCAAAACAAAGTGGTGAAG 9300
ArD7117            GGGCACAGAGCTCTGGCGTTGGCCGTGATTAAATACACATACCAAAACAAAGTGGTGAAG 9300
ARD_41519          GGGCACAGAGCTCTGGCGTTGGCCGTGATTAAATACACATACCAAAACAAAGTGGTGAAG 9300
ARD157995          GGGCACAGAACTCTGGCGTTGGCCGTGATTAAATACACATACCAAAACAAAGTGGTGAAG 9300
MR_766             GGGCACAGAACTCTGGCGTTGGCCGTGATTAAATACACATACCAAAACAAAGTGGTGAAG 9288
ARD158084          GGGCACAGAACTCTGGCGTTGGCCGTGATTAAATACACATACCAAAACAAAGTGGTGAAG 9300
ArB1362            GGGCACAGAGCTCTGGCGTTGGCTGTGATTAAATACACATACCAAAACAAAGTGGTGAAG 9300
ARB13565           GGGCACAGAGCTCTGGCGTTGGCTGTGATTAAGTACACAAA---AAACAAAGTGGTGAAG 9297
ARB7701            GGGCACAGAGCTCTGGCGTTGGCTGTGATTAAGTACACAAA---AAACAAAGTGGTGAAG 9297
ARB15076           GGGCATAGAGCTCTAGCATTGGCTGTGATTAAGTACACATACCAAAACAAAGTGGTGAAG 9282
                   ***** **  *  * ** *****  * ** ** ****** *   ************ ***

P6-740             GTCCTTAGACCAGCTGAAAGAGGGAAGACAGTTATGGACATCATCTCAAGACAAGACCAA 9360
CPC0740            GTCCTTAGACCAGCTGAAAAAGGGAAGACAGTTATGGACATTATTTCAAGACAAGACCAA 9360
YAP2007            GTCCTCAGACCAGCTGAAAAAGGGAAGACAGTTATGGACATTATTTCAAGACAAGACCAA 9360
FSS13025           GTCCTTAGACCAGCTGAAAAAGGGAAGACAGTTATGGACATTATTTCAAGACAAGACCAA 9360
PLCal_ZV           GTCCTTAGACCAGCTGAAAAAGGGAAGACAGTTATGGACATTATTTCAAGACAAGACCAA 9249
SV0127/14          GTCCTTAGACCAGCTGAAAAAGGGAAGACAGTTATGGACATTATTTCAAGACAAGACCAA 9360
8375               GTCCTTAGACCAGCTGAAAAAGGGAAAACAGTTATGGACATTATTTCAAGACAAGACCAA 9360
103344             GTCCTTAGACCAGCTGAAAAAGGGAAAACAGTTATGGACATTATTTCAAGACAAGACCAA 9360
BrasilZKV2015      GTCCTTAGACCAGCTGAAAAAGGGAAAACAGTTATGGACATTATTTCGAGACAAGACCAA 9360
GD01               GTCCTTAGACCAGCTGAAAAAGGGAAAACAGTTATGGACATTATTTCGAGACAAGACCAA 9360
```

```
Martinique          GTCCTTAGACCAGCTGAAAAAGGGAAAACAGTTATGGACATTATTTCGAGACAAGACCAA 9360
NatalRGN            GTCCTTAGACCAGCTGAAAAAGGGAAAACAGTTATGGACATTATTTCGAGACAAGACCAA 9360
HPF2013             GTCCTTAGACCAGCTGAAAAAGGGAAGACAGTTATGGACATTATTTCGAGACAAGACCAA 9360
SPH2015             GTCCTTAGACCAGCTGAAAAAGGGAAAACAGTTATGGACATTATTTCGAGACAAGACCAA 9360
Haiti2014           GTCCTTAGACCAGCTGAAAAAGGGAAGACAGTTATGGACATTATTTCGAGACAAGACCAA 9360
PRVABC59            GTCCTTAGACCAGCTGAAAAAGGGAAAACAGTTATGGACATTATTTCGAGACAAGACCAA 9360
BeH819015           GTCCTTAGACCAGCTGAAAAAGGGAAAACAGTTATGGACATTATTTCGAGACAAGACCAA 9360
Z1106033            GTCCTTAGACCAGCTGAAAAAGGGAAAACAGTTATGGACATTATTTCGAGACAAGACCAA 9360
BEH819966           GTCCTTAGACCAGCTGAAAAAGGGAAAACAGTTATGGACATTATTTCGAGACAAGACCAA 9360
SSABr               GTCCTTAGACCAGCTGAAAAAGGGAAAACAGTTATGGACATTATTTCGAGACAAGACCAA 9360
Beh815744           GTCCTTAGACCAGCTGAAAAAGGGAAAACAGTTATGGACATTATTTCGAGACAAGACCAA 9360
BEH818995           GTCCTTAGACCAGCTGAAAAAGGGAAAACAGTTATGGACATTATTTCGAGACAAGACCAA 9360
IbH30656            GTTCTCAGACCAGCTGAAGGAGGGAAAACAGTCATGGACATCATCTCAAGACAAGACCAG 9342
ArD128000           GTTCTCAGACCAGCTGAAGGAGGGAAAACAGTCATGGACATCATCTCAAGACAAGACCAG 9360
ArD7117             GTTCTCAGACCAGCTGAAGGAGGGAAAACAGTCATGGACATCATCTCAAGACAAGACCAG 9360
ARD_41519           GTTCTCAGACCAGCTGAAGGAGGGAAAACAGTCATGGACATCATCTCAAGACAAGACCAG 9360
ARD157995           GTCCTCAGACCAGCTGAAGGAGGAAAAACAGTTATGGACATCATTTCAAGACAAGACCAG 9360
MR_766              GTTCTCAGACCAGCTGAAGGAGGAAAAACAGTTATGGACATCATTTCAAGACAAGACCAG 9348
ARD158084           GTCCTCAGACCAGCTGAAGGAGGAAAAACAGTTATGGACATCATTTCAAGACAAGACCAG 9360
ArB1362             GTCCTTAGACCAGCTGAAGGAGGAAAAACAGTTATGGACATCATTTCAAGACAAGACCAG 9360
ARB13565            GTCCTCAGACCAGCTGAAGGAGGGAAAACAGTTATGGACATCATTTCAAGACAGGACCAG 9357
ARB7701             GTCCTCAGACCAGCTGAAGGAGGGAAAACAGTTATGGACATCATTTCAAGACAGGACCAG 9357
ARB15076            GTCCTCAGACCAGCTGAAGGAGGGAAAACAGTTATGGACATCATTTCAAGACAAGACCAG 9342
                    ** ** ************  *** ** ***** ******** ** ** ***** *****

P6-740              AGAGGGAGCGGACAAGTTGTTACTTACGCTCTTAATACATTCACCAACCTGGTGGTGCAG 9420
CPC0740             AGGGGGAGCGGACAAGTTGTCACTTACGCTCTTAATACATTCACCAACCTGGTGGTGCAG 9420
YAP2007             AGGGGGAGCGGACAAGTTGTCACTTACGCTCTTAATACATTCACCAACCTGGTGGTGCAG 9420
FSS13025            AGGGGGAGCGGACAAGTTGTCACTTACGCTCTTAATACATTTACCAACCTAGTGGTGCAG 9420
PLCal_ZV            AGGGGGAGCGGACAAGTTGTCACCTACGCTCTTAATACATTTACCAACCTAGTGGTGCAG 9309
SV0127/14           AGGGGGAGCGGACAAGTTGTCACTTACGCTCTTAACACATTTACCAACCTAGTGGTGCAA 9420
8375                AGGGGGAGCGGACAAGTTGTCACTTACGCTCTTAACACATTTACCAACCTAGTGGTGCAA 9420
103344              AGGGGGAGCGGACAAGTTGTCACTTACGCTCTTAACACATTTACCAACCTAGTGGTGCAA 9420
BrasilZKV2015       AGGGGGAGCGGACAAGTTGTCACTTACGCTCTTAACACATTTACCAACCTAGTGGTGCAA 9420
GD01                AGGGGGAGCGGACAAGTTGTCACTTACGCTCTTAACACATTTACCAACCTAGTGGTGCAA 9420
Martinique          AGGGGGAGCGGACAAGTTGTCACTTACGCTCTTAACACATTTACCAACCTAGTGGTGCAA 9420
NatalRGN            AGGGGGAGCGGACAAGTTGTCACTTACGCTCTTAACACATTTACCAACCTAGTGGTGCAA 9420
HPF2013             AGGGGGAGCGGACAAGTTGTCACTTACGCTCTTAACACATTTACCAACCTAGTGGTGCAA 9420
SPH2015             AGGGGGAGCGGACAAGTTGTCACTTACGCTCTTAACACATTTACCAACCTAGTGGTGCAA 9420
Haiti2014           AGGGGGAGCGGACAAGTTGTCACTTACGCTCTTAACACATTTACCAACCTAGTGGTGCAA 9420
PRVABC59            AGGGGGAGCGGACAAGTTGTCACTTACGCTCTTAACACATTTACCAACCTAGTGGTGCAA 9420
BeH819015           AGGGGGAGCGGACAAGTTGTCACTTACGCTCTTAACACATTTACCAACCTAGTGGTGCAA 9420
Z1106033            AGGGGGAGCGGACAAGTTGTCACTTACGCTCTTAACACATTTACCAACCTAGTGGTGCAA 9420
BEH819966           AGGGGGAGCGGACAAGTTGTCACTTACGCTCTTAACACATTTACCAACCTAGTGGTGCAA 9420
SSABr               AGGGGGAGCGGACAAGTTGTCACTTACGCTCTTAACACATTTACCAACCTAGTGGTGCAA 9420
Beh815744           AGGGGGAGCGGACAAGTTGTCACTTACGCTCTTAACACATTTACCAACCTAGTGGTGCAA 9420
BEH818995           AGGGGGAGCGGACAAGTTGTCACTTACGCTCTTAACACATTTACCAACCTAGTGGTGCAA 9420
IbH30656            AGAGGGAGCGGACAAGTTGTTACTTATGCTCTCAACACATTCACCAACCTGGTGGTGCAG 9402
ArD128000           AGAGGGAGTGGACAAGTTGTTACTTATGCCCTCAACACATTCACCAACCTGGTGGTGCAG 9420
ArD7117             AGAGGGAGCGGACAAGTTGTTACTTATGCTCTCAACACATTCACCAACCTGGTGGTGCAG 9420
ARD_41519           AGAGGGAGTGGACAAGTTGTTACTTATGCCCTCAACACATTCACCAACCTGGTGGTGCAG 9420
ARD157995           AGAGGGAGTGGACAAGTTGTCACTTATGCTCTCAACACATTCACCAACTTGGTGGTGCAG 9420
MR_766              AGAGGGAGTGGACAAGTTGTCACTTATGCTCTCAACACATTCACCAACTTGGTGGTGCAG 9408
ARD158084           AGAGGGAGTGGACAAGTTGTCACTTATGCTCTCAACACATTCACCAACTTGGTGGTGCAG 9420
ArB1362             AGAGGGAGTGGGCAAGTTGTCACTTATGCTCTCAACACGTTTACCAACTTAGTGGTGCAG 9420
ARB13565            AGAGGGAGTGGACAAGTTGTCACCTATGCTCTCAACACATTTACCAACTTAGTGGTGCAG 9417
ARB7701             AGAGGGAGTGGACAAGTTGTCACCTATGCTCTCAACACATTTACCAACTTAGTGGTGCAG 9417
ARB15076            AGAGGGAGTGGACAAGTTGTCACTTATGCTCTCAACACATTTACCAACTTGGTGGTGCAG 9402
                    ** ***** ** ******** ** ** ** ** ** ** ** ****** * ********

P6-740              CTCATTCGGAACATGGAGGCTGAGGAAGTTCTAGAGATGCAAGACTTGTGGCTGTTGAGG 9480
CPC0740             CTCATTCGGAATATGGAGGCTGAGGAAGTTCTAGAGATGCAAGACTTGTGGCTGCTGCGG 9480
YAP2007             CTCATTCGGAATATGGAGGCTGAGGAAGTTCTAGAGATGCAAGACTTGTGGCTGCTGCGG 9480
FSS13025            CTCATTCGGAATATGGAGGCTGAGGAAGTTCTAGAGATGCAAGACTTGTGGCTGCTGCGG 9480
PLCal_ZV            CTCATTCGGAATATGGAGGCTGAGGAAGTTCTAGAGATGCAAGACTTGTGGCTGCTGCGG 9369
SV0127/14           CTCATTCGGAATATGGAGGCTGAGGAAGTTCTAGAGATGCAAGACTTGTGGCTGCTGCGG 9480
8375                CTCATTCGGAATATGGAGGCTGAGGAAGTTCTAGAGATGCAAGACTTGTGGCTGCTGCGG 9480
103344              CTCATTCGGAATATGGAGGCTGAGGAAGTTCTAGAGATGCAAGACTTGTGGCTGCTGCGG 9480
```

```
BrasilZKV2015      CTCATTCGGAATATGGAGGCTGAGGAAGTTCTAGAGATGCAAGACTTGTGGCTGCTGCGG 9480
GD01               CTCATTCGGAATATGGAGGCTGAGGAAGTTCTAGAGATGCAAGACTTGTGGCTGCTGCGG 9480
Martinique         CTCATTCGGAATATGGAGGCTGAGGAAGTTCTAGAGATGCAAGACTTGTGGCTGCTGCGG 9480
NatalRGN           CTCATTCGGAATATGGAGGCTGAGGAAGTTCTAGAGATGCAAGACTTGTGGCTGCTGCGG 9480
HPF2013            CTCATTCGGAATATGGAGGCTGAGGAAGTTCTAGAGATGCAAGACTTGTGGCTGCTGCGG 9480
SPH2015            CTCATTCGGAATATGGAGGCTGAGGAAGTCCTAGAGATGCAAGACTTGTGGCTGCTGCGG 9480
Haiti2014          CTCATTCGGAATATGGAGGCTGAGGAAGTTCTAGAGATGCAAGACTTGTGGCTGCTGCGG 9480
PRVABC59           CTCATTCGGAATATGGAGGCTGAGGAAGTTCTAGAGATGCAAGACTTGTGGCTGCTGCGG 9480
BeH819015          CTCATTCGGAATATGGAGGCTGAGGAAGTTCTAGAGATGCAAGACTTGTGGCTGCTGCGG 9480
Z1106033           CTCATTCGGAATATGGAGGCTGAGGAAGTTCTAGAGATGCAAGACTTGTGGCTGCTGCGG 9480
BEH819966          CTCATTCGGAATATGGAGGCTGAGGAAGTTCTAGAGATGCAAGACTTGTGGCTGCTGCGG 9480
SSABr              CTCATTCGGAATATGGAGGCTGAGGAAGTTCTAGAGATGCAAGACTTGTGGCTGCTGCGG 9480
Beh815744          CTCATTCGGAATATGGAGGCTGAGGAAGTTCTAGAGATGCAAGACTTGTGGCTGCTGCGG 9480
BEH818995          CTCATTCGGAATATGGAGGCTGAGGAAGTTCTAGAGATGCAAGACTTGTGGCTGCTGCGG 9480
IbH30656           CTTATCCGGAACATGGAGGCTGAGGAGGTGCTAGAGATGCATGATCTATGGCTGTTGAGG 9462
ArD128000          CTTATCCGGAACATGGAGGCTGAGGAAGTGCTAGAGATGCATGATCTGTGGTTGTTGAGG 9480
ArD7117            CTTATCCGGAACATGGAGGCTGAGGAAGTGCTAGAGATGCATGATCTATGGTTGTTGAGG 9480
ARD_41519          CTTATCCGGAACATGGAGGCTGAGGAAGTGCTAGAGATGCATGATCTGTGGTTGTTGAGG 9480
ARD157995          CTTATCCGGAACATGGAAGCTGAGGAAGTGTTAGAGATGCAAGACTTATGGCTGTTGAGG 9480
MR_766             CTTATCCGGAACATGGAAGCTGAGGAAGTGTTAGAGATGCAAGACTTATGGTTGTTGAGG 9468
ARD158084          CTTATCCGGAACATGGAAGCTGAGGAAGTGTTAGAGATGCAAGACTTATGGCTGTTGAGG 9480
ArB1362            CTTATTCGGAACATGGAGGCTGAGGAAGTGTTAGAGATGCAAGACTTATGGCTGTTGAGG 9480
ARB13565           CTTATCCGGAATATGGAGGCTGAGGAAGTGTTAGAGATGCAAGACTTATGGCTGTTGAGG 9477
ARB7701            CTTATCCGGAATATGGAGGCTGAGGAAGTGTTAGAGATGCAAGACTTATGGCTGTTGAGG 9477
ARB15076           CTTATCCGGAACATGGAGGCTGAGGAAGTGCTAGAGATGCAAGACTTATGGCTGCTGAGG 9462
                   ** ** ***** ***** ******** **  ********** **  * *** ** ** **

P6-740             AGGCCAGAGAAGGTGACCAGCTGGTTGCAGAGCAACGGATGGGATAGGCTCAAACGAATG 9540
CPC0740            AGGCCAGAGAAAGTGACCAACTGGTTGCAAAGCAACGGATGGGATAGGCTCAAAAGAATG 9540
YAP2007            AGGTCAGAGAAAGTGACCAACTGGTTGCAGAGCAACGGATGGGATAGGCTCAAACGAATG 9540
FSS13025           AGGTCAGAGAAAGTGACCAACTGGTTGCAGAGCAATGGATGGGATAGGCTCAAACGAATG 9540
PLCal_ZV           AGGTCAGAGAAAGTGACCAACTGGTTGCAGAGCAACGGATGGGATAGGCTCAAACGAATG 9429
SV0127/14          AGGTCAGAGAAAGTGACCAACTGGTTGCAGAGCAACGGATGGGATAGGCTCAAACGAATG 9540
8375               AGGTCAGAGAAAGTGACCAACTGGTTGCAGAGCAACGGATGGGATAGGCTCAAACGAATG 9540
103344             AGGTCAGAGAAAGTGACCAACTGGTTGCAGAGCAACGGATGGGATAGGCTCAAACGAATG 9540
BrasilZKV2015      AGGTCAGAGAAAGTGACCAACTGGTTGCAGAGCAACGGATGGGATAGGCTCAAACGAATG 9540
GD01               AGGTCAGAGAAAGTGACCAACTGGTTGCAGAGCAACGGATGGGATAGGCTCAAACGAATG 9540
Martinique         AGGTCAGAGAAAGTGACCAACTGGTTGCAGAGCAACGGATGGGATAGGCTCAAACGAATG 9540
NatalRGN           AGGTCAGAGAAAGTGACCAACTGGTTGCAGAGCAACGGATGGGATAGGCTCAAACGAATG 9540
HPF2013            AGGTCAGAGAAAGTGACCAACTGGTTGCAGAGCAACGGATGGGATAGGCTCAAACGAATG 9540
SPH2015            AGGTCAGAGAAAGTGACCAACTGGTTGCAGAGCAACGGATGGGATAGGCTCAAACGAATG 9540
Haiti2014          AGGTCAGAGAAAGTGACCAACTGGTTGCAGAGCAACGGATGGGATAGGCTCAAACGAATG 9540
PRVABC59           AGGTCAGAGAAAGTGACCAACTGGTTGCAGAGCAACGGATGGGATAGGCTCAAACGAATG 9540
BeH819015          AGGTCAGAGAAAGTGACCAACTGGTTGCAGAGCAACGGATGGGATAGGCTCAAACGAATG 9540
Z1106033           AGGTCAGAGAAAGTGACTAACTGGTTGCAGAGCAACGGATGGGATAGGCTCAAACGAATG 9540
BEH819966          AGGTCAGAGAAAGTGACCAACTGGTTGCAGAGCAACGGATGGGATAGGCTCAAACGAATG 9540
SSABr              AGGTCAGAGAAAGTGACCAACTGGTTGCAGAGCAACGGATGGGATAGGCTCAAACGAATG 9540
Beh815744          AGGTCAGAGAAAGTGACCAACTGGTTGCAGAGCAACGGATGGGATAGGCTCAAACGAATG 9540
BEH818995          AGGTCAGAGAAAGTGACCAACTGGTTGCAGAGCAACGGATGGGATAGGCTCAAACGAATG 9540
IbH30656           AAGCCAGAGAAAGTGACCAGATGGTTGCAGAGCAATGGATGGGACAGACTCAAACGAATG 9522
ArD128000          AAACCAGAGAAAGTGACCAGATGGTTGCAGAGTAATGGATGGGACAGACTCAAACGGATG 9540
ArD7117            AAGCCAGAGAAAGTGACCAGATGGTTGCAGAGCAATGGATGGGACAGACTCAAACGGATG 9540
ARD_41519          AAACCAGAGAAAGTGACCAGATGGTTGCAGAGTAATGGATGGGACAGACTCAAACGGATG 9540
ARD157995          AAGCCAGAGAAAGTGACCAGATGGTTGCAGAGCAATGGATGGGATAGACTCAAACGAATG 9540
MR_766             AAGCCAGAGAAAGTGACCAGATGGTTGCAGAGCAATGGATGGGATAGACTCAAACGAATG 9528
ARD158084          AAGCCAGAGAAAGTGACCAGATGGTTGCAGAGCAATGGATGGGATGGACTCAAACGAATG 9540
ArB1362            AAGCCAGAGAAAGTGACTAGATGGTTGCAGAGTAATGGATGGGACAGACTCAAACGAATG 9540
ARB13565           AAGCCAGAGAAAGTGACCAGATGGTTGCAGTGCAATGGATGGGACAGACTCAAACGAATG 9537
ARB7701            AAGCCAGAGAAAGTGACCAGATGGTTGCAGTGCAATGGATGGGACAGACTCAAACGAATG 9537
ARB15076           AAGCCAGAGAAAGTGACCAGATGGTTGCAGTGCAATGGATGGGACAGACTCAAACGAATG 9522
                   *   ******* ***** *  ********  * ** ********  * ****** * ***

P6-740             GCAGTCAGTGGAGATGATTGTGTTGTGAAACCAATTGATGATAGGTTTGCACATGCCCTC 9600
CPC0740            GCAGTCAGTGGAGATGATTGCGTTGTGAAACCAATTGATGATAGGTTTGCACATGCCCTC 9600
YAP2007            GCAGTCAGTGGAGATGATTGCGTTGTGAAACCAATTGATGATAGGTTTGCACATGCCCTC 9600
FSS13025           GCAGTCAGTGGAGATGATTGCGTTGTGAAACCAATTGATGATAGGTTTGCACATGCTCTC 9600
PLCal_ZV           GCAGTCAGTGGAGATGATTGCGTTGTGAAGCCAATTGATGATAGGTTTGCACATGCCCTC 9489
SV0127/14          GCAGTCAGTGGAGATGATTGCGTTGTGAAGCCAATTGATGATAGGTTTGCACATGCCCTC 9600
```

```
8375            GCAGTCAGTGGAGATGATTGCGTTGTGAAGCCAATTGATGATAGGTTTGCACATGCCCTC 9600
103344          GCAGTCAGTGGAGATGATTGCGTTGTGAAGCCAATTGATGATAGGTTTGCACATGCCCTC 9600
BrasilZKV2015   GCAGTCAGTGGAGATGATTGCGTTGTGAAGCCAATTGATGATAGGTTTGCACATGCCCTC 9600
GD01            GCAGTCAGTGGAGATGATTGCGTTGTGAAGCCAATTGATGATAGGTTTGCACATGCCCTC 9600
Martinique      GCAGTCAGTGGAGATGATTGCGTTGTGAAGCCAATTGATGATAGGTTTGCACATGCCCTC 9600
NatalRGN        GCAGTCAGTGGAGATGATTGCGTTGTGAAGCCAATTGATGATAGGTTTGCACATGCCCTC 9600
HPF2013         GCAGTCAGTGGAGATGATTGCGTTGTGAAGCCAATTGATGATAGGTTTGCACATGCCCTC 9600
SPH2015         GCAGTCAGTGGAGATGATTGCGTTGTGAAGCCAATTGATGATAGGTTTGCACATGCCCTC 9600
Haiti2014       GCAGTCAGTGGAGATGATTGCGTTGTGAAGCCAATTGATGATAGGTTTGCACATGCCCTC 9600
PRVABC59        GCAGTCAGTGGAGATGATTGCGTTGTGAAGCCAATTGATGATAGGTTTGCACATGCCCTC 9600
BeH819015       GCAGTCAGTGGAGATGATTGCGTTGTGAAGCCAATTGATGATAGGTTTGCACATGCCCTC 9600
Z1106033        GCAGTCAGTGGAGATGATTGCGTTGTGAAGCCAATTGATGATAGGTTTGCACATGCCCTC 9600
BEH819966       GCAGTCAGTGGAGATGATTGCGTTGTGAAGCCAATTGATGATAGGTTTGCACATGCCCTC 9600
SSABr           GCAGTCAGTGGAGATGATTGCGTTGTGAAGCCAATTGATGATAGGTTTGCACATGCCCTC 9600
Beh815744       GCAGTCAGTGGAGATGATTGCGTTGTGAAGCCAATTGATGATAGGTTTGCACATGCCCTC 9600
BEH818995       GCAGTCAGTGGAGATGATTGCGTTGTGAAGCCAATTGATGATAGGTTTGCACATGCCCTC 9600
IbH30656        GCAGTCAGTGGAGATGACTGCGTTGTAAAGCCAATTGATGATAGGTTTGCACATGCCCTC 9582
ArD128000       GCAGTTAGTGGAGATGACTGCGTTGTAAAGCCAATTGATGATAGGTTTGCACATGCCCTC 9600
ArD7117         GCAGTTAGTGGAGATGACTGCGTTGTAAAGCCAATTGATGATAGGTTTGCACATGCCCTC 9600
ARD_41519       GCAGTTAGTGGAGATGACTGCGTTGTGAAGCCAATTGATGATAGGTTTGCACATGCCCTC 9600
ARD157995       GCGGTCAGTGGAGATGACTGCGTTGTGAAGCCAATCGATGATAGGTTTGCACATGCCCTC 9600
MR_766          GCGGTCAGTGGAGATGACTGCGTTGTGAAGCCAATCGATGATAGGTTTGCACATGCCCTC 9588
ARD158084       GCGGTCAGTGGAGATGACTGCGTTGTGAAGCCAATCGATGATAGGTTTGCACATGCCCTC 9600
ArB1362         GCAGTCAGTGGAGATGACTGTGTTGTGAAGCCAATTGATGATAGGTTTGCATGCTGCCTC 9600
ARB13565        GCAGTCAGTGGAGATGACTGCGTTGTGAAGCCAATTGATGATAGGTTTGCGCATGCCCTC 9597
ARB7701         GCAGTCAGTGGAGATGACTGCGTTGTGAAGCCAATTGATGATAGGTTTGCGCATGCCCTC 9597
ARB15076        GCAGTCAGTGGAGATGACTGCGTTGTGAAGCCAATTGATGATAGGTTTGCACATGCCCTC 9582
                ** ** *********** ** ***** ** ***** **************       ***

P6-740          AGGTTTTTGAATGACATGGGGAAAGTTAGGAAGGACACACAGGAGTGGAAACCCTCAACT 9660
CPC0740         AGGTTCTTGAATGATATGGGAAAAGTTAGGAAGGACACACAAGAGTGGAAACCCTCAACT 9660
YAP2007         AGGTTCTTGAATGATATGGGAAAAGTCAGGAAGGACACACAAGAGTGGAAACCCTCAACT 9660
FSS13025        AGGTTCTTGAATGATATGGGAAAAGTTAGGAAGGACACACAAGAGTGGAAGCCCTCAACT 9660
PLCal_ZV        AGGTTCTTGAATGATATGGGAAAAGTTAGGAAGGACACACAAGAGTGGAAACCCTCAACT 9549
SV0127/14       AGGTTCTTGAATGATATGGGAAAAGTTAGGAAGGACACACAAGAGTGGAAACCCTCAACT 9660
8375            AGGTTCTTGAATGATATGGGAAAAGTTAGGAAGGACACACAAGAGTGGAAACCCTCAACT 9660
103344          AGGTTCTTGAATGATATGGGAAAAGTTAGGAAGGACACACAAGAGTGGAAACCCTCAACT 9660
BrasilZKV2015   AGGTTCTTGAATGATATGGGAAAAGTTAGGAAGGACACACAAGAGTGGAAACCCTCAACT 9660
GD01            AGGTTCTTGAATGATATGGGAAAAGTTAGGAAGGACACACAAGAGTGGAAACCCTCAACT 9660
Martinique      AGGTTCTTGAATGATATGGGAAAAGTTAGGAAGGACACACAAGAGTGGAAACCCTCAACT 9660
NatalRGN        AGGTTCTTGAATGATATGGGAAAAGTTAGGAAGGACACACAAGAGTGGAAACCCTCAACT 9660
HPF2013         AGGTTCTTGAATGATATGGGAAAAGTTAGGAAGGACACACAAGAGTGGAAACCCTCAACT 9660
SPH2015         AGGTTCTTGAATGATATGGGAAAAGTTAGGAAGGACACACAAGAGTGGAAACCCTCAACT 9660
Haiti2014       AGGTTCTTGAATGATATGGGAAAAGTTAGGAAGGACACACAAGAGTGGAAACCCTCAACT 9660
PRVABC59        AGGTTCTTGAATGATATGGGAAAAGTTAGGAAGGACACACAAGAGTGGAAACCCTCAACT 9660
BeH819015       AGGTTCTTGAATGATATGGGAAAAGTTAGAAAGGACACACAAGAGTGGAAACCCTCAACT 9660
Z1106033        AGGTTCTTGAATGATATGGGAAAAGTTAGGAAGGACACACAAGAGTGGAAACCCTCAACT 9660
BEH819966       AGGTTCTTGAATGATATGGGAAAAGTTAGGAAGGACACACAAGAGTGGAAACCCTCAACT 9660
SSABr           AGGTTCTTGAATGATATGGGAAAAGTTAGGAAGGACACACAAGAGTGGAAACCCTCAACT 9660
Beh815744       AGGTTCTTGAATGATATGGGAAAAGTTAGGAAGGACACACAAGAGTGGAAACCCTCAACT 9660
BEH818995       AGGTTCTTGAATGATATGGGAAAAGTTAGGAAGGACACACAAGAGTGGAAACCCTCAACT 9660
IbH30656        AGGTTCTTGAATGACATGGGAAAAGTTAGGAAAGACACACAGGAATGGAAACCCTCGACT 9642
ArD128000       AGGTTCTTGAATGACATGGGAAAGGTTAGGAAAGACACACAGGAATGGAAGCCCTCGACT 9660
ArD7117         AGGTTCTTGAATGACATGGGAAAGGTTAGGAAAGACACACAGGAATGGAAACCCTCGACT 9660
ARD_41519       AGGTTCTTGAATGACATGGGAAAGGTTAGGAAAGACACACAGGAATGGAAACCCTCGACT 9660
ARD157995       AGGTTCTTGAATGACATGGGAAAAGTTAGGAAAGACACACAGGAGTGGAAACCCTCGACT 9660
MR_766          AGGTTCTTGAATGACATGGGAAAAGTTAGGAAAGACACACAGGAGTGGAAACCCTCGACT 9648
ARD158084       AGGTTCTTGAATGACATGGGAAAAGTTAGGAAAGACACACAGGAGTGGAAACCCTCGACT 9660
ArB1362         AGGTTCCTAAACGACATGGGGAAAGTTAGGAAAGACACACAGGAATGGAAACCCTCGACT 9660
ARB13565        AGGTTCTTAAATGACATGGGAAAAGTTAGGAAAGACACACAGGAATGGAAACCCTCGACT 9657
ARB7701         AGGTTCTTAAATGACATGGGAAAAGTTAGGAAAGACACACAGGAATGGAAACCCTCGACT 9657
ARB15076        AGGTTCTTGAATGACATGGGAAAAGTTAGGAAAGACACACAGGAATGGAAACCCTCGACT 9642
                *****  * ** ** ***** ** ** ** ** ******** ** ***** ***** ***

P6-740          GGATGGAGCAACTGGGAAGAAGTTCCGTTTTGCTCCCATCACTTCAACAAGCTTTACCTC 9720
CPC0740         GGATGGGACAACTGGGAAGAAGTTCCGTTTTGCTCCCACCACTTCAACAAACTCCATCTT 9720
YAP2007         GGATGGGACAATTGGGAAGAAGTTCCGTTTTGCTCCCACCACTTCAACAAGCTCCATCTC 9720
FSS13025        GGATGGGACAACTGGGAAGAAGTTCCGTTTTGCTCCCACCACTTCAACAAGCTCCATCTC 9720
```

```
PLCal_ZV           GGATGGGACAACTGGGAAGAAGTTCCGTTTTGCTCCCACCACTTCAACAAGCTTCATCTC 9609
SV0127/14          GGATGGGACAACTGGGAAGAAGTTCCGTTTTGTTCCCACCACTTCAACAAGCTCCATCTC 9720
8375               GGATGGGACAACTGGGAAGAAGTTCCGTTTTGCTCCCACCACTTCAACAAGCTCCATCTC 9720
103344             GGATGGGACAACTGGGAAGAAGTTCCGTTTTGCTCCCACCACTTCAACAAGCTCCATCTC 9720
BrasilZKV2015      GGATGGGACAACTGGGAAGAAGTTCCGTTTTGCTCCCACCACTTCAACAAGCTCCATCTC 9720
GD01               GGATGGGACAACTGGGAAGAAGTTCCGTTTTGCTCCCACCATTTCAACAAGCTCCATCTC 9720
Martinique         GGATGGGACAACTGGGAAGAAGTTCCGTTTTGCTCCCACCACTTCAACAAGCTCCATCTC 9720
NatalRGN           GGATGGGACAACTGGGAAGAAGTTCCGTTTTGCTCCCACCACTTCAACAAGCTCCATCTC 9720
HPF2013            GGATGGGACAACTGGGAAGAAGTTCCGTTTTGCTCCCACCACTTCAACAAGCTCCATCTC 9720
SPH2015            GGATGGGACAACTGGGAAGAAGTTCCGTTTTGCTCCCACCACTTCAACAAGCTCCATCTC 9720
Haiti2014          GGATGGGACAACTGGGAAGAAGTTCCGTTTTGCTCCCACCACTTCAACAAGCTCCATCTC 9720
PRVABC59           GGATGGGACAACTGGGAAGAAGTTCCGTTTTGCTCCCACCACTTCAACAAGCTCCATCTC 9720
BeH819015          GGATGGGACAACTGGGAAGAAGTTCCGTTTTGCTCCCACCACTTCAACAAGCTCCATCTC 9720
Z1106033           GGATGGGACAACTGGGAAGAAGTTCCGTTTTGCTCCCACCACTTCAACAAGCTCCATCTC 9720
BEH819966          GGATGGGACAACTGGGAAGAAGTTCCGTTTTGCTCCCACCACTTCAACAAGCTCCATCTC 9720
SSABr              GGATGGGACAACTGGGAAGAAGTTCCGTTTTGCTCCCACCACTTCAACAAGCTCCATCTC 9720
Beh815744          GGATGGGACAACTGGGAAGAAGTTCCGTTTTGCTCCCACCACTTCAACAAGCTCCATCTC 9720
BEH818995          GGATGGGACAACTGGGAAGAAGTTCCGTTTTGCTCCCACCACTTCAACAAGCTCCATCTC 9720
IbH30656           GGATGGAGCAATTGGGAAGAAGTCCCGTTCTGTTCCCACCACTTCAACAAGCTGCACCTC 9702
ArD128000          GGATGGAGCAATTGGGAGGAGGTCCCGTTCTGTTCTCACCACTTCAACAAGCTGCACCTC 9720
ArD7117            GGATGGAGCAATTGGGAAGAGGTCCCGTTCTGTTCTCACCACTTCAACAAGCTGCACCTC 9720
ARD_41519          GGATGGAGCAATTGGGAAGAGGTCCCGTTCTGTTCTCACCACTTTAACAAGCTGCACCTC 9720
ARD157995          GGATGGAGCAATTGGGAAGAAGTCCCGTTCTGCTCCCACCACTTCAACAAGCTGTACCTC 9720
MR_766             GGATGGAGCAATTGGGAAGAAGTCCCGTTCTGCTCCCACCACTTCAACAAGCTGTACCTC 9708
ARD158084          GGATGGAGCAATTGGGAAGAAGTCCCGTTCTGCTCCCACCACTTCAACAAGCTGTACCTC 9720
ArB1362            GGATGGAGCAATTGGGAAGAAGTCCCTTTCTGCTCCCACCACTTCAACAAGCTGCACCTC 9720
ARB13565           GGATGGAGCAATTGGGAAGAAGTCCCGTTCTGCTCCCACCATTTCAACAAGCTGCACCTC 9717
ARB7701            GGATGGAGCAATTGGGAAGAAGTCCCGTTCTGCTCCCACCATTTCAACAAGCTGCACCTC 9717
ARB15076           GGATGGAGCAATTGGGAAGAAGTCCCGTTCTGCTCCCACCATTTCAACAAGCTGCACCTC 9702
                   ******  *** ***** ** ** ** ** ** ** ** ** ** ***** **  * **

P6-740             AAGGACGGGAGGTCCATTGTGGTCCCCTGTCGCCACCAAGATGAACTGATTGGCCGAGCC 9780
CPC0740            AAGGACGGGAGGTCCATTGTGGTTCCCTGCCGCCACCAAGATGAACTGATTGGCCGAGCC 9780
YAP2007            AAGGACGGGAGGTCCATTGTGGTTCCCTGCCGCCACCAAGATGAACTGATTGGCCGAGCC 9780
FSS13025           AAGGACGGGAGGTCCATTGTGGTTCCCTGCCGCCACCAAGATGAACTGATTGGCCGAGCT 9780
PLCal_ZV           AAGGACGGGAGGTCCATTGTGGTTCCCTGCCGCCACCAAGATGAACTGATTGGCCGGGCC 9669
SV0127/14          AAGGACGGGAGGTCCATTGTGGTTCCCTGCCGCCACCAAGATGAACTGATTGGCCGGGCC 9780
8375               AAGGACGGGAGGTCCATTGTGGTTCCCTGCCGCCACCAAGATGAACTGATTGGCCGGGCC 9780
103344             AAGGACGGGAGGTCCATTGTGGTTCCCTGCCGCCACCAAGATGAACTGATTGGCCGGGCC 9780
BrasilZKV2015      AAGGACGGGAGGTCCATTGTGGTTCCCTGCCGCCACCAAGATGAACTGATTGGCCGGGCC 9780
GD01               AAGGACGGGAGGTCCATTGTGGTTCCCTGCCGCCACCAAGATGAACTGATTGGCCGGGCC 9780
Martinique         AAGGACGGGAGGTCCATTGTGGTTCCCTGCCGCCACCAAGATGAACTGATTGGCCGGGCC 9780
NatalRGN           AAGGACGGGAGGTCCATTGTGGTTCCCTGCCGCCACCAAGATGAACTGATTGGCCGGGCC 9780
HPF2013            AAGGACGGGAGGTCCATTGTGGTTCCCTGCCGCCACCAAGATGAACTGATTGGCCGGGCC 9780
SPH2015            AAGGACGGGAGGTCCATTGTGGTTCCCTGCCGCCACCAAGATGAACTGATTGGCCGGGCC 9780
Haiti2014          AAGGACGGGAGGTCCATTGTGGTTCCCTGCCGCCACCAAGATGAACTGATTGGCCGGGCC 9780
PRVABC59           AAGGACGGGAGGTCCATTGTGGTTCCCTGCCGCCACCAAGATGAACTGATTGGCCGGGCC 9780
BeH819015          AAGGACGGGAGGTCCATTGTGGTTCCCTGCCGCCACCAAGATGAACTGATTGGCCGGGCC 9780
Z1106033           AAGGACGGGAGGTCCATTGTGGTTCCCTGCCGCCACCAAGATGAACTGATTGGCCGGGCC 9780
BEH819966          AAGGACGGGAGGTCCATTGTGGTTCCCTGCCGCCACCAAGATGAACTGATTGGCCGGGCC 9780
SSABr              AAGGACGGGAGGTCCATTGTGGTTCCCTGCCGCCACCAAGATGAACTGATTGGCCGGGCC 9780
Beh815744          AAGGACGGGAGGTCCATTGTGGTTCCCTGCCGCCACCAAGATGAACTGATTGGCCGGGCC 9780
BEH818995          AAGGACGGGAGGTCCATTGTGGTTCCCTGCCGCCACCAAGATGAACTGATTGGCCGGGCC 9780
IbH30656           AAGGATGGGAGATCCATTGTGGTCCCCTGCCGCCACCAAGATGAACTGATTGGCCGAGCC 9762
ArD128000          AAGGACGGGAGATCCATTGTGGTCCCCTGCCGCCACCAAGATGAACTGATTGGCCGAGCC 9780
ArD7117            AAGGACGGGAGATCCATTGTGGTCCCCTGCCGCCACCAAGATGAACTGATTGGCCGAGCC 9780
ARD_41519          AAGGACGGGAGATCCATTGTGGTCCCCTGCCGCCACCAAGATGAACTGATTGGCCGAGCC 9780
ARD157995          AAGGATGGGAGATCCATTGTGGTCCCTTGCCGCCACCAAGATGAACTGATTGGCCGAGCT 9780
MR_766             AAGGATGGGAGATCCATTGTGGTCCCTTGCCGCCACCAAGATGAACTGATTGGCCGAGCT 9768
ARD158084          AAGGATGGGAGATCCATTGTGGTCCCTTGCCGCCACCAAGATGAACTGATTGGCCGAGCT 9780
ArB1362            AAGGATGGTAGATCCATTGTGGTCCCTTGCCGCCACCAAGATGAACTGATTGGCCGAGCC 9780
ARB13565           AAGGATGGTAGATCCATTGTGGTCCCTTGCCGCCACCAAGATGAATTGATTGGCCGAGCC 9777
ARB7701            AAGGATGGTAGATCCATTGTGGTCCCTTGCCGCCACCAAGATGAATTGATTGGCCGAGCC 9777
ARB15076           AAGGATGGTAGATCCATTGTGGTCCCTTGCCGCCACCAAGATGAATTGATTGGCCGAGCC 9762
                   ***** ** ** *********** ** ** *************** ********** **

P6-740             CGCGTCTCACCAGGGGCGGGATGGAGCATCCGGGAGACTGCTTGCCTAGCAAAATCATAT 9840
CPC0740            CGCGTATCACCAGGGGCGGGATGGAGCATCCGGGAGACTGCTTGCCTAGCAAAATCATAT 9840
```

```
YAP2007         CGCGTCTCACCAGGGGCGGGATGGAGCATCCGGGAGACTGCTTGCCTAGCAAAATCATAT 9840
FSS13025        CGCGTCTCACCGGGGGCGGGATGGAGCATCCGGGAGACTGCTTGCCTAGCAAAATCATAT 9840
PLCal_ZV        CGCGTCTCTCCAGGGGCGGGATGGAGCATCCGGGAGACTGCTTGCCTGGCAAAATCATAT 9729
SV0127/14       CGTGTCTCTCCAGGGGCGGGATGGAGCATCCGGGAGACTGCTTGCCTAGCAAAGTCATAT 9840
8375            CGCGTCTCTCCAGGGGCGGGATGGAGCATCCGGGAGACTGCTTGCCTAGCAAAATCATAT 9840
103344          CGCGTCTCTCCAGGGGCGGGATGGAGCATCCGGGAGACTGCTTGCCTAGCAAAATCATAT 9840
BrasilZKV2015   CGCGTCTCTCCAGGGGCGGGATGGAGCATCCGGGAGACTGCTTGCCTAGCAAAATCATAT 9840
GD01            CGCGTCTCTCCAGGGGCGGGATGGAGCATCCGGGAGACTGCTTGCCTAGCAAAATCATAT 9840
Martinique      CGCGTCTCTCCAGGGGCGGGATGGAGCATCCGGGAGACTGCTTGCCTAGCAAAATCATAT 9840
NatalRGN        CGCGTCTCTCCAGGGGCGGGATGGAGCATCCGGGAGACTGCTTGCCTAGCAAAATCATAT 9840
HPF2013         CGCGTCTCTCCAGGGGCGGGATGGAGCATCCGGGAGACTGCTTGCCTAGCAAAATCATAT 9840
SPH2015         CGCGTCTCTCCAGGGGCGGGATGGAGCATCCGGGAGACTGCTTGCCTAGCAAAATCATAT 9840
Haiti2014       CGCGTCTCTCCAGGGGCGGGATGGAGCATCCGGGAGACTGCTTGCCTAGCAAAATCATAT 9840
PRVABC59        CGCGTCTCTCCAGGGGCGGGATGGAGCATCCGGGAGACTGCTTGCCTAGCAAAATCATAT 9840
BeH819015       CGCGTCTCTCCAGGGGCGGGATGGAGCATCCGGGAGACTGCTTGCCTAGCAAAATCATAT 9840
Z1106033        CGCGTCTCTCCAGGGGCGGGATGGAGCATCCGGGAGACTGCTTGCCTAGCAAAATCATAT 9840
BEH819966       CGCGTCTCTCCAGGGGCGGGATGGAGCATCCGGGAGACTGCTTGCCTAGCAAAATCATAT 9840
SSABr           CGCGTCTCTCCAGGGGCGGGATGGAGCATCCGGGAGACTGCTTGCCTAGCAAAATCATAT 9840
Beh815744       CGCGTCTCTCCAGGGGCGGGATGGAGCATCCGGGAGACTGCTTGCCTAGCAAAATCATAT 9840
BEH818995       CGCGTCTCTCCAGGGGCGGGATGGAGCATCCGGGAGACTGCTTGCCTAGCAAAATCATAT 9840
IbH30656        CGTGTCTCACCAGGGGCAGGATGGAGCATCCGGGAGACTGCCTGTCTTGCAAAATCATAT 9822
ArD128000       CGTGTCTCACCAGGGGCAGGATGGAGCATCCGTGAGACTGCTTGCCTTGCAAAATCATAT 9840
ArD7117         CGTGTCTCACCAGGGGCAGGATGGAGCATCCGGGAGACTGCTTGTCTTGCAAAATCATAT 9840
ARD_41519       CGTGTCTCACCAGGGGCAGGATGGAGCATCCGTGAGACTGCTTGCCTTGCAAAATCATAT 9840
ARD157995       CGCGTTTCACCAGGGGCAGGATGGAGCATCCGGGAGACTGCCTGTCTTGCAAAATCATAT 9840
MR_766          CGCGTCTCACCAGGGGCAGGATGGAGCATCCGGGAGACTGCCTGTCTTGCAAAATCATAT 9828
ARD158084       CGCGTTTCACCAGGGGCAGGATGGAGCATCCGGGAGACTGCCTGTCTTGCAAAATCATAT 9840
ArB1362         CGTGTATCACCAGGGGCAGGATGGAGCATCCGGGAGACTGCCTGTCTTGCAAAATCATAT 9840
ARB13565        CGTGTATCACCAGGGGCAGGATGGAGCATTCGGGAGACTGCCTGTCTAGCAAAATCATAT 9837
ARB7701         CGTGTATCACCAGGGGCAGGATGGAGCATTCGGGAGACTGCCTGTCTAGCAAAATCATAT 9837
ARB15076        CGTGTATCACCAGGGGCAGGATGGAGCATCCGGGAGACTGCCTGTCTAGCAAAATCATAT 9822
                ** ** ** ** ***** *********** ** ******** ** ** ***** ******

P6-740          GCACAAATGTGGCAGCTTCTTTATTTCCACAGAAGGGACCTCCGACTGATGGCCAACGCC 9900
CPC0740         GCGCAAATGTGGCAGCTCCTTTATTTCCACAGAAGGGACCTCCGACTGATGGCCAATGCC 9900
YAP2007         GCGCAAATGTGGCAGCTCCTTTATTTCCACAGAAGGGACCTCCGACTGATGGCCAATGCC 9900
FSS13025        GCGCAAATGTGGCAGCTCCTTTATTTCCACAGAAGGGACCTCCGACTGATGGCCAATGCC 9900
PLCal_ZV        GCGCAAATGTGGCAGCTCCTTTATTTCCACAGAAGGGACCTCCGACTGATGGCCAATGCC 9789
SV0127/14       GCGCAAATGTGGCAGCTCCTTTATTTCCACAGAAGGGACCTCCGACTGATGGCCAATGCC 9900
8375            GCGCAAATGTGGCAGCTCCTTTATTTCCACAGAAGGGACCTCCGACTGATGGCCAATGCC 9900
103344          GCGCAAATGTGGCAGCTCCTTTATTTCCACAGAAGGGACCTCCGACTGATGGCCAATGCC 9900
BrasilZKV2015   GCGCAAATGTGGCAGCTCCTTTATTTCCACAGAAGGGACCTCCGACTGATGGCCAATGCC 9900
GD01            GCGCAAATGTGGCAGCTCCTTTATTTCCACAGAAGGGACCTCCGACTGATGGCCAATGCC 9900
Martinique      GCGCAAATGTGGCAGCTCCTTTATTTCCACAGAAGGGACCTCCGACTGATGGCCAATGCC 9900
NatalRGN        GCGCAAATGTGGCAGCTCCTTTATTTCCACAGAAGGGACCTCCGACTGATGGCCAATGCC 9900
HPF2013         GCGCAAATGTGGCAGCTCCTTTATTTCCACAGAAGGGACCTCCGACTGATGGCCAATGCC 9900
SPH2015         GCGCAAATGTGGCAGCTCCTTTATTTCCACAGAAGGGACCTCCGACTGATGGCCAATGCC 9900
Haiti2014       GCGCAAATGTGGCAGCTCCTTTATTTCCACAGAAGGGACCTCCGACTGATGGCCAATGCC 9900
PRVABC59        GCGCAAATGTGGCAGCTCCTTTATTTCCACAGAAGGGACCTCCGACTGATGGCCAATGCC 9900
BeH819015       GCGCAGATGTGGCAGCTCCTTTATTTCCACAGAAGGGACCTCCGACTGATGGCCAATGCC 9900
Z1106033        GCGCAAATGTGGCAGCTCCTTTATTTCCACAGAAGGGACCTCCGACTGATGGCCAATGCC 9900
BEH819966       GCGCAAATGTGGCAGCTCCTTTATTTCCACAGAAGGGACCTCCGACTGATGGCCAATGCC 9900
SSABr           GCGCAAATGTGGCAGCTCCTTTATTTCCACAGAAGGGACCTCCGACTGATGGCCAATGCC 9900
Beh815744       GCGCAAATGTGGCAGCTCCTTTATTTCCACAGAAGGGACCTCCGACTGATGGCCAATGCC 9900
BEH818995       GCGCAAATGTGGCAGCTCCTTTATTTCCACAGAAGGGACCTCCGACTGATGGCCAATGCC 9900
IbH30656        GCCCAGATGTGGCAGCTTCTTTATTTCCACAGAAGAGACCTCCGACTGATGGCCAATGCC 9882
ArD128000       GCACAGATGTGGCAGCTTCTTTATTTCCACAGAAGAGATCTCCGACTGATGGCCAATGCC 9900
ArD7117         GCACAGATGTGGCAGCTTCTTTATTTCCACAAAAGAGACCTCCGACTGATGGCCAATGCC 9900
ARD_41519       GCACAGATGTGGCAGCTTCTTTATTTCCACAGAAGAGATCTCCGACTGATGGCCAATGCC 9900
ARD157995       GCGCAGATGTGGCAGCTCCTTTATTTCCACAGAAGAGACCTTCGACTGATGGCTAATGCC 9900
MR_766          GCGCAGATGTGGCAGCTCCTTTATTTCCACAGAAGAGACCTTCGACTGATGGCTAATGCC 9888
ARD158084       GCGCAGATGTGGCAGCTCCTTTATTTCCACAGAAGAGACCTTCGACTGATGGCTAATGCC 9900
ArB1362         GCACAGATGTGGCAGCTTCTTTACTTCCACAGAAGAGACCTTCGACTGATGGCCAACGCC 9900
ARB13565        GCACAGATGTGGCAGCTTCTTTACTTCCACAGAAGAGACCTTCGACTGATGGCCAATGCT 9897
ARB7701         GCACAGATGTGGCAGCTTCTTTACTTCCACAGAAGAGACCTTCGACTGATGGCCAATGCT 9897
ARB15076        GCACAGATGTGGCAGCTTCTTTACTTCCACAGAAGAGACCTTCGACTGATGGCCAATGCT 9882
                ** ** *********** ***** ******* *** ** ** ********** ** **
```

```
P6-740          ATTTGTTCATCTGTGCCAGTTGACTGGGTTCCAACTGGGAGAACCACCTGGTCAATCCAT 9960
CPC0740         ATTTGTTCATCTGTGCCAGTTGATTGGGTTCCAACTGGGAGAACTACCTGGTCAATCCAT 9960
YAP2007         ATTTGTTCATCTGTGCCAGTTGACTGGGTTCCAACTGGGAGAACTACCTGGTCAATCCAT 9960
FSS13025        ATTTGTTCATCTGTGCCAGTTGACTGGGTTCCAACTGGGAGAACTACCTGGTCAATCCAT 9960
PLCal_ZV        ATTTGTTCATCTGTGCCAGTTGACTGGGTTCCAACTGGGAGAACTACCTGGTCAATCCAT 9849
SV0127/14       ATCTGTTCATCTGTGCCAGTTGACTGGGTTCCAACTGGGAGAACTACCTGGTCAATCCAT 9960
8375            ATTTGTTCATCTGTGCCAGTTGACTGGGTTCCAACTGGGAGAACTACCTGGTCAATCCAT 9960
103344          ATTTGTTCATCTGTGCCAGTTGACTGGGTTCCAACTGGGAGAACTACCTGGTCAATCCAT 9960
BrasilZKV2015   ATTTGTTCATCTGTGCCAGTTGACTGGGTTCCAACTGGGAGAACTACCTGGTCAATCCAT 9960
GD01            ATTTGTTCATCTGTGCCAGTTGACTGGGTTCCAACTGGGAGAACTACCTGGTCAATCCAT 9960
Martinique      ATTTGTTCATCTGTGCCAGTTGACTGGGTTCCAACTGGGAGAACTACCTGGTCAATCCAT 9960
NatalRGN        ATTTGTTCATCTGTGCCAGTTGACTGGGTTCCAACTGGGAGAACTACCTGGTCAATCCAT 9960
HPF2013         ATTTGTTCATCTGTGCCAGTTGACTGGGTTCCAACTGGGAGAACTACCTGGTCAATCCAT 9960
SPH2015         ATTTGTTCATCTGTGCCAGTTGACTGGGTTCCAACTGGGAGAACTACCTGGTCAATCCAT 9960
Haiti2014       ATTTGTTCATCTGTGCCAGTTGACTGGGTTCCAACTGGGAGAACTACCTGGTCAATCCAT 9960
PRVABC59        ATTTGTTCATCTGTGCCAGTTGACTGGGTTCCAACTGGGAGAACTACCTGGTCAATCCAT 9960
BeH819015       ATTTGTTCATCTGTGCCAGTTGACTGGGTTCCAACTGGGAGAACTACCTGGTCAATCCAT 9960
Z1106033        ATTTGTTCATCTGTGCCAGTTGACTGGGTTCCAACTGGGAGAACTACCTGGTCAATCCAT 9960
BEH819966       ATTTGTTCATCTGTGCCAGTTGACTGGGTTCCAACTGGGAGAACTACCTGGTCAATCCAT 9960
SSABr           ATTTGTTCATCTGTGCCAGTTGACTGGGTTCCAACTGGGAGAACTACCTGGTCAATCCAT 9960
Beh815744       ATTTGTTCATCTGTGCCAGTTGACTGGGTTCCAACTGGGAGAACTACCTGGTCAATCCAT 9960
BEH818995       ATTTGTTCATCTGTGCCAGTTGACTGGGTTCCAACTGGGAGAACTACCTGGTCAATCCAT 9960
IbH30656        ATCTGTTCGGCCGTGCCAGCCGACTGGGTCCCAACTGGGAGAACCACCTGGTCAATCCAT 9942
ArD128000       ATTTGTTCGGCTGTGCCAGTTGATTGGGTACCAACTGGGAGAACCACCTGGTCAATCCAT 9960
ArD7117         ATTTGTTCGGCTGTGCCAGTTGACTGGGTACCAACTGGGAGAACCACCTGGTCAATCCAT 9960
ARD_41519       ATTTGTTCGGCTGTGCCAGTTGATTGGGTACCAACTGGGAGAACCACCTGGTCAATCCAT 9960
ARD157995       ATTTGCTCGGCTGTACCAGTTGACTGGGTACCAACTGGGAGAACCACCTGGTCAATCCAT 9960
MR_766          ATTTGCTCGGCTGTGCCAGTTGACTGGGTACCAACTGGGAGAACCACCTGGTCAATCCAT 9948
ARD158084       ATTTGCTCGGCTGTACCAGTTGACTGGGTACCAACTGGGAGAACCACCTGGTCAATCCAT 9960
ArB1362         ATTTGTTCGGCTGTGCCAGTCGACTGGGTACCAACCGGGAGAACCACCTGGTCAATCCAC 9960
ARB13565        ATTTGTTCGGCTGTGCCAGTTGACTGGGTACCAACCGGGAGAACCACCTGGTCAATCCAC 9957
ARB7701         ATTTGTTCGGCTGTGCCAGTTGACTGGGTACCAACCGGGAGAACCACCTGGTCAATCCAC 9957
ARB15076        ATTTGTTCGGCTGTGCCAGTTGACTGGGTGCCAACCGGGAGAACCACCTGGTCAATCCAC 9942
                ** ** **  * ** ****  ** ***** ***** ******** **************

P6-740          GGAAAGGGAGAATGGATGACCACTGAGGACATGCTTGTGGTGTGGAACAGAGTGTGGATT 10020
CPC0740         GGAAAGGGAGAATGGATGACCACTGAAGACATGCTTGTGGTATGGAACAGAGTGTGGATT 10020
YAP2007         GGAAAGGGAGAATGGATGACCACTGAAGACATGCTTGTGGTGTGGAACAGAGTGTGGATT 10020
FSS13025        GGAAAGGGAGAATGGATGACCACTGAAGACATGCTTGTGGTGTGGAACAGAGTGTGGATT 10020
PLCal_ZV        GGAAAGGGAGAATGGATGACCACTGAAGACATGCTTGTGGTGTGGAACAGAGTGTGGATC 9909
SV0127/14       GGAAAGGGAGAATGGATGACCACTGAAGACATGCTTGTGGTGTGGAACAGAGTGTGGATT 10020
8375            GGAAAGGGAGAATGGATGACCACTGAAGACATGCTTGTGGTGTGGAACAGAGTGTGGATT 10020
103344          GGAAAGGGAGAATGGATGACCACTGAAGACATGCTTGTGGTGTGGAACAGAGTGTGGATT 10020
BrasilZKV2015   GGAAAGGGAGAATGGATGACCACTGAAGACATGCTTGTGGTGTGGAACAGAGTGTGGATT 10020
GD01            GGAAAGGGAGAATGGATGACCACTGAAGACATGCTTGTGGTGTGGAACAGAGTGTGGATT 10020
Martinique      GGAAAGGGAGAATGGATGACCACTGAAGACATGCTTGTGGTGTGGAACAGAGTGTGGATT 10020
NatalRGN        GGAAAGGGAGAATGGATGACCACTGAAGACATGCTTGTGGTGTGGAACAGAGTGTGGATT 10020
HPF2013         GGAAAGGGAGAATGGATGACCACTGAAGACATGCTTGTGGTGTGGAACAGAGTGTGGATT 10020
SPH2015         GGAAAGGGAGAATGGATGACCACTGAAGACATGCTTGTGGTGTGGAACAGAGTGTGGATT 10020
Haiti2014       GGAAAGGGAGAATGGATGACCACTGAAGACATGCTTGTGGTGTGGAACAGAGTGTGGATT 10020
PRVABC59        GGAAAGGGAGAATGGATGACCACTGAAGACATGCTTGTGGTGTGGAACAGAGTGTGGATT 10020
BeH819015       GGAAAGGGAGAATGGATGACCACTGAAGACATGCTTGTGGTGTGGAACAGAGTGTGGATT 10020
Z1106033        GGAAAGGGAGAATGGATGACCACTGAAGACATGCTTGTGGTGTGGAACAGAGTGTGGATT 10020
BEH819966       GGAAAGGGAGAATGGATGACCACTGAAGACATGCTTGTGGTGTGGAACAGAGTGTGGATT 10020
SSABr           GGAAAGGGAGAATGGATGACCACTGAAGACATGCTTGTGGTGTGGAACAGAGTGTGGATT 10020
Beh815744       GGAAAGGGAGAATGGATGACCACTGAAGACATGCTTGTGGTGTGGAACAGAGTGTGGATT 10020
BEH818995       GGAAAGGGAGAATGGATGACCACTGAAGACATGCTTGTGGTGTGGAACAGAGTGTGGATT 10020
IbH30656        GGAAAGGGAGAATGGATGACTAATGAGGACATGCTCATGGTGTGGAATAGAGTGTGGATT 10002
ArD128000       GGAAAGGGAGAATGGATGACTACTGAGGACATGCTCATGGTGTGGAATAGAGTGTGGATT 10020
ArD7117         GGAAAGGGAGAATGGATGACTACTGAGGACATGCTCATGGTGTGGAATAGAGTGTGGATT 10020
ARD_41519       GGAAAGGGAGAATGGATGACTACTGAGGACATGCTCATGGTGTGGAATAGAGTGTGGATT 10020
ARD157995       GGAAAGGGAGAATGGATGACCACTGAGGACATGCTCATGGTGTGGAATAGAGTGTGGATT 10020
MR_766          GGAAAGGGAGAATGGATGACCACTGAGGACATGCTCATGGTGTGGAATAGAGTGTGGATT 10008
ARD158084       GGAAAGGGAGAATGGATGACCACTGAGGACATGCTCATGGTGTGGAATAGAGTGTGGATT 10020
ArB1362         GGGAAGGGAGAATGGATGACCACTGAGGACATGCTCATGGTGTGGAATAGAGTGTGGATT 10020
ARB13565        GGAAAGGGAGAATGGATGACTACTGAGGACATGCTCATGGTGTGGAATAGAGTGTGGATT 10017
ARB7701         GGAAAGGGAGAATGGATGACTACTGAGGACATGCTCATGGTGTGGAATAGAGTGTGGATT 10017
ARB15076        GGAAAGGGAGAATGGATGACTACTGAGGACATGCTCATGGTGTGGAATAGAGTGTGGATT 10002
```

```
                   ** ***************** * *** ********  **** ***** ***********

P6-740             GAGGAGAACGACCACATGGAGGACAAGACCCCAGTCACGAAATGGACAGACATTCCCTAT 10080
CPC0740            GAGGAAAACGACCACATGGAAGACAAGACCCCAGTTACAAAATGGACAGACATTCCCTAT 10080
YAP2007            GAGGAGAACGACCACATGGAAGACAAGACCCCAGTTACGAAATGGACAGACATTCCCTAT 10080
FSS13025           GAGGAGAACGACCACATGGAAGACAAGACCCCAGTTACGAAATGGACAGACATTCCCTAT 10080
PLCal_ZV           GAGGAGAACGACCACATGGAAGACAAGACCCCAGTAACGAAATGGACAGACATTCCCTAT 9969
SV0127/14          GAGGAGAACGACCACATGGAAGACAAGACCCCAGTTACGAAATGGACAGACATTCCCTAT 10080
8375               GAGGAGAACGACCACATGGAAGACAAGACCCCAGTTACGAAATGGACAGACATTCCCTAT 10080
103344             GAGGAGAACGACCACATGGAAGACAAGACCCCAGTTACGAAATGGACAGACATTCCCTAT 10080
BrasilZKV2015      GAGGAGAACGACCACATGGAAGACAAGACCCCAGTTACGAAATGGACAGACATTCCCTAT 10080
GD01               GAGGAGAACGACCACATGGAAGACAAGACCCCAGTTACGAAATGGACAGACATTCCCTAT 10080
Martinique         GAGGAGAACGACCACATGGAAGACAAGACCCCAGTTGCGAAATGGACAGACATTCCCTAT 10080
NatalRGN           GAGGAGAACGACCACATGGAAGACAAGACCCCAGTTACGAAATGGACAGACATTCCCTAT 10080
HPF2013            GAGGAGAACGACCACATGGAAGACAAGACCCCAGTTACGAAATGGACAGACATTCCCTAT 10080
SPH2015            GAGGAGAACGACCACATGGAAGACAAGACCCCAGTTACGAAATGGACAGACATTCCCTAT 10080
Haiti2014          GAGGAGAACGACCACATGGAAGACAAGACCCCAGTTACGAAATGGACAGACATTCCCTAT 10080
PRVABC59           GAGGAGAACGACCACATGGAAGACAAGACCCCAGTTACGAAATGGACAGACATTCCCTAT 10080
BeH819015          GAGGAGAACGACCACATGGAAGACAAGACCCCAGTTACGAAATGGACAGACATTCCCTAT 10080
Z1106033           GAGGAGAACGACCACATGGAAGACAAGACCCCAGTTACGAAATGGACAGACATTCCCTAT 10080
BEH819966          GAGGAGAACGACCACATGGAAGACAAGACCCCAGTTACGAAATGGACAGACATCCCCTAT 10080
SSABr              GAGGAGAACGACCACATGGAAGACAAGACCCCAGTTACGAAATGGACAGACATCCCCTAT 10080
Beh815744          GAGGAGAACGACCACATGGAAGACAAGACCCCAGTTACGAAATGGACAGACATTCCCTAT 10080
BEH818995          GAGGAGAACGACCACATGGAAGACAAGACCCCAGTTACGAAATGGACAGACATCCCCTAT 10080
IbH30656           GAGGAGAACGACCACATGGGGGACAAGACCCCTGTAACAAAATGGACAGACATTCCCTAT 10062
ArD128000          GAGGAGAACGACCACATGGAGGACAAGACCCCTGTTCCAAAATGGACAGAAATTCCCTAC 10080
ArD7117            GAGGAGAACGACCACATGGAGGACAAGACCCCTGTAACAAAATGGACAGACATTCCCTAT 10080
ARD_41519          GAGGAGAACGACCACATGGAGGACAAGACCCCTGTAACAAAATGGACAGACATTCCCTAT 10080
ARD157995          GAGGAGAACGACCATATGGAGGACAAGACTCCTGTAACAAAATGGACAGACATTCCCTAT 10080
MR_766             GAGGAGAACGACCATATGGAGGACAAGACTCCTGTAACAAAATGGACAGACATTCCCTAT 10068
ARD158084          GAGGAGAACGACCATATGGAGGACAAGACTCCTGTAACAAAATGGACAGACATTCCCTAT 10080
ArB1362            GAGGAGAACGACCATATGGAGGACAAGACTCCTGTAACAAAGTGGACTGACATTCCCTAC 10080
ARB13565           GAGGAGAACGACCATATGGAGGACAAGACTCCTGTGACAAAGTGGACAGACATCCCCTAT 10077
ARB7701            GAGGAGAACGACCATATGGAGGACAAGACTCCTGTGACAAAGTGGACAGACATCCCCTAT 10077
ARB15076           GAGGAGAACGACCACATGGAGGACAAGACTCCTGTGACAAAGTGGACAGACATCCCCTAT 10062
                   ***** ******** ****  ******** ** **  * ** ***** ** ** *****

P6-740             TTGGGAAAAAGGGAAGACTTATGGTGTGGATCTCTTATAGGGCACAGACCACGCACTACT 10140
CPC0740            TTGGGAAAAAGAGAAGACTTGTGGTGTGGATCTCTCATAGGGCACAGACCGCGTACTACC 10140
YAP2007            TTGGGAAAAAGGGAAGACTTGTGGTGTGGATCTCTCATAGGGCACAGACCGCGCACTACC 10140
FSS13025           TTGGGAAAAAGGGAAGACTTGTGGTGTGGGTCTCTCATAGGGCACAGACCGCGCACCACC 10140
PLCal_ZV           TTGGGAAAAAGGGAAGACTTGTGGTGTGGATCTCTCATAGGGCACAGACCGCGCACCACC 10029
SV0127/14          CTGGGAAAAAGGGAAGACTTGTGGTGTGGATCTCTCATAGGGCACAGACCGCGCACCACC 10140
8375               TTGGGAAAAAGGGAAGACTTGTGGTGTGGATCTCTCATAGGGCACAGACCGCGCACCACC 10140
103344             TTGGGAAAAAGGGAAGACTTGTGGTGTGGATCTCTCATAGGGCACAGACCGCGCACCACC 10140
BrasilZKV2015      TTGGGAAAAAGGGAAGACTTGTGGTGTGGATCTCTCATAGGGCACAGACCGCGCACCACC 10140
GD01               TTGGGAAAAAGGGAAGACTTGTGGTGTGGATCTCTCATAGGGCACAGACCGCGCACCACC 10140
Martinique         TTGGGAAAAAGGGAAGACTTGTGGTGTGGATCTCTCATAGGGCACAGACCGCGCACCACC 10140
NatalRGN           TTGGGAAAAAGGGAAGACTTGTGGTGTGGATCTCTCATAGGGCACAGACCGCGCACCACC 10140
HPF2013            TTGGGAAAAAGGGAAGACTTGTGGTGTGGATCTCTCATAGGGCACAGACCGCGCACCACC 10140
SPH2015            TTGGGAAAAAGGGAAGACTTGTGGTGTGGATCTCTCATAGGGCACAGACCGCGCACCACC 10140
Haiti2014          TTGGGAAAAAGGGAAGACTTGTGGTGTGGATCTCTCATAGGGCACAGACCGCGCACCACC 10140
PRVABC59           TTGGGAAAAAGGGAAGACTTGTGGTGTGGATCTCTCATAGGGCACAGACCGCGCACCACC 10140
BeH819015          TTGGGAAAAAGGGAAGACTTGTGGTGTGGATCTCTCATAGGGCACAGACCGCGCACCACC 10140
Z1106033           TTGGGAAAAAGGGAAGACTTGTGGTGTGGATCTCTCATAGGGCACAGACCGCGCACCACC 10140
BEH819966          TTGGGAAAAAGGGAAGACTTGTGGTGTGGATCTCTCATAGGGCACAGACCGCGCACCACC 10140
SSABr              TTGGGAAAAAGGGAAGACTTGTGGTGTGGATCTCTCATAGGGCACAGACCGCGCACCACC 10140
Beh815744          TTGGGAAAAAGGGAAGACTTGTGGTGTGGATCTCTCATAGGGCACAGACCGCGCACCACC 10140
BEH818995          TTGGGAAAAAGGGAAGACTTGTGGTGTGGATCTCTCATAGGGCACAGACCGCGCACCACC 10140
IbH30656           TTGGGAAAAAGGGAGGACTTATGGTGTGGATCCCTTATAGGGCACAGACCTCGCACCACT 10122
ArD128000          TTGGGGAAAAGGGAGTCCCTATGGTGTGGATACATTATAGGGCACAGACCTCGCACCACT 10140
ArD7117            TTGGGGAAAAGGGAGGACTTATGGTGTGGATCCCTTATAGGGCACAGACCTCGCACCACT 10140
ARD_41519          TTGGGGAAAAGGGAGGACTTATGGTGTGGATCCCTTATAGGGCACAGACCTCGCACCACT 10140
ARD157995          CTAGGAAAAAGGGAGGACTTATGGTGTGGATCCCTTATAGGGCACAGACCCCGCACCACT 10140
MR_766             CTAGGAAAAAGGGAGGACTTATGGTGTGGATCCCTTATAGGGCACAGACCCCGCACCACT 10128
ARD158084          CTAGGAAAAAGGGAGGACTTATGGTGTGGATCCCTTATAGGGCACAGACCCCGCACCACT 10140
ArB1362            CTGGGAAAAAGGGAGGACTTATGGTGTGGATCCCTTATAGGGCACAGACCCCGCACCACT 10140
ARB13565           CTGGGAAAAAGGGAGGACTTATGGTGTGGATCCCTTATAGGGCACAGGCCCCGCACCACT 10137
```

```
ARB7701            CTGGGAAAAAGGGAGGACTTATGGTGTGGATCCCTTATAGGGCACAGGCCCCGCACCACT 10137
ARB15076           CTGGGAAAAAGGGAGGACTTATGGTGTGGATCCCTTATAGGGCACAGGCCCCGCACCACT 10122
                    * ** ***** **   * * ******** *   * *********** ** ** ** ** 

P6-740             TGGGCTGAGAACATTAAAGACACAGTCAACATGGTGCGCAGGATCATAGGTGATGAAGAA 10200
CPC0740            TGGGCTGAGAACATCAAAAATACAGTCAACATGATGCGCAGGATCATAGGTGATGAAGAA 10200
YAP2007            TGGGCTGAGAACATCAAAAACACAGTCAACATGATGCGCAGGATCATAGGTGATGAAGAA 10200
FSS13025           TGGGCTGAGAACATTAAAAACACAGTCAACATGATGCGTAGGATCATAGGTGATGAAGAA 10200
PLCal_ZV           TGGGCTGAGAACATCAAAAACACAGTCAACATGGTGCGCAGGATCATAGGTGATGAAGAA 10089
SV0127/14          TGGGCTGAGAACATTAAAAACACAGTCAACATGGTGCGCAGGATCATAGGTGATGAAGAA 10200
8375               TGGGCTGAGAACATTAAAAACACAGTCAACATGGTGCGCAGGATCATAGGTGATGAAGAA 10200
103344             TGGGCTGAGAACATTAAAAACACAGTCAACATGGTGCGCAGGATCATAGGTGATGAAGAA 10200
BrasilZKV2015      TGGGCTGAGAACATTAAAAATACAGTCAACATGGTGCGCAGGATCATAGGTGATGAAGAA 10200
GD01               TGGGCTGAGAACATTAAAAACACAGTCAACATGGTGCGCAGGATCATAGGTGATGAAGAA 10200
Martinique         TGGGCTGAGAACATTAAAAACACAGTCAACATGGTGCGCAGGATCATAGGTGATGAAGAA 10200
NatalRGN           TGGGCTGAGAACATTAAAAACACAGTCAACATGGTGCGCAGGATCATAGGTGATGAAGAA 10200
HPF2013            TGGGCTGAGAACATTAAAAACACAGTCAACATGGTGCGCAGGATCATAGGTGATGAAGAA 10200
SPH2015            TGGGCTGAGAACATTAAAAACACAGTCAACATGGTGCGCAGGATCATAGGTGATGAAGAA 10200
Haiti2014          TGGGCTGAGAACATTAAAAACACAGTCAACATGGTGCGCAGGATCATAGGTGATGAAGAA 10200
PRVABC59           TGGGCTGAGAACATTAAAAACACAGTCAACATGGTGCGCAGGATCATAGGTGATGAAGAA 10200
BeH819015          TGGGCTGAGAACATTAAAAACACAGTCAACATGGTGCGCAGGATCATAGGTGATGAAGAA 10200
Z1106033           TGGGCTGAGAACATTAAAAACACAGTCAACATGGTGCGCAGGATCATAGGTGATGAAGAA 10200
BEH819966          TGGGCTGAGAACATTAAAAACACAGTCAACATGGTGCGCAGGATCATAGGTGATGAAGAA 10200
SSABr              TGGGCTGAGAACATTAAAAACACAGTCAACATGGTGCGCAGGATCATAGGTGATGAAGAA 10200
Beh815744          TGGGCTGAGAACATTAAAAACACAGTCAACATGGTGCGCAGGATCATAGGTGATGAAGAA 10200
BEH818995          TGGGCTGAGAACATTAAAAACACAGTCAACATGGTGCGCAGGATCATAGGTGATGAAGAA 10200
IbH30656           TGGGCTGAGAACATCAAAGACACAGTCAACATGGTGCGTAGGATCATAGGTGATGAAGAA 10182
ArD128000          TGGGCTGAGAACATCAAAGACACAGTCAACATGGTGCTTAGGCTCATAGGTGATGAAGAA 10200
ArD7117            TGGGCTGAGAACATCAAAGACACAGTCAACATGGTGCGTAGGATCATAGGTGATGAAGAA 10200
ARD_41519          TGGGCTGAGAACATCAAAGACACAGTCAACATGGTGCGTAGGATCATAGGTGATGAAGAA 10200
ARD157995          TGGGCTGAAAACATCAAAGACACAGTCAACATGGTGCGCAGGATCATAGGTGATGAAGAA 10200
MR_766             TGGGCTGAAAACATCAAAGACACAGTCAACATGGTGCGCAGGATCATAGGTGATGAAGAA 10188
ARD158084          TGGGCTGAAAACATCAAAGACACAGTCAACATGGTGCGCAGGATCATAGGTGATGAAGAA 10200
ArB1362            TGGGCTGAGAACATCAAAGACACAGTCAACATGGTGCGCAGGATCATAGGTGATGAAGAA 10200
ARB13565           TGGGCTGAGAACATCAAAGACACAGTCAACATGGTGCGCAGGATCATAGGTGATGAAGAA 10197
ARB7701            TGGGCTGAGAACATCAAAGACACAGTCAACATGGTGCGCAGGATCATAGGTGATGAAGAA 10197
ARB15076           TGGGCTGAGAACATCAAAGACACAGTCAACATGGTGCGCAGGATCATAGGTGATGAAGAA 10182
                   ******** ***** *** * ************ ***  *** *****************

P6-740             AAGTACATGGACTACCTATCCACTCAAGTTCGCTACTTGGGTGAAGAAGGGTCCACACCT 10260
CPC0740            AAGTACATGGACTACCTATCCACCCAGGTTCGCTACTTGGGTGAAGAAGGGTCCACACCT 10260
YAP2007            AAGTACATGGACTACCTATCCACCCAAGTTCGCTACTTGGGTGAAGAAGGGTCCACACCC 10260
FSS13025           AAGTACGTGGACTACCTATCCACCCAAGTTCGCTACTTGGGCGAAGAAGGGTCCACACCT 10260
PLCal_ZV           AAGTACATGGACTACCTATCCACCCAAGTTCGCTACTTGGGCGAAGAAGGG--------- 10140
SV0127/14          AAGTACATGGACTACCTATCCACCCAAGTTCGCTACTTGGGTGAAGAAGGGTCTACACCT 10260
8375               AAGTACATGGACTACCTATCCACCCAAGTTCGCTACTTGGGTGAAGAAGGGTCTACACCT 10260
103344             AAGTACATGGACTACCTATCCACCCAAGTTCGCTACTTGGGTGAAGAAGGGTCTACACCT 10260
BrasilZKV2015      AAGTACATGGACTACCTATCCACCCAAGTTCGCTACTTGGGTGAAGAAGGGTCTACACCT 10260
GD01               AAGTACATGGACTACCTATCCACCCAAGTTCGCTACTTGGGTGAAGAAGGGTCTACACCT 10260
Martinique         AAGTACATGGACTACCTATCCACCCAAGTTCGCTACTTGGGTGAAGAAGGGTCTACACCT 10260
NatalRGN           AAGTACATGGACTACCTATCCACCCAAGTTCGCTACTTGGGTGAAGAAGGGTCTACACCT 10260
HPF2013            AAGTACATGGACTACCTATCCACCCAAGTTCGCTACTTGGGTGAAGAAGGGTCTACACCT 10260
SPH2015            AAGTACATGGACTACCTATCCACCCAAGTTCGCTACTTGGGTGAAGAAGGGTCTACACCT 10260
Haiti2014          AAGTACATGGACTACCTATCCACCCAAGTTCGCTACTTGGGTGAAGAAGGGTCTACACCT 10260
PRVABC59           AAGTACATGGACTACCTATCCACCCAAGTTCGCTACTTGGGTGAAGAAGGGTCTACACCT 10260
BeH819015          AAGTACATGGACTACCTATCCACCCAAGTTCGCTACTTGGGTGAAGAAGGGTCTACACCT 10260
Z1106033           AAGTACATGGACTACCTATCCACCCAAGTTCGCTACTTGGGTGAAGAAGGGTCTACACCT 10260
BEH819966          AAGTACATGGACTACCTATCCACCCAAGTTCGCTACTTGGGTGAAGAAGGGTCTACACCT 10260
SSABr              AAGTACATGGACTACCTATCCACCCAAGTTCGCTACTTGGGTGAAGAAGGGTCTACACCT 10260
Beh815744          AAGTACATGGACTACCTATCCACCCAAGTTCGCTACTTGGGTGAAGAAGGGTCTACACCT 10260
BEH818995          AAGTACATGGACTACCTATCCACCCAAGTTCGCTACTTGGGTGAAGAAGGGTCTACACCT 10260
IbH30656           AGGTACATGGACTACCTATCCACCCAGGTACGCTACTTGGGTGAGGAGGGGTCCACACCT 10242
ArD128000          AAGTACATGGACTACCTATCCACCCAAGTACGCTACTTGGGTGAGGAAGGGTCCACACCT 10260
ArD7117            AAGTACATGGACTACCTATCCACCCAAGTACGCTACTTGGGTGAGGAAGGGTCCACACCT 10260
ARD_41519          AAGTaCATGGACTACCTATCCACCCAAGTACGTTACTTGGGTGAGGAAGGGTCCACACCT 10260
ARD157995          AAGTACATGGACTATCTATCCACCCAAGTCCGCTACTTGGGTGAGGAAGGGTCCACACCC 10260
MR_766             AAGTACATGGACTATCTATCCACCCAAGTCCGCTACTTGGGTGAGGAAGGGTCCACACCC 10248
ARD158084          AAGTACATGGACTATCTATCCACCCAAGTCCGCTACTTGGGTGAGGAAGGGTCCACACCC 10260
```

```
ArB1362           AAATACATGGACTACCTATCCACCCAAGTCCGCTACTTGGGTGAGGAAGGGTCCACACCT 10260
ARB13565          AAATACATGGACTACTTATCCACCCAGGTCCGCTACTTGGGTGAGGAAGGGTCCACACCT 10257
ARB7701           AAATACATGGACTACTTATCCACCCAGGTCCGCTACTTGGGTGAGGAAGGGTCCACACCT 10257
ARB15076          AAATACATGGACTACTTATCCACCCAGGTCCGCTACTTGGGTGAGGAAGGGTCCACACCT 10242
                  *  *** *******  ******* ** ** ** ******** ** ** ***

P6-740            GGAGTGTTA    10269
CPC0740           GGAGTGCTG    10269
YAP2007           GGAGTGTTG    10269
FSS13025          GGAGTGCTA    10269
PLCal_ZV          ---------    10140
SV0127/14         GGAGTGCTA    10269
8375              GGAGTGCTG    10269
103344            GGAGTGCTG    10269
BrasilZKV2015     GGAGTGCTG    10269
GD01              GGAGTGCTG    10269
Martinique        GGAGTGCTG    10269
NatalRGN          GGAGTGCTG    10269
HPF2013           GGAGTGCTG    10269
SPH2015           GGAGTGCTG    10269
Haiti2014         GGAGTGCTG    10269
PRVABC59          GGAGTGCTG    10269
BeH819015         GGAGTGCTG    10269
Z1106033          GGAGTGCTG    10269
BEH819966         GGAGTGCTG    10269
SSABr             GGAGTGCTG    10269
Beh815744         GGAGTGCTG    10269
BEH818995         GGAGTGCTG    10269
IbH30656          GGAGTGCTG    10251
ArD128000         GGAGTGCTG    10269
ArD7117           GGAGTGCTG    10269
ARD_41519         GGAGTGCTG    10269
ARD157995         GGAGTGTTG    10269
MR_766            GGAGTGTTG    10257
ARD158084         GGAGTGTTG    10269
ArB1362           GGAGTGTTG    10269
ARB13565          GGAGTGTTG    10266
ARB7701           GGAGTGTTG    10266
ARB15076          GGAGTGTTG    10251
```

PLEASE NOTE: Showing colors on large alignments is slow.
