# Supplementary material for: A Comprehensive Systems Biology Approach to Studying Zika Virus
Source: PLoS One. 2016 Sep 1;11(9):e0161355. doi: 10.1371/journal.pone.0161355 (PMC5008700; doi:10.1371/journal.pone.0161355)
Supplement: S5 Data — (PDF) [file pone.0161355.s005.pdf]

Codon	Consensus AA	ω	[Confidence Interval](* if lower bound > 1)
1	M	0.018	[6.2e-10,0.26]
2	K	0.025	[6.2e-10,0.26]
3	N	0.024	[6.2e-10,0.26]
4	P	0.033	[6.2e-10,0.26]
5	K	0.024	[6.2e-10,0.26]
6	K	0.79	[0.26,1]
7	K	0.59	[0.26,1]
8	S	0.92	[0.26,1]
9	G	0.4	[0.054,1]
10	G	0.64	[0.26,1]
11	F	0.45	[0.054,1]
12	R	0.033	[6.2e-10,0.26]
13	I	0.028	[6.2e-10,0.26]
14	V	0.026	[6.2e-10,0.26]
15	N	0.027	[6.2e-10,0.26]
16	M	0.018	[6.2e-10,0.26]
17	L	0.037	[6.2e-10,0.26]
18	K	0.025	[6.2e-10,0.26]
19	R	0.033	[6.2e-10,0.26]
20	G	0.027	[6.2e-10,0.26]
21	V	0.026	[6.2e-10,0.26]
22	A	0.026	[6.2e-10,0.26]
23	R	0.037	[6.2e-10,0.26]
24	V	0.025	[6.2e-10,0.26]
25	S	0.21	[0.013,1]
26	P	0.033	[6.2e-10,0.26]
27	F	0.28	[0.013,1]
28	G	0.025	[6.2e-10,0.26]
29	G	0.028	[6.2e-10,0.26]
30	L	0.035	[6.2e-10,0.26]
31	K	0.023	[6.2e-10,0.26]
32	R	0.024	[6.2e-10,0.26]
33	L	0.035	[6.2e-10,0.26]
34	P	0.032	[6.2e-10,0.26]
35	A	0.026	[6.2e-10,0.26]
36	G	0.027	[6.2e-10,0.26]
37	L	0.035	[6.2e-10,0.26]
38	L	0.035	[6.2e-10,0.26]
39	L	0.035	[6.2e-10,0.26]
40	G	0.029	[6.2e-10,0.26]
41	H	0.033	[6.2e-10,0.26]
42	G	0.026	[6.2e-10,0.26]
43	P	0.033	[6.2e-10,0.26]
44	I	0.024	[6.2e-10,0.26]
45	R	0.025	[6.2e-10,0.26]
46	M	0.018	[6.2e-10,0.26]

47	V	0.028	[6.2e-10,0.26]
48	L	0.035	[6.2e-10,0.26]
49	A	0.024	[6.2e-10,0.26]
50	I	0.021	[6.2e-10,0.26]
51	L	0.037	[6.2e-10,0.26]
52	A	0.026	[6.2e-10,0.26]
53	F	0.032	[6.2e-10,0.26]
54	L	0.035	[6.2e-10,0.26]
55	R	0.026	[6.2e-10,0.26]
56	F	0.031	[6.2e-10,0.26]
57	T	0.025	[6.2e-10,0.26]
58	A	0.025	[6.2e-10,0.26]
59	I	0.024	[6.2e-10,0.26]
60	K	0.023	[6.2e-10,0.26]
61	P	0.032	[6.2e-10,0.26]
62	S	0.035	[6.2e-10,0.26]
63	L	0.035	[6.2e-10,0.26]
64	G	0.027	[6.2e-10,0.26]
65	L	0.032	[6.2e-10,0.26]
66	I	0.024	[6.2e-10,0.26]
67	N	0.026	[6.2e-10,0.26]
68	R	0.026	[6.2e-10,0.26]
69	W	0.035	[6.2e-10,0.26]
70	G	0.03	[6.2e-10,0.26]
71	S	0.033	[6.2e-10,0.26]
72	V	0.023	[6.2e-10,0.26]
73	G	0.024	[6.2e-10,0.26]
74	K	0.024	[6.2e-10,0.26]
75	K	0.024	[6.2e-10,0.26]
76	E	0.022	[6.2e-10,0.26]
77	A	0.029	[6.2e-10,0.26]
78	M	0.018	[6.2e-10,0.26]
79	E	0.024	[6.2e-10,0.26]
80	I	0.19	[0.013,0.26]
81	I	0.02	[6.2e-10,0.26]
82	K	0.023	[6.2e-10,0.26]
83	K	0.023	[6.2e-10,0.26]
84	F	0.03	[6.2e-10,0.26]
85	K	0.023	[6.2e-10,0.26]
86	K	0.025	[6.2e-10,0.26]
87	D	0.026	[6.2e-10,0.26]
88	L	0.035	[6.2e-10,0.26]
89	A	0.029	[6.2e-10,0.26]
90	A	0.026	[6.2e-10,0.26]
91	M	0.018	[6.2e-10,0.26]
92	L	0.035	[6.2e-10,0.26]
93	R	0.026	[6.2e-10,0.26]

94	I	0.02	[6.2e-10,0.26]
95	I	0.024	[6.2e-10,0.26]
96	N	0.027	[6.2e-10,0.26]
97	A	0.029	[6.2e-10,0.26]
98	R	0.023	[6.2e-10,0.26]
99	K	0.023	[6.2e-10,0.26]
100	E	0.022	[6.2e-10,0.26]
101	K	0.21	[0.013,1]
102	K	0.023	[6.2e-10,0.26]
103	R	0.026	[6.2e-10,0.26]
104	R	0.037	[6.2e-10,0.26]
105	G	0.027	[6.2e-10,0.26]
106	A	0.63	[0.26,1]
107	D	0.35	[0.054,1]
108	T	0.23	[0.013,1]
109	S	0.026	[6.2e-10,0.26]
110	V	0.21	[0.013,1]
111	G	0.027	[6.2e-10,0.26]
112	I	0.025	[6.2e-10,0.26]
113	V	0.47	[0.054,1]
114	G	0.23	[0.013,1]
115	L	0.032	[6.2e-10,0.26]
116	L	0.035	[6.2e-10,0.26]
117	L	0.035	[6.2e-10,0.26]
118	T	0.028	[6.2e-10,0.26]
119	T	0.026	[6.2e-10,0.26]
120	A	0.22	[0.013,1]
121	M	0.018	[6.2e-10,0.26]
122	A	0.025	[6.2e-10,0.26]
123	V	0.37	[0.054,1]
124	E	0.022	[6.2e-10,0.26]
125	V	0.22	[0.013,1]
126	T	0.029	[6.2e-10,0.26]
127	R	0.026	[6.2e-10,0.26]
128	R	0.036	[6.2e-10,0.26]
129	G	0.024	[6.2e-10,0.26]
130	S	0.23	[0.013,1]
131	A	0.025	[6.2e-10,0.26]
132	Y	0.032	[6.2e-10,0.26]
133	Y	0.034	[6.2e-10,0.26]
134	M	0.018	[6.2e-10,0.26]
135	Y	0.032	[6.2e-10,0.26]
136	L	0.035	[6.2e-10,0.26]
137	D	0.023	[6.2e-10,0.26]
138	R	0.024	[6.2e-10,0.26]
139	S	0.21	[0.013,1]
140	D	0.026	[6.2e-10,0.26]

141	A	0.029	[6.2e-10,0.26]
142	G	0.025	[6.2e-10,0.26]
143	E	0.2	[0.013,0.26]
144	A	0.026	[6.2e-10,0.26]
145	I	0.024	[6.2e-10,0.26]
146	S	0.035	[6.2e-10,0.26]
147	F	0.032	[6.2e-10,0.26]
148	P	0.47	[0.054,1]
149	T	0.026	[6.2e-10,0.26]
150	T	0.27	[0.013,1]
151	L	0.29	[0.013,1]
152	G	0.025	[6.2e-10,0.26]
153	V	0.2	[0.013,1]
154	N	0.025	[6.2e-10,0.26]
155	K	0.024	[6.2e-10,0.26]
156	C	0.033	[6.2e-10,0.26]
157	Y	0.27	[0.013,1]
158	I	0.21	[0.013,1]
159	Q	0.033	[6.2e-10,0.26]
160	I	0.024	[6.2e-10,0.26]
161	M	0.018	[6.2e-10,0.26]
162	D	0.025	[6.2e-10,0.26]
163	L	0.033	[6.2e-10,0.26]
164	G	0.025	[6.2e-10,0.26]
165	H	0.03	[6.2e-10,0.26]
166	M	0.17	[0.013,0.26]
167	C	0.035	[6.2e-10,0.26]
168	D	0.024	[6.2e-10,0.26]
169	A	0.026	[6.2e-10,0.26]
170	T	0.026	[6.2e-10,0.26]
171	M	0.018	[6.2e-10,0.26]
172	S	0.026	[6.2e-10,0.26]
173	Y	0.036	[6.2e-10,0.26]
174	E	0.023	[6.2e-10,0.26]
175	C	0.032	[6.2e-10,0.26]
176	P	0.035	[6.2e-10,0.26]
177	M	0.018	[6.2e-10,0.26]
178	L	0.035	[6.2e-10,0.26]
179	D	0.024	[6.2e-10,0.26]
180	E	0.022	[6.2e-10,0.26]
181	G	0.026	[6.2e-10,0.26]
182	V	0.024	[6.2e-10,0.26]
183	E	0.024	[6.2e-10,0.26]
184	P	0.032	[6.2e-10,0.26]
185	D	0.026	[6.2e-10,0.26]
186	D	0.024	[6.2e-10,0.26]
187	V	0.025	[6.2e-10,0.26]

188	D	0.026	[6.2e-10,0.26]
189	C	0.033	[6.2e-10,0.26]
190	W	0.035	[6.2e-10,0.26]
191	C	0.032	[6.2e-10,0.26]
192	N	0.024	[6.2e-10,0.26]
193	T	0.024	[6.2e-10,0.26]
194	T	0.026	[6.2e-10,0.26]
195	S	0.035	[6.2e-10,0.26]
196	T	0.03	[6.2e-10,0.26]
197	W	0.035	[6.2e-10,0.26]
198	V	0.029	[6.2e-10,0.26]
199	V	0.023	[6.2e-10,0.26]
200	Y	0.033	[6.2e-10,0.26]
201	G	0.027	[6.2e-10,0.26]
202	T	0.026	[6.2e-10,0.26]
203	C	0.034	[6.2e-10,0.26]
204	H	0.27	[0.013,1]
205	H	0.031	[6.2e-10,0.26]
206	K	0.025	[6.2e-10,0.26]
207	K	0.025	[6.2e-10,0.26]
208	G	0.03	[6.2e-10,0.26]
209	E	0.024	[6.2e-10,0.26]
210	A	0.22	[0.013,1]
211	R	0.034	[6.2e-10,0.26]
212	R	0.034	[6.2e-10,0.26]
213	S	0.035	[6.2e-10,0.26]
214	R	0.026	[6.2e-10,0.26]
215	R	0.026	[6.2e-10,0.26]
216	A	0.24	[0.013,1]
217	V	0.023	[6.2e-10,0.26]
218	T	0.21	[0.013,1]
219	L	0.032	[6.2e-10,0.26]
220	P	0.29	[0.013,1]
221	S	0.29	[0.013,1]
222	H	0.031	[6.2e-10,0.26]
223	S	0.28	[0.013,1]
224	T	0.027	[6.2e-10,0.26]
225	R	0.023	[6.2e-10,0.26]
226	K	0.023	[6.2e-10,0.26]
227	L	0.035	[6.2e-10,0.26]
228	Q	0.035	[6.2e-10,0.26]
229	T	0.024	[6.2e-10,0.26]
230	R	0.034	[6.2e-10,0.26]
231	S	0.032	[6.2e-10,0.26]
232	Q	0.033	[6.2e-10,0.26]
233	T	0.027	[6.2e-10,0.26]
234	W	0.035	[6.2e-10,0.26]

235	L	0.036	[6.2e-10,0.26]
236	E	0.024	[6.2e-10,0.26]
237	S	0.035	[6.2e-10,0.26]
238	R	0.026	[6.2e-10,0.26]
239	E	0.024	[6.2e-10,0.26]
240	Y	0.033	[6.2e-10,0.26]
241	T	0.22	[0.013,1]
242	K	0.023	[6.2e-10,0.26]
243	H	0.03	[6.2e-10,0.26]
244	L	0.035	[6.2e-10,0.26]
245	I	0.025	[6.2e-10,0.26]
246	R	0.35	[0.054,1]
247	V	0.028	[6.2e-10,0.26]
248	E	0.024	[6.2e-10,0.26]
249	N	0.025	[6.2e-10,0.26]
250	W	0.035	[6.2e-10,0.26]
251	I	0.02	[6.2e-10,0.26]
252	F	0.03	[6.2e-10,0.26]
253	R	0.023	[6.2e-10,0.26]
254	N	0.024	[6.2e-10,0.26]
255	P	0.034	[6.2e-10,0.26]
256	G	0.025	[6.2e-10,0.26]
257	F	0.032	[6.2e-10,0.26]
258	A	0.023	[6.2e-10,0.26]
259	L	0.3	[0.013,1]
260	A	0.58	[0.26,1]
261	A	0.23	[0.013,1]
262	A	0.24	[0.013,1]
263	A	0.22	[0.013,1]
264	I	0.027	[6.2e-10,0.26]
265	A	0.23	[0.013,1]
266	W	0.035	[6.2e-10,0.26]
267	L	0.035	[6.2e-10,0.26]
268	L	0.29	[0.013,1]
269	G	0.027	[6.2e-10,0.26]
270	S	0.025	[6.2e-10,0.26]
271	S	0.27	[0.013,1]
272	T	0.024	[6.2e-10,0.26]
273	S	0.024	[6.2e-10,0.26]
274	Q	0.035	[6.2e-10,0.26]
275	K	0.025	[6.2e-10,0.26]
276	V	0.026	[6.2e-10,0.26]
277	I	0.02	[6.2e-10,0.26]
278	Y	0.033	[6.2e-10,0.26]
279	L	0.035	[6.2e-10,0.26]
280	V	0.22	[0.013,1]
281	M	0.018	[6.2e-10,0.26]

282	I	0.02	[6.2e-10,0.26]
283	L	0.29	[0.013,1]
284	L	0.035	[6.2e-10,0.26]
285	I	0.027	[6.2e-10,0.26]
286	A	0.22	[0.013,1]
287	P	0.031	[6.2e-10,0.26]
288	A	0.025	[6.2e-10,0.26]
289	Y	0.033	[6.2e-10,0.26]
290	S	0.025	[6.2e-10,0.26]
291	I	0.024	[6.2e-10,0.26]
292	R	0.21	[0.013,1]
293	C	0.032	[6.2e-10,0.26]
294	I	0.022	[6.2e-10,0.26]
295	G	0.027	[6.2e-10,0.26]
296	V	0.026	[6.2e-10,0.26]
297	S	0.024	[6.2e-10,0.26]
298	N	0.026	[6.2e-10,0.26]
299	R	0.025	[6.2e-10,0.26]
300	D	0.023	[6.2e-10,0.26]
301	F	0.26	[0.013,1]
302	V	0.023	[6.2e-10,0.26]
303	E	0.023	[6.2e-10,0.26]
304	G	0.027	[6.2e-10,0.26]
305	M	0.018	[6.2e-10,0.26]
306	S	0.035	[6.2e-10,0.26]
307	G	0.03	[6.2e-10,0.26]
308	G	0.024	[6.2e-10,0.26]
309	T	0.027	[6.2e-10,0.26]
310	W	0.035	[6.2e-10,0.26]
311	V	0.029	[6.2e-10,0.26]
312	D	0.026	[6.2e-10,0.26]
313	V	0.24	[0.013,1]
314	V	0.025	[6.2e-10,0.26]
315	L	0.035	[6.2e-10,0.26]
316	E	0.024	[6.2e-10,0.26]
317	H	0.033	[6.2e-10,0.26]
318	G	0.026	[6.2e-10,0.26]
319	G	0.029	[6.2e-10,0.26]
320	C	0.033	[6.2e-10,0.26]
321	V	0.027	[6.2e-10,0.26]
322	T	0.026	[6.2e-10,0.26]
323	V	0.22	[0.013,1]
324	M	0.018	[6.2e-10,0.26]
325	A	0.025	[6.2e-10,0.26]
326	Q	0.033	[6.2e-10,0.26]
327	D	0.023	[6.2e-10,0.26]
328	K	0.023	[6.2e-10,0.26]

329	P	0.032	[6.2e-10,0.26]
330	T	0.23	[0.013,1]
331	V	0.028	[6.2e-10,0.26]
332	D	0.023	[6.2e-10,0.26]
333	I	0.021	[6.2e-10,0.26]
334	E	0.022	[6.2e-10,0.26]
335	L	0.035	[6.2e-10,0.26]
336	V	0.026	[6.2e-10,0.26]
337	T	0.025	[6.2e-10,0.26]
338	T	0.22	[0.013,1]
339	T	0.024	[6.2e-10,0.26]
340	V	0.028	[6.2e-10,0.26]
341	S	0.025	[6.2e-10,0.26]
342	N	0.024	[6.2e-10,0.26]
343	M	0.018	[6.2e-10,0.26]
344	A	0.025	[6.2e-10,0.26]
345	E	0.022	[6.2e-10,0.26]
346	V	0.22	[0.013,1]
347	R	0.026	[6.2e-10,0.26]
348	S	0.033	[6.2e-10,0.26]
349	Y	0.033	[6.2e-10,0.26]
350	C	0.032	[6.2e-10,0.26]
351	Y	0.034	[6.2e-10,0.26]
352	E	0.022	[6.2e-10,0.26]
353	A	0.025	[6.2e-10,0.26]
354	S	0.034	[6.2e-10,0.26]
355	I	0.19	[0.013,0.26]
356	S	0.032	[6.2e-10,0.26]
357	D	0.023	[6.2e-10,0.26]
358	M	0.018	[6.2e-10,0.26]
359	A	0.029	[6.2e-10,0.26]
360	S	0.032	[6.2e-10,0.26]
361	D	0.21	[0.013,1]
362	S	0.026	[6.2e-10,0.26]
363	R	0.033	[6.2e-10,0.26]
364	C	0.033	[6.2e-10,0.26]
365	P	0.032	[6.2e-10,0.26]
366	T	0.026	[6.2e-10,0.26]
367	Q	0.035	[6.2e-10,0.26]
368	G	0.029	[6.2e-10,0.26]
369	E	0.024	[6.2e-10,0.26]
370	A	0.22	[0.013,1]
371	Y	0.27	[0.013,1]
372	L	0.035	[6.2e-10,0.26]
373	D	0.023	[6.2e-10,0.26]
374	K	0.023	[6.2e-10,0.26]
375	Q	0.034	[6.2e-10,0.26]

376	S	0.035	[6.2e-10,0.26]
377	D	0.023	[6.2e-10,0.26]
378	T	0.03	[6.2e-10,0.26]
379	Q	0.035	[6.2e-10,0.26]
380	Y	0.3	[0.013,1]
381	V	0.026	[6.2e-10,0.26]
382	C	0.032	[6.2e-10,0.26]
383	K	0.024	[6.2e-10,0.26]
384	R	0.026	[6.2e-10,0.26]
385	T	0.025	[6.2e-10,0.26]
386	L	0.036	[6.2e-10,0.26]
387	V	0.21	[0.013,1]
388	D	0.023	[6.2e-10,0.26]
389	R	0.026	[6.2e-10,0.26]
390	G	0.029	[6.2e-10,0.26]
391	W	0.035	[6.2e-10,0.26]
392	G	0.027	[6.2e-10,0.26]
393	N	0.026	[6.2e-10,0.26]
394	G	0.025	[6.2e-10,0.26]
395	C	0.035	[6.2e-10,0.26]
396	G	0.026	[6.2e-10,0.26]
397	L	0.29	[0.013,1]
398	F	0.033	[6.2e-10,0.26]
399	G	0.026	[6.2e-10,0.26]
400	K	0.025	[6.2e-10,0.26]
401	G	0.024	[6.2e-10,0.26]
402	S	0.024	[6.2e-10,0.26]
403	L	0.035	[6.2e-10,0.26]
404	V	0.023	[6.2e-10,0.26]
405	T	0.026	[6.2e-10,0.26]
406	C	0.034	[6.2e-10,0.26]
407	A	0.23	[0.013,1]
408	K	0.023	[6.2e-10,0.26]
409	F	0.032	[6.2e-10,0.26]
410	A	0.21	[0.013,1]
411	C	0.033	[6.2e-10,0.26]
412	S	0.28	[0.013,1]
413	K	0.023	[6.2e-10,0.26]
414	K	0.024	[6.2e-10,0.26]
415	M	0.018	[6.2e-10,0.26]
416	T	0.23	[0.013,1]
417	G	0.024	[6.2e-10,0.26]
418	K	0.023	[6.2e-10,0.26]
419	S	0.024	[6.2e-10,0.26]
420	I	0.025	[6.2e-10,0.26]
421	Q	0.033	[6.2e-10,0.26]
422	P	0.031	[6.2e-10,0.26]

423	E	0.022	[6.2e-10,0.26]
424	N	0.025	[6.2e-10,0.26]
425	L	0.035	[6.2e-10,0.26]
426	E	0.022	[6.2e-10,0.26]
427	Y	0.033	[6.2e-10,0.26]
428	R	0.034	[6.2e-10,0.26]
429	I	0.02	[6.2e-10,0.26]
430	M	0.018	[6.2e-10,0.26]
431	L	0.036	[6.2e-10,0.26]
432	S	0.29	[0.013,1]
433	V	0.025	[6.2e-10,0.26]
434	H	0.033	[6.2e-10,0.26]
435	G	0.026	[6.2e-10,0.26]
436	S	0.033	[6.2e-10,0.26]
437	Q	0.033	[6.2e-10,0.26]
438	H	0.03	[6.2e-10,0.26]
439	S	0.026	[6.2e-10,0.26]
440	G	0.024	[6.2e-10,0.26]
441	M	0.018	[6.2e-10,0.26]
442	I	0.027	[6.2e-10,0.26]
443	V	0.028	[6.2e-10,0.26]
444	N	0.028	[6.2e-10,0.26]
445	D	0.21	[0.013,1]
446	X	0.4	[0.054,1]
447	G	0.028	[6.2e-10,0.26]
448	H	0.29	[0.013,1]
449	E	0.026	[6.2e-10,0.26]
450	T	0.031	[6.2e-10,0.26]
451	D	0.026	[6.2e-10,0.26]
452	E	0.024	[6.2e-10,0.26]
453	N	0.22	[0.013,1]
454	R	0.026	[6.2e-10,0.26]
455	A	0.024	[6.2e-10,0.26]
456	K	0.21	[0.013,1]
457	V	0.027	[6.2e-10,0.26]
458	E	0.022	[6.2e-10,0.26]
459	I	0.21	[0.013,1]
460	T	0.024	[6.2e-10,0.26]
461	P	0.033	[6.2e-10,0.26]
462	N	0.027	[6.2e-10,0.26]
463	S	0.035	[6.2e-10,0.26]
464	P	0.032	[6.2e-10,0.26]
465	R	0.026	[6.2e-10,0.26]
466	A	0.025	[6.2e-10,0.26]
467	E	0.024	[6.2e-10,0.26]
468	A	0.025	[6.2e-10,0.26]
469	T	0.026	[6.2e-10,0.26]

470	L	0.035	[6.2e-10,0.26]
471	G	0.026	[6.2e-10,0.26]
472	G	0.028	[6.2e-10,0.26]
473	F	0.033	[6.2e-10,0.26]
474	G	0.026	[6.2e-10,0.26]
475	S	0.024	[6.2e-10,0.26]
476	L	0.037	[6.2e-10,0.26]
477	G	0.027	[6.2e-10,0.26]
478	L	0.035	[6.2e-10,0.26]
479	D	0.025	[6.2e-10,0.26]
480	C	0.035	[6.2e-10,0.26]
481	E	0.024	[6.2e-10,0.26]
482	P	0.032	[6.2e-10,0.26]
483	R	0.023	[6.2e-10,0.26]
484	T	0.026	[6.2e-10,0.26]
485	G	0.026	[6.2e-10,0.26]
486	L	0.035	[6.2e-10,0.26]
487	D	0.023	[6.2e-10,0.26]
488	F	0.032	[6.2e-10,0.26]
489	S	0.035	[6.2e-10,0.26]
490	D	0.026	[6.2e-10,0.26]
491	L	0.035	[6.2e-10,0.26]
492	Y	0.036	[6.2e-10,0.26]
493	Y	0.033	[6.2e-10,0.26]
494	L	0.035	[6.2e-10,0.26]
495	T	0.027	[6.2e-10,0.26]
496	M	0.018	[6.2e-10,0.26]
497	N	0.025	[6.2e-10,0.26]
498	N	0.025	[6.2e-10,0.26]
499	K	0.023	[6.2e-10,0.26]
500	H	0.032	[6.2e-10,0.26]
501	W	0.29	[0.013,1]
502	L	0.035	[6.2e-10,0.26]
503	V	0.024	[6.2e-10,0.26]
504	H	0.25	[0.013,1]
505	K	0.024	[6.2e-10,0.26]
506	E	0.022	[6.2e-10,0.26]
507	W	0.035	[6.2e-10,0.26]
508	F	0.031	[6.2e-10,0.26]
509	H	0.032	[6.2e-10,0.26]
510	D	0.023	[6.2e-10,0.26]
511	I	0.025	[6.2e-10,0.26]
512	P	0.032	[6.2e-10,0.26]
513	L	0.037	[6.2e-10,0.26]
514	P	0.036	[6.2e-10,0.26]
515	W	0.035	[6.2e-10,0.26]
516	H	0.033	[6.2e-10,0.26]

517	A	0.44	[0.054,1]
518	G	0.025	[6.2e-10,0.26]
519	A	0.025	[6.2e-10,0.26]
520	D	0.023	[6.2e-10,0.26]
521	T	0.027	[6.2e-10,0.26]
522	G	0.23	[0.013,1]
523	T	0.03	[6.2e-10,0.26]
524	P	0.032	[6.2e-10,0.26]
525	H	0.031	[6.2e-10,0.26]
526	W	0.035	[6.2e-10,0.26]
527	N	0.024	[6.2e-10,0.26]
528	N	0.024	[6.2e-10,0.26]
529	K	0.024	[6.2e-10,0.26]
530	E	0.023	[6.2e-10,0.26]
531	A	0.025	[6.2e-10,0.26]
532	L	0.035	[6.2e-10,0.26]
533	V	0.025	[6.2e-10,0.26]
534	E	0.022	[6.2e-10,0.26]
535	F	0.03	[6.2e-10,0.26]
536	K	0.023	[6.2e-10,0.26]
537	D	0.024	[6.2e-10,0.26]
538	A	0.026	[6.2e-10,0.26]
539	H	0.031	[6.2e-10,0.26]
540	A	0.026	[6.2e-10,0.26]
541	K	0.023	[6.2e-10,0.26]
542	R	0.023	[6.2e-10,0.26]
543	Q	0.035	[6.2e-10,0.26]
544	T	0.029	[6.2e-10,0.26]
545	V	0.026	[6.2e-10,0.26]
546	V	0.024	[6.2e-10,0.26]
547	V	0.029	[6.2e-10,0.26]
548	L	0.036	[6.2e-10,0.26]
549	G	0.024	[6.2e-10,0.26]
550	S	0.22	[0.013,1]
551	Q	0.034	[6.2e-10,0.26]
552	E	0.024	[6.2e-10,0.26]
553	G	0.027	[6.2e-10,0.26]
554	A	0.026	[6.2e-10,0.26]
555	V	0.029	[6.2e-10,0.26]
556	H	0.03	[6.2e-10,0.26]
557	T	0.024	[6.2e-10,0.26]
558	A	0.028	[6.2e-10,0.26]
559	L	0.034	[6.2e-10,0.26]
560	A	0.029	[6.2e-10,0.26]
561	G	0.027	[6.2e-10,0.26]
562	A	0.029	[6.2e-10,0.26]
563	L	0.035	[6.2e-10,0.26]

564	E	0.022	[6.2e-10,0.26]
565	A	0.029	[6.2e-10,0.26]
566	E	0.022	[6.2e-10,0.26]
567	M	0.018	[6.2e-10,0.26]
568	D	0.026	[6.2e-10,0.26]
569	G	0.029	[6.2e-10,0.26]
570	A	0.025	[6.2e-10,0.26]
571	K	0.023	[6.2e-10,0.26]
572	G	0.027	[6.2e-10,0.26]
573	R	0.023	[6.2e-10,0.26]
574	L	0.036	[6.2e-10,0.26]
575	S	0.47	[0.054,1]
576	S	0.036	[6.2e-10,0.26]
577	G	0.026	[6.2e-10,0.26]
578	H	0.032	[6.2e-10,0.26]
579	L	0.035	[6.2e-10,0.26]
580	K	0.025	[6.2e-10,0.26]
581	C	0.033	[6.2e-10,0.26]
582	R	0.033	[6.2e-10,0.26]
583	L	0.036	[6.2e-10,0.26]
584	K	0.025	[6.2e-10,0.26]
585	M	0.018	[6.2e-10,0.26]
586	D	0.024	[6.2e-10,0.26]
587	K	0.023	[6.2e-10,0.26]
588	L	0.035	[6.2e-10,0.26]
589	R	0.025	[6.2e-10,0.26]
590	L	0.035	[6.2e-10,0.26]
591	K	0.023	[6.2e-10,0.26]
592	G	0.027	[6.2e-10,0.26]
593	V	0.023	[6.2e-10,0.26]
594	S	0.035	[6.2e-10,0.26]
595	Y	0.035	[6.2e-10,0.26]
596	S	0.033	[6.2e-10,0.26]
597	L	0.035	[6.2e-10,0.26]
598	C	0.034	[6.2e-10,0.26]
599	T	0.027	[6.2e-10,0.26]
600	A	0.025	[6.2e-10,0.26]
601	A	0.21	[0.013,1]
602	F	0.25	[0.013,1]
603	T	0.026	[6.2e-10,0.26]
604	F	0.49	[0.054,1]
605	T	0.23	[0.013,1]
606	K	0.023	[6.2e-10,0.26]
607	I	0.22	[0.013,1]
608	P	0.032	[6.2e-10,0.26]
609	A	0.029	[6.2e-10,0.26]
610	E	0.024	[6.2e-10,0.26]

611	T	0.026	[6.2e-10,0.26]
612	L	0.035	[6.2e-10,0.26]
613	H	0.032	[6.2e-10,0.26]
614	G	0.026	[6.2e-10,0.26]
615	T	0.026	[6.2e-10,0.26]
616	V	0.026	[6.2e-10,0.26]
617	T	0.026	[6.2e-10,0.26]
618	V	0.023	[6.2e-10,0.26]
619	E	0.022	[6.2e-10,0.26]
620	V	0.024	[6.2e-10,0.26]
621	Q	0.033	[6.2e-10,0.26]
622	Y	0.29	[0.013,1]
623	A	0.025	[6.2e-10,0.26]
624	G	0.024	[6.2e-10,0.26]
625	T	0.23	[0.013,1]
626	D	0.026	[6.2e-10,0.26]
627	G	0.027	[6.2e-10,0.26]
628	P	0.033	[6.2e-10,0.26]
629	C	0.032	[6.2e-10,0.26]
630	K	0.023	[6.2e-10,0.26]
631	V	0.23	[0.013,1]
632	P	0.032	[6.2e-10,0.26]
633	A	0.23	[0.013,1]
634	Q	0.033	[6.2e-10,0.26]
635	M	0.018	[6.2e-10,0.26]
636	A	0.023	[6.2e-10,0.26]
637	V	0.023	[6.2e-10,0.26]
638	D	0.023	[6.2e-10,0.26]
639	M	0.018	[6.2e-10,0.26]
640	Q	0.034	[6.2e-10,0.26]
641	T	0.027	[6.2e-10,0.26]
642	L	0.035	[6.2e-10,0.26]
643	T	0.026	[6.2e-10,0.26]
644	P	0.032	[6.2e-10,0.26]
645	V	0.028	[6.2e-10,0.26]
646	G	0.025	[6.2e-10,0.26]
647	R	0.023	[6.2e-10,0.26]
648	L	0.035	[6.2e-10,0.26]
649	I	0.02	[6.2e-10,0.26]
650	T	0.026	[6.2e-10,0.26]
651	A	0.027	[6.2e-10,0.26]
652	N	0.024	[6.2e-10,0.26]
653	P	0.035	[6.2e-10,0.26]
654	V	0.024	[6.2e-10,0.26]
655	I	0.025	[6.2e-10,0.26]
656	T	0.03	[6.2e-10,0.26]
657	E	0.024	[6.2e-10,0.26]

658	S	0.21	[0.013,1]
659	T	0.03	[6.2e-10,0.26]
660	E	0.022	[6.2e-10,0.26]
661	N	0.026	[6.2e-10,0.26]
662	S	0.035	[6.2e-10,0.26]
663	K	0.023	[6.2e-10,0.26]
664	M	0.018	[6.2e-10,0.26]
665	M	0.018	[6.2e-10,0.26]
666	L	0.035	[6.2e-10,0.26]
667	E	0.023	[6.2e-10,0.26]
668	L	0.033	[6.2e-10,0.26]
669	D	0.024	[6.2e-10,0.26]
670	P	0.032	[6.2e-10,0.26]
671	P	0.032	[6.2e-10,0.26]
672	F	0.033	[6.2e-10,0.26]
673	G	0.024	[6.2e-10,0.26]
674	D	0.025	[6.2e-10,0.26]
675	S	0.036	[6.2e-10,0.26]
676	Y	0.032	[6.2e-10,0.26]
677	I	0.027	[6.2e-10,0.26]
678	V	0.025	[6.2e-10,0.26]
679	I	0.02	[6.2e-10,0.26]
680	G	0.027	[6.2e-10,0.26]
681	V	0.027	[6.2e-10,0.26]
682	G	0.024	[6.2e-10,0.26]
683	D	0.21	[0.013,1]
684	K	0.023	[6.2e-10,0.26]
685	K	0.024	[6.2e-10,0.26]
686	I	0.024	[6.2e-10,0.26]
687	T	0.026	[6.2e-10,0.26]
688	H	0.031	[6.2e-10,0.26]
689	H	0.03	[6.2e-10,0.26]
690	W	0.035	[6.2e-10,0.26]
691	X	0.27	[0.013,1]
692	R	0.24	[0.013,1]
693	S	0.4	[0.054,1]
694	G	0.028	[6.2e-10,0.26]
695	S	0.024	[6.2e-10,0.26]
696	T	0.4	[0.054,1]
697	I	0.025	[6.2e-10,0.26]
698	G	0.027	[6.2e-10,0.26]
699	K	0.024	[6.2e-10,0.26]
700	A	0.025	[6.2e-10,0.26]
701	F	0.033	[6.2e-10,0.26]
702	E	0.024	[6.2e-10,0.26]
703	A	0.026	[6.2e-10,0.26]
704	T	0.03	[6.2e-10,0.26]

705	V	0.023	[6.2e-10,0.26]
706	R	0.026	[6.2e-10,0.26]
707	G	0.029	[6.2e-10,0.26]
708	A	0.026	[6.2e-10,0.26]
709	K	0.023	[6.2e-10,0.26]
710	R	0.026	[6.2e-10,0.26]
711	M	0.018	[6.2e-10,0.26]
712	A	0.025	[6.2e-10,0.26]
713	V	0.026	[6.2e-10,0.26]
714	L	0.035	[6.2e-10,0.26]
715	G	0.025	[6.2e-10,0.26]
716	D	0.023	[6.2e-10,0.26]
717	T	0.026	[6.2e-10,0.26]
718	A	0.026	[6.2e-10,0.26]
719	W	0.035	[6.2e-10,0.26]
720	D	0.023	[6.2e-10,0.26]
721	F	0.032	[6.2e-10,0.26]
722	G	0.027	[6.2e-10,0.26]
723	S	0.035	[6.2e-10,0.26]
724	V	0.027	[6.2e-10,0.26]
725	G	0.025	[6.2e-10,0.26]
726	G	0.029	[6.2e-10,0.26]
727	A	0.38	[0.054,1]
728	L	0.26	[0.013,1]
729	N	0.024	[6.2e-10,0.26]
730	S	0.035	[6.2e-10,0.26]
731	L	0.035	[6.2e-10,0.26]
732	G	0.027	[6.2e-10,0.26]
733	K	0.023	[6.2e-10,0.26]
734	G	0.027	[6.2e-10,0.26]
735	I	0.39	[0.054,1]
736	H	0.031	[6.2e-10,0.26]
737	Q	0.034	[6.2e-10,0.26]
738	I	0.026	[6.2e-10,0.26]
739	F	0.033	[6.2e-10,0.26]
740	G	0.027	[6.2e-10,0.26]
741	A	0.025	[6.2e-10,0.26]
742	A	0.028	[6.2e-10,0.26]
743	F	0.03	[6.2e-10,0.26]
744	K	0.025	[6.2e-10,0.26]
745	S	0.035	[6.2e-10,0.26]
746	L	0.035	[6.2e-10,0.26]
747	F	0.032	[6.2e-10,0.26]
748	G	0.027	[6.2e-10,0.26]
749	G	0.027	[6.2e-10,0.26]
750	M	0.018	[6.2e-10,0.26]
751	S	0.033	[6.2e-10,0.26]

752	W	0.035	[6.2e-10,0.26]
753	F	0.03	[6.2e-10,0.26]
754	S	0.035	[6.2e-10,0.26]
755	Q	0.034	[6.2e-10,0.26]
756	I	0.025	[6.2e-10,0.26]
757	L	0.032	[6.2e-10,0.26]
758	I	0.021	[6.2e-10,0.26]
759	G	0.026	[6.2e-10,0.26]
760	T	0.024	[6.2e-10,0.26]
761	L	0.035	[6.2e-10,0.26]
762	L	0.035	[6.2e-10,0.26]
763	V	0.2	[0.013,1]
764	W	0.035	[6.2e-10,0.26]
765	L	0.035	[6.2e-10,0.26]
766	G	0.029	[6.2e-10,0.26]
767	L	0.035	[6.2e-10,0.26]
768	N	0.024	[6.2e-10,0.26]
769	T	0.22	[0.013,1]
770	K	0.023	[6.2e-10,0.26]
771	N	0.027	[6.2e-10,0.26]
772	G	0.027	[6.2e-10,0.26]
773	S	0.036	[6.2e-10,0.26]
774	I	0.025	[6.2e-10,0.26]
775	S	0.033	[6.2e-10,0.26]
776	L	0.033	[6.2e-10,0.26]
777	T	0.21	[0.013,1]
778	C	0.032	[6.2e-10,0.26]
779	L	0.035	[6.2e-10,0.26]
780	A	0.026	[6.2e-10,0.26]
781	L	0.036	[6.2e-10,0.26]
782	G	0.024	[6.2e-10,0.26]
783	G	0.027	[6.2e-10,0.26]
784	V	0.023	[6.2e-10,0.26]
785	L	0.2	[0.013,0.26]
786	I	0.024	[6.2e-10,0.26]
787	F	0.03	[6.2e-10,0.26]
788	L	0.035	[6.2e-10,0.26]
789	S	0.033	[6.2e-10,0.26]
790	T	0.025	[6.2e-10,0.26]
791	A	0.028	[6.2e-10,0.26]
792	V	0.027	[6.2e-10,0.26]
793	S	0.036	[6.2e-10,0.26]
794	A	0.029	[6.2e-10,0.26]
795	D	0.025	[6.2e-10,0.26]
796	V	0.024	[6.2e-10,0.26]
797	G	0.024	[6.2e-10,0.26]
798	C	0.032	[6.2e-10,0.26]

799	S	0.032	[6.2e-10,0.26]
800	V	0.023	[6.2e-10,0.26]
801	D	0.023	[6.2e-10,0.26]
802	F	0.03	[6.2e-10,0.26]
803	S	0.035	[6.2e-10,0.26]
804	K	0.024	[6.2e-10,0.26]
805	K	0.37	[0.054,1]
806	E	0.024	[6.2e-10,0.26]
807	T	0.024	[6.2e-10,0.26]
808	R	0.026	[6.2e-10,0.26]
809	C	0.034	[6.2e-10,0.26]
810	G	0.027	[6.2e-10,0.26]
811	T	0.024	[6.2e-10,0.26]
812	G	0.024	[6.2e-10,0.26]
813	V	0.024	[6.2e-10,0.26]
814	F	0.03	[6.2e-10,0.26]
815	V	0.39	[0.054,1]
816	Y	0.034	[6.2e-10,0.26]
817	N	0.025	[6.2e-10,0.26]
818	D	0.024	[6.2e-10,0.26]
819	V	0.029	[6.2e-10,0.26]
820	E	0.21	[0.013,1]
821	A	0.026	[6.2e-10,0.26]
822	W	0.035	[6.2e-10,0.26]
823	R	0.023	[6.2e-10,0.26]
824	D	0.023	[6.2e-10,0.26]
825	R	0.028	[6.2e-10,0.26]
826	Y	0.032	[6.2e-10,0.26]
827	K	0.2	[0.013,1]
828	Y	0.033	[6.2e-10,0.26]
829	H	0.033	[6.2e-10,0.26]
830	P	0.036	[6.2e-10,0.26]
831	D	0.023	[6.2e-10,0.26]
832	S	0.033	[6.2e-10,0.26]
833	P	0.034	[6.2e-10,0.26]
834	R	0.034	[6.2e-10,0.26]
835	R	0.026	[6.2e-10,0.26]
836	L	0.035	[6.2e-10,0.26]
837	A	0.025	[6.2e-10,0.26]
838	A	0.025	[6.2e-10,0.26]
839	A	0.22	[0.013,1]
840	V	0.026	[6.2e-10,0.26]
841	K	0.023	[6.2e-10,0.26]
842	Q	0.034	[6.2e-10,0.26]
843	A	0.027	[6.2e-10,0.26]
844	W	0.035	[6.2e-10,0.26]
845	E	0.024	[6.2e-10,0.26]

846	D	0.21	[0.013,1]
847	G	0.024	[6.2e-10,0.26]
848	I	0.026	[6.2e-10,0.26]
849	C	0.035	[6.2e-10,0.26]
850	G	0.024	[6.2e-10,0.26]
851	I	0.024	[6.2e-10,0.26]
852	S	0.033	[6.2e-10,0.26]
853	S	0.034	[6.2e-10,0.26]
854	V	0.029	[6.2e-10,0.26]
855	S	0.034	[6.2e-10,0.26]
856	R	0.026	[6.2e-10,0.26]
857	M	0.018	[6.2e-10,0.26]
858	E	0.024	[6.2e-10,0.26]
859	N	0.024	[6.2e-10,0.26]
860	I	0.024	[6.2e-10,0.26]
861	M	0.018	[6.2e-10,0.26]
862	W	0.035	[6.2e-10,0.26]
863	R	0.22	[0.013,1]
864	S	0.035	[6.2e-10,0.26]
865	V	0.024	[6.2e-10,0.26]
866	E	0.024	[6.2e-10,0.26]
867	G	0.024	[6.2e-10,0.26]
868	E	0.022	[6.2e-10,0.26]
869	L	0.032	[6.2e-10,0.26]
870	N	0.025	[6.2e-10,0.26]
871	A	0.026	[6.2e-10,0.26]
872	I	0.024	[6.2e-10,0.26]
873	L	0.036	[6.2e-10,0.26]
874	E	0.023	[6.2e-10,0.26]
875	E	0.022	[6.2e-10,0.26]
876	N	0.027	[6.2e-10,0.26]
877	G	0.027	[6.2e-10,0.26]
878	V	0.028	[6.2e-10,0.26]
879	Q	0.035	[6.2e-10,0.26]
880	L	0.035	[6.2e-10,0.26]
881	T	0.025	[6.2e-10,0.26]
882	V	0.028	[6.2e-10,0.26]
883	V	0.028	[6.2e-10,0.26]
884	V	0.024	[6.2e-10,0.26]
885	G	0.026	[6.2e-10,0.26]
886	S	0.036	[6.2e-10,0.26]
887	V	0.025	[6.2e-10,0.26]
888	K	0.025	[6.2e-10,0.26]
889	N	0.024	[6.2e-10,0.26]
890	P	0.033	[6.2e-10,0.26]
891	M	0.018	[6.2e-10,0.26]
892	W	0.035	[6.2e-10,0.26]

893	R	0.026	[6.2e-10,0.26]
894	G	0.25	[0.013,1]
895	P	0.032	[6.2e-10,0.26]
896	Q	0.29	[0.013,1]
897	R	0.026	[6.2e-10,0.26]
898	L	0.035	[6.2e-10,0.26]
899	P	0.033	[6.2e-10,0.26]
900	V	0.023	[6.2e-10,0.26]
901	P	0.035	[6.2e-10,0.26]
902	V	0.024	[6.2e-10,0.26]
903	N	0.026	[6.2e-10,0.26]
904	E	0.21	[0.013,1]
905	L	0.035	[6.2e-10,0.26]
906	P	0.033	[6.2e-10,0.26]
907	H	0.032	[6.2e-10,0.26]
908	G	0.026	[6.2e-10,0.26]
909	W	0.035	[6.2e-10,0.26]
910	K	0.024	[6.2e-10,0.26]
911	A	0.026	[6.2e-10,0.26]
912	W	0.035	[6.2e-10,0.26]
913	G	0.024	[6.2e-10,0.26]
914	K	0.025	[6.2e-10,0.26]
915	S	0.032	[6.2e-10,0.26]
916	Y	0.28	[0.013,1]
917	F	0.032	[6.2e-10,0.26]
918	V	0.026	[6.2e-10,0.26]
919	R	0.025	[6.2e-10,0.26]
920	A	0.024	[6.2e-10,0.26]
921	A	0.025	[6.2e-10,0.26]
922	K	0.023	[6.2e-10,0.26]
923	T	0.026	[6.2e-10,0.26]
924	N	0.024	[6.2e-10,0.26]
925	N	0.024	[6.2e-10,0.26]
926	S	0.22	[0.013,1]
927	F	0.033	[6.2e-10,0.26]
928	V	0.028	[6.2e-10,0.26]
929	V	0.025	[6.2e-10,0.26]
930	D	0.024	[6.2e-10,0.26]
931	G	0.029	[6.2e-10,0.26]
932	D	0.023	[6.2e-10,0.26]
933	T	0.026	[6.2e-10,0.26]
934	L	0.035	[6.2e-10,0.26]
935	K	0.023	[6.2e-10,0.26]
936	E	0.024	[6.2e-10,0.26]
937	C	0.034	[6.2e-10,0.26]
938	P	0.031	[6.2e-10,0.26]
939	L	0.032	[6.2e-10,0.26]

940	K	0.36	[0.054,1]
941	H	0.032	[6.2e-10,0.26]
942	R	0.026	[6.2e-10,0.26]
943	A	0.025	[6.2e-10,0.26]
944	W	0.035	[6.2e-10,0.26]
945	N	0.025	[6.2e-10,0.26]
946	S	0.025	[6.2e-10,0.26]
947	F	0.031	[6.2e-10,0.26]
948	L	0.035	[6.2e-10,0.26]
949	V	0.023	[6.2e-10,0.26]
950	E	0.022	[6.2e-10,0.26]
951	D	0.026	[6.2e-10,0.26]
952	H	0.031	[6.2e-10,0.26]
953	G	0.024	[6.2e-10,0.26]
954	F	0.031	[6.2e-10,0.26]
955	G	0.024	[6.2e-10,0.26]
956	V	0.23	[0.013,1]
957	F	0.031	[6.2e-10,0.26]
958	H	0.03	[6.2e-10,0.26]
959	T	0.027	[6.2e-10,0.26]
960	S	0.027	[6.2e-10,0.26]
961	V	0.028	[6.2e-10,0.26]
962	W	0.035	[6.2e-10,0.26]
963	L	0.034	[6.2e-10,0.26]
964	K	0.023	[6.2e-10,0.26]
965	V	0.027	[6.2e-10,0.26]
966	R	0.026	[6.2e-10,0.26]
967	E	0.023	[6.2e-10,0.26]
968	D	0.025	[6.2e-10,0.26]
969	Y	0.033	[6.2e-10,0.26]
970	S	0.035	[6.2e-10,0.26]
971	L	0.037	[6.2e-10,0.26]
972	E	0.023	[6.2e-10,0.26]
973	C	0.035	[6.2e-10,0.26]
974	D	0.024	[6.2e-10,0.26]
975	P	0.032	[6.2e-10,0.26]
976	A	0.026	[6.2e-10,0.26]
977	V	0.026	[6.2e-10,0.26]
978	I	0.021	[6.2e-10,0.26]
979	G	0.027	[6.2e-10,0.26]
980	T	0.026	[6.2e-10,0.26]
981	A	0.029	[6.2e-10,0.26]
982	A	0.43	[0.054,1]
983	K	0.023	[6.2e-10,0.26]
984	G	0.027	[6.2e-10,0.26]
985	K	0.2	[0.013,1]
986	E	0.023	[6.2e-10,0.26]

987	A	0.027	[6.2e-10,0.26]
988	V	0.21	[0.013,1]
989	H	0.03	[6.2e-10,0.26]
990	S	0.027	[6.2e-10,0.26]
991	D	0.026	[6.2e-10,0.26]
992	L	0.037	[6.2e-10,0.26]
993	G	0.23	[0.013,1]
994	Y	0.034	[6.2e-10,0.26]
995	W	0.035	[6.2e-10,0.26]
996	I	0.027	[6.2e-10,0.26]
997	E	0.023	[6.2e-10,0.26]
998	S	0.027	[6.2e-10,0.26]
999	E	0.024	[6.2e-10,0.26]
1000	K	0.023	[6.2e-10,0.26]
1001	N	0.026	[6.2e-10,0.26]
1002	D	0.023	[6.2e-10,0.26]
1003	T	0.026	[6.2e-10,0.26]
1004	W	0.035	[6.2e-10,0.26]
1005	R	0.023	[6.2e-10,0.26]
1006	L	0.035	[6.2e-10,0.26]
1007	K	0.36	[0.054,1]
1008	R	0.023	[6.2e-10,0.26]
1009	A	0.027	[6.2e-10,0.26]
1010	H	0.031	[6.2e-10,0.26]
1011	L	0.035	[6.2e-10,0.26]
1012	I	0.026	[6.2e-10,0.26]
1013	E	0.022	[6.2e-10,0.26]
1014	M	0.018	[6.2e-10,0.26]
1015	K	0.025	[6.2e-10,0.26]
1016	T	0.026	[6.2e-10,0.26]
1017	C	0.035	[6.2e-10,0.26]
1018	E	0.023	[6.2e-10,0.26]
1019	W	0.035	[6.2e-10,0.26]
1020	P	0.032	[6.2e-10,0.26]
1021	K	0.023	[6.2e-10,0.26]
1022	S	0.035	[6.2e-10,0.26]
1023	H	0.03	[6.2e-10,0.26]
1024	T	0.026	[6.2e-10,0.26]
1025	L	0.035	[6.2e-10,0.26]
1026	W	0.035	[6.2e-10,0.26]
1027	T	0.22	[0.013,1]
1028	D	0.026	[6.2e-10,0.26]
1029	G	0.027	[6.2e-10,0.26]
1030	I	0.34	[0.054,1]
1031	E	0.024	[6.2e-10,0.26]
1032	E	0.024	[6.2e-10,0.26]
1033	S	0.027	[6.2e-10,0.26]

1034	D	0.026	[6.2e-10,0.26]
1035	L	0.035	[6.2e-10,0.26]
1036	I	0.024	[6.2e-10,0.26]
1037	I	0.02	[6.2e-10,0.26]
1038	P	0.033	[6.2e-10,0.26]
1039	K	0.023	[6.2e-10,0.26]
1040	S	0.035	[6.2e-10,0.26]
1041	L	0.037	[6.2e-10,0.26]
1042	A	0.029	[6.2e-10,0.26]
1043	G	0.027	[6.2e-10,0.26]
1044	P	0.032	[6.2e-10,0.26]
1045	L	0.032	[6.2e-10,0.26]
1046	S	0.024	[6.2e-10,0.26]
1047	H	0.031	[6.2e-10,0.26]
1048	H	0.03	[6.2e-10,0.26]
1049	N	0.024	[6.2e-10,0.26]
1050	T	0.026	[6.2e-10,0.26]
1051	R	0.026	[6.2e-10,0.26]
1052	E	0.022	[6.2e-10,0.26]
1053	G	0.028	[6.2e-10,0.26]
1054	Y	0.033	[6.2e-10,0.26]
1055	R	0.025	[6.2e-10,0.26]
1056	T	0.029	[6.2e-10,0.26]
1057	Q	0.035	[6.2e-10,0.26]
1058	V	0.57	[0.26,1]
1059	K	0.025	[6.2e-10,0.26]
1060	G	0.024	[6.2e-10,0.26]
1061	P	0.032	[6.2e-10,0.26]
1062	W	0.035	[6.2e-10,0.26]
1063	H	0.032	[6.2e-10,0.26]
1064	S	0.027	[6.2e-10,0.26]
1065	E	0.024	[6.2e-10,0.26]
1066	E	0.022	[6.2e-10,0.26]
1067	L	0.035	[6.2e-10,0.26]
1068	E	0.024	[6.2e-10,0.26]
1069	I	0.025	[6.2e-10,0.26]
1070	R	0.033	[6.2e-10,0.26]
1071	F	0.033	[6.2e-10,0.26]
1072	E	0.022	[6.2e-10,0.26]
1073	E	0.024	[6.2e-10,0.26]
1074	C	0.034	[6.2e-10,0.26]
1075	P	0.032	[6.2e-10,0.26]
1076	G	0.026	[6.2e-10,0.26]
1077	T	0.027	[6.2e-10,0.26]
1078	K	0.023	[6.2e-10,0.26]
1079	V	0.028	[6.2e-10,0.26]
1080	H	0.46	[0.054,1]

1081	V	0.023	[6.2e-10,0.26]
1082	E	0.022	[6.2e-10,0.26]
1083	E	0.023	[6.2e-10,0.26]
1084	T	0.026	[6.2e-10,0.26]
1085	C	0.033	[6.2e-10,0.26]
1086	G	0.026	[6.2e-10,0.26]
1087	T	0.028	[6.2e-10,0.26]
1088	R	0.026	[6.2e-10,0.26]
1089	G	0.027	[6.2e-10,0.26]
1090	P	0.032	[6.2e-10,0.26]
1091	S	0.036	[6.2e-10,0.26]
1092	L	0.035	[6.2e-10,0.26]
1093	R	0.026	[6.2e-10,0.26]
1094	S	0.035	[6.2e-10,0.26]
1095	T	0.027	[6.2e-10,0.26]
1096	T	0.03	[6.2e-10,0.26]
1097	A	0.025	[6.2e-10,0.26]
1098	S	0.026	[6.2e-10,0.26]
1099	G	0.027	[6.2e-10,0.26]
1100	R	0.023	[6.2e-10,0.26]
1101	V	0.025	[6.2e-10,0.26]
1102	I	0.025	[6.2e-10,0.26]
1103	E	0.022	[6.2e-10,0.26]
1104	E	0.024	[6.2e-10,0.26]
1105	W	0.035	[6.2e-10,0.26]
1106	C	0.032	[6.2e-10,0.26]
1107	C	0.034	[6.2e-10,0.26]
1108	R	0.024	[6.2e-10,0.26]
1109	E	0.024	[6.2e-10,0.26]
1110	C	0.032	[6.2e-10,0.26]
1111	T	0.026	[6.2e-10,0.26]
1112	M	0.018	[6.2e-10,0.26]
1113	P	0.033	[6.2e-10,0.26]
1114	P	0.032	[6.2e-10,0.26]
1115	L	0.036	[6.2e-10,0.26]
1116	S	0.032	[6.2e-10,0.26]
1117	F	0.031	[6.2e-10,0.26]
1118	R	0.29	[0.013,1]
1119	A	0.026	[6.2e-10,0.26]
1120	K	0.025	[6.2e-10,0.26]
1121	D	0.024	[6.2e-10,0.26]
1122	G	0.026	[6.2e-10,0.26]
1123	C	0.033	[6.2e-10,0.26]
1124	W	0.035	[6.2e-10,0.26]
1125	Y	0.036	[6.2e-10,0.26]
1126	G	0.027	[6.2e-10,0.26]
1127	M	0.018	[6.2e-10,0.26]

1128	E	0.022	[6.2e-10,0.26]
1129	I	0.02	[6.2e-10,0.26]
1130	R	0.023	[6.2e-10,0.26]
1131	P	0.033	[6.2e-10,0.26]
1132	R	0.024	[6.2e-10,0.26]
1133	K	0.024	[6.2e-10,0.26]
1134	E	0.024	[6.2e-10,0.26]
1135	P	0.032	[6.2e-10,0.26]
1136	E	0.023	[6.2e-10,0.26]
1137	S	0.025	[6.2e-10,0.26]
1138	N	0.024	[6.2e-10,0.26]
1139	L	0.037	[6.2e-10,0.26]
1140	V	0.024	[6.2e-10,0.26]
1141	R	0.023	[6.2e-10,0.26]
1142	S	0.035	[6.2e-10,0.26]
1143	M	0.17	[0.013,0.26]
1144	V	0.023	[6.2e-10,0.26]
1145	T	0.027	[6.2e-10,0.26]
1146	A	0.025	[6.2e-10,0.26]
1147	G	0.026	[6.2e-10,0.26]
1148	S	0.035	[6.2e-10,0.26]
1149	T	0.027	[6.2e-10,0.26]
1150	D	0.026	[6.2e-10,0.26]
1151	H	0.031	[6.2e-10,0.26]
1152	M	0.018	[6.2e-10,0.26]
1153	D	0.025	[6.2e-10,0.26]
1154	H	0.03	[6.2e-10,0.26]
1155	F	0.03	[6.2e-10,0.26]
1156	S	0.035	[6.2e-10,0.26]
1157	L	0.035	[6.2e-10,0.26]
1158	G	0.027	[6.2e-10,0.26]
1159	V	0.023	[6.2e-10,0.26]
1160	L	0.035	[6.2e-10,0.26]
1161	V	0.023	[6.2e-10,0.26]
1162	I	0.027	[6.2e-10,0.26]
1163	L	0.036	[6.2e-10,0.26]
1164	L	0.032	[6.2e-10,0.26]
1165	M	0.018	[6.2e-10,0.26]
1166	V	0.023	[6.2e-10,0.26]
1167	Q	0.033	[6.2e-10,0.26]
1168	E	0.024	[6.2e-10,0.26]
1169	G	0.025	[6.2e-10,0.26]
1170	L	0.035	[6.2e-10,0.26]
1171	K	0.023	[6.2e-10,0.26]
1172	K	0.023	[6.2e-10,0.26]
1173	R	0.026	[6.2e-10,0.26]
1174	M	0.018	[6.2e-10,0.26]

1175	T	0.026	[6.2e-10,0.26]
1176	T	0.026	[6.2e-10,0.26]
1177	K	0.023	[6.2e-10,0.26]
1178	I	0.024	[6.2e-10,0.26]
1179	I	0.024	[6.2e-10,0.26]
1180	I	0.28	[0.054,1]
1181	S	0.024	[6.2e-10,0.26]
1182	T	0.026	[6.2e-10,0.26]
1183	S	0.035	[6.2e-10,0.26]
1184	M	0.018	[6.2e-10,0.26]
1185	A	0.025	[6.2e-10,0.26]
1186	V	0.21	[0.013,1]
1187	L	0.035	[6.2e-10,0.26]
1188	V	0.025	[6.2e-10,0.26]
1189	A	0.23	[0.013,1]
1190	M	0.018	[6.2e-10,0.26]
1191	I	0.22	[0.013,1]
1192	L	0.035	[6.2e-10,0.26]
1193	G	0.027	[6.2e-10,0.26]
1194	G	0.027	[6.2e-10,0.26]
1195	F	0.032	[6.2e-10,0.26]
1196	S	0.29	[0.013,1]
1197	M	0.018	[6.2e-10,0.26]
1198	S	0.027	[6.2e-10,0.26]
1199	D	0.023	[6.2e-10,0.26]
1200	L	0.035	[6.2e-10,0.26]
1201	A	0.029	[6.2e-10,0.26]
1202	K	0.023	[6.2e-10,0.26]
1203	L	0.035	[6.2e-10,0.26]
1204	A	0.21	[0.013,1]
1205	I	0.025	[6.2e-10,0.26]
1206	L	0.035	[6.2e-10,0.26]
1207	M	0.018	[6.2e-10,0.26]
1208	G	0.03	[6.2e-10,0.26]
1209	A	0.026	[6.2e-10,0.26]
1210	T	0.028	[6.2e-10,0.26]
1211	F	0.03	[6.2e-10,0.26]
1212	A	0.025	[6.2e-10,0.26]
1213	E	0.024	[6.2e-10,0.26]
1214	M	0.018	[6.2e-10,0.26]
1215	N	0.024	[6.2e-10,0.26]
1216	T	0.03	[6.2e-10,0.26]
1217	G	0.027	[6.2e-10,0.26]
1218	G	0.027	[6.2e-10,0.26]
1219	D	0.026	[6.2e-10,0.26]
1220	V	0.024	[6.2e-10,0.26]
1221	A	0.029	[6.2e-10,0.26]

1222	H	0.031	[6.2e-10,0.26]
1223	L	0.035	[6.2e-10,0.26]
1224	A	0.025	[6.2e-10,0.26]
1225	L	0.035	[6.2e-10,0.26]
1226	I	0.57	[0.26,1]
1227	A	0.023	[6.2e-10,0.26]
1228	A	0.025	[6.2e-10,0.26]
1229	F	0.031	[6.2e-10,0.26]
1230	K	0.025	[6.2e-10,0.26]
1231	V	0.025	[6.2e-10,0.26]
1232	R	0.026	[6.2e-10,0.26]
1233	P	0.033	[6.2e-10,0.26]
1234	A	0.025	[6.2e-10,0.26]
1235	L	0.035	[6.2e-10,0.26]
1236	L	0.035	[6.2e-10,0.26]
1237	V	0.026	[6.2e-10,0.26]
1238	S	0.034	[6.2e-10,0.26]
1239	F	0.03	[6.2e-10,0.26]
1240	I	0.025	[6.2e-10,0.26]
1241	F	0.26	[0.013,1]
1242	R	0.026	[6.2e-10,0.26]
1243	A	0.026	[6.2e-10,0.26]
1244	N	0.026	[6.2e-10,0.26]
1245	W	0.035	[6.2e-10,0.26]
1246	T	0.026	[6.2e-10,0.26]
1247	P	0.033	[6.2e-10,0.26]
1248	R	0.036	[6.2e-10,0.26]
1249	E	0.022	[6.2e-10,0.26]
1250	S	0.024	[6.2e-10,0.26]
1251	M	0.018	[6.2e-10,0.26]
1252	L	0.035	[6.2e-10,0.26]
1253	L	0.036	[6.2e-10,0.26]
1254	A	0.026	[6.2e-10,0.26]
1255	L	0.035	[6.2e-10,0.26]
1256	A	0.028	[6.2e-10,0.26]
1257	S	0.032	[6.2e-10,0.26]
1258	C	0.035	[6.2e-10,0.26]
1259	L	0.28	[0.013,1]
1260	L	0.035	[6.2e-10,0.26]
1261	Q	0.035	[6.2e-10,0.26]
1262	T	0.03	[6.2e-10,0.26]
1263	X	0.024	[6.2e-10,0.26]
1264	I	0.024	[6.2e-10,0.26]
1265	S	0.034	[6.2e-10,0.26]
1266	A	0.028	[6.2e-10,0.26]
1267	L	0.035	[6.2e-10,0.26]
1268	E	0.024	[6.2e-10,0.26]

1269	G	0.027	[6.2e-10,0.26]
1270	D	0.2	[0.013,0.26]
1271	L	0.035	[6.2e-10,0.26]
1272	M	0.018	[6.2e-10,0.26]
1273	V	0.027	[6.2e-10,0.26]
1274	L	0.27	[0.013,1]
1275	I	0.4	[0.054,1]
1276	N	0.027	[6.2e-10,0.26]
1277	G	0.028	[6.2e-10,0.26]
1278	F	0.033	[6.2e-10,0.26]
1279	A	0.029	[6.2e-10,0.26]
1280	L	0.035	[6.2e-10,0.26]
1281	A	0.026	[6.2e-10,0.26]
1282	W	0.035	[6.2e-10,0.26]
1283	L	0.035	[6.2e-10,0.26]
1284	A	0.025	[6.2e-10,0.26]
1285	I	0.19	[0.013,0.26]
1286	R	0.04	[6.2e-10,0.26]
1287	A	0.025	[6.2e-10,0.26]
1288	M	0.018	[6.2e-10,0.26]
1289	A	0.23	[0.013,1]
1290	V	0.025	[6.2e-10,0.26]
1291	P	0.032	[6.2e-10,0.26]
1292	R	0.033	[6.2e-10,0.26]
1293	T	0.03	[6.2e-10,0.26]
1294	D	0.023	[6.2e-10,0.26]
1295	N	0.024	[6.2e-10,0.26]
1296	I	0.025	[6.2e-10,0.26]
1297	T	0.24	[0.013,1]
1298	L	0.036	[6.2e-10,0.26]
1299	A	0.22	[0.013,1]
1300	I	0.37	[0.054,1]
1301	L	0.035	[6.2e-10,0.26]
1302	A	0.24	[0.013,1]
1303	A	0.029	[6.2e-10,0.26]
1304	L	0.036	[6.2e-10,0.26]
1305	T	0.026	[6.2e-10,0.26]
1306	P	0.032	[6.2e-10,0.26]
1307	L	0.036	[6.2e-10,0.26]
1308	A	0.026	[6.2e-10,0.26]
1309	R	0.035	[6.2e-10,0.26]
1310	G	0.026	[6.2e-10,0.26]
1311	T	0.026	[6.2e-10,0.26]
1312	L	0.035	[6.2e-10,0.26]
1313	L	0.033	[6.2e-10,0.26]
1314	V	0.024	[6.2e-10,0.26]
1315	A	0.025	[6.2e-10,0.26]

1316	W	0.035	[6.2e-10,0.26]
1317	R	0.026	[6.2e-10,0.26]
1318	A	0.024	[6.2e-10,0.26]
1319	G	0.026	[6.2e-10,0.26]
1320	L	0.033	[6.2e-10,0.26]
1321	A	0.028	[6.2e-10,0.26]
1322	T	0.03	[6.2e-10,0.26]
1323	C	0.035	[6.2e-10,0.26]
1324	G	0.026	[6.2e-10,0.26]
1325	G	0.024	[6.2e-10,0.26]
1326	F	0.25	[0.013,1]
1327	M	0.018	[6.2e-10,0.26]
1328	L	0.032	[6.2e-10,0.26]
1329	L	0.27	[0.013,1]
1330	S	0.034	[6.2e-10,0.26]
1331	L	0.035	[6.2e-10,0.26]
1332	K	0.024	[6.2e-10,0.26]
1333	G	0.024	[6.2e-10,0.26]
1334	K	0.025	[6.2e-10,0.26]
1335	G	0.029	[6.2e-10,0.26]
1336	S	0.027	[6.2e-10,0.26]
1337	V	0.023	[6.2e-10,0.26]
1338	K	0.023	[6.2e-10,0.26]
1339	K	0.023	[6.2e-10,0.26]
1340	N	0.024	[6.2e-10,0.26]
1341	L	0.036	[6.2e-10,0.26]
1342	P	0.033	[6.2e-10,0.26]
1343	F	0.033	[6.2e-10,0.26]
1344	V	0.025	[6.2e-10,0.26]
1345	M	0.018	[6.2e-10,0.26]
1346	A	0.026	[6.2e-10,0.26]
1347	L	0.035	[6.2e-10,0.26]
1348	G	0.026	[6.2e-10,0.26]
1349	L	0.29	[0.013,1]
1350	T	0.22	[0.013,1]
1351	A	0.029	[6.2e-10,0.26]
1352	V	0.023	[6.2e-10,0.26]
1353	R	0.023	[6.2e-10,0.26]
1354	L	0.47	[0.054,1]
1355	V	0.025	[6.2e-10,0.26]
1356	D	0.023	[6.2e-10,0.26]
1357	P	0.033	[6.2e-10,0.26]
1358	I	0.026	[6.2e-10,0.26]
1359	N	0.025	[6.2e-10,0.26]
1360	V	0.023	[6.2e-10,0.26]
1361	V	0.024	[6.2e-10,0.26]
1362	G	0.027	[6.2e-10,0.26]

1363	L	0.036	[6.2e-10,0.26]
1364	L	0.035	[6.2e-10,0.26]
1365	L	0.036	[6.2e-10,0.26]
1366	L	0.032	[6.2e-10,0.26]
1367	T	0.026	[6.2e-10,0.26]
1368	R	0.024	[6.2e-10,0.26]
1369	S	0.027	[6.2e-10,0.26]
1370	G	0.024	[6.2e-10,0.26]
1371	K	0.023	[6.2e-10,0.26]
1372	R	0.033	[6.2e-10,0.26]
1373	S	0.024	[6.2e-10,0.26]
1374	W	0.035	[6.2e-10,0.26]
1375	P	0.033	[6.2e-10,0.26]
1376	P	0.036	[6.2e-10,0.26]
1377	S	0.027	[6.2e-10,0.26]
1378	E	0.024	[6.2e-10,0.26]
1379	V	0.025	[6.2e-10,0.26]
1380	L	0.033	[6.2e-10,0.26]
1381	T	0.026	[6.2e-10,0.26]
1382	A	0.029	[6.2e-10,0.26]
1383	V	0.029	[6.2e-10,0.26]
1384	G	0.026	[6.2e-10,0.26]
1385	L	0.035	[6.2e-10,0.26]
1386	I	0.02	[6.2e-10,0.26]
1387	C	0.035	[6.2e-10,0.26]
1388	A	0.025	[6.2e-10,0.26]
1389	L	0.035	[6.2e-10,0.26]
1390	A	0.026	[6.2e-10,0.26]
1391	G	0.027	[6.2e-10,0.26]
1392	G	0.024	[6.2e-10,0.26]
1393	F	0.031	[6.2e-10,0.26]
1394	A	0.026	[6.2e-10,0.26]
1395	K	0.023	[6.2e-10,0.26]
1396	A	0.025	[6.2e-10,0.26]
1397	D	0.024	[6.2e-10,0.26]
1398	I	0.022	[6.2e-10,0.26]
1399	E	0.022	[6.2e-10,0.26]
1400	M	0.018	[6.2e-10,0.26]
1401	A	0.029	[6.2e-10,0.26]
1402	G	0.024	[6.2e-10,0.26]
1403	P	0.033	[6.2e-10,0.26]
1404	M	0.018	[6.2e-10,0.26]
1405	A	0.028	[6.2e-10,0.26]
1406	A	0.025	[6.2e-10,0.26]
1407	V	0.026	[6.2e-10,0.26]
1408	G	0.027	[6.2e-10,0.26]
1409	L	0.035	[6.2e-10,0.26]

1410	L	0.037	[6.2e-10,0.26]
1411	I	0.027	[6.2e-10,0.26]
1412	V	0.026	[6.2e-10,0.26]
1413	S	0.027	[6.2e-10,0.26]
1414	Y	0.035	[6.2e-10,0.26]
1415	V	0.023	[6.2e-10,0.26]
1416	V	0.025	[6.2e-10,0.26]
1417	S	0.28	[0.013,1]
1418	G	0.027	[6.2e-10,0.26]
1419	K	0.023	[6.2e-10,0.26]
1420	S	0.027	[6.2e-10,0.26]
1421	V	0.023	[6.2e-10,0.26]
1422	D	0.023	[6.2e-10,0.26]
1423	M	0.018	[6.2e-10,0.26]
1424	Y	0.032	[6.2e-10,0.26]
1425	I	0.027	[6.2e-10,0.26]
1426	E	0.024	[6.2e-10,0.26]
1427	R	0.026	[6.2e-10,0.26]
1428	A	0.025	[6.2e-10,0.26]
1429	G	0.03	[6.2e-10,0.26]
1430	D	0.024	[6.2e-10,0.26]
1431	I	0.024	[6.2e-10,0.26]
1432	T	0.026	[6.2e-10,0.26]
1433	W	0.035	[6.2e-10,0.26]
1434	E	0.024	[6.2e-10,0.26]
1435	K	0.024	[6.2e-10,0.26]
1436	D	0.024	[6.2e-10,0.26]
1437	A	0.024	[6.2e-10,0.26]
1438	E	0.024	[6.2e-10,0.26]
1439	V	0.23	[0.013,1]
1440	T	0.03	[6.2e-10,0.26]
1441	G	0.027	[6.2e-10,0.26]
1442	N	0.024	[6.2e-10,0.26]
1443	S	0.027	[6.2e-10,0.26]
1444	P	0.034	[6.2e-10,0.26]
1445	R	0.033	[6.2e-10,0.26]
1446	L	0.034	[6.2e-10,0.26]
1447	D	0.024	[6.2e-10,0.26]
1448	V	0.023	[6.2e-10,0.26]
1449	A	0.025	[6.2e-10,0.26]
1450	L	0.037	[6.2e-10,0.26]
1451	D	0.026	[6.2e-10,0.26]
1452	E	0.022	[6.2e-10,0.26]
1453	S	0.027	[6.2e-10,0.26]
1454	G	0.03	[6.2e-10,0.26]
1455	D	0.026	[6.2e-10,0.26]
1456	F	0.03	[6.2e-10,0.26]

1457	S	0.033	[6.2e-10,0.26]
1458	L	0.035	[6.2e-10,0.26]
1459	V	0.024	[6.2e-10,0.26]
1460	E	0.022	[6.2e-10,0.26]
1461	D	0.21	[0.013,1]
1462	D	0.026	[6.2e-10,0.26]
1463	G	0.029	[6.2e-10,0.26]
1464	P	0.033	[6.2e-10,0.26]
1465	P	0.033	[6.2e-10,0.26]
1466	M	0.018	[6.2e-10,0.26]
1467	R	0.026	[6.2e-10,0.26]
1468	E	0.022	[6.2e-10,0.26]
1469	I	0.024	[6.2e-10,0.26]
1470	I	0.02	[6.2e-10,0.26]
1471	L	0.032	[6.2e-10,0.26]
1472	K	0.023	[6.2e-10,0.26]
1473	V	0.023	[6.2e-10,0.26]
1474	V	0.025	[6.2e-10,0.26]
1475	L	0.035	[6.2e-10,0.26]
1476	M	0.018	[6.2e-10,0.26]
1477	T	0.22	[0.013,1]
1478	I	0.024	[6.2e-10,0.26]
1479	C	0.035	[6.2e-10,0.26]
1480	G	0.026	[6.2e-10,0.26]
1481	M	0.018	[6.2e-10,0.26]
1482	N	0.024	[6.2e-10,0.26]
1483	P	0.032	[6.2e-10,0.26]
1484	I	0.02	[6.2e-10,0.26]
1485	A	0.026	[6.2e-10,0.26]
1486	I	0.02	[6.2e-10,0.26]
1487	P	0.033	[6.2e-10,0.26]
1488	F	0.032	[6.2e-10,0.26]
1489	A	0.028	[6.2e-10,0.26]
1490	A	0.027	[6.2e-10,0.26]
1491	G	0.027	[6.2e-10,0.26]
1492	A	0.023	[6.2e-10,0.26]
1493	W	0.035	[6.2e-10,0.26]
1494	Y	0.035	[6.2e-10,0.26]
1495	V	0.024	[6.2e-10,0.26]
1496	Y	0.036	[6.2e-10,0.26]
1497	V	0.024	[6.2e-10,0.26]
1498	K	0.023	[6.2e-10,0.26]
1499	T	0.03	[6.2e-10,0.26]
1500	G	0.025	[6.2e-10,0.26]
1501	K	0.024	[6.2e-10,0.26]
1502	R	0.023	[6.2e-10,0.26]
1503	S	0.027	[6.2e-10,0.26]

1504	G	0.029	[6.2e-10,0.26]
1505	A	0.027	[6.2e-10,0.26]
1506	L	0.034	[6.2e-10,0.26]
1507	W	0.035	[6.2e-10,0.26]
1508	D	0.025	[6.2e-10,0.26]
1509	V	0.023	[6.2e-10,0.26]
1510	P	0.036	[6.2e-10,0.26]
1511	A	0.029	[6.2e-10,0.26]
1512	P	0.033	[6.2e-10,0.26]
1513	K	0.024	[6.2e-10,0.26]
1514	E	0.024	[6.2e-10,0.26]
1515	V	0.024	[6.2e-10,0.26]
1516	K	0.024	[6.2e-10,0.26]
1517	K	0.024	[6.2e-10,0.26]
1518	G	0.026	[6.2e-10,0.26]
1519	E	0.022	[6.2e-10,0.26]
1520	T	0.027	[6.2e-10,0.26]
1521	T	0.026	[6.2e-10,0.26]
1522	D	0.026	[6.2e-10,0.26]
1523	G	0.026	[6.2e-10,0.26]
1524	V	0.024	[6.2e-10,0.26]
1525	Y	0.033	[6.2e-10,0.26]
1526	R	0.026	[6.2e-10,0.26]
1527	V	0.024	[6.2e-10,0.26]
1528	M	0.018	[6.2e-10,0.26]
1529	T	0.029	[6.2e-10,0.26]
1530	R	0.034	[6.2e-10,0.26]
1531	R	0.026	[6.2e-10,0.26]
1532	L	0.035	[6.2e-10,0.26]
1533	L	0.036	[6.2e-10,0.26]
1534	G	0.03	[6.2e-10,0.26]
1535	S	0.035	[6.2e-10,0.26]
1536	T	0.026	[6.2e-10,0.26]
1537	Q	0.034	[6.2e-10,0.26]
1538	V	0.029	[6.2e-10,0.26]
1539	G	0.027	[6.2e-10,0.26]
1540	V	0.023	[6.2e-10,0.26]
1541	G	0.026	[6.2e-10,0.26]
1542	V	0.026	[6.2e-10,0.26]
1543	M	0.018	[6.2e-10,0.26]
1544	Q	0.035	[6.2e-10,0.26]
1545	E	0.022	[6.2e-10,0.26]
1546	G	0.026	[6.2e-10,0.26]
1547	V	0.025	[6.2e-10,0.26]
1548	F	0.03	[6.2e-10,0.26]
1549	H	0.03	[6.2e-10,0.26]
1550	T	0.027	[6.2e-10,0.26]

1551	M	0.018	[6.2e-10,0.26]
1552	W	0.035	[6.2e-10,0.26]
1553	H	0.03	[6.2e-10,0.26]
1554	V	0.026	[6.2e-10,0.26]
1555	T	0.026	[6.2e-10,0.26]
1556	K	0.025	[6.2e-10,0.26]
1557	G	0.027	[6.2e-10,0.26]
1558	S	0.25	[0.013,1]
1559	A	0.025	[6.2e-10,0.26]
1560	L	0.035	[6.2e-10,0.26]
1561	R	0.024	[6.2e-10,0.26]
1562	S	0.024	[6.2e-10,0.26]
1563	G	0.03	[6.2e-10,0.26]
1564	E	0.024	[6.2e-10,0.26]
1565	G	0.025	[6.2e-10,0.26]
1566	R	0.026	[6.2e-10,0.26]
1567	L	0.035	[6.2e-10,0.26]
1568	D	0.026	[6.2e-10,0.26]
1569	P	0.032	[6.2e-10,0.26]
1570	Y	0.033	[6.2e-10,0.26]
1571	W	0.035	[6.2e-10,0.26]
1572	G	0.025	[6.2e-10,0.26]
1573	D	0.025	[6.2e-10,0.26]
1574	V	0.026	[6.2e-10,0.26]
1575	K	0.023	[6.2e-10,0.26]
1576	Q	0.033	[6.2e-10,0.26]
1577	D	0.024	[6.2e-10,0.26]
1578	L	0.035	[6.2e-10,0.26]
1579	V	0.023	[6.2e-10,0.26]
1580	S	0.035	[6.2e-10,0.26]
1581	Y	0.033	[6.2e-10,0.26]
1582	C	0.035	[6.2e-10,0.26]
1583	G	0.025	[6.2e-10,0.26]
1584	P	0.032	[6.2e-10,0.26]
1585	W	0.035	[6.2e-10,0.26]
1586	K	0.023	[6.2e-10,0.26]
1587	L	0.036	[6.2e-10,0.26]
1588	D	0.026	[6.2e-10,0.26]
1589	A	0.025	[6.2e-10,0.26]
1590	A	0.23	[0.013,1]
1591	W	0.035	[6.2e-10,0.26]
1592	D	0.025	[6.2e-10,0.26]
1593	G	0.026	[6.2e-10,0.26]
1594	H	0.29	[0.013,1]
1595	S	0.026	[6.2e-10,0.26]
1596	E	0.023	[6.2e-10,0.26]
1597	V	0.024	[6.2e-10,0.26]

1598	Q	0.033	[6.2e-10,0.26]
1599	L	0.034	[6.2e-10,0.26]
1600	L	0.035	[6.2e-10,0.26]
1601	A	0.026	[6.2e-10,0.26]
1602	V	0.025	[6.2e-10,0.26]
1603	P	0.033	[6.2e-10,0.26]
1604	P	0.033	[6.2e-10,0.26]
1605	G	0.027	[6.2e-10,0.26]
1606	E	0.022	[6.2e-10,0.26]
1607	R	0.024	[6.2e-10,0.26]
1608	A	0.026	[6.2e-10,0.26]
1609	R	0.22	[0.013,1]
1610	N	0.024	[6.2e-10,0.26]
1611	I	0.026	[6.2e-10,0.26]
1612	Q	0.033	[6.2e-10,0.26]
1613	T	0.029	[6.2e-10,0.26]
1614	L	0.29	[0.013,1]
1615	P	0.035	[6.2e-10,0.26]
1616	G	0.027	[6.2e-10,0.26]
1617	I	0.19	[0.013,0.26]
1618	F	0.03	[6.2e-10,0.26]
1619	K	0.023	[6.2e-10,0.26]
1620	T	0.026	[6.2e-10,0.26]
1621	K	0.023	[6.2e-10,0.26]
1622	D	0.026	[6.2e-10,0.26]
1623	G	0.024	[6.2e-10,0.26]
1624	D	0.023	[6.2e-10,0.26]
1625	I	0.025	[6.2e-10,0.26]
1626	G	0.027	[6.2e-10,0.26]
1627	A	0.025	[6.2e-10,0.26]
1628	V	0.029	[6.2e-10,0.26]
1629	A	0.028	[6.2e-10,0.26]
1630	L	0.036	[6.2e-10,0.26]
1631	D	0.023	[6.2e-10,0.26]
1632	Y	0.033	[6.2e-10,0.26]
1633	P	0.033	[6.2e-10,0.26]
1634	A	0.025	[6.2e-10,0.26]
1635	G	0.026	[6.2e-10,0.26]
1636	T	0.028	[6.2e-10,0.26]
1637	S	0.035	[6.2e-10,0.26]
1638	G	0.027	[6.2e-10,0.26]
1639	S	0.036	[6.2e-10,0.26]
1640	P	0.031	[6.2e-10,0.26]
1641	I	0.024	[6.2e-10,0.26]
1642	L	0.037	[6.2e-10,0.26]
1643	D	0.023	[6.2e-10,0.26]
1644	K	0.024	[6.2e-10,0.26]

1645	C	0.034	[6.2e-10,0.26]
1646	G	0.026	[6.2e-10,0.26]
1647	R	0.026	[6.2e-10,0.26]
1648	V	0.023	[6.2e-10,0.26]
1649	I	0.02	[6.2e-10,0.26]
1650	G	0.027	[6.2e-10,0.26]
1651	L	0.034	[6.2e-10,0.26]
1652	Y	0.036	[6.2e-10,0.26]
1653	G	0.026	[6.2e-10,0.26]
1654	N	0.027	[6.2e-10,0.26]
1655	G	0.024	[6.2e-10,0.26]
1656	V	0.028	[6.2e-10,0.26]
1657	V	0.023	[6.2e-10,0.26]
1658	I	0.024	[6.2e-10,0.26]
1659	K	0.023	[6.2e-10,0.26]
1660	N	0.027	[6.2e-10,0.26]
1661	G	0.026	[6.2e-10,0.26]
1662	S	0.025	[6.2e-10,0.26]
1663	Y	0.036	[6.2e-10,0.26]
1664	V	0.029	[6.2e-10,0.26]
1665	S	0.027	[6.2e-10,0.26]
1666	A	0.026	[6.2e-10,0.26]
1667	I	0.022	[6.2e-10,0.26]
1668	T	0.026	[6.2e-10,0.26]
1669	Q	0.034	[6.2e-10,0.26]
1670	G	0.026	[6.2e-10,0.26]
1671	R	0.21	[0.013,1]
1672	R	0.023	[6.2e-10,0.26]
1673	E	0.022	[6.2e-10,0.26]
1674	E	0.023	[6.2e-10,0.26]
1675	E	0.022	[6.2e-10,0.26]
1676	T	0.26	[0.013,1]
1677	P	0.033	[6.2e-10,0.26]
1678	V	0.029	[6.2e-10,0.26]
1679	E	0.023	[6.2e-10,0.26]
1680	C	0.033	[6.2e-10,0.26]
1681	F	0.03	[6.2e-10,0.26]
1682	E	0.024	[6.2e-10,0.26]
1683	P	0.034	[6.2e-10,0.26]
1684	S	0.032	[6.2e-10,0.26]
1685	M	0.018	[6.2e-10,0.26]
1686	L	0.035	[6.2e-10,0.26]
1687	K	0.21	[0.013,1]
1688	K	0.023	[6.2e-10,0.26]
1689	K	0.023	[6.2e-10,0.26]
1690	Q	0.033	[6.2e-10,0.26]
1691	L	0.037	[6.2e-10,0.26]

1692	T	0.029	[6.2e-10,0.26]
1693	V	0.025	[6.2e-10,0.26]
1694	L	0.035	[6.2e-10,0.26]
1695	D	0.025	[6.2e-10,0.26]
1696	L	0.035	[6.2e-10,0.26]
1697	H	0.033	[6.2e-10,0.26]
1698	P	0.034	[6.2e-10,0.26]
1699	G	0.027	[6.2e-10,0.26]
1700	A	0.027	[6.2e-10,0.26]
1701	G	0.025	[6.2e-10,0.26]
1702	K	0.024	[6.2e-10,0.26]
1703	T	0.027	[6.2e-10,0.26]
1704	R	0.023	[6.2e-10,0.26]
1705	R	0.025	[6.2e-10,0.26]
1706	V	0.029	[6.2e-10,0.26]
1707	L	0.035	[6.2e-10,0.26]
1708	P	0.036	[6.2e-10,0.26]
1709	E	0.024	[6.2e-10,0.26]
1710	I	0.02	[6.2e-10,0.26]
1711	V	0.025	[6.2e-10,0.26]
1712	R	0.036	[6.2e-10,0.26]
1713	E	0.024	[6.2e-10,0.26]
1714	A	0.026	[6.2e-10,0.26]
1715	I	0.02	[6.2e-10,0.26]
1716	K	0.024	[6.2e-10,0.26]
1717	T	0.22	[0.013,1]
1718	R	0.026	[6.2e-10,0.26]
1719	L	0.032	[6.2e-10,0.26]
1720	R	0.034	[6.2e-10,0.26]
1721	T	0.026	[6.2e-10,0.26]
1722	V	0.21	[0.013,1]
1723	I	0.024	[6.2e-10,0.26]
1724	L	0.036	[6.2e-10,0.26]
1725	A	0.027	[6.2e-10,0.26]
1726	P	0.032	[6.2e-10,0.26]
1727	T	0.026	[6.2e-10,0.26]
1728	R	0.25	[0.013,1]
1729	V	0.029	[6.2e-10,0.26]
1730	V	0.25	[0.013,1]
1731	A	0.47	[0.054,1]
1732	A	0.25	[0.013,1]
1733	E	0.34	[0.054,1]
1734	M	0.3	[0.054,1]
1735	E	0.33	[0.054,1]
1736	E	0.36	[0.054,1]
1737	A	0.22	[0.013,1]
1738	L	0.036	[6.2e-10,0.26]

1739	R	0.22	[0.013,1]
1740	G	0.39	[0.054,1]
1741	L	0.53	[0.054,1]
1742	P	0.49	[0.054,1]
1743	V	0.21	[0.013,1]
1744	R	0.036	[6.2e-10,0.26]
1745	Y	0.51	[0.054,1]
1746	M	0.28	[0.054,1]
1747	T	0.026	[6.2e-10,0.26]
1748	T	0.026	[6.2e-10,0.26]
1749	A	0.025	[6.2e-10,0.26]
1750	V	0.026	[6.2e-10,0.26]
1751	N	0.025	[6.2e-10,0.26]
1752	V	0.025	[6.2e-10,0.26]
1753	T	0.026	[6.2e-10,0.26]
1754	H	0.033	[6.2e-10,0.26]
1755	S	0.036	[6.2e-10,0.26]
1756	G	0.024	[6.2e-10,0.26]
1757	T	0.026	[6.2e-10,0.26]
1758	E	0.024	[6.2e-10,0.26]
1759	I	0.024	[6.2e-10,0.26]
1760	V	0.029	[6.2e-10,0.26]
1761	D	0.025	[6.2e-10,0.26]
1762	L	0.036	[6.2e-10,0.26]
1763	M	0.018	[6.2e-10,0.26]
1764	C	0.032	[6.2e-10,0.26]
1765	H	0.033	[6.2e-10,0.26]
1766	A	0.026	[6.2e-10,0.26]
1767	T	0.028	[6.2e-10,0.26]
1768	F	0.03	[6.2e-10,0.26]
1769	T	0.03	[6.2e-10,0.26]
1770	S	0.035	[6.2e-10,0.26]
1771	R	0.034	[6.2e-10,0.26]
1772	L	0.037	[6.2e-10,0.26]
1773	L	0.037	[6.2e-10,0.26]
1774	Q	0.035	[6.2e-10,0.26]
1775	P	0.033	[6.2e-10,0.26]
1776	I	0.024	[6.2e-10,0.26]
1777	R	0.026	[6.2e-10,0.26]
1778	V	0.025	[6.2e-10,0.26]
1779	P	0.034	[6.2e-10,0.26]
1780	N	0.025	[6.2e-10,0.26]
1781	Y	0.033	[6.2e-10,0.26]
1782	N	0.026	[6.2e-10,0.26]
1783	L	0.033	[6.2e-10,0.26]
1784	Y	0.91	[0.26,1]
1785	I	0.025	[6.2e-10,0.26]

1786	M	0.018	[6.2e-10,0.26]
1787	D	0.026	[6.2e-10,0.26]
1788	E	0.023	[6.2e-10,0.26]
1789	A	0.026	[6.2e-10,0.26]
1790	H	0.03	[6.2e-10,0.26]
1791	F	0.03	[6.2e-10,0.26]
1792	T	0.026	[6.2e-10,0.26]
1793	D	0.024	[6.2e-10,0.26]
1794	P	0.033	[6.2e-10,0.26]
1795	S	0.035	[6.2e-10,0.26]
1796	S	0.027	[6.2e-10,0.26]
1797	I	0.02	[6.2e-10,0.26]
1798	A	0.028	[6.2e-10,0.26]
1799	A	0.025	[6.2e-10,0.26]
1800	R	0.026	[6.2e-10,0.26]
1801	G	0.027	[6.2e-10,0.26]
1802	Y	0.033	[6.2e-10,0.26]
1803	I	0.021	[6.2e-10,0.26]
1804	S	0.035	[6.2e-10,0.26]
1805	T	0.026	[6.2e-10,0.26]
1806	R	0.023	[6.2e-10,0.26]
1807	V	0.029	[6.2e-10,0.26]
1808	E	0.023	[6.2e-10,0.26]
1809	M	0.018	[6.2e-10,0.26]
1810	G	0.026	[6.2e-10,0.26]
1811	E	0.022	[6.2e-10,0.26]
1812	A	0.023	[6.2e-10,0.26]
1813	A	0.029	[6.2e-10,0.26]
1814	A	0.026	[6.2e-10,0.26]
1815	I	0.026	[6.2e-10,0.26]
1816	F	0.031	[6.2e-10,0.26]
1817	M	0.018	[6.2e-10,0.26]
1818	T	0.028	[6.2e-10,0.26]
1819	A	0.026	[6.2e-10,0.26]
1820	T	0.025	[6.2e-10,0.26]
1821	P	0.032	[6.2e-10,0.26]
1822	P	0.032	[6.2e-10,0.26]
1823	G	0.027	[6.2e-10,0.26]
1824	T	0.026	[6.2e-10,0.26]
1825	R	0.036	[6.2e-10,0.26]
1826	D	0.025	[6.2e-10,0.26]
1827	A	0.024	[6.2e-10,0.26]
1828	F	0.032	[6.2e-10,0.26]
1829	P	0.033	[6.2e-10,0.26]
1830	D	0.023	[6.2e-10,0.26]
1831	S	0.035	[6.2e-10,0.26]
1832	N	0.024	[6.2e-10,0.26]

1833	S	0.035	[6.2e-10,0.26]
1834	P	0.032	[6.2e-10,0.26]
1835	I	0.025	[6.2e-10,0.26]
1836	M	0.018	[6.2e-10,0.26]
1837	D	0.023	[6.2e-10,0.26]
1838	T	0.026	[6.2e-10,0.26]
1839	E	0.024	[6.2e-10,0.26]
1840	V	0.023	[6.2e-10,0.26]
1841	E	0.024	[6.2e-10,0.26]
1842	V	0.026	[6.2e-10,0.26]
1843	P	0.032	[6.2e-10,0.26]
1844	E	0.022	[6.2e-10,0.26]
1845	R	0.026	[6.2e-10,0.26]
1846	A	0.026	[6.2e-10,0.26]
1847	W	0.035	[6.2e-10,0.26]
1848	S	0.024	[6.2e-10,0.26]
1849	S	0.29	[0.013,1]
1850	G	0.026	[6.2e-10,0.26]
1851	F	0.033	[6.2e-10,0.26]
1852	D	0.025	[6.2e-10,0.26]
1853	W	0.035	[6.2e-10,0.26]
1854	V	0.023	[6.2e-10,0.26]
1855	T	0.025	[6.2e-10,0.26]
1856	D	0.024	[6.2e-10,0.26]
1857	H	0.27	[0.013,1]
1858	S	0.036	[6.2e-10,0.26]
1859	G	0.025	[6.2e-10,0.26]
1860	K	0.025	[6.2e-10,0.26]
1861	T	0.026	[6.2e-10,0.26]
1862	V	0.25	[0.013,1]
1863	W	0.035	[6.2e-10,0.26]
1864	F	0.032	[6.2e-10,0.26]
1865	V	0.029	[6.2e-10,0.26]
1866	P	0.032	[6.2e-10,0.26]
1867	S	0.026	[6.2e-10,0.26]
1868	V	0.023	[6.2e-10,0.26]
1869	R	0.025	[6.2e-10,0.26]
1870	N	0.82	[0.26,1]
1871	G	0.026	[6.2e-10,0.26]
1872	N	0.85	[0.26,1]
1873	E	0.023	[6.2e-10,0.26]
1874	I	0.36	[0.054,1]
1875	A	0.22	[0.013,1]
1876	A	0.027	[6.2e-10,0.26]
1877	C	0.035	[6.2e-10,0.26]
1878	L	0.035	[6.2e-10,0.26]
1879	T	0.026	[6.2e-10,0.26]

1880	K	0.023	[6.2e-10,0.26]
1881	A	0.029	[6.2e-10,0.26]
1882	G	0.027	[6.2e-10,0.26]
1883	K	0.023	[6.2e-10,0.26]
1884	R	0.033	[6.2e-10,0.26]
1885	V	0.026	[6.2e-10,0.26]
1886	I	0.02	[6.2e-10,0.26]
1887	Q	0.033	[6.2e-10,0.26]
1888	L	0.032	[6.2e-10,0.26]
1889	S	0.024	[6.2e-10,0.26]
1890	R	0.024	[6.2e-10,0.26]
1891	K	0.023	[6.2e-10,0.26]
1892	T	0.03	[6.2e-10,0.26]
1893	F	0.033	[6.2e-10,0.26]
1894	E	0.022	[6.2e-10,0.26]
1895	T	0.026	[6.2e-10,0.26]
1896	E	0.022	[6.2e-10,0.26]
1897	F	0.031	[6.2e-10,0.26]
1898	Q	0.28	[0.013,1]
1899	K	0.024	[6.2e-10,0.26]
1900	T	0.026	[6.2e-10,0.26]
1901	K	0.025	[6.2e-10,0.26]
1902	N	0.23	[0.013,1]
1903	Q	0.035	[6.2e-10,0.26]
1904	E	0.022	[6.2e-10,0.26]
1905	W	0.035	[6.2e-10,0.26]
1906	D	0.023	[6.2e-10,0.26]
1907	F	0.032	[6.2e-10,0.26]
1908	V	0.025	[6.2e-10,0.26]
1909	V	0.2	[0.013,0.26]
1910	T	0.026	[6.2e-10,0.26]
1911	T	0.03	[6.2e-10,0.26]
1912	D	0.023	[6.2e-10,0.26]
1913	I	0.026	[6.2e-10,0.26]
1914	S	0.035	[6.2e-10,0.26]
1915	E	0.022	[6.2e-10,0.26]
1916	M	0.018	[6.2e-10,0.26]
1917	G	0.027	[6.2e-10,0.26]
1918	A	0.025	[6.2e-10,0.26]
1919	N	0.025	[6.2e-10,0.26]
1920	F	0.03	[6.2e-10,0.26]
1921	K	0.024	[6.2e-10,0.26]
1922	A	0.029	[6.2e-10,0.26]
1923	D	0.023	[6.2e-10,0.26]
1924	R	0.033	[6.2e-10,0.26]
1925	V	0.025	[6.2e-10,0.26]
1926	I	0.02	[6.2e-10,0.26]

1927	D	0.025	[6.2e-10,0.26]
1928	S	0.034	[6.2e-10,0.26]
1929	R	0.023	[6.2e-10,0.26]
1930	R	0.026	[6.2e-10,0.26]
1931	C	0.032	[6.2e-10,0.26]
1932	L	0.037	[6.2e-10,0.26]
1933	K	0.023	[6.2e-10,0.26]
1934	P	0.032	[6.2e-10,0.26]
1935	V	0.026	[6.2e-10,0.26]
1936	I	0.02	[6.2e-10,0.26]
1937	L	0.035	[6.2e-10,0.26]
1938	D	0.026	[6.2e-10,0.26]
1939	G	0.028	[6.2e-10,0.26]
1940	E	0.022	[6.2e-10,0.26]
1941	R	0.026	[6.2e-10,0.26]
1942	V	0.025	[6.2e-10,0.26]
1943	I	0.025	[6.2e-10,0.26]
1944	L	0.035	[6.2e-10,0.26]
1945	A	0.029	[6.2e-10,0.26]
1946	G	0.025	[6.2e-10,0.26]
1947	P	0.033	[6.2e-10,0.26]
1948	M	0.018	[6.2e-10,0.26]
1949	P	0.036	[6.2e-10,0.26]
1950	V	0.025	[6.2e-10,0.26]
1951	T	0.025	[6.2e-10,0.26]
1952	H	0.033	[6.2e-10,0.26]
1953	A	0.027	[6.2e-10,0.26]
1954	S	0.026	[6.2e-10,0.26]
1955	A	0.029	[6.2e-10,0.26]
1956	A	0.028	[6.2e-10,0.26]
1957	Q	0.033	[6.2e-10,0.26]
1958	R	0.023	[6.2e-10,0.26]
1959	R	0.025	[6.2e-10,0.26]
1960	G	0.025	[6.2e-10,0.26]
1961	R	0.035	[6.2e-10,0.26]
1962	I	0.19	[0.013,0.26]
1963	G	0.026	[6.2e-10,0.26]
1964	R	0.023	[6.2e-10,0.26]
1965	N	0.025	[6.2e-10,0.26]
1966	P	0.034	[6.2e-10,0.26]
1967	N	0.024	[6.2e-10,0.26]
1968	K	0.025	[6.2e-10,0.26]
1969	P	0.036	[6.2e-10,0.26]
1970	G	0.027	[6.2e-10,0.26]
1971	D	0.026	[6.2e-10,0.26]
1972	E	0.022	[6.2e-10,0.26]
1973	Y	0.034	[6.2e-10,0.26]

1974	M	0.19	[0.013,0.26]
1975	Y	0.036	[6.2e-10,0.26]
1976	G	0.027	[6.2e-10,0.26]
1977	G	0.03	[6.2e-10,0.26]
1978	G	0.024	[6.2e-10,0.26]
1979	C	0.034	[6.2e-10,0.26]
1980	A	0.025	[6.2e-10,0.26]
1981	E	0.022	[6.2e-10,0.26]
1982	T	0.029	[6.2e-10,0.26]
1983	D	0.026	[6.2e-10,0.26]
1984	E	0.024	[6.2e-10,0.26]
1985	D	0.35	[0.054,1]
1986	H	0.033	[6.2e-10,0.26]
1987	A	0.025	[6.2e-10,0.26]
1988	H	0.03	[6.2e-10,0.26]
1989	W	0.035	[6.2e-10,0.26]
1990	L	0.035	[6.2e-10,0.26]
1991	E	0.024	[6.2e-10,0.26]
1992	A	0.025	[6.2e-10,0.26]
1993	R	0.026	[6.2e-10,0.26]
1994	M	0.018	[6.2e-10,0.26]
1995	L	0.035	[6.2e-10,0.26]
1996	L	0.035	[6.2e-10,0.26]
1997	D	0.023	[6.2e-10,0.26]
1998	N	0.024	[6.2e-10,0.26]
1999	I	0.026	[6.2e-10,0.26]
2000	Y	0.032	[6.2e-10,0.26]
2001	L	0.032	[6.2e-10,0.26]
2002	Q	0.034	[6.2e-10,0.26]
2003	D	0.026	[6.2e-10,0.26]
2004	G	0.026	[6.2e-10,0.26]
2005	L	0.032	[6.2e-10,0.26]
2006	I	0.02	[6.2e-10,0.26]
2007	A	0.026	[6.2e-10,0.26]
2008	S	0.032	[6.2e-10,0.26]
2009	L	0.032	[6.2e-10,0.26]
2010	Y	0.036	[6.2e-10,0.26]
2011	R	0.035	[6.2e-10,0.26]
2012	P	0.036	[6.2e-10,0.26]
2013	E	0.022	[6.2e-10,0.26]
2014	A	0.026	[6.2e-10,0.26]
2015	D	0.024	[6.2e-10,0.26]
2016	K	0.023	[6.2e-10,0.26]
2017	V	0.025	[6.2e-10,0.26]
2018	A	0.026	[6.2e-10,0.26]
2019	A	0.026	[6.2e-10,0.26]
2020	I	0.027	[6.2e-10,0.26]

2021	E	0.022	[6.2e-10,0.26]
2022	G	0.027	[6.2e-10,0.26]
2023	E	0.022	[6.2e-10,0.26]
2024	F	0.03	[6.2e-10,0.26]
2025	K	0.023	[6.2e-10,0.26]
2026	L	0.035	[6.2e-10,0.26]
2027	R	0.023	[6.2e-10,0.26]
2028	T	0.025	[6.2e-10,0.26]
2029	E	0.022	[6.2e-10,0.26]
2030	Q	0.035	[6.2e-10,0.26]
2031	R	0.023	[6.2e-10,0.26]
2032	K	0.023	[6.2e-10,0.26]
2033	T	0.026	[6.2e-10,0.26]
2034	F	0.032	[6.2e-10,0.26]
2035	V	0.023	[6.2e-10,0.26]
2036	E	0.024	[6.2e-10,0.26]
2037	L	0.032	[6.2e-10,0.26]
2038	M	0.018	[6.2e-10,0.26]
2039	K	0.023	[6.2e-10,0.26]
2040	R	0.026	[6.2e-10,0.26]
2041	G	0.027	[6.2e-10,0.26]
2042	D	0.024	[6.2e-10,0.26]
2043	L	0.035	[6.2e-10,0.26]
2044	P	0.034	[6.2e-10,0.26]
2045	V	0.029	[6.2e-10,0.26]
2046	W	0.035	[6.2e-10,0.26]
2047	L	0.036	[6.2e-10,0.26]
2048	A	0.026	[6.2e-10,0.26]
2049	Y	0.036	[6.2e-10,0.26]
2050	Q	0.034	[6.2e-10,0.26]
2051	V	0.028	[6.2e-10,0.26]
2052	A	0.025	[6.2e-10,0.26]
2053	S	0.036	[6.2e-10,0.26]
2054	A	0.026	[6.2e-10,0.26]
2055	G	0.027	[6.2e-10,0.26]
2056	I	0.02	[6.2e-10,0.26]
2057	T	0.028	[6.2e-10,0.26]
2058	Y	0.032	[6.2e-10,0.26]
2059	T	0.026	[6.2e-10,0.26]
2060	D	0.024	[6.2e-10,0.26]
2061	R	0.026	[6.2e-10,0.26]
2062	R	0.22	[0.013,1]
2063	W	0.035	[6.2e-10,0.26]
2064	C	0.032	[6.2e-10,0.26]
2065	F	0.033	[6.2e-10,0.26]
2066	D	0.026	[6.2e-10,0.26]
2067	G	0.026	[6.2e-10,0.26]

2068	T	0.38	[0.054,1]
2069	T	0.026	[6.2e-10,0.26]
2070	N	0.024	[6.2e-10,0.26]
2071	N	0.024	[6.2e-10,0.26]
2072	T	0.026	[6.2e-10,0.26]
2073	I	0.02	[6.2e-10,0.26]
2074	M	0.17	[0.013,0.26]
2075	E	0.024	[6.2e-10,0.26]
2076	D	0.023	[6.2e-10,0.26]
2077	S	0.027	[6.2e-10,0.26]
2078	V	0.025	[6.2e-10,0.26]
2079	P	0.032	[6.2e-10,0.26]
2080	A	0.025	[6.2e-10,0.26]
2081	E	0.022	[6.2e-10,0.26]
2082	V	0.024	[6.2e-10,0.26]
2083	W	0.035	[6.2e-10,0.26]
2084	T	0.026	[6.2e-10,0.26]
2085	R	0.21	[0.013,1]
2086	Y	0.29	[0.013,1]
2087	G	0.027	[6.2e-10,0.26]
2088	E	0.022	[6.2e-10,0.26]
2089	K	0.023	[6.2e-10,0.26]
2090	R	0.026	[6.2e-10,0.26]
2091	V	0.023	[6.2e-10,0.26]
2092	L	0.032	[6.2e-10,0.26]
2093	K	0.025	[6.2e-10,0.26]
2094	P	0.031	[6.2e-10,0.26]
2095	R	0.024	[6.2e-10,0.26]
2096	W	0.035	[6.2e-10,0.26]
2097	M	0.018	[6.2e-10,0.26]
2098	D	0.025	[6.2e-10,0.26]
2099	A	0.027	[6.2e-10,0.26]
2100	R	0.024	[6.2e-10,0.26]
2101	V	0.027	[6.2e-10,0.26]
2102	C	0.035	[6.2e-10,0.26]
2103	S	0.035	[6.2e-10,0.26]
2104	D	0.026	[6.2e-10,0.26]
2105	H	0.033	[6.2e-10,0.26]
2106	A	0.024	[6.2e-10,0.26]
2107	A	0.026	[6.2e-10,0.26]
2108	L	0.035	[6.2e-10,0.26]
2109	K	0.023	[6.2e-10,0.26]
2110	S	0.033	[6.2e-10,0.26]
2111	F	0.03	[6.2e-10,0.26]
2112	K	0.024	[6.2e-10,0.26]
2113	E	0.024	[6.2e-10,0.26]
2114	F	0.032	[6.2e-10,0.26]

2115	A	0.026	[6.2e-10,0.26]
2116	A	0.029	[6.2e-10,0.26]
2117	G	0.024	[6.2e-10,0.26]
2118	K	0.024	[6.2e-10,0.26]
2119	R	0.026	[6.2e-10,0.26]
2120	G	0.027	[6.2e-10,0.26]
2121	A	0.21	[0.013,1]
2122	A	0.028	[6.2e-10,0.26]
2123	F	0.28	[0.013,1]
2124	G	0.027	[6.2e-10,0.26]
2125	V	0.025	[6.2e-10,0.26]
2126	M	0.17	[0.013,0.26]
2127	E	0.21	[0.013,1]
2128	A	0.026	[6.2e-10,0.26]
2129	L	0.035	[6.2e-10,0.26]
2130	G	0.027	[6.2e-10,0.26]
2131	T	0.026	[6.2e-10,0.26]
2132	L	0.035	[6.2e-10,0.26]
2133	P	0.032	[6.2e-10,0.26]
2134	G	0.027	[6.2e-10,0.26]
2135	H	0.03	[6.2e-10,0.26]
2136	M	0.018	[6.2e-10,0.26]
2137	T	0.026	[6.2e-10,0.26]
2138	E	0.022	[6.2e-10,0.26]
2139	R	0.024	[6.2e-10,0.26]
2140	F	0.032	[6.2e-10,0.26]
2141	Q	0.033	[6.2e-10,0.26]
2142	E	0.024	[6.2e-10,0.26]
2143	A	0.026	[6.2e-10,0.26]
2144	I	0.027	[6.2e-10,0.26]
2145	D	0.023	[6.2e-10,0.26]
2146	N	0.025	[6.2e-10,0.26]
2147	L	0.032	[6.2e-10,0.26]
2148	A	0.029	[6.2e-10,0.26]
2149	V	0.023	[6.2e-10,0.26]
2150	L	0.032	[6.2e-10,0.26]
2151	M	0.018	[6.2e-10,0.26]
2152	R	0.037	[6.2e-10,0.26]
2153	A	0.025	[6.2e-10,0.26]
2154	E	0.022	[6.2e-10,0.26]
2155	T	0.03	[6.2e-10,0.26]
2156	G	0.027	[6.2e-10,0.26]
2157	S	0.025	[6.2e-10,0.26]
2158	R	0.023	[6.2e-10,0.26]
2159	P	0.035	[6.2e-10,0.26]
2160	Y	0.033	[6.2e-10,0.26]
2161	K	0.21	[0.013,1]

2162	A	0.025	[6.2e-10,0.26]
2163	A	0.023	[6.2e-10,0.26]
2164	A	0.025	[6.2e-10,0.26]
2165	A	0.026	[6.2e-10,0.26]
2166	Q	0.29	[0.013,1]
2167	L	0.035	[6.2e-10,0.26]
2168	P	0.031	[6.2e-10,0.26]
2169	E	0.022	[6.2e-10,0.26]
2170	T	0.026	[6.2e-10,0.26]
2171	L	0.037	[6.2e-10,0.26]
2172	E	0.022	[6.2e-10,0.26]
2173	T	0.026	[6.2e-10,0.26]
2174	I	0.025	[6.2e-10,0.26]
2175	M	0.018	[6.2e-10,0.26]
2176	L	0.034	[6.2e-10,0.26]
2177	L	0.035	[6.2e-10,0.26]
2178	G	0.028	[6.2e-10,0.26]
2179	L	0.035	[6.2e-10,0.26]
2180	L	0.035	[6.2e-10,0.26]
2181	G	0.027	[6.2e-10,0.26]
2182	T	0.026	[6.2e-10,0.26]
2183	V	0.028	[6.2e-10,0.26]
2184	S	0.032	[6.2e-10,0.26]
2185	L	0.035	[6.2e-10,0.26]
2186	G	0.025	[6.2e-10,0.26]
2187	I	0.024	[6.2e-10,0.26]
2188	F	0.03	[6.2e-10,0.26]
2189	F	0.032	[6.2e-10,0.26]
2190	V	0.025	[6.2e-10,0.26]
2191	L	0.035	[6.2e-10,0.26]
2192	M	0.018	[6.2e-10,0.26]
2193	R	0.032	[6.2e-10,0.26]
2194	N	0.024	[6.2e-10,0.26]
2195	K	0.023	[6.2e-10,0.26]
2196	G	0.026	[6.2e-10,0.26]
2197	I	0.21	[0.013,1]
2198	G	0.024	[6.2e-10,0.26]
2199	K	0.023	[6.2e-10,0.26]
2200	M	0.018	[6.2e-10,0.26]
2201	G	0.026	[6.2e-10,0.26]
2202	F	0.032	[6.2e-10,0.26]
2203	G	0.027	[6.2e-10,0.26]
2204	M	0.018	[6.2e-10,0.26]
2205	V	0.025	[6.2e-10,0.26]
2206	T	0.027	[6.2e-10,0.26]
2207	L	0.035	[6.2e-10,0.26]
2208	G	0.024	[6.2e-10,0.26]

2209	A	0.026	[6.2e-10,0.26]
2210	S	0.026	[6.2e-10,0.26]
2211	A	0.025	[6.2e-10,0.26]
2212	W	0.035	[6.2e-10,0.26]
2213	L	0.033	[6.2e-10,0.26]
2214	M	0.018	[6.2e-10,0.26]
2215	W	0.035	[6.2e-10,0.26]
2216	L	0.034	[6.2e-10,0.26]
2217	S	0.032	[6.2e-10,0.26]
2218	E	0.024	[6.2e-10,0.26]
2219	I	0.027	[6.2e-10,0.26]
2220	E	0.023	[6.2e-10,0.26]
2221	P	0.032	[6.2e-10,0.26]
2222	A	0.026	[6.2e-10,0.26]
2223	R	0.026	[6.2e-10,0.26]
2224	I	0.027	[6.2e-10,0.26]
2225	A	0.025	[6.2e-10,0.26]
2226	C	0.035	[6.2e-10,0.26]
2227	V	0.025	[6.2e-10,0.26]
2228	L	0.032	[6.2e-10,0.26]
2229	I	0.027	[6.2e-10,0.26]
2230	V	0.028	[6.2e-10,0.26]
2231	V	0.023	[6.2e-10,0.26]
2232	F	0.032	[6.2e-10,0.26]
2233	L	0.036	[6.2e-10,0.26]
2234	L	0.036	[6.2e-10,0.26]
2235	L	0.035	[6.2e-10,0.26]
2236	V	0.023	[6.2e-10,0.26]
2237	V	0.024	[6.2e-10,0.26]
2238	L	0.032	[6.2e-10,0.26]
2239	I	0.02	[6.2e-10,0.26]
2240	P	0.034	[6.2e-10,0.26]
2241	E	0.022	[6.2e-10,0.26]
2242	P	0.032	[6.2e-10,0.26]
2243	E	0.023	[6.2e-10,0.26]
2244	K	0.023	[6.2e-10,0.26]
2245	Q	0.035	[6.2e-10,0.26]
2246	R	0.026	[6.2e-10,0.26]
2247	S	0.036	[6.2e-10,0.26]
2248	P	0.033	[6.2e-10,0.26]
2249	Q	0.033	[6.2e-10,0.26]
2250	D	0.024	[6.2e-10,0.26]
2251	N	0.024	[6.2e-10,0.26]
2252	Q	0.034	[6.2e-10,0.26]
2253	M	0.018	[6.2e-10,0.26]
2254	A	0.025	[6.2e-10,0.26]
2255	I	0.025	[6.2e-10,0.26]

2256	I	0.024	[6.2e-10,0.26]
2257	I	0.024	[6.2e-10,0.26]
2258	M	0.018	[6.2e-10,0.26]
2259	V	0.21	[0.013,1]
2260	A	0.025	[6.2e-10,0.26]
2261	V	0.024	[6.2e-10,0.26]
2262	G	0.027	[6.2e-10,0.26]
2263	L	0.035	[6.2e-10,0.26]
2264	L	0.035	[6.2e-10,0.26]
2265	G	0.028	[6.2e-10,0.26]
2266	L	0.035	[6.2e-10,0.26]
2267	I	0.021	[6.2e-10,0.26]
2268	T	0.028	[6.2e-10,0.26]
2269	A	0.025	[6.2e-10,0.26]
2270	N	0.026	[6.2e-10,0.26]
2271	E	0.024	[6.2e-10,0.26]
2272	L	0.033	[6.2e-10,0.26]
2273	G	0.027	[6.2e-10,0.26]
2274	W	0.035	[6.2e-10,0.26]
2275	L	0.035	[6.2e-10,0.26]
2276	E	0.023	[6.2e-10,0.26]
2277	R	0.025	[6.2e-10,0.26]
2278	T	0.026	[6.2e-10,0.26]
2279	K	0.024	[6.2e-10,0.26]
2280	S	0.24	[0.013,1]
2281	D	0.024	[6.2e-10,0.26]
2282	L	0.22	[0.013,1]
2283	G	0.44	[0.054,1]
2284	H	0.28	[0.013,1]
2285	L	0.037	[6.2e-10,0.26]
2286	M	0.018	[6.2e-10,0.26]
2287	G	0.027	[6.2e-10,0.26]
2288	R	0.023	[6.2e-10,0.26]
2289	R	0.22	[0.013,1]
2290	E	0.023	[6.2e-10,0.26]
2291	E	0.023	[6.2e-10,0.26]
2292	G	0.025	[6.2e-10,0.26]
2293	A	0.41	[0.054,1]
2294	T	0.027	[6.2e-10,0.26]
2295	M	0.97	[0.26,1]
2296	G	0.027	[6.2e-10,0.26]
2297	F	0.03	[6.2e-10,0.26]
2298	S	0.035	[6.2e-10,0.26]
2299	M	0.018	[6.2e-10,0.26]
2300	D	0.024	[6.2e-10,0.26]
2301	I	0.027	[6.2e-10,0.26]
2302	D	0.025	[6.2e-10,0.26]

2303	L	0.035	[6.2e-10,0.26]
2304	R	0.033	[6.2e-10,0.26]
2305	P	0.032	[6.2e-10,0.26]
2306	A	0.026	[6.2e-10,0.26]
2307	S	0.034	[6.2e-10,0.26]
2308	A	0.027	[6.2e-10,0.26]
2309	W	0.035	[6.2e-10,0.26]
2310	A	0.029	[6.2e-10,0.26]
2311	I	0.025	[6.2e-10,0.26]
2312	Y	0.036	[6.2e-10,0.26]
2313	A	0.026	[6.2e-10,0.26]
2314	A	0.026	[6.2e-10,0.26]
2315	L	0.035	[6.2e-10,0.26]
2316	T	0.026	[6.2e-10,0.26]
2317	T	0.03	[6.2e-10,0.26]
2318	L	0.27	[0.013,1]
2319	I	0.024	[6.2e-10,0.26]
2320	T	0.026	[6.2e-10,0.26]
2321	P	0.032	[6.2e-10,0.26]
2322	A	0.026	[6.2e-10,0.26]
2323	V	0.025	[6.2e-10,0.26]
2324	Q	0.035	[6.2e-10,0.26]
2325	H	0.033	[6.2e-10,0.26]
2326	A	0.024	[6.2e-10,0.26]
2327	V	0.025	[6.2e-10,0.26]
2328	T	0.026	[6.2e-10,0.26]
2329	T	0.029	[6.2e-10,0.26]
2330	S	0.035	[6.2e-10,0.26]
2331	Y	0.033	[6.2e-10,0.26]
2332	N	0.024	[6.2e-10,0.26]
2333	N	0.024	[6.2e-10,0.26]
2334	Y	0.032	[6.2e-10,0.26]
2335	S	0.033	[6.2e-10,0.26]
2336	L	0.037	[6.2e-10,0.26]
2337	M	0.018	[6.2e-10,0.26]
2338	A	0.023	[6.2e-10,0.26]
2339	M	0.018	[6.2e-10,0.26]
2340	A	0.026	[6.2e-10,0.26]
2341	T	0.025	[6.2e-10,0.26]
2342	Q	0.035	[6.2e-10,0.26]
2343	A	0.029	[6.2e-10,0.26]
2344	G	0.026	[6.2e-10,0.26]
2345	V	0.023	[6.2e-10,0.26]
2346	L	0.035	[6.2e-10,0.26]
2347	F	0.033	[6.2e-10,0.26]
2348	G	0.027	[6.2e-10,0.26]
2349	M	0.018	[6.2e-10,0.26]

2350	G	0.026	[6.2e-10,0.26]
2351	K	0.025	[6.2e-10,0.26]
2352	G	0.024	[6.2e-10,0.26]
2353	M	0.018	[6.2e-10,0.26]
2354	P	0.032	[6.2e-10,0.26]
2355	F	0.031	[6.2e-10,0.26]
2356	Y	1	[1,1]*
2357	A	0.94	[0.26,1]
2358	W	0.52	[0.054,1]
2359	D	0.023	[6.2e-10,0.26]
2360	F	0.49	[0.054,1]
2361	G	0.027	[6.2e-10,0.26]
2362	V	0.025	[6.2e-10,0.26]
2363	P	0.031	[6.2e-10,0.26]
2364	L	0.035	[6.2e-10,0.26]
2365	L	0.037	[6.2e-10,0.26]
2366	M	0.018	[6.2e-10,0.26]
2367	M	0.4	[0.26,1]
2368	G	0.03	[6.2e-10,0.26]
2369	C	0.032	[6.2e-10,0.26]
2370	Y	0.033	[6.2e-10,0.26]
2371	S	0.035	[6.2e-10,0.26]
2372	Q	0.035	[6.2e-10,0.26]
2373	L	0.037	[6.2e-10,0.26]
2374	T	0.026	[6.2e-10,0.26]
2375	P	0.033	[6.2e-10,0.26]
2376	L	0.035	[6.2e-10,0.26]
2377	T	0.026	[6.2e-10,0.26]
2378	L	0.036	[6.2e-10,0.26]
2379	I	0.02	[6.2e-10,0.26]
2380	V	0.024	[6.2e-10,0.26]
2381	A	0.027	[6.2e-10,0.26]
2382	I	0.024	[6.2e-10,0.26]
2383	I	0.027	[6.2e-10,0.26]
2384	L	0.035	[6.2e-10,0.26]
2385	L	0.034	[6.2e-10,0.26]
2386	V	0.023	[6.2e-10,0.26]
2387	A	0.024	[6.2e-10,0.26]
2388	H	0.03	[6.2e-10,0.26]
2389	Y	0.033	[6.2e-10,0.26]
2390	M	0.018	[6.2e-10,0.26]
2391	Y	0.033	[6.2e-10,0.26]
2392	L	0.035	[6.2e-10,0.26]
2393	I	0.024	[6.2e-10,0.26]
2394	P	0.032	[6.2e-10,0.26]
2395	G	0.027	[6.2e-10,0.26]
2396	L	0.036	[6.2e-10,0.26]

2397	Q	0.033	[6.2e-10,0.26]
2398	A	0.025	[6.2e-10,0.26]
2399	A	0.025	[6.2e-10,0.26]
2400	A	0.026	[6.2e-10,0.26]
2401	A	0.024	[6.2e-10,0.26]
2402	R	0.036	[6.2e-10,0.26]
2403	A	0.029	[6.2e-10,0.26]
2404	A	0.026	[6.2e-10,0.26]
2405	Q	0.033	[6.2e-10,0.26]
2406	K	0.023	[6.2e-10,0.26]
2407	R	0.025	[6.2e-10,0.26]
2408	T	0.025	[6.2e-10,0.26]
2409	A	0.025	[6.2e-10,0.26]
2410	A	0.029	[6.2e-10,0.26]
2411	G	0.026	[6.2e-10,0.26]
2412	I	0.024	[6.2e-10,0.26]
2413	M	0.018	[6.2e-10,0.26]
2414	K	0.023	[6.2e-10,0.26]
2415	N	0.025	[6.2e-10,0.26]
2416	P	0.034	[6.2e-10,0.26]
2417	V	0.029	[6.2e-10,0.26]
2418	V	0.024	[6.2e-10,0.26]
2419	D	0.026	[6.2e-10,0.26]
2420	G	0.027	[6.2e-10,0.26]
2421	I	0.02	[6.2e-10,0.26]
2422	V	0.023	[6.2e-10,0.26]
2423	V	0.024	[6.2e-10,0.26]
2424	T	0.03	[6.2e-10,0.26]
2425	D	0.023	[6.2e-10,0.26]
2426	I	0.027	[6.2e-10,0.26]
2427	D	0.023	[6.2e-10,0.26]
2428	T	0.026	[6.2e-10,0.26]
2429	M	0.018	[6.2e-10,0.26]
2430	T	0.026	[6.2e-10,0.26]
2431	I	0.027	[6.2e-10,0.26]
2432	D	0.023	[6.2e-10,0.26]
2433	P	0.27	[0.013,1]
2434	Q	0.29	[0.013,1]
2435	V	0.023	[6.2e-10,0.26]
2436	E	0.022	[6.2e-10,0.26]
2437	K	0.023	[6.2e-10,0.26]
2438	K	0.023	[6.2e-10,0.26]
2439	M	0.018	[6.2e-10,0.26]
2440	G	0.026	[6.2e-10,0.26]
2441	Q	0.035	[6.2e-10,0.26]
2442	V	0.023	[6.2e-10,0.26]
2443	L	0.037	[6.2e-10,0.26]

2444	L	0.032	[6.2e-10,0.26]
2445	I	0.19	[0.013,0.26]
2446	A	0.025	[6.2e-10,0.26]
2447	V	0.025	[6.2e-10,0.26]
2448	A	0.026	[6.2e-10,0.26]
2449	I	0.61	[0.26,1]
2450	S	0.033	[6.2e-10,0.26]
2451	S	0.027	[6.2e-10,0.26]
2452	A	0.028	[6.2e-10,0.26]
2453	V	0.21	[0.013,1]
2454	L	0.035	[6.2e-10,0.26]
2455	L	0.34	[0.013,1]
2456	R	0.033	[6.2e-10,0.26]
2457	T	0.027	[6.2e-10,0.26]
2458	A	0.027	[6.2e-10,0.26]
2459	W	0.29	[0.013,1]
2460	G	0.026	[6.2e-10,0.26]
2461	W	0.035	[6.2e-10,0.26]
2462	G	0.024	[6.2e-10,0.26]
2463	E	0.022	[6.2e-10,0.26]
2464	A	0.029	[6.2e-10,0.26]
2465	G	0.025	[6.2e-10,0.26]
2466	A	0.028	[6.2e-10,0.26]
2467	L	0.035	[6.2e-10,0.26]
2468	I	0.024	[6.2e-10,0.26]
2469	T	0.026	[6.2e-10,0.26]
2470	A	0.026	[6.2e-10,0.26]
2471	A	0.025	[6.2e-10,0.26]
2472	T	0.027	[6.2e-10,0.26]
2473	S	0.033	[6.2e-10,0.26]
2474	T	0.027	[6.2e-10,0.26]
2475	L	0.035	[6.2e-10,0.26]
2476	W	0.035	[6.2e-10,0.26]
2477	E	0.024	[6.2e-10,0.26]
2478	G	0.026	[6.2e-10,0.26]
2479	S	0.036	[6.2e-10,0.26]
2480	P	0.032	[6.2e-10,0.26]
2481	N	0.024	[6.2e-10,0.26]
2482	K	0.024	[6.2e-10,0.26]
2483	Y	0.032	[6.2e-10,0.26]
2484	W	0.035	[6.2e-10,0.26]
2485	N	0.024	[6.2e-10,0.26]
2486	S	0.033	[6.2e-10,0.26]
2487	S	0.035	[6.2e-10,0.26]
2488	T	0.026	[6.2e-10,0.26]
2489	A	0.026	[6.2e-10,0.26]
2490	T	0.027	[6.2e-10,0.26]

2491	S	0.035	[6.2e-10,0.26]
2492	L	0.035	[6.2e-10,0.26]
2493	C	0.033	[6.2e-10,0.26]
2494	N	0.024	[6.2e-10,0.26]
2495	I	0.025	[6.2e-10,0.26]
2496	F	0.032	[6.2e-10,0.26]
2497	R	0.025	[6.2e-10,0.26]
2498	G	0.027	[6.2e-10,0.26]
2499	S	0.027	[6.2e-10,0.26]
2500	Y	0.034	[6.2e-10,0.26]
2501	L	0.035	[6.2e-10,0.26]
2502	A	0.027	[6.2e-10,0.26]
2503	G	0.026	[6.2e-10,0.26]
2504	A	0.029	[6.2e-10,0.26]
2505	S	0.035	[6.2e-10,0.26]
2506	L	0.035	[6.2e-10,0.26]
2507	I	0.026	[6.2e-10,0.26]
2508	Y	0.033	[6.2e-10,0.26]
2509	T	0.22	[0.013,1]
2510	V	0.024	[6.2e-10,0.26]
2511	T	0.026	[6.2e-10,0.26]
2512	R	0.026	[6.2e-10,0.26]
2513	N	0.025	[6.2e-10,0.26]
2514	A	0.028	[6.2e-10,0.26]
2515	G	0.027	[6.2e-10,0.26]
2516	L	0.035	[6.2e-10,0.26]
2517	V	0.027	[6.2e-10,0.26]
2518	K	0.023	[6.2e-10,0.26]
2519	R	0.026	[6.2e-10,0.26]
2520	R	0.036	[6.2e-10,0.26]
2521	G	0.026	[6.2e-10,0.26]
2522	G	0.03	[6.2e-10,0.26]
2523	G	0.026	[6.2e-10,0.26]
2524	T	0.024	[6.2e-10,0.26]
2525	G	0.027	[6.2e-10,0.26]
2526	E	0.022	[6.2e-10,0.26]
2527	T	0.028	[6.2e-10,0.26]
2528	L	0.035	[6.2e-10,0.26]
2529	G	0.026	[6.2e-10,0.26]
2530	E	0.022	[6.2e-10,0.26]
2531	K	0.023	[6.2e-10,0.26]
2532	W	0.035	[6.2e-10,0.26]
2533	K	0.024	[6.2e-10,0.26]
2534	A	0.027	[6.2e-10,0.26]
2535	R	0.033	[6.2e-10,0.26]
2536	L	0.035	[6.2e-10,0.26]
2537	N	0.025	[6.2e-10,0.26]

2538	Q	0.033	[6.2e-10,0.26]
2539	M	0.018	[6.2e-10,0.26]
2540	S	0.032	[6.2e-10,0.26]
2541	A	0.026	[6.2e-10,0.26]
2542	L	0.035	[6.2e-10,0.26]
2543	E	0.022	[6.2e-10,0.26]
2544	F	0.03	[6.2e-10,0.26]
2545	Y	0.033	[6.2e-10,0.26]
2546	S	0.035	[6.2e-10,0.26]
2547	Y	0.033	[6.2e-10,0.26]
2548	K	0.025	[6.2e-10,0.26]
2549	K	0.023	[6.2e-10,0.26]
2550	S	0.035	[6.2e-10,0.26]
2551	G	0.026	[6.2e-10,0.26]
2552	I	0.024	[6.2e-10,0.26]
2553	T	0.027	[6.2e-10,0.26]
2554	E	0.024	[6.2e-10,0.26]
2555	V	0.023	[6.2e-10,0.26]
2556	C	0.034	[6.2e-10,0.26]
2557	R	0.025	[6.2e-10,0.26]
2558	E	0.023	[6.2e-10,0.26]
2559	E	0.022	[6.2e-10,0.26]
2560	A	0.026	[6.2e-10,0.26]
2561	R	0.033	[6.2e-10,0.26]
2562	R	0.033	[6.2e-10,0.26]
2563	A	0.026	[6.2e-10,0.26]
2564	L	0.032	[6.2e-10,0.26]
2565	K	0.023	[6.2e-10,0.26]
2566	D	0.22	[0.013,1]
2567	G	0.027	[6.2e-10,0.26]
2568	V	0.023	[6.2e-10,0.26]
2569	A	0.025	[6.2e-10,0.26]
2570	T	0.026	[6.2e-10,0.26]
2571	G	0.027	[6.2e-10,0.26]
2572	G	0.026	[6.2e-10,0.26]
2573	H	0.033	[6.2e-10,0.26]
2574	A	0.029	[6.2e-10,0.26]
2575	V	0.025	[6.2e-10,0.26]
2576	S	0.033	[6.2e-10,0.26]
2577	R	0.035	[6.2e-10,0.26]
2578	G	0.027	[6.2e-10,0.26]
2579	S	0.025	[6.2e-10,0.26]
2580	A	0.025	[6.2e-10,0.26]
2581	K	0.023	[6.2e-10,0.26]
2582	L	0.28	[0.013,1]
2583	R	0.026	[6.2e-10,0.26]
2584	W	0.035	[6.2e-10,0.26]

2585	L	0.035	[6.2e-10,0.26]
2586	V	0.21	[0.013,1]
2587	E	0.022	[6.2e-10,0.26]
2588	R	0.026	[6.2e-10,0.26]
2589	G	0.027	[6.2e-10,0.26]
2590	Y	0.033	[6.2e-10,0.26]
2591	L	0.035	[6.2e-10,0.26]
2592	Q	0.033	[6.2e-10,0.26]
2593	P	0.033	[6.2e-10,0.26]
2594	Y	0.52	[0.054,1]
2595	G	0.026	[6.2e-10,0.26]
2596	K	0.023	[6.2e-10,0.26]
2597	V	0.028	[6.2e-10,0.26]
2598	I	0.24	[0.013,1]
2599	D	0.024	[6.2e-10,0.26]
2600	L	0.034	[6.2e-10,0.26]
2601	G	0.027	[6.2e-10,0.26]
2602	C	0.035	[6.2e-10,0.26]
2603	G	0.026	[6.2e-10,0.26]
2604	R	0.026	[6.2e-10,0.26]
2605	G	0.024	[6.2e-10,0.26]
2606	G	0.026	[6.2e-10,0.26]
2607	W	0.035	[6.2e-10,0.26]
2608	S	0.026	[6.2e-10,0.26]
2609	Y	0.035	[6.2e-10,0.26]
2610	Y	0.034	[6.2e-10,0.26]
2611	A	0.23	[0.013,1]
2612	A	0.026	[6.2e-10,0.26]
2613	T	0.026	[6.2e-10,0.26]
2614	I	0.024	[6.2e-10,0.26]
2615	R	0.034	[6.2e-10,0.26]
2616	K	0.025	[6.2e-10,0.26]
2617	V	0.025	[6.2e-10,0.26]
2618	Q	0.034	[6.2e-10,0.26]
2619	E	0.023	[6.2e-10,0.26]
2620	V	0.024	[6.2e-10,0.26]
2621	K	0.38	[0.054,1]
2622	G	0.027	[6.2e-10,0.26]
2623	Y	0.032	[6.2e-10,0.26]
2624	T	0.026	[6.2e-10,0.26]
2625	K	0.023	[6.2e-10,0.26]
2626	G	0.027	[6.2e-10,0.26]
2627	G	0.028	[6.2e-10,0.26]
2628	P	0.035	[6.2e-10,0.26]
2629	G	0.029	[6.2e-10,0.26]
2630	H	0.033	[6.2e-10,0.26]
2631	E	0.024	[6.2e-10,0.26]

2632	E	0.024	[6.2e-10,0.26]
2633	P	0.033	[6.2e-10,0.26]
2634	T	0.29	[0.054,1]
2635	L	0.035	[6.2e-10,0.26]
2636	V	0.023	[6.2e-10,0.26]
2637	Q	0.035	[6.2e-10,0.26]
2638	S	0.024	[6.2e-10,0.26]
2639	Y	0.036	[6.2e-10,0.26]
2640	G	0.024	[6.2e-10,0.26]
2641	W	0.035	[6.2e-10,0.26]
2642	N	0.024	[6.2e-10,0.26]
2643	I	0.02	[6.2e-10,0.26]
2644	V	0.028	[6.2e-10,0.26]
2645	R	0.036	[6.2e-10,0.26]
2646	L	0.033	[6.2e-10,0.26]
2647	K	0.023	[6.2e-10,0.26]
2648	S	0.027	[6.2e-10,0.26]
2649	G	0.026	[6.2e-10,0.26]
2650	V	0.023	[6.2e-10,0.26]
2651	D	0.023	[6.2e-10,0.26]
2652	V	0.025	[6.2e-10,0.26]
2653	F	0.031	[6.2e-10,0.26]
2654	H	0.031	[6.2e-10,0.26]
2655	M	0.018	[6.2e-10,0.26]
2656	A	0.023	[6.2e-10,0.26]
2657	A	0.029	[6.2e-10,0.26]
2658	E	0.022	[6.2e-10,0.26]
2659	S	0.27	[0.013,1]
2660	C	0.035	[6.2e-10,0.26]
2661	D	0.023	[6.2e-10,0.26]
2662	T	0.029	[6.2e-10,0.26]
2663	L	0.035	[6.2e-10,0.26]
2664	L	0.035	[6.2e-10,0.26]
2665	C	0.035	[6.2e-10,0.26]
2666	D	0.023	[6.2e-10,0.26]
2667	I	0.02	[6.2e-10,0.26]
2668	G	0.029	[6.2e-10,0.26]
2669	E	0.022	[6.2e-10,0.26]
2670	S	0.035	[6.2e-10,0.26]
2671	S	0.035	[6.2e-10,0.26]
2672	S	0.036	[6.2e-10,0.26]
2673	S	0.027	[6.2e-10,0.26]
2674	P	0.036	[6.2e-10,0.26]
2675	E	0.024	[6.2e-10,0.26]
2676	V	0.023	[6.2e-10,0.26]
2677	E	0.024	[6.2e-10,0.26]
2678	E	0.023	[6.2e-10,0.26]

2679	A	0.22	[0.013,1]
2680	R	0.037	[6.2e-10,0.26]
2681	T	0.025	[6.2e-10,0.26]
2682	L	0.032	[6.2e-10,0.26]
2683	R	0.026	[6.2e-10,0.26]
2684	V	0.024	[6.2e-10,0.26]
2685	L	0.032	[6.2e-10,0.26]
2686	S	0.034	[6.2e-10,0.26]
2687	M	0.018	[6.2e-10,0.26]
2688	V	0.023	[6.2e-10,0.26]
2689	G	0.21	[0.013,1]
2690	D	0.024	[6.2e-10,0.26]
2691	W	0.035	[6.2e-10,0.26]
2692	L	0.035	[6.2e-10,0.26]
2693	E	0.023	[6.2e-10,0.26]
2694	K	0.21	[0.013,1]
2695	R	0.026	[6.2e-10,0.26]
2696	P	0.032	[6.2e-10,0.26]
2697	G	0.024	[6.2e-10,0.26]
2698	A	0.026	[6.2e-10,0.26]
2699	F	0.031	[6.2e-10,0.26]
2700	C	0.034	[6.2e-10,0.26]
2701	I	0.19	[0.013,0.26]
2702	K	0.023	[6.2e-10,0.26]
2703	V	0.023	[6.2e-10,0.26]
2704	L	0.035	[6.2e-10,0.26]
2705	C	0.032	[6.2e-10,0.26]
2706	P	0.032	[6.2e-10,0.26]
2707	Y	0.032	[6.2e-10,0.26]
2708	T	0.026	[6.2e-10,0.26]
2709	S	0.024	[6.2e-10,0.26]
2710	T	0.029	[6.2e-10,0.26]
2711	M	0.018	[6.2e-10,0.26]
2712	M	0.018	[6.2e-10,0.26]
2713	E	0.023	[6.2e-10,0.26]
2714	T	0.026	[6.2e-10,0.26]
2715	L	0.21	[0.013,1]
2716	E	0.022	[6.2e-10,0.26]
2717	R	0.039	[6.2e-10,0.26]
2718	L	0.035	[6.2e-10,0.26]
2719	Q	0.034	[6.2e-10,0.26]
2720	R	0.036	[6.2e-10,0.26]
2721	R	0.023	[6.2e-10,0.26]
2722	Y	0.56	[0.054,1]
2723	G	0.024	[6.2e-10,0.26]
2724	G	0.027	[6.2e-10,0.26]
2725	G	0.027	[6.2e-10,0.26]

2726	L	0.036	[6.2e-10,0.26]
2727	V	0.025	[6.2e-10,0.26]
2728	R	0.026	[6.2e-10,0.26]
2729	V	0.023	[6.2e-10,0.26]
2730	P	0.032	[6.2e-10,0.26]
2731	L	0.038	[6.2e-10,0.26]
2732	S	0.28	[0.013,1]
2733	R	0.033	[6.2e-10,0.26]
2734	N	0.024	[6.2e-10,0.26]
2735	S	0.034	[6.2e-10,0.26]
2736	T	0.026	[6.2e-10,0.26]
2737	H	0.033	[6.2e-10,0.26]
2738	E	0.022	[6.2e-10,0.26]
2739	M	0.018	[6.2e-10,0.26]
2740	Y	0.034	[6.2e-10,0.26]
2741	W	0.035	[6.2e-10,0.26]
2742	V	0.025	[6.2e-10,0.26]
2743	S	0.036	[6.2e-10,0.26]
2744	G	0.026	[6.2e-10,0.26]
2745	A	0.025	[6.2e-10,0.26]
2746	K	0.025	[6.2e-10,0.26]
2747	S	0.025	[6.2e-10,0.26]
2748	N	0.024	[6.2e-10,0.26]
2749	I	0.6	[0.26,1]
2750	I	0.02	[6.2e-10,0.26]
2751	K	0.024	[6.2e-10,0.26]
2752	S	0.027	[6.2e-10,0.26]
2753	V	0.023	[6.2e-10,0.26]
2754	S	0.033	[6.2e-10,0.26]
2755	T	0.026	[6.2e-10,0.26]
2756	T	0.025	[6.2e-10,0.26]
2757	S	0.026	[6.2e-10,0.26]
2758	Q	0.033	[6.2e-10,0.26]
2759	L	0.032	[6.2e-10,0.26]
2760	L	0.032	[6.2e-10,0.26]
2761	L	0.035	[6.2e-10,0.26]
2762	G	0.026	[6.2e-10,0.26]
2763	R	0.033	[6.2e-10,0.26]
2764	M	0.018	[6.2e-10,0.26]
2765	D	0.22	[0.013,1]
2766	G	0.024	[6.2e-10,0.26]
2767	P	0.033	[6.2e-10,0.26]
2768	R	0.023	[6.2e-10,0.26]
2769	R	0.023	[6.2e-10,0.26]
2770	P	0.032	[6.2e-10,0.26]
2771	V	0.023	[6.2e-10,0.26]
2772	K	0.025	[6.2e-10,0.26]

2773	Y	0.036	[6.2e-10,0.26]
2774	E	0.022	[6.2e-10,0.26]
2775	E	0.022	[6.2e-10,0.26]
2776	D	0.026	[6.2e-10,0.26]
2777	V	0.023	[6.2e-10,0.26]
2778	N	0.21	[0.013,1]
2779	L	0.032	[6.2e-10,0.26]
2780	G	0.026	[6.2e-10,0.26]
2781	S	0.034	[6.2e-10,0.26]
2782	G	0.026	[6.2e-10,0.26]
2783	T	0.025	[6.2e-10,0.26]
2784	R	0.038	[6.2e-10,0.26]
2785	A	0.029	[6.2e-10,0.26]
2786	V	0.023	[6.2e-10,0.26]
2787	A	0.39	[0.054,1]
2788	S	0.024	[6.2e-10,0.26]
2789	C	0.034	[6.2e-10,0.26]
2790	A	0.029	[6.2e-10,0.26]
2791	E	0.023	[6.2e-10,0.26]
2792	A	0.029	[6.2e-10,0.26]
2793	P	0.033	[6.2e-10,0.26]
2794	N	0.024	[6.2e-10,0.26]
2795	L	0.18	[0.013,0.26]
2796	K	0.023	[6.2e-10,0.26]
2797	I	0.21	[0.013,1]
2798	I	0.027	[6.2e-10,0.26]
2799	G	0.029	[6.2e-10,0.26]
2800	N	0.55	[0.26,1]
2801	R	0.033	[6.2e-10,0.26]
2802	V	0.25	[0.013,1]
2803	E	0.022	[6.2e-10,0.26]
2804	R	0.025	[6.2e-10,0.26]
2805	I	0.024	[6.2e-10,0.26]
2806	R	0.033	[6.2e-10,0.26]
2807	S	0.41	[0.054,1]
2808	E	0.023	[6.2e-10,0.26]
2809	H	0.032	[6.2e-10,0.26]
2810	A	0.025	[6.2e-10,0.26]
2811	E	0.024	[6.2e-10,0.26]
2812	T	0.025	[6.2e-10,0.26]
2813	W	0.035	[6.2e-10,0.26]
2814	F	0.03	[6.2e-10,0.26]
2815	F	0.27	[0.013,1]
2816	D	0.025	[6.2e-10,0.26]
2817	E	0.023	[6.2e-10,0.26]
2818	N	0.024	[6.2e-10,0.26]
2819	H	0.03	[6.2e-10,0.26]

2820	P	0.032	[6.2e-10,0.26]
2821	Y	0.033	[6.2e-10,0.26]
2822	R	0.023	[6.2e-10,0.26]
2823	T	0.026	[6.2e-10,0.26]
2824	W	0.035	[6.2e-10,0.26]
2825	A	0.027	[6.2e-10,0.26]
2826	Y	0.032	[6.2e-10,0.26]
2827	H	0.033	[6.2e-10,0.26]
2828	G	0.024	[6.2e-10,0.26]
2829	S	0.025	[6.2e-10,0.26]
2830	Y	0.033	[6.2e-10,0.26]
2831	E	0.21	[0.013,1]
2832	A	0.026	[6.2e-10,0.26]
2833	P	0.034	[6.2e-10,0.26]
2834	T	0.025	[6.2e-10,0.26]
2835	Q	0.035	[6.2e-10,0.26]
2836	G	0.024	[6.2e-10,0.26]
2837	S	0.035	[6.2e-10,0.26]
2838	A	0.023	[6.2e-10,0.26]
2839	S	0.035	[6.2e-10,0.26]
2840	S	0.034	[6.2e-10,0.26]
2841	L	0.033	[6.2e-10,0.26]
2842	I	0.22	[0.013,1]
2843	N	0.024	[6.2e-10,0.26]
2844	G	0.024	[6.2e-10,0.26]
2845	V	0.029	[6.2e-10,0.26]
2846	V	0.028	[6.2e-10,0.26]
2847	R	0.025	[6.2e-10,0.26]
2848	L	0.032	[6.2e-10,0.26]
2849	L	0.035	[6.2e-10,0.26]
2850	S	0.035	[6.2e-10,0.26]
2851	K	0.023	[6.2e-10,0.26]
2852	P	0.033	[6.2e-10,0.26]
2853	W	0.035	[6.2e-10,0.26]
2854	D	0.026	[6.2e-10,0.26]
2855	V	0.024	[6.2e-10,0.26]
2856	V	0.023	[6.2e-10,0.26]
2857	T	0.03	[6.2e-10,0.26]
2858	G	0.027	[6.2e-10,0.26]
2859	V	0.027	[6.2e-10,0.26]
2860	T	0.026	[6.2e-10,0.26]
2861	G	0.027	[6.2e-10,0.26]
2862	I	0.02	[6.2e-10,0.26]
2863	A	0.027	[6.2e-10,0.26]
2864	M	0.018	[6.2e-10,0.26]
2865	T	0.028	[6.2e-10,0.26]
2866	D	0.023	[6.2e-10,0.26]

2867	T	0.026	[6.2e-10,0.26]
2868	T	0.026	[6.2e-10,0.26]
2869	P	0.032	[6.2e-10,0.26]
2870	Y	0.034	[6.2e-10,0.26]
2871	G	0.027	[6.2e-10,0.26]
2872	Q	0.034	[6.2e-10,0.26]
2873	Q	0.035	[6.2e-10,0.26]
2874	R	0.026	[6.2e-10,0.26]
2875	V	0.027	[6.2e-10,0.26]
2876	F	0.03	[6.2e-10,0.26]
2877	K	0.024	[6.2e-10,0.26]
2878	E	0.024	[6.2e-10,0.26]
2879	K	0.025	[6.2e-10,0.26]
2880	V	0.023	[6.2e-10,0.26]
2881	D	0.023	[6.2e-10,0.26]
2882	T	0.028	[6.2e-10,0.26]
2883	R	0.023	[6.2e-10,0.26]
2884	V	0.023	[6.2e-10,0.26]
2885	P	0.032	[6.2e-10,0.26]
2886	D	0.025	[6.2e-10,0.26]
2887	P	0.033	[6.2e-10,0.26]
2888	Q	0.035	[6.2e-10,0.26]
2889	E	0.024	[6.2e-10,0.26]
2890	G	0.026	[6.2e-10,0.26]
2891	T	0.03	[6.2e-10,0.26]
2892	R	0.034	[6.2e-10,0.26]
2893	Q	0.28	[0.013,1]
2894	V	0.87	[0.26,1]
2895	M	0.018	[6.2e-10,0.26]
2896	N	0.21	[0.013,1]
2897	M	0.28	[0.054,1]
2898	V	0.026	[6.2e-10,0.26]
2899	S	0.035	[6.2e-10,0.26]
2900	S	0.033	[6.2e-10,0.26]
2901	W	0.035	[6.2e-10,0.26]
2902	L	0.036	[6.2e-10,0.26]
2903	W	0.035	[6.2e-10,0.26]
2904	K	0.023	[6.2e-10,0.26]
2905	E	0.022	[6.2e-10,0.26]
2906	L	0.036	[6.2e-10,0.26]
2907	G	0.026	[6.2e-10,0.26]
2908	K	0.025	[6.2e-10,0.26]
2909	H	0.27	[0.013,1]
2910	K	0.023	[6.2e-10,0.26]
2911	R	0.033	[6.2e-10,0.26]
2912	P	0.032	[6.2e-10,0.26]
2913	R	0.037	[6.2e-10,0.26]

2914	V	0.026	[6.2e-10,0.26]
2915	C	0.033	[6.2e-10,0.26]
2916	T	0.026	[6.2e-10,0.26]
2917	K	0.025	[6.2e-10,0.26]
2918	E	0.024	[6.2e-10,0.26]
2919	E	0.022	[6.2e-10,0.26]
2920	F	0.03	[6.2e-10,0.26]
2921	I	0.024	[6.2e-10,0.26]
2922	N	0.025	[6.2e-10,0.26]
2923	K	0.023	[6.2e-10,0.26]
2924	V	0.025	[6.2e-10,0.26]
2925	R	0.034	[6.2e-10,0.26]
2926	S	0.024	[6.2e-10,0.26]
2927	N	0.027	[6.2e-10,0.26]
2928	A	0.025	[6.2e-10,0.26]
2929	A	0.025	[6.2e-10,0.26]
2930	L	0.036	[6.2e-10,0.26]
2931	G	0.026	[6.2e-10,0.26]
2932	A	0.025	[6.2e-10,0.26]
2933	I	0.02	[6.2e-10,0.26]
2934	F	0.033	[6.2e-10,0.26]
2935	E	0.024	[6.2e-10,0.26]
2936	E	0.022	[6.2e-10,0.26]
2937	E	0.024	[6.2e-10,0.26]
2938	K	0.025	[6.2e-10,0.26]
2939	E	0.024	[6.2e-10,0.26]
2940	W	0.035	[6.2e-10,0.26]
2941	K	0.023	[6.2e-10,0.26]
2942	T	0.026	[6.2e-10,0.26]
2943	A	0.027	[6.2e-10,0.26]
2944	V	0.024	[6.2e-10,0.26]
2945	E	0.024	[6.2e-10,0.26]
2946	A	0.029	[6.2e-10,0.26]
2947	V	0.023	[6.2e-10,0.26]
2948	N	0.025	[6.2e-10,0.26]
2949	D	0.026	[6.2e-10,0.26]
2950	P	0.032	[6.2e-10,0.26]
2951	R	0.024	[6.2e-10,0.26]
2952	F	0.031	[6.2e-10,0.26]
2953	W	0.035	[6.2e-10,0.26]
2954	A	0.027	[6.2e-10,0.26]
2955	L	0.037	[6.2e-10,0.26]
2956	V	0.023	[6.2e-10,0.26]
2957	D	0.024	[6.2e-10,0.26]
2958	K	0.2	[0.013,1]
2959	E	0.023	[6.2e-10,0.26]
2960	R	0.026	[6.2e-10,0.26]

2961	E	0.023	[6.2e-10,0.26]
2962	H	0.031	[6.2e-10,0.26]
2963	H	0.03	[6.2e-10,0.26]
2964	L	0.035	[6.2e-10,0.26]
2965	R	0.026	[6.2e-10,0.26]
2966	G	0.027	[6.2e-10,0.26]
2967	E	0.022	[6.2e-10,0.26]
2968	C	0.035	[6.2e-10,0.26]
2969	Q	0.26	[0.013,1]
2970	S	0.024	[6.2e-10,0.26]
2971	C	0.035	[6.2e-10,0.26]
2972	V	0.023	[6.2e-10,0.26]
2973	Y	0.032	[6.2e-10,0.26]
2974	N	0.024	[6.2e-10,0.26]
2975	M	0.018	[6.2e-10,0.26]
2976	M	0.018	[6.2e-10,0.26]
2977	G	0.027	[6.2e-10,0.26]
2978	K	0.025	[6.2e-10,0.26]
2979	R	0.026	[6.2e-10,0.26]
2980	E	0.024	[6.2e-10,0.26]
2981	K	0.023	[6.2e-10,0.26]
2982	K	0.023	[6.2e-10,0.26]
2983	Q	0.035	[6.2e-10,0.26]
2984	G	0.026	[6.2e-10,0.26]
2985	E	0.024	[6.2e-10,0.26]
2986	F	0.031	[6.2e-10,0.26]
2987	G	0.025	[6.2e-10,0.26]
2988	K	0.024	[6.2e-10,0.26]
2989	A	0.025	[6.2e-10,0.26]
2990	K	0.024	[6.2e-10,0.26]
2991	G	0.026	[6.2e-10,0.26]
2992	S	0.024	[6.2e-10,0.26]
2993	R	0.033	[6.2e-10,0.26]
2994	A	0.025	[6.2e-10,0.26]
2995	I	0.024	[6.2e-10,0.26]
2996	W	0.035	[6.2e-10,0.26]
2997	Y	0.033	[6.2e-10,0.26]
2998	M	0.018	[6.2e-10,0.26]
2999	W	0.035	[6.2e-10,0.26]
3000	L	0.036	[6.2e-10,0.26]
3001	G	0.026	[6.2e-10,0.26]
3002	A	0.027	[6.2e-10,0.26]
3003	R	0.026	[6.2e-10,0.26]
3004	F	0.032	[6.2e-10,0.26]
3005	L	0.036	[6.2e-10,0.26]
3006	E	0.022	[6.2e-10,0.26]
3007	F	0.032	[6.2e-10,0.26]

3008	E	0.024	[6.2e-10,0.26]
3009	A	0.4	[0.054,1]
3010	L	0.035	[6.2e-10,0.26]
3011	G	0.026	[6.2e-10,0.26]
3012	F	0.03	[6.2e-10,0.26]
3013	L	0.035	[6.2e-10,0.26]
3014	N	0.024	[6.2e-10,0.26]
3015	E	0.022	[6.2e-10,0.26]
3016	D	0.024	[6.2e-10,0.26]
3017	H	0.031	[6.2e-10,0.26]
3018	W	0.035	[6.2e-10,0.26]
3019	M	0.018	[6.2e-10,0.26]
3020	G	0.025	[6.2e-10,0.26]
3021	R	0.026	[6.2e-10,0.26]
3022	E	0.023	[6.2e-10,0.26]
3023	N	0.024	[6.2e-10,0.26]
3024	S	0.035	[6.2e-10,0.26]
3025	G	0.027	[6.2e-10,0.26]
3026	G	0.029	[6.2e-10,0.26]
3027	G	0.029	[6.2e-10,0.26]
3028	V	0.028	[6.2e-10,0.26]
3029	E	0.024	[6.2e-10,0.26]
3030	G	0.024	[6.2e-10,0.26]
3031	L	0.036	[6.2e-10,0.26]
3032	G	0.027	[6.2e-10,0.26]
3033	L	0.036	[6.2e-10,0.26]
3034	Q	0.035	[6.2e-10,0.26]
3035	R	0.025	[6.2e-10,0.26]
3036	L	0.034	[6.2e-10,0.26]
3037	G	0.026	[6.2e-10,0.26]
3038	Y	0.036	[6.2e-10,0.26]
3039	V	0.41	[0.054,1]
3040	L	0.037	[6.2e-10,0.26]
3041	E	0.024	[6.2e-10,0.26]
3042	E	0.024	[6.2e-10,0.26]
3043	M	0.018	[6.2e-10,0.26]
3044	S	0.39	[0.054,1]
3045	R	0.52	[0.054,1]
3046	T	0.37	[0.054,1]
3047	P	0.032	[6.2e-10,0.26]
3048	G	0.027	[6.2e-10,0.26]
3049	G	0.026	[6.2e-10,0.26]
3050	K	0.21	[0.013,1]
3051	M	0.018	[6.2e-10,0.26]
3052	Y	0.035	[6.2e-10,0.26]
3053	A	0.025	[6.2e-10,0.26]
3054	D	0.026	[6.2e-10,0.26]

3055	D	0.024	[6.2e-10,0.26]
3056	T	0.027	[6.2e-10,0.26]
3057	A	0.029	[6.2e-10,0.26]
3058	G	0.026	[6.2e-10,0.26]
3059	W	0.035	[6.2e-10,0.26]
3060	D	0.023	[6.2e-10,0.26]
3061	T	0.026	[6.2e-10,0.26]
3062	R	0.033	[6.2e-10,0.26]
3063	I	0.026	[6.2e-10,0.26]
3064	S	0.026	[6.2e-10,0.26]
3065	R	0.34	[0.054,1]
3066	F	0.033	[6.2e-10,0.26]
3067	D	0.026	[6.2e-10,0.26]
3068	L	0.035	[6.2e-10,0.26]
3069	E	0.022	[6.2e-10,0.26]
3070	N	0.027	[6.2e-10,0.26]
3071	E	0.024	[6.2e-10,0.26]
3072	A	0.027	[6.2e-10,0.26]
3073	L	0.036	[6.2e-10,0.26]
3074	I	0.025	[6.2e-10,0.26]
3075	T	0.027	[6.2e-10,0.26]
3076	N	0.024	[6.2e-10,0.26]
3077	Q	0.035	[6.2e-10,0.26]
3078	M	0.018	[6.2e-10,0.26]
3079	E	0.023	[6.2e-10,0.26]
3080	K	0.21	[0.013,1]
3081	G	0.024	[6.2e-10,0.26]
3082	H	0.03	[6.2e-10,0.26]
3083	R	0.025	[6.2e-10,0.26]
3084	A	0.24	[0.013,1]
3085	L	0.035	[6.2e-10,0.26]
3086	A	0.024	[6.2e-10,0.26]
3087	L	0.035	[6.2e-10,0.26]
3088	A	0.026	[6.2e-10,0.26]
3089	I	0.22	[0.013,1]
3090	I	0.026	[6.2e-10,0.26]
3091	K	0.024	[6.2e-10,0.26]
3092	Y	0.032	[6.2e-10,0.26]
3093	T	0.026	[6.2e-10,0.26]
3094	Y	0.033	[6.2e-10,0.26]
3095	Q	0.29	[0.013,1]
3096	N	0.024	[6.2e-10,0.26]
3097	K	0.025	[6.2e-10,0.26]
3098	V	0.023	[6.2e-10,0.26]
3099	V	0.024	[6.2e-10,0.26]
3100	K	0.023	[6.2e-10,0.26]
3101	V	0.026	[6.2e-10,0.26]

3102	L	0.033	[6.2e-10,0.26]
3103	R	0.026	[6.2e-10,0.26]
3104	P	0.032	[6.2e-10,0.26]
3105	A	0.029	[6.2e-10,0.26]
3106	E	0.024	[6.2e-10,0.26]
3107	R	0.4	[0.054,1]
3108	G	0.024	[6.2e-10,0.26]
3109	K	0.024	[6.2e-10,0.26]
3110	T	0.026	[6.2e-10,0.26]
3111	V	0.028	[6.2e-10,0.26]
3112	M	0.018	[6.2e-10,0.26]
3113	D	0.023	[6.2e-10,0.26]
3114	I	0.025	[6.2e-10,0.26]
3115	I	0.026	[6.2e-10,0.26]
3116	S	0.035	[6.2e-10,0.26]
3117	R	0.026	[6.2e-10,0.26]
3118	Q	0.035	[6.2e-10,0.26]
3119	D	0.023	[6.2e-10,0.26]
3120	Q	0.034	[6.2e-10,0.26]
3121	R	0.025	[6.2e-10,0.26]
3122	G	0.024	[6.2e-10,0.26]
3123	S	0.026	[6.2e-10,0.26]
3124	G	0.026	[6.2e-10,0.26]
3125	Q	0.035	[6.2e-10,0.26]
3126	V	0.029	[6.2e-10,0.26]
3127	V	0.027	[6.2e-10,0.26]
3128	T	0.03	[6.2e-10,0.26]
3129	Y	0.035	[6.2e-10,0.26]
3130	A	0.029	[6.2e-10,0.26]
3131	L	0.033	[6.2e-10,0.26]
3132	N	0.024	[6.2e-10,0.26]
3133	T	0.026	[6.2e-10,0.26]
3134	F	0.03	[6.2e-10,0.26]
3135	T	0.026	[6.2e-10,0.26]
3136	N	0.024	[6.2e-10,0.26]
3137	L	0.035	[6.2e-10,0.26]
3138	V	0.023	[6.2e-10,0.26]
3139	V	0.023	[6.2e-10,0.26]
3140	Q	0.033	[6.2e-10,0.26]
3141	L	0.034	[6.2e-10,0.26]
3142	I	0.025	[6.2e-10,0.26]
3143	R	0.033	[6.2e-10,0.26]
3144	N	0.024	[6.2e-10,0.26]
3145	M	0.018	[6.2e-10,0.26]
3146	E	0.022	[6.2e-10,0.26]
3147	A	0.029	[6.2e-10,0.26]
3148	E	0.022	[6.2e-10,0.26]

3149	E	0.024	[6.2e-10,0.26]
3150	V	0.025	[6.2e-10,0.26]
3151	L	0.037	[6.2e-10,0.26]
3152	E	0.022	[6.2e-10,0.26]
3153	M	0.018	[6.2e-10,0.26]
3154	Q	0.29	[0.013,1]
3155	D	0.024	[6.2e-10,0.26]
3156	L	0.036	[6.2e-10,0.26]
3157	W	0.035	[6.2e-10,0.26]
3158	L	0.035	[6.2e-10,0.26]
3159	L	0.035	[6.2e-10,0.26]
3160	R	0.025	[6.2e-10,0.26]
3161	R	0.21	[0.013,1]
3162	P	0.51	[0.054,1]
3163	E	0.022	[6.2e-10,0.26]
3164	K	0.024	[6.2e-10,0.26]
3165	V	0.023	[6.2e-10,0.26]
3166	T	0.026	[6.2e-10,0.26]
3167	S	0.38	[0.054,1]
3168	W	0.035	[6.2e-10,0.26]
3169	L	0.035	[6.2e-10,0.26]
3170	Q	0.033	[6.2e-10,0.26]
3171	S	0.22	[0.013,1]
3172	N	0.025	[6.2e-10,0.26]
3173	G	0.027	[6.2e-10,0.26]
3174	W	0.035	[6.2e-10,0.26]
3175	D	0.024	[6.2e-10,0.26]
3176	R	0.22	[0.013,1]
3177	L	0.032	[6.2e-10,0.26]
3178	K	0.025	[6.2e-10,0.26]
3179	R	0.038	[6.2e-10,0.26]
3180	M	0.018	[6.2e-10,0.26]
3181	A	0.025	[6.2e-10,0.26]
3182	V	0.026	[6.2e-10,0.26]
3183	S	0.027	[6.2e-10,0.26]
3184	G	0.027	[6.2e-10,0.26]
3185	D	0.026	[6.2e-10,0.26]
3186	D	0.024	[6.2e-10,0.26]
3187	C	0.032	[6.2e-10,0.26]
3188	V	0.029	[6.2e-10,0.26]
3189	V	0.024	[6.2e-10,0.26]
3190	K	0.023	[6.2e-10,0.26]
3191	P	0.032	[6.2e-10,0.26]
3192	I	0.027	[6.2e-10,0.26]
3193	D	0.026	[6.2e-10,0.26]
3194	D	0.026	[6.2e-10,0.26]
3195	R	0.023	[6.2e-10,0.26]

3196	F	0.033	[6.2e-10,0.26]
3197	A	0.025	[6.2e-10,0.26]
3198	H	0.5	[0.054,1]
3199	A	0.39	[0.054,1]
3200	L	0.032	[6.2e-10,0.26]
3201	R	0.023	[6.2e-10,0.26]
3202	F	0.03	[6.2e-10,0.26]
3203	L	0.035	[6.2e-10,0.26]
3204	N	0.026	[6.2e-10,0.26]
3205	D	0.024	[6.2e-10,0.26]
3206	M	0.018	[6.2e-10,0.26]
3207	G	0.026	[6.2e-10,0.26]
3208	K	0.024	[6.2e-10,0.26]
3209	V	0.029	[6.2e-10,0.26]
3210	R	0.023	[6.2e-10,0.26]
3211	K	0.024	[6.2e-10,0.26]
3212	D	0.023	[6.2e-10,0.26]
3213	T	0.026	[6.2e-10,0.26]
3214	Q	0.034	[6.2e-10,0.26]
3215	E	0.023	[6.2e-10,0.26]
3216	W	0.035	[6.2e-10,0.26]
3217	K	0.025	[6.2e-10,0.26]
3218	P	0.033	[6.2e-10,0.26]
3219	S	0.033	[6.2e-10,0.26]
3220	T	0.03	[6.2e-10,0.26]
3221	G	0.027	[6.2e-10,0.26]
3222	W	0.035	[6.2e-10,0.26]
3223	S	0.37	[0.054,1]
3224	N	0.025	[6.2e-10,0.26]
3225	W	0.035	[6.2e-10,0.26]
3226	E	0.024	[6.2e-10,0.26]
3227	E	0.024	[6.2e-10,0.26]
3228	V	0.027	[6.2e-10,0.26]
3229	P	0.031	[6.2e-10,0.26]
3230	F	0.031	[6.2e-10,0.26]
3231	C	0.033	[6.2e-10,0.26]
3232	S	0.033	[6.2e-10,0.26]
3233	H	0.03	[6.2e-10,0.26]
3234	H	0.03	[6.2e-10,0.26]
3235	F	0.03	[6.2e-10,0.26]
3236	N	0.024	[6.2e-10,0.26]
3237	K	0.023	[6.2e-10,0.26]
3238	L	0.034	[6.2e-10,0.26]
3239	Y	0.5	[0.054,1]
3240	L	0.032	[6.2e-10,0.26]
3241	K	0.023	[6.2e-10,0.26]
3242	D	0.024	[6.2e-10,0.26]

3243	G	0.025	[6.2e-10,0.26]
3244	R	0.025	[6.2e-10,0.26]
3245	S	0.033	[6.2e-10,0.26]
3246	I	0.027	[6.2e-10,0.26]
3247	V	0.023	[6.2e-10,0.26]
3248	V	0.026	[6.2e-10,0.26]
3249	P	0.034	[6.2e-10,0.26]
3250	C	0.032	[6.2e-10,0.26]
3251	R	0.033	[6.2e-10,0.26]
3252	H	0.03	[6.2e-10,0.26]
3253	Q	0.035	[6.2e-10,0.26]
3254	D	0.026	[6.2e-10,0.26]
3255	E	0.024	[6.2e-10,0.26]
3256	L	0.035	[6.2e-10,0.26]
3257	I	0.027	[6.2e-10,0.26]
3258	G	0.026	[6.2e-10,0.26]
3259	R	0.039	[6.2e-10,0.26]
3260	A	0.026	[6.2e-10,0.26]
3261	R	0.035	[6.2e-10,0.26]
3262	V	0.026	[6.2e-10,0.26]
3263	S	0.035	[6.2e-10,0.26]
3264	P	0.032	[6.2e-10,0.26]
3265	G	0.024	[6.2e-10,0.26]
3266	A	0.025	[6.2e-10,0.26]
3267	G	0.027	[6.2e-10,0.26]
3268	W	0.035	[6.2e-10,0.26]
3269	S	0.024	[6.2e-10,0.26]
3270	I	0.024	[6.2e-10,0.26]
3271	R	0.033	[6.2e-10,0.26]
3272	E	0.022	[6.2e-10,0.26]
3273	T	0.03	[6.2e-10,0.26]
3274	A	0.027	[6.2e-10,0.26]
3275	C	0.034	[6.2e-10,0.26]
3276	L	0.036	[6.2e-10,0.26]
3277	A	0.025	[6.2e-10,0.26]
3278	K	0.025	[6.2e-10,0.26]
3279	S	0.035	[6.2e-10,0.26]
3280	Y	0.036	[6.2e-10,0.26]
3281	A	0.025	[6.2e-10,0.26]
3282	Q	0.034	[6.2e-10,0.26]
3283	M	0.018	[6.2e-10,0.26]
3284	W	0.035	[6.2e-10,0.26]
3285	Q	0.033	[6.2e-10,0.26]
3286	L	0.034	[6.2e-10,0.26]
3287	L	0.035	[6.2e-10,0.26]
3288	Y	0.035	[6.2e-10,0.26]
3289	F	0.03	[6.2e-10,0.26]

3290	H	0.03	[6.2e-10,0.26]
3291	R	0.22	[0.013,1]
3292	R	0.025	[6.2e-10,0.26]
3293	D	0.023	[6.2e-10,0.26]
3294	L	0.033	[6.2e-10,0.26]
3295	R	0.04	[6.2e-10,0.26]
3296	L	0.035	[6.2e-10,0.26]
3297	M	0.018	[6.2e-10,0.26]
3298	A	0.026	[6.2e-10,0.26]
3299	N	0.026	[6.2e-10,0.26]
3300	A	0.026	[6.2e-10,0.26]
3301	I	0.027	[6.2e-10,0.26]
3302	C	0.035	[6.2e-10,0.26]
3303	S	0.033	[6.2e-10,0.26]
3304	S	0.27	[0.013,1]
3305	V	0.024	[6.2e-10,0.26]
3306	P	0.032	[6.2e-10,0.26]
3307	V	0.24	[0.013,1]
3308	D	0.023	[6.2e-10,0.26]
3309	W	0.035	[6.2e-10,0.26]
3310	V	0.027	[6.2e-10,0.26]
3311	P	0.032	[6.2e-10,0.26]
3312	T	0.029	[6.2e-10,0.26]
3313	G	0.024	[6.2e-10,0.26]
3314	R	0.026	[6.2e-10,0.26]
3315	T	0.027	[6.2e-10,0.26]
3316	T	0.026	[6.2e-10,0.26]
3317	W	0.035	[6.2e-10,0.26]
3318	S	0.035	[6.2e-10,0.26]
3319	I	0.024	[6.2e-10,0.26]
3320	H	0.033	[6.2e-10,0.26]
3321	G	0.026	[6.2e-10,0.26]
3322	K	0.023	[6.2e-10,0.26]
3323	G	0.027	[6.2e-10,0.26]
3324	E	0.024	[6.2e-10,0.26]
3325	W	0.035	[6.2e-10,0.26]
3326	M	0.018	[6.2e-10,0.26]
3327	T	0.027	[6.2e-10,0.26]
3328	T	0.25	[0.013,1]
3329	E	0.023	[6.2e-10,0.26]
3330	D	0.023	[6.2e-10,0.26]
3331	M	0.018	[6.2e-10,0.26]
3332	L	0.033	[6.2e-10,0.26]
3333	V	0.18	[0.013,0.26]
3334	V	0.023	[6.2e-10,0.26]
3335	W	0.035	[6.2e-10,0.26]
3336	N	0.025	[6.2e-10,0.26]

3337	R	0.026	[6.2e-10,0.26]
3338	V	0.023	[6.2e-10,0.26]
3339	W	0.035	[6.2e-10,0.26]
3340	I	0.027	[6.2e-10,0.26]
3341	E	0.022	[6.2e-10,0.26]
3342	E	0.022	[6.2e-10,0.26]
3343	N	0.024	[6.2e-10,0.26]
3344	D	0.023	[6.2e-10,0.26]
3345	H	0.031	[6.2e-10,0.26]
3346	M	0.018	[6.2e-10,0.26]
3347	E	0.2	[0.013,0.26]
3348	D	0.023	[6.2e-10,0.26]
3349	K	0.023	[6.2e-10,0.26]
3350	T	0.027	[6.2e-10,0.26]
3351	P	0.035	[6.2e-10,0.26]
3352	V	0.026	[6.2e-10,0.26]
3353	T	0.38	[0.054,1]
3354	K	0.024	[6.2e-10,0.26]
3355	W	0.035	[6.2e-10,0.26]
3356	T	0.026	[6.2e-10,0.26]
3357	D	0.2	[0.013,0.26]
3358	I	0.027	[6.2e-10,0.26]
3359	P	0.033	[6.2e-10,0.26]
3360	Y	0.036	[6.2e-10,0.26]
3361	L	0.035	[6.2e-10,0.26]
3362	G	0.026	[6.2e-10,0.26]
3363	K	0.025	[6.2e-10,0.26]
3364	R	0.023	[6.2e-10,0.26]
3365	E	0.023	[6.2e-10,0.26]
3366	D	0.35	[0.054,1]
3367	L	0.037	[6.2e-10,0.26]
3368	W	0.035	[6.2e-10,0.26]
3369	C	0.035	[6.2e-10,0.26]
3370	G	0.026	[6.2e-10,0.26]
3371	S	0.28	[0.013,1]
3372	L	0.29	[0.013,1]
3373	I	0.02	[6.2e-10,0.26]
3374	G	0.024	[6.2e-10,0.26]
3375	H	0.03	[6.2e-10,0.26]
3376	R	0.026	[6.2e-10,0.26]
3377	P	0.033	[6.2e-10,0.26]
3378	R	0.033	[6.2e-10,0.26]
3379	T	0.027	[6.2e-10,0.26]
3380	T	0.029	[6.2e-10,0.26]
3381	W	0.035	[6.2e-10,0.26]
3382	A	0.029	[6.2e-10,0.26]
3383	E	0.022	[6.2e-10,0.26]

3384	N	0.024	[6.2e-10,0.26]
3385	I	0.025	[6.2e-10,0.26]
3386	K	0.025	[6.2e-10,0.26]
3387	D	0.21	[0.013,1]
3388	T	0.026	[6.2e-10,0.26]
3389	V	0.025	[6.2e-10,0.26]
3390	N	0.024	[6.2e-10,0.26]
3391	M	0.018	[6.2e-10,0.26]
3392	V	0.35	[0.054,1]
3393	R	0.28	[0.013,1]
3394	R	0.023	[6.2e-10,0.26]
3395	I	0.21	[0.013,1]
3396	I	0.02	[6.2e-10,0.26]
3397	G	0.03	[6.2e-10,0.26]
3398	D	0.026	[6.2e-10,0.26]
3399	E	0.024	[6.2e-10,0.26]
3400	E	0.024	[6.2e-10,0.26]
3401	K	0.21	[0.013,1]
3402	Y	0.033	[6.2e-10,0.26]
3403	M	0.17	[0.013,0.26]
3404	D	0.023	[6.2e-10,0.26]
3405	Y	0.033	[6.2e-10,0.26]
3406	L	0.037	[6.2e-10,0.26]
3407	S	0.033	[6.2e-10,0.26]
3408	T	0.026	[6.2e-10,0.26]
3409	Q	0.035	[6.2e-10,0.26]
3410	V	0.027	[6.2e-10,0.26]
3411	R	0.033	[6.2e-10,0.26]
3412	Y	0.032	[6.2e-10,0.26]
3413	L	0.035	[6.2e-10,0.26]
3414	G	0.029	[6.2e-10,0.26]
3415	E	0.023	[6.2e-10,0.26]
3416	E	0.024	[6.2e-10,0.26]
3417	G	0.024	[6.2e-10,0.26]
3418	S	0.033	[6.2e-10,0.26]
3419	T	0.026	[6.2e-10,0.26]
3420	P	0.036	[6.2e-10,0.26]
3421	G	0.027	[6.2e-10,0.26]
3422	V	0.024	[6.2e-10,0.26]
3423	L	0.035	[6.2e-10,0.26]
