# Supplementary material for: A Comprehensive Systems Biology Approach to Studying Zika Virus
Source: PLoS One. 2016 Sep 1;11(9):e0161355. doi: 10.1371/journal.pone.0161355 (PMC5008700; doi:10.1371/journal.pone.0161355)
Supplement: S6 Data — (PDF) [file pone.0161355.s006.pdf]

Protein	Codon	α	β^-	$Pr[\beta=\beta^-]$	β^+	$Pr[\beta=\beta^+]$	p-value	q-value
Capsid	7	0.009334	0.009334	0.878642	17.2277	0.121358	0.002382	0.38826
Capsid	8	0	0	0.84813	21.3324	0.15187	0.0017634	0.431151
Capsid	10	0.375586	0.0119471	0.857217	10.8653	0.142783	0.0834692	1
Capsid	11	0.0055977	0.0055977	0.945736	21.3318	0.0542638	0.0145876	1
Capsid	12	0	0	0.624636	0.0286798	0.375364	0.0831914	1
Capsid	25	0	0	0.576995	0.82531	0.423005	0.0029847	0.42569
Capsid	27	0	0	0.576005	1.3071	0.423995	0.000936	0.457681
Capsid	30	0	0	0.716718	0.0874357	0.283282	0.0487013	1
Capsid	39	0	0	0.496283	0.0249003	0.503717	0.0718621	1
Capsid	48	0	0	0.579503	0.0315306	0.420497	0.0741423	1
Capsid	63	0	0	0.496283	0.0249003	0.503717	0.0718621	1
Capsid	80	0.0089288	0.0028848	0.954049	21.3193	0.0459512	0.007818	0.892036
Capsid	95	0	0	0.684489	0.0352703	0.315511	0.0868799	1
Capsid	101	0.005899	0	0.74183	1.10888	0.25817	0.000443	0.379067
Capsid	110	0	0	0.576867	0.928589	0.423133	0.0034411	0.471151
Capsid	111	0	0	0.660539	0.0327324	0.339461	0.066616	1
Capsid	115	0	0	0.750305	0.132615	0.249695	0.0623545	1
Capsid	117	0	0	0.725625	0.111839	0.274375	0.0519813	1
Capsid	119	0	0	0.768524	0.165201	0.231476	0.0625035	1
PrP	139	0	0	0.645226	1.30909	0.354774	0.0010675	0.456734
PrP	143	0.0015544	0.0015544	0.732238	1.11403	0.267762	0.0017334	0.456425
PrP	150	0.137713	0.0071828	0.866727	21.3324	0.133273	0.0035638	0.469187
PrP	160	0	0	0.745211	0.110129	0.254789	0.0453063	1
PrP	193	0	0	0.685589	0.0322809	0.314411	0.079028	1
PrP	210	0.0089615	0.0022621	0.924657	7.83044	0.0753431	0.0157462	1
PrM	215	0	0	0.759069	0.131691	0.240931	0.0304819	1
PrM	218	0	0	0.855329	6.16799	0.144671	0.0143603	1
PrM	229	0	0	0.680983	0.0306333	0.319017	0.0923506	1
PrM	242	0	0	0.697742	0.0396921	0.302258	0.073891	1
PrM	269	0	0	0.678509	0.0383528	0.321491	0.0494857	1
PrM	286	0.0114512	0.0022967	0.931849	9.86193	0.0681507	0.0150332	1
E, gp1	292	0	0	0.810053	2.73949	0.189947	0.0100342	0.928295
E, gp1	315	0	0	0.572013	0.0306002	0.427988	0.0720272	1
E, gp1	355	0.0035971	0.0035971	0.952717	21.3322	0.0472834	0.0156768	1
E, gp1	365	0	0	0.757691	0.152128	0.242309	0.0437479	1
E, gp1	370	0	0	0.950966	21.3324	0.0490339	0.001461	0.555659
E, gp1	371	0	0	0.845317	6.80327	0.154683	0.010601	0.907179
E, gp1	380	0	0	0.845549	6.81688	0.154451	0.0087037	0.902814
E, gp1	387	0	0	0.897632	21.3324	0.102368	0.0104039	0.937175
E, gp1	399	0	0	0.665951	0.0314585	0.334049	0.082523	1
E, gp1	517	0.0318068	0.0024915	0.949098	21.3317	0.050902	0.032464	1
E, gpC	636	0	0	1.00E-09	0.0449676	1	0.0402895	1
E, stem	693	0.758696	0.0745262	0.942723	21.2937	0.0572769	0.0360833	1
E, stem	706	0	0	0.441791	0.098371	0.558209	0.0747206	1
E, stem	722	0	0	0.473232	0.228903	0.526768	0.0178922	1

E, stem	763	0	0	0.340685	0.717103	0.659315	0.0308482	1
NS1	807	0	0	1.00E-09	0.0246953	1	0.0820381	1
NS1	815	0.0152693	0.0152693	0.699437	1.51763	0.300563	0.065435	1
NS1	893	0	0	0.498657	0.173998	0.501343	0.0560806	1
NS1	898	0	0	1.00E-09	0.0693382	1	0.0847485	1
NS1	904	0.221946	0.0246266	0.946262	21.3324	0.0537384	0.0676701	1
NS1	1051	0	0	0.751189	0.112006	0.248811	0.0114472	0.932945
NS1	1066	0	0	0.740288	0.103624	0.259712	0.0665008	1
NS1	1084	0	0	1.00E-09	0.0945872	1	0.086624	1
NS1	1114	0	0	1.00E-09	0.0802103	1	0.0825419	1
NS1	1124	0	0	1.00E-09	0.0390789	1	0.0899368	1
NS1	1129	0	0	1.00E-09	0.0660982	1	0.0915276	1
NS2A	1180	0	0	0.439993	1.26238	0.560007	0.0403869	1
NS2A	1196	0.036768	0.0042057	0.612919	0.693071	0.387081	0.0707548	1
NS2A	1251	0	0	0.423304	0.0653583	0.576696	0.0320767	1
NS2A	1274	0.1492	0.0180079	0.943493	21.2686	0.0565075	0.0716831	1
NS2A	1305	0.0016351	0.0016351	0.678733	0.179151	0.321267	0.0272849	1
NS2A	1312	0.0042342	0	0.410904	0.115829	0.589096	0.0329996	1
NS2A	1327	0	0	1.00E-09	0.0569766	1	0.0903471	1
NS2B	1415	0	0	1.00E-09	0.0271521	1	0.0891381	1
NS3	1535	0	0	0.400464	0.108223	0.599536	0.0446536	1
NS3	1604	0	0	1.00E-09	0.0604106	1	0.0312123	1
NS3	1638	0.0042889	0.0042889	0.222796	0.0953429	0.777204	0.0988884	1
NS3,DEAI	1728	0.201933	0.0523296	0.941636	21.1803	0.0583643	0.008334	0.891479
NS3,DEAI	1730	1.0311	0.0220954	0.93219	21.1937	0.0678105	0.0525829	1
NS3,DEAI	1731	0.0335249	0.0142746	0.916251	21.3324	0.0837489	0.0002165	0.247004
NS3,DEAI	1733	1.55073	0.076529	0.936933	21.2427	0.0630666	0.0321261	1
NS3,DEAI	1735	0.11727	0.11727	0.936053	21.3324	0.063947	0.0108653	0.907122
NS3,DEAI	1740	1.41998	0.0736514	0.936544	21.3181	0.0634562	0.0215011	1
NS3,DEAI	1741	0.101606	0.101606	0.931801	21.3324	0.0681992	0.0069846	0.824426
NS3,DEAI	1742	1.19619	0.0031847	0.844524	21.2281	0.155476	0.0204977	1
NS3,DEAI	1745	2.51155	0.109065	0.933587	21.186	0.0664133	0.0558773	1
NS3,DEAI	1746	0.0139337	0.0139337	0.947752	21.3324	0.0522484	0.0005771	0.329234
NS3,DEAI	1795	0	0	1.00E-09	0.0976992	1	0.0769915	1
NS3, Hel	1872	1.02491	0.109488	0.880617	21.3324	0.119383	0.0004887	0.334534
NS3, Hel	1975	0	0	1.00E-09	0.0411686	1	0.0800168	1
ClvFrag	2087	0	0	0.443466	0.122588	0.556534	0.0736326	1
NS4A	2163	0	0	1.00E-09	0.0524741	1	0.0665604	1
NS4B	2357	0.840599	0.113908	0.937057	21.2275	0.0629431	0.0044056	0.558536
NS4B	2409	0	0	0.469217	0.219339	0.530783	0.0442509	1
NS4B	2455	0	0	0.715804	5.66539	0.284196	8.67E-05	0.296635
NS4B	2489	0	0	1.00E-09	0.0988295	1	0.0791836	1
ClvFrag	2541	0	0	0.511187	0.186497	0.488813	0.0617025	1
NS5, FlaJ	2671	0	0	0.48197	0.205351	0.51803	0.0693816	1
NS5, FlaJ	2724	0	0	1.00E-09	0.0984304	1	0.0857608	1
NS5, Pol	2815	0.020823	0.016105	0.672984	1.63793	0.327016	0.0607051	1

NS5, Pol	2826	0	0	0.425218	0.0894899	0.574782	0.0425081	1
NS5, Pol	2897	0	0	0.464207	0.323302	0.535793	0.0669745	1
NS5, Pol	2909	0.0077803	0.0077803	0.602106	1.04204	0.397894	0.0209076	1
NS5, Pol	2947	0	0	1.00E-09	0.0500159	1	0.0822774	1
NS5, Pol	3025	0	0	1.00E-09	0.0928735	1	0.0818143	1
NS5, Pol	3094	0.0257038	0.0257038	0.933691	19.4532	0.0663091	0.0145093	1
NS5, Pol	3097	0.143576	0.0746194	0.936107	18.8477	0.0638929	0.0589186	1
NS5, Pol	3118	0.0257659	0	0.561221	2.19121	0.438779	0.0845968	1
NS5, Pol	3125	0.0560076	0.0560076	0.927003	21.3324	0.0729974	0.0022873	0.412068
NS5, Pol	3128	0.573761	0.021002	0.924692	12.3952	0.0753079	0.0679089	1
NS5, Pol	3133	0.724804	0.0481774	0.941384	21.2239	0.0586163	0.0999335	1
NS5, Pol	3136	0.134045	0.104722	0.939978	21.2953	0.0600218	0.0016724	0.520427
NS5, Pol	3139	0.0280075	0.0280075	0.897227	10.4874	0.102773	0.0123743	0.985053
NS5, Pol	3143	0.689763	0.0798638	0.924533	17.1513	0.0754673	0.084367	1
NS5, Pol	3147	0.0822799	0.0822799	0.937656	21.3323	0.0623444	0.0525564	1
NS5, Pol	3151	0.668988	0.0632924	0.920682	21.332	0.0793184	0.0219173	1
NS5, Pol	3152	0.0364526	0.020395	0.874615	4.25645	0.125385	0.0269068	1
NS5, Pol	3155	1.27392	0.0893196	0.937055	21.196	0.0629452	0.0582744	1
NS5, Pol	3162	0.380607	0.380607	0.864281	21.3324	0.135719	0.0835248	1
NS5, Pol	3169	0.0657583	0.0657583	0.886207	9.55128	0.113793	0.0586122	1
NS5, Pol	3176	1.75841	0.0519601	0.886493	21.223	0.113507	0.0336207	1
NS5, Pol	3178	0.0143383	0.0060824	0.720209	3.01174	0.279791	0.0845373	1
NS5, Pol	3184	0	0	0.496855	1.03053	0.503145	0.0172846	1
NS5, Pol	3191	0.119685	0.0718703	0.934264	21.3319	0.0657358	0.0187548	1
NS5, Pol	3194	0.0972875	0.0972875	0.861551	4.81594	0.138449	0.0427783	1
NS5, Pol	3195	0.121266	0.121266	0.940125	21.3323	0.0598745	0.0019993	0.402569
NS5, Pol	3196	0.109525	0.0382561	0.936949	21.3324	0.0630514	0.0095911	0.938013
NS5, Pol	3198	0.779841	0.124455	0.830955	12.7448	0.169045	0.0225588	1
NS5, Pol	3199	0.772617	0.0991686	0.870304	21.3324	0.129696	0.0243543	1
NS5, Pol	3200	0.0923534	0.0923534	0.932001	21.3324	0.0679993	0.0025487	0.396548
NS5, Pol	3201	0.122107	0.122107	0.939786	21.3324	0.0602144	0.0020301	0.38606
NS5, Pol	3209	0.591826	0.0425171	0.933334	21.2994	0.0666658	0.0839293	1
NS5, Pol	3212	0.135702	0.109405	0.937902	21.3219	0.0620985	0.0174322	1
NS5, Pol	3213	0.0838775	0.0838775	0.940285	21.3324	0.0597147	0.0099305	0.944224
NS5, Pol	3220	0.0155698	0.0155698	0.93956	21.3323	0.06044	0.002288	0.391584
NS5, Pol	3222	0.0050955	0.0050955	0.522463	1.89382	0.477537	0.0914373	1
NS5, Pol	3240	0.831847	0.0703236	0.931629	21.209	0.0683709	0.0331956	1
NS5, Pol	3244	1.83773	0.100414	0.940256	21.1802	0.0597444	0.0634601	1
NS5, Pol	3255	0.0995634	0.0104837	0.932768	21.2951	0.0672317	0.0017109	0.488042
NS5, Pol	3259	1.43913	0.140503	0.92285	21.1881	0.0771496	0.0610296	1
NS5, Pol	3270	1.1799	0.109691	0.937548	21.3063	0.0624523	0.0711973	1
NS5, Pol	3272	0.124875	0.124875	0.935479	21.3324	0.0645211	0.0155056	1
NS5, Pol	3277	0.0360637	0.0360637	0.883383	4.70981	0.116617	0.0393934	1
NS5, Pol	3278	0.653352	0.055259	0.93258	15.2759	0.0674202	0.0676316	1
NS5, Pol	3280	0.123544	0.115486	0.932006	21.3324	0.0679943	0.0028592	0.42552
NS5, Pol	3283	0.29522	0.049336	0.938345	12.5574	0.0616551	0.0182662	1
NS5, Pol	3289	0.134011	0.0361508	0.869654	4.76918	0.130346	0.0501186	1

NS5, Pol	3290	0.134222	0.118391	0.92111	14.7	0.0788898	0.0187621	1
NS5, Pol	3312	0.73713	0.123201	0.939476	21.307	0.060524	0.0988572	1
NS5, Pol	3316	0.065013	0	0.826738	14.754	0.173262	0.0271966	1
NS5, Pol	3318	0.104012	0.0619282	0.930192	21.3135	0.069808	0.0161412	1
NS5, Pol	3319	0.221302	0.0375884	0.925961	12.4508	0.0740394	0.0175124	1
NS5, Pol	3324	0.130679	0.130679	0.935871	21.3324	0.064129	0.0957696	1
NS5, Pol	3325	0.0147327	0.0147327	0.901187	11.6935	0.0988131	0.0249671	1
NS5, Pol	3328	0.125805	0.125805	0.938558	21.3324	0.0614416	0.012681	0.98652
NS5, Pol	3330	0.116986	0.0824187	0.937989	21.3239	0.0620112	0.0129942	0.988428
NS5, Pol	3334	0.685629	0.120804	0.912183	11.501	0.0878166	0.06237	1
NS5, Pol	3337	0.0588329	0.0588329	0.93792	20.111	0.0620795	0.0522019	1
NS5, Pol	3338	0.081083	0.080688	0.913466	11.0733	0.0865345	0.0250435	1
NS5, Pol	3340	0.0938391	0.0763359	0.938914	21.3323	0.0610861	0.0094893	0.955349
NS5, Pol	3345	1.06363	0.127885	0.935498	21.1797	0.0645017	0.0466333	1
NS5, Pol	3366	0.13468	0.108537	0.872358	21.3322	0.127642	9.36E-05	0.160189
NS5, Pol	3370	1.73605	0.117532	0.935092	21.2833	0.0649082	0.0704995	1
NS5, Pol	3374	0.0556003	0.0556003	0.933372	18.3886	0.0666277	0.0054492	0.666167
NS5, Pol	3375	0.0066126	0.0066126	0.817038	7.49867	0.182962	0.0104369	0.91604
NS5, Pol	3376	0.0328585	0.0328585	0.943822	21.3323	0.0561784	0.0016127	0.552018
NS5, Pol	3380	1.85288	0.0626418	0.941927	21.2265	0.0580733	0.0672302	1
NS5, Pol	3382	0.0315072	0.0315072	0.940918	21.3322	0.0590824	0.0184379	1
NS5, Pol	3386	0.0083971	0.0083971	0.687398	1.72773	0.312602	0.0983546	1
NS5, Pol	3390	0.0139197	0.0139197	0.942979	21.3324	0.0570207	0.0215647	1
NS5, Pol	3392	0.166118	0.166118	0.823343	18.5098	0.176657	0.008061	0.890089
NS5, Pol	3394	0.075983	0.075983	0.941055	21.3324	0.0589446	0.0571716	1
NS5, Pol	3402	0.15252	0.0527307	0.933776	21.2672	0.0662244	0.0018092	0.412862
NS5, Pol	3406	0.0416852	0.0416852	0.922146	21.3324	0.0778536	0.0688097	1
NS5, Pol	3408	1.39644	0.0541287	0.942366	21.2499	0.0576336	0.0554422	1
NS5, Pol	3409	0.959741	0.139959	0.923446	21.2003	0.0765543	0.0608424	1
NS5, Pol	3412	0.129044	0.129044	0.891312	8.16297	0.108688	0.0902471	1
NS5, Pol	3413	0.0821719	0.0821719	0.921871	21.3324	0.0781288	0.0018299	0.391478
ClvFrag	3417	0.695183	0.10183	0.930402	21.2862	0.0695984	0.0263099	1
