# Supplementary figures and images for: A Comprehensive Systems Biology Approach to Studying Zika Virus
Source: PLoS One. 2016 Sep 1;11(9):e0161355. doi: 10.1371/journal.pone.0161355 (PMC5008700; doi:10.1371/journal.pone.0161355)

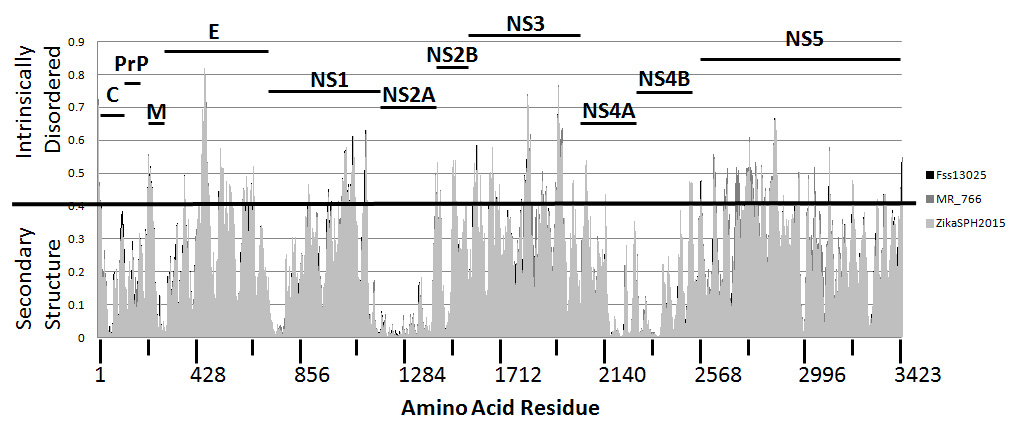

Supplement: S1 Fig — Disordered scores for 3 ZIKV strains are displayed for each amino acid site (X axis). The cutoff value between predicted disorder and structure is indicated at 0.4. The boundaries of mature proteins are indicated by black lines. Strains MR_766, Fss13025, and ZikaSPH2015 were utilized as representatives of the African, Asian, and American clades, respectively. (TIF) [file pone.0161355.s007.tif]
